# Autophagic flux inhibition potentiates disulfidptosis susceptibility in human hepatocellular carcinoma

**DOI:** 10.1038/s41419-026-09135-9

**Published:** 2026-08-11

**Authors:** Mengjiao Shi, Liangwen Yan, Kailing Hu, Lin Li, Jiayi Xu, Xinyan Li, Rongrong Liu, Yinggang Zhang, Yaping Zhao, Ying Guo, Hong Wang, Yetong Feng, Xu Gao, Enrui Xie, Fanpu Ji, Gang Wang, Pengfei Liu

**Affiliations:** 1 Department of Critical Care Medicine, National & Local Joint Engineering Research Center of Biodiagnosis and Biotherapy, The Second Affiliated Hospital of Xi’an Jiaotong University, Xi’an, China; 2 International Joint Research Center on Cell Stress and Disease Diagnosis and Therapy, National & Local Joint Engineering Research Center of Biodiagnosis and Biotherapy, The Second Affiliated Hospital of Xi’an Jiaotong University, Xi’an, China; 3 Shaanxi Provincial Clinical Research Center for Hepatic & Splenic Diseases, The Second Affiliated Hospital of Xi’an Jiaotong University, Xi’an, China; 4 Core Research Laboratory, The Second Affiliated Hospital of Xi’an Jiaotong University, Xi’an, China; 5 Department of Hepatology, the Second Affiliated Hospital of Xi’an Jiaotong University, Xi’an, China; 6 Key Laboratory of Surgical Critical Care and Life Support, Xi’an Jiaotong University, Ministry of Education of China, Xi’an, China; 7 Key Laboratory of Environment and Genes Related To Diseases, Xi’an Jiaotong University, Ministry of Education of China, Xi’an, China

**Keywords:** Drug development, Autophagy

## Abstract

Disulfidptosis is a newly recognized form of non-classical cell death, characterized by the abnormal accumulation of disulfide bonds and the collapse of the cytoskeleton. Nevertheless, the regulatory mechanisms governing this process are not fully understood. Although autophagy is a crucial cellular process necessary for maintaining homeostasis, its influence on the susceptibility to disulfidptosis and the associated mechanisms is currently unknown. This study primarily explored the interaction between autophagy and disulfidptosis in hepatocellular carcinoma (HCC) cells. In this research, both a pharmacological targeting model and a gene-modified model were established for functional and mechanistic assays. The impact of autophagy on disulfidptosis was assessed both in vitro and in vivo. Our findings revealed that the inhibition of autophagic flux significantly sensitized HCC cells to disulfidptosis through metabolic reprogramming, where the suppression of autophagy resulted in the downregulation of glucose-6-phosphate dehydrogenase (G6PD) and impaired the generation of nicotinamide adenine dinucleotide phosphate hydrogen (NADPH) and glutathione (GSH), which are critical for maintaining redox balance during disulfidptosis. Furthermore, the inhibition of autophagy also led to the suppression of mitophagy and mitochondrial dysfunction, which may further contribute to metabolic disruptions. Meanwhile, we identified endoplasmic reticulum (ER) stress as an autophagy-dependent regulator of disulfidptosis, thereby establishing a comprehensive ER stress-autophagy-disulfidptosis axis. More importantly, we repurposed the clinical drug clofazimine as a novel disruptor of the equilibrium between ER stress and autophagy, which significantly enhances disulfidptosis in preclinical HCC models. These results improve our understanding of the regulation of disulfidptosis and provide evidence that autophagy acts as a principal suppressor of disulfidptosis, suggesting that pharmacological targeting of the homeostasis between ER stress and autophagy may represent a promising therapeutic approach for the treatment of HCC.

**Figure:**
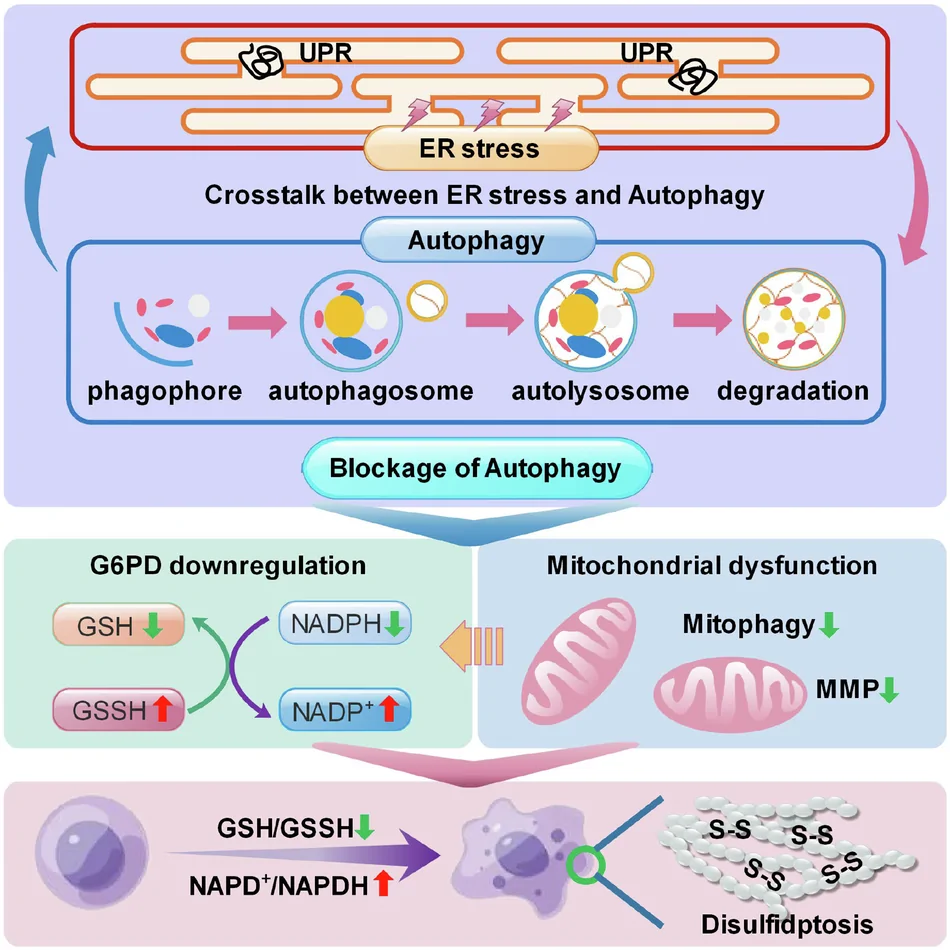

## Introduction

Programmed cell death (PCD) refers to genetically regulated, orderly processes of cell death that are critical for embryonic development, tissue homeostasis and disease defense. In 2023, a newly identified non-classical form of PCD triggered by disulfide stress, termed disulfidptosis, was discovered by scientists, and the mechanism of disulfidptosis is markedly distinct from established forms of cell death, including apoptosis, necroptosis, pyroptosis, ferroptosis, and autophagy-dependent cell death [1, 2]. Disulfidptosis is mainly characterized by actin cytoskeleton collapse caused by abnormal accumulation of intracellular disulfides, and its distinctive biochemical features offer novel potential targets for tumor and other diseases treatment. Current research highlights the major metabolic pathways involved in disulfidptosis. For example, cells import extracellular cystine via the SLC7A11 transporter, reducing it to cysteine for glutathione (GSH) synthesis and antioxidant defense. Under conditions of energy depletion or reduced reductive capacity, impaired clearance results in the abnormal accumulation of cystine and other disulfides, inducing disulfide stress, which leads to cytoskeletal protein cross-linking and structural damage [1, 2]. Meanwhile, nicotinamide adenine dinucleotide phosphate hydrogen provides reducing equivalents for GSH reduction and antioxidant reactions. Glucose starvation leads to the depletion of NADPH levels in tumor cells, resulting in the abnormal accumulation of disulfides and the induction of disulfide stress [3]. Therefore, hexokinase (HK) catalyzes the conversion of glucose to glucose-6-phosphate (G6P), which enters the pentose phosphate pathway (PPP) to generate NADPH and may hold an important position in disulfidptosis regulation. Moreover, intracellular disulfide accumulation activates the small GTPase RAC1. Activated RAC1 recruits NCKAP1, promoting the assembly of the WAVE Regulatory Complex (WRC), which includes subunits such as WAVE2, CYFIP1, ABI2, and HSPC300. This complex regulates actin (F-actin) polymerization and lamellipodia formation, playing a central role in cytoskeletal dynamics, cell migration, and the execution of disulfidptosis [1, 4]. Recently, another study has demonstrated that the induction of disulfidptosis is closely associated with endoplasmic reticulum (ER) stress. Specific ER stress inhibitors enhance disulfidptosis in SLC7A11-high tumor cells during glucose starvation, whereas some ER stress inducers suppress the process of disulfidptosis. These findings suggest a potentially protective role of ER stress in disulfidptosis, although the precise mechanisms require further investigation [5].

Autophagy is a highly conserved degradation mechanism activated in response to various stimuli, such as energy deprivation, oxidative stress, and pathogen invasion. Autophagic flux involves the generation of double-membrane autophagosomes, which encapsulate substrates such as proteins, organelles (e.g., mitochondria and ER), and microorganisms. These autophagosomes are subsequently transported to lysosomes for fusion, resulting in the formation of autolysosomes. Within these autolysosomes, the sequestered substrates are degraded into recyclable small molecules, including amino acids and monosaccharides, thereby facilitating the recycling of cellular components [6–8]. The core function of autophagy is to maintain cellular homeostasis. In recent years, researchers have uncovered that its regulation of various PCD pathways demonstrates a remarkable level of complexity and dynamism. For example, in autophagic cell death, excessive autophagy directly induces cell death [9, 10]. In ferroptosis, autophagy accelerates cell destruction by dysregulating iron homeostasis and promoting lipid peroxide accumulation [11, 12]. In pyroptosis, autophagy negatively regulates inflammasome assembly and mitigates gasdermin D (GSDMD)-dependent pore formation, thereby suppressing cell death [13]. These findings indicate that autophagy might exert a bidirectional regulatory effect in diverse PCD models. Furthermore, an intricate and bidirectional regulatory interplay exists between autophagy and ER stress. Both processes collaborate to maintain cellular homeostasis and play critical roles in tumorigenesis. On one hand, autophagy alleviates ER stress by degrading misfolded proteins, acting as a complementary mechanism to the unfolded protein response (UPR) for restoring ER homeostasis. It can also directly mitigate ER stress by removing specific stressors. On the other hand, ER stress is capable of activating numerous associated functional proteins of autophagy. Upon activation, autophagy decreases the level of ER stress via multiple pathways [14–16]. This feedback regulatory mechanism establishes the homeostasis between ER stress and autophagy by dynamically balancing stress responses and autophagic activities.

Based on the conceptual framework, a key emerging question is whether autophagy is involved in regulating disulfidptosis. While the precise relationship remains to be clarified, there is growing speculation that autophagy may intersect with disulfidptosis through several molecular pathways. Disulfidptosis is tightly linked to SLC7A11-dependent cystine uptake and abnormal disulfide accumulation resulting in redox imbalance and cross-linking of cytoskeletal proteins. These processes are all potentially subject to autophagic regulation. For instance, autophagy has been reported to regulate SLC7A11 expression or turnover, potentially influencing cellular cystine uptake. Blockage of autophagy results in the inactivation of SLC7A11 [17, 18]. Additionally, the redox environment represents a crucial determinant for the formation of disulfide bonds [19]. Autophagy is also involved in the maintenance of cellular redox homeostasis by regulating the production and scavenging of reactive oxygen species (ROS), which may affect the redox conditions necessary for disulfide bond formation [20–22]. Furthermore, a complex interplay exists between the formation of F-actin stress fibers and the autophagy pathway, and autophagy can influence actin cytoskeleton remodeling by modulating cytoskeletal-associated proteins, which could in turn affect disulfidptosis susceptibility [23, 24]. Consequently, a strong relationship should exist between autophagy and disulfidptosis.

As the predominant type of liver cancer, hepatocellular carcinoma (HCC) has now emerged as one of the most lethal cancers. Human HCC is generally diagnosed at a late stage and is characterized by a significant level of drug resistance to chemotherapy, which diminishes the efficacy of treatment and raises the likelihood of recurrence post-therapy. Consequently, it is crucial to devise strategies to counteract drug resistance in the management of HCC. In recent years, the advancement of innovative cell death models, such as ferroptosis and disulfidptosis, has opened up promising pathways for addressing drug-resistant HCC in both preclinical and clinical environments. In this research, our findings primarily suggest that the blockage of autophagic flux facilitates disulfidptosis in human HCC cells. The inhibition of autophagy led to a reduction in the expression of glucose-6-phosphate dehydrogenease (G6PD), which is the rate-limiting enzyme responsible for catalyzing the initial step of the PPP. Both GSH and NADPH metabolism were affected by autophagy within the disulfidptosis model. Furthermore, the obstruction of autophagic flux also caused mitophagy suppression and mitochondrial dysfunction, thereby increasing the susceptibility of HCC to disulfidptosis. Importantly, our findings further indicated that the impact of ER stress on disulfidptosis was dependent on the regulation of autophagy. The clinical drug, clofazimine (CFZ), was identified as a potential agent targeting the homeostasis between ER stress and autophagy, thereby enhancing susceptibility to disulfidptosis and presenting a novel therapeutic approach against HCC in clinical settings.

## Materials and methods

### Cell lines and culture

The human HCC cell line, MHCC97-H was obtained from the China Center for Type Culture Collection (CCTCC, China), and Hep 3B cell line was sourced from the American Type Culture Collection (ATCC, USA). All cell lines were maintained under standardized culture conditions: 37 °C in a 5% CO_2_ humidified atmosphere using High-Glucose DMEM supplemented with 10% fetal bovine serum (FBS), 100 U/mL penicillin, and 100 μg/mL streptomycin. Routine maintenance involved medium replacement every 48 hours and subculturing at 3–4 day intervals using standard trypsinization protocols.

In this study, CRISPR/Cas9-mediated knockout of *ATG7* was performed using established procedures from prior research, while transient gene silencing of *G6PD* was achieved through validated siRNA transfection according to previously published methodologies [25, 26]. The human *G6PD*-specific siRNA (RY204682) was obtained from GENECARER, China. The transfection of the siRNA was conducted using Lipofectamine 3000 solution (Thermo Fisher Scientific) in accordance with the manufacturer’s guidelines. The sgRNA sequences (5’-3’) targeting the human *ATG7* gene used are as follows:

Human *ATG7*-sgRNA-1: 5’-TGAACGAGGAGTACGAGACG-3’;

Human *ATG7*-sgRNA-2: 5’-GGTGCACAAATGGAGTCCAG-3’

### Chemicals

The chemicals utilized in this study included the small-molecule autophagy inhibitors Autophinib (HY-101920) and Chloroquine (HY-17589A), the small-molecule ER stress inhibitors Melatonin (HY-B0075) and 4-Phenylbutyric Acid (HY-A0281), the small-molecule activator Cinchonine (HY-Y0152), G6PD activator AG1 (HY-123962) as well as Clofazimine (HY-B1046) and the glucose transport receptor inhibitor BAY-876 (HY-100017). All these compounds were procured from MCE. Food and Drug Administration (FDA)-approved Drug Library (L1300, Selleckchem) was used for drug screen work in our study.

### Cell viability assay

To assess cell death across experimental groups, samples were incubated with propidium iodide (PI, Catalog No. 40710ES03, YEASEN), a membrane-impermeable DNA intercalating agent, for 15 minutes at room temperature in the dark. After three washes with phosphate-buffered saline (PBS) to remove unbound dye, PI-stained cells were quantitatively analyzed via fluorescence microscopy (Zeiss). Furthermore, cell viability was evaluated using the Cell Counting Kit-8 (CCK-8, Dojindo). In summary, 10 μl of CCK-8 solution was introduced into each well of a 96-well microplate, which contained 100 μl of medium. The microplate was then incubated at 37 °C for a duration of 2 hours. Afterwards, the optical density (OD) values at 450 nm were recorded to assess cell viability (*n* = 3).

### Real-time qRT-PCR

Real-time quantitative reverse transcription PCR (qRT-PCR) analysis was conducted in strict accordance with the methodologies established in previous studies [27, 28]. Total RNA was isolated using TRIzol reagent (Invitrogen), and 1 μg of RNA was subsequently reverse-transcribed into complementary DNA templates. The quantification procedure utilized *GAPDH* as an endogenous control for data normalization, with each sample being analyzed in triplicate to ensure technical reproducibility. The primer pairs employed for amplification were designed based on the following nucleotide sequences (5’-3’):

Human-*G6PD*-F: CGAGGCCGTCACCAAGAAC

Human-*G6PD*-R: GTAGTGGTCGATGCGGTAGA

Human-*PGD*-F: ATGGCCCAAGCTGACATCG

Human-*PGD*-R: AAAGCCGTGGTCATTCATGTT

Human-*PGLS*-F: GGAGCCTCGTCTCGATGCTA

Human-*PGLS*-R: GAGAGAAGATGCGTCCGGT

Human-*RPE*-F: AAGCCAGAACAGTGGGTAAAGC

Human-*RPE*-R: CAAGGCCAACCTGCAATGG

Human-*RPIA*-F: AGTGCTGGGAATTGGAAGTGG

Human-*RPIA*-R: GGGAATACAGACGAGGTTCAGA

Human-*TKT*-F: TCCACACCATGCGCTACAAG

Human-*TKT*-R: CAAGTCGGAGCTGATCTTCCT

Human-*TALDO1*-F: CTCACCCGTGAAGCGTCAG

Human-*TALDO1*-R: GTTGGTGGTAGCATCCTGGG

Human-*GAPDH*-F: CTGACTTCAACAGCGACACC

Human-*GAPDH*-R: TGCTGTAGCCAAATTCGTTGT

### Western blot

Western blot analysis was performed in accordance with established protocols, with necessary methodological modifications incorporated as required [29]. Specifically, cellular proteins were extracted and solubilized in SDS sample buffer containing 50 mM Tris-HCl (pH 6.8), 2% SDS, 100 mM DTT, 10% glycerol, and 0.1% bromophenol blue. Meanwhile, cell samples harvested using SDS sample buffer without DTT were applied for a non-reducing Western blot to examine those high-molecular-weight aggregates of various cytoskeletal proteins (DREBRIN and FLNA) during the glucose starvation process. The protein lysates were subsequently resolved by electrophoresis on 8% or 10% SDS-PAGE gels and then transferred electrophoretically onto nitrocellulose membranes. The membranes were blocked using 5% non-fat milk in Tris-buffered saline with 0.1% Tween-20 (TBST) for 1 hour at room temperature, followed by incubation overnight at 4 °C with specific primary antibodies. After three washes with TBST, the membranes were incubated with species-matched horseradish peroxidase (HRP)-conjugated secondary antibodies for 1 hour at room temperature. Protein signals were detected using a SuperSignal West Femto chemiluminescent substrate (Thermo Fisher Scientific, USA) and quantified using a ChemiDoc MP imaging system (Bio-Rad Laboratories, USA). The following antibodies were employed in this study: anti-p62 (1:1000; Proteintech, 18420-1-AP), anti-LC3 (1:1000; Proteintech, 14600-1-AP), anti-ATG7 (1:1000; Proteintech, 10088-2-AP), anti-Beclin1 (1:1000; Proteintech, 11306-1-AP), anti-FLNA (1:3000; Proteintech, 67133-1-Ig), anti-DREBRIN (1:3000; Proteintech, 10260-1-AP), anti-ATF4 (1:1000; Proteintech, 10835-1-AP), anti-ElF2S1 (1:1000; Proteintech, 11170-1-AP), anti-Phospho-ElF2S1 (1:1000; Proteintech, 28740-1-AP), anti-G6PD (1:1000; Proteintech, 25413-1-AP), anti-GAPDH (1:3000; Proteintech, 10494-1-AP), anti-mouse IgG (HRP-conjugated; 1:3000; ABclonal, AS003) and anti-rabbit IgG (HRP-conjugated; 1:3000; ABclonal, AS014). All original images of western blots have been supplied in Supplementary Materials.

### Network pharmacology analysis

The bioavailable molecular targets of CFZ were systematically predicted through structural analysis employing the SwissTargetPrediction database [30] and TargetNet platform [31]. Following structural alignment, consensus targets identified across both databases underwent functional clustering analysis via Metascape [32]. Concurrently, HCC-associated genetic variations were comprehensively annotated using the DisGeNET database [33], enabling systematic integration of CFZ targets with documented HCC pathomechanisms. Protein-protein interaction (PPI) networks were subsequently constructed through the STING database (version 11.5) to elucidate target interconnectivity [34].

### RNA sequencing

Cellular RNA extraction was systematically performed across experimental cohorts (CFZ-treated vs. untreated controls) using TRIzol reagent (Invitrogen), with subsequent polyadenylated mRNA selection using oligo(dT)-conjugated magnetic beads. RNA integrity was verified through microfluidic analysis prior to library preparation. Strand-specific sequencing libraries were constructed in biological triplicate employing the NEBNext Ultra RNA Library Prep Kit for Illumina (NEB) according to the manufacturer’s specifications. Final library quality control was performed using an Agilent 2100 Bioanalyzer. High-throughput sequencing was conducted on an Illumina NovaSeq 6000 platform provided by Novogene Co., Ltd. (Beijing, China). The raw data have been submitted to the GEO database (GSE327595 and GSE327600).

### Metabolomics

Metabolomic profiling was performed on HCC models under differential CFZ exposure conditions (treated versus untreated cohorts), employing a comprehensive and systematic analytical approach. In our research, the samples for the metabolomics assay were prepared by Cosmos Wisdom Biomedical Tech Co., LTD, in Hangzhou, China. Herein, various metabolites were separated utilizing a Waters ACQUITY BEH C18 Column (1.7 µm × 2.1 mm × 100 mm) on a Vanquish Flex UPLC. Then, the detection and identification of metabolites were conducted using the XCMS and Spectra R package.

### Analysis of glutamate, cysteine, and glycine

This study assessed the levels of glutamate, cysteine, and glycine to investigate the functionality of system Xc^−^ as well as the metabolism of amino acids. All cell or tissue samples were prepared utilizing the Glycine Assay Kit (ELK Biotechnology, ELK8276), Glutamate Assay Kit (Sangon Biotech, D799585-0050), and Cysteine Assay Kit (Solarbio, BC0185). The concentrations of glycine, cysteine, and glutamate in the various groups were determined following the manufacturer’s guidelines.

### Evaluation of NADPH metabolism and GSH metabolism

This study also aimed to evaluate NADPH metabolism and GSH metabolism across different groups by measuring the levels of NADP^+^/NADPH and GSH/GSSH. In this analysis, cell or tissue samples were prepared using NADP^+^/NADPH Assay Kit (Beyotime Biotechnology, S0180S) and GSH and GSSG Assay Kit (Beyotime Biotechnology, S0053). The levels of NADP^+^/NADPH and GSH/GSSH in the various groups were calculated in accordance with the manufacturer’s instructions.

### Cytoskeleton staining

Cellular architecture analysis was conducted using Actin-Tracker Red-555 solution (Beyotime) in accordance with the manufacturer’s guidelines. The experimental groups were fixed with 3.7% paraformaldehyde in PBS for 20 minutes at 25 °C first. Then, permeabilization was performed using PBS containing 0.1% Triton X-100/PBS solution (three cycles of 5 minutes each), and fluorescent labeling was carried out using a staining solution (diluted 1:200 in PBS containing 0.1% Triton X-100/PBS solution, incubated for 30 minutes under dark conditions). Moreover, the nuclear counterstaining was achieved using DAPI (1 μg/mL, 5 minutes). After staining, specimens were subjected to three thorough PBS washes (5 minutes per wash). Finally, high-resolution imaging was subsequently acquired using a fluorescence microscope (Zeiss).

### Measurement of mitochondrial membrane potential

To evaluate mitochondrial membrane potential (MMP), we utilized the Mitochondrial Membrane Potential Assay Kit (Beyotime) in current work. Specifically, cells from different experimental groups were incubated with the JC-1 working solution for 30 minutes at 37°C under light-protected conditions. Following incubation, the cells were washed twice with PBS. Then, fluorescence intensity measurements were subsequently conducted using a BD FACSAria II flow cytometer. The acquired data were analyzed using FlowJo software (version 7.6.1).

### Transmission electron microscopy

For transmission electron microscopy (TEM) analysis, MHCC97-H cells were seeded in D60 dishes and treated with different compounds for 16 hours under standard conditions, followed by glucose starvation for 6–8 hours. Subsequently, the cells were fixed with a solution containing 2.5% glutaraldehyde and 2% paraformaldehyde (PFA) in 0.1 M PIPES buffer (pH 7.4) for 1 hour at room temperature. After sequential buffer washes, the samples were post-fixed with 1% osmium tetroxide to enhance contrast. The specimens were then subjected to block staining with 2% uranyl acetate. Following deionized water (DIW) rinses, the specimens were dehydrated through a graded ethanol series (30–100%). Ultrathin sections were prepared and analyzed using a transmission electron microscope.

### Live cell immunofluorescence microscopy

To assess the impact of chemical compounds on autophagic flux, the ptf-LC3 vector (mRFP-GFP-LC3 reporter construct) was employed in live cell immunofluorescence. After transfecting MHCC97-H cells with ptf-LC3 vectors using Lipofectamine 3000 solution (Thermo Fisher Scientific), the HCC cells were exposed to chemical compounds for 16 hours. Following this, both treated and untreated cell samples were imaged using a Zeiss fluorescence microscope.

### Cell apoptosis analysis and ROS assay

In our study, apoptosis rates were measured utilizing the Annexin V-FITC/PI Apoptosis Detection Kit (Pumoke) in accordance with the manufacturer’s guidelines. In summary, the harvested cells underwent two washes with ice-cold PBS and were then resuspended in 1× Binding Buffer to achieve a final concentration of 1 × 10^6^ cells/mL. A 100 μL sample of the cell suspension was placed into a flow tube, followed by the addition of 5 μL of Annexin V-FITC solution. The resulting mixture was incubated in the dark at room temperature for a duration of 10 minutes. Afterwards, 10 μL of PI solution was introduced 5 minutes before detection. The percentage of apoptotic cells, encompassing both early and late apoptosis, was evaluated through flow cytometry.

In addition, the generation of cellular ROS across various groups was assessed utilizing the Reactive Oxygen Species Assay Kit (Solarbio) in accordance with the manufacturer’s instructions. In brief, liver cells were treated with DCFH-DA (10 μM) for a duration of 20 minutes at 37 °C. Subsequently, the cells underwent three washes with PBS and were dissociated into individual cells. Ultimately, the cells were analyzed using a FACSAria II Flow Cytometer (BD Biosciences). The FlowJo 7.6.1 software was employed to evaluate the results of cellular ROS.

### Animal studies

All animal experiments were formally approved by the Biomedical Ethics Committee of the Health Science Center at Xi’an Jiaotong University (Approval Number: 2022–1371) on June 9, 2022. SCID Beige Mice, obtained from Charles River Laboratories, were utilized for the xenograft mouse model in this study. In this context, MHCC97-H cells were employed for the xenograft, and the 8-week-old male mice (weighing 22–24 g) were inoculated with a cancer cell suspension containing 5 × 10^6^ cells per mouse (i.h.). The mice were randomly categorized into four groups: Ctrl, CQ, BAY-876, and BAY-876 + CQ. Tumor dimensions were measured using a vernier caliper, and the tumor volume was calculated using the formula: Volume = *π*/6 × Length × Width^2^. In this study, both CQ (75 mg/kg) and BAY-876 (3 mg/kg) were formulated in a 5% DMSO/corn oil solution and administered intraperitoneally to the mice twice a week for a total duration of 6 weeks. After this treatment period, the mice were euthanized, and the tumor weight and metabolism were assessed across different groups (*n* = 5).

### Statistical analysis

In this study, the results are expressed as mean values ± standard deviations (SD). All statistical analyses were conducted using GraphPad Prism software. The normality of the data distribution was assessed by evaluating skewness and kurtosis metrics. For comparisons between two experimental groups, a two-tailed Student’s *t* test was utilized, whereas one-way analysis of variance followed by Tukey’s post hoc test was employed for multi-group comparisons. Statistical significance was set at *P* < 0.05.

## Results

### Autophagy blockage potentiates disulfidptosis susceptibility in human HCC

**Fig. 1:**
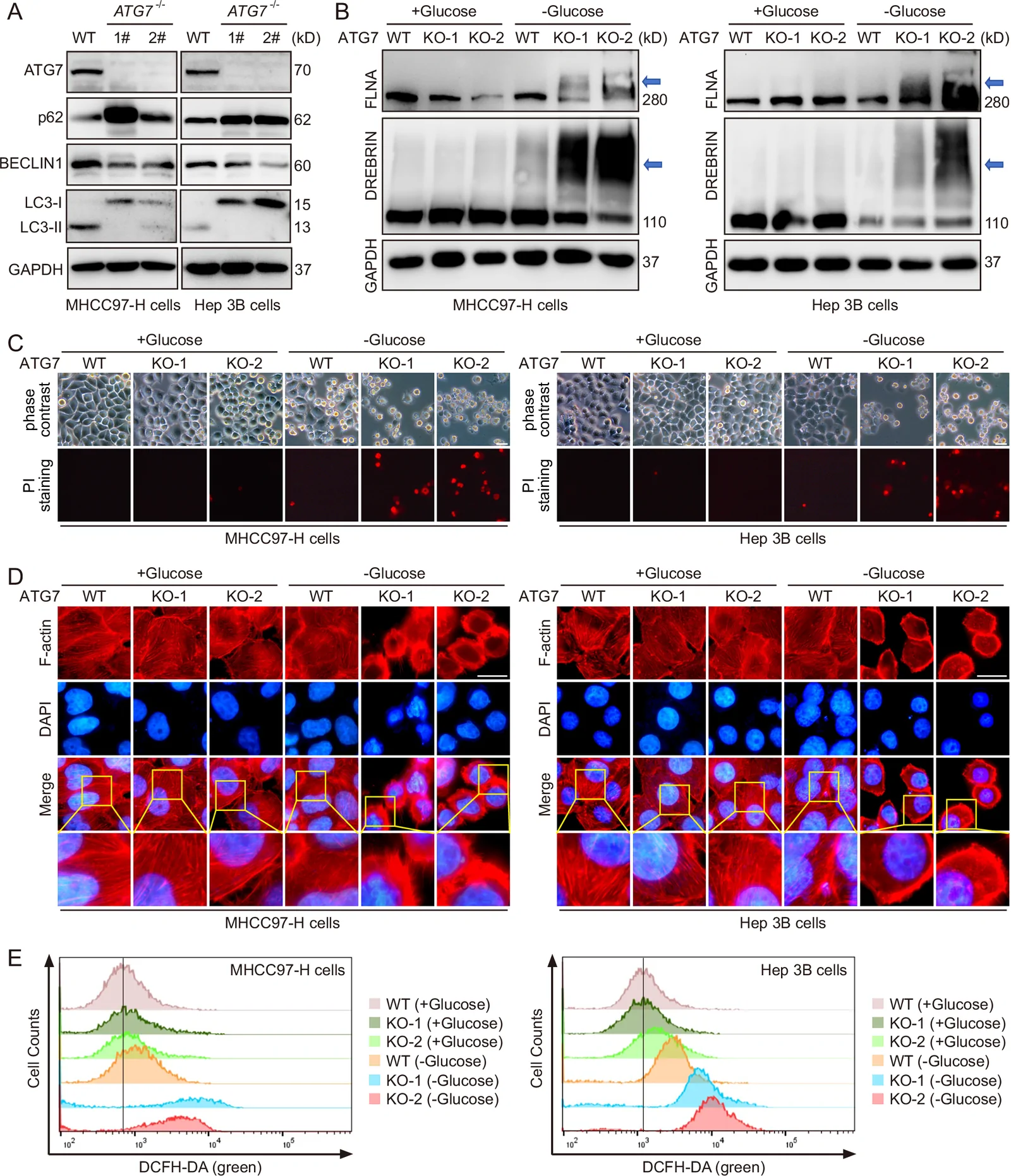
*ATG7* knockout potentiates disulfidptosis susceptibility in Human HCC. The *ATG7* knockout HCC cell lines (MHCC97-H and Hep 3B) were created utilizing the CRISPR/Cas9 technique, and the effectiveness of gene knockout was verified through Western blot analysis (**A**). In addition, both the wild type (WT) and *ATG7* knockout (KO) cells were initially subjected to glucose starvation (6–8 hours) to induce disulfidptosis. Subsequently, the formation of disulfide bonds in cytoskeletal proteins (FLNA and DREBRIN) induced by glucose starvation was analyzed using a non-reducing Western blot (**B**). Meanwhile, the changes in morphology and cell death were assessed in each group (**C** Scale bar = 20 μm). Furthermore, phalloidin staining was performed to examine the F-actin across different groups (**D** Scale bar = 10 μm). Finally, the ROS levels in different groups were evaluated via DCFH-DA staining and flow cytometry analysis (**E**).

To evaluate the position of autophagy in disulfidptosis regulation, *ATG7* knockout HCC cell lines (MHCC97-H and Hep 3B) were established in the current work. Compared with wild-type cells, the protein level of p62, an autophagy marker, was increased in *ATG7* knockout cells, together with increased LC3-I and decreased LC3-II and BECLIN1 (Fig. 1A), suggesting the blockage of autophagy in *ATG7* knockout HCC cells. Then, both wild-type and *ATG7* knockout cells were exposed to a glucose deprivation condition (6–8 hours) to trigger disulfidptosis, and the formation of disulfide bonds in cytoskeletal proteins was assessed using a non-reducing Western blot. Our findings indicated that *ATG7* knockout significantly heightened the susceptibility of HCC cells to glucose deprivation-induced disulfidptosis, and both FLNA and DREBRIN displayed markedly slower migration with smears in *ATG7* knockout cells compared with wild-type cells (Fig. 1B, C). Moreover, phalloidin staining was utilized to assess the actin filament (F-actin) in various groups. Under normal conditions, we observed that F-actin was predominantly organized in the cell cortex and stress fibers. In contrast, morphological alterations were caused by glucose deprivation, characterized by F-actin contraction and marginal clustering, which could be intensified by *ATG7* knockout-induced autophagy blockage (Fig. 1D). Additionally, the level of ROS was measured utilizing the DCFH-DA staining technique. The findings revealed that glucose deprivation elevated the ROS levels in HCC cells, while the autophagy blockage facilitated disulfidptosis alongside increased ROS levels in HCC cells (Fig. 1E). We further facilitated the overexpression of *ATG7* in HCC cells through pCMV-ATG7 transfection. The effect of overexpression was validated by western blot analysis, which revealed that the upregulation of ATG7 led to increased levels of BECLIN1 and LC3, while simultaneously decreasing the level of p62 (Fig. S1A). This indicates that the overexpression of *ATG7* enhances the autophagy process. Subsequently, HCC cells were subjected to glucose deprivation conditions to induce disulfidptosis, and our findings demonstrated that *ATG7* overexpression mitigated the vulnerability of HCC cells to glucose deprivation-induced disulfidptosis (Fig. S1B). The formation of disulfide bonds in cytoskeletal proteins was evaluated using non-reducing western blot techniques. Our results suggested that *ATG7* overexpression conferred protection to HCC cells against disulfidptosis, with both FLNA and DREBRIN exhibiting significantly slower migration and smears in the normal group compared to the *ATG7* overexpression group (Fig. S1C). Furthermore, phalloidin staining results indicated that *ATG7* overexpression alleviated F-actin contraction and marginal clustering induced by glucose deprivation (Fig. S1D), suggesting that the activation of autophagy reduces the susceptibility of HCC cells to disulfidptosis. The ROS levels in different groups were consistent with the cell death tendency (Fig. S1E).

**Fig. 2:**
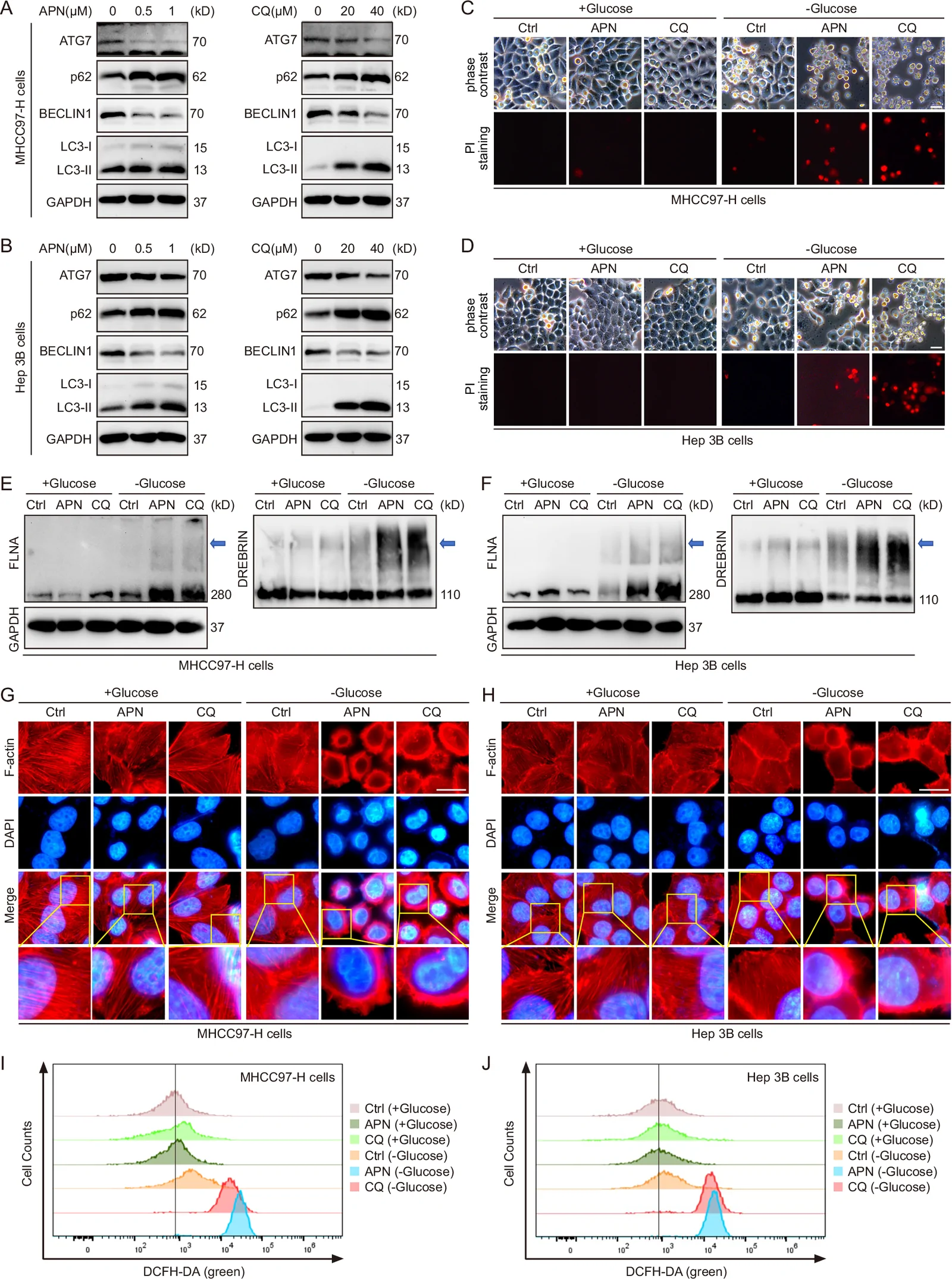
Chemical autophagy inhibitors sensitize HCC cells to glucose deprivation-induced disulfidptosis. Both MHCC97-H and Hep 3B cells were treated with Autophinib (APN) and Chloroquine (CQ) for 16 hours, and the effect of APN and CQ on autophagy markers was confirmed using western blot (**A**, **B**). The treated cells were then followed by glucose starvation (6–8 hours) to induce disulfidptosis. Then, the changes in morphology and cell death were assessed in each group (**C**, **D** Scale bar = 20 μm). Subsequently, the formation of disulfide bonds in cytoskeletal proteins (FLNA and DREBRIN) induced by glucose starvation was analyzed using non-reducing western blot (**E**, **F**) and phalloidin staining was performed to evaluate the structure of F-actin across different groups (**G**, **H** Scale bar = 10 μm). Finally, the ROS levels in different groups were evaluated via DCFH-DA staining and flow cytometry analysis (**I**, **J**).

Additionally, two different small-molecule autophagy inhibitors, Autophinib (APN) and Chloroquine (CQ), were tested in the disulfidptosis model to confirm the role of autophagy. Both MHCC97-H and Hep 3B cells were treated with APN and CQ for 16 hours. Similar to *ATG7* knockout model, we noticed that treatment with APN and CQ led to the accumulation of p62 together with decreased BECLIN1, suggesting the blockage of autophagic flux. Differently, both autophagy inhibitors led to the upregulation of LC3-II (Fig. 2A, B). Both inhibitor-treated and untreated cells were further exposed to a glucose deprivation condition (6–8 hours); the results showed that APN and CQ treatment augmented the sensitivity of HCC cells to disulfidptosis (Fig. 2C, D). Besides, the non-reducing western blot also indicated that the pharmacological suppression of autophagy resulted in slower migration of cytoskeleton proteins (Fig. 2E, F). Consistently, the phalloidin staining results showed that the contraction of F-actin and marginal clustering induced by glucose starvation was amplified through APN and CQ treatment (Fig. 2G, H). Besides, the level of ROS was measured via the DCFH-DA staining. We noticed that glucose deprivation elevated the ROS levels, while the treatment with autophagy inhibitors facilitated disulfidptosis together with increased ROS levels in HCC cells (Fig. 2I, J). Subsequently, the impact of the autophagy activator, Rapamycin (Rap), on disulfidptosis was further assessed in the present study. HCC cell lines, MHCC97-H and Hep 3B, were subjected to Rap treatment for 16 hours, after which the cells were collected to evaluate the effect of Rap on autophagy. The findings indicated that Rap treatment led to an increase in the protein levels of ATG7 and BECLIN1, while the expression levels of both p62 and LC3 were downregulated in HCC cells, implying an enhanced autophagic flux (Fig. S2A). Additionally, both control and Rap-treated cells were subjected to glucose deprivation conditions to trigger disulfidptosis. As anticipated, Rap treatment conferred protection to the cells against glucose deprivation-induced disulfidptosis, which was contrary to the effects observed with APN and CQ (Fig. S2B). The structure of F-actin was analyzed through phalloidin staining, and the results demonstrated that glucose starvation-induced F-actin contraction or marginal clustering was ameliorated by Rap treatment, with a greater organization of F-actin in the cell cortex and stress fibers in Rap-treated cells compared to untreated cells (Fig. S2C). The ROS level was also suppressed by Rap treatment in the condition of glucose starvation (Fig. S2D).

The impact of diminished autophagic flux on cell viability and cell death was also examined in our current work. The findings revealed that the deletion of *ATG7* did not influence the proliferation ability of HCC cells. The administration of CQ at a high dose inhibited cell proliferation after 48 hours, which may be attributed to drug toxicity. Conversely, the application of a low dose of CQ or treatment within 48 hours did not affect cell proliferation (Fig. S3A, B). Additionally, the level of apoptosis in HCC cells was assessed using Annexin V-FITC/PI staining, and the results further corroborated that the inhibition of autophagy did not lead to significant cell death (Fig. S3C, D). Consequently, although the inhibition of autophagic flux enhances susceptibility to disulfidptosis, the blockage of autophagy does not directly induce cell death in HCC cells.

### The downregulation of *G6PD* induced by autophagy inhibition plays a crucial role in disulfidptosis model

To conduct a more in-depth analysis of the mechanism regarding the impact of autophagy on disulfidptosis, both CQ-treated and untreated HCC cells were gathered for RNA-seq analysis (GSE327595). The genome-wide mRNA expression profiles indicated that 93 genes were upregulated while 81 genes were downregulated in the CQ-treated group in comparison to the Ctrl group (Fig. S4A, B). Following this, both the upregulated and downregulated genes underwent cluster analysis utilizing Metascape, with the top 20 enriched terms illustrated as a network plot. The network analysis revealed that Cholesterol metabolism involving the Bloch and KR pathways, Steroid biosynthesis, and cholesterol homeostasis were the most pertinent pathways and processes linked to CQ-regulated genes, alongside the positive regulation of the ROS metabolic process and the positive regulation of PCD, which also featured among the top 20 enriched terms (Fig. S4C). Moreover, the cluster analysis further suggested that the cellular response to glucose starvation and the regulation of autophagy and mitochondrion organization were all connected to the autophagy blockage induced by CQ (Fig. S4D), underscoring the critical role of autophagy in mediating disulfidptosis. The differentially expressed genes were subsequently analyzed through gene set enrichment analysis (GSEA), with results indicating a significant enrichment of genes associated with oxidative phosphorylation and the metabolism of amino acids and derivatives (Fig. S4E), thereby reinforcing the essential role of metabolic reprogramming and mitochondrial dysfunction in the process of autophagy regulation of disulfidptosis in HCC cells. The DO enrichment analysis revealed that various cancer types and liver diseases were closely associated with CQ-induced autophagy blockage. Additionally, the GO enrichment assay also suggested that various metabolic processes could be influenced by CQ-induced autophagy blockage, further confirming the significance of metabolic reprogramming (Fig. S4F, G). Our study also examined two distinct transcriptomic datasets (GSE30351 and GSE209582) sourced from the GEO database that pertain to the function of CQ. Following CQ treatment, the transcription levels of 379 genes were found to be upregulated, while 472 genes exhibited downregulation in the transcriptomic dataset GSE30351. In addition, the transcriptomic dataset GSE209582 revealed that 615 genes were upregulated and 98 genes were downregulated in CQ-treated cells compared with untreated cells (Fig. S5A, B). The differentially expressed genes were subsequently utilized for cluster analysis through Metascape, with results demonstrating that the response to oxidative stress, glucose metabolic processes, and amino acid metabolic processes were all linked to CQ treatment. Notably, CQ treatment also influenced the phosphate metabolic process, suggesting the role of autophagy in modulating the PPP, which is a vital pathway for glucose metabolism and serves as the primary source of cytosolic NADPH (Fig. S5C, D). To validate this hypothesis, we proceeded to analyze the transcription of several key genes (*G6PD*, *PGD*, *PGLS*, *RPE*, *RPIA*, *TKT* and *TALDO1*) involved in the PPP as a reference [35]. The findings indicated that the expression of *G6PD*, *PGD*, *PGLS*, *TKT* and *TALDO1* was inhibited by CQ treatment, suggesting that the inhibition of autophagy could lead to a reduction in the activity of PPP (Fig. S5E).

Furthermore, a metabolomics analysis was performed to evaluate the effects of CQ-induced autophagy inhibition on the metabolic profiles of HCC cells. Initially, the Model Overview and Permutation testing were carried out, followed by the Loading S-plot (OPLS-DA) and the Score (OPLS-DA) plot, which validated the high quality of the various samples (Fig. S6A). The results from the metabolomics analysis revealed that CQ-treated HCC cells displayed 65 downregulated metabolites and 87 upregulated metabolites when compared to untreated cells (Fig. S6B and Table S1). The heatmap showed that the suppression of autophagy significantly influenced the metabolism of alkaloids, benzenoids, and lipids (Fig. S6C), while the KEGG enrichment analysis indicated that Glycerolipid metabolism, Pentose phosphate pathway, and Amino sugar and nucleotide sugar metabolism might be affected by autophagy suppression (Fig. S6D). Moreover, the different metabolites were classified according to various pathways, revealing that carbohydrate, lipid, and amino acid metabolism were all linked to autophagy suppression, further emphasizing the essential role of autophagy in energy metabolism and GSH metabolism (Fig. S6E), which was consistent with the MSEA enrichment findings (Fig. S6F). Given the critical role of GSH-GSSH metabolism and NADP^+^-NADPH metabolism in the regulation of disulfidptosis, along with the relationship of mitochondrial hemopoiesis to the aforementioned metabolism, this multi-omics data underscores the significant role of metabolic reprogramming and mitochondrial dysfunction in the process of autophagy regulating disulfidptosis in HCC cells, which may be regarded as the primary underlying mechanism. Particularly, the function of PPP may play a crucial role in the process of autophagy that regulates disulfidptosis.

**Fig. 3:**
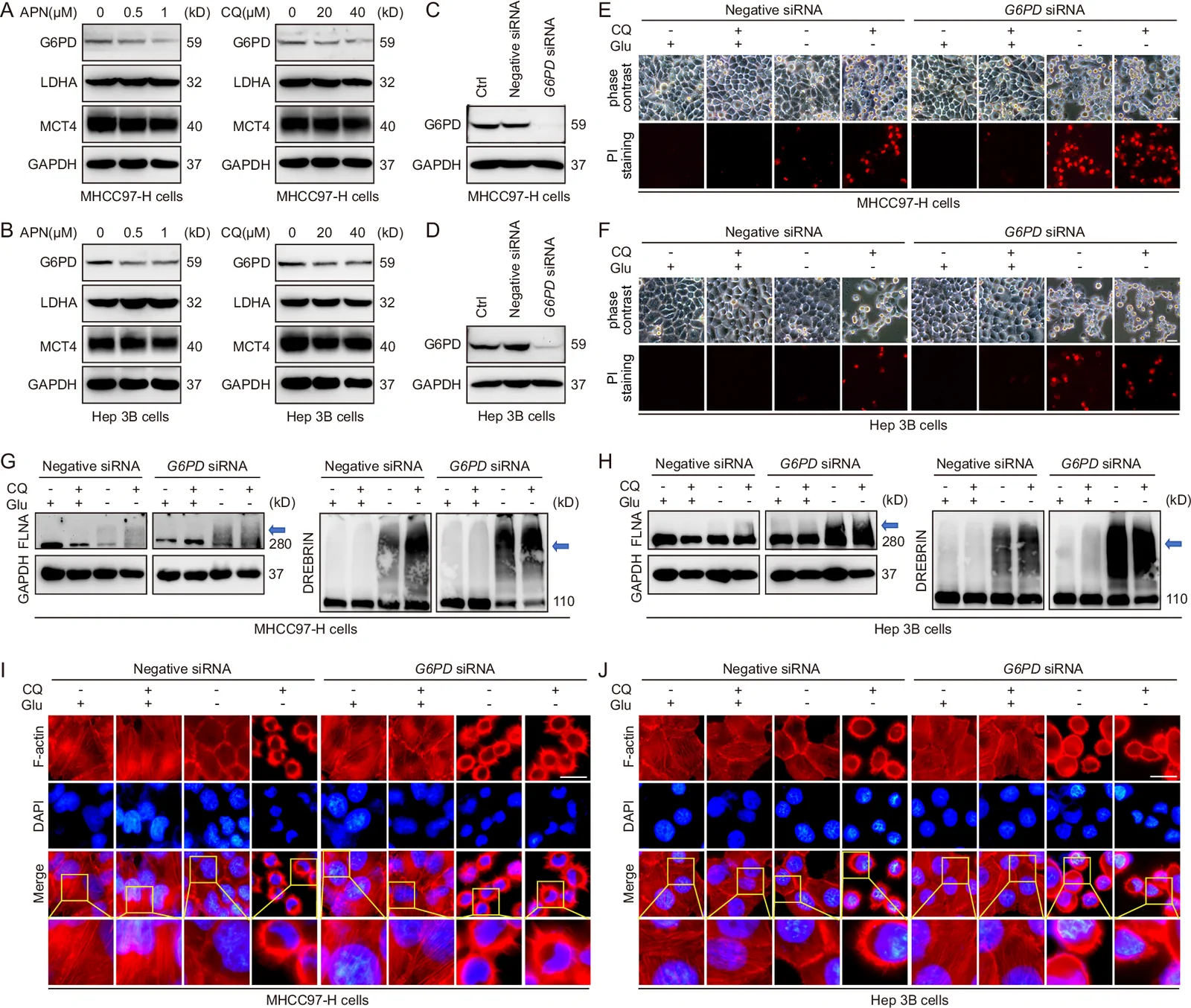
The downregulation of G6PD caused by the inhibition of autophagy plays a significant role in the disulfidptosis model. Herein, both MHCC97-H and Hep 3B cells were treated with APN and Chloroquine CQ for 16 hours, the protein levels of G6PD and other key genes in glucose metabolism were measured using Western blot (**A**, **B**). The knockdown of G6PD was induced via specific siRNA transfection, and the cells transfected with Negative siRNA were used as Ctrl group. The knockdown effectiveness was verified through western blot analysis (**C**, **D**). Both Ctrl group and the knockdown group were treated with CQ (40 μM) for 16 hours, followed by glucose starvation (6–8 hours) to induce disulfidptosis. Then, the changes in morphology and cell death were assessed in each group (**E**, **F** Scale bar = 20 μm). Meanwhile, the formation of disulfide bonds in cytoskeletal proteins (FLNA and DREBRIN) induced by glucose starvation was analyzed using non-reducing western blot (**G**, **H**), and phalloidin staining was performed to evaluate the structure of F-actin across different groups (**I**, **J** Scale bar = 10 μm).

G6PD is regarded as the rate-limiting enzyme that catalyzes the initial step of the PPP. Subsequently, we evaluated the protein levels of G6PD in HCC cells subjected to treatment with APN and CQ. Our findings indicated that treatment with APN and CQ resulted in a significant downregulation of G6PD. Nevertheless, other regulators related to glucose metabolism, such as LDHA and MCT4, were not affected by the autophagy inhibitors (Fig. 3A, B). In addition, a *G6PD* knockdown model was established using the siRNA transfection technique in MHCC97-H and Hep 3B cells (Fig. 3C, D). Following this, we assessed the impact of autophagy on disulfidptosis triggered by glucose starvation in both wild-type and *G6PD* knockdown HCC cells. Our results revealed that *G6PD* knockdown enhanced susceptibility to disulfidptosis, and both APN (Fig. S7A, B) and CQ (Fig. 3E, F) heightened the sensitivity of HCC cells to disulfidptosis, a process that could be mitigated by *G6PD* knockdown. Furthermore, non-reducing western blot analysis demonstrated that CQ treatment facilitated the slower migration of cytoskeletal proteins, while *G6PD* knockdown significantly reduced the effects of CQ (Fig. 3G, H). The distribution of F-actin across various groups was further analyzed through phalloidin staining, with results showing that CQ treatment intensified glucose starvation-induced F-actin contraction and marginal clustering in wild-type HCC cells, but not in *G6PD* knockdown HCC cells (Fig. 3I, J). To further validate the role of G6PD in the regulation of autophagy and disulfidptosis, we employed both *G6PD* overexpression and a G6PD activator in rescue experiments to investigate the proposed pathways. In this study, we facilitated the overexpression of *G6PD* in HCC cells via pCMV-G6PD transfection, while the activity of G6PD was enhanced through treatment with the G6PD activator AG1, which did not alter the protein levels. Our findings indicated that CQ treatment increased the sensitivity of HCC cells to disulfidptosis and resulted in a slower migration of cytoskeletal proteins, whereas *G6PD* overexpression and activation significantly mitigated the effects of CQ (Fig. S8A–D). Furthermore, CQ treatment exacerbated glucose starvation-induced F-actin contraction and marginal clustering in wild-type HCC cells, but this was not observed in *G6PD* overexpressing HCC cells, and the CQ effect was also restricted by the application of the G6PD activator AG1 (Fig. S8E, F). These findings suggest that G6PD, the rate-limiting enzyme in the PPP, plays a crucial role in the regulatory function of autophagy in disulfidptosis.

### Autophagic flux inhibition disturbs GSH synthesis and exhausts NADPH in disulfidptosis model

**Fig. 4:**
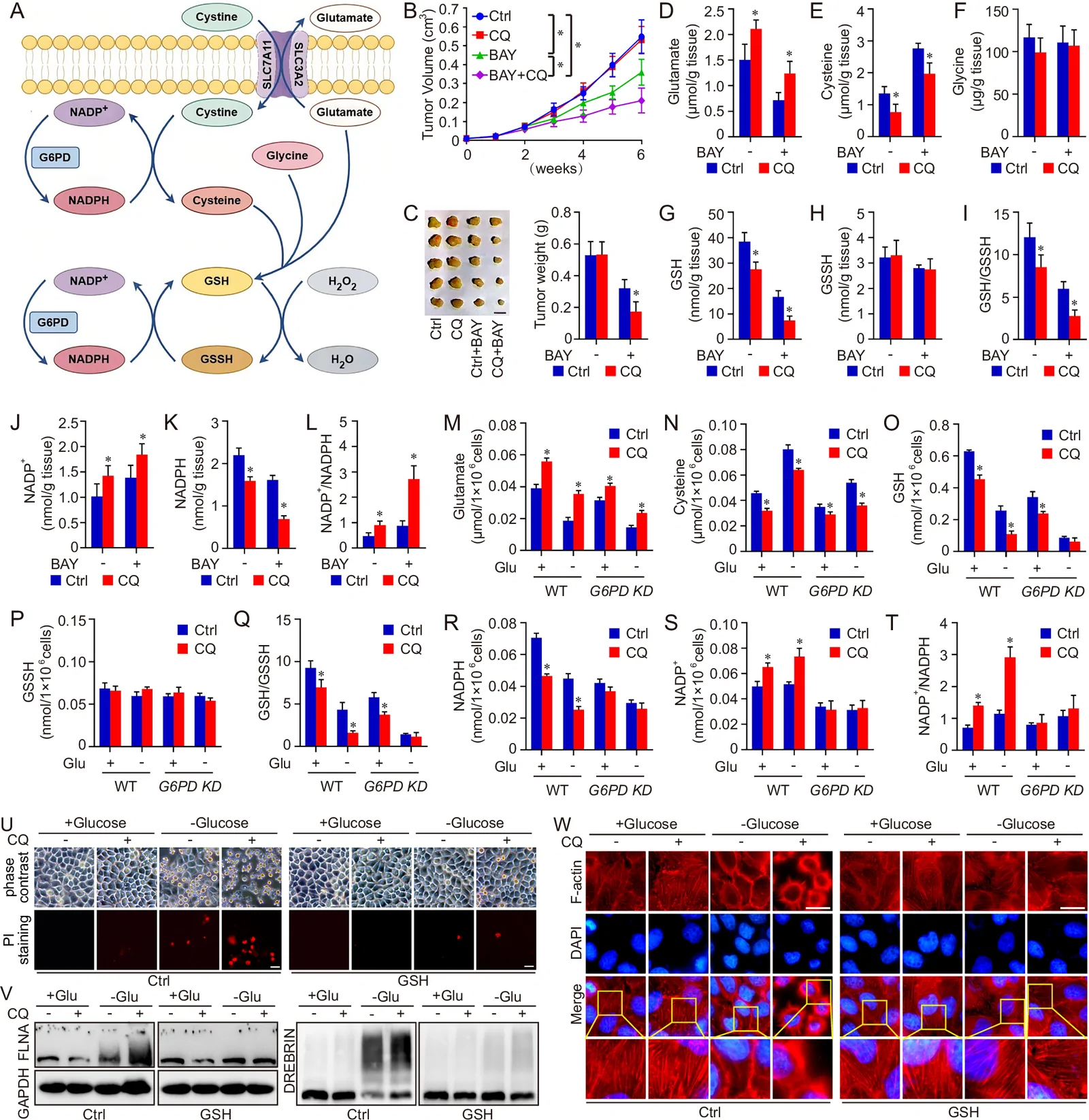
Inhibition of autophagic flux disrupts GSH synthesis and depletes NADPH within the disulfidptosis model. Firstly, an overview of NADPH metabolism and GSH synthesis was presented in (**A**). Wild-type MHCC97-H cells were used to establish xenograft model, which was subsequently treated with CQ and BAY-876 (BAY). Our study measured both tumor volume (**B**) and tumor weight (**C**). Furthermore, the levels of glutamate (**D**), cysteine (**E**), and glycine (**F**) were assessed in tumor tissues from each group. Additionally, GSH metabolism (**G**–**I**) and NADPH metabolism (**J**–**L**) in tumor tissues from different groups were evaluated in our research. Conversely, the effect of CQ on GSH synthesis and NADPH metabolism was analyzed using wild type and *G6PD* knockdown HCC cells in vitro. Both wild-type and *G6PD* knockdown MHCC97-H cells were initially treated with CQ (40 μM) for 16 hours, followed by glucose starvation (6–8 hours) to induce disulfidptosis. The levels of glutamate (**M**) and cysteine (**N**) were measured in each group, and GSH metabolism (**O**–**Q**) and NADPH metabolism (**R**–**T**) in various groups were evaluated respectively. The position of GSH was confirmed in current work, and the cells were co-treated with CQ (40 μM) and GSH (1 mM), followed by glucose starvation. The morphological changes and cell death were assessed in each group (**U** Scale bar = 20 μm). Non-reducing western blot was used to determine the formation of disulfide bonds in cytoskeletal proteins (**V**), and phalloidin staining was utilized to evaluate the actin filament (F-actin) in different groups (**W** Scale bar = 10 μm). (Data are presented as means ± SD. *p < 0.05 compared to Ctrl groups).

As a crucial element in PPP, G6PD primarily regulates the generation of NADPH, which is also associated with the processes of GSH synthesis (Fig. 4A). Consequently, the impact of autophagy blockage on GSH synthesis and NADPH metabolism was assessed both in vitro and in vivo. In this context, BAY-876 was utilized to induce HCC disulfidptosis in vivo as references [1, 25]. Notably, the combination of autophagy inhibitor (CQ) and BAY-876 exhibited enhanced therapeutic outcomes compared to either agent alone. Both tumor volume and tumor weight were diminished in the combination group relative to the individual CQ or BAY-876 groups (Fig. 4B, C). As essential elements in the GSH synthesis and metabolism process, the levels of glutamate, cysteine, and glycine were measured in tumor tissues from different groups. Our findings showed that CQ treatment led to a decrease in cysteine levels while increasing glutamate levels in tumor tissues with or without BAY-876 exposure (Fig. 4D, E). The glycine level did not show a significant variation among different groups (Fig. 4F). Subsequently, both GSH-GSSH metabolism and NADP^+^-NADPH metabolism were investigated in the current work. The findings indicated that CQ-induced autophagy suppression influenced GSH-GSSH metabolism and decreased the ratio of GSH/GSSH (Fig. 4G, I). Following this, we examined NADP^+^-NADPH metabolism in tumor tissues, and the results indicated that treatment with CQ decreased NADPH level while increasing NADP^+^ level as well as NADP^+^/NADPH ratio. The function of CQ was further amplified under BAY-876 exposure compared to normal conditions (Fig. 4J–L).

To further validate the significance of G6PD in the function of autophagy on disulfidptosis regulation, we conducted an in vitro analysis of the impact of CQ-induced autophagy inhibition on GSH synthesis and NADPH metabolism using both wild-type and *G6PD* knockdown HCC cells. The findings revealed that *G6PD* knockdown mitigated the effect of CQ on cysteine and glutamate levels, although CQ treatment still resulted in a reduction of cysteine levels and an elevation of glutamate levels in both wild-type and *G6PD* knockdown cells (Fig. 4M, N). Additionally, when compared to wild-type cells, *G6PD* knockdown cells exhibited significantly lower levels of GSH and NADPH, and the impact of CQ-induced autophagy inhibition on GSH and NADPH metabolism was restricted in *G6PD* knockdown cells. CQ treatment led to a decrease in GSH levels and the GSH/GSSH ratio in wild-type cells, while the effect of CQ was notably inhibited in *G6PD* knockdown cells, particularly under glucose starvation conditions (Fig. 4O–Q). Concurrently, CQ treatment lowered the NADPH level and increased the NADP^+^/NADPH ratio in wild-type cells, which was completely obstructed in *G6PD* knockdown cells (Fig. 4R–T). Therefore, CQ-induced autophagy inhibition disrupted GSH and NADPH metabolism in a G6PD-dependent manner. Our previous research has highlighted the critical role of GSH in the regulation of disulfidptosis [25], and the effect of autophagy on disulfidptosis was assessed in both GSH-treated and GSH-untreated HCC cells. We found that both CQ treatment and *ATG7* knockout-induced autophagy inhibition heightened the sensitivity of HCC cells to disulfidptosis, and this effect could be entirely reversed by the addition of GSH (Figs. 4U and S9A). Moreover, a non-reducing Western blot assay demonstrated that the inhibition of autophagy led to slower migration of cytoskeletal proteins, which could be ameliorated by GSH administration (Fig. 4V and S9B). Consistently, phalloidin staining showed that GSH co-treatment mitigated the effects of autophagy, as well as reduced glucose starvation-induced F-actin contraction and marginal clustering in HCC cells (Figs. 4W and S9C). Overall, these findings validate that the inhibition of autophagic flux enhances the sensitivity of HCC cells to disulfidptosis by influencing the PPP. The downregulation of G6PD disturbs GSH synthesis and exhausts NADPH, which can be the key underlying metabolic mechanisms in the disulfidptosis model.

### The obstruction of autophagic flux results in mitochondrial dysfunction and an increasing the susceptibility of HCC to disulfidptosis

**Fig. 5:**
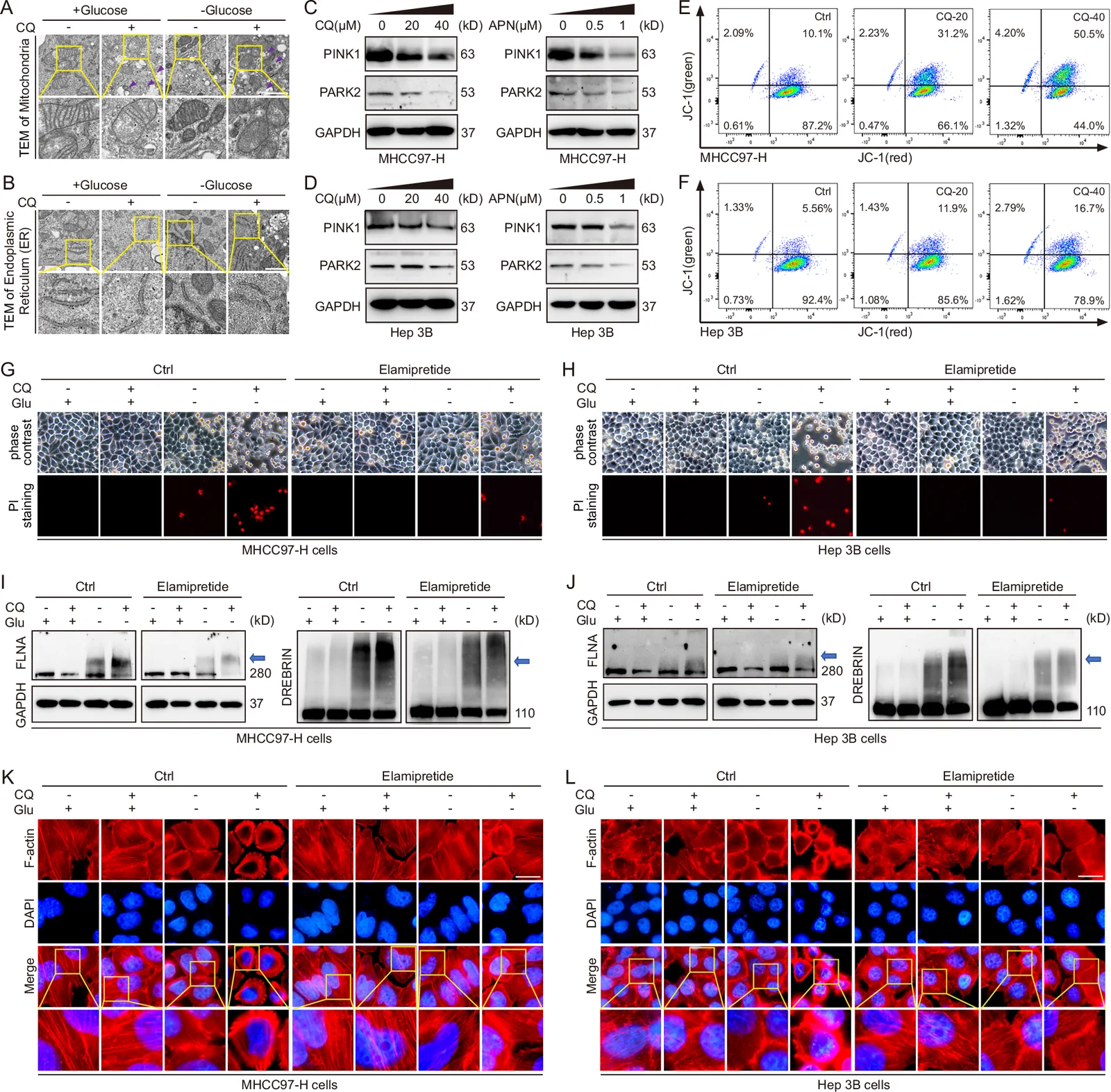
The blockage of autophagic flux leads to mitochondrial dysfunction, thereby heightening the susceptibility of HCC to disulfidptosis. MHCC97-H cells were treated with CQ (40 μM) for 16 hours, followed by glucose starvation (6–8 hours). Then, the cells from different groups were harvested for transmission electron microscopy (TEM) analysis (**A**, **B** Scale bar = 1 μm, arrowhead: autophagosome). In addition, the effect of APN and CQ on mitophagy markers (PINK1 and PARK2) was evaluated by western blot assay (**C**, **D**). The mitochondrial membrane potential (MMP) was also analyzed via JC-1 staining (**E**, **F**). Herein, elamipretide (10 μM), a peptide compound that enhances mitochondrial function, was applied in the co-treatment, and the morphological changes and cell death were assessed in each group (**G**, **H** scale bar = 20 μm). A non-reducing Western blot was used to determine the formation of disulfide bonds in cytoskeletal proteins (**I**, **J**), and phalloidin staining was utilized to evaluate the actin filament (F-actin) in different groups (**K**, **L** Scale bar = 10 μm).

To further elucidate the characteristics of cell death enhanced by autophagy inhibition during glucose deprivation, MHCC97-H cells, both treated and untreated with CQ, were subjected to glucose starvation. Subsequently, the cells were collected for TEM analysis. The findings indicated that CQ treatment resulted in the accumulation of autophagosomes. Notably, the electron density of mitochondria was significantly increased under glucose deprivation, and CQ treatment facilitated the dissolution of mitochondrial cristae (Fig. 5A), which was also consistent with our previous RNA-seq prediction (Fig. S4D, E). Some studies have established a close relationship between ER stress and disulfidptosis [5]. The structure of the ER was also examined in our research. However, no significant differences were observed in the ER structure between the CQ-treated and untreated groups (Fig. 5B). Therefore, it is plausible that mitochondria play a crucial role in the process of disulfidptosis. As functional autophagy inhibitors, the effects of CQ and APN on mitophagy were assessed in HCC cells. Our results revealed that treatment with CQ and APN reduced the protein levels of PINK1 and PARK2, which are two essential markers of mitophagy, suggesting that pharmacological inhibition of autophagy also impedes mitophagy in HCC cells (Fig. 5C, D). Furthermore, the MMP across various groups was further analyzed using JC-1 staining. The results demonstrated that CQ treatment increased the green signal in both MHCC97-H and Hep 3B cells, indicating that the autophagy blockage induced by CQ elevated the levels of JC-1 monomer and reduced MMP in HCC cells (Fig. 5E, F). Consequently, the disruption of autophagic flux leads to mitochondrial dysfunction in HCC cells, which could be associated with the impact of autophagy on disulfidptosis.

To validate the aforementioned hypothesis, elamipretide, a peptide compound that specifically targets the inner mitochondrial membrane and enhances mitochondrial function, was utilized in our research. HCC cell lines, MHCC97-H and Hep 3B cells, were subjected to treatment with either single CQ or CQ in combination with elamipretide for a duration of 16 hours, followed by glucose deprivation (6–8 hours) to induce disulfidptosis. The cell death in each group was assessed initially. Our findings indicated that CQ amplified cell death triggered by glucose deprivation, while the co-treatment with Elamipretide inhibited the function of CQ in the disulfidptosis model (Fig. 5G, H). Furthermore, the migration of cytoskeletal proteins (FLNA and DREBRIN) was analyzed using non-reducing western blot techniques, revealing that CQ-induced suppression of autophagy led to a slower migration of cytoskeletal proteins, which could be improved by the co-treatment with Elamipretide (Fig. 5I, J). Subsequently, the structure of F-actin was examined through phalloidin staining, and it was observed that the co-treatment with Elamipretide mitigated the effects of autophagy inhibition on disulfidptosis, improving glucose starvation-induced F-actin contraction and reducing clustering in HCC cells (Fig. 5K, L). This suggests that mitochondrial dysfunction resulting from the inhibition of autophagic flux is also linked to the influence of autophagy on disulfidptosis.

### The influence of ER stress on disulfidptosis depends on the regulation of autophagy

**Fig. 6:**
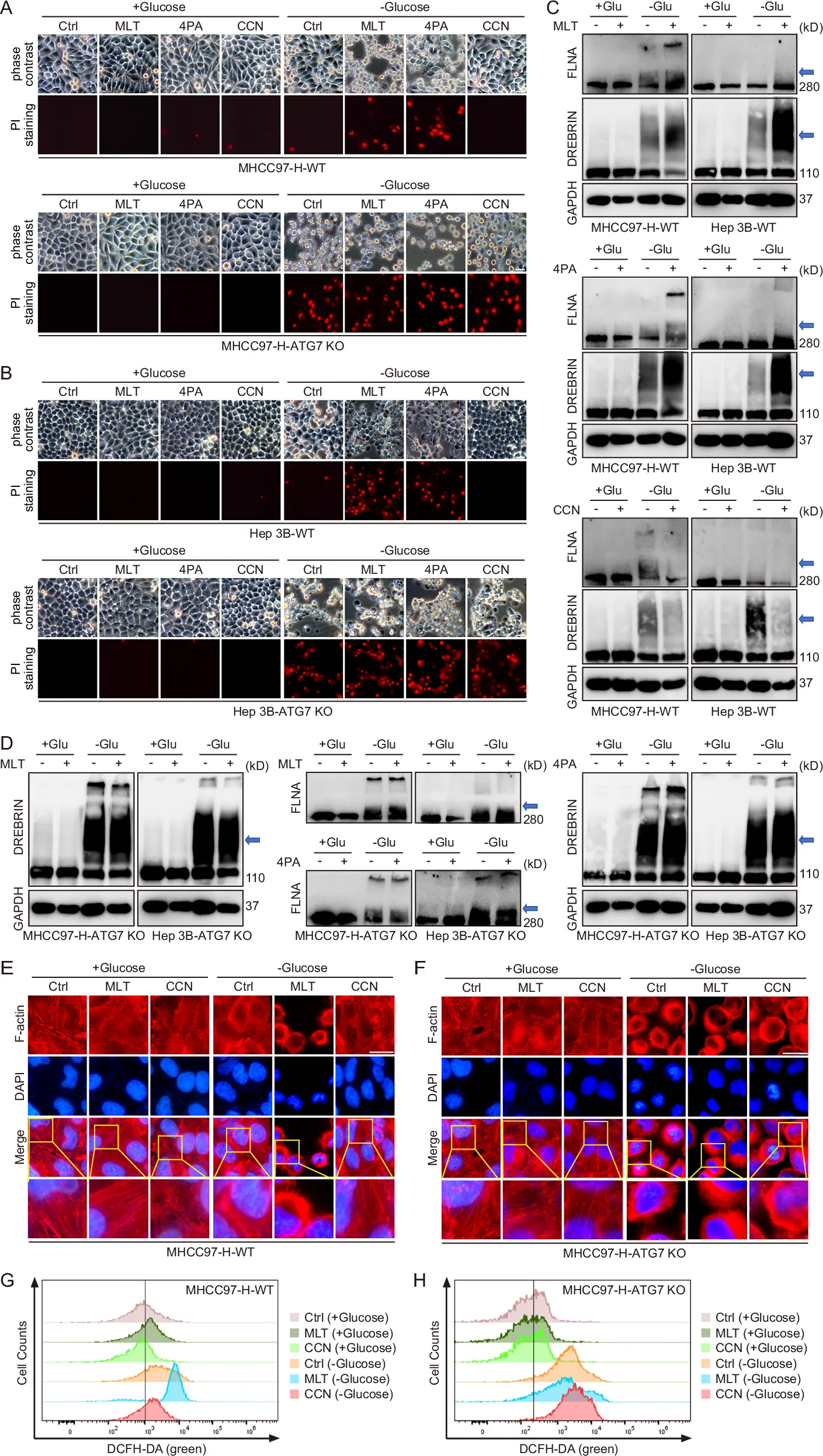
The effect of ER stress on disulfidptosis is dependent upon the regulation of autophagy. To confirm the crucial role of autophagy in the effects of ER stress on disulfidptosis regulation, both wild-type (WT) and *ATG7* knockout (KO) HCC cell lines (MHCC97-H and Hep 3B) were used in the current work. The cells were treated with ER stress inhibitors, Melatonin (MLT, 0.2 mM) and 4-phenylbutyric acid (4PA, 2 mM), and ER stress activator Cinchonine (CCN, 20 μM), as then followed by glucose starvation (6–8 hours) to induce disulfidptosis. Then, the changes in morphology and cell death were assessed in each group (**A**, **B** Scale bar = 20 μm). Subsequently, the formation of disulfide bonds in cytoskeletal proteins (FLNA and DREBRIN) induced by glucose starvation was analyzed using non-reducing western blot (**C**, **D**), and phalloidin staining was performed to evaluate the structure of F-actin across different groups (**E**, **F** Scale bar = 10 μm). Finally, the ROS levels in different groups were evaluated via DCFH-DA staining and flow cytometry analysis (**G**, **H**).

Some researchers have highlighted the significant role of ER stress in the regulation of disulfidptosis. In this context, ER stress inhibitors have been shown to enhance disulfidptosis during periods of glucose starvation, while certain ER stress inducers have the opposite effect, suppressing disulfidptosis [5]. Given the interaction between ER stress and autophagy, it is plausible that autophagy plays a crucial role in the effects of ER stress on disulfidptosis regulation. In our current study, we utilized two small-molecule ER stress inhibitors, melatonin (MLT) and 4-phenylbutyric acid (4PA), along with one small-molecule ER stress activator, cinchonine (CCN). We assessed the effects of various ER stress regulators in both wild-type and *ATG7* knockout cells to validate the role of autophagy in the influence of ER stress on disulfidptosis regulation. Our findings revealed that treatment with MLT and 4PA facilitated cell death induced by glucose deprivation, whereas CCN treatment conferred protection to HCC cells against disulfidptosis in wild-type cells. Nonetheless, the effects of all ER stress regulators were markedly diminished in *ATG7* knockout cells (Fig. 6A, B). Furthermore, we conducted a non-reducing western blot analysis to examine cytoskeletal proteins in HCC cells, which indicated that treatment with MLT and 4PA resulted in a slower migration of cytoskeletal proteins. In contrast, CCN treatment was found to enhance the migration of these cytoskeletal proteins. However, the effects of both MLT and 4PA were inhibited in *ATG7* knockout cells (Fig. 6C, D). Additionally, we assessed the structure of F-actin through phalloidin staining, discovering that the application of the ER stress inhibitor (MLT) promoted disulfidptosis induced by glucose deprivation, while the cell death could be mitigated by the ER stress activator (CCN) in wild-type HCC cells, but not in *ATG7* knockout HCC cells (Fig. 6E, F). Moreover, the level of ROS was measured in different groups. The results showed that glucose deprivation elevated the ROS levels, which could be enhanced by MLT treatment and decreased by CCN treatment in wild-type HCC cells, and the function of ER stress regulators was blocked in *ATG7* knockout HCC cells (Fig. 6G, H). Consequently, these results imply that the impact of ER stress on disulfidptosis is contingent upon the regulation of autophagy in HCC cells.

To determine whether the biological processes, ER stress-impaired mitophagy-metabolic disruption, operate in parallel or sequentially prior to disulfidptosis, we employed two distinct ER stress inhibitors, MLT and 4PA, in the treatment of HCC to examine ER stress, mitophagy, and metabolic disruption at various time points (4 h, 8 h, and 16 h). Our findings revealed that the protein levels of ATF4 and p-eIF2α exhibited a significant decrease in HCC cells following a 4-hour treatment with MLT and 4PA (Fig. S10A, B), alongside the upregulation of p62 and LC3, as well as the downregulation of BECLIN1 (Fig. S10C, D). Furthermore, the mitophagy markers, PINK and PARK2, were downregulated after an 8-hour treatment in HCC cells (Fig. S10E, F). We also assessed the alterations in NADP^+^-NADPH metabolism and GSH-GSSH metabolism at different time points, and the results indicated that both MLT and 4PA treatments could disrupt these metabolic processes, with the GSH/GSSH ratio decreasing in treated cells after 16 hours, while the NADP^+^/NADPH ratio increased following treatment with MLT or 4PA (Fig. S10G–J). Overall, these results suggest that treatment with ER stress inhibitors initially disrupted the balance between ER stress and autophagy, subsequently leading to impaired mitophagy. Consequently, NADP^+^-NADPH metabolism and GSH-GSSH metabolism were affected in HCC cells. Therefore, the axis of “homeostasis disbalance of ER stress and autophagy-impaired mitophagy-metabolic disruption” may be regarded as the sequential upstream of disulfidptosis.

Our earlier findings have demonstrated that treatment with ER stress inhibitors (MLT and 4PA) disrupted the balance between ER stress and autophagy (Fig. S10). To thoroughly examine various cell death pathways, we initially harvested both MLT-treated and untreated HCC cells for RNA-seq analysis (GSE327595). The genome-wide mRNA expression profiles indicated that 910 genes were upregulated while 1163 genes were downregulated in the MLT-treated group in comparison to the Ctrl group (Fig. S11A, B). Following this, both the upregulated and downregulated genes underwent cluster analysis utilizing Metascape, with the top 20 enriched terms illustrated as a network plot. The network analysis revealed that gland development and tube morphogenesis were the most pertinent pathways and processes linked to MLT-regulated genes, with positive regulation of PCD also appearing among the top 20 enriched terms (Fig. S11C). Moreover, the cluster analysis further suggested that MLT treatment influenced the regulation of autophagy and the ER stress-related UPR. Additionally, Endocytosis, Apoptosis, Efferocytosis, and Ferroptosis were noted in the enrichment list. Although disulfidptosis was not included in the database, Glycolysis and gluconeogenesis, actin cytoskeleton, and cellular response to glucose starvation were all closely related to MLT treatment (Fig. S11D). GSEA analysis also revealed a significant enrichment of genes associated with Glycolysis/Gluconeogenesis and oxidative phosphorylation (Fig. S11E), underscoring the critical role of metabolic reprogramming and mitochondrial dysfunction. The genes associated with autophagy and endocytosis (Fig. S11F, G), PCD (Fig. S11H–J), and the disulfidptosis-related pathway (Fig. S11K–M) were arranged in distinct heatmaps. Consequently, in addition to disulfidptosis, the balance between ER stress and autophagy may also be linked to other forms of cell death, including apoptosis and ferroptosis. We subsequently assessed the impact of ER stress inhibitors on Cisplatin-induced apoptosis and Erastin/Sorafenib-induced ferroptosis. In contrast to disulfidptosis, the co-administration of ER stress inhibitors conferred protection to HCC cells against apoptosis and ferroptosis (Fig. S11N–P). We also examined the effect of ER stress inhibitors on Elesclomol-induced Cuproptosis, despite Cuproptosis not being listed in the enrichment analysis. The findings revealed that only MLT provided minimal protection against Cuproptosis, while 4PA had no effect on Elesclomol-induced Cuproptosis in HCC cells (Fig. S11Q). Therefore, although the homeostasis between ER stress and autophagy is also associated with apoptosis and ferroptosis, the inhibition of ER stress offered protection to cells from cell death, which is contrary to the role of ER stress in disulfidptosis.

Furthermore, a metabolomics analysis was performed to evaluate the effects of MLT-induced inhibition of ER stress on the metabolic profiles of HCC cells. Initially, the Model Overview and Permutation testing were carried out, followed by the Loading S-plot (OPLS-DA) and the Score (OPLS-DA) plot, which validated the high quality of the various samples (Fig. S12A). The results from the metabolomics analysis revealed that MLT-treated HCC cells displayed 133 downregulated metabolites and 159 upregulated metabolites when compared to untreated cells (Fig. S12B and Table S2). The heatmap demonstrated that the suppression of autophagy had a significant impact on the metabolism of benzenoids and lipids (Fig. S12C), while the KEGG enrichment assay indicated that the Pentose phosphate pathway, along with Amino sugar and nucleotide sugar metabolism, could be affected by the suppression of ER stress (Fig. S12D). Moreover, the different metabolites were classified according to various pathways, revealing that carbohydrate, lipid, and amino acid metabolism were all linked to the suppression of ER stress, further emphasizing the essential role of ER stress-autophagy in energy metabolism and GSH metabolism (Fig. S12E), which was consistent with the MSEA enrichment results (Fig. S12F). Consequently, the metabolomics data provided additional support for the notion that the homeostasis between ER stress and autophagy is closely related to disulfidptosis.

### The clinical agent clofazimine acts as a potential modulator that targets the homeostasis between ER stress and autophagy

**Fig. 7:**
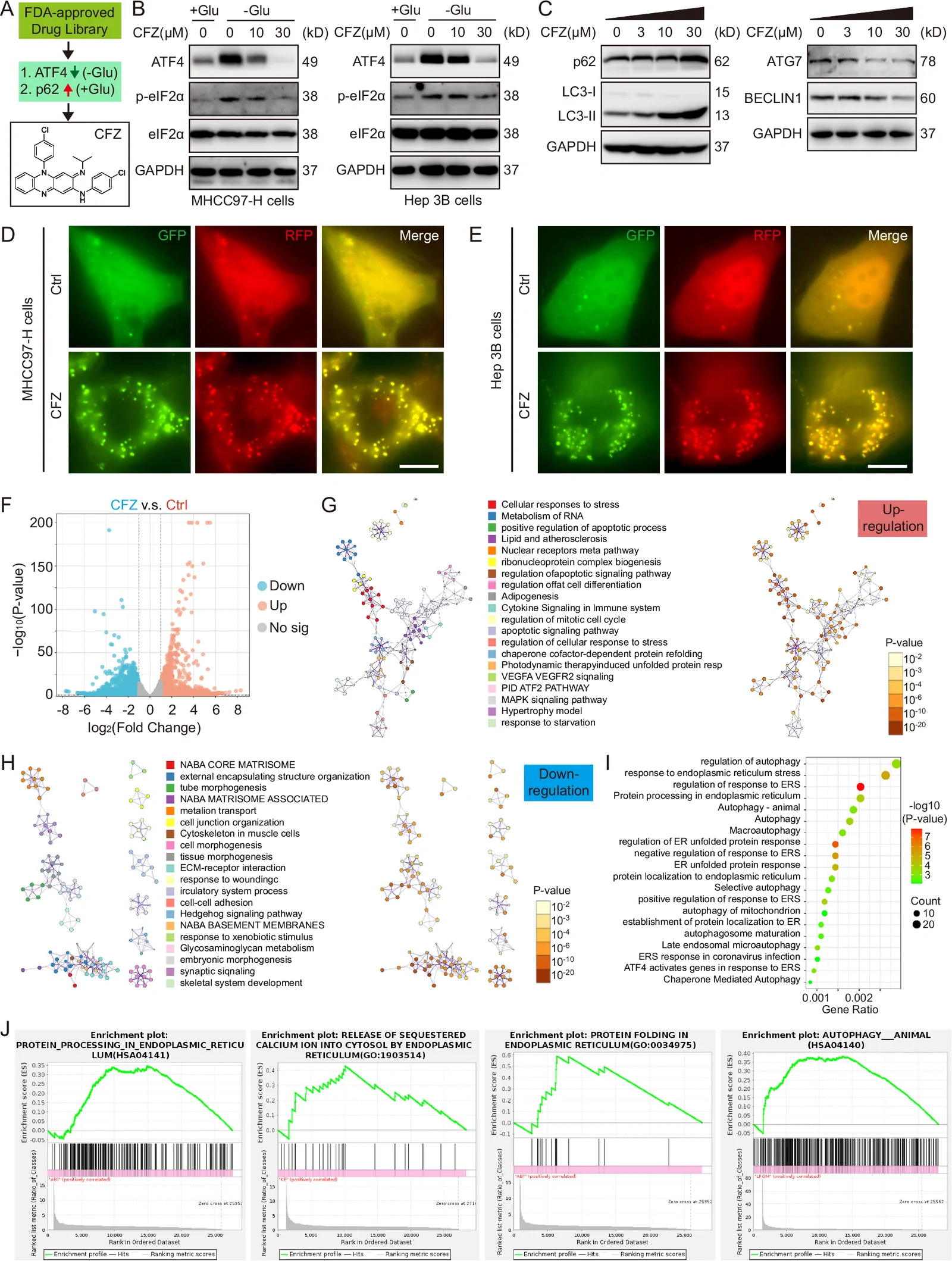
The clinical agent clofazimine serves as a potential modulator that influences the balance between ER stress and autophagy. The strategy for the drug screen work was shown in (**A**). HCC cells treated with Clofazimine (CFZ) in different concentrations were collected for western blot assay. Herein, ER stress markers (ATF4 and p-eIF2α) as well as autophagy markers (p62, LC3, ATG7, and BECLIN1) were examined, respectively (**B**, **C**). Furthermore, the effect of CFZ on autophagic flux was investigated using the tandem mRFP-GFP-LC3 reporter construct (**D**, **E**). To further assess the pharmacological role of CFZ, MHCC97-H cells treated with CFZ (30 μM) were collected for RNA-seq analysis. A volcano plot was created to assess the impact of CFZ treatment on gene transcription (**F**). Additionally, both upregulated and downregulated genes were utilized for cluster analysis through Metascape. The network of enriched terms for both upregulated and downregulated genes was color-coded by cluster ID, and the same terms were further distinguished by *p* value (**G**, **H**). Moreover, several enriched terms associated with autophagy and ER stress were presented in (**I**). Lastly, the differentially expressed genes were further investigated through GSEA (**J**).

At present, the compounds that target the balance between ER stress and autophagy remain scarce, despite the established complex relationship between these two processes as confirmed by researchers [14, 15, 36]. In this study, the FDA-approved Drug Library was utilized for drug screening, where the identified compounds were expected to reduce the expression of ATF4 during glucose starvation while increasing the protein level of p62 under normal conditions (Fig. 7A). Ultimately, the clinical agent CFZ was recognized as a functional modulator that targets the homeostasis between ER stress and autophagy in HCC cells. Our findings demonstrated that glucose starvation intensified ER stress, which was associated with the upregulation of ATF4 and p-eIF2α, effects that could be mitigated by CFZ treatment (Fig. 7B). Additionally, the impact of CFZ on autophagy was assessed in HCC cells. The results revealed that CFZ treatment elevated the protein levels of p62 and LC3, while the expression of two other autophagy markers, ATG7 and BECLIN1, was downregulated by CFZ treatment, indicating CFZ’s inhibitory effect on autophagy (Fig. 7C). Furthermore, the influence of CFZ on autophagic flux was further examined using the tandem mRFP-GFP-LC3 reporter construct. HCC cell lines, MHCC97-H and Hep 3B, were transfected with the mRFP-GFP-LC3 construct and subsequently treated with CFZ for 16 hours. Our results indicated that the number of yellow puncta (autophagosomes) was increased in the CFZ-treated HCC cells compared to the untreated group, suggesting that CFZ treatment led to the obstruction of autophagic flux along with the accumulation of autophagosomes (Fig. 7D, E).

To further explore the possible role of CFZ in HCC cells, both CFZ-treated and untreated MHCC97-H cells were collected for RNA-seq analysis (GSE327600). The genome-wide mRNA expression profiles revealed that 1769 genes were upregulated and 2312 genes were downregulated in the CFZ-treated group compared to the Ctrl group (Fig. 7F). Subsequently, both the upregulated and downregulated genes were subjected to cluster analysis using Metascape, and the top 20 enriched terms were presented as a network plot. The network analysis showed that Cellular responses to stress, Metabolism of RNA, and positive regulation of the apoptotic process were the most relevant pathways and processes associated with CFZ-upregulated genes, while the NABA CORE MATRISOME, external encapsulating structure organization, and tube morphogenesis were the most relevant pathways and processes linked to CFZ-downregulated genes (Fig. 7G, H). Furthermore, the cluster analysis also indicated that the regulation of autophagy, response to ER stress, and ER UPR were all associated with CFZ treatment (Fig. 7I), highlighting the significant role of CFZ in mediating the interaction between ER stress and autophagy. The differentially expressed genes were then subjected to GSEA, and the findings indicated a significant enrichment of genes related to ER function and autophagy (Fig. 7J), supporting the regulatory role of CFZ in maintaining the balance between ER stress and autophagy.

Subsequently, a network pharmacology analysis was conducted to investigate the potential targets and functions of CFZ based on its chemical structure (Fig. S13A). Our study utilized both the SwissTargetPrediction database and the TargetNet database, which predicted 100 and 273 potential bioavailable targets, respectively. Furthermore, 40 overlapping targets were identified in the predicted outcomes (Fig. S13B, C and Table S3), which were selected for further cluster analysis using Metascape. Initially, a PPI network was established (Fig. S13D), and the 20 enriched terms were compiled into a network plot. The results of the network analysis indicated that the cellular response to nitrogen compounds, regulatory circuits of STAT3 signaling, and macrophage-stimulating protein MSP signaling were most closely associated with the bioavailable targets of CFZ (Fig. S13E, F). Additionally, the regulation of autophagy and protein processing in the ER were also linked to CFZ targets, aligning with the RNA-seq findings (Fig. S13G). Moreover, genes related to hepatocarcinogenesis were examined in the DisGeNET database, revealing 12 genes that overlapped with the bioavailable targets of CFZ (Fig. S13H, I), highlighting the significant role of CFZ in the treatment of HCC.

### Clofazimine enhances the sensitivity of HCC cells to disulfidptosis via suppressing autophagy

Due to the role of CFZ in ER stress and autophagy, our study further assessed the impact of CFZ on the sensitivity of HCC cells to disulfidptosis. Both MHCC97-H and Hep 3B cells were subjected to CFZ treatment for 16 hours, followed by exposure to glucose starvation for 6–8 hours. Our findings revealed that CFZ treatment significantly heightened the sensitivity of HCC cells to disulfidptosis induced by glucose starvation (Fig. S14A). Additionally, a non-reducing western blot assay demonstrated that CFZ treatment resulted in slower migration of cytoskeletal proteins in HCC cells, while phalloidin staining indicated that CFZ treatment enhanced F-actin contraction and marginal clustering in both MHCC97-H and Hep 3B cells (Fig. S14B, C). We also analyzed the effect of CFZ on mitochondria in our research. Consistent with other autophagy inhibitors, the TEM assay indicated that CFZ treatment led to the accumulation of autophagosomes. Furthermore, glucose deprivation increased mitochondrial electron density, and CFZ treatment facilitated the dissolution of mitochondrial cristae. Importantly, the structure of the ER remained unaffected by CFZ treatment (Fig. S15A). The MMP in each group was further evaluated using JC-1 staining, with results showing that CFZ treatment enhanced the green signal in HCC cells, indicating a decrease in MMP in CFZ-treated cells compared to untreated cells (Fig. S15B, C). Additionally, we assessed the effect of CFZ on mitophagy by measuring PINK1 and PARK2 levels. The western blot analysis revealed that CFZ treatment reduced the protein levels of both PINK1 and PARK2, along with a decrease in *G6PD* expression, aligning with the effects observed with other autophagy inhibitors, APN and CQ (Fig. S15D, E).

**Fig. 8:**
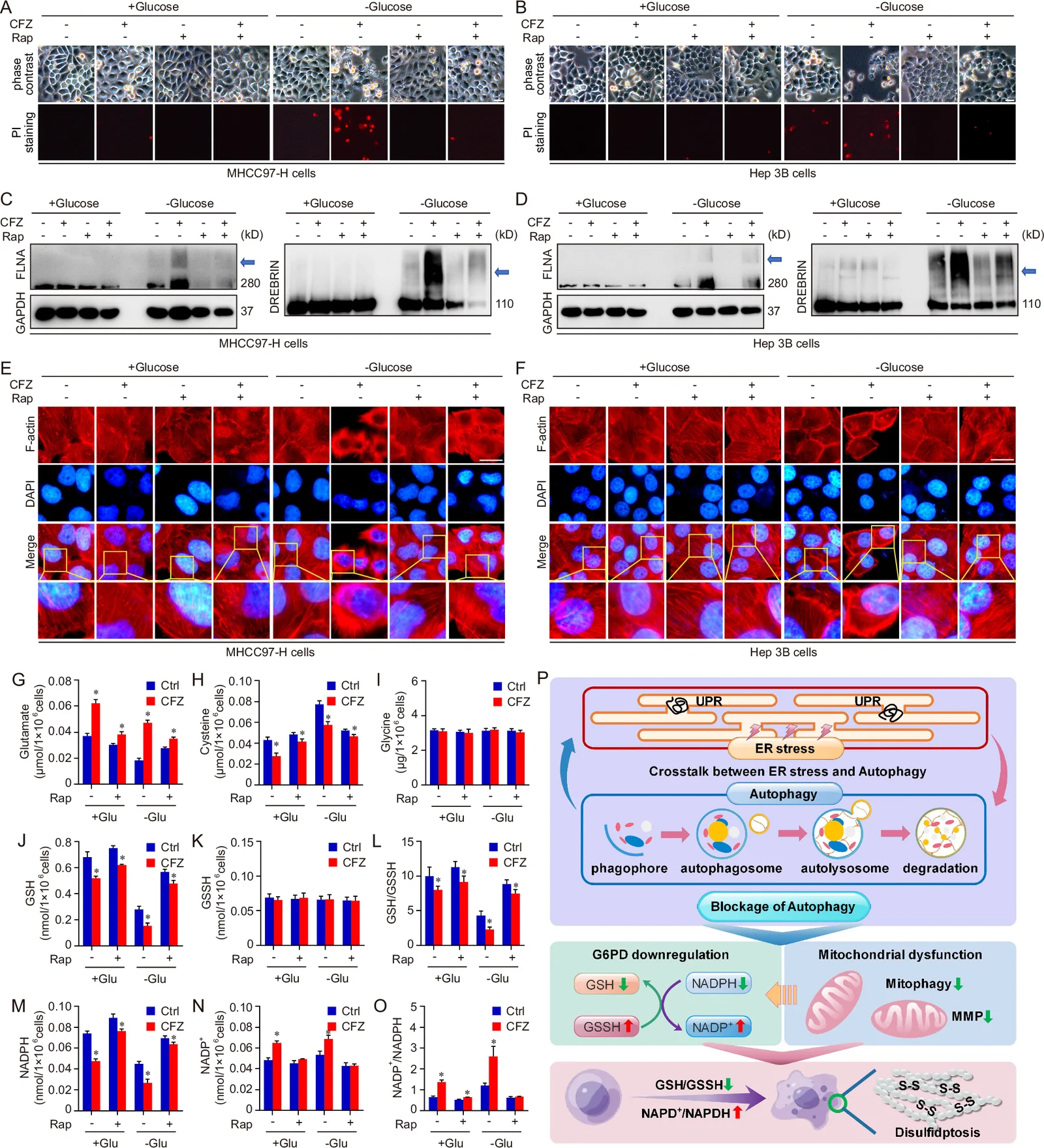
Clofazimine increases the sensitivity of HCC cells to disulfidptosis by inhibiting autophagy. To further evaluate the role of autophagy in the pharmacological effects of CFZ on disulfidptosis, autophagy activator, Rap was applied in the co-treatment model. Both MHCC97-H and Hep 3B cells were treated with CFZ (30 μM) and Rap (1 μM) for 16 hours, followed by glucose starvation (6–8 hours) to induce disulfidptosis. Then, the changes in morphology and cell death were assessed in each group (**A**, **B** Scale bar = 20 μm). Subsequently, the formation of disulfide bonds in cytoskeletal proteins (FLNA and DREBRIN) induced by glucose starvation was analyzed using a non-reducing western blot (**C**, **D**), and phalloidin staining was performed to evaluate the structure of F-actin across different groups (**E**, **F** Scale bar = 10 μm). The levels of glutamate (**G**), cysteine (**H**), and glycine (**I**) were measured in each group, and GSH metabolism (**J**–**L**) and NADPH metabolism (**M**–**O**) in various groups were evaluated, respectively. Finally, the proposed model for the relationship between autophagy and disulfidptosis was shown in (**P**). (Data are presented as means ± SD. *p < 0.05 compared to Ctrl groups).

The role of autophagy was further elucidated in the pharmacological effects of CFZ on disulfidptosis. In this study, the autophagy activator, Rap, was utilized during the process of HCC disulfidptosis triggered by glucose deprivation. Our findings demonstrated that CFZ treatment exacerbated cell death induced by glucose starvation, which could be completely inhibited by concurrent treatment with Rap (Fig. 8A, B). Furthermore, the non-reducing western blot analysis revealed that Rap treatment mitigated the slower migration of cytoskeletal proteins caused by CFZ treatment (Fig. 8C, D). Additionally, phalloidin staining illustrated that the co-treatment with Rap inhibited the effects of CFZ, rescuing glucose starvation-induced F-actin contraction and marginal clustering in HCC cells (Fig. 8E, F), thereby confirming the significant role of autophagy in the impact of CFZ on disulfidptosis. Moreover, the influence of CFZ on GSH synthesis and NADPH metabolism was also assessed in HCC cells. The results indicated that CFZ treatment elevated the glutamate levels while reducing cysteine levels in HCC cells, and the effects of CFZ could be diminished under the condition of Rap co-treatment (Fig. 8G–I). Similar to other autophagy inhibitors, CFZ treatment also led to a marked downregulation of GSH and NADPH. The GSH/GSSH ratio was reduced in CFZ-treated cells compared to untreated cells, while the NADP^+^/NADPH ratio was increased following CFZ treatment. However, all the aforementioned effects of CFZ could be inhibited by co-treatment with the autophagy activator, Rap, indicating that the impact of CFZ on the disulfidptosis susceptibility of HCC cells depended on the inhibition of autophagy (Fig. 8J–O).

Finally, a metabolomics analysis was conducted to assess the impact of CFZ on the metabolic profiles of HCC cells. Initially, the Model Overview and Permutation testing were executed, followed by the Loading S-plot (OPLS-DA) and the Score (OPLS-DA) plot, which confirmed the high quality of the various samples (Fig. S16A). The findings from the metabolomics analysis indicated that CFZ-treated HCC cells exhibited 123 downregulated metabolites and 189 upregulated metabolites in comparison to untreated cells (Fig. S16B and Table S4). CFZ treatment influenced all aspects of amino acid metabolism, carbohydrate metabolism, and lipid metabolism in HCC cells (Fig. S16C, D). Notably, the biosynthesis of unsaturated fatty acids and glycerophospholipid metabolism could play a central role in the metabolic network, potentially representing the underlying metabolic mechanism of CFZ’s action (Fig. S16E–G). Nevertheless, the precise role of unsaturated fatty acids and glycerophospholipids in disulfidptosis still requires further exploration.

## Discussion

As a newly recognized form of cell death, the fundamental molecular mechanisms of disulfidptosis still require detailed elucidation. Meanwhile, the interaction between disulfidptosis and classical survival regulatory pathways remains unclear and urgently necessitates thorough investigation. Autophagy, serving as a crucial checkpoint in determining cell fate, is likely to have complex regulatory effects during the disulfidptosis process. This study aims to focus on the significance of autophagy within the disulfidptosis model, examining its regulatory sites and underlying mechanisms. We found that inhibiting autophagy enhanced disulfidptosis in HCC cells both in vitro and in vivo. The downregulation of *G6PD* expression and the inhibition of the PPP could be the primary mechanisms involved. The suppression of autophagic flux resulted in reduced levels of GSH and NADPH, which depended on G6PD regulation. Furthermore, the inhibition of autophagy also led to the suppression of mitophagy and mitochondrial dysfunction, which could further contribute to the abnormal metabolism of NADPH due to the close relationship between mitochondrial function and NADPH metabolism. Additionally, our findings highlighted the significance of the interaction between ER stress and autophagy in regulating disulfidptosis, with the influence of ER stress on disulfidptosis being contingent upon autophagy regulation (Fig. 8P). The drug screening work identified a clinical agent, CFZ, as a potential candidate for targeting the homeostasis between ER stress and autophagy. Taken together, these findings provide new insights into the development of combined intervention strategies for HCC treatment.

The core mechanism of disulfidptosis depends on the disulfide stress formed by the accumulation of cysteine. GSH and NADPH are the crucial reducing systems for scavenging these reactive substances [2, 25, 37]. In addition, autophagy plays a central role in maintaining redox balance through its impact on different metabolic processes [10, 38]. Research findings indicate that autophagy can indirectly decrease the production of ROS by facilitating the clearance of damaged mitochondria and peroxisomes [39, 40]. Consequently, the consumption of GSH and NADPH could be diminished via regulating autophagy. For instance, the activation of autophagy is crucial for enhancing the membrane localization of solute carrier family 7 member 11 (SLC7A11), which preserves cystine transport capability and thereby influences GSH synthesis [18]. Additionally, NADPH is primarily produced through the PPP, and the activation of autophagy can liberate glucose from glycogen granules, supplying substrates for glycolysis and the PPP to produce ATP, NADPH, and pyruvate for energy metabolism [41–43]. Furthermore, some studies have suggested that PI3K/mTOR inhibitors significantly promote the autophagic degradation of G6PD and contribute to oxidative stress damage in small cell lung cancer cells [44]. Therefore, the metabolic processes of both GSH and NADPH can be modulated by autophagy through various pathways and processes, and autophagy may influence the redox buffering capacity of cells under disulfide stress, thereby affecting their susceptibility to disulfidptosis.

Our present research primarily demonstrated that the inhibition of autophagic flux increased the susceptibility of HCC cells to disulfidptosis, whereas the activation of autophagy provided protection against disulfidptosis induced by glucose starvation. Nonetheless, the role of autophagy varies in different forms of cell death. For instance, the activation of autophagy facilitated the process of ferritinophagy and intensified ferroptosis in cells, while mitophagy also plays a crucial role in the induction of ferroptosis [12, 45]. Notably, excessive autophagy activation can lead to autophagic cell death, which is another form of non-apoptotic PCD characterized by a significant accumulation of autophagic vacuoles within the cells. Furthermore, autophagic cell death is often accompanied by the suppression of mTOR activity and the stimulation of AMPK [46, 47]. Generally, autophagy acts as a protective mechanism that promotes cell survival under stress conditions. However, under certain circumstances, such as overactivation or dysregulated, autophagy may result in cellular energy depletion and the disruption of essential cellular functions, ultimately leading to cell death. Related studies also suggest that autophagic cell death is frequently linked to various biological responses and plays a vital role in numerous diseases [10, 48]. Consequently, autophagy serves multiple functions across various cell death models, which must be considered when formulating new therapeutic strategies against cancer that utilize autophagy modulators.

Mitochondria serve as central regulators of energy metabolism, ROS generation and cellular death pathways. Mitophagy, a selective form of autophagy, plays a pivotal role in removing dysfunctional mitochondria, attenuating excessive ROS production and maintaining the efficiency of ATP synthesis. Primarily, it facilitates the ubiquitination of damaged mitochondria and promotes the recruitment of autophagosomes for their degradation via the PINK1/PARK2 pathway [49, 50]. Our study indicated that the blockage of autophagy suppressed mitophagy as well as PINK1/PARK2 signal pathway, revealing that PINK1/PARK2-mediated mitophagy exerts protective effects by alleviating oxidative damage induced by disulfide stress, thereby potentially inhibiting the progression of disulfidptosis. Some research has indicated that a moderate level of mitophagy activation is crucial for maintaining MMP, supporting ATP production, and regulating ROS homeostasis [51, 52]. Furthermore, our study found that inhibiting mitophagy led to a reduction in MMP and mitochondrial dysfunction, while the compound that enhances mitochondrial function provided protection against disulfidptosis. Notably, mitochondria play a significant role in the metabolism of GSH and NADPH [53, 54], and mitochondrial dysfunction may lead to disturbances in GSH and NADPH metabolism, underscoring the critical role of mitochondria in disulfidptosis. Nevertheless, the specific relationship between mitochondria and disulfidptosis requires further exploration.

CFZ, a well-known anti-leprosy medication, has gained increasing recognition for its potential to enhance cancer treatment in recent years [55, 56]. For instance, CFZ treatment facilitates E2F1 activation in CD8-positive T cells and counteracts pathogenic Th17 cells to overcome resistance, thereby improving the anti-cancer efficacy of anti-PD-1 and CTLA-4 immunotherapy [55]. Furthermore, some researchers have investigated the connection between CFZ and autophagy, discovering that the combination of CFZ and Rap effectively eliminates drug-resistant tuberculosis by enhancing polyfunctional central memory T-cell responses [57]. Nevertheless, the precise impact of CFZ on autophagy remains uncertain. In our current study, CFZ was recognized as an effective agent targeting ER stress and autophagy. Treatment with CFZ inhibited both autophagy and ER stress, while also increasing the sensitivity of HCC cells to disulfidptosis. Considering the benefits of CFZ in clinical settings, it presents itself as a promising candidate for combination drug therapy aimed at HCC through the regulation of disulfidptosis.

The clinical application of disulfidptosis presents significant potential, especially for tumors that exhibit a high resistance to standard treatments. By leveraging a distinct metabolic weakness, disulfidptosis provides a new avenue for the targeted destruction of malignant cells. Ongoing studies have already shown its capability in improving chemotherapy responsiveness, addressing drug resistance, and forming new prognostic frameworks for various cancers. The primary mechanism for triggering disulfidptosis involves inducing a state of glucose deprivation and NADPH depletion, particularly in cells that express high levels of SLC7A11. Nevertheless, the current in vivo therapeutic approaches remain limited. Scientists have established that disulfidptosis can be effectively triggered by glucose deprivation. However, inducing glucose deprivation within the tumor microenvironment is particularly challenging. It is advisable to directly restrict glucose supply or inhibit glucose uptake through GLUT transporters (for instance, BAY-876) as a fundamental strategy. Consequently, a high-throughput drug screening model is crucial for the advancement of more effective compounds that target glucose metabolism, which is rapidly converting this innovative cell death mechanism into a practical and potent therapeutic option in clinical settings.

## Conclusion

In conclusion, our research has identified autophagy as a key regulator of disulfidptosis in HCC cells. The suppression of autophagic flux enhances the susceptibility of HCC cells to disulfidptosis by decreasing the rate-limiting enzyme in the PPP, G6PD, and influencing GSH and NADPH metabolism. Furthermore, the inhibition of autophagy resulted in reduced mitophagy and subsequent mitochondrial dysfunction, which may also contribute to the disruptions of GSH and NADPH metabolism, thereby heightening the vulnerability of HCC to disulfidptosis. Additionally, we discovered that the effect of ER stress on disulfidptosis was contingent upon the regulation of autophagy, underscoring the significance of maintaining homeostasis between ER stress and autophagy. CFZ, a clinically approved drug for treating mycobacterial infections, was recognized as an effective agent for targeting this homeostasis, offering a novel therapeutic strategy against HCC in clinical settings through the regulation of disulfidptosis. Overall, this study not only elucidates the fundamental mechanisms that connect autophagy to disulfidptosis but also presents a clinically viable approach for HCC treatment, positioning organelle-targeted autophagy modulation as an innovative strategy to overcome resistance in HCC treatment.

## Supplementary information

Supplementary Materials

Original images of western blots

Reproducibility checklist

## Data Availability

All data generated or analyzed during this study are included in this published article and its supplementary information files.

## References

[1] Liu X, Nie L, Zhang Y, Yan Y, Wang C, Colic M, et al. Actin cytoskeleton vulnerability to disulfide stress mediates disulfidptosis. Nat Cell Biol. 2023;25:404–14. 10.1038/s41556-023-01091-2 PMC10027392 36747082

[2] Liu X, Zhuang L, Gan B. Disulfidptosis: disulfide stress-induced cell death. Trends Cell Biol. 2024;34:327–37. 10.1016/j.tcb.2023.07.009 37574347

[3] Liu X, Olszewski K, Zhang Y, Lim EW, Shi J, Zhang X, et al. Cystine transporter regulation of pentose phosphate pathway dependency and disulfide stress exposes a targetable metabolic vulnerability in cancer. Nat Cell Biol. 2020;22:476–86. 10.1038/s41556-020-0496-x PMC7194135 32231310

[4] Liu XG, Zhuang L, Gan BY. Disulfidptosis: disulfide stress-induced cell death. Trends Cell Biol. 2024;34:327–37. 10.1016/j.tcb.2023.07.009 37574347

[5] Wang J, Chen J, Fan K, Wang M, Gao M, Ren Y, et al. Inhibition of endoplasmic reticulum stress cooperates with SLC7A11 to promote disulfidptosis and suppress tumor growth upon glucose limitation. Adv Sci. 2025;12:e2408789. 10.1002/advs.202408789 PMC11831432 39739602

[6] Debnath J, Gammoh N, Ryan KM. Autophagy and autophagy-related pathways in cancer. Nat Rev Mol Cell Biol. 2023;24:560–75. 10.1038/s41580-023-00585-z PMC9980873 36864290

[7] Feng Y, Chen Y, Wu X, Chen J, Zhou Q, Liu B, et al. Interplay of energy metabolism and autophagy. Autophagy. 2024;20:4–14. 10.1080/15548627.2023.2247300 PMC10761056 37594406

[8] Shi M, Zhang J, Li M, Zhao Y, Guo Y, Xu J, et al. Liquiritigenin confers liver protection by enhancing NRF2 signaling through both canonical and non-canonical signaling pathways. J Med Chem. 2023;66:11324–34. 10.1021/acs.jmedchem.3c00815 37534604

[9] Jung S, Choe S, Woo H, Jeong H, An HK, Moon H, et al. Autophagic death of neural stem cells mediates chronic stress-induced decline of adult hippocampal neurogenesis and cognitive deficits. Autophagy. 2020;16:512–30. 10.1080/15548627.2019.1630222 PMC6999625 31234698

[10] Liu S, Yao S, Yang H, Liu S, Wang Y. Autophagy: regulator of cell death. Cell Death Dis. 2023;14:648. 10.1038/s41419-023-06154-8 PMC10551038 37794028

[11] Yang WS, Stockwell BR. Ferroptosis: death by lipid peroxidation. Trends Cell Biol. 2016;26:165–76. 10.1016/j.tcb.2015.10.014 PMC4764384 26653790

[12] Chen X, Tsvetkov AS, Shen HM, Isidoro C, Ktistakis NT, Linkermann A, et al. International consensus guidelines for the definition, detection, and interpretation of autophagy-dependent ferroptosis. Autophagy. 2024;20:1213–46. 10.1080/15548627.2024.2319901 PMC11210914 38442890

[13] Zhao P, Yin S, Qiu Y, Sun C, Yu H. Ferroptosis and pyroptosis are connected through autophagy: a new perspective of overcoming drug resistance. Mol Cancer. 2025;24:23. 10.1186/s12943-024-02217-2 PMC11740669 39825385

[14] Cybulsky AV. Endoplasmic reticulum stress, the unfolded protein response and autophagy in kidney diseases. Nat Rev Nephrol. 2017;13:681–96. 10.1038/nrneph.2017.129 28970584

[15] Chino H, Mizushima N. ER-phagy: quality and quantity control of the endoplasmic reticulum by autophagy. Cold Spring Harb Perspect Biol. 2023;15:a041256. 10.1101/cshperspect.a041256 PMC9808580 35940904

[16] Naama M, Bel S. Autophagy-ER stress crosstalk controls mucus secretion and susceptibility to gut inflammation. Autophagy. 2023;19:3014–6. 10.1080/15548627.2023.2228191 PMC10549187 37436421

[17] Mukhopadhyay S, Biancur DE, Parker SJ, Yamamoto K, Banh RS, Paulo JA, et al. Autophagy is required for proper cysteine homeostasis in pancreatic cancer through regulation of SLC7A11. Proc Natl Acad Sci USA. 2021;118:e2021475118. 10.1073/pnas.2021475118 PMC8017731 33531365

[18] Liu J, Huang C, Liu J, Meng C, Gu Q, Du X, et al. Nrf2 and its dependent autophagy activation cooperatively counteract ferroptosis to alleviate acute liver injury. Pharm Res. 2023;187:106563. 10.1016/j.phrs.2022.106563 36410674

[19] Xin Y, Wang Q, Yang J, Wu X, Xia Y, Xun L, et al. Polysulfides promote protein disulfide bond formation in microorganisms growing under anaerobic conditions. Appl Environ Microbiol. 2025;91:e0192624. 10.1128/aem.01926-24 PMC11921322 39918318

[20] Xu Y, Ji Y, Li X, Ding J, Chen L, Huang Y, et al. URI1 suppresses irradiation-induced reactive oxygen species (ROS) by activating autophagy in hepatocellular carcinoma cells. Int J Biol Sci. 2021;17:3091–103. 10.7150/ijbs.55689 PMC8375238 34421352

[21] Garza-Lombo C, Pappa A, Panayiotidis MI, Franco R. Redox homeostasis, oxidative stress and mitophagy. Mitochondrion. 2020;51:105–17. 10.1016/j.mito.2020.01.002 PMC7062406 31972372

[22] Li J, Wang Y, Luo Y, Liu Y, Yi Y, Li J, et al. USP5-Beclin 1 axis overrides p53-dependent senescence and drives Kras-induced tumorigenicity. Nat Commun. 2022;13:7799. 10.1038/s41467-022-35557-y PMC9759531 36528652

[23] Schmid ET, Schinaman JM, Liu-Abramowicz N, Williams KS, Walker DW. Accumulation of F-actin drives brain aging and limits healthspan in Drosophila. Nat Commun. 2024;15:9238. 10.1038/s41467-024-53389-w PMC11512044 39455560

[24] Kast DJ, Dominguez R. The cytoskeleton-autophagy connection. Curr Biol. 2017;27:R318–26. 10.1016/j.cub.2017.02.061 PMC5444402 28441569

[25] Shi M, Li X, Guo Y, Zhang Y, Xu J, Yan L, et al. Gaudichaudione H enhances the sensitivity of hepatocellular carcinoma cells to disulfidptosis via regulating NRF2-SLC7A11 signaling pathway. Adv Sci. 2025;12:e2411131. 10.1002/advs.202411131 PMC11923960 39840687

[26] Li M, Wang Y, Li X, Xu J, Yan L, Tang S, et al. Pharmacological targeting of the mitochondrial phosphatase PTPMT1 sensitizes hepatocellular carcinoma to ferroptosis. Cell Death Dis. 2025;16:257. 10.1038/s41419-025-07581-5 PMC11973169 40189563

[27] Li M, Xu J, Du K, Wang G, Feng Y, Liu P. Alexidine dihydrochloride enhances the sensitivity of human hepatocellular carcinoma to disulfidptosis via ATF4-DDIT3 activation. Free Radic Biol Med. 2025;237:585–99. 10.1016/j.freeradbiomed.2025.06.020 40518125

[28] Shi M, Guo Y, Xu J, Yan L, Li X, Liu R, et al. Gaudichaudione H ameliorates liver fibrosis and inflammation by targeting NRF2 signaling pathway. Free Radic Biol Med. 2024;224:15. 10.1016/j.freeradbiomed.2024.09.020 39313014

[29] Feng Y, Shi M, Zhang Y, Li X, Yan L, Xu J, et al. Protocatechuic acid relieves ferroptosis in hepatic lipotoxicity and steatosis via regulating NRF2 signaling pathway. Cell Biol Toxicol. 2024;40:104. 10.1007/s10565-024-09953-7 PMC11599353 39589556

[30] Daina A, Michielin O, Zoete V. Swiss target prediction: updated data and new features for efficient prediction of protein targets of small molecules. Nucleic Acids Res. 2019;47:W357–64. 10.1093/nar/gkz382 PMC6602486 31106366

[31] Yao ZJ, Dong J, Che YJ, Zhu MF, Wen M, Wang NN, et al. TargetNet: a web service for predicting potential drug-target interaction profiling via multi-target SAR models. J Comput Aided Mol Des. 2016;30:413–24. 10.1007/s10822-016-9915-2 27167132

[32] Zhou YY, Zhou B, Pache L, Chang M, Khodabakhshi AH, Tanaseichuk O, et al. Metascape provides a biologist-oriented resource for the analysis of systems-level datasets. Nat Commun. 2019;10:1523. 10.1038/s41467-019-09234-6 PMC6447622 30944313

[33] Piñero J, Ramírez-Anguita JM, Saüch-Pitarch J, Ronzano F, Centeno E, Sanz F, et al. The DisGeNET knowledge platform for disease genomics: 2019 update. Nucleic Acids Res. 2020;48:D845–55. 10.1093/nar/gkz1021 PMC7145631 31680165

[34] Szklarczyk D, Gable AL, Nastou KC, Lyon D, Kirsch R, Pyysalo S, et al. The STRING database in 2021: customizable protein-protein networks, and functional characterization of user-uploaded gene/measurement sets. Nucleic Acids Res. 2021;49:D605–12. 10.1093/nar/gkaa1074 PMC7779004 33237311

[35] Yao HF, Ge J, Chen J, Tang X, Li C, Hu X, et al. CASC8 activates the pentose phosphate pathway to inhibit disulfidptosis in pancreatic ductal adenocarcinoma though the c-Myc-GLUT1 axis. J Exp Clin Cancer Res. 2025;44:26. 10.1186/s13046-025-03295-w PMC11771065 39865281

[36] Qi Z, Chen L. Endoplasmic reticulum stress and autophagy. Adv Exp Med Biol. 2019;1206:167–77. 10.1007/978-981-15-0602-4_8 31776985

[37] Ferrer M, Mourikis N, Davidson EE, Kleeman SO, Zaccaria M, Habel J, et al. Ketogenic diet promotes tumor ferroptosis but induces relative corticosterone deficiency that accelerates cachexia. Cell Metab. 2023;35:1147–62. 10.1016/j.cmet.2023.05.008 PMC11037504 37311455

[38] Shu F, Xiao H, Li QN, Ren XS, Liu ZG, Hu BW, et al. Epigenetic and post-translational modifications in autophagy: biological functions and therapeutic targets. Signal Transduct Target Ther. 2023;8:32. 10.1038/s41392-022-01300-8 PMC9842768 36646695

[39] Wilhelm LP, Ganley IG. Mitochondria and peroxisomes: partners in autophagy. Autophagy. 2023;19:2162–3. 10.1080/15548627.2022.2155368 PMC10283438 36572844

[40] Liu J, Wu Y, Meng S, Xu P, Li S, Li Y, et al. Selective autophagy in cancer: mechanisms, therapeutic implications, and future perspectives. Mol Cancer. 2024;23:22. 10.1186/s12943-024-01934-y PMC10807193 38262996

[41] Feng YY, Chen Y, Wu XY, Chen JY, Zhou QY, Liu B, et al. Interplay of energy metabolism and autophagy. Autophagy. 2024;20:4–14. 10.1080/15548627.2023.2247300 PMC10761056 37594406

[42] Koutsifeli P, Varma U, Daniels LJ, Annandale M, Li X, Neale JPH, et al. Glycogen-autophagy: molecular machinery and cellular mechanisms of glycophagy. J Biol Chem. 2022;298:102093. 10.1016/j.jbc.2022.102093 PMC9249846 35654138

[43] Niu X, Stancliffe E, Gelman SJ, Wang L, Schwaiger-Haber M, Rowles JL 3rd, et al. Cytosolic and mitochondrial NADPH fluxes are independently regulated. Nat Chem Biol. 2023;19:837–45. 10.1038/s41589-023-01283-9 PMC12175224 36973440

[44] Deng H, Chen YM, Wang L, Zhang YB, Hang QQ, Li PJ, et al. PI3K/mTOR inhibitors promote G6PD autophagic degradation and exacerbate oxidative stress damage to radiosensitize small cell lung cancer. Cell Death Dis. 2023;14:652. 10.1038/s41419-023-06171-7 PMC10558571 37802999

[45] Tang D, Chen X, Kang R, Kroemer G. Ferroptosis: molecular mechanisms and health implications. Cell Res. 2021;31:107–25. 10.1038/s41422-020-00441-1 PMC8026611 33268902

[46] Wang G, Dai S, Chen J, Zhang K, Huang C, Zhang J, et al. USP19 potentiates autophagic cell death via inhibiting mTOR pathway through deubiquitinating NEK9 in pancreatic cancer. Cell Death Differ. 2025;32:702–13. 10.1038/s41418-024-01426-y PMC11982380 39627360

[47] Song CF, Hu YH, Mang ZG, Ye Z, Chen HD, Jing DS, et al. Hernandezine induces autophagic cell death in human pancreatic cancer cells via activation of the ROS/AMPK signaling pathway. Acta Pharm Sin. 2023;44:865–76. 10.1038/s41401-022-01006-1 PMC10042859 36284209

[48] Park W, Wei S, Kim BS, Kim B, Bae SJ, Chae YC, et al. Diversity and complexity of cell death: a historical review. Exp Mol Med. 2023;55:1573–94. 10.1038/s12276-023-01078-x PMC10474147 37612413

[49] Picca A, Faitg J, Auwerx J, Ferrucci L, D’Amico D. Mitophagy in human health, ageing and disease. Nat Metab. 2023;5:2047–61. 10.1038/s42255-023-00930-8 PMC12159423 38036770

[50] Wang S, Long H, Hou L, Feng B, Ma Z, Wu Y, et al. The mitophagy pathway and its implications in human diseases. Signal Transduct Target Ther. 2023;8:304. 10.1038/s41392-023-01503-7 PMC10427715 37582956

[51] Song Y, Liang H, Li G, Ma L, Zhu D, Zhang W, et al. The NLRX1-SLC39A7 complex orchestrates mitochondrial dynamics and mitophagy to rejuvenate intervertebral disc by modulating mitochondrial Zn(2+) trafficking. Autophagy. 2024;20:809–29. 10.1080/15548627.2023.2274205 PMC11062375 37876250

[52] Sun K, Jing X, Guo J, Yao X, Guo F. Mitophagy in degenerative joint diseases. Autophagy. 2021;17:2082–92. 10.1080/15548627.2020.1822097 PMC8496714 32967533

[53] Zhu J, Schworer S, Berisa M, Kyung YJ, Ryu KW, Yi J, et al. Mitochondrial NADP(H) generation is essential for proline biosynthesis. Science. 2021;372:968–72. 10.1126/science.abd5491 PMC8241437 33888598

[54] Wang Y, Yen FS, Zhu XG, Timson RC, Weber R, Xing C, et al. SLC25A39 is necessary for mitochondrial glutathione import in mammalian cells. Nature. 2021;599:136–40. 10.1038/s41586-021-04025-w PMC10981497 34707288

[55] Xue G, Li X, Kalim M, Fang J, Jiang Z, Zheng N, et al. Clinical drug screening reveals clofazimine potentiates the efficacy while reducing the toxicity of anti-PD-1 and CTLA-4 immunotherapy. Cancer Cell. 2024;42:17. 10.1016/j.ccell.2024.03.001 PMC11756590 38518774

[56] Kraehenbuehl L, Wolchok JD, Merghoub T, Hirschhorn D. Having the cake and eating it? Clofazimine boosts immunotherapy while limiting side effects. Cancer Cell. 2024;42:738–41. 10.1016/j.ccell.2024.03.007 38579723

[57] Singh DK, Bhaskar A, Pahuja I, Shaji A, Moitra B, Shi Y, et al. Cotreatment with clofazimine and rapamycin eliminates drug-resistant tuberculosis by inducing polyfunctional central memory t-cell responses. J Infect Dis. 2023;228:1166–78. 10.1093/infdis/jiad214 37290049

