# Supplementary material for: Autophagic flux inhibition potentiates disulfidptosis susceptibility in human hepatocellular carcinoma
Source: Cell Death Dis. 2026 Aug 11;17(1):948. doi: 10.1038/s41419-026-09135-9 (PMC13745592; doi:10.1038/s41419-026-09135-9)
Supplement: Supplementary file 1 [file 41419_2026_9135_MOESM1_ESM.pdf]

Supplementary Materials for

Autophagic Flux Inhibition Potentiates Disulfidptosis Susceptibility in Human Hepatocellular Carcinoma

Mengjiao Shi [1,2,3], Liangwen Yan [2], Kailing Hu [2], Lin Li [2], Jiayi Xu [2], Xinyan Li [2], Rongrong Liu [2,3], Yinggang Zhang [2,3], Yaping Zhao [2,3], Ying Guo [2,3], Hong Wang [2], Yetong Feng [2,4], Xu Gao [5], Enrui Xie [5], Fanpu Ji [5], Gang Wang [1,6], Pengfei Liu [1,2,3,7]*

1. Department of Critical Care Medicine, National & Local Joint Engineering Research Center of Biodiagnosis and Biotherapy, The Second Affiliated Hospital of Xi'an Jiaotong University, Xi'an 710004, China
2. International Joint Research Center on Cell Stress and Disease Diagnosis and Therapy, National & Local Joint Engineering Research Center of Biodiagnosis and Biotherapy, The Second Affiliated Hospital of Xi'an Jiaotong University, Xi'an 710004, China
3. Shaanxi Provincial Clinical Research Center for Hepatic & Splenic Diseases, The Second Affiliated Hospital of Xi'an Jiaotong University, Xi'an 710004, China
4. Core Research Laboratory, The Second Affiliated Hospital of Xi'an Jiaotong University, Xi'an 710004, China
5. Department of Hepatology, the Second Affiliated Hospital of Xi'an Jiaotong University, Xi'an 710004, China.
6. Key Laboratory of Surgical Critical Care and Life Support, Xi'an Jiaotong University, Ministry of Education of China, Xi'an 710004, China
7. Key Laboratory of Environment and Genes Related To Diseases, Xi'an Jiaotong University, Ministry of Education of China, Xi'an 710004, China

* Corresponding author:
Pengfei Liu, E-mail address: liupengfei@xjtu.edu.cn

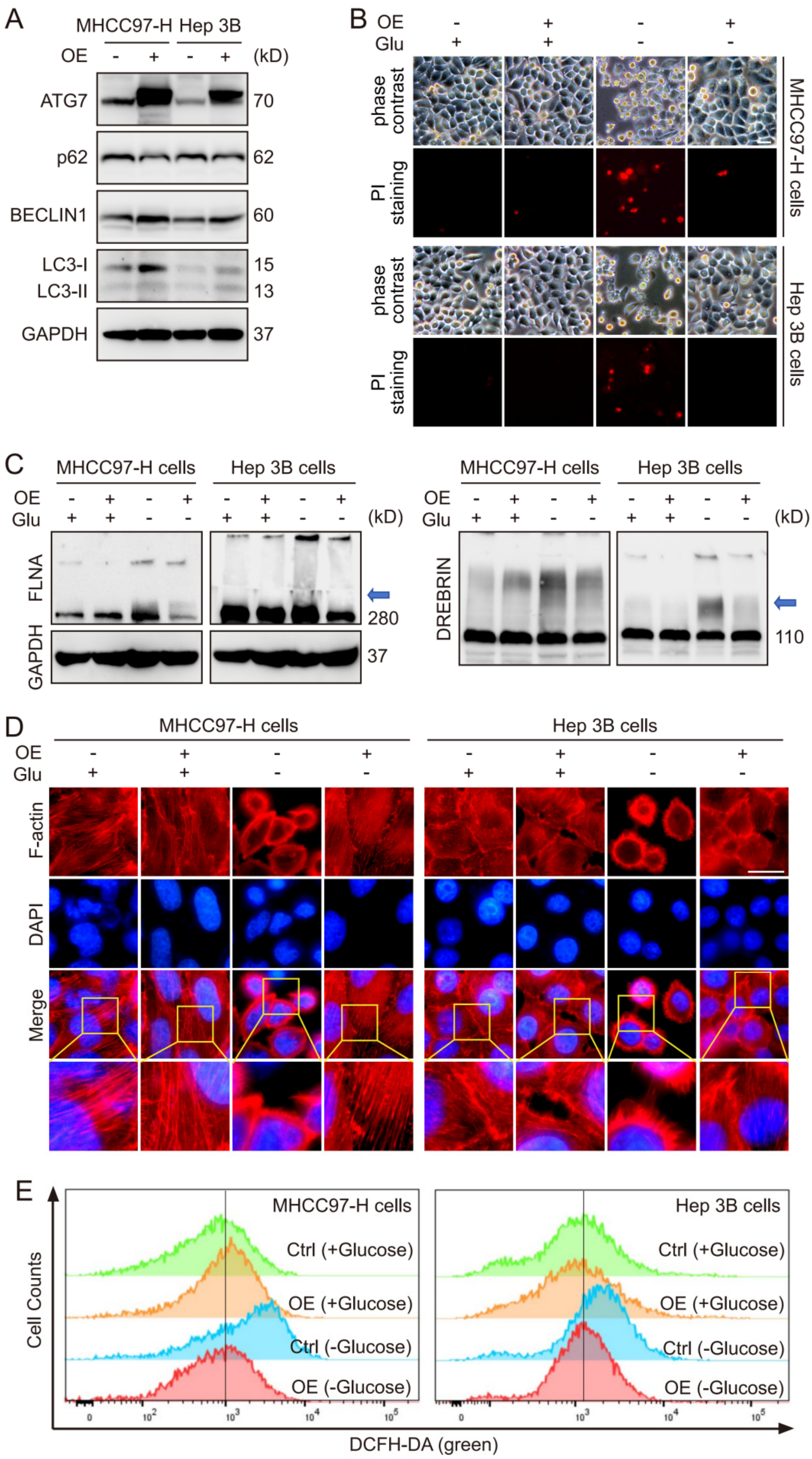

Figure S1. *ATG7* overexpression protects HCC cells from glucose deprivation-induced disulfidptosis. The overexpression of *ATG7* in HCC cells was induced via pCMV-ATG7 transfection, and the overexpression effect was confirmed using western blot (A). The normal cells and overexpressed cells were then followed by glucose

starvation to induce disulfidptosis. Then, the changes in morphology and cell death were assessed in each group (B, Scale bar=20 μm). The formation of disulfide bonds in cytoskeletal proteins (FLNA and DREBRIN) induced by glucose starvation was analyzed using non-reducing western blot (C). Subsequently, phalloidin staining was performed to evaluate the structure of F-actin across different groups (D, Scale bar=10 μm). Finally, the ROS levels in different groups were evaluated via DCFH-DA staining and flow cytometry analysis (E).

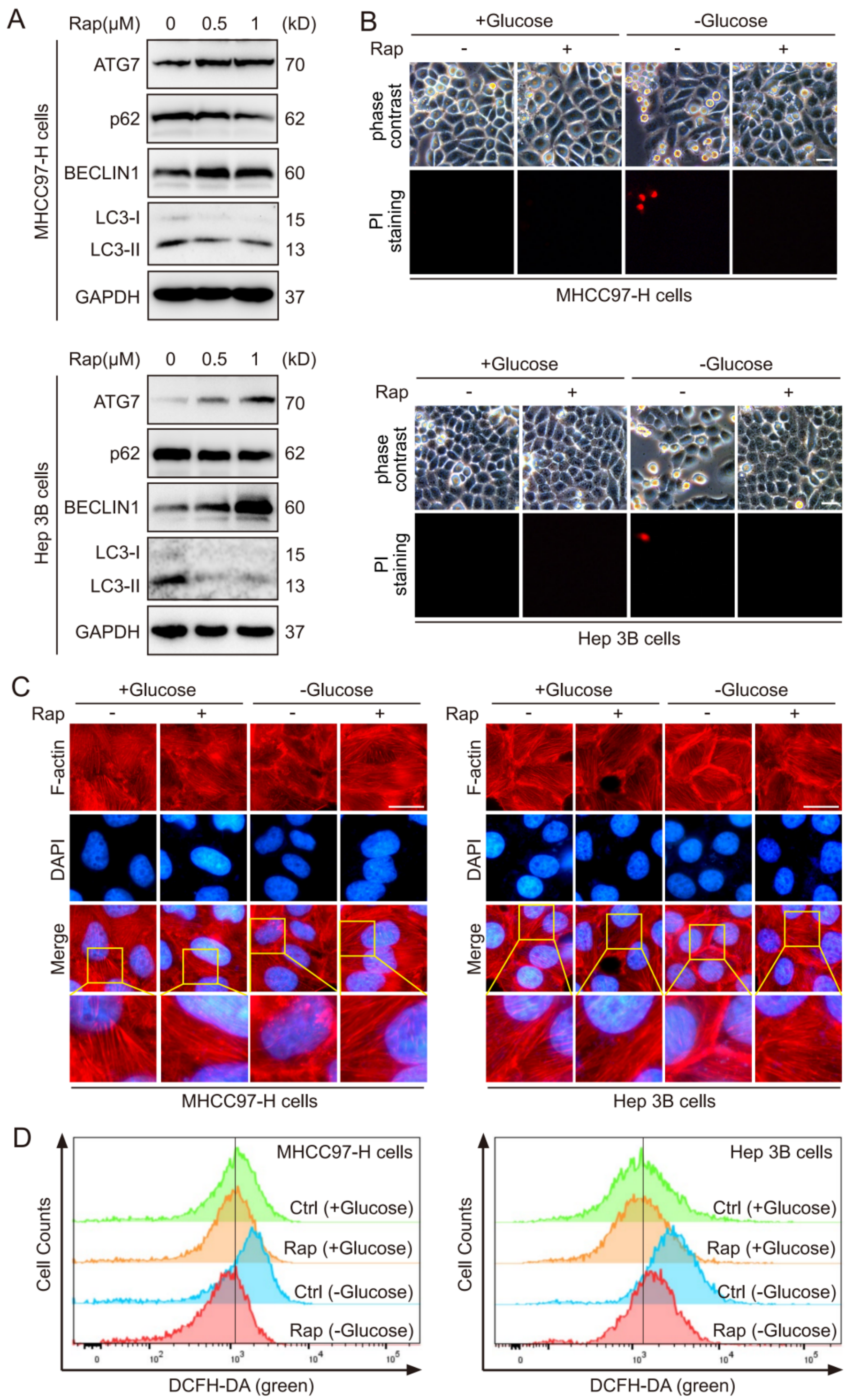

Figure S2. Chemical autophagy activator protects HCC cells from glucose deprivation-induced disulfidptosis. Both MHCC97-H and Hep 3B cells were treated with Rapamycin (Rap) for 16 hours, and the effect of Rap on autophagy makers was confirmed using western blot (A). The treated cells were then followed by glucose starvation to induce disulfidptosis. Then, the changes in morphology and cell death were assessed in each group (B, Scale bar=20 μm). Subsequently, phalloidin staining was performed to evaluate the structure of F-actin across different groups (C, Scale bar=10 μm). Finally, the ROS levels in different groups were evaluated via DCFH-DA staining and flow cytometry analysis (D).

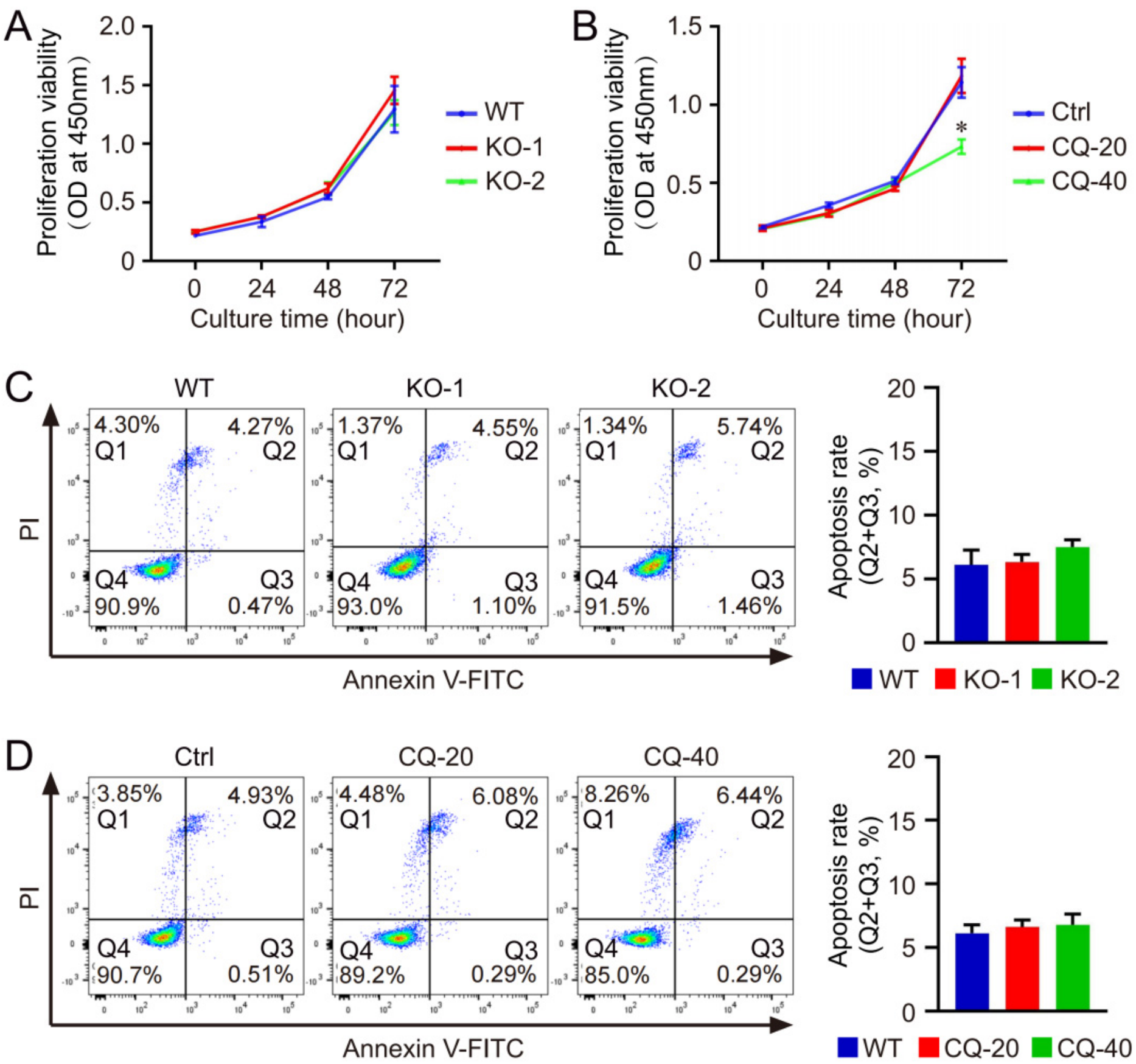

Figure S3. The effect of autophagy suppression on cell viability and cell death. Herein, both ATG7 knockout and CQ (20 and 40 μM) treatment were applied to suppress autophagy in HCC cells, and the cell viability was measured using CCK-8 method (A-B). In addition, the level of apoptosis in HCC cells was assessed using Annexin V-FITC/PI staining (C-D). (Data are presented as means ± SD. *: $p < 0.05$ compared to Ctrl group)

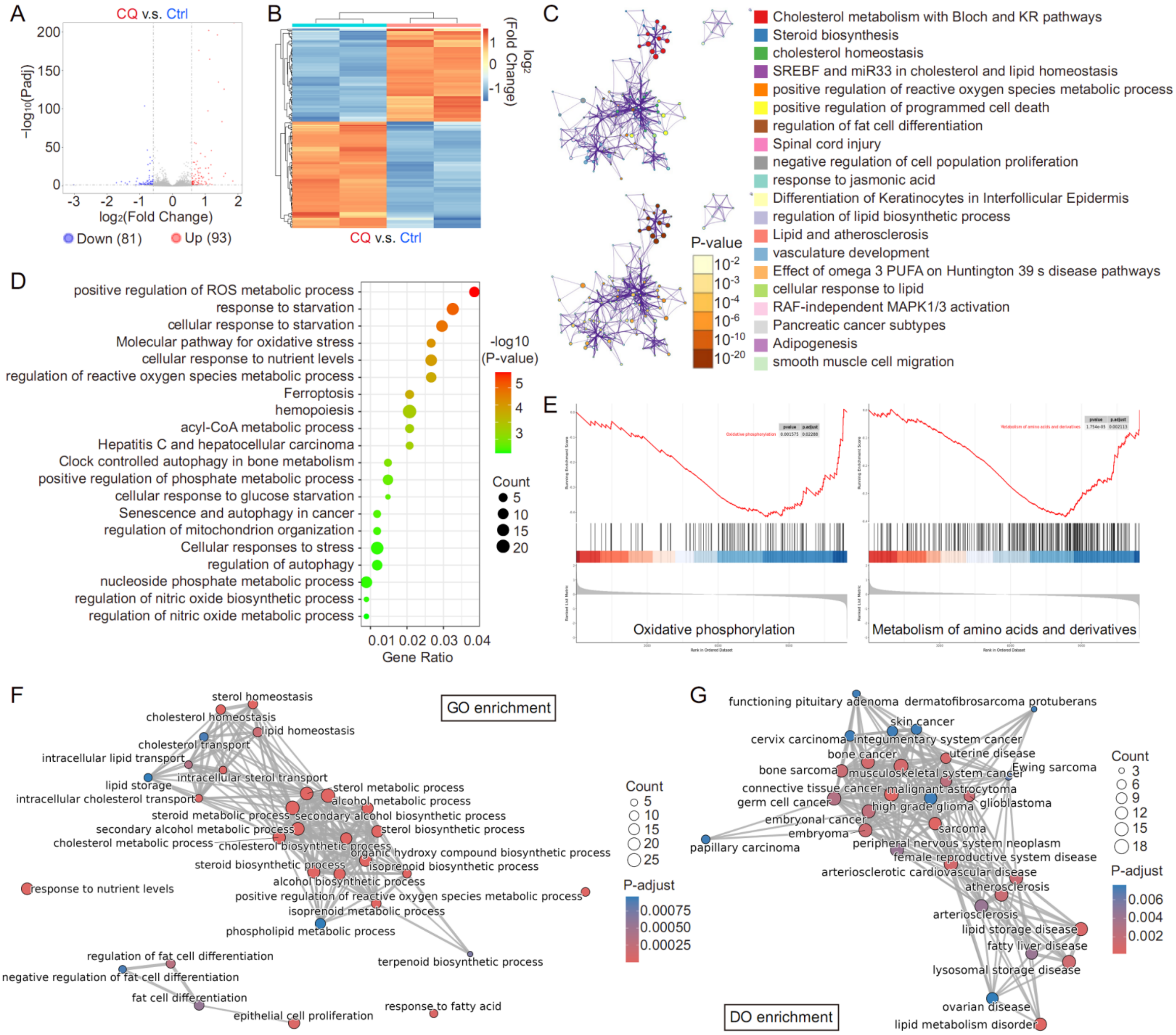

Figure S4. CQ-induced autophagy suppression influences various genes associated with metabolic reprogramming and mitochondrial dysfunction. To further assess the function of autophagy in disulfidptosis, both CQ-treated (40 μM for 16 hours) and untreated MHCC97-H cells were gathered for RNA-seq analysis. A volcano plot was created to assess the impact of autophagy blockage on gene transcription (A). Additionally, both up-regulated and down-regulated genes were organized in a heatmap (B), and utilized for cluster analysis through Metascape. The network of enriched terms for differentially expressed genes was color-coded by cluster ID, and the same terms were further distinguished by p-value (C). Moreover, several enriched terms associated with metabolic reprogramming and mitochondrial dysfunction were presented in (D). Additionally, the differentially expressed genes were further investigated through Gene Set Enrichment Analysis (GSEA) (E). Finally, the differentially expressed genes in RNA-sequencing were analyzed via GO enrichment (F) and DO enrichment (G) .

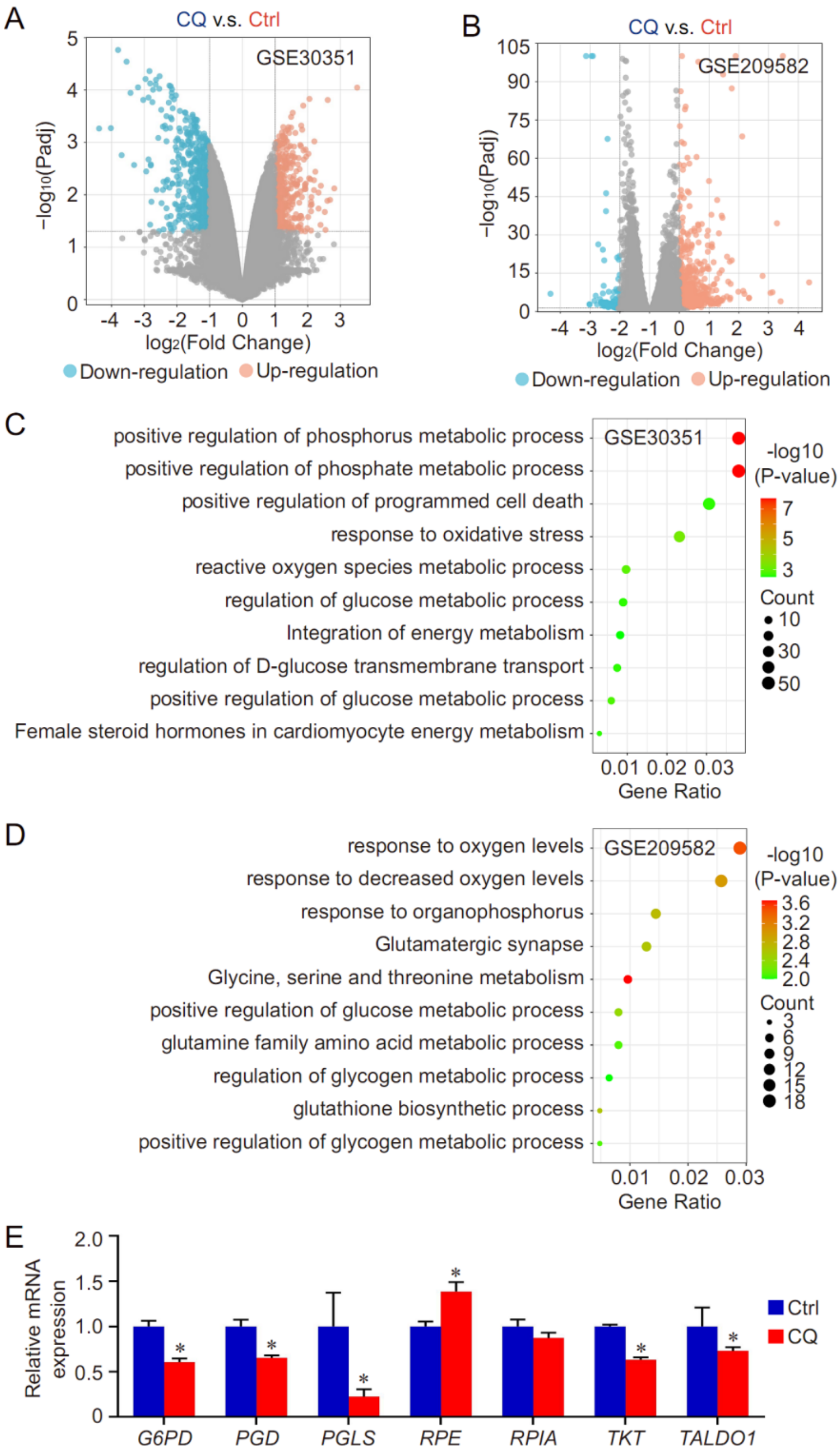

Figure S5. Analysis of mechanism regarding the impact of autophagy inhibitor. Herein, two distinct transcriptomic datasets (GSE30351 and GSE209582) sourced from the GEO database were used to analyze the function of autophagy inhibitor, chloroquine (CQ).Volcano plots were created to assess the impact of CQ treatment on gene transcription (A-B). Additionally, the differently expressed genes were utilized for cluster analysis through Metascape. The enriched terms associated with oxidative stress, glucose metabolic processes, and amino acid metabolic processes were presented in (C-D). Then, MHCC97-H cells were treated with CQ for 16 hours, the transcription of several key genes (*G6PD*, *PGD*, *PGLS*, *RPE*, *RPIA*, *TKT* and *TALDO1*) involved in the PPP was evaluated using Real-Time qRT-PCR (E). (Data are presented as means ± SD. *: $p < 0.05$ compared to Ctrl group)

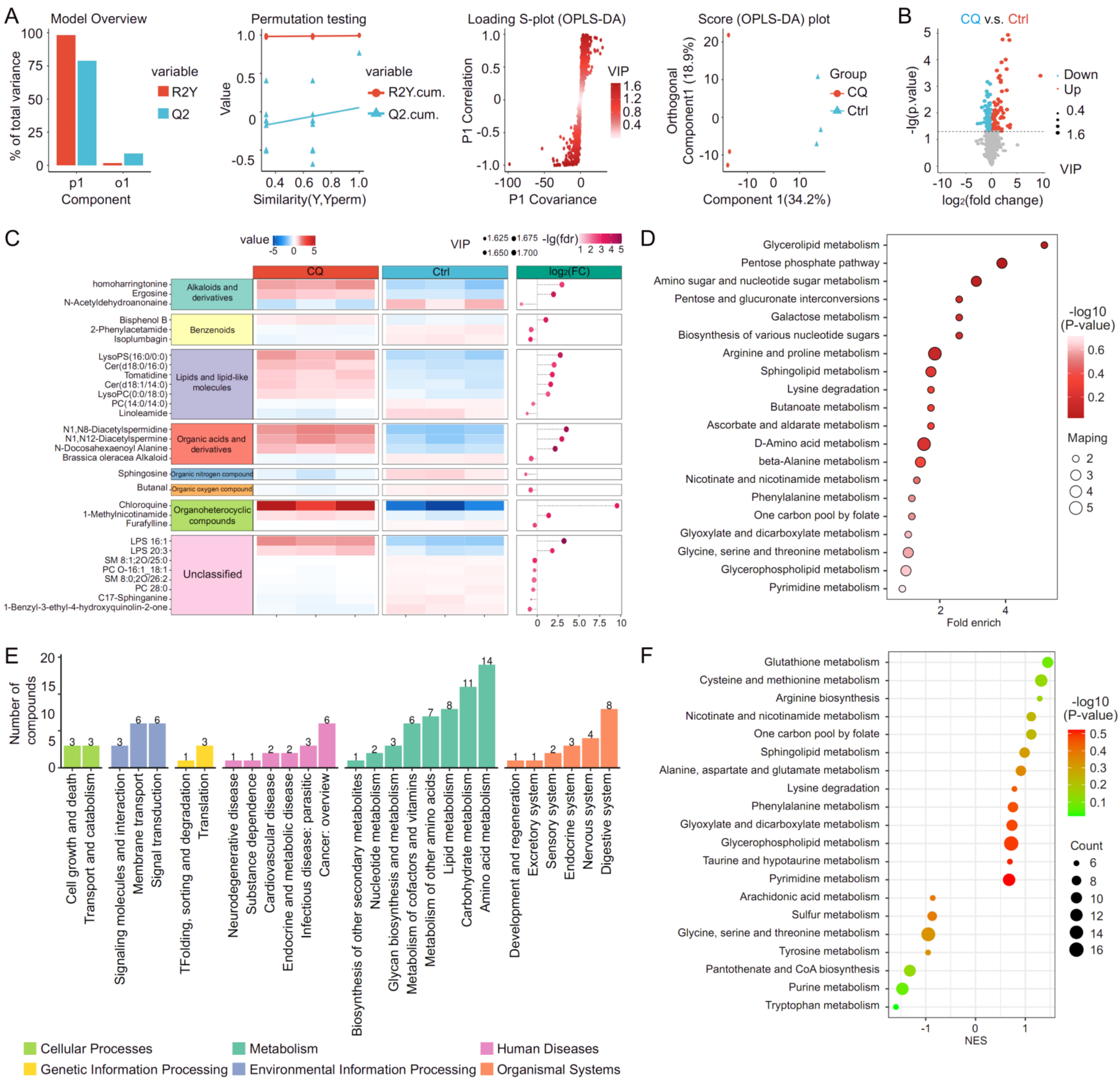

Figure S6. Metabolomics analysis reveals the key position of CQ-induced autophagy suppression in energy and GSH metabolism regulation. Both CQ-treated (40 μM for 16 hours) and untreated MHCC97-H cells were collected for metabolomics analysis. The initial steps included executing the Model Overview and Permutation testing, followed by the Loading S-plot (OPLS-DA) and the Score (OPLS-DA) plot (A). Then, both upregulated and downregulated metabolites (CQ v.s. Ctrl) was presented in (B). Following this, a heatmap was organized to show the altered metabolites (C), and the KEGG enrichment assay (D) was performed in our work. Additionally, the different metabolites were categorized according to various pathways (E), and MSEA enrichment assay was conducted based on the altered metabolites (F).

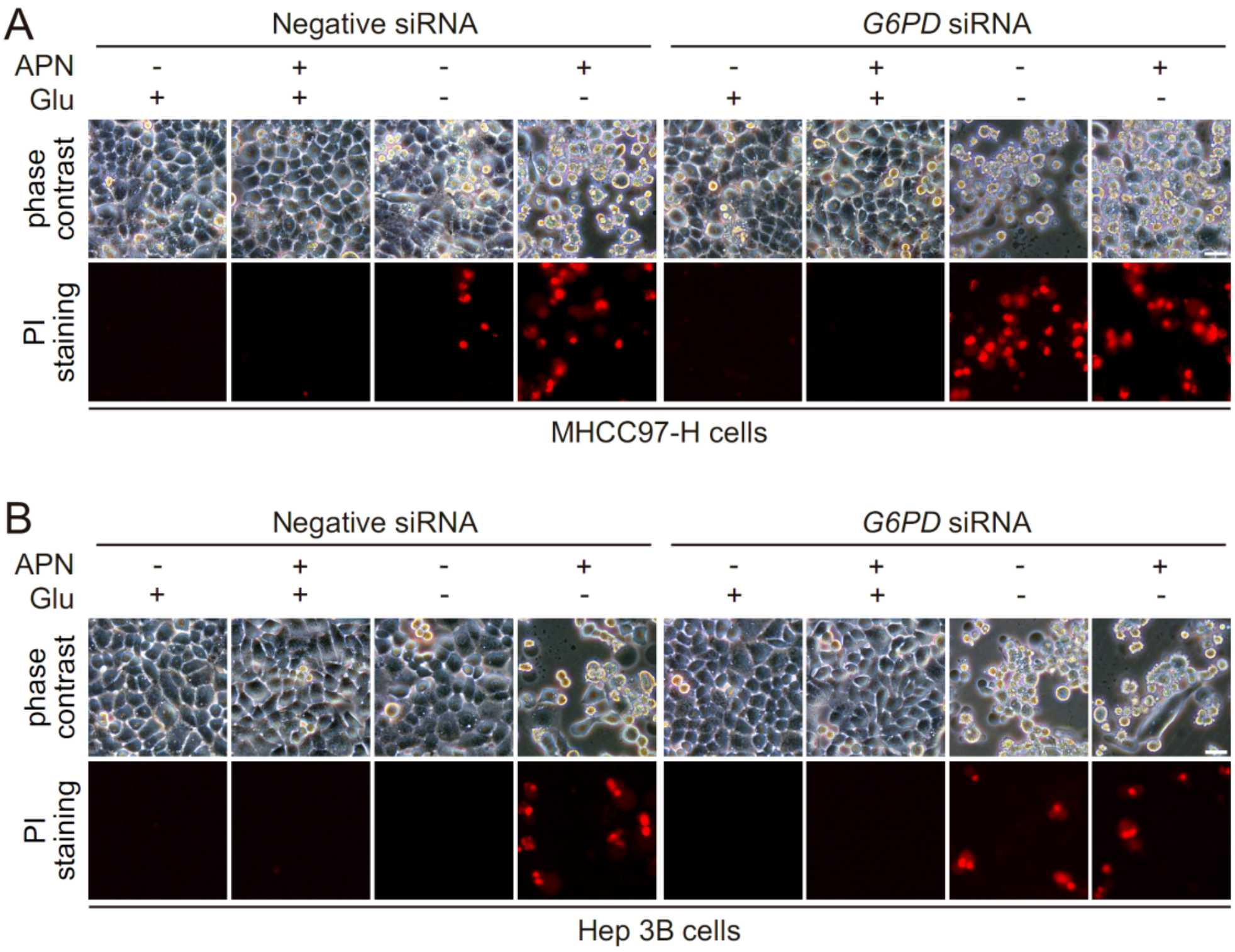

Figure S7. The downregulation of *G6PD* caused by the inhibition of autophagy plays a significant role in disulfidptosis model. The knockdown of *G6PD* was induced via specific siRNA transfection, and the cells transfected with Negative siRNA was used as Ctrl group. Both Ctrl group and knockdown group were treated with APN (1 μM) for 16 hours, followed by glucose starvation (6-8 hours) to induce disulfidptosis. Then, the changes in morphology and cell death were assessed in each group (A-B, Scale bar=20 μm).

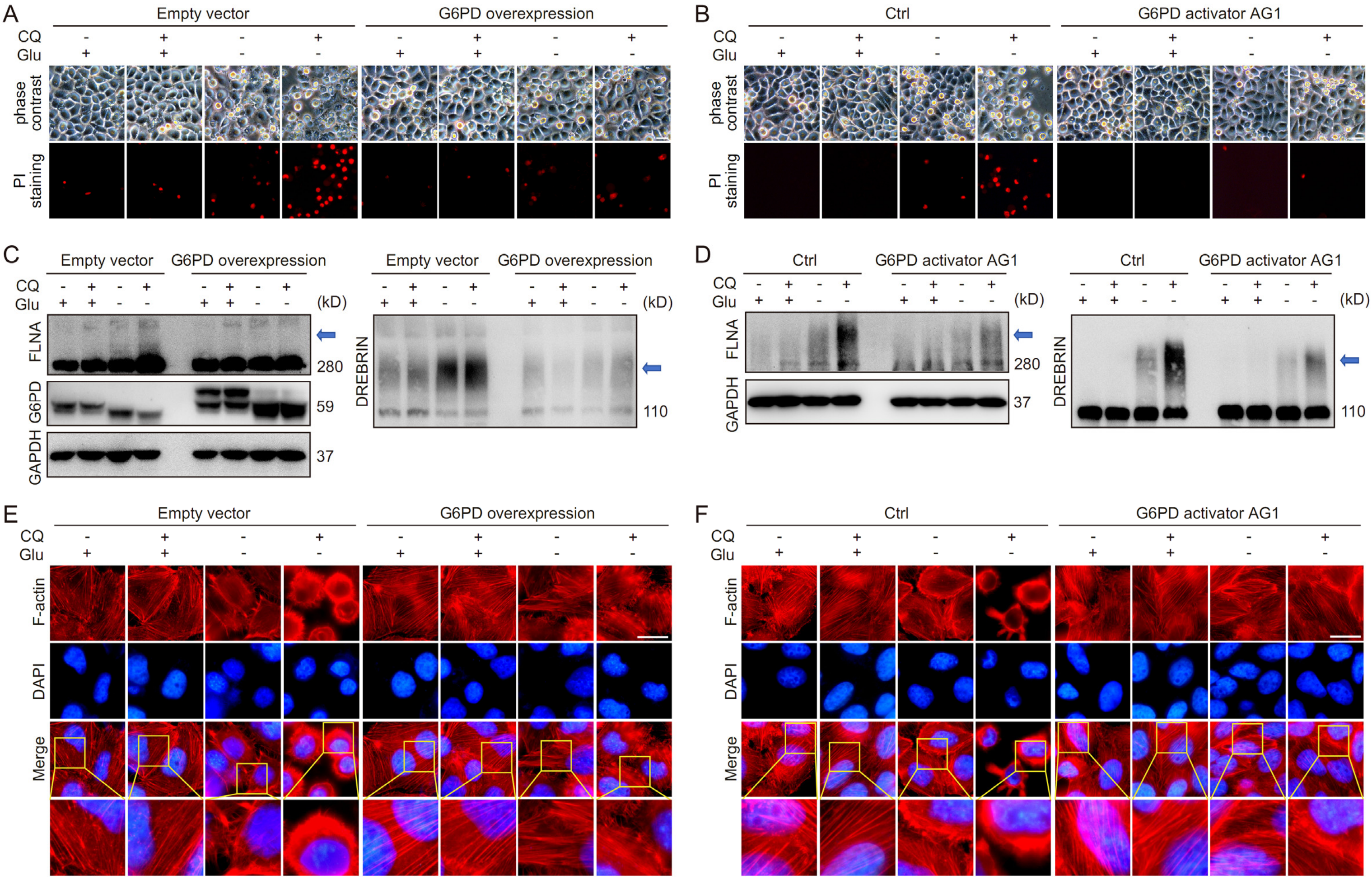

Figure S8. *G6PD* overexpression and G6PD activation effectively mitigated the effects of CQ-induced autphagy suppression in disulfidptosis model. Herein, we facilitated the overexpression of *G6PD* in HCC cells via pCMV-G6PD transfection, while the activity of G6PD was enhanced through treatment with the G6PD activator AG1 (5 μM for 16 h). Disulfidptosis was induced by glucose starvation (6-8 hours). Then, the changes in morphology and cell death were assessed in each group (A-B, Scale bar=20 μm). Meanwhile, the formation of disulfide bonds in cytoskeletal proteins (FLNA and DREBRIN) induced by glucose starvation was analyzed using non-reducing western blot (C-D). Furthermore, phalloidin staining was performed to examine the F-actin across different groups (E-F, Scale bar=10 μm).

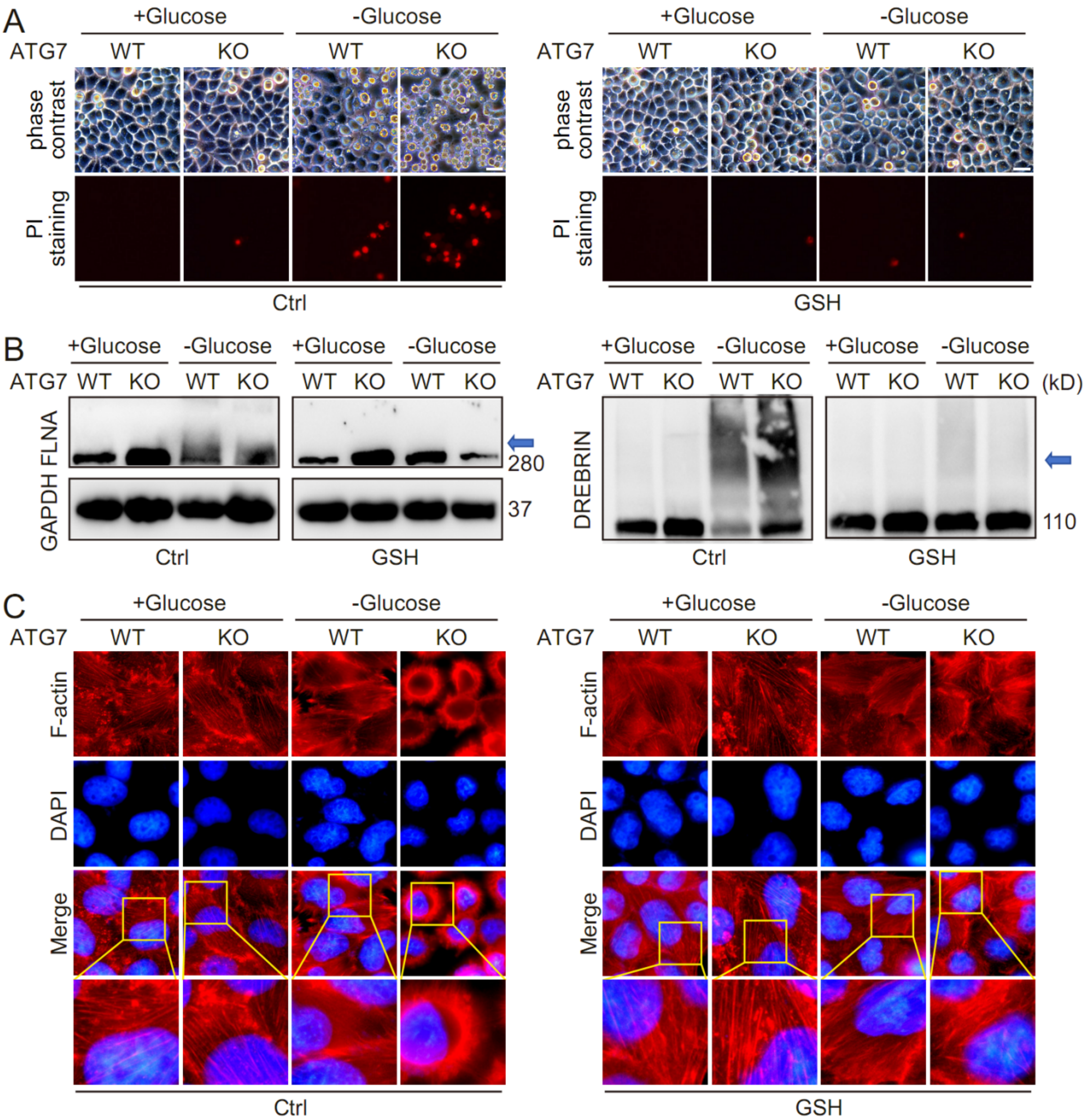

Figure S9. The critical role of GSH in the function of autophagy on disulfidptosis regulation. Both wild type (WT) and *ATG7* knockout (KO) cells were initially treated with GSH (1 mM), then subjected to glucose starvation (6-8 hours) to induce disulfidptosis. Subsequently, the changes in morphology and cell death were assessed in each group (A, Scale bar=20 μm). Meanwhile, the formation of disulfide bonds in cytoskeletal proteins (FLNA and DREBRIN) induced by glucose starvation was analyzed using non-reducing western blot (B). Furthermore, phalloidin staining was performed to examine the F-actin across different groups (C, Scale bar=10 μm).

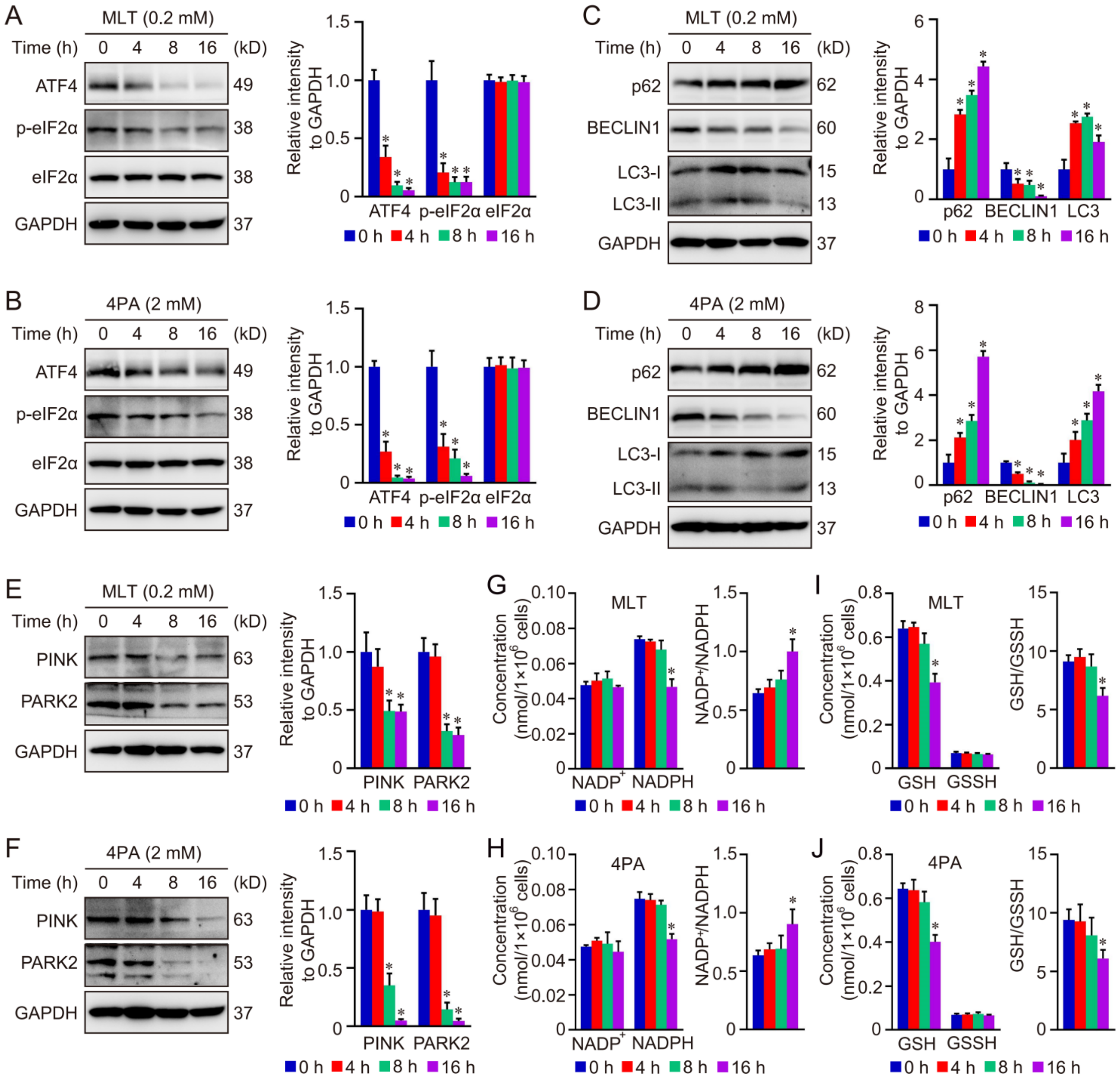

Figure S10. Time-dependent assay of "homeostasis disbalance of ER stress and autophagy-impaired mitophagy-metabolic disruption" axis. To determine whether the biological processes, ER stress-impaired mitophagy-metabolic disruption, operate in parallel or sequentially prior to disulfidptosis, we employed two distinct ER stress inhibitors, MLT and 4PA, in the treatment of MHCC97-H to examine ER stress, mitophagy, and metabolic disruption at various time points (4 h, 8 h, and 16 h). The makers of ER stress were measured using western blot (A-B). Then, cell autophagy was analyzed in different groups (C-D). The protein levels of PINK and PARK2 were tested to evaluate mitophagy (E-F). Finally, $NADP^+$-NADPH metabolism and GSH-GSSH metabolism were assessed to confirm metabolic disruption (G-J). (Data are presented as means ± SD. *: $p < 0.05$ compared to Ctrl group)

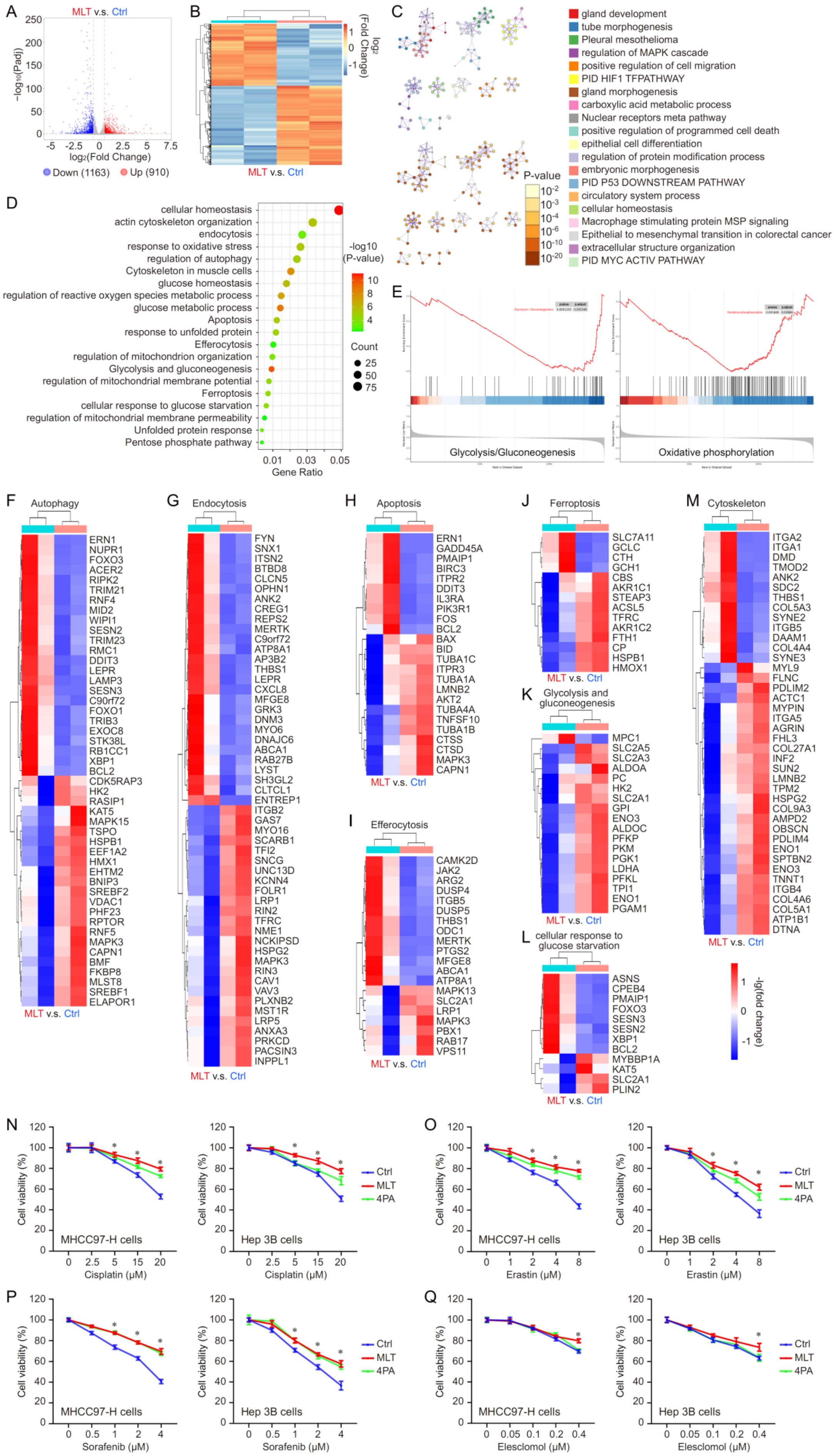
A
MLT v.s. Ctrl
−log10(Padj)
log2(Fold Change)
Down (1163)
Up (910)
B
log2 (Fold Change)
MLT v.s. Ctrl
C
gland development
tube morphogenesis
Pleural mesothelioma
regulation of MAPK cascade
positive regulation of cell migration
PID HIF1 TFPATHWAY
gland morphogenesis
carboxylic acid metabolic process
Nuclear receptors meta pathway
positive regulation of programmed cell death
epithelial cell differentiation
regulation of protein modification process
embryonic morphogenesis
PID P53 DOWNSTREAM PATHWAY
circulatory system process
cellular homeostasis
Macrophage stimulating protein MSP signaling
Epithelial to mesenchymal transition in colorectal cancer
extracellular structure organization
PID MYC ACTIV PATHWAY
P-value
D
cellular homeostasis
actin cytoskeleton organization
endocytosis
response to oxidative stress
regulation of autophagy
Cytoskeleton in muscle cells
glucose homeostasis
regulation of reactive oxygen species metabolic process
glucose metabolic process
Apoptosis
response to unfolded protein
Efferocytosis
regulation of mitochondrion organization
Glycolysis and gluconeogenesis
regulation of mitochondrial membrane potential
Ferroptosis
cellular response to glucose starvation
regulation of mitochondrial membrane permeability
Unfolded protein response
Pentose phosphate pathway
-log10 (P-value)
Count
Gene Ratio
E
Glycolysis/Gluconeogenesis
Oxidative phosphorylation
F
Autophagy
ERN1 NUPR1 FOXO3 ACER2 RIPK2 TRIM21 RNF4 MID2 WIPI1 SESN2 TRIM23 RMC1 DDIT3 LEPR LAMP3 SESN3 C9orf72 FOXO1 TRIB3 EXOC8 STK38L RB1CC1 XBP1 BCL2 CDK5RAP3 HK2 RASIP1 KAT5 MAPK15 TSPO HSPB1 EEF1A2 HMX1 EHTM2 BNIP3 SREBF2 VDAC1 PHF23 RPTOR RNF5 MAPK3 CAPN1 BMF FKBP8 MLST8 SREBF1 ELAPOR1
MLT v.s. Ctrl
G
Endocytosis
FYN SNX1 ITSN2 BTBD8 CLCN5 OPHN1 ANK2 CREG1 REPS2 MERTK C9orf72 ATP8A1 AP3B2 THBS1 LEPR CXCL8 MFGE8 GRK3 DNM3 MYO6 DNAJC6 ABCA1 RAB27B LYST SH3GL2 CLTCL1 ENTREP1 ITGB2 GAS7 MYO16 SCARB1 TFI2 SNCG UNC13D KCNN4 FOLR1 LRP1 RIN2 TFRC NME1 NCKIPSD HSPG2 MAPK3 RIN3 CAV1 VAV3 PLXNB2 MST1R LRP5 ANXA3 PRKCD PACSIN3 INPPL1
MLT v.s. Ctrl
H
Apoptosis
ERN1 GADD45A PMAIP1 BIRC3 ITPR2 DDIT3 IL3RA PIK3R1 FOS BCL2 BAX BID TUBA1C ITPR3 TUBA1A LMNB2 AKT2 TUBA4A TNFSF10 TUBA1B CTSS CTSD MAPK3 CAPN1
MLT v.s. Ctrl
I
Efferocytosis
CAMK2D JAK2 ARG2 DUSP4 ITGB5 DUSP5 THBS1 ODC1 MERTK PTGS2 MFGE8 ABCA1 ATP8A1 MAPK13 SLC2A1 LRP1 MAPK3 PBX1 RAB17 VPS11
MLT v.s. Ctrl
J
Ferroptosis
SLC7A11 GCLC CTH GCH1 CBS AKR1C1 STEAP3 ACSL5 TFRC AKR1C2 FTH1 CP HSPB1 HMOX1
MLT v.s. Ctrl
K
Glycolysis and gluconeogenesis
MPC1 SLC2A5 SLC2A3 ALDOA PC HK2 SLC2A1 GPI ENO3 ALDOC PFKP PKM PGK1 LDHA PFKL TPI1 ENO1 PGAM1
MLT v.s. Ctrl
L
cellular response to glucose starvation
ASNS CPEB4 PMAIP1 FOXO3 SESN3 SESN2 XBP1 BCL2 MYBBP1A KAT5 SLC2A1 PLIN2
MLT v.s. Ctrl
M
Cytoskeleton
ITGA2 ITGA1 DMD TMOD2 ANK2 SDC2 THBS1 COL5A3 SYNE2 ITGB5 DAAM1 COL4A4 SYNE3 MYL9 FLNC PDLIM2 ACTC1 MYPIN ITGA5 AGRIN FHL3 COL27A1 INF2 SUN2 LMNB2 TPM2 HSPG2 COL9A3 AMPD2 OBSCN PDLIM4 ENO1 SPTBN2 ENO3 TNNT1 ITGB4 COL4A6 COL5A1 ATP1B1 DTNA
MLT v.s. Ctrl
-lg(fold change)
N
Cell viability (%)
MHCC97-H cells
Cisplatin (μM)
Hep 3B cells
Cisplatin (μM)
Ctrl
MLT
4PA
O
MHCC97-H cells
Erastin (μM)
Hep 3B cells
Erastin (μM)
P
MHCC97-H cells
Sorafenib (μM)
Hep 3B cells
Sorafenib (μM)
Q
MHCC97-H cells
Elesclomol (μM)
Hep 3B cells
Elesclomol (μM)

Figure S11. Homeostasis between ER stress and autophagy is associated with different types of cell death. To thoroughly examine various cell death pathways, we initially harvested both MLT-treated (0.2 mM for 16 hours) and untreated HCC cells for RNA-seq analysis. A volcano plot was created to assess the impact of autophagy blockage on gene transcription (A). Additionally, both up-regulated and down-regulated genes were organized in a heatmap (B), and utilized for cluster analysis through Metascape. The network of enriched terms for differentially expressed genes was color-coded by cluster ID, and the same terms were further distinguished by p-value (C). Moreover, several enriched terms associated with cell death were presented in (D). Additionally, some differentially expressed genes were further investigated through Gene Set Enrichment Analysis (GSEA) (E). Finally, the differentially expressed genes related with cell death were organized in heatmaps (F-M). The effect of ER stress inhibitors (MLT and 4PA) on Cisplatin-induced apoptosis, Erastin/Sorafenib-induced ferroptosis and Elesclomol-induced Cuproptosis were evaluated using CCK-8 method (N-Q). (Data are presented as means ± SD. *: $p < 0.05$ compared to Ctrl group)

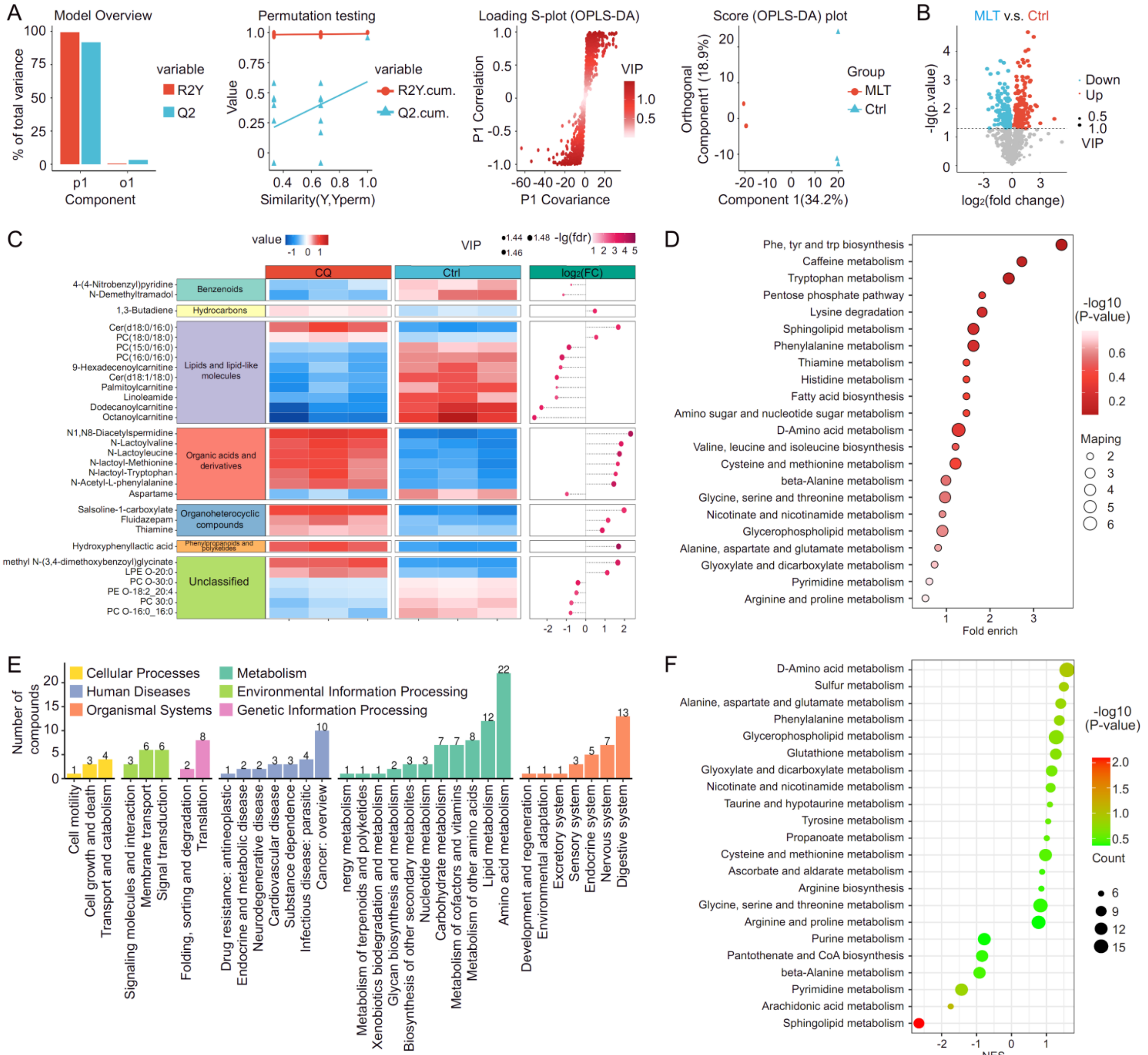

Figure S12. Homeostasis between ER stress and autophagy is associated NADPH metabolism and GSH metabolism regulation. Both MLT-treated (0.2 mM for 16 hours) and untreated HCC cells were collected for metabolomics analysis. The initial steps included executing the Model Overview and Permutation testing, followed by the Loading S-plot (OPLS-DA) and the Score (OPLS-DA) plot (A). Then, both upregulated and downregulated metabolites (MLT v.s. Ctrl) was presented in (B). Following this, a heatmap was organized to show the altered metabolites (C), and the KEGG enrichment assay (D) was performed in our work. Additionally, the different metabolites were categorized according to various pathways (E), and MSEA enrichment assay was conducted based on the altered metabolites (F).

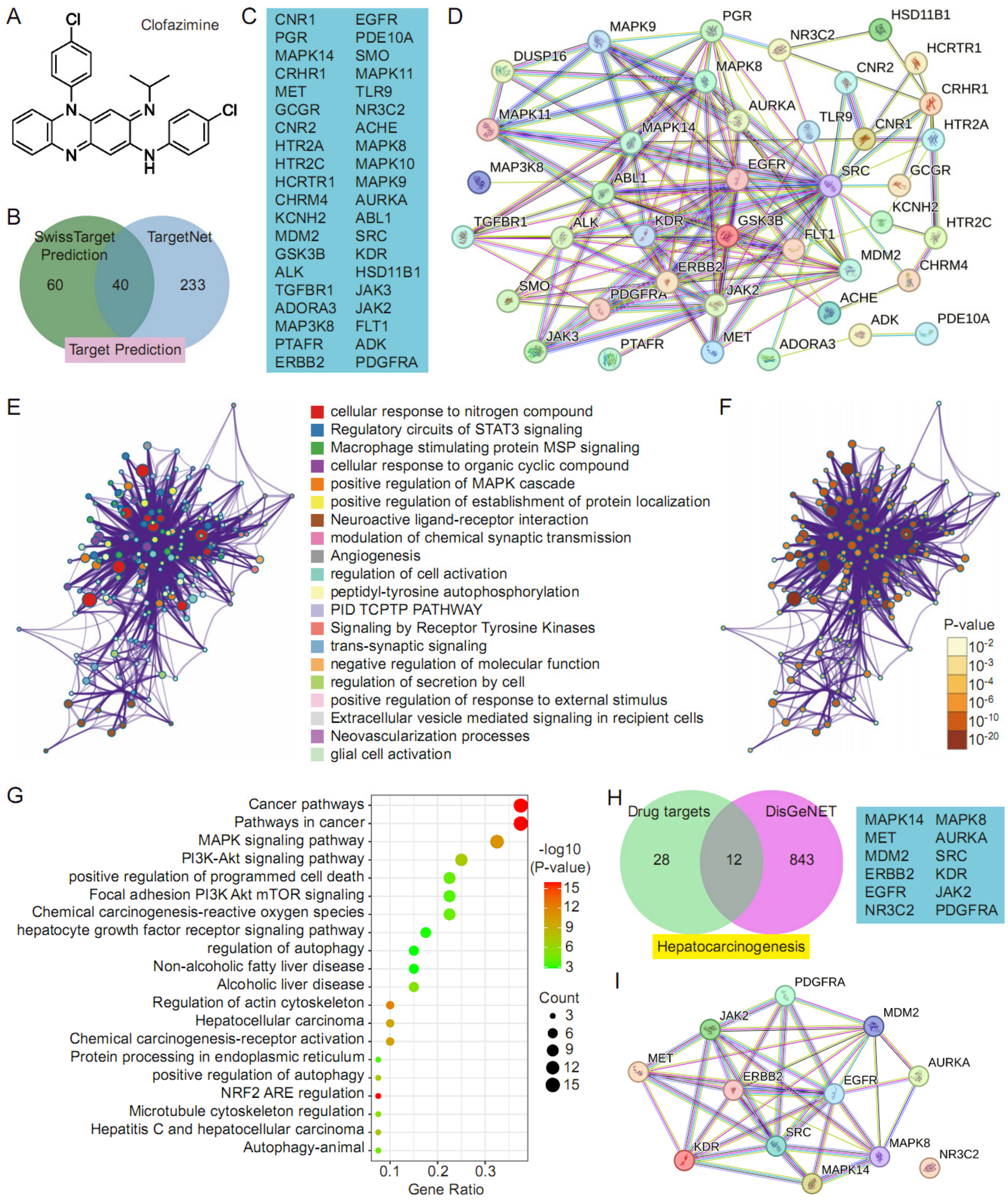

Figure S13. Network pharmacology assay of clofazimine potential bioavailable targets and therapeutic action. The chemical structure of clofazimine (CFZ) was initially presented in (A). The potential bioavailable targets of CFZ were predicted utilizing the SwissTargetPrediction database and the TargetNet database, respectively (B). The overlapping section between the results from the two databases was employed to construct a protein-protein interaction network (C-D). Furthermore, the overlapping genes were utilized for cluster analysis through Metascape, and the top 20 enriched terms from the cluster analysis were compiled into a network plot (E-F). The items associated with autophagy and liver diseases were summarized in (G). Finally, the potential bioavailable targets of CFZ related to hepatocarcinogenesis were organized in (H-I).

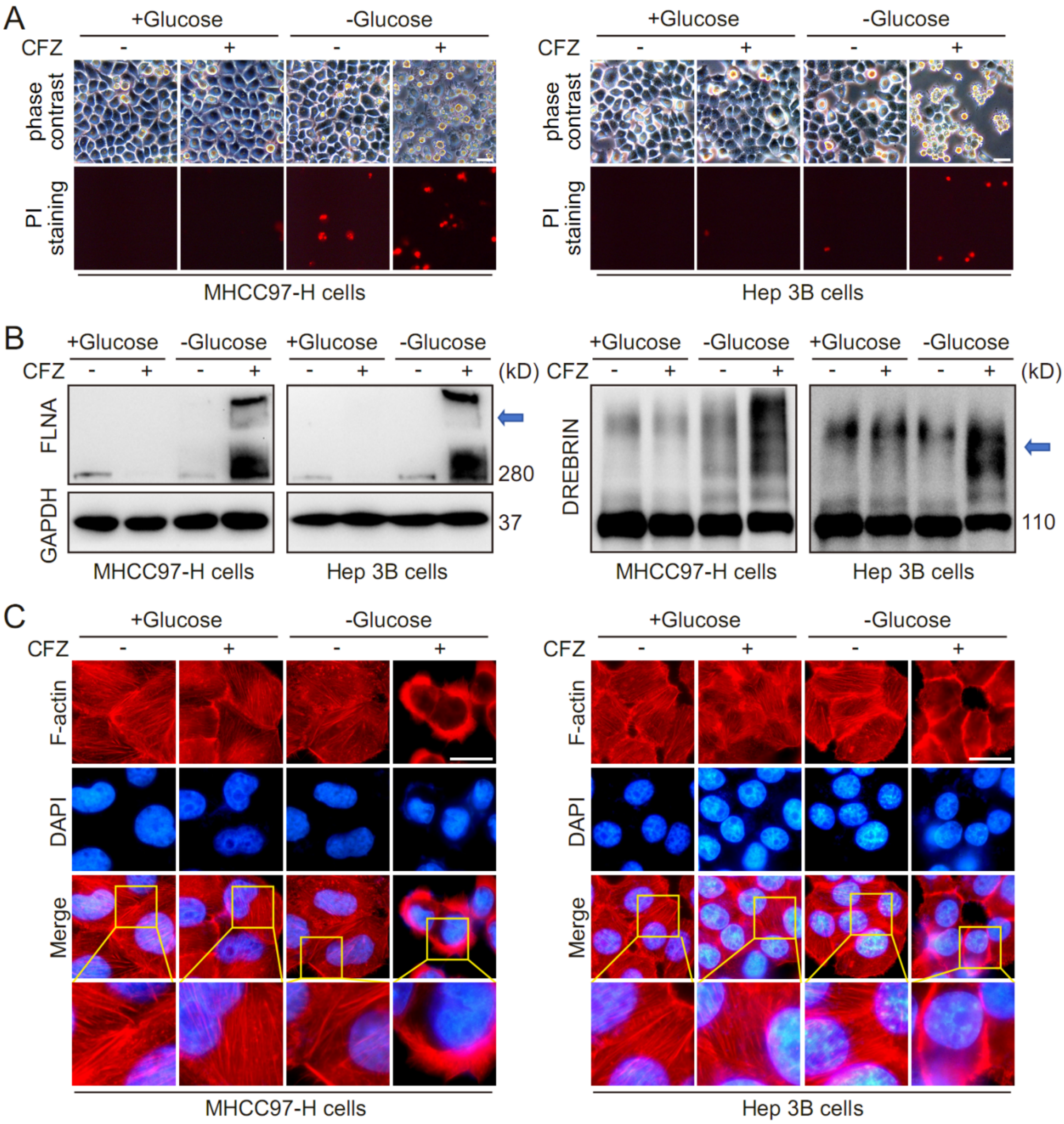

Figure S14. Clofazimine enhances the sensitivity of HCC cells to disulfidptosis. Herein, Both MHCC97-H and Hep 3B cells were treated with clofazimine (CFZ, 30 μM) for 16 hours, followed by glucose starvation (6-8 hours) to induce disulfidptosis. Subsequently, the alterations in morphology and cell death were assessed in each group (A, Scale bar=20 μm). Furthermore, the formation of disulfide bonds in cytoskeletal proteins (FLNA and DREBRIN) induced by glucose starvation was analyzed using non-reducing western blot (B). Lastly, phalloidin staining was utilized to examine the F-actin structure across various groups (C, Scale bar=10 μm).

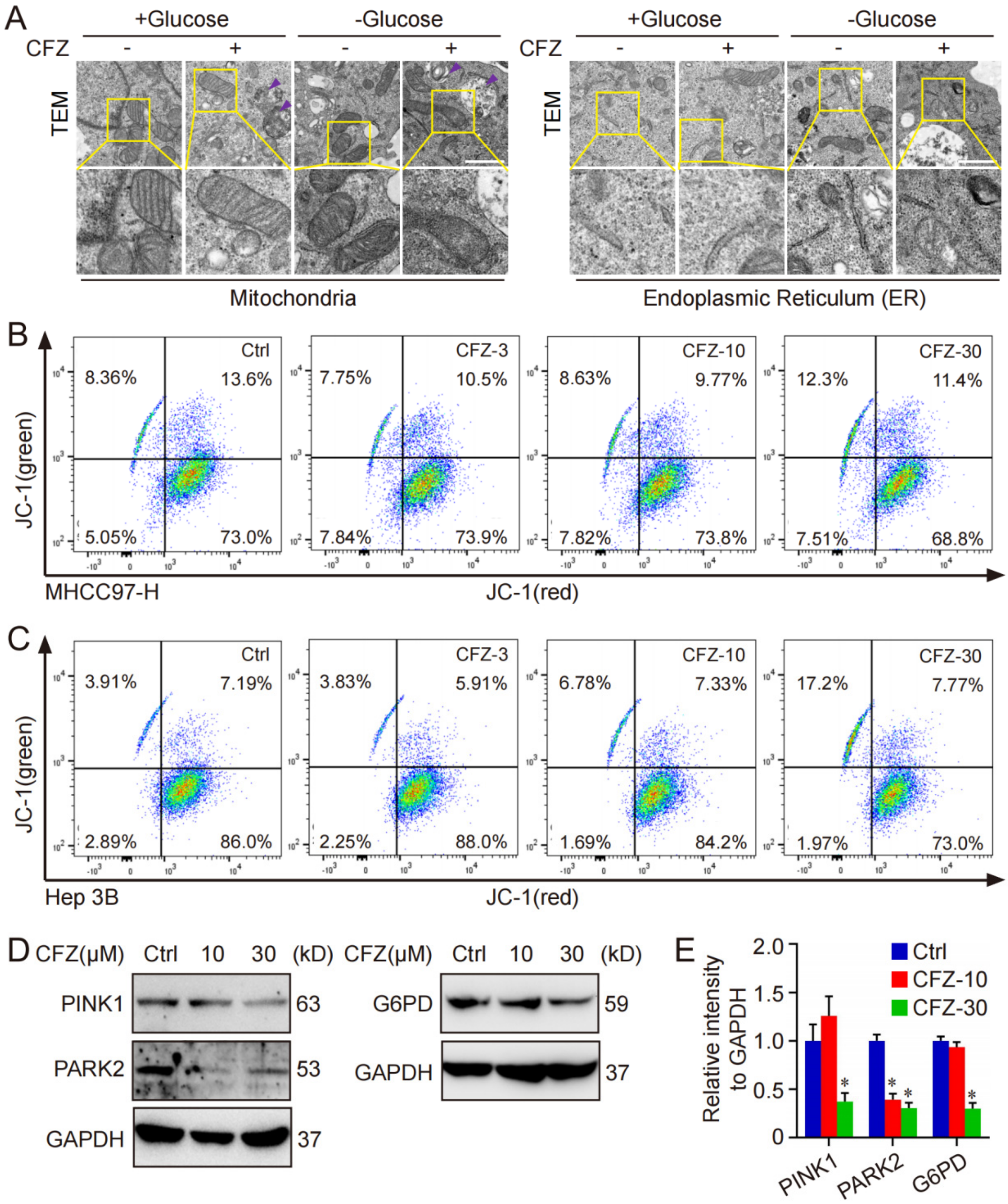

Figure S15. The effect of clofazimine on mitochondria function. Firstly, MHCC97-H cells were treated with clofazimine (CFZ, 30 μM) for 16 hours, followed by glucose starvation (6-8 hours) to induce disulfidptosis. Then, the cells from different groups were harvested for transmission electron microscopy (TEM) analysis (A, Scale bar=1 μm, arrowhead: autophagosome). In addition, the effect of CFZ on mitochondrial membrane potential (MMP) was analyzed via JC-1 staining (B-C), and the protein levels of mitophagy makers (PINK1 and PARK2) and G6PD were evaluated by western blot assay (D). The quantitative results of western blot were shown in (E). (Data are presented as means ± SD. *: $p < 0.05$ compared to Ctrl group)

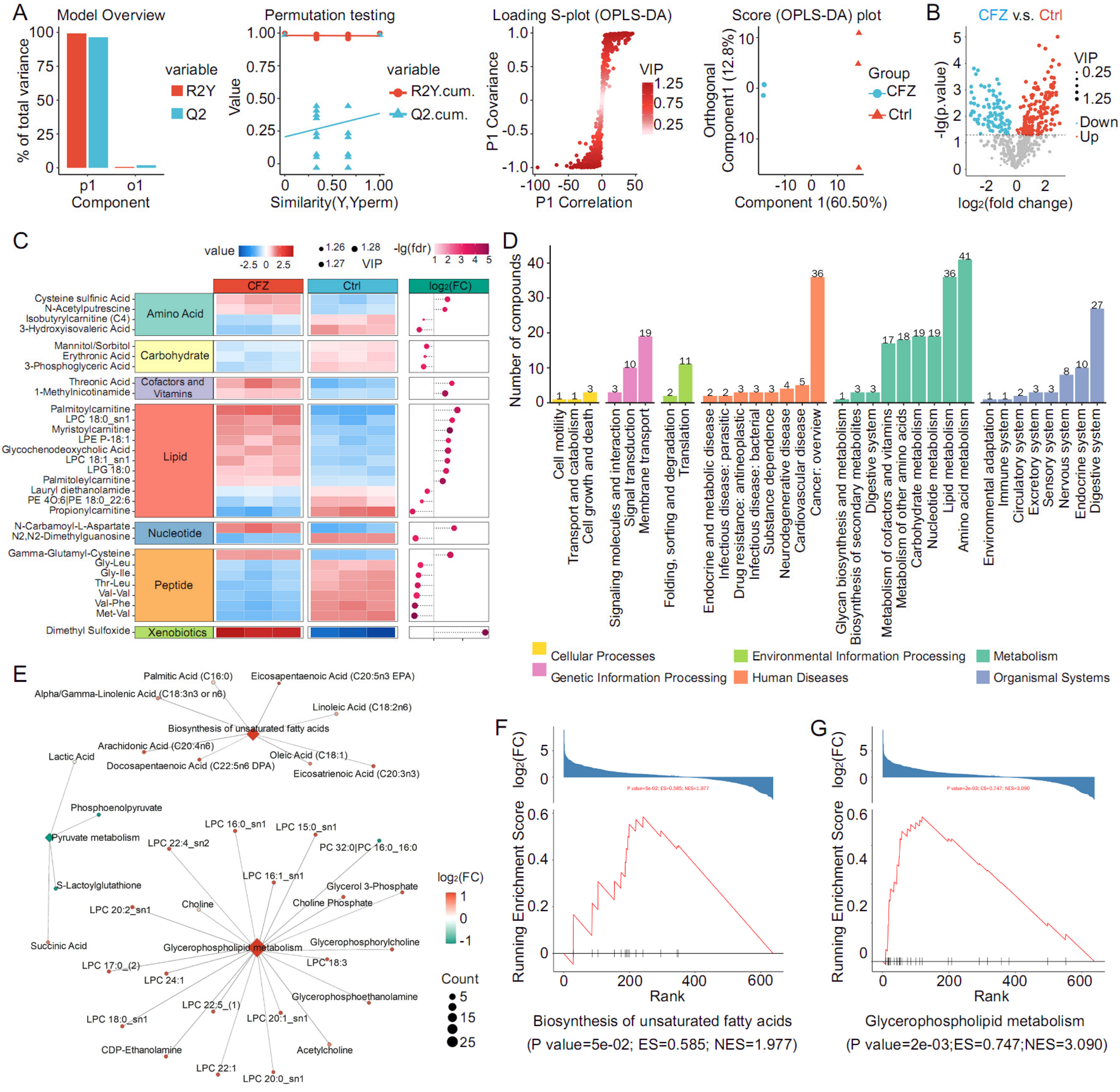

Figure S16. The effect of clofazimine on different metabolites. In this study, the HCC cell line MHCC97-H was subjected to treatment with clofazimine (CFZ, 30 μM) for a duration of 16 hours. Subsequently, the cell samples were collected for metabolomics analysis. The initial steps included executing the Model Overview and Permutation testing, followed by the Loading S-plot (OPLS-DA) and the Score (OPLS-DA) plot (A). A summary of both upregulated and downregulated metabolites was presented in (B). Following this, cluster analysis was conducted based on the altered metabolites. The enriched terms are displayed in (C-D), and the interactions among differential metabolites are summarized in (E). Finally, the significant metabolic pathways in HCC cells affected by CFZ were assessed through Gene Set Enrichment Analysis (F-G).

Table S1. The metabolites in metabolomics analysis (CQ v.s. Ctrl)

Alignment ID	Metabolite name	HMDB_SuperClass	HMDB_Class	HMDB_SubClass	HMDB ID	cpd_id	log2fc	p.value	fdr	VIP	regulation
Meta_1007	PC(16:0/20:4(8Z,11Z,14Z,17Z))	Lipids and lipid-like molecules	Glycerophospholipids	Glycerophosphocholines	HMDB0007983		-0.360666667	0.0251983891334243	0.0251983891334243	1.59178145634715	down
Meta_1008	PC 16:1_20:3	Unclassified	Unclassified	Unclassified			-0.358666667	0.0464153736078815	0.0464153736078815	1.41977672459167	down
Meta_103	2-Aminoadipic acid	Organic acids and derivatives	Carboxylic acids and derivatives	Amino acids, peptides, and analogues	HMDB0302754	C00956	-0.685333333	0.0111483836628836	0.0111483836628836	1.65957120814973	down
Meta_135	1-Phenyl-1-pentanone	Organic oxygen compounds	Organooxygen compounds	Carbonyl compounds	HMDB0031208		-0.524	0.0433321454038041	0.0433321454038041	1.47101827838219	down
Meta_139	Phosphohydroxypyruvic acid	Organic oxygen compounds	Organooxygen compounds	Carbonyl compounds	HMDB0001024	C03232	-0.166333333	0.00745787439340298	0.00745787439340298	1.59121908635995	down
Meta_144	Dichloromethane	Organohalogen compounds	Alkyl halides	Halomethanes	HMDB0031548	C02271	-0.499	0.0441103502062987	0.0441103502062987	1.45144472353704	down
Meta_146	p-Hydroxymandelic acid	Benzenoids	Phenols	1-hydroxy-2-unsubstituted benzenoids	HMDB0000822	C11527	-0.705333333	0.0388805982990979	0.0388805982990979	1.51128286622736	down
Meta_161	2-Oxoarginine	Organic acids and derivatives	Keto acids and derivatives	Short-chain keto acids and derivatives	HMDB0004225	C03771	-0.263333333	0.0278316190456881	0.0278316190456881	1.54532394338168	down
Meta_174	Glycerol pyruvate	Lipids and lipid-like molecules	Glycerolipids	Monoradylglycerols	HMDB0252845		-0.944333333	0.0315883234944317	0.0315883234944317	1.5026725069663	down
Meta_180	Phosphorylcholine	Organic nitrogen compounds	Organonitrogen compounds	Quaternary ammonium salts	HMDB0001565	C00588	-0.916	0.0469854288793757	0.0469854288793757	1.42483743483099	down
Meta_186	Isoplumbagin	Benzenoids	Naphthalenes	Naphthoquinones	HMDB0035291		-0.808666667	0.000673937546933566	0.000673937546933566	1.67041375078731	down
Meta_198	Glycyl-Isoleucine	Organic acids and derivatives	Carboxylic acids and derivatives	Amino acids, peptides, and analogues	HMDB0028844		-0.619333333	0.0142425613990006	0.0142425613990006	1.56734269931959	down
Meta_207	Gluconic acid	Organic oxygen compounds	Organooxygen compounds	Carbohydrates and carbohydrate conjugates	HMDB0000625	C00257	-0.864666667	0.0443579403337183	0.0443579403337183	1.44357152924699	down
Meta_224	Indolelactic acid	Organoheterocyclic compounds	Indoles and derivatives	Indolyl carboxylic acids and derivatives	HMDB0000671	C02043	-0.191333333	0.0432579881571418	0.0432579881571418	1.45274252252538	down
Meta_227	N-(3-Methylbut-2-EN-1-YL)-9H-purin-6-amine	Organoheterocyclic compounds	Imidazopyrimidines	Purines and purine derivatives	HMDB0245646	C04083	-0.554333333	0.0426190240971756	0.0426190240971756	1.40029884448815	down
Meta_246	4-(4-Nitrobenzyl)pyridine	Benzenoids	Benzene and substituted derivatives	Nitrobenzenes	HMDB0246294		-0.367333333	0.0144248226187769	0.0144248226187769	1.55703835791085	down
Meta_264	Serylleucine	Organic acids and derivatives	Carboxylic acids and derivatives	Amino acids, peptides, and analogues	HMDB0029043		-0.180333333	0.014228143893957	0.014228143893957	1.5421175729897	down
Meta_275	Glycyl-Phenylalanine	Organic acids and derivatives	Carboxylic acids and derivatives	Amino acids, peptides, and analogues	HMDB0028848		-0.604666667	0.0255726554845187	0.0255726554845187	1.48450458524182	down
Meta_314	alpha-D-Glucose	Organic oxygen compounds	Organooxygen compounds	Carbohydrates and carbohydrate conjugates	HMDB0003345	C00267	-0.320666667	0.0416914508480644	0.0416914508480644	1.48052784979385	down
Meta_35	gamma-Aminobutyric acid	Organic acids and derivatives	Carboxylic acids and derivatives	Amino acids, peptides, and analogues	HMDB0000112	C00334	-0.493	0.018423170442047	0.018423170442047	1.56939289994963	down
Meta_351	3-(6,7-Dimethoxy-1,2,3,4-tetrahydroisoquinolin-1-yl)propan-1-ol	Unclassified	Unclassified	Unclassified			-0.983	0.0474480533702355	0.0474480533702355	1.38389130320526	down
Meta_36	Choline	Organic nitrogen compounds	Organonitrogen compounds	Quaternary ammonium salts	HMDB0000097	C00114	-0.810333333	0.0431664353462713	0.0431664353462713	1.42663598444935	down
Meta_37	Glyceric acid	Organic oxygen compounds	Organooxygen compounds	Carbohydrates and carbohydrate conjugates	HMDB0000139	C00258	-0.983333333	0.0314800782619087	0.0314800782619087	1.56095659608008	down
Meta_373	Furafylline	Organoheterocyclic compounds	Imidazopyrimidines	Purines and purine derivatives	HMDB0252528		-0.300333333	0.00201622012785229	0.00201622012785229	1.65880853568537	down
Meta_391	3'-HPPH	Organoheterocyclic compounds	Azolidines	Imidazolidines	HMDB0013896		-0.234	0.0394882977483187	0.0394882977483187	1.41394061646564	down
Meta_393	N-(4-Aminobenzoyl)-L-glutamic acid	Organic acids and derivatives	Carboxylic acids and derivatives	Amino acids, peptides, and analogues	HMDB0255007		-0.647	0.0358044305483818	0.0358044305483818	1.43861120112635	down
Meta_423	Pantetheine	Organic acids and derivatives	Carboxylic acids and derivatives	Amino acids, peptides, and analogues	HMDB0003426	C00831	-1.005666667	0.0100424607634155	0.0100424607634155	1.60006466456758	down
Meta_427	1-Benzyl-3-ethyl-4-hydroxyquinolin-2-one	Unclassified	Unclassified	Unclassified			-0.898	0.00217959767473861	0.00217959767473861	1.66167672433884	down
Meta_434	Asparaginyl-Phenylalanine	Organic acids and derivatives	Carboxylic acids and derivatives	Amino acids, peptides, and analogues	HMDB0028738		-0.891666667	0.0126137661512325	0.0126137661512325	1.62957286403726	down
Meta_435	Linoleamide	Lipids and lipid-like molecules	Fatty Acyls	Fatty amides	HMDB0062656		-1.214666667	0.00540002435926478	0.00540002435926478	1.62201009340783	down
Meta_437	2'-O-Methylinosine	Unclassified	Unclassified	Unclassified			-0.394666667	0.0328610672119135	0.0328610672119135	1.57780418623176	down
Meta_445	Sphingosine	Organic nitrogen compounds	Organonitrogen compounds	Amines	HMDB0000252	C00319	-1.376	0.00353021005625612	0.00353021005625612	1.62320626233963	down
Meta_454	C17-Sphinganine	Unclassified	Unclassified	Unclassified			-0.734333333	0.00685906231001398	0.00685906231001398	1.60753449787246	down
Meta_461	Brassica oleracea Alkaloid	Organic acids and derivatives	Carboxylic acids and derivatives	Amino acids, peptides, and analogues	HMDB0033602		-0.734666667	0.00211145444434437	0.00211145444434437	1.68688813032993	down
Meta_463	Aspartame	Organic acids and derivatives	Carboxylic acids and derivatives	Amino acids, peptides, and analogues	HMDB0001894	C11045	-0.680666667	0.0078143599494659	0.0078143599494659	1.67401449944075	down
Meta_472	3'-O-Methylguanosine	Nucleosides, nucleotides, and analogues	Purine nucleosides	Unclassified	HMDB0006038		-0.597	0.00859190182176119	0.00859190182176119	1.60223205489161	down
Meta_473	trans-4-Nonenal	Organic oxygen compounds	Organooxygen compounds	Carbonyl compounds	HMDB0032535		-1.280666667	0.00809741169669759	0.00809741169669759	1.58744825408636	down
Meta_479	Oxadixyl	Unclassified	Unclassified	Unclassified		C18753	-2.727333333	0.0251860192812619	0.0251860192812619	1.55812955402732	down
Meta_481	""""\4-hydroxy-5,7-dimethyl-3-benzylhydroquinolin-2-one CollisionEnergy:102040\""""""""""""""""""""""	Unclassified	Unclassified	Unclassified			-0.954333333	0.0015286820773852	0.0015286820773852	1.67626986455994	down
Meta_486	N-Acetyldehydroanonaine	Alkaloids and derivatives	Aporphines	Unclassified	HMDB0041537		-1.910333333	0.00617732665988353	0.00617732665988353	1.62187107688868	down
Meta_49	Butanal	Organic oxygen compounds	Organooxygen compounds	Carbonyl compounds	HMDB0003543	C01412	-0.791	0.00196823887716981	0.00196823887716981	1.67410863248253	down
Meta_508	Dehydrophytosphingosine	Organic nitrogen compounds	Organonitrogen compounds	Amines	HMDB0038057		-2.130666667	0.0244357185859599	0.0244357185859599	1.60915113909374	down
Meta_577	MG(0:0/18:2(9Z,12Z)/0:0)	Lipids and lipid-like molecules	Fatty Acyls	Lineolic acids and derivatives	HMDB0011538		-0.517666667	0.0293355952025483	0.0293355952025483	1.53323555485534	down
Meta_656	N6-Carbamoyl-L-threonyladenosine	Unclassified	Unclassified	Unclassified			-1.276	0.0159531080461722	0.0159531080461722	1.62139340766645	down
Meta_67	Thymine	Organoheterocyclic compounds	Diazines	Pyrimidines and pyrimidine derivatives	HMDB0000262	C00178	-0.656666667	0.0496838472356928	0.0496838472356928	1.5049227484461	down
Meta_701	N-[2-(3,4-dihydroxyphenyl)ethyl]docosa-4,7,10,13,16,19-hexaenamide	Unclassified	Unclassified	Unclassified			-0.623333333	0.0463482948574371	0.0463482948574371	1.40081716624149	down
Meta_709	LPC O-15:0	Unclassified	Unclassified	Unclassified			-0.982666667	0.0233070616103239	0.0233070616103239	1.49247692274884	down
Meta_71	5,6-dihydrothymine	Organoheterocyclic compounds	Diazines	Pyrimidines and pyrimidine derivatives	HMDB0304210	C00906	-0.900333333	0.0175486871179477	0.0175486871179477	1.54927935499271	down
Meta_731	3b,18b-3-Methoxy-11-oxo-12-oleanen-30-oic acid	Lipids and lipid-like molecules	Prenol lipids	Triterpenoids	HMDB0034516		-0.877333333	0.0257895349823773	0.0257895349823773	1.46667928566718	down
Meta_748	Frangulanine	Organic acids and derivatives	Carboxylic acids and derivatives	Amino acids, peptides, and analogues	HMDB0030199	C10003	-0.256	0.0189981765439709	0.0189981765439709	1.51935889997268	down
Meta_765	myo-Inositol 1,3,4,5-tetrakisphosphate	Organic oxygen compounds	Organooxygen compounds	Alcohols and polyols	HMDB0001059	C01272	-0.535333333	0.0213022407693699	0.0213022407693699	1.53566794873913	down
Meta_846	PC(14:0/14:0)	Lipids and lipid-like molecules	Glycerophospholipids	Glycerophosphocholines	HMDB0007866		-0.490333333	0.00518524766249509	0.00518524766249509	1.64155537976086	down
Meta_859	PC 28:0	Unclassified	Unclassified	Unclassified			-0.488666667	0.0043219943940076	0.0043219943940076	1.64720953315612	down
Meta_860	PE O-16:1_18:1	Unclassified	Unclassified	Unclassified			-0.541333333	0.0427622213057728	0.0427622213057728	1.54756732602493	down
Meta_874	LNAPE 16:1/N-18:0	Unclassified	Unclassified	Unclassified			-0.855666667	0.0229368525557741	0.0229368525557741	1.5048895568729	down
Meta_875	PE(16:0/18:1(9Z))	Lipids and lipid-like molecules	Glycerophospholipids	Glycerophosphoethanolamines	HMDB0008927	C13877	-0.408	0.0498967133352933	0.0498967133352933	1.48058824723133	down
Meta_884	PE O-16:1_20:4	Unclassified	Unclassified	Unclassified			-0.333333333	0.0100488867825648	0.0100488867825648	1.61620653752973	down
Meta_885	PE(20:4(5Z,8Z,11Z,14Z)/P-16:0)	Lipids and lipid-like molecules	Glycerophospholipids	Glycerophosphoethanolamines	HMDB0009411		-0.795666667	0.0116646715792864	0.0116646715792864	1.59682462564861	down
Meta_886	PE O-16:1_20:3	Unclassified	Unclassified	Unclassified			-0.501333333	0.0462595310493026	0.0462595310493026	1.54429823271657	down
Meta_898	SM 8:1;2O/25:0	Unclassified	Unclassified	Unclassified			-0.298666667	0.000805598110039355	0.000805598110039355	1.68161936130272	down
Meta_918	SM 8:0;2O/26:2	Unclassified	Unclassified	Unclassified			-0.381333333	0.00501572620172025	0.00501572620172025	1.68856231989261	down
Meta_927	PE O-18:2_20:4	Unclassified	Unclassified	Unclassified			-0.445666667	0.0403435535668412	0.0403435535668412	1.56384889302766	down
Meta_94	2-Phenylacetamide	Benzenoids	Benzene and substituted derivatives	Phenylacetamides	HMDB0010715	C02505	-0.737666667	0.00245201845207751	0.00245201845207751	1.6829168759864	down
Meta_951	PC(14:1(9Z)/P-16:0)	Lipids and lipid-like molecules	Glycerophospholipids	Glycerophosphocholines	HMDB0007928		-1.447	0.0221630227225323	0.0221630227225323	1.53136145912944	down
Meta_973	PC O-16:1_18:1	Unclassified	Unclassified	Unclassified			-0.322666667	0.00419009009919245	0.00419009009919245	1.63465335688175	down
Meta_1	Acetaldehyde oxime	Organic nitrogen compounds	Organonitrogen compounds	Oximes	HMDB0003656	C02658	5.07166666666667	0.158626966088525	0.158626966088525	1.27344106308901	unchanged
Meta_10	Glyoxylic acid	Organic acids and derivatives	Carboxylic acids and derivatives	Carboxylic acids	HMDB0000119	C00048	0.226333333333335	0.106472548063584	0.106472548063584	1.28299449509769	unchanged
Meta_100	Chloroform	Organohalogen compounds	Alkyl halides	Halomethanes	HMDB0029596	C13827	-0.183666667	0.24207863405002	0.24207863405002	1.04358971937311	unchanged

Meta_1000	PC 17:0_18:1	Unclassified	Unclassified	Unclassified			0.302666666666667	0.459726819725588	0.459726819725588	0.682312661720513	unchanged
Meta_1001	PC O-40:7	Unclassified	Unclassified	Unclassified			-0.176333333	0.353716222107753	0.353716222107753	0.825313494578937	unchanged
Meta_1002	PC O-40:6	Unclassified	Unclassified	Unclassified			0.0250000000000004	0.920789750520265	0.920789750520265	0.0494401712803834	unchanged
Meta_1003	PC(20:1(11Z)/P-18:0)	Lipids and lipid-like molecules	Glycerophospholipids	Glycerophosphocholines	HMDB0008325		0.189666666666668	0.699828293575721	0.699828293575721	0.327721863201033	unchanged
Meta_1004	[5-Hydroxy-6-[2-(3-hydroxy-4-methoxyphenyl)ethoxy]-2-[[3,4,5-trihydroxy-6-(hydroxymethyl)oxan-2-yl]oxymethyl]-4-(3,4,5-trihydroxy-6-methyloxan-2-yl)oxyoxan-3-yl] 3-(3,4-dihydroxyphenyl)prop-2-enoate	Unclassified	Unclassified	Unclassified			0.586666666666666	0.23208847514007	0.23208847514007	1.08561893040889	unchanged
Meta_1005	PC(16:0/20:5(5Z,8Z,11Z,14Z,17Z))	Lipids and lipid-like molecules	Glycerophospholipids	Glycerophosphocholines	HMDB0007984		0.161666666666667	0.357262608424176	0.357262608424176	0.785543074238002	unchanged
Meta_1006	PC(16:1(9Z)/20:4(8Z,11Z,14Z,17Z))	Lipids and lipid-like molecules	Glycerophospholipids	Glycerophosphocholines	HMDB0008016		-0.497666667	0.110553454303238	0.110553454303238	1.33779569816453	unchanged
Meta_1009	PC 38:6	Unclassified	Unclassified	Unclassified			-0.176666667	0.464898661885456	0.464898661885456	0.68155207821293	unchanged
Meta_101	2-Acetyl-4-methylthiazole	Organic oxygen compounds	Organooxygen compounds	Carbonyl compounds	HMDB0302368		-0.731333333	0.202615848792499	0.202615848792499	1.13325647633791	unchanged
Meta_1010	PC(18:1(9Z)/18:2(9Z,12Z))	Lipids and lipid-like molecules	Glycerophospholipids	Glycerophosphocholines	HMDB0008105		-0.178666667	0.675432705426685	0.675432705426685	0.373722873997851	unchanged
Meta_1011	PC 16:0_20:3	Unclassified	Unclassified	Unclassified			-0.343	0.210075128590071	0.210075128590071	1.0916383467452	unchanged
Meta_1012	PC 38:5	Unclassified	Unclassified	Unclassified			0.0813333333333333	0.587430888402324	0.587430888402324	0.48821697635928	unchanged
Meta_1013	PC(20:4(5Z,8Z,11Z,14Z)/20:4(5Z,8Z,11Z,14Z))	Lipids and lipid-like molecules	Glycerophospholipids	Glycerophosphocholines	HMDB0008443		0.204666666666668	0.832650702768245	0.832650702768245	0.210697967506797	unchanged
Meta_1015	SM 8:0;2O/32:1	Unclassified	Unclassified	Unclassified			-1.081333333	0.103781715623434	0.103781715623434	1.23113667054194	unchanged
Meta_1016	PC(18:0/18:1(9Z))	Lipids and lipid-like molecules	Glycerophospholipids	Glycerophosphocholines	HMDB0008038		0.0723333333333329	0.580876612885275	0.580876612885275	0.502476466913616	unchanged
Meta_1017	PC O-16:1_22:6	Unclassified	Unclassified	Unclassified			-0.037666667	0.925712834655874	0.925712834655874	0.125364168162172	unchanged
Meta_1018	PC(18:0/18:0)	Lipids and lipid-like molecules	Glycerophospholipids	Glycerophosphocholines	HMDB0008036		0.222666666666667	0.29877353412505	0.29877353412505	0.940758409158242	unchanged
Meta_1019	PC O-18:2_20:4	Unclassified	Unclassified	Unclassified			0.223000000000001	0.523950814117337	0.523950814117337	0.562150641459741	unchanged
Meta_102	5-(2-Hydroxyethyl)-4-methylthiazole	Organoheterocyclic compounds	Azoles	Thiazoles	HMDB0032985	C04294	2.24233333333333	0.147901064433803	0.147901064433803	1.27012966225941	unchanged
Meta_1020	PC 38:2	Unclassified	Unclassified	Unclassified			-0.169333333	0.463243706920807	0.463243706920807	0.666124288696795	unchanged
Meta_1021	PC 17:1_20:4	Unclassified	Unclassified	Unclassified			-0.196333333	0.495901613633854	0.495901613633854	0.619338505233905	unchanged
Meta_1022	PC O-18:1_20:4	Unclassified	Unclassified	Unclassified			0.157333333333334	0.612896054709011	0.612896054709011	0.41344388263939	unchanged
Meta_1023	PC O-16:0_22:5	Unclassified	Unclassified	Unclassified			-0.816333333	0.373481240927318	0.373481240927318	0.853325853047538	unchanged
Meta_1024	PC O-16:0_22:4	Unclassified	Unclassified	Unclassified			-0.124333333	0.353666505224201	0.353666505224201	0.826038443420875	unchanged
Meta_1025	PC O-18:0_20:4	Unclassified	Unclassified	Unclassified			-0.273666667	0.0597837130690232	0.0597837130690232	1.44945883521796	unchanged
Meta_1026	PC 18:1_19:1	Unclassified	Unclassified	Unclassified			-0.138	0.234039091264391	0.234039091264391	1.01184765265479	unchanged
Meta_1027	PG(i-22:0/18:1(12Z)-O(9S,10R))	Unclassified	Unclassified	Unclassified	HMDB0272143		0.0543333333333332	0.516939217920062	0.516939217920062	0.637165066846543	unchanged
Meta_1028	PC(16:0/22:6(4Z,7Z,10Z,13Z,16Z,19Z))	Lipids and lipid-like molecules	Glycerophospholipids	Glycerophosphocholines	HMDB0007991		-0.151	0.246761213393952	0.246761213393952	0.984412314107375	unchanged
Meta_1029	PC(18:1(9Z)/20:5(5Z,8Z,11Z,14Z,17Z))	Lipids and lipid-like molecules	Glycerophospholipids	Glycerophosphocholines	HMDB0008116		0.346666666666668	0.330958146641114	0.330958146641114	0.884410902647994	unchanged
Meta_1030	PC(18:1(9Z)/20:4(8Z,11Z,14Z,17Z))	Lipids and lipid-like molecules	Glycerophospholipids	Glycerophosphocholines	HMDB0008115		0.0313333333333325	0.575206530293841	0.575206530293841	0.48805498964164	unchanged
Meta_1031	PC(18:0/20:4(8Z,11Z,14Z,17Z))	Lipids and lipid-like molecules	Glycerophospholipids	Glycerophosphocholines	HMDB0008049		0.396666666666667	0.134640712700094	0.134640712700094	1.34050769768647	unchanged
Meta_1032	PC 18:1_20:3	Unclassified	Unclassified	Unclassified			-0.758666667	0.224264889127087	0.224264889127087	1.12898668494892	unchanged
Meta_1033	PC(18:1(9Z)/20:2(11Z,14Z))	Lipids and lipid-like molecules	Glycerophospholipids	Glycerophosphocholines	HMDB0008111		0.274333333333335	0.402369919271077	0.402369919271077	0.800755682234197	unchanged
Meta_1034	SM 8:0;2O/34:2	Unclassified	Unclassified	Unclassified			-0.474666667	0.0512074454609837	0.0512074454609837	1.4021280589762	unchanged
Meta_1035	PC(18:1(9Z)/20:1(11Z))	Lipids and lipid-like molecules	Glycerophospholipids	Glycerophosphocholines	HMDB0008110		-0.077	0.649089034955338	0.649089034955338	0.365457542151153	unchanged
Meta_1036	SM 8:0;2O/34:1	Unclassified	Unclassified	Unclassified			-0.148333333	0.884029285879003	0.884029285879003	0.102451263727804	unchanged
Meta_1037	LacCer(d18:1/16:0)	Lipids and lipid-like molecules	Sphingolipids	Glycosphingolipids	HMDB0006750	C01290	-0.471	0.217823094717801	0.217823094717801	1.05491699461782	unchanged
Meta_1038	PC(18:1(9Z)/22:6(4Z,7Z,10Z,13Z,16Z,19Z))	Lipids and lipid-like molecules	Glycerophospholipids	Glycerophosphocholines	HMDB0008123		-0.017333333	0.925431512048242	0.925431512048242	0.0997483378295418	unchanged
Meta_1039	PC(18:1(9Z)/22:5(7Z,10Z,13Z,16Z,19Z))	Lipids and lipid-like molecules	Glycerophospholipids	Glycerophosphocholines	HMDB0008122		0.402000000000001	0.0695401584119077	0.0695401584119077	1.34747223336335	unchanged
Meta_104	1-Naphthylamine	Benzenoids	Naphthalenes	Unclassified	HMDB0243964	C14790	0.222666666666665	0.178572803315213	0.178572803315213	1.1079205704514	unchanged
Meta_105	Oxoglutaric acid	Organic acids and derivatives	Keto acids and derivatives	Gamma-keto acids and derivatives	HMDB0000208	C00026	0.341333333333335	0.533701317381582	0.533701317381582	0.548209933172469	unchanged
Meta_107	L-Glutamic acid	Organic acids and derivatives	Carboxylic acids and derivatives	Amino acids, peptides, and analogues	HMDB0000148	C00025	0.303333333333335	0.515377422304075	0.515377422304075	0.602252079815298	unchanged
Meta_108	Indole-3-carboxaldehyde	Organoheterocyclic compounds	Indoles and derivatives	Indoles	HMDB0029737	C08493	0.102666666666668	0.300507069488946	0.300507069488946	0.875976313599659	unchanged
Meta_109	2-Aminoheptanoate	Organic acids and derivatives	Carboxylic acids and derivatives	Amino acids, peptides, and analogues	HMDB0094649		0.251666666666665	0.166974437620794	0.166974437620794	1.19067213782826	unchanged
Meta_11	2-Methyl-1-propylamine	Organic nitrogen compounds	Organonitrogen compounds	Amines	HMDB0034198	C02787	-0.108666667	0.876229994103249	0.876229994103249	0.120741718842456	unchanged
Meta_110	Acetylcholine	Organic nitrogen compounds	Organonitrogen compounds	Quaternary ammonium salts	HMDB0000895	C01996	0.0626666666666687	0.428163997088314	0.428163997088314	0.702984439332338	unchanged
Meta_111	Spermidine	Organic nitrogen compounds	Organonitrogen compounds	Amines	HMDB0001257	C00315	-0.462	0.373373810773317	0.373373810773317	0.780999919315135	unchanged
Meta_112	L-2-Hydroxyglutaric acid	Organic acids and derivatives	Hydroxy acids and derivatives	Short-chain hydroxy acids and derivatives	HMDB0000694	C03196	0.0696666666666665	0.852124209745485	0.852124209745485	0.163449481767041	unchanged
Meta_113	Propyl 2-mercaptopropionate	Organic acids and derivatives	Carboxylic acids and derivatives	Carboxylic acid derivatives	HMDB0032495		0.363	0.239528513590511	0.239528513590511	0.954591784854217	unchanged
Meta_114	L-Lysine	Organic acids and derivatives	Carboxylic acids and derivatives	Amino acids, peptides, and analogues	HMDB0000182	C00047	0.254000000000001	0.140352705532193	0.140352705532193	1.28139878892369	unchanged
Meta_115	Phthalic anhydride	Organoheterocyclic compounds	Benzofurans	Benzofuranones	HMDB0256501		0.472000000000001	0.0906933702890426	0.0906933702890426	1.2921686785075	unchanged

Meta_116	L-Methionine	Organic acids and derivatives	Carboxylic acids and derivatives	Amino acids, peptides, and analogues	HMDB0000696	C00073	0.05266666666666671	0.730304457399181	0.730304457399181	0.357640851634532	unchanged
Meta_117	Xanthine	Organoheterocyclic compounds	Imidazopyrimidines	Purines and purine derivatives	HMDB0000292	C00385	-0.485333333	0.124149580347831	0.124149580347831	1.16591174224653	unchanged
Meta_118	Guanine	Organoheterocyclic compounds	Imidazopyrimidines	Purines and purine derivatives	HMDB0000132	C00242	-0.063666667	0.825003021998995	0.825003021998995	0.25865158899923	unchanged
Meta_119	Dopamine	Benzenoids	Phenols	Benzenediols	HMDB0000073	C03758	-0.714	0.129182295055049	0.129182295055049	1.24637238486522	unchanged
Meta_12	Propionic acid	Organic acids and derivatives	Carboxylic acids and derivatives	Carboxylic acids	HMDB0000237	C00163	0.709	0.365300987767101	0.365300987767101	0.811213699794296	unchanged
Meta_120	2-Azaspiro[4.5]decan-3-one	Organoheterocyclic compounds	Azaspirodecane derivatives	Unclassified	HMDB0247166		-0.774666667	0.235710074759483	0.235710074759483	1.08514938912125	unchanged
Meta_121	4-Chlorobenzoic acid	Benzenoids	Benzene and substituted derivatives	Benzoic acids and derivatives	HMDB0246393	C02370	0.06466666666666675	0.740366506164361	0.740366506164361	0.29216062727566	unchanged
Meta_123	Dimethyl sulfoxide	Organosulfur compounds	Sulfoxides	Unclassified	HMDB0002151	C11143	0.207000000000001	0.470433851160013	0.470433851160013	0.665464461766845	unchanged
Meta_124	2,5-Dimethyl-4-ethoxy-3(2H)-furanone	Organoheterocyclic compounds	Dihydrofurans	Furanones	HMDB0032232		-0.529	0.144349277078658	0.144349277078658	1.28744632109354	unchanged
Meta_125	N-Acetylvaline	Organic acids and derivatives	Carboxylic acids and derivatives	Amino acids, peptides, and analogues	HMDB0011757		-0.191666667	0.485178923867564	0.485178923867564	0.696022820293626	unchanged
Meta_126	3a,6a,7b-Trihydroxy-5b-cholanoic acid	Lipids and lipid-like molecules	Steroids and steroid derivatives	Bile acids, alcohols and derivatives	HMDB0000364		0.06766666666666659	0.63095445808178	0.63095445808178	0.405480229383973	unchanged
Meta_127	Citiolone	Organic acids and derivatives	Carboxylic acids and derivatives	Amino acids, peptides, and analogues	HMDB0250301		0.212333333333333	0.559164068521051	0.559164068521051	0.573763954322391	unchanged
Meta_128	8-Hydroxy-2-methylquinoline	Unclassified	Unclassified	Unclassified			0.272333333333334	0.305289592480754	0.305289592480754	0.99090801936656	unchanged
Meta_129	1-Propanol, 3,3,3-trichloro-	Organic oxygen compounds	Organooxygen compounds	Alcohols and polyols	HMDB0244002		0.955333333333336	0.355943318612398	0.355943318612398	0.881366426710209	unchanged
Meta_13	Glycine	Organic acids and derivatives	Carboxylic acids and derivatives	Amino acids, peptides, and analogues	HMDB0000123	C00037	0.239666666666668	0.421279919569703	0.421279919569703	0.72418219193153	unchanged
Meta_130	2-Naphthalenethiol	Benzenoids	Naphthalenes	Unclassified	HMDB0029689		-0.244	0.165348993680282	0.165348993680282	1.14192591885287	unchanged
Meta_131	3-Hydroxymethylglutaric acid	Lipids and lipid-like molecules	Fatty Acyls	Fatty acids and conjugates	HMDB0000355	C03761	0.258666666666663	0.338315809200388	0.338315809200388	0.845305214001269	unchanged
Meta_133	Glutamic acid gamma-methyl ester	Organic acids and derivatives	Carboxylic acids and derivatives	Amino acids, peptides, and analogues	HMDB0061715		0.545000000000002	0.0817848292549927	0.0817848292549927	1.34990647097474	unchanged
Meta_134	L-Carnitine	Organic nitrogen compounds	Organonitrogen compounds	Quaternary ammonium salts	HMDB0000062	C00318	-0.401666667	0.208741675972014	0.208741675972014	1.08920277716026	unchanged
Meta_136	(S1)-Methoxy-3-heptanethiol	Organic oxygen compounds	Organooxygen compounds	Ethers	HMDB0032380		-0.053333333	0.696755909879285	0.696755909879285	0.365702836321882	unchanged
Meta_137	Methionine methyl ester	Organic acids and derivatives	Carboxylic acids and derivatives	Amino acids, peptides, and analogues	HMDB0254602		-0.327666667	0.355163063718623	0.355163063718623	0.820818899242114	unchanged
Meta_138	Chloral hydrate	Organohalogen compounds	Halohydrins	Chlorohydrins	HMDB0060451	C06899	0.407333333333333	0.348259459410679	0.348259459410679	0.835310292030755	unchanged
Meta_14	Cyclohexa-1,2,4,5-tetraene	Allenes	Cyclic allenes	Unclassified	HMDB0248980		-0.021666667	0.770587912989176	0.770587912989176	0.230563227302949	unchanged
Meta_140	Phthalic acid	Benzenoids	Benzene and substituted derivatives	Benzoic acids and derivatives	HMDB0002107	C01606	-0.152333333	0.803816693128327	0.803816693128327	0.180006426705712	unchanged
Meta_141	1-Methylxanthine	Organoheterocyclic compounds	Imidazopyrimidines	Purines and purine derivatives	HMDB0010738	C16358	-0.565333333	0.0614372850361541	0.0614372850361541	1.42898832950993	unchanged
Meta_142	L-3-Phenyllactic acid	Phenylpropanoids and polyketides	Phenylpropanoic acids	Unclassified	HMDB0000748	C05607	-0.12	0.57868144674881	0.57868144674881	0.456537774459054	unchanged
Meta_143	L-Phenylalanine	Organic acids and derivatives	Carboxylic acids and derivatives	Amino acids, peptides, and analogues	HMDB0000159	C00079	-0.085666667	0.540994310144537	0.540994310144537	0.558194756825843	unchanged
Meta_145	Uric acid	Organoheterocyclic compounds	Imidazopyrimidines	Purines and purine derivatives	HMDB0000289	C00366	-0.148333333	0.403935233625382	0.403935233625382	0.750053034245483	unchanged
Meta_147	Pyridoxal	Organoheterocyclic compounds	Pyridines and derivatives	Pyridine carboxaldehydes	HMDB0001545	C00250	-0.315	0.0930501389038178	0.0930501389038178	1.32411701588481	unchanged
Meta_148	Chloromethyl chloroformate	Organic acids and derivatives	Organic carbonic acids and derivatives	Unclassified	HMDB0250119		0.393666666666668	0.385061982044753	0.385061982044753	0.772672974771759	unchanged
Meta_15	Dichloromethylene	Organohalogen compounds	Organochlorides	Unclassified	HMDB0251189		-0.003	0.985379164144539	0.985379164144539	0.016858794860668	unchanged
Meta_150	1-Naphthyl isocyanate	Benzenoids	Naphthalenes	Unclassified	HMDB0243960		0.149999999999999	0.154506729779817	0.154506729779817	1.15273183757155	unchanged
Meta_151	Pyridoxine	Organoheterocyclic compounds	Pyridines and derivatives	Pyridoxines	HMDB0000239	C00314	-0.210333333	0.265999371137611	0.265999371137611	0.925450061932188	unchanged
Meta_152	Glycerophosphoric acid	Lipids and lipid-like molecules	Glycerophospholipids	Glycerophosphates	HMDB0252849	C03189	0.05366666666666648	0.918429545014396	0.918429545014396	0.0902441780668146	unchanged
Meta_153	Capric acid	Lipids and lipid-like molecules	Fatty Acyls	Fatty acids and conjugates	HMDB0000511	C01571	0.0056666666666666649	0.927878132634351	0.927878132634351	0.106648638127579	unchanged
Meta_154	Hexanoylglycine	Organic acids and derivatives	Carboxylic acids and derivatives	Amino acids, peptides, and analogues	HMDB0000701		-0.096	0.813926827999898	0.813926827999898	0.191816728417973	unchanged
Meta_155	Gabapentin hydrochloride	Unclassified	Unclassified	Unclassified			0.274333333333331	0.675339050217343	0.675339050217343	0.371038067976325	unchanged
Meta_156	Phenol sulphate	Organic acids and derivatives	Organic sulfuric acids and derivatives	Arylsulfates	HMDB0060015	C02180	1.151	0.0706730704602419	0.0706730704602419	1.48397906161741	unchanged
Meta_158	Suberic acid	Lipids and lipid-like molecules	Fatty Acyls	Fatty acids and conjugates	HMDB0000893	C08278	-0.124	0.70725620642079	0.70725620642079	0.29794469009618	unchanged
Meta_159	(卤)-3-Hydroxynonanoic acid	Organic acids and derivatives	Hydroxy acids and derivatives	Medium-chain hydroxy acids and derivatives	HMDB0031513		0.174333333333333	0.371797936809363	0.371797936809363	0.801292734389161	unchanged
Meta_16	4-Hydroxy-2-butenoic acid gamma-lactone	Organoheterocyclic compounds	Dihydrofurans	Furanones	HMDB0032330	C17601	0.04666666666666669	0.790382512545324	0.790382512545324	0.244694595491266	unchanged
Meta_160	N-Acetyl-L-aspartic acid	Organic acids and derivatives	Carboxylic acids and derivatives	Amino acids, peptides, and analogues	HMDB0000812	C01042	0.450333333333333	0.0863122710193187	0.0863122710193187	1.33302847288456	unchanged
Meta_162	4-(Dimethylamino)butyl carbamimidothioate	Organosulfur compounds	Isothioureas	Unclassified	HMDB0246305		-0.054333333	0.174163076423722	0.174163076423722	1.10374944385445	unchanged
Meta_163	Ureidosuccinic acid	Organic acids and derivatives	Carboxylic acids and derivatives	Amino acids, peptides, and analogues	HMDB0000828		1.48433333333333	0.128748879438554	0.128748879438554	1.22190123645779	unchanged
Meta_166	N-Formyl-L-methionine	Organic acids and derivatives	Carboxylic acids and derivatives	Amino acids, peptides, and analogues	HMDB0001015	C03145	0.364999999999998	0.0806047801014571	0.0806047801014571	1.34116243504793	unchanged
Meta_167	L-Alloisoleucine	Organic acids and derivatives	Carboxylic acids and derivatives	Amino acids, peptides, and analogues	HMDB0000557		-1.015666667	0.0665564751997755	0.0665564751997755	1.45175373866771	unchanged
Meta_168	Gentianine	Organoheterocyclic compounds	Pyranopyridines	Unclassified	HMDB0303030	C06525	-0.183666667	0.488321522130563	0.488321522130563	0.646398639712737	unchanged
Meta_169	Indoleacetic acid	Organoheterocyclic compounds	Indoles and derivatives	Indolyl carboxylic acids and derivatives	HMDB0000197	C00954	0.465999999999999	0.346203720631812	0.346203720631812	0.914868251614882	unchanged
Meta_17	Piperidine	Organoheterocyclic compounds	Piperidines	Unclassified	HMDB0034301	C01746	-0.287666667	0.496547618919246	0.496547618919246	0.598108105052225	unchanged
Meta_170	Methylthio 2-(propanoyloxy)propanoate	Organic acids and derivatives	Thiocarboxylic acids and derivatives	Thioesters	HMDB0040003		-0.32	0.0640167750921202	0.0640167750921202	1.42447638936514	unchanged
Meta_171	N,N-dimethylindoliumolate	Organoheterocyclic compounds	Indoles and derivatives	Unclassified	HMDB0060063		0.126333333333333	0.388667969880798	0.388667969880798	0.71005603019382	unchanged
Meta_172	Cysteinylglycine	Organic acids and derivatives	Carboxylic acids and derivatives	Amino acids, peptides, and analogues	HMDB0000078	C01419	0.764666666666667	0.192128197699331	0.192128197699331	1.06098671132312	unchanged
Meta_173	myo-Inositol	Organic oxygen compounds	Organooxygen compounds	Alcohols and polyols	HMDB0000211	C00137	-0.039333333	0.836410397246762	0.836410397246762	0.140836065835158	unchanged
Meta_175	Methyl l-phenylalaninate	Organic acids and derivatives	Carboxylic acids and derivatives	Amino acids, peptides, and analogues	HMDB0245704		-0.623666667	0.0701321700415431	0.0701321700415431	1.31633859861422	unchanged
Meta_177	Methyl Phenylglyoxalate	Benzenoids	Benzene and substituted derivatives	Benzoyl derivatives	HMDB0062605		0.124666666666666	0.355536211497568	0.355536211497568	0.752979123991865	unchanged
Meta_178	L-Tyrosine	Organic acids and derivatives	Carboxylic acids and derivatives	Amino acids, peptides, and analogues	HMDB0000158	C00082	0.223666666666666	0.140458326112368	0.140458326112368	1.18877307523085	unchanged
Meta_179	2,4-Dinitrophenol	Benzenoids	Phenols	Nitrophenols	HMDB0245462	C02496	-0.123333333	0.352341436376406	0.352341436376406	0.881431840298038	unchanged

Meta_18	Pyruvic acid	Organic acids and derivatives	Keto acids and derivatives	Alpha-keto acids and derivatives	HMDB0000243	C00022	0.223666666666666	0.666927065538024	0.666927065538024	0.387581697259788	unchanged
Meta_181	Normetanephrine	Benzenoids	Phenols	Methoxyphenols	HMDB0000819	C05589	0.195000000000004	0.390365866982553	0.390365866982553	0.770638220445865	unchanged
Meta_182	N-Heptanoylglycine	Organic acids and derivatives	Carboxylic acids and derivatives	Amino acids, peptides, and analogues	HMDB0013010		0.0323333333333338	0.860808363328379	0.860808363328379	0.171264227676821	unchanged
Meta_183	TRIBUTYLAMINE	Organic nitrogen compounds	Organonitrogen compounds	Amines	HMDB0259165		-0.708	0.25685195987834	0.25685195987834	1.02263087748977	unchanged
Meta_184	Oxypurinol	Organoheterocyclic compounds	Imidazopyrimidines	Purines and purine derivatives	HMDB0000786	C07599	-0.467666667	0.203286228366722	0.203286228366722	1.15482074759863	unchanged
Meta_185	p-Cresol sulfate	Organic acids and derivatives	Organic sulfuric acids and derivatives	Arylsulfates	HMDB0011635		0.164	0.384664766958597	0.384664766958597	0.785065865575855	unchanged
Meta_187	Azelaic acid	Lipids and lipid-like molecules	Fatty Acyls	Fatty acids and conjugates	HMDB0000784	C08261	-0.128333333	0.577842041603777	0.577842041603777	0.460612758828638	unchanged
Meta_188	3-Hydroxycapric acid	Organic acids and derivatives	Hydroxy acids and derivatives	Medium-chain hydroxy acids and derivatives	HMDB0002203		-0.105	0.36500550106782	0.36500550106782	0.737851172532957	unchanged
Meta_189	N-Acetyl-L-glutamic acid	Organic acids and derivatives	Carboxylic acids and derivatives	Amino acids, peptides, and analogues	HMDB0001138	C00624	0.382999999999999	0.220720657041207	0.220720657041207	1.07057881400211	unchanged
Meta_19	L-Alanine	Organic acids and derivatives	Carboxylic acids and derivatives	Amino acids, peptides, and analogues	HMDB0000161	C00041	0.296000000000001	0.334164049361319	0.334164049361319	0.908898937158297	unchanged
Meta_190	4-Hydroxy-4-(pyridin-2-yl)butan-2-one	Unclassified	Unclassified	Unclassified			-0.120666667	0.147291573761583	0.147291573761583	1.14703270097211	unchanged
Meta_191	4-Methylquinoline-2-carboxylic acid	Unclassified	Unclassified	Unclassified			0.698333333333334	0.125573367646896	0.125573367646896	1.20396361378469	unchanged
Meta_193	Pivagabine	Organic acids and derivatives	Carboxylic acids and derivatives	Amino acids, peptides, and analogues	HMDB0246575		-0.050666667	0.879301519128214	0.879301519128214	0.133298454268794	unchanged
Meta_195	4',5'-Dihydropsoralen	Phenylpropanoids and polyketides	Coumarins and derivatives	Furanocoumarins	HMDB0302123		0.0210000000000008	0.884101406296103	0.884101406296103	0.103687500748615	unchanged
Meta_196	Leucyl-Glycine	Organic acids and derivatives	Carboxylic acids and derivatives	Amino acids, peptides, and analogues	HMDB0028929		-0.232	0.24128226433783	0.24128226433783	1.03418447664418	unchanged
Meta_197	Glycylleucine	Organic acids and derivatives	Carboxylic acids and derivatives	Amino acids, peptides, and analogues	HMDB0000759	C02155	-0.05	0.552196863444145	0.552196863444145	0.505311921447619	unchanged
Meta_199	N-Acetyl-L-methionine	Organic acids and derivatives	Carboxylic acids and derivatives	Amino acids, peptides, and analogues	HMDB0011745	C02712	0.343999999999998	0.104398297528871	0.104398297528871	1.30097496896399	unchanged
Meta_2	Thiirane	Organoheterocyclic compounds	Thiiranes	Unclassified	HMDB0258994	C19419	0.598333333333333	0.241603950163322	0.241603950163322	1.0862245306835	unchanged
Meta_20	Morpholine	Organoheterocyclic compounds	Oxazinanes	Morpholines	HMDB0031581	C14452	-1.083666667	0.157942440532506	0.157942440532506	1.14138129325076	unchanged
Meta_201	Menadione	Benzenoids	Naphthalenes	Naphthoquinones	HMDB0001892	C05377	0.887666666666668	0.169532618588603	0.169532618588603	1.17732192475959	unchanged
Meta_202	Citric acid	Organic acids and derivatives	Carboxylic acids and derivatives	Tricarboxylic acids and derivatives	HMDB0000094	C00158	0.0169999999999995	0.919614226203996	0.919614226203996	0.105161987800752	unchanged
Meta_203	Phenylacetylglycine	Organic acids and derivatives	Carboxylic acids and derivatives	Amino acids, peptides, and analogues	HMDB0000821	C05598	0.184999999999999	0.356440832486597	0.356440832486597	0.831257664030974	unchanged
Meta_204	Hydroxycotinine	Organoheterocyclic compounds	Pyridines and derivatives	Pyrrolidinylpyridines	HMDB0001390		0.00600000000000023	0.976556260909415	0.976556260909415	0.00706990324978278	unchanged
Meta_205	N-Acetylleucine	Organic acids and derivatives	Carboxylic acids and derivatives	Amino acids, peptides, and analogues	HMDB0011756	C02710	-0.069333333	0.344474370490954	0.344474370490954	0.823249783340064	unchanged
Meta_206	1,3-Dihydro-(2H)-indol-2-one	Organoheterocyclic compounds	Indoles and derivatives	Indolines	HMDB0061918	C12312	-0.804666667	0.213490323652081	0.213490323652081	1.1010656609543	unchanged
Meta_208	Ethyl Phenylglyoxylate	Benzenoids	Benzene and substituted derivatives	Benzoyl derivatives	HMDB0062729		-0.232666667	0.0846323576214165	0.0846323576214165	1.39986283370137	unchanged
Meta_209	Tyrosine methylester	Organic acids and derivatives	Carboxylic acids and derivatives	Amino acids, peptides, and analogues	HMDB0029217		-0.569666667	0.201504328771262	0.201504328771262	1.05086164642713	unchanged
Meta_21	L-Lactic acid	Organic acids and derivatives	Hydroxy acids and derivatives	Alpha hydroxy acids and derivatives	HMDB0000190	C00186	-0.16	0.701469986000571	0.701469986000571	0.356943396363405	unchanged
Meta_210	2-Hydroxyadipic acid	Lipids and lipid-like molecules	Fatty Acyls	Fatty acids and conjugates	HMDB0000321	C02360	-0.130666667	0.446731226349459	0.446731226349459	0.747316424138579	unchanged
Meta_211	methyl 2-(2-amino-4-hydroxy-6-methylpyrimidin-5-yl)acetate	Unclassified	Unclassified	Unclassified			0.161333333333333	0.550956495758029	0.550956495758029	0.540843267013146	unchanged
Meta_212	Caffeine	Organoheterocyclic compounds	Imidazopyrimidines	Purines and purine derivatives	HMDB0001847		-0.040333333	0.0633629712771455	0.0633629712771455	1.3686406061207	unchanged
Meta_213	AICA-riboside	Nucleosides, nucleotides, and analogues	Imidazole ribonucleosides and ribonucleotides	Unclassified	HMDB0062179		-0.180666667	0.185794075291982	0.185794075291982	1.21554749024809	unchanged
Meta_214	Sebacic acid	Lipids and lipid-like molecules	Fatty Acyls	Fatty acids and conjugates	HMDB0000792	C08277	0.251666666666665	0.394735821056167	0.394735821056167	0.774952788533023	unchanged
Meta_215	Simazine	Organoheterocyclic compounds	Triazines	1,3,5-triazines	HMDB0258294	C11172	-0.281333333	0.338548374794525	0.338548374794525	0.810216803008423	unchanged
Meta_216	N-Lactoylleucine	Organic acids and derivatives	Carboxylic acids and derivatives	Amino acids, peptides, and analogues	HMDB0062176		0.205333333333332	0.301654836234226	0.301654836234226	0.906497052302105	unchanged
Meta_217	Benzenesulfonic acid	Benzenoids	Benzene and substituted derivatives	Benzenesulfonic acids and derivatives	HMDB0248986		-0.203666667	0.285878719526342	0.285878719526342	0.93633684769902	unchanged
Meta_218	Theophylline	Organoheterocyclic compounds	Imidazopyrimidines	Purines and purine derivatives	HMDB0001889	C07130	0.282	0.353371843539513	0.353371843539513	0.81581394484812	unchanged
Meta_220	Alanylleucine	Organic acids and derivatives	Carboxylic acids and derivatives	Amino acids, peptides, and analogues	HMDB0028691		-0.221666667	0.419351368930587	0.419351368930587	0.736047863419819	unchanged
Meta_221	Leucylalanine	Organic acids and derivatives	Carboxylic acids and derivatives	Amino acids, peptides, and analogues	HMDB0028922		-0.044666667	0.878646093806037	0.878646093806037	0.175513973226295	unchanged
Meta_222	Spermine	Organic nitrogen compounds	Organonitrogen compounds	Amines	HMDB0001256	C00750	-0.445666667	0.612101401628539	0.612101401628539	0.45391214662274	unchanged
Meta_223	Monosodium glutamate	Organic acids and derivatives	Carboxylic acids and derivatives	Amino acids, peptides, and analogues	HMDB0303331		0.287333333333333	0.232585113786607	0.232585113786607	0.968112689085664	unchanged
Meta_226	L-Acetylcarnitine	Lipids and lipid-like molecules	Fatty Acyls	Fatty acid esters	HMDB0000201	C02571	0.0749999999999993	0.664096319738727	0.664096319738727	0.38006498885332	unchanged
Meta_228	Galactitol	Organic oxygen compounds	Organooxygen compounds	Carbohydrates and carbohydrate conjugates	HMDB0000107	C01697	-0.005666667	0.965291461608606	0.965291461608606	0.0340109319249777	unchanged
Meta_229	L-Tryptophan	Organoheterocyclic compounds	Indoles and derivatives	Indolyl carboxylic acids and derivatives	HMDB0000929	C00078	0.0359999999999978	0.752013718829626	0.752013718829626	0.292459718906363	unchanged
Meta_23	beta-Alanine	Organic acids and derivatives	Carboxylic acids and derivatives	Amino acids, peptides, and analogues	HMDB0000056	C00099	0.044666666666668	0.873748816709298	0.873748816709298	0.175556030841689	unchanged
Meta_230	Pyridoxine hydrochloride	Organoheterocyclic compounds	Pyridines and derivatives	Pyridoxines	HMDB0301760		-0.848	0.0714697211224517	0.0714697211224517	1.35708642155057	unchanged
Meta_232	1-Naphthalenesulfonic acid	Benzenoids	Naphthalenes	Naphthalene sulfonic acids and derivatives	HMDB0243955	C16201	-0.033333333	0.949118804288658	0.949118804288658	0.103970322941893	unchanged
Meta_233	Diethyl L-malate	Organic acids and derivatives	Hydroxy acids and derivatives	Beta hydroxy acids and derivatives	HMDB0040220		0.0733333333333341	0.575046787732581	0.575046787732581	0.525958797146765	unchanged
Meta_234	N-Acetylglutamine	Organic acids and derivatives	Carboxylic acids and derivatives	Amino acids, peptides, and analogues	HMDB0006029		0.188000000000001	0.76506714605737	0.76506714605737	0.295824719812424	unchanged
Meta_235	Hydrocinnamic acid	Phenylpropanoids and polyketides	Phenylpropanoic acids	Unclassified	HMDB0000764	C05629	-0.024	0.930235043713219	0.930235043713219	0.0450490769521376	unchanged
Meta_236	L-Kynurenine	Organic oxygen compounds	Organooxygen compounds	Carbonyl compounds	HMDB0000684	C00328	0.327666666666667	0.151235246983585	0.151235246983585	1.24047027936224	unchanged
Meta_237	Talipexole	Organoheterocyclic compounds	Azepines	Unclassified	HMDB0258691		-0.046666667	0.896827821087701	0.896827821087701	0.118712139498971	unchanged
Meta_239	Varenicline	Organoheterocyclic compounds	Benzazepines	Unclassified	HMDB0015398		-0.130333333	0.239988556188219	0.239988556188219	1.04363295832915	unchanged
Meta_24	2-(Methylthio)ethanol	Organosulfur compounds	Thioethers	Dialkylthioethers	HMDB0032425		-0.289	0.328911793779106	0.328911793779106	0.868886796180295	unchanged
Meta_240	Dodecatrienoic acid	Lipids and lipid-like molecules	Fatty Acyls	Fatty acids and conjugates	HMDB0302622		-0.332333333	0.111953559879193	0.111953559879193	1.26091519395721	unchanged
Meta_241	(+)-1,2-Epoxyneomenthyl acetate	Organoheterocyclic compounds	Oxepanes	Unclassified	HMDB0302747		-0.092	0.352442893882573	0.352442893882573	0.821357471897381	unchanged

Meta_242	3-Oxododecanoic acid	Organic acids and derivatives	Keto acids and derivatives	Medium-chain keto acids and derivatives	HMDB0010727	C02367	0.332666666666666	0.222578362852226	0.222578362852226	1.02331716458736	unchanged
Meta_243	1,3,5-Trinitrobenzene	Benzenoids	Benzene and substituted derivatives	Nitrobenzenes	HMDB0244187		0.335000000000001	0.102195607857515	0.102195607857515	1.2429476618583	unchanged
Meta_244	5-Hydroxyindoleacetic acid	Organoheterocyclic compounds	Indoles and derivatives	Indolyl carboxylic acids and derivatives	HMDB0000763	C05635	-1.899666667	0.137296809504795	0.137296809504795	1.30107994714777	unchanged
Meta_245	N-Nonanoylglycine	Organic acids and derivatives	Carboxylic acids and derivatives	Amino acids, peptides, and analogues	HMDB0013279		-0.333666667	0.236319072059598	0.236319072059598	1.0100631505764	unchanged
Meta_247	1-Pyrenemethanol	Benzenoids	Pyrenes	Unclassified	HMDB0244007		0.497666666666666	0.234833160240006	0.234833160240006	1.06359377484196	unchanged
Meta_248	Undecanedioic acid	Lipids and lipid-like molecules	Fatty Acyls	Fatty acids and conjugates	HMDB0000888		0.05566666666666672	0.88306022307795	0.88306022307795	0.17558502794447	unchanged
Meta_249	3-Hydroxydodecanoic acid	Organic acids and derivatives	Hydroxy acids and derivatives	Medium-chain hydroxy acids and derivatives	HMDB0000387		0.02166666666666665	0.894419105223412	0.894419105223412	0.158196182853104	unchanged
Meta_250	N-Formylphenylalanine	Organic acids and derivatives	Carboxylic acids and derivatives	Amino acids, peptides, and analogues	HMDB0240317		-0.026333333	0.866132189277429	0.866132189277429	0.112821844636457	unchanged
Meta_251	xi-5-Dodecanolide	Organoheterocyclic compounds	Lactones	Delta valerolactones	HMDB0037742		-0.177666667	0.613387514219504	0.613387514219504	0.449775002196139	unchanged
Meta_252	Glyceraldehyde phosphate	Organic acids and derivatives	Organic phosphoric acids and derivatives	Unclassified	HMDB0252837		-0.161	0.277147762982276	0.277147762982276	0.905771823497713	unchanged
Meta_253	Alanylglutamic acid	Organic acids and derivatives	Carboxylic acids and derivatives	Amino acids, peptides, and analogues	HMDB0028686		0.623999999999999	0.0924089436321222	0.0924089436321222	1.32473898126442	unchanged
Meta_254	1-Methylpyrene	Benzenoids	Pyrenes	Unclassified	HMDB0243945		-0.323333333	0.637547730676109	0.637547730676109	0.442562011467285	unchanged
Meta_255	(2R)-2-Amino-2-[[(1S)-1-carboxyethyl]amino]-4-methylpentanoic acid	Organic acids and derivatives	Carboxylic acids and derivatives	Amino acids, peptides, and analogues	HMDB0242765		-0.165333333	0.21746346696838	0.21746346696838	1.04351802853667	unchanged
Meta_256	Valylvaline	Organic acids and derivatives	Carboxylic acids and derivatives	Amino acids, peptides, and analogues	HMDB0029140		-0.501	0.105338025155941	0.105338025155941	1.37896394111105	unchanged
Meta_258	Pantothenic acid	Organic oxygen compounds	Organooxygen compounds	Alcohols and polyols	HMDB0000210	C00864	0.107666666666667	0.3576021415971	0.3576021415971	0.80740626001382	unchanged
Meta_26	Cytosine	Organoheterocyclic compounds	Diazines	Pyrimidines and pyrimidine derivatives	HMDB0000630	C00380	-0.048333333	0.606982428208357	0.606982428208357	0.464543581073634	unchanged
Meta_261	Propionylcarnitine	Lipids and lipid-like molecules	Fatty Acyls	Fatty acid esters	HMDB0000824		0.366666666666667	0.082046378015806	0.082046378015806	1.29243212386821	unchanged
Meta_262	Alanylmethionine	Organic acids and derivatives	Carboxylic acids and derivatives	Amino acids, peptides, and analogues	HMDB0028693		-0.672333333	0.348750501712036	0.348750501712036	0.846888100994973	unchanged
Meta_263	Methyl 2-amino-3-(1H-indol-3-yl)propanoate	Organic acids and derivatives	Carboxylic acids and derivatives	Amino acids, peptides, and analogues	HMDB0254563		-0.165666667	0.604988526956161	0.604988526956161	0.458532810363711	unchanged
Meta_265	6-Chloro-3-cyano-7-methylchromone	Unclassified	Unclassified	Unclassified			-0.301666667	0.500167643547557	0.500167643547557	0.607391640510667	unchanged
Meta_266	(5-Methyl-1,3-dioxo-1,3-dihydro-2H-isoindol-2-yl)acetic acid	Unclassified	Unclassified	Unclassified			0.0470000000000006	0.860576338007183	0.860576338007183	0.148433648049798	unchanged
Meta_267	N-lactoyl-Methionine	Organic acids and derivatives	Carboxylic acids and derivatives	Amino acids, peptides, and analogues	HMDB0062182		0.247333333333335	0.195682139867508	0.195682139867508	1.13661050268012	unchanged
Meta_268	ID11614	Organoheterocyclic compounds	Diazinanes	Piperazines	HMDB0060827		-0.137333333	0.62896961139115	0.62896961139115	0.459699387539529	unchanged
Meta_269	Diethyl phthalic acid	Benzenoids	Benzene and substituted derivatives	Benzoic acids and derivatives	HMDB0094660	C14175	-0.163	0.596688156453204	0.596688156453204	0.450106669603231	unchanged
Meta_27	Phenol	Benzenoids	Phenols	1-hydroxy-4-unsubstituted benzenoids	HMDB0000228	C15584; C00146	0.174666666666667	0.362230830195467	0.362230830195467	0.850185585790815	unchanged
Meta_270	5-Hydroxy-L-tryptophan	Organoheterocyclic compounds	Indoles and derivatives	Tryptamines and derivatives	HMDB0000472	C00643	0.103	0.823044053729137	0.823044053729137	0.237481230149582	unchanged
Meta_271	2,5-Di-tert-butylhydroquinone	Benzenoids	Benzene and substituted derivatives	Phenylpropanes	HMDB0245492		-0.041666667	0.902342090907224	0.902342090907224	0.0652715259696297	unchanged
Meta_272	Methyl 4-chloro-1H-indole-3-acetate	Organoheterocyclic compounds	Indoles and derivatives	Indolyl carboxylic acids and derivatives	HMDB0032937		-0.016	0.929501764300493	0.929501764300493	0.0442502287505933	unchanged
Meta_273	L-Cystathionine	Organic acids and derivatives	Carboxylic acids and derivatives	Amino acids, peptides, and analogues	HMDB0000099	C02291	0.671333333333333	0.0670500878249574	0.0670500878249574	1.34979266193746	unchanged
Meta_274	L-Threo-3-Phenylserine	Organic acids and derivatives	Carboxylic acids and derivatives	Amino acids, peptides, and analogues	HMDB0002184		-0.194	0.210870685476958	0.210870685476958	1.05085972093903	unchanged
Meta_276	Dinitolmide	Benzenoids	Benzene and substituted derivatives	Toluenes	HMDB0032558		0.144	0.2577333975467	0.2577333975467	1.01133381124552	unchanged
Meta_277	Chlorquinaldol	Organoheterocyclic compounds	Quinolines and derivatives	Haloquinolines	HMDB0250139		0.0689999999999991	0.788802059994062	0.788802059994062	0.2115206880593	unchanged
Meta_278	L-Glutamic acid 5-phosphate	Organic acids and derivatives	Carboxylic acids and derivatives	Amino acids, peptides, and analogues	HMDB0001228	C03287	0.302333333333333	0.343304977596915	0.343304977596915	0.866864712113369	unchanged
Meta_279	(R)-1,2-dimethyl-5,6-dihydroxy-tetrahydroisoquinoline	Organoheterocyclic compounds	Tetrahydroisoquinolines	Unclassified	HMDB0012484		-0.094666667	0.90921071784359	0.90921071784359	0.0723688602879609	unchanged
Meta_28	Succinic anhydride	Organic acids and derivatives	Carboxylic acids and derivatives	Dicarboxylic acids and derivatives	HMDB0032523	C19524	0.156666666666666	0.473031562193688	0.473031562193688	0.689818575103073	unchanged
Meta_280	cis-Vitamin K1	Lipids and lipid-like molecules	Prenol lipids	Quinone and hydroquinone lipids	HMDB0250298		-0.484666667	0.189424306799346	0.189424306799346	1.10246158581889	unchanged
Meta_281	Trifluoroacetic acid	Organic acids and derivatives	Carboxylic acids and derivatives	Alpha-halocarboxylic acids and derivatives	HMDB0014118		-0.250333333	0.118131636748839	0.118131636748839	1.3027505606023	unchanged
Meta_282	Carnosine	Organic acids and derivatives	Peptidomimetics	Hybrid peptides	HMDB0000033	C00386	-0.073333333	0.750196220026908	0.750196220026908	0.289314899262524	unchanged
Meta_283	Myristic acid	Lipids and lipid-like molecules	Fatty Acyls	Fatty acids and conjugates	HMDB0000806	C06424	0.01066666666666655	0.97139276028266	0.97139276028266	0.0487634195433454	unchanged
Meta_284	2-Chloro-1,3,5-triazine	Organoheterocyclic compounds	Triazines	1,3,5-triazines	HMDB0245050		-0.231333333	0.112673579605406	0.112673579605406	1.3999635265185	unchanged
Meta_285	N-Decanoylglycine	Organic acids and derivatives	Carboxylic acids and derivatives	Amino acids, peptides, and analogues	HMDB0013267		-0.728333333	0.173458619684534	0.173458619684534	1.21785579500081	unchanged
Meta_286	Methyl (2E,6Z)-dodecadienoate	Lipids and lipid-like molecules	Fatty Acyls	Fatty acid esters	HMDB0031014		-0.382	0.336058366466571	0.336058366466571	0.866741722328851	unchanged
Meta_287	Pentadecylamine	Organic nitrogen compounds	Organonitrogen compounds	Amines	HMDB0243828		-1.120666667	0.3424328520349	0.3424328520349	0.877135917987594	unchanged
Meta_288	Ibudilast	Organoheterocyclic compounds	Pyrazolopyridines	Unclassified	HMDB0015614		0.242333333333335	0.297195064709683	0.297195064709683	0.955082967062866	unchanged
Meta_289	Pro-Ile	Organic acids and derivatives	Carboxylic acids and derivatives	Amino acids, peptides, and analogues	HMDB0304810		-0.434	0.331083636823129	0.331083636823129	0.902144011032982	unchanged
Meta_29	2-Piperidinone	Organoheterocyclic compounds	Piperidines	Piperidinones	HMDB0011749		-0.152666667	0.334070050647153	0.334070050647153	0.848053682462627	unchanged
Meta_290	Dodecanedioic acid	Lipids and lipid-like molecules	Fatty Acyls	Fatty acids and conjugates	HMDB0000623	C02678	-0.064	0.882625007946947	0.882625007946947	0.105137988381489	unchanged
Meta_292	Isoleucyl-Valine	Organic acids and derivatives	Carboxylic acids and derivatives	Amino acids, peptides, and analogues	HMDB0028920		-0.050666667	0.833053402004085	0.833053402004085	0.186990874903983	unchanged
Meta_294	Lauramine oxide	Unclassified	Unclassified	Unclassified			-0.114	0.174114504652032	0.174114504652032	1.14498842472151	unchanged
Meta_295	Cassiachromone	Organoheterocyclic compounds	Benzopyrans	1-benzopyrans	HMDB0030813		-0.202333333	0.453823310726274	0.453823310726274	0.668417740054188	unchanged
Meta_296	Benzanthrone	Benzenoids	Phenanthrenes and derivatives	Unclassified	HMDB0248974		0.230666666666666	0.633038195149386	0.633038195149386	0.433968988804938	unchanged
Meta_297	Leucyl-Valine	Organic acids and derivatives	Carboxylic acids and derivatives	Amino acids, peptides, and analogues	HMDB0028942		-0.588	0.0980111665380383	0.0980111665380383	1.42284618708767	unchanged
Meta_298	Valylleucine	Organic acids and derivatives	Carboxylic acids and derivatives	Amino acids, peptides, and analogues	HMDB0029131		-2.954	0.115091979534089	0.115091979534089	1.36126967026216	unchanged
Meta_299	N-Carboxyacetyl-D-phenylalanine	Organic acids and derivatives	Carboxylic acids and derivatives	Amino acids, peptides, and analogues	HMDB0039102		0.0663333333333291	0.756462605112745	0.756462605112745	0.313301281603681	unchanged
Meta_3	Urea	Organic acids and derivatives	Organic carbonic acids and derivatives	Ureas	HMDB0000294	C00086	-0.383333333	0.264108385341543	0.264108385341543	0.965085906320727	unchanged
Meta_30	Cyclohexylamine	Organic nitrogen compounds	Organonitrogen compounds	Cyclohexylamines	HMDB0031404	C00571	-0.372333333	0.114032784273278	0.114032784273278	1.30374503220316	unchanged

Meta_300	7-Diethylamino-4-methylcoumarin	Unclassified	Unclassified	Unclassified			0.942666666666666	0.108329665432585	0.108329665432585	1.31998482720933	unchanged
Meta_301	Isobutyryl-L-carnitine	Lipids and lipid-like molecules	Fatty Acyls	Fatty acid esters	HMDB0000736		0.362000000000002	0.207309510833368	0.207309510833368	1.06678559085885	unchanged
Meta_304	Threonylleucine	Organic acids and derivatives	Carboxylic acids and derivatives	Amino acids, peptides, and analogues	HMDB0029065		-0.416666667	0.0532587179309019	0.0532587179309019	1.40566839114519	unchanged
Meta_305	Dehydrocurdione	Lipids and lipid-like molecules	Prenol lipids	Sesquiterpenoids	HMDB0035407	C16949	-0.05	0.849065662179529	0.849065662179529	0.17044184803791	unchanged
Meta_306	4-(2-Ethyl-6-methylphenyl)-5-methylmorpholin-3-one	Unclassified	Unclassified	Unclassified			0.0383333333333334	0.697977216165963	0.697977216165963	0.349988349204529	unchanged
Meta_308	Cyheptamide	Benzenoids	Dibenzocycloheptenes	Unclassified	HMDB0250690		0.171666666666667	0.536357144043206	0.536357144043206	0.598077765687197	unchanged
Meta_309	15(R)-hydroperoxy-EPE	Organoheterocyclic compounds	Diazinanes	Piperazines	HMDB0062295		0.586333333333332	0.171288019559598	0.171288019559598	1.2410593922295	unchanged
Meta_31	2-Ketobutyric acid	Organic acids and derivatives	Keto acids and derivatives	Short-chain keto acids and derivatives	HMDB0000005	C00109	0.222	0.715217802700248	0.715217802700248	0.32763025023327	unchanged
Meta_310	Phenylalanylalanine	Organic acids and derivatives	Carboxylic acids and derivatives	Amino acids, peptides, and analogues	HMDB0028988		-0.241333333	0.300175936481673	0.300175936481673	0.906283196733031	unchanged
Meta_311	Alanylphenylalanine	Organic acids and derivatives	Carboxylic acids and derivatives	Amino acids, peptides, and analogues	HMDB0028694		-0.283333333	0.143667436404969	0.143667436404969	1.27407492495396	unchanged
Meta_312	1,4-Bisdesoxycyadox	Organoheterocyclic compounds	Diazanaphthalenes	Benzodiazines	HMDB0244200		-0.177666667	0.712864134708729	0.712864134708729	0.295108292885579	unchanged
Meta_313	N,N-diethyl-3,4-dimethoxybenzamide	Unclassified	Unclassified	Unclassified			-0.540666667	0.22384615551006	0.22384615551006	1.03608611234511	unchanged
Meta_315	Pimelic acid	Lipids and lipid-like molecules	Fatty Acyls	Fatty acids and conjugates	HMDB0000857	C02656	-0.177666667	0.186971129352938	0.186971129352938	1.06406233427239	unchanged
Meta_316	Pentaethylene glycol	Organic oxygen compounds	Organooxygen compounds	Ethers	HMDB0256256		0.111666666666666	0.923530867889027	0.923530867889027	0.0776100457080386	unchanged
Meta_317	5-Acetyl-1-benzylpyrrolidin-2-one	Unclassified	Unclassified	Unclassified			0.158999999999999	0.350453114198123	0.350453114198123	0.831142438132254	unchanged
Meta_318	2,4,5,6-Phenanthrenetetrol	Benzenoids	Phenanthrenes and derivatives	Phenanthrols	HMDB0032866		0.125666666666667	0.688547094774662	0.688547094774662	0.393716849367186	unchanged
Meta_319	Croscarmellose	Organic oxygen compounds	Organooxygen compounds	Carbohydrates and carbohydrate conjugates	HMDB0303567		-0.751333333	0.306282905768527	0.306282905768527	0.937580705557568	unchanged
Meta_32	2-Aminoisobutyric acid	Organic acids and derivatives	Carboxylic acids and derivatives	Amino acids, peptides, and analogues	HMDB0001906	C03665	0.0603333333333336	0.873172772556533	0.873172772556533	0.109243793220749	unchanged
Meta_321	3-Oxotetradecanoic acid	Lipids and lipid-like molecules	Fatty Acyls	Fatty acids and conjugates	HMDB0010730		0.164	0.471153773315228	0.471153773315228	0.632352118615889	unchanged
Meta_322	N-Undecanoylglycine	Organic acids and derivatives	Carboxylic acids and derivatives	Amino acids, peptides, and analogues	HMDB0013286		-0.203333333	0.538438031950206	0.538438031950206	0.55398898552421	unchanged
Meta_323	Canthin-6-one	Alkaloids and derivatives	Indolonaphthyridine alkaloids	Unclassified	HMDB0249593	C09098	0.388333333333334	0.491950283483103	0.491950283483103	0.694074250173154	unchanged
Meta_324	Pseudouridine	Nucleosides, nucleotides, and analogues	Nucleoside and nucleotide analogues	Unclassified	HMDB0000767	C02067	-0.408333333	0.319645078914258	0.319645078914258	0.865169471573998	unchanged
Meta_325	Lumichrome	Organoheterocyclic compounds	Pteridines and derivatives	Alloxazines and isoalloxazines	HMDB0254199	C01727	0.07800000000000012	0.6888093512527	0.6888093512527	0.414338046997916	unchanged
Meta_326	Pyroglutamyl isoleucine	Unclassified	Unclassified	Unclassified			0.438000000000001	0.0848606687833511	0.0848606687833511	1.33813461662767	unchanged
Meta_327	1,11-Undecanedicarboxylic acid	Lipids and lipid-like molecules	Fatty Acyls	Fatty acids and conjugates	HMDB0002327		-0.243	0.395613470990523	0.395613470990523	0.703309313520047	unchanged
Meta_328	Falcarinone	Organic oxygen compounds	Organooxygen compounds	Carbonyl compounds	HMDB0033693	C08445	-0.327666667	0.294555753529022	0.294555753529022	0.866134680175243	unchanged
Meta_329	(R)-3-Hydroxy-tetradecanoic acid	Lipids and lipid-like molecules	Fatty Acyls	Fatty acids and conjugates	HMDB0010731		-0.135666667	0.578557615339926	0.578557615339926	0.479853614516598	unchanged
Meta_33	L-Homoserine	Organic acids and derivatives	Carboxylic acids and derivatives	Amino acids, peptides, and analogues	HMDB0000719	C00263	0.131999999999998	0.463820977311538	0.463820977311538	0.627162799376864	unchanged
Meta_330	N-Acetylmannosamine	Organic oxygen compounds	Organooxygen compounds	Carbohydrates and carbohydrate conjugates	HMDB0001129	C00645	0.753000000000002	0.0817147174386021	0.0817147174386021	1.32391351114106	unchanged
Meta_331	Isopentenyl pyrophosphate	Lipids and lipid-like molecules	Prenol lipids	Isoprenoid phosphates	HMDB0001347	C00129	-0.285333333	0.165811366092951	0.165811366092951	1.11174879481504	unchanged
Meta_332	Glycerophosphoglycerol	Organic acids and derivatives	Organic phosphoric acids and derivatives	Phosphate esters	HMDB0240316		-0.546333333	0.385031088501146	0.385031088501146	0.855433030985843	unchanged
Meta_333	Isoleucyl-Leucine	Organic acids and derivatives	Carboxylic acids and derivatives	Amino acids, peptides, and analogues	HMDB0028911		-0.521	0.142555204668973	0.142555204668973	1.18450449480301	unchanged
Meta_334	Leucylleucine	Organic acids and derivatives	Carboxylic acids and derivatives	Amino acids, peptides, and analogues	HMDB0028933	C11332	0.07633333333333307	0.806264296722398	0.806264296722398	0.241658617692166	unchanged
Meta_335	Tetraacetylethylenediamine	Organic acids and derivatives	Carboxylic acids and derivatives	Carboxylic acid derivatives	HMDB0040573		-0.355	0.414228815469989	0.414228815469989	0.720423842341673	unchanged
Meta_336	Bisphenol A	Benzenoids	Benzene and substituted derivatives	Diphenylmethanes	HMDB0032133	C13624	0.862	0.115397429366242	0.115397429366242	1.19659682831835	unchanged
Meta_337	Isovalerylcarnitine	Lipids and lipid-like molecules	Fatty Acyls	Fatty acid esters	HMDB0000688		-0.092666667	0.596935177276657	0.596935177276657	0.433285352705429	unchanged
Meta_338	Calcium galactarate	Organic oxygen compounds	Organooxygen compounds	Carbohydrates and carbohydrate conjugates	HMDB0303726		-0.182666667	0.321467504300378	0.321467504300378	0.892732256842081	unchanged
Meta_339	3-Indolehydracrylic acid	Organoheterocyclic compounds	Indoles and derivatives	Indolyl carboxylic acids and derivatives	HMDB0059765		1.29833333333333	0.398019428373267	0.398019428373267	0.725764730223051	unchanged
Meta_34	alpha-Hydroxyisobutyric acid	Organic acids and derivatives	Hydroxy acids and derivatives	Alpha hydroxy acids and derivatives	HMDB0000729		-0.089	0.297993411276797	0.297993411276797	0.91457607702343	unchanged
Meta_340	gamma-Glutamylvaline	Organic acids and derivatives	Carboxylic acids and derivatives	Amino acids, peptides, and analogues	HMDB0011172		0.366333333333333	0.120902798466806	0.120902798466806	1.32950257742147	unchanged
Meta_343	Zederone	Lipids and lipid-like molecules	Prenol lipids	Triterpenoids	HMDB0036767		0.370999999999999	0.176907659517931	0.176907659517931	1.16863801102814	unchanged
Meta_344	3-Hydroxybutyrylcarnitine	Lipids and lipid-like molecules	Fatty Acyls	Fatty acid esters	HMDB0013127		0.0096666666666666782	0.983719191804612	0.983719191804612	0.00391854792080848	unchanged
Meta_345	3,4-Dichloroisocoumarin	Phenylpropanoids and polyketides	Isocoumarins and derivatives	Unclassified	HMDB0246026		0.546999999999999	0.0759372384937891	0.0759372384937891	1.36300813667296	unchanged
Meta_346	2,3 or 10-Mercaptopinane	Lipids and lipid-like molecules	Prenol lipids	Monoterpenoids	HMDB0032377		-0.727	0.154831236826937	0.154831236826937	1.29393521650188	unchanged
Meta_347	Pindolol	Organoheterocyclic compounds	Indoles and derivatives	Indoles	HMDB0015095	C07445	-0.563333333	0.240784308184004	0.240784308184004	1.039092836	unchanged
Meta_348	2,6-Di-tert-butyl-4-nitrophenol	Unclassified	Unclassified	Unclassified			0.104333333333333	0.408034880924743	0.408034880924743	0.780484719336949	unchanged
Meta_349	N-lactoyl-Tyrosine	Organic acids and derivatives	Carboxylic acids and derivatives	Amino acids, peptides, and analogues	HMDB0062177		0.509	0.0747155310570447	0.0747155310570447	1.33319875558885	unchanged
Meta_350	L-Serine	Organic acids and derivatives	Carboxylic acids and derivatives	Amino acids, peptides, and analogues	HMDB0000187	C00065	-0.144333333	0.41368523919311	0.41368523919311	0.708937587242701	unchanged
Meta_352	Ritalinic acid	Organic nitrogen compounds	Organonitrogen compounds	Amines	HMDB0042008		0.08033333333333338	0.892986188518448	0.892986188518448	0.10015246154522	unchanged
Meta_353	cis-4-Carboxymethylenebut-2-en-4-olide	Organoheterocyclic compounds	Dihydrofurans	Furanones	HMDB0060459	C04431	0.07733333333333337	0.681285677304547	0.681285677304547	0.387559575113168	unchanged
Meta_354	Serylphenylalanine	Organic acids and derivatives	Carboxylic acids and derivatives	Amino acids, peptides, and analogues	HMDB0029046		-0.455	0.166997259691756	0.166997259691756	1.22681124377215	unchanged
Meta_355	Methyl (S)-2-Methylbutanoate	Lipids and lipid-like molecules	Fatty Acyls	Fatty acid esters	HMDB0029762		-0.016	0.852529855393322	0.852529855393322	0.205427597965267	unchanged
Meta_356	Palmitoleic acid	Lipids and lipid-like molecules	Fatty Acyls	Fatty acids and conjugates	HMDB0003229	C08362	0.274666666666667	0.363593083585064	0.363593083585064	0.801097700140004	unchanged
Meta_357	6-Chloro-4-hydroxy-7-methyl-3-nitrocoumarin	Unclassified	Unclassified	Unclassified			-0.188333333	0.681151438787881	0.681151438787881	0.396009576796114	unchanged
Meta_358	Ketorolac	Organic oxygen compounds	Organooxygen compounds	Carbonyl compounds	HMDB0014608	C07062	0.866666666666667	0.232730550455052	0.232730550455052	0.962805430559245	unchanged

Meta_359	10-Azabenzo(a)pyrene	Unclassified	Unclassified	Unclassified			-0.29	0.22533911716176	0.22533911716176	1.08817495114921	unchanged
Meta_360	5-Methoxy-3,4-methylenedioxyamphetamine	Organoheterocyclic compounds	Benzodioxoles	Unclassified	HMDB0254817		-0.149666667	0.766709625935902	0.766709625935902	0.246576466378265	unchanged
Meta_361	O-Demethylmetoprolol	Benzenoids	Phenols	Tyrosols and derivatives	HMDB0255850		-0.022333333	0.911447317269158	0.911447317269158	0.140213918419904	unchanged
Meta_362	Liquiritigenin	Phenylpropanoids and polyketides	Flavonoids	Flavans	HMDB0029519	C09762	0.07566666666666668	0.825439903323995	0.825439903323995	0.222518210040635	unchanged
Meta_363	7,12-Dimethylbenz[a]anthracene	Benzenoids	Phenanthrenes and derivatives	Unclassified	HMDB0062429	C19488	0.503666666666668	0.0504827962910853	0.0504827962910853	1.42659263753826	unchanged
Meta_364	2-Hexyldecanoic acid	Unclassified	Unclassified	Unclassified			-0.168	0.501751457554325	0.501751457554325	0.597321878329919	unchanged
Meta_365	Palmitic acid	Lipids and lipid-like molecules	Fatty Acyls	Fatty acids and conjugates	HMDB0000220	C00249	-0.175666667	0.520051466640257	0.520051466640257	0.542720784290345	unchanged
Meta_366	Palmitic amide	Lipids and lipid-like molecules	Fatty Acyls	Fatty amides	HMDB0012273		-2.299	0.103442247822603	0.103442247822603	1.28467897863224	unchanged
Meta_367	Alternariol	Phenylpropanoids and polyketides	Coumarins and derivatives	Unclassified	HMDB0030831	C16838	-0.201666667	0.406405453623451	0.406405453623451	0.750520440250481	unchanged
Meta_368	2'-O-Methyluridine	Nucleosides, nucleotides, and analogues	Pyrimidine nucleosides	Unclassified	HMDB0240328		-0.145	0.345184272311999	0.345184272311999	0.840351382484832	unchanged
Meta_369	Lumiflavin	Organoheterocyclic compounds	Pteridines and derivatives	Alloxazines and isoalloxazines	HMDB0254201		0.491666666666667	0.234817547059618	0.234817547059618	1.06289986131867	unchanged
Meta_370	Tetradecanedioic acid	Lipids and lipid-like molecules	Fatty Acyls	Fatty acids and conjugates	HMDB0000872		-0.281	0.558297340204087	0.558297340204087	0.527344477522952	unchanged
Meta_372	Mannose 6-phosphate	Organic oxygen compounds	Organooxygen compounds	Carbohydrates and carbohydrate conjugates	HMDB0001078		0.369666666666667	0.122600927368769	0.122600927368769	1.33964901989133	unchanged
Meta_374	gamma-Glutamylleucine	Organic acids and derivatives	Carboxylic acids and derivatives	Amino acids, peptides, and analogues	HMDB0011171		0.280333333333333	0.366876794460179	0.366876794460179	0.868274195490811	unchanged
Meta_376	Glutaminylisoleucine	Organic acids and derivatives	Carboxylic acids and derivatives	Amino acids, peptides, and analogues	HMDB0028800		0.0056666666666666826	0.977634390980904	0.977634390980904	0.0735590761077725	unchanged
Meta_377	4-Methylpentanoylcarnitine	Lipids and lipid-like molecules	Fatty Acyls	Fatty acid esters	HMDB0241032		-0.090333333	0.734005346166817	0.734005346166817	0.30917789034108	unchanged
Meta_378	Mannitol 1-phosphate	Organic oxygen compounds	Organooxygen compounds	Carbohydrates and carbohydrate conjugates	HMDB0001530	C00644	-0.114666667	0.402305291960102	0.402305291960102	0.709085805659273	unchanged
Meta_379	Celereoin	Phenylpropanoids and polyketides	Coumarins and derivatives	Furanocoumarins	HMDB0039027		0.269333333333334	0.577252169266202	0.577252169266202	0.47246895973427	unchanged
Meta_38	4-Hydroxybenzaldehyde	Organic oxygen compounds	Organooxygen compounds	Carbonyl compounds	HMDB0011718	C00633	0.0023333333333333263	0.98068854826982	0.98068854826982	0.0569991359688515	unchanged
Meta_380	2-(4-oxoquinazolin-3(4H)-yl)ethyl isobutyrate	Unclassified	Unclassified	Unclassified			0.05766666666666696	0.955810971153933	0.955810971153933	0.0432976093443469	unchanged
Meta_381	Imazapyr	Organic acids and derivatives	Carboxylic acids and derivatives	Amino acids, peptides, and analogues	HMDB0253405	C18864	-0.880333333	0.276792684192684	0.276792684192684	1.02773668175543	unchanged
Meta_382	3-Hydroxyisovalerylcarnitine	Lipids and lipid-like molecules	Fatty Acyls	Fatty acid esters	HMDB0061189		0.05833333333333336	0.89408226284156	0.89408226284156	0.0858529054543429	unchanged
Meta_383	methyl 2-[(4-methyl-2-oxo-2H-chromen-7-yl)oxy]propanoate	Unclassified	Unclassified	Unclassified			-1.373666667	0.148476772283168	0.148476772283168	1.22673923284551	unchanged
Meta_384	Phosphatidylglycerol	Lipids and lipid-like molecules	Glycerophospholipids	Glycerophosphates	HMDB0302468		0.07166666666666672	0.696155323273523	0.696155323273523	0.374194673530297	unchanged
Meta_385	Benzarone	Organic oxygen compounds	Organooxygen compounds	Carbonyl compounds	HMDB0248975	C14474	-0.491333333	0.0570508225108201	0.0570508225108201	1.4334456075824	unchanged
Meta_388	Phenylalanylvaline	Organic acids and derivatives	Carboxylic acids and derivatives	Amino acids, peptides, and analogues	HMDB0029008		-0.697333333	0.405394812756434	0.405394812756434	0.771071963081385	unchanged
Meta_389	Valylphenylalanine	Organic acids and derivatives	Carboxylic acids and derivatives	Amino acids, peptides, and analogues	HMDB0029134		-0.217666667	0.524080014120256	0.524080014120256	0.561648439774968	unchanged
Meta_39	Guaiacol	Benzenoids	Phenols	Methoxyphenols	HMDB0001398	C15572; C01502	0.132666666666669	0.484942053940581	0.484942053940581	0.656413064904403	unchanged
Meta_390	3-(Diethylamino)-1-(3,4-dimethoxyphenyl)propan-1-one	Unclassified	Unclassified	Unclassified			-0.256	0.583740984929561	0.583740984929561	0.472631859507669	unchanged
Meta_392	Inosine	Nucleosides, nucleotides, and analogues	Purine nucleosides	Unclassified	HMDB0000195	C00294	-0.094333333	0.500512785305613	0.500512785305613	0.61238055773117	unchanged
Meta_394	Threonylphenylalanine	Organic acids and derivatives	Carboxylic acids and derivatives	Amino acids, peptides, and analogues	HMDB0029068		-0.406	0.30699690711755	0.30699690711755	0.933756881366201	unchanged
Meta_395	Phenol glucuronide	Organic oxygen compounds	Organooxygen compounds	Carbohydrates and carbohydrate conjugates	HMDB0060014		0.111666666666666	0.628077947593456	0.628077947593456	0.468508769156842	unchanged
Meta_396	3-Oxohexadecanoic acid	Lipids and lipid-like molecules	Fatty Acyls	Fatty acids and conjugates	HMDB0010733		0.298666666666666	0.167292496658228	0.167292496658228	1.18772613138254	unchanged
Meta_397	Pemirolast	Organoheterocyclic compounds	Pyridopyrimidines	Unclassified	HMDB0015023		-0.026666667	0.910990668223022	0.910990668223022	0.117497103802849	unchanged
Meta_398	Octadecylamine	Organic nitrogen compounds	Organonitrogen compounds	Amines	HMDB0029586		-0.016	0.976451805905428	0.976451805905428	0.0651835281442744	unchanged
Meta_399	3-(2-Chlorophenyl)-7-hydroxychromen-2-one	Unclassified	Unclassified	Unclassified			0.161	0.606785591501268	0.606785591501268	0.47480995177496	unchanged
Meta_4	Trifluoromethane	Organohalogen compounds	Alkyl halides	Halomethanes	HMDB0252378		-0.057	0.881742426774809	0.881742426774809	0.118194897436802	unchanged
Meta_40	m-Cresol	Benzenoids	Phenols	Cresols	HMDB0002048	C01467	-0.028666667	0.75985165689292	0.75985165689292	0.252591333120124	unchanged
Meta_400	lysine phosphoester	Organic acids and derivatives	Carboxylic acids and derivatives	Amino acids, peptides, and analogues	HMDB0254265		0.15133333333333	0.326143962515375	0.326143962515375	0.878967268518198	unchanged
Meta_401	2-(4-Fluorophenyl)-3-hydroxy-7-methylchromen-4-one	Unclassified	Unclassified	Unclassified			0.190666666666669	0.814035351371691	0.814035351371691	0.213107692679563	unchanged
Meta_403	2-Hydroxyhexadecanoic acid	Lipids and lipid-like molecules	Fatty Acyls	Fatty acids and conjugates	HMDB0031057		-0.584	0.109344897055913	0.109344897055913	1.34063417744051	unchanged
Meta_404	16-Hydroxyhexadecanoic acid	Lipids and lipid-like molecules	Fatty Acyls	Fatty acids and conjugates	HMDB0006294	C18218	0.594333333333333	0.431543201598159	0.431543201598159	0.709969511028469	unchanged
Meta_405	L-Thyronine	Organic acids and derivatives	Carboxylic acids and derivatives	Amino acids, peptides, and analogues	HMDB0000667		0.873666666666669	0.168256762263075	0.168256762263075	1.11142442439415	unchanged
Meta_406	Hept-3-enoylcarnitine	Lipids and lipid-like molecules	Fatty Acyls	Fatty acid esters	HMDB0241681		-0.437	0.117467840530842	0.117467840530842	1.33846296192612	unchanged
Meta_407	N-(2-hydroxyethyl)tetradecanamide	Unclassified	Unclassified	Unclassified			0.406333333333334	0.316676871275306	0.316676871275306	0.88986046683832	unchanged
Meta_408	beta-naphthoflavone	Phenylpropanoids and polyketides	Flavonoids	Flavones	HMDB0249144		0.162333333333333	0.380543340710941	0.380543340710941	0.83532437471972	unchanged
Meta_409	Imazamethabenz	Organic acids and derivatives	Carboxylic acids and derivatives	Amino acids, peptides, and analogues	HMDB0034885		0.233333333333334	0.217795959022709	0.217795959022709	1.07955308071074	unchanged
Meta_410	3-Hydroxylidocaine	Organic acids and derivatives	Carboxylic acids and derivatives	Amino acids, peptides, and analogues	HMDB0060655	C16560	0.350666666666665	0.243501376173979	0.243501376173979	0.929636975006764	unchanged
Meta_411	p(MeOx1-co-PheOx1-CH3) Na+	Unclassified	Unclassified	Unclassified			0.06066666666666662	0.705688657854185	0.705688657854185	0.312471151757214	unchanged
Meta_412	Lorcaserin sulfamate	Organoheterocyclic compounds	Benzazepines	Unclassified	HMDB0061153		-0.12	0.48812431945658	0.48812431945658	0.647206292751961	unchanged
Meta_413	Cordycepin	Nucleosides, nucleotides, and analogues	Purine nucleosides	Purine 3'-deoxyribonucleosides	HMDB0250451		0.256666666666668	0.1297768125651	0.1297768125651	1.21183709318554	unchanged

Meta_414	Lauryldiethanolamine	Unclassified	Unclassified	Unclassified			0.0276666666666685	0.90150755505826	0.90150755505826	0.114967154584329	unchanged
Meta_416	Bakers yeast extract	Benzenoids	Fluorenes	Unclassified	HMDB0032173		0.100666666666665	0.810589591077768	0.810589591077768	0.211529796993778	unchanged
Meta_417	Cyclobrassinin	Organoheterocyclic compounds	Indoles and derivatives	Indoles	HMDB0033352		-0.136666667	0.390162940201131	0.390162940201131	0.738371677001408	unchanged
Meta_419	4-Formylaminoantipyrine	Organoheterocyclic compounds	Azoles	Pyrazoles	HMDB0246429		-0.129	0.104017417945095	0.104017417945095	1.3807205232778	unchanged
Meta_42	Methylphosphate	Organic acids and derivatives	Organic phosphoric acids and derivatives	Phosphate esters	HMDB0061711		-0.198666667	0.199855791956947	0.199855791956947	1.08772514707525	unchanged
Meta_420	(卤)-Ribaline	Organoheterocyclic compounds	Quinolines and derivatives	Dihydrofuranoquinolines	HMDB0033345		0.529333333333334	0.11525904101066	0.11525904101066	1.35079330196202	unchanged
Meta_421	Fenthion	Organic acids and derivatives	Organic thiophosphoric acids and derivatives	Thiophosphoric acid esters	HMDB0033209	C14420	0.0636666666666663	0.623977659186958	0.623977659186958	0.502028387890452	unchanged
Meta_422	Pyr-phe-OH	Unclassified	Unclassified	Unclassified			1.227	0.201416631089253	0.201416631089253	1.09194455480501	unchanged
Meta_425	Longistylin C	Phenylpropanoids and polyketides	Stilbenes	Unclassified	HMDB0038544		-0.423666667	0.290481656922266	0.290481656922266	0.961933599733079	unchanged
Meta_426	Fexinidazole	Organoheterocyclic compounds	Azoles	Imidazoles	HMDB0252240		0.0500000000000007	0.732983549141131	0.732983549141131	0.355968686956215	unchanged
Meta_428	1-[(2R,3S,5R)-3,4-Dihydroxy-5-(hydroxymethyl)oxolan-2-yl]pyrimidine-2,4-dione	Nucleosides, nucleotides, and analogues	Pyrimidine nucleosides	Unclassified	HMDB0246131		0.0843333333333334	0.448943525082341	0.448943525082341	0.694370308791312	unchanged
Meta_429	(E)-2-(Hydroxymethyl)-3-[(1S,2R,5S)-5-(hydroxymethyl)-2-propan-2-ylcyclohexyl]prop-2-enoic acid	Unclassified	Unclassified	Unclassified			-0.184333333	0.124888969298453	0.124888969298453	1.19425470582188	unchanged
Meta_43	3-Furoic acid	Organoheterocyclic compounds	Furans	Furoic acid and derivatives	HMDB0000444		0.780666666666665	0.0944117291902695	0.0944117291902695	1.24251466536987	unchanged
Meta_431	Phenylalanylleucine	Organic acids and derivatives	Carboxylic acids and derivatives	Amino acids, peptides, and analogues	HMDB0253025		-0.187	0.29158834677353	0.29158834677353	0.871458863491171	unchanged
Meta_432	Leucylphenylalanine	Organic acids and derivatives	Carboxylic acids and derivatives	Amino acids, peptides, and analogues	HMDB0013243		-0.260333333	0.560408513352277	0.560408513352277	0.546178881897176	unchanged
Meta_433	Glycerophosphocholine	Lipids and lipid-like molecules	Glycerophospholipids	Glycerophosphocholines	HMDB0000086	C00670	-0.298666667	0.322176921677622	0.322176921677622	0.954753652420336	unchanged
Meta_436	Hexadecenal	Lipids and lipid-like molecules	Fatty Acyls	Fatty aldehydes	HMDB0060482	C06123	-0.277	0.270044259142698	0.270044259142698	0.966628914423839	unchanged
Meta_438	Methyl 5-(2-oxohexahydro-1H-thieno[3,4-d]imidazol-4-yl)pentanoate	Unclassified	Unclassified	Unclassified			-0.108666667	0.958714107853447	0.958714107853447	0.0105953812004692	unchanged
Meta_439	L-beta-aspartyl-L-phenylalanine	Organic acids and derivatives	Peptidomimetics	Hybrid peptides	HMDB0011167		0.222666666666667	0.206445156237879	0.206445156237879	1.05160173001803	unchanged
Meta_44	Uracil	Organoheterocyclic compounds	Diazines	Pyrimidines and pyrimidine derivatives	HMDB0000300	C00106	-0.211	0.309521651814443	0.309521651814443	0.844325502391666	unchanged
Meta_441	Oleic acid	Lipids and lipid-like molecules	Fatty Acyls	Fatty acids and conjugates	HMDB0000207	C00712	0.183333333333334	0.358784173585305	0.358784173585305	0.791938588267336	unchanged
Meta_442	Hexanoylcarnitine	Lipids and lipid-like molecules	Fatty Acyls	Fatty acid esters	HMDB0000756		-0.065666667	0.818816729946734	0.818816729946734	0.187955042167631	unchanged
Meta_443	N-Demethyltramadol	Benzenoids	Phenol ethers	Anisoles	HMDB0255112		-0.619333333	0.275977297832786	0.275977297832786	0.978431938774954	unchanged
Meta_444	Oleamide	Lipids and lipid-like molecules	Fatty Acyls	Fatty amides	HMDB0002117	C19670	-0.263	0.245715407059407	0.245715407059407	1.06581977876265	unchanged
Meta_446	Myristoylglycine	Organic acids and derivatives	Carboxylic acids and derivatives	Amino acids, peptides, and analogues	HMDB0013250		-0.535333333	0.136174180149898	0.136174180149898	1.15609131869661	unchanged
Meta_447	Altretamine	Organic nitrogen compounds	Organonitrogen compounds	Amines	HMDB0014631		-2.656333333	0.144606232086294	0.144606232086294	1.20510393756172	unchanged
Meta_448	Isovalerylalanine	Organic acids and derivatives	Carboxylic acids and derivatives	Amino acids, peptides, and analogues	HMDB0000747		0.115666666666666	0.165632836992846	0.165632836992846	1.0893858964754	unchanged
Meta_449	N-(2-Hydroxyethyl)pentadecanamide	Unclassified	Unclassified	Unclassified			-0.102	0.517008777701918	0.517008777701918	0.631311359245498	unchanged
Meta_45	2,4-Dimethylthiazole	Organoheterocyclic compounds	Azoles	Thiazoles	HMDB0032974		-0.283666667	0.101277385254844	0.101277385254844	1.23834180785316	unchanged
Meta_450	4'-Methoxymucidin	Benzenoids	Phenol ethers	Anisoles	HMDB0030019		0.573333333333334	0.0542798884169904	0.0542798884169904	1.50167171261854	unchanged
Meta_452	Octanoylcarnitine	Lipids and lipid-like molecules	Fatty Acyls	Fatty acid esters	HMDB0000791	C02838	-0.433666667	0.123591657001141	0.123591657001141	1.28810785588879	unchanged
Meta_453	Lauroyl diethanolamide	Lipids and lipid-like molecules	Fatty Acyls	Fatty amides	HMDB0032358		0.0590000000000011	0.703068520386649	0.703068520386649	0.385449061100046	unchanged
Meta_455	Diazoxon	Organic acids and derivatives	Organic phosphoric acids and derivatives	Phosphate esters	HMDB0251156		0.0250000000000004	0.960582795318974	0.960582795318974	0.0820946845149263	unchanged
Meta_456	N-gamma-Glutamyl-S-(1-propenyl)cysteine	Organic acids and derivatives	Carboxylic acids and derivatives	Amino acids, peptides, and analogues	HMDB0038553		0.801	0.224363437207132	0.224363437207132	1.1010225399713	unchanged
Meta_458	Androsterone	Lipids and lipid-like molecules	Steroids and steroid derivatives	Androstane steroids	HMDB0000031	C00523	-0.079666667	0.821448469987461	0.821448469987461	0.222594024308858	unchanged
Meta_459	Glutamylphenylalanine	Organic acids and derivatives	Carboxylic acids and derivatives	Amino acids, peptides, and analogues	HMDB0029156		0.223333333333333	0.408295442098172	0.408295442098172	0.783816346767987	unchanged
Meta_46	3-Hydroxyproline	Organic acids and derivatives	Carboxylic acids and derivatives	Amino acids, peptides, and analogues	HMDB0245903		-0.254	0.404835491839299	0.404835491839299	0.81742059405203	unchanged
Meta_460	Gingerol	Benzenoids	Phenols	Methoxyphenols	HMDB0005783	C10462	0.0746666666666656	0.772170724935965	0.772170724935965	0.300381980607833	unchanged
Meta_464	gamma-Glutamylphenylalanine	Organic acids and derivatives	Carboxylic acids and derivatives	Amino acids, peptides, and analogues	HMDB0000594		0.151333333333334	0.651757900057619	0.651757900057619	0.457747065975502	unchanged
Meta_465	Isoleucyl-Cysteine	Organic acids and derivatives	Carboxylic acids and derivatives	Amino acids, peptides, and analogues	HMDB0028904		-0.274666667	0.400881220775856	0.400881220775856	0.780420945905692	unchanged
Meta_466	Tyrosyl-Leucine	Organic acids and derivatives	Carboxylic acids and derivatives	Amino acids, peptides, and analogues	HMDB0029109		0.107666666666667	0.641531183745651	0.641531183745651	0.459009986898219	unchanged
Meta_467	2'-O-Methylguanosine	Unclassified	Unclassified	Unclassified			-0.295666667	0.288304667341368	0.288304667341368	0.878345913813647	unchanged
Meta_468	(9Z,12Z)-N-(2-hydroxyethyl)hexadeca-9,12-dienamide	Unclassified	Unclassified	Unclassified			-0.103666667	0.775217549265779	0.775217549265779	0.272611398018894	unchanged
Meta_469	3-Oxooctadecanoic acid	Lipids and lipid-like molecules	Fatty Acyls	Fatty acids and conjugates	HMDB0010736		-0.134	0.816987068208231	0.816987068208231	0.195930497023267	unchanged
Meta_47	Acetylenedicarboxylic acid	Organic acids and derivatives	Carboxylic acids and derivatives	Dicarboxylic acids and derivatives	HMDB0247933	C03248	-0.234	0.156050721663109	0.156050721663109	1.28750873462529	unchanged
Meta_471	5'-Methylthioadenosine	Nucleosides, nucleotides, and analogues	5'-deoxyribonucleosides	5'-deoxy-5'-thionucleosides	HMDB0001173	C00170	0.443666666666665	0.285494966382809	0.285494966382809	0.878558800231754	unchanged
Meta_474	N,N-Dimethyloctadecylamine	Organic nitrogen compounds	Organonitrogen compounds	Amines	HMDB0255266		-0.178666667	0.727622088204758	0.727622088204758	0.35732504890432	unchanged
Meta_475	5,7-Dihydroxy-4'-methoxy-8-methylflavanone	Phenylpropanoids and polyketides	Flavonoids	O-methylated flavonoids	HMDB0041321		0.0106666666666655	0.966650192691709	0.966650192691709	0.0723727730058262	unchanged
Meta_476	12-Hydroxystearic acid	Lipids and lipid-like molecules	Fatty Acyls	Fatty acids and conjugates	HMDB0061706		0.0596666666666668	0.816112055702754	0.816112055702754	0.211007568389009	unchanged
Meta_478	Fludiazepam	Organoheterocyclic compounds	Benzodiazepines	1,4-benzodiazepines	HMDB0015513		0.191666666666666	0.299678705109745	0.299678705109745	0.95689166062045	unchanged
Meta_48	Creatinine	Organic acids and derivatives	Carboxylic acids and derivatives	Amino acids, peptides, and analogues	HMDB0000562	C00791	0.147333333333332	0.208347834365415	0.208347834365415	1.0836755420773	unchanged

Meta_482	(4-oxido-2,3,5,6,7,8-hexahydro-1H-pyrrolizin-4-ium-1-yl)methyl 2-hydroxy-2-(1-hydroxyethyl)-3-methylbutanoate	Unclassified	Unclassified	Unclassified			1.81933333333333	0.197250921485523	0.197250921485523	1.12773344096554	unchanged
Meta_483	4-Hydroxy-3',6-dimethoxy-5'-methylspiro[1-benzouran-2,4'-cyclohex-2-	Unclassified	Unclassified	Unclassified			0.182666666666666	0.408405963262684	0.408405963262684	0.777230270945498	unchanged
Meta_484	N-Acetylaspartylglutamic acid	Organic acids and derivatives	Carboxylic acids and derivatives	Amino acids, peptides, and analogues	HMDB0001067	C12270	0.183666666666667	0.229490917094186	0.229490917094186	0.970423041778978	unchanged
Meta_485	Eicosapentaenoic acid	Lipids and lipid-like molecules	Fatty Acyls	Fatty acids and conjugates	HMDB0001999	C06428	1.03066666666667	0.247232192346708	0.247232192346708	1.09265404960469	unchanged
Meta_487	Benzododecinium	Unclassified	Unclassified	Unclassified			-0.003666667	0.929668319514539	0.929668319514539	0.0704652309671142	unchanged
Meta_488	4-Oxo-1,2,3,4-tetrahydrocyclopenta[c]chromen-7-yl benzoate	Unclassified	Unclassified	Unclassified			-0.217	0.379776289843659	0.379776289843659	0.734381247104507	unchanged
Meta_489	Hexaethylene glycol	Organic oxygen compounds	Organooxygen compounds	Ethers	HMDB0061822		0.318666666666665	0.295325190566061	0.295325190566061	0.888155938716991	unchanged
Meta_490	Glutathione	Organic acids and derivatives	Carboxylic acids and derivatives	Amino acids, peptides, and analogues	HMDB0000125	C00051	0.519333333333332	0.354828734056164	0.354828734056164	0.810403650393843	unchanged
Meta_491	3-Hydroxyimino-10,13-dimethyl-1,2,4,5,6,7,8,9,11,12,14,15,16,17-tetradecahydrocyclopenta[a]phenanthren-17-ol	Unclassified	Unclassified	Unclassified			-0.049	0.821914059430398	0.821914059430398	0.231129479115843	unchanged
Meta_492	Oxidized glutathione	Organic acids and derivatives	Carboxylic acids and derivatives	Amino acids, peptides, and analogues	HMDB0003337	C00127	0.0746666666666656	0.811647901956323	0.811647901956323	0.199968254268629	unchanged
Meta_493	N-Acetylneuraminic acid	Organic oxygen compounds	Organooxygen compounds	Carbohydrates and carbohydrate conjugates	HMDB0000230	C19910	0.0039999999999999956	0.983959417416568	0.983959417416568	0.0176795242056748	unchanged
Meta_494	methyl 2-ethyl-4-[(3R,4R,5S)-5-hydroxy-4,5-dimethyl-2-oxooxolan-3-yl]-2-methyl-3-oxobutanoate	Unclassified	Unclassified	Unclassified			0.225666666666665	0.354649036724991	0.354649036724991	0.859838304774355	unchanged
Meta_495	Melophlin M	Unclassified	Unclassified	Unclassified			0.262333333333332	0.179992194148718	0.179992194148718	1.07789247045963	unchanged
Meta_496	Oleyl alcohol	Lipids and lipid-like molecules	Fatty Acyls	Fatty alcohols	HMDB0029632		-0.274	0.792503042319173	0.792503042319173	0.241143496129277	unchanged
Meta_497	Zaluzanin D	Lipids and lipid-like molecules	Prenol lipids	Terpene lactones	HMDB0302712		0.110999999999999	0.787318569016899	0.787318569016899	0.284178955719939	unchanged
Meta_498	gamma-Glutamyltyrosine	Organic acids and derivatives	Carboxylic acids and derivatives	Amino acids, peptides, and analogues	HMDB0011741		0.375333333333334	0.213154103584232	0.213154103584232	1.05311508149366	unchanged
Meta_499	2-hydroxyethinylestradiol	Lipids and lipid-like molecules	Steroids and steroid derivatives	Estrane steroids	HMDB0061027		-0.634666667	0.112496979417618	0.112496979417618	1.2489216042564	unchanged
Meta_5	ISOPRENE	Hydrocarbons	Unsaturated hydrocarbons	Branched unsaturated hydrocarbons	HMDB0253673	C16521	0.183	0.245064982800167	0.245064982800167	0.970477091817594	unchanged
Meta_500	Mestranol	Lipids and lipid-like molecules	Steroids and steroid derivatives	Estrane steroids	HMDB0015446	C07618	-0.232666667	0.583657198923783	0.583657198923783	0.538903183626823	unchanged
Meta_501	Phenylalanylphenylalanine	Organic acids and derivatives	Carboxylic acids and derivatives	Amino acids, peptides, and analogues	HMDB0013302		0.0583333333333318	0.916862600943908	0.916862600943908	0.0743805666567771	unchanged
Meta_502	3-Palmitoyl-sn-glycerol	Lipids and lipid-like molecules	Glycerolipids	Monoradylglycerols	HMDB0245964		-0.582666667	0.171375915751743	0.171375915751743	1.07236572048741	unchanged
Meta_503	9,10-Dihydroxystearic acid	Lipids and lipid-like molecules	Fatty Acyls	Fatty acids and conjugates	HMDB0302281	C19622	0.199666666666667	0.653939394987367	0.653939394987367	0.41360185946386	unchanged
Meta_504	Phosmet	Organoheterocyclic compounds	Isoindoles and derivatives	Isoindolines	HMDB0256463	C18756	-0.099666667	0.812353957031143	0.812353957031143	0.192018642349788	unchanged
Meta_505	(2Z)-6-Hydroxynon-2-enoylcarnitine	Lipids and lipid-like molecules	Fatty Acyls	Fatty acid esters	HMDB0241750		-0.699333333	0.342949516746276	0.342949516746276	0.793803575008916	unchanged
Meta_506	Val-Val-Val	Organic acids and derivatives	Carboxylic acids and derivatives	Amino acids, peptides, and analogues	HMDB0094676		-0.044	0.869702816663646	0.869702816663646	0.121136726415533	unchanged
Meta_507	Decanoylcarnitine	Lipids and lipid-like molecules	Fatty Acyls	Fatty acid esters	HMDB0000651		-1.316666667	0.0738343701385596	0.0738343701385596	1.45397371675241	unchanged
Meta_509	8-(2,6-Diethyl-4-methylphenyl)-1,2,4,5-tetrahydropyrazolo[1,2-d][1,4,5]oxadiazepine-7,9-dione	Unclassified	Unclassified	Unclassified			0.191000000000001	0.715716540357122	0.715716540357122	0.311427889562147	unchanged
Meta_51	Maleic acid	Organic acids and derivatives	Carboxylic acids and derivatives	Dicarboxylic acids and derivatives	HMDB0000176	C01384	0.0023333333333333619	0.996754956212751	0.996754956212751	0.00541367284370719	unchanged
Meta_510	Cocaethylene	Benzenoids	Benzene and substituted derivatives	Benzoic acids and derivatives	HMDB0041860		0.0646666666666675	0.353581601850758	0.353581601850758	0.807875642624802	unchanged
Meta_511	Leucyl-Tryptophan	Organic acids and derivatives	Carboxylic acids and derivatives	Amino acids, peptides, and analogues	HMDB0028940		0.197333333333333	0.85049315175979	0.85049315175979	0.162829900398403	unchanged
Meta_512	(3Z,6Z,9Z,12Z,15Z)-N-(2-hydroxyethyl)octadeca-3,6,9,12,15-pentaenamide	Unclassified	Unclassified	Unclassified			-0.292	0.366596433266591	0.366596433266591	0.823406313800668	unchanged
Meta_513	Phytosphingosine	Organic nitrogen compounds	Organonitrogen compounds	Amines	HMDB0004610	C12144	0.805666666666667	0.0858361190485762	0.0858361190485762	1.3115442563408	unchanged
Meta_514	2,3-Dihydro-2,3-dihydroxy-4-(4-methoxyphenyl)-1H-phenalen-1-one	Benzenoids	Naphthalenes	Phenylnaphthalenes	HMDB0041463		0.0196666666666658	0.915961375695079	0.915961375695079	0.145026378564281	unchanged
Meta_515	Cryptotanshinone	Lipids and lipid-like molecules	Prenol lipids	Diterpenoids	HMDB0035220		-0.757	0.220279912939874	0.220279912939874	1.11823034042319	unchanged
Meta_516	(5epsilon,9epsilon,16epsilon)-17-Hydroxykauran-19-oic acid	Unclassified	Unclassified	Unclassified			0.766333333333332	0.108105826643909	0.108105826643909	1.24621345254117	unchanged
Meta_517	2-[4-(3,4-Methylenedioxyphenyl)butyl]-4(1H)-quinolinone	Organoheterocyclic compounds	Quinolines and derivatives	Quinolones and derivatives	HMDB0030382		0.0789999999999989	0.496755945362494	0.496755945362494	0.656662045052735	unchanged
Meta_519	5-Thymidylic acid	Nucleosides, nucleotides, and analogues	Pyrimidine nucleotides	Pyrimidine deoxyribonucleotides	HMDB0001227	C00364	0.277000000000001	0.21640509194501	0.21640509194501	1.05847319544995	unchanged
Meta_52	Levulinic acid	Organic acids and derivatives	Keto acids and derivatives	Gamma-keto acids and derivatives	HMDB0000720		0.143000000000001	0.26194663145766	0.26194663145766	0.944806848281126	unchanged
Meta_520	5alpha-Pregnane	Lipids and lipid-like molecules	Steroids and steroid derivatives	Pregnane steroids	HMDB0247017		-0.998666667	0.431339182827883	0.431339182827883	0.718196184333754	unchanged
Meta_521	Uridine 5'-monophosphate	Nucleosides, nucleotides, and analogues	Pyrimidine nucleotides	Pyrimidine ribonucleotides	HMDB0000288	C00105	0.763333333333332	0.184748405914086	0.184748405914086	1.05897194088178	unchanged
Meta_522	Trihexyphenidyl	Organic nitrogen compounds	Organonitrogen compounds	Amines	HMDB0014520	C07171	-0.317666667	0.380317956158877	0.380317956158877	0.783204557918013	unchanged
Meta_523	Ssr180711	Unclassified	Unclassified	Unclassified			0.414333333333333	0.345319707586014	0.345319707586014	0.846832428927671	unchanged
Meta_524	TRIPHENYL PHOSPHATE	Organic acids and derivatives	Organic phosphoric acids and derivatives	Phosphate esters	HMDB0259262	C14235	0.298000000000002	0.454247430627971	0.454247430627971	0.676892215525972	unchanged
Meta_525	Denatonium	Unclassified	Unclassified	Unclassified			-0.117	0.51820602072371	0.51820602072371	0.615096648221783	unchanged
Meta_526	Octadecanamide	Organic acids and derivatives	Carboximidic acids and derivatives	Carboximidic acids	HMDB0034146	C13846	-1.942	0.0979928053106278	0.0979928053106278	1.26596102866206	unchanged

Meta_527	Oleoylethanolamide	Organic nitrogen compounds	Organonitrogen compounds	Amines	HMDB0002088		-0.365	0.245153149176529	0.245153149176529	0.995180908799758	unchanged
Meta_528	(R)-Heraclenol	Phenylpropanoids and polyketides	Coumarins and derivatives	Furanocoumarins	HMDB0030745		0.302000000000003	0.41843702982914	0.41843702982914	0.677917825188042	unchanged
Meta_529	10-(4-Sulfophenyl)decanoic acid	Unclassified	Unclassified	Unclassified			-0.912666667	0.0745969501443396	0.0745969501443396	1.43476560766454	unchanged
Meta_530	Heptaethylene glycol	Organic oxygen compounds	Organooxygen compounds	Ethers	HMDB0061835		0.06033333333333342	0.72463093053109	0.72463093053109	0.360759982460208	unchanged
Meta_531	Avocadyne 4-acetate	Lipids and lipid-like molecules	Fatty Acyls	Fatty alcohols	HMDB0031049		-0.062333333	0.873771891558531	0.873771891558531	0.104063062457792	unchanged
Meta_532	N-(1-Deoxy-1-fructosyl)phenylalanine	Organic acids and derivatives	Carboxylic acids and derivatives	Amino acids, peptides, and analogues	HMDB0037846		0.503666666666668	0.204652554422735	0.204652554422735	1.18704186571544	unchanged
Meta_533	Stearoylethanolamide	Organic nitrogen compounds	Organonitrogen compounds	Amines	HMDB0013078		-0.388	0.385710679725238	0.385710679725238	0.730090364374441	unchanged
Meta_534	Phenylalanyltyrosine	Organic acids and derivatives	Carboxylic acids and derivatives	Amino acids, peptides, and analogues	HMDB0029007		-0.123	0.738938629918721	0.738938629918721	0.313123054629607	unchanged
Meta_535	(15Z)-9,12,13-Trihydroxy-15-octadecenoic acid	Unclassified	Unclassified	Unclassified			1.087	0.112520887038158	0.112520887038158	1.32512715821578	unchanged
Meta_536	Rhodamine 110	Organoheterocyclic compounds	Benzopyrans	1-benzopyrans	HMDB0257198		0.266666666666668	0.12646101795947	0.12646101795947	1.22748289893957	unchanged
Meta_537	Bexarotene	Lipids and lipid-like molecules	Prenol lipids	Retinoids	HMDB0014452		0.243666666666666	0.545991666613652	0.545991666613652	0.518489035358094	unchanged
Meta_538	gamma-Glutamyltryptophan	Organic acids and derivatives	Carboxylic acids and derivatives	Amino acids, peptides, and analogues	HMDB0029160		-0.008666667	0.986188718314608	0.986188718314608	0.00550793961623073	unchanged
Meta_539	2,4,12-Octadecatrienoic acid isobutylamide	Lipids and lipid-like molecules	Fatty Acyls	Fatty amides	HMDB0032033		-0.261666667	0.810088485765285	0.810088485765285	0.220370564137026	unchanged
Meta_541	Pentids	Organic acids and derivatives	Carboxylic acids and derivatives	Amino acids, peptides, and analogues	HMDB0256229		0.433666666666667	0.231315432560889	0.231315432560889	0.978755987594228	unchanged
Meta_542	Pipericine	Lipids and lipid-like molecules	Fatty Acyls	Fatty amides	HMDB0031678		-1.291666667	0.434097038750839	0.434097038750839	0.702145394139054	unchanged
Meta_543	8-Hydroxy-9,10-epoxystearic acid	Unclassified	Unclassified	Unclassified			-0.092666667	0.90409401680068	0.90409401680068	0.139751171935398	unchanged
Meta_544	20-Hydroxyeicosatetraenoic acid	Lipids and lipid-like molecules	Fatty Acyls	Fatty acids and conjugates	HMDB0005998	C14748	-0.401333333	0.184105090338545	0.184105090338545	1.12132732952802	unchanged
Meta_545	13-Docosenamide	Lipids and lipid-like molecules	Fatty Acyls	Fatty amides	HMDB0244507		-0.850333333	0.463098926399606	0.463098926399606	0.664549415897492	unchanged
Meta_546	Fructose 1,6-bisphosphate	Organic oxygen compounds	Organooxygen compounds	Carbohydrates and carbohydrate conjugates	HMDB0001058	C05378	0.434333333333335	0.0540136131959221	0.0540136131959221	1.46901935389839	unchanged
Meta_547	Canrenone	Lipids and lipid-like molecules	Steroids and steroid derivatives	Steroid lactones	HMDB0003033		0.28233333333333	0.842578076308284	0.842578076308284	0.215005392312745	unchanged
Meta_548	12-KETE	Lipids and lipid-like molecules	Fatty Acyls	Fatty acids and conjugates	HMDB0013633	C14807	0.145666666666667	0.866794822136748	0.866794822136748	0.15606603245432	unchanged
Meta_549	Monoelaidin	Lipids and lipid-like molecules	Glycerolipids	Monoradylglycerols	HMDB0254854		-0.416666667	0.103097295896853	0.103097295896853	1.27526384977533	unchanged
Meta_55	3-Hydroxyisovaleric acid	Lipids and lipid-like molecules	Fatty Acyls	Fatty acids and conjugates	HMDB0000754		-0.092666667	0.567811547682836	0.567811547682836	0.478696667401239	unchanged
Meta_550	(3E,5E,8Z)-Deca-3,5,8-trienedioylcarnitine	Lipids and lipid-like molecules	Fatty Acyls	Fatty acid esters	HMDB0241127		0.141666666666668	0.353284630736178	0.353284630736178	0.816806029525015	unchanged
Meta_551	Docosanamide	Lipids and lipid-like molecules	Fatty Acyls	Fatty amides	HMDB0000583		-0.945666667	0.470382009121409	0.470382009121409	0.661167377298927	unchanged
Meta_552	12-[(Cyclohexylcarbamoyl)amino]dodecanoic acid	Unclassified	Unclassified	Unclassified			-0.079333333	0.697877345229562	0.697877345229562	0.35285025908677	unchanged
Meta_554	5-Dodecenoylcarnitine	Lipids and lipid-like molecules	Fatty Acyls	Fatty acid esters	HMDB0241212		-0.330666667	0.142260283856034	0.142260283856034	1.1388720606353	unchanged
Meta_555	[2,2'-Biquinoline]-4,4'-dicarboxylic acid	Organoheterocyclic compounds	Quinolines and derivatives	Quinoline carboxylic acids	HMDB0244480		0.237	0.403596674200462	0.403596674200462	0.692463980340039	unchanged
Meta_557	Aritmina	Alkaloids and derivatives	Ajmaline-sarpagine alkaloids	Unclassified	HMDB0248601		0.309333333333333	0.0994933859128097	0.0994933859128097	1.32267504969123	unchanged
Meta_558	Dodecanoylcarnitine	Lipids and lipid-like molecules	Fatty Acyls	Fatty acid esters	HMDB0002250		-0.335333333	0.118812002876606	0.118812002876606	1.23540912613709	unchanged
Meta_559	3-[2-(2,3-dihydro-1,4-benzodioxin-6-yl)-2-oxoethyl]quinazolin-4(3H)-one	Unclassified	Unclassified	Unclassified			-0.001333333	0.9949388246836	0.9949388246836	0.00335952224087428	unchanged
Meta_56	2-Hydroxy-2-methylbutyric acid	Lipids and lipid-like molecules	Fatty Acyls	Fatty acids and conjugates	HMDB0001987		0.707000000000001	0.0548804503756641	0.0548804503756641	1.3915104409597	unchanged
Meta_560	2-Methoxy-4-vinylphenol	Benzenoids	Phenols	Methoxyphenols	HMDB0013744	C17883	0.584666666666667	0.107846927993942	0.107846927993942	1.26931056208414	unchanged
Meta_561	Tyrosyl-Tyrosine	Organic acids and derivatives	Carboxylic acids and derivatives	Amino acids, peptides, and analogues	HMDB0029117		-0.52	0.373084136790071	0.373084136790071	0.79820032409762	unchanged
Meta_562	Cytidine monophosphate	Nucleosides, nucleotides, and analogues	Pyrimidine nucleotides	Pyrimidine ribonucleotides	HMDB0000095	C00055	0.929666666666666	0.0968328569501491	0.0968328569501491	1.24447385474963	unchanged
Meta_563	Adenosine monophosphate	Nucleosides, nucleotides, and analogues	Purine nucleotides	Purine ribonucleotides	HMDB0000045	C00020	0.0820000000000007	0.753378782814623	0.753378782814623	0.256631654175331	unchanged
Meta_564	(+)-Calycanthine	Organoheterocyclic compounds	Quinolines and derivatives	Aminoquinolines and derivatives	HMDB0029561	C10573	-0.118	0.74953835829296	0.74953835829296	0.285620217857983	unchanged
Meta_565	3(2'-Chlorophenyl)-7-hydroxy-4-phenylcoumarin	Unclassified	Unclassified	Unclassified			-0.299666667	0.480922549074376	0.480922549074376	0.635509971936689	unchanged
Meta_566	hydroquinidine	Alkaloids and derivatives	Cinchona alkaloids	Unclassified	HMDB0242615		0.142666666666667	0.330808997621976	0.330808997621976	0.910946595969129	unchanged
Meta_567	NA-Dopamine 13:0	Unclassified	Unclassified	Unclassified			0.0316666666666663	0.934074428048841	0.934074428048841	0.0495241192337355	unchanged
Meta_568	3-(1H-Indol-3-YL)-2-[2-(2-methylphenoxy)acetamido]propanoic acid	Unclassified	Unclassified	Unclassified			0.304333333333332	0.0855382433982207	0.0855382433982207	1.32407023206196	unchanged
Meta_569	N-(4-fluorophenyl)-2-(4-hydroxyquinazolin-2-ylthio)acetamide CollisionEnergy:102040	Unclassified	Unclassified	Unclassified			0.274333333333335	0.378507976113662	0.378507976113662	0.764674233190454	unchanged
Meta_57	2,5-Dimethyltetrahydrothiophene	Organoheterocyclic compounds	Thiolanes	Unclassified	HMDB0302173		0.0916666666666686	0.309474916510105	0.309474916510105	0.843903798334979	unchanged
Meta_570	Prostaglandin B2	Lipids and lipid-like molecules	Fatty Acyls	Eicosanoids	HMDB0004236	C05954	-0.070333333	0.777533230163483	0.777533230163483	0.300817817540379	unchanged
Meta_571	Dutasteride	Lipids and lipid-like molecules	Steroids and steroid derivatives	Androstane steroids	HMDB0015258		-0.518666667	0.116395359671763	0.116395359671763	1.18394051141895	unchanged
Meta_572	THIODICARB	Organic acids and derivatives	Organic carbonic acids and derivatives	Unclassified	HMDB0259010		0.558666666666667	0.099744626101774	0.099744626101774	1.35637807539516	unchanged
Meta_573	Penicilloic acid	Organic acids and derivatives	Carboxylic acids and derivatives	Amino acids, peptides, and analogues	HMDB0060617		0.152333333333335	0.598241732078157	0.598241732078157	0.53505941785972	unchanged
Meta_574	5,7-Dihydroxy-2-(4-methoxyphenyl)-6-(3-methylbut-2-enyl)-2,3-dihydrochromen-4-one	Unclassified	Unclassified	Unclassified			-0.181333333	0.689618765697321	0.689618765697321	0.344765554553491	unchanged
Meta_575	Phenolsulfonphthalein	Unclassified	Unclassified	Unclassified		C12600	0.211666666666666	0.392053807972382	0.392053807972382	0.82512631756217	unchanged
Meta_576	Gingerenone A	Phenylpropanoids and polyketides	Diarylheptanoids	Linear diarylheptanoids	HMDB0035403	C10460	-0.533	0.306997320090931	0.306997320090931	0.923263678232809	unchanged

Meta_578	Cinacalcet	Benzenoids	Naphthalenes	Unclassified	HMDB0015147		-0.149	0.430222645878087	0.430222645878087	0.718469821085943	unchanged
Meta_579	5,4'-Dihydroxy-3,3'-dimethoxy-6:7-methylenedioxyflavone	Phenylpropanoids and polyketides	Flavonoids	O-methylated flavonoids	HMDB0029272		0.09366666666666675	0.589672315636659	0.589672315636659	0.516580694790977	unchanged
Meta_58	Indole	Organoheterocyclic compounds	Indoles and derivatives	Indoles	HMDB0000738	C00463	0.09966666666666677	0.487099386650076	0.487099386650076	0.619307220610802	unchanged
Meta_580	2-[(3,5-dioxo-2H-1,2,4-triazin-6-yl)amino]-N-[(4-hydroxy-3-methoxyphenyl)methylideneamino]acetamide	Unclassified	Unclassified	Unclassified			0.0240000000000009	0.932124042974582	0.932124042974582	0.109594432428108	unchanged
Meta_581	Pantetheine 4'-phosphate	Organic acids and derivatives	Carboxylic acids and derivatives	Amino acids, peptides, and analogues	HMDB0001416	C01134	0.103666666666667	0.774007412691251	0.774007412691251	0.302796826656508	unchanged
Meta_582	Dopexamine	Benzenoids	Phenols	Benzenediols	HMDB0041882		-0.050666667	0.918184980067652	0.918184980067652	0.0516754667831309	unchanged
Meta_583	Arachidic acid	Lipids and lipid-like molecules	Fatty Acyls	Fatty acids and conjugates	HMDB0002212	C06425	-0.334	0.809434250067218	0.809434250067218	0.262566746288529	unchanged
Meta_584	Tetracosapentaenoic acid (24:5n-6)	Lipids and lipid-like molecules	Fatty Acyls	Fatty acids and conjugates	HMDB0006322		0.03466666666666664	0.811362914576901	0.811362914576901	0.178453594092686	unchanged
Meta_585	Meta-Tyrosine	Organic acids and derivatives	Carboxylic acids and derivatives	Amino acids, peptides, and analogues	HMDB0059720		0.07499999999999993	0.524211739822081	0.524211739822081	0.601097905927893	unchanged
Meta_586	O-Arachidonoyl Glycidol	Unclassified	Unclassified	Unclassified			-0.744	0.0842385998449075	0.0842385998449075	1.31814040572276	unchanged
Meta_587	Guanosine monophosphate	Nucleosides, nucleotides, and analogues	Purine nucleotides	Purine ribonucleotides	HMDB0001397	C00144	0.256333333333334	0.669637376750593	0.669637376750593	0.354461068311209	unchanged
Meta_588	Carbocromen	Phenylpropanoids and polyketides	Coumarins and derivatives	Unclassified	HMDB0249632		-0.037	0.919212491593799	0.919212491593799	0.130468519771298	unchanged
Meta_589	(+)-Butaclamol hydrochloride	Unclassified	Unclassified	Unclassified			-0.057666667	0.879321264086774	0.879321264086774	0.111474072257554	unchanged
Meta_59	Betaine	Organic acids and derivatives	Carboxylic acids and derivatives	Amino acids, peptides, and analogues	HMDB0000043	C00719	0.392000000000003	0.0788037739278329	0.0788037739278329	1.38269298628047	unchanged
Meta_590	9,10-Epoxystearic acid	Lipids and lipid-like molecules	Fatty Acyls	Lineolic acids and derivatives	HMDB0247617	C19418	-0.694333333	0.0678528640962759	0.0678528640962759	1.32453432228635	unchanged
Meta_592	Perfluoroheptanoic acid	Organohalogen compounds	Alkyl halides	Alkyl fluorides	HMDB0256322		0.805	0.387510858766838	0.387510858766838	0.75265318570872	unchanged
Meta_593	(4-Benzylpiperazin-1-yl)(3,5-dimethoxyphenyl)methanone	Unclassified	Unclassified	Unclassified			0.369666666666666	0.118559882699615	0.118559882699615	1.2517440397771	unchanged
Meta_594	Docosanal	Lipids and lipid-like molecules	Fatty Acyls	Fatty aldehydes	HMDB0251560		-0.456	0.647900296735038	0.647900296735038	0.424687863693842	unchanged
Meta_595	Tryptophan 2-C-mannoside	Organoheterocyclic compounds	Indoles and derivatives	Indolyl carboxylic acids and derivatives	HMDB0240296		0.231999999999999	0.25321795574622	0.25321795574622	0.936173698853839	unchanged
Meta_596	N-(3-chlorophenyl)-2-(4-hydroxyquinazolin-2-ylthio)acetamide CollisionEnergy:102040	Unclassified	Unclassified	Unclassified			0.974666666666668	0.149059272056273	0.149059272056273	1.24802994254871	unchanged
Meta_597	3-(3-Methyl-5-pentylfuran-2-yl)propanoylcarnitine	Lipids and lipid-like molecules	Fatty Acyls	Fatty acid esters	HMDB0241844		-0.228666667	0.564835854934493	0.564835854934493	0.551321279571154	unchanged
Meta_598	Methyltricaprylylammonium	Unclassified	Unclassified	Unclassified			-0.164	0.770177529802994	0.770177529802994	0.265024170993859	unchanged
Meta_599	Octadecyl fumarate	Lipids and lipid-like molecules	Fatty Acyls	Fatty acid esters	HMDB0038073		-0.141666667	0.772938228946466	0.772938228946466	0.236889802524044	unchanged
Meta_6	Pyrroline	Organoheterocyclic compounds	Pyrrolines	Unclassified	HMDB0245392		0.04599999999999994	0.698297728136264	0.698297728136264	0.325178614868837	unchanged
Meta_60	L-Valine	Organic acids and derivatives	Carboxylic acids and derivatives	Amino acids, peptides, and analogues	HMDB0000883	C00183	0.652000000000001	0.342773808138045	0.342773808138045	0.82806197596724	unchanged
Meta_600	Cholesterol	Lipids and lipid-like molecules	Steroids and steroid derivatives	Cholestane steroids	HMDB0000067	C00187	-0.143666667	0.208781898195325	0.208781898195325	1.0232054844551	unchanged
Meta_601	Adenosine 2'-phosphate	Organic oxygen compounds	Organooxygen compounds	Carbohydrates and carbohydrate conjugates	HMDB0011617	C00946	0.228999999999999	0.326705794080507	0.326705794080507	0.853645678594519	unchanged
Meta_602	Myristoleoylcarnitine	Lipids and lipid-like molecules	Fatty Acyls	Fatty acid esters	HMDB0240588		-0.203333333	0.257660565400359	0.257660565400359	0.950392048288576	unchanged
Meta_603	Angelicin	Phenylpropanoids and polyketides	Coumarins and derivatives	Furanocoumarins	HMDB0033930	C09060	0.856	0.0560623093910364	0.0560623093910364	1.51864655306684	unchanged
Meta_604	(-)-Arctigenin	Lignans, neolignans and related compounds	Furanoid lignans	Tetrahydrofuran lignans	HMDB0030087		-0.647	0.105728169892669	0.105728169892669	1.33091748586074	unchanged
Meta_605	Docosahexaenoyl Ethanolamide	Organic nitrogen compounds	Organonitrogen compounds	Amines	HMDB0013658		-0.608333333	0.215156749137736	0.215156749137736	0.999271614499683	unchanged
Meta_606	Tetradecanoylcarnitine	Lipids and lipid-like molecules	Fatty Acyls	Fatty acid esters	HMDB0005066		0.02299999999999997	0.947160939149101	0.947160939149101	0.06352107933333	unchanged
Meta_607	(7Z,10Z,13Z,16Z,19Z)-N-(2-Hydroxyethyl)docosa-7,10,13,16,19-pentaenamide	Unclassified	Unclassified	Unclassified			-0.405	0.568356251086496	0.568356251086496	0.515180056860506	unchanged
Meta_608	(10Z,13Z,16Z,19Z)-N-(2-hydroxyethyl)docosa-10,13,16,19-tetraenamide	Unclassified	Unclassified	Unclassified			-0.548	0.500838249683723	0.500838249683723	0.630511446219162	unchanged
Meta_609	Riboflavin	Organoheterocyclic compounds	Pteridines and derivatives	Alloxazines and isoalloxazines	HMDB0000244	C00255	-0.217333333	0.665328447317131	0.665328447317131	0.387702337215685	unchanged
Meta_61	L-Threonine	Organic acids and derivatives	Carboxylic acids and derivatives	Amino acids, peptides, and analogues	HMDB0000167	C00188	0.710666666666665	0.177930372144754	0.177930372144754	1.19432198233855	unchanged
Meta_610	7a-Hydroxy-5b-cholanic acid	Lipids and lipid-like molecules	Steroids and steroid derivatives	Bile acids, alcohols and derivatives	HMDB0002492		0.01333333333333354	0.931830455954524	0.931830455954524	0.0503979760146634	unchanged
Meta_611	S-Lactoylglutathione	Organic acids and derivatives	Carboxylic acids and derivatives	Amino acids, peptides, and analogues	HMDB0001066	C03451	0.550666666666666	0.193592129201581	0.193592129201581	1.03801639165835	unchanged
Meta_612	(10Z,13Z,16Z)-N-(2-hydroxyethyl)docosa-10,13,16-trienamide	Unclassified	Unclassified	Unclassified			-0.741	0.164564183050865	0.164564183050865	1.25393006425804	unchanged
Meta_613	Mangostinone	Organoheterocyclic compounds	Benzopyrans	1-benzopyrans	HMDB0041292		0.191333333333334	0.552790601255634	0.552790601255634	0.578557155466843	unchanged
Meta_614	N-[2-(1H-indol-3-yl)ethyl]-2,2-diphenylcyclopropane-1-carboxamide	Unclassified	Unclassified	Unclassified			0.04466666666666662	0.95960837472237	0.95960837472237	0.0900349154532466	unchanged
Meta_615	Tetraethylene glycol monododecyl ether	Organic oxygen compounds	Organooxygen compounds	Ethers	HMDB0246913		-0.226666667	0.225477843051144	0.225477843051144	0.973691187873854	unchanged
Meta_616	Ochrephilone	Unclassified	Unclassified	Unclassified			-0.345333333	0.499239518362813	0.499239518362813	0.597490453991027	unchanged
Meta_618	2-[[1-(4-Nitrobenzoyl)pyrrolidine-2-carbonyl]amino]benzoic acid	Unclassified	Unclassified	Unclassified			0.539	0.0833519665533463	0.0833519665533463	1.41934351130782	unchanged
Meta_619	(6beta,7alpha,12beta,13beta)-7-Hydroxy-11,16-dioxo-8,14-apianadien-22,6-olide	Lipids and lipid-like molecules	Prenol lipids	Terpene lactones	HMDB0031380		-0.366	0.125872827466669	0.125872827466669	1.30252914442952	unchanged
Meta_62	Phenylacetaldehyde	Benzenoids	Benzene and substituted derivatives	Phenylacetaldehydes	HMDB0006236	C00601	0.591666666666667	0.458046246824667	0.458046246824667	0.746082541604457	unchanged
Meta_620	Corynoxeine	Organoheterocyclic compounds	Indolizidines	Unclassified	HMDB0250480		-0.191666667	0.352265749662917	0.352265749662917	0.844801126690431	unchanged
Meta_621	Cholesta-4,6-dien-3-one	Lipids and lipid-like molecules	Steroids and steroid derivatives	Cholestane steroids	HMDB0002394		1.341	0.103757945504818	0.103757945504818	1.37991931966786	unchanged

Meta_622	Succinyladenosine	Nucleosides, nucleotides, and analogues	Purine nucleosides	Unclassified	HMDB0000912		0.403666666666666	0.207100438443086	0.207100438443086	1.03209376478075	unchanged
Meta_623	(10Z)-Pentadec-10-enoylcarnitine	Lipids and lipid-like molecules	Fatty Acyls	Fatty acid esters	HMDB0241436		1.43433333333333	0.126863585127201	0.126863585127201	1.28239513682602	unchanged
Meta_624	N-Palmitoyl Lysine	Organic acids and derivatives	Carboxylic acids and derivatives	Amino acids, peptides, and analogues	HMDB0241929		-0.059333333	0.882215969958742	0.882215969958742	0.113656846428978	unchanged
Meta_625	Fingolimod phosphate ester, S-	Organic acids and derivatives	Organic phosphoric acids and derivatives	Phosphate esters	HMDB0243705		0.655999999999999	0.316206644548446	0.316206644548446	0.860831000947188	unchanged
Meta_626	Pentadecanoylcarnitine	Lipids and lipid-like molecules	Fatty Acyls	Fatty acid esters	HMDB0062517		0.164333333333335	0.610292264065048	0.610292264065048	0.463246582807064	unchanged
Meta_627	ethyl 2-amino-5-oxo-4-(pyridin-3-yl)-4H,5H-pyrano[3,2-c]chromene-3-carboxylate	Unclassified	Unclassified	Unclassified			0.174	0.622103914985456	0.622103914985456	0.436877112912466	unchanged
Meta_628	Tcmdc-142530	Unclassified	Unclassified	Unclassified			0.0906666666666667 3	0.729202883479095	0.729202883479095	0.328109805007047	unchanged
Meta_629	2-Methoxyethyl 5-hydroxy-2-methyl-1-(3-methylphenyl)benzo[g]indole-3-carboxylate	Unclassified	Unclassified	Unclassified			0.0386666666666667 7	0.885664251703689	0.885664251703689	0.157748462850417	unchanged
Meta_63	2-Methyl-1,4-benzoquinone	Unclassified	Unclassified	Unclassified			-0.045666667	0.641392605804203	0.641392605804203	0.436401848337737	unchanged
Meta_630	3-Hydroxydodec-6-enedioylcarnitine	Lipids and lipid-like molecules	Fatty Acyls	Fatty acid esters	HMDB0241231		0.691000000000001	0.243263717867088	0.243263717867088	0.967088934397163	unchanged
Meta_631	3-[2-(2,3-dihydroxy-5,6,8a-trimethyloctahydro-2H-spiro[naphthalene-1,2'-oxiran]-5-yl)ethyl]-5-hydroxyfuran-2(5H)-one	Unclassified	Unclassified	Unclassified			-0.112	0.295051573188765	0.295051573188765	0.93663386152885	unchanged
Meta_632	1-(3,4-dihydroisoquinolin-2(1H)-yl)-2-(4-iodo-1H-pyrazol-1-yl)ethanone	Unclassified	Unclassified	Unclassified			0.426999999999996	0.12523562898539	0.12523562898539	1.17929549970346	unchanged
Meta_633	Bis(2-ethylhexyl) phthalate	Benzenoids	Benzene and substituted derivatives	Benzoic acids and derivatives	HMDB0249243	C03690	0.462666666666667	0.355623794265768	0.355623794265768	0.836687966507411	unchanged
Meta_634	Deoxycholic acid	Lipids and lipid-like molecules	Steroids and steroid derivatives	Bile acids, alcohols and derivatives	HMDB0000626	C04483	-0.759333333	0.461865835926857	0.461865835926857	0.693599425762016	unchanged
Meta_635	MG(0:0/16:0/0:0)	Lipids and lipid-like molecules	Glycerolipids	Monoradylglycerols	HMDB0011533		-0.021666667	0.920592354854339	0.920592354854339	0.0776946748836336	unchanged
Meta_636	Octaethylene glycol	Organic oxygen compounds	Organooxygen compounds	Ethers	HMDB0094680		0.0820000000000000 7	0.411152951826238	0.411152951826238	0.726981802527123	unchanged
Meta_637	N-Linoleoyl Isoleucine	Unclassified	Unclassified	Unclassified	HMDB0241978		-0.138	0.655658718802688	0.655658718802688	0.42409561496583	unchanged
Meta_638	1-hexadecyl-glycero-3-phosphate	Lipids and lipid-like molecules	Glycerophospholipids	Glycerophosphates	HMDB0062325		-0.217	0.595070487901196	0.595070487901196	0.491617750935759	unchanged
Meta_639	Petromyzonol	Unclassified	Unclassified	Unclassified		C16258	0.0876666666666667 2	0.422853239783601	0.422853239783601	0.727658999665372	unchanged
Meta_64	Niacinamide	Organoheterocyclic compounds	Pyridines and derivatives	Pyridinecarboxylic acids and derivatives	HMDB0001406	C00153	0.0839999999999999 6	0.863585773999428	0.863585773999428	0.148717953959992	unchanged
Meta_640	Kanzonol M	Phenylpropanoids and polyketides	Isoflavonoids	O-methylated isoflavonoids	HMDB0041101		0.0280000000000000 5	0.959294677914258	0.959294677914258	0.0938433148695382	unchanged
Meta_641	Kyotorphin	Organic acids and derivatives	Carboxylic acids and derivatives	Amino acids, peptides, and analogues	HMDB0005768	C02993	-0.097333333	0.782366472365075	0.782366472365075	0.253949197560238	unchanged
Meta_643	9-Hexadecenoylcarnitine	Lipids and lipid-like molecules	Fatty Acyls	Fatty acid esters	HMDB0013207		0.366666666666667	0.129632052991521	0.129632052991521	1.20816852090629	unchanged
Meta_644	S-Adenosylmethionine	Nucleosides, nucleotides, and analogues	5'-deoxyribonucleosides	5'-deoxy-5'-thionucleosides	HMDB0001185		0.144666666666666	0.607791647155697	0.607791647155697	0.467799689723835	unchanged
Meta_645	Palmitoylcarnitine	Lipids and lipid-like molecules	Fatty Acyls	Fatty acid esters	HMDB0000222	C02990	-0.157	0.552049491556985	0.552049491556985	0.549323750572054	unchanged
Meta_646	L-Gulonolactone	Organoheterocyclic compounds	Lactones	Gamma butyrolactones	HMDB0003466	C01040	0.0836666666666667 7	0.772866327282267	0.772866327282267	0.240876520553104	unchanged
Meta_647	7a-Hydroxy-cholestene-3-one	Lipids and lipid-like molecules	Steroids and steroid derivatives	Cholestane steroids	HMDB0001993	C05455	0.421666666666667	0.181056099956252	0.181056099956252	1.09660223639047	unchanged
Meta_648	7-Ketocholesterol	Lipids and lipid-like molecules	Steroids and steroid derivatives	Cholestane steroids	HMDB0000501		-0.137	0.457792407426881	0.457792407426881	0.612316345898617	unchanged
Meta_649	Uridine 5'-diphosphate	Nucleosides, nucleotides, and analogues	Pyrimidine nucleotides	Pyrimidine ribonucleotides	HMDB0000295	C00015	-0.391	0.56476423970062	0.56476423970062	0.564397243546135	unchanged
Meta_65	Taurine	Organic acids and derivatives	Organic sulfonic acids and derivatives	Organosulfonic acids and derivatives	HMDB0000251	C00245	0.122	0.73796092023897	0.73796092023897	0.279149325771126	unchanged
Meta_650	Osajin	Unclassified	Unclassified	Unclassified			0.497333333333335	0.314679178761964	0.314679178761964	0.941041851558912	unchanged
Meta_651	N-[2-(3,4-dihydroxyphenyl)ethyl]heptadecanamide	Unclassified	Unclassified	Unclassified			-0.302333333	0.221398016289164	0.221398016289164	1.09545459642398	unchanged
Meta_652	Isorhynchophylline	Organoheterocyclic compounds	Indolizidines	Unclassified	HMDB0253688		-0.129	0.813691160601695	0.813691160601695	0.240852820947418	unchanged
Meta_653	Heliocide H3	Benzenoids	Anthracenes	Anthraquinones	HMDB0030741		-0.190333333	0.614287920004554	0.614287920004554	0.421210297902175	unchanged
Meta_654	N-Oleoyl phenylalanine	Organic acids and derivatives	Carboxylic acids and derivatives	Amino acids, peptides, and analogues	HMDB0062336		-0.133333333	0.316142964718117	0.316142964718117	0.888881313240834	unchanged
Meta_655	6-Amino-2-(octadec-9-enoylamino)hexanoic acid	Unclassified	Unclassified	Unclassified			0.455666666666666	0.261831616475193	0.261831616475193	1.03171901425448	unchanged
Meta_657	Benzethonium	Unclassified	Unclassified	Unclassified			-0.191666667	0.557221042772719	0.557221042772719	0.526198148455414	unchanged
Meta_658	Echimidine N-oxide	Unclassified	Unclassified	Unclassified			-0.191666667	0.542553352895743	0.542553352895743	0.558840943038706	unchanged
Meta_659	Heptadecanoyl carnitine	Lipids and lipid-like molecules	Fatty Acyls	Fatty acid esters	HMDB0006210		0.0806666666666667 6	0.715040054233124	0.715040054233124	0.365285902770868	unchanged
Meta_66	Nicotinic acid	Organoheterocyclic compounds	Pyridines and derivatives	Pyridinecarboxylic acids and derivatives	HMDB0001488	C00253	-0.184666667	0.479312913112087	0.479312913112087	0.65720683963597	unchanged
Meta_660	Gelomulide N	Unclassified	Unclassified	Unclassified			2.68466666666667	0.263522827720458	0.263522827720458	0.966786333638517	unchanged
Meta_662	N-(3-(dimethylamino)propyl)-2-((3,4,8,8-tetramethyl-2-oxo-2,8,9,10-tetrahydropyrano[2,3-f]chromen-5-yl)oxy)acetamide	Unclassified	Unclassified	Unclassified			0.0626666666666666 9	0.702496499151307	0.702496499151307	0.338603919126946	unchanged
Meta_663	Syringic acid glucuronide	Phenylpropanoids and polyketides	Coumarins and derivatives	Unclassified	HMDB0240562		0.149333333333333	0.243022296941214	0.243022296941214	1.0249484809058	unchanged
Meta_664	1,1,2-Trimethyl-1H-benzo[E]indole	Benzenoids	Naphthalenes	Unclassified	HMDB0246655		0.209	0.136643400715004	0.136643400715004	1.21412379893282	unchanged
Meta_665	LPE O-15:1	Unclassified	Unclassified	Unclassified			-0.024	0.974324469318736	0.974324469318736	0.0086732386637154 3	unchanged
Meta_666	LysoPE(14:0/0:0)	Lipids and lipid-like molecules	Glycerophospholipids	Glycerophosphoethanolamines	HMDB0011500		0.290666666666667	0.256387292531858	0.256387292531858	0.991093979273965	unchanged
Meta_668	Adenosine 3',5'-diphosphate	Nucleosides, nucleotides, and analogues	Purine nucleotides	Purine ribonucleotides	HMDB0000061	C00054	-0.876	0.308612730102278	0.308612730102278	0.940098433239269	unchanged

Meta_669	LPC O-12:0	Unclassified	Unclassified	Unclassified			-0.389	0.156732003119856	0.156732003119856	1.17447719050778	unchanged
Meta_670	Oleoylcarnitine	Lipids and lipid-like molecules	Fatty Acyls	Fatty acid esters	HMDB0005065		0.163666666666668	0.546634151404247	0.546634151404247	0.539721964865521	unchanged
Meta_671	14-Hydroxy-6,10,23-trimethyl-4-azahexacyclo[12.11.0.02,11.04,9.015,24.018,23]pentacosane-17,20-dione	Unclassified	Unclassified	Unclassified			0.297666666666666	0.779016567535703	0.779016567535703	0.256240648418814	unchanged
Meta_672	Stearoylcarnitine	Lipids and lipid-like molecules	Fatty Acyls	Fatty acid esters	HMDB0000848		-0.285333333	0.12922107163977	0.12922107163977	1.22005986296408	unchanged
Meta_673	Derhamnosylmaysin	Phenylpropanoids and polyketides	Flavonoids	Flavonoid glycosides	HMDB0301877		0.0879999999999992	0.641246668622177	0.641246668622177	0.455134662273747	unchanged
Meta_675	Desmosterol	Lipids and lipid-like molecules	Steroids and steroid derivatives	Cholestane steroids	HMDB0002719	C01802	-0.501	0.216638305252079	0.216638305252079	1.04221274845544	unchanged
Meta_676	7-Ketologanin	Unclassified	Unclassified	Unclassified			-0.069333333	0.735097311809651	0.735097311809651	0.265154642652921	unchanged
Meta_677	LPE O-16:2	Unclassified	Unclassified	Unclassified			-0.890333333	0.205866974452383	0.205866974452383	1.00931597681397	unchanged
Meta_678	DG 21:1	Unclassified	Unclassified	Unclassified			-0.032666667	0.835208946818907	0.835208946818907	0.224606673070098	unchanged
Meta_679	LPE O-16:1	Unclassified	Unclassified	Unclassified			-0.424	0.0876027123882821	0.0876027123882821	1.38139571127721	unchanged
Meta_68	Pyroglutamic acid	Organic acids and derivatives	Carboxylic acids and derivatives	Amino acids, peptides, and analogues	HMDB0000267	C01879	-0.087333333	0.448020174443126	0.448020174443126	0.680196056507112	unchanged
Meta_680	Dinonyl phthalate	Benzenoids	Benzene and substituted derivatives	Benzoic acids and derivatives	HMDB0251424		0.267333333333335	0.51220397059338	0.51220397059338	0.616983558189889	unchanged
Meta_681	gamma-Glutamylalanine	Organic acids and derivatives	Carboxylic acids and derivatives	Amino acids, peptides, and analogues	HMDB0006248	C03740	2.29966666666667	0.260806365491743	0.260806365491743	1.01364746064899	unchanged
Meta_682	Nonaethylene glycol	Unclassified	Unclassified	Unclassified			0.0489999999999995	0.841009632325224	0.841009632325224	0.168558644733992	unchanged
Meta_683	Myosmine	Organoheterocyclic compounds	Pyridines and derivatives	Unclassified	HMDB0030386	C10160	-0.026666667	0.956126653785288	0.956126653785288	0.0298306452903793	unchanged
Meta_684	27-Nor-5b-cholestane-3a,7a,12a,24,25-pentol	Lipids and lipid-like molecules	Steroids and steroid derivatives	Bile acids, alcohols and derivatives	HMDB0002126		-0.042	0.912272206377624	0.912272206377624	0.0784560510052116	unchanged
Meta_685	Folic acid	Benzenoids	Phenols	Methoxyphenols	HMDB0000121	C00504	0.268666666666666	0.240573021949838	0.240573021949838	1.05417682330397	unchanged
Meta_686	Cycloart-23-ene-3beta,25-diol	Unclassified	Unclassified	Unclassified			-0.121333333	0.252359786073239	0.252359786073239	0.953348651780921	unchanged
Meta_687	Glycoursodeoxycholic acid	Lipids and lipid-like molecules	Steroids and steroid derivatives	Bile acids, alcohols and derivatives	HMDB0000708		-0.084333333	0.692841703584509	0.692841703584509	0.332230242874344	unchanged
Meta_688	LPE O-17:1	Unclassified	Unclassified	Unclassified			-0.446333333	0.054970678135271	0.054970678135271	1.38199479266884	unchanged
Meta_689	LysoPE(16:1(9Z)/0:0)	Lipids and lipid-like molecules	Glycerophospholipids	Glycerophosphoethanolamines	HMDB0011504		-0.014333333	0.961226747588141	0.961226747588141	0.000640757359770482	unchanged
Meta_69	S-2-Propenyl propanethioate	Organic acids and derivatives	Thiocarboxylic acids and derivatives	Thioesters	HMDB0037493		-0.020333333	0.917611275400163	0.917611275400163	0.053782982437848	unchanged
Meta_690	Peiminine	Lipids and lipid-like molecules	Steroids and steroid derivatives	Steroidal alkaloids	HMDB0256206		-0.419666667	0.122492306764589	0.122492306764589	1.1877707104129	unchanged
Meta_691	1-[1-(4-Methoxyphenyl)-1,3,4,9-tetrahydropyrido[3,4-b]indol-2-yl]-3-phenylprop-2-en-1-one	Unclassified	Unclassified	Unclassified			0.361666666666666	0.128560085051984	0.128560085051984	1.2201633098059	unchanged
Meta_692	Dioctyl phthalate	Benzenoids	Benzene and substituted derivatives	Benzoic acids and derivatives	HMDB0251427	C14227	0.196333333333332	0.769869141965815	0.769869141965815	0.283300402178543	unchanged
Meta_693	LysoPE(16:0/0:0)	Lipids and lipid-like molecules	Glycerophospholipids	Glycerophosphoethanolamines	HMDB0011503		-0.452333333	0.08769099444198	0.08769099444198	1.42592674301786	unchanged
Meta_694	LPC O-14:0	Unclassified	Unclassified	Unclassified			-1.134	0.223504995335394	0.223504995335394	1.07616590052608	unchanged
Meta_695	Flavin mononucleotide	Nucleosides, nucleotides, and analogues	Flavin nucleotides	Unclassified	HMDB0001520	C00061	0.355333333333332	0.305656027542024	0.305656027542024	0.989122102864286	unchanged
Meta_697	Citranaxanthin	Lipids and lipid-like molecules	Prenol lipids	Triterpenoids	HMDB0036883		0.0560000000000009	0.916105499622496	0.916105499622496	0.12918579949243	unchanged
Meta_698	(2R,3S,4S,5R,6R)-2-[[(2S,3R,4R)-3,4-dihydroxy-4-(hydroxymethyl)oxolan-2-yl]oxymethyl]-6-[4-(4-hydroxyphenyl)butan-2-yloxy]oxane-3,4,5-triol	Unclassified	Unclassified	Unclassified			0.438333333333333	0.141515021758447	0.141515021758447	1.18647529986485	unchanged
Meta_699	Adenylosuccinic acid	Nucleosides, nucleotides, and analogues	Purine nucleotides	Purine ribonucleotides	HMDB0248010		0.651	0.143986869284554	0.143986869284554	1.15059129089792	unchanged
Meta_7	2-Methyl-1-butene	Hydrocarbons	Unsaturated hydrocarbons	Branched unsaturated hydrocarbons	HMDB0245210		-0.107666667	0.271029773241547	0.271029773241547	0.965793143678422	unchanged
Meta_70	Ketoleucine	Organic acids and derivatives	Keto acids and derivatives	Short-chain keto acids and derivatives	HMDB0000695	C00233	0.157333333333334	0.475405583805135	0.475405583805135	0.608951842121373	unchanged
Meta_700	LPE O-18:2	Unclassified	Unclassified	Unclassified			-0.351333333	0.199084336437138	0.199084336437138	1.11352956388903	unchanged
Meta_702	LysoPE(P-18:0/0:0)	Lipids and lipid-like molecules	Glycerophospholipids	Glycerophosphoethanolamines	HMDB0240598		-0.726666667	0.187329924107972	0.187329924107972	1.09741482468897	unchanged
Meta_703	LPE O-18:1	Unclassified	Unclassified	Unclassified			-0.295	0.249912187091854	0.249912187091854	1.00760582152892	unchanged
Meta_704	Acetildenafil	Organic oxygen compounds	Organooxygen compounds	Carbonyl compounds	HMDB0247908		-0.613333333	0.126659245898645	0.126659245898645	1.1708695890216	unchanged
Meta_705	LysoSM(d18:1)	Lipids and lipid-like molecules	Sphingolipids	Phosphosphingolipids	HMDB0006482		0.128999999999998	0.466575469238306	0.466575469238306	0.719461426358645	unchanged
Meta_706	LPE O-18:0	Unclassified	Unclassified	Unclassified			-0.589666667	0.0852342413265036	0.0852342413265036	1.42701191416357	unchanged
Meta_707	1-Heptadecanoylglycerophosphoethanolamine	Lipids and lipid-like molecules	Glycerophospholipids	Glycerophosphoethanolamines	HMDB0061691		-0.126333333	0.452997730897075	0.452997730897075	0.65403969832217	unchanged
Meta_708	LysoPC(14:0/0:0)	Lipids and lipid-like molecules	Glycerophospholipids	Glycerophosphocholines	HMDB0010379		0.0253333333333323	0.932908728078259	0.932908728078259	0.111949540788248	unchanged
Meta_710	histamine-C23:1	Unclassified	Unclassified	Unclassified			-0.518	0.206921818773634	0.206921818773634	1.07341187056347	unchanged
Meta_711	Glycitin	Phenylpropanoids and polyketides	Isoflavonoids	Isoflavonoid O-glycosides	HMDB0002219		-0.167333333	0.522252092823946	0.522252092823946	0.614064849478991	unchanged
Meta_712	Withaferin A	Lipids and lipid-like molecules	Steroids and steroid derivatives	Steroid lactones	HMDB0259897	C08841	0.0173333333333332	0.879454299622394	0.879454299622394	0.156616242572011	unchanged
Meta_713	Raloxifene	Organic oxygen compounds	Organooxygen compounds	Carbonyl compounds	HMDB0014624	C07228	1.624	0.0888414336014981	0.0888414336014981	1.39591115991057	unchanged
Meta_714	Nutriacholic acid	Lipids and lipid-like molecules	Steroids and steroid derivatives	Bile acids, alcohols and derivatives	HMDB0000467		-0.352333333	0.456795169772899	0.456795169772899	0.648539941015349	unchanged
Meta_716	1-[1-[4-(Diethylamino)phenyl]-4a-hydroxy-1,3,4,5,6,7,8,8a-octahydroisoquinolin-2-yl]-3-(4-propan-2-ylphenyl)prop-2-en-1-one	Unclassified	Unclassified	Unclassified			-0.368666667	0.151541897116388	0.151541897116388	1.18229232165086	unchanged
Meta_717	N-(3-chloro-4-methoxyphenyl)-2-[4-(morpholin-4-ylcarbonyl)-2-oxoquinolin-1(2H)-yl]acetamide	Unclassified	Unclassified	Unclassified			0.841333333333333	0.342282193484508	0.342282193484508	0.877260314611278	unchanged
Meta_718	LysoPE(18:2(9Z,12Z)/0:0)	Lipids and lipid-like molecules	Glycerophospholipids	Glycerophosphoethanolamines	HMDB0011507		-0.234	0.657833138834727	0.657833138834727	0.365231600993868	unchanged
Meta_719	Crustecdysone	Lipids and lipid-like molecules	Steroids and steroid derivatives	Bile acids, alcohols and derivatives	HMDB0030180	C02633	-0.063	0.332282827608859	0.332282827608859	0.873399918284254	unchanged
Meta_72	QUINUCLIDINE	Organoheterocyclic compounds	Quinuclidines	Unclassified	HMDB0257057		-0.433333333	0.493710676753199	0.493710676753199	0.602220295557598	unchanged
Meta_720	LysoPE(18:1(9Z)/0:0)	Lipids and lipid-like molecules	Glycerophospholipids	Glycerophosphoethanolamines	HMDB0011506		-0.233666667	0.183973724924666	0.183973724924666	1.04437972502521	unchanged

Meta_721	LysoPE(18:0/0:0)	Lipids and lipid-like molecules	Glycerophospholipids	Glycerophosphoethanolamines	HMDB0011130		-0.086333333	0.336490001857238	0.336490001857238	0.86870790783046	unchanged
Meta_722	LysoPC(P-16:0/0:0)	Lipids and lipid-like molecules	Glycerophospholipids	Glycerophosphocholines	HMDB0010407		-0.431333333	0.138109086376981	0.138109086376981	1.14699797305697	unchanged
Meta_723	(E)-6,10-Dimethyl-9-methylene-5-undecen-2-one	Organic oxygen compounds	Organooxygen compounds	Carbonyl compounds	HMDB0035862		-0.317	0.313848927854548	0.313848927854548	0.907759661539685	unchanged
Meta_724	Peldesine	Organoheterocyclic compounds	Pyrrolopyrimidines	Unclassified	HMDB0256208		1.28433333333333	0.148627103295051	0.148627103295051	1.11282919850917	unchanged
Meta_725	17-(Furan-3-yl)-6-hydroxy-4,4,8-trimethyl-3,7-dioxo-14,15-epoxyandrosta-1,5-dien-11-yl acetate	Unclassified	Unclassified	Unclassified			0.374000000000001	0.431962934571496	0.431962934571496	0.687135923045926	unchanged
Meta_726	LPG 16:1	Unclassified	Unclassified	Unclassified			0.845333333333333	0.125201298153601	0.125201298153601	1.25030751260638	unchanged
Meta_727	Decaethylene glycol	Unclassified	Unclassified	Unclassified			-0.069666667	0.667364473857726	0.667364473857726	0.40016859217597	unchanged
Meta_728	LysoPC(15:0/0:0)	Lipids and lipid-like molecules	Glycerophospholipids	Glycerophosphocholines	HMDB0010381	C04230	0.0323333333333338	0.849598626899142	0.849598626899142	0.203138454394128	unchanged
Meta_729	1-O-Hexadecyl-sn-glycero-3-phosphocholine	Lipids and lipid-like molecules	Glycerophospholipids	Glycerophosphocholines	HMDB0243890		-0.663333333	0.0534739534763116	0.0534739534763116	1.46813406733985	unchanged
Meta_730	22-Hydoxy-2-hopen-1-one	Unclassified	Unclassified	Unclassified			-0.126	0.434034467493542	0.434034467493542	0.699634641116563	unchanged
Meta_732	""""""\2-[9-(4-methylphenyl)-6-oxohydropurin-8-ylthio]-N-(2,3,5,6-tetrafluorophenyl)a cetamide CollisionEnergy:205060\""""""""""""""""""""""""""	Unclassified	Unclassified	Unclassified			1.43766666666667	0.191138421315457	0.191138421315457	1.03944903095602	unchanged
Meta_733	Arg-C22:5	Unclassified	Unclassified	Unclassified			0.048	0.589817706386023	0.589817706386023	0.460435495745669	unchanged
Meta_734	LPE O-20:1	Unclassified	Unclassified	Unclassified			0.0086666666666666	0.928408870763745	0.928408870763745	0.108252500789979	unchanged
Meta_735	1-[1-[4-(Diethylamino)phenyl]-4a-hydroxy-1,3,4,5,6,7,8,8a-octahydroisoquinolin-2-yl]-3-(3,4-dimethoxyphenyl)prop-2-en-1-one	Unclassified	Unclassified	Unclassified			-0.658666667	0.331457338259761	0.331457338259761	0.873709641236615	unchanged
Meta_737	LysoPC(16:1(9Z)/0:0)	Lipids and lipid-like molecules	Glycerophospholipids	Glycerophosphocholines	HMDB0010383	C04230	-0.145	0.472814994607751	0.472814994607751	0.599169455499724	unchanged
Meta_738	LPE O-20:0	Unclassified	Unclassified	Unclassified			-0.328333333	0.109417008599348	0.109417008599348	1.24902227320673	unchanged
Meta_739	CerP(d15:0/10:0)	Unclassified	Unclassified	Unclassified			0.205333333333332	0.317019347747976	0.317019347747976	0.967202632032543	unchanged
Meta_74	N-Acetyl-L-alanine	Organic acids and derivatives	Carboxylic acids and derivatives	Amino acids, peptides, and analogues	HMDB0000766		0.482333333333333	0.0500258254152175	0.0500258254152175	1.37881669894339	unchanged
Meta_740	LPC O-17:1	Unclassified	Unclassified	Unclassified			-0.371333333	0.655369272255968	0.655369272255968	0.427092469784473	unchanged
Meta_741	Amppd	Organic acids and derivatives	Organic phosphoric acids and derivatives	Phosphate esters	HMDB0248363		-0.229	0.678859120593766	0.678859120593766	0.357828225172581	unchanged
Meta_743	LysoPC(0:0/16:0)	Lipids and lipid-like molecules	Glycerophospholipids	Glycerophosphocholines	HMDB0240262		-0.63	0.169969375083627	0.169969375083627	1.07644382798373	unchanged
Meta_744	LysoPC(16:0/0:0)	Lipids and lipid-like molecules	Glycerophospholipids	Glycerophosphocholines	HMDB0010382	C04230	-0.273	0.263774951589192	0.263774951589192	0.950131977505137	unchanged
Meta_745	LPC O-17:0	Unclassified	Unclassified	Unclassified			-0.499	0.196899176172846	0.196899176172846	1.07566699785751	unchanged
Meta_746	Lobarin	Unclassified	Unclassified	Unclassified			-0.680666667	0.399588841574793	0.399588841574793	0.725262433165334	unchanged
Meta_747	LysoPE(20:4(8Z,11Z,14Z,17Z)/0:0)	Lipids and lipid-like molecules	Glycerophospholipids	Glycerophosphoethanolamines	HMDB0011518		-0.588333333	0.235699629701048	0.235699629701048	1.00375465923255	unchanged
Meta_749	Methocarbamol	Benzenoids	Phenol ethers	Anisoles	HMDB0014567		-0.072	0.708712638619213	0.708712638619213	0.346775430262738	unchanged
Meta_75	Isoquinoline	Organoheterocyclic compounds	Isoquinolines and derivatives	Unclassified	HMDB0034244	C06323	0.0356666666666658	0.869367546269339	0.869367546269339	0.196082317913805	unchanged
Meta_750	LysoPE(20:1(11Z)/0:0)	Lipids and lipid-like molecules	Glycerophospholipids	Glycerophosphoethanolamines	HMDB0011512		-0.162	0.409554389241165	0.409554389241165	0.689855039978594	unchanged
Meta_751	LPC O-18:2	Unclassified	Unclassified	Unclassified			-0.616333333	0.225232730867394	0.225232730867394	0.991983431756845	unchanged
Meta_752	[(1S,9S,13R,14R)-14-hydroxy-9-(hydroxymethyl)-5,5-dimethyltetracyclo[11.2.1.0,?.0?,?]hexadecan-14-yl]methyl (2E)-3-(3,4-dihydroxyphenyl)prop-2-enoate	Unclassified	Unclassified	Unclassified			0.171999999999999	0.207848281059204	0.207848281059204	1.06391986006909	unchanged
Meta_753	LPC 17:1-SN1	Unclassified	Unclassified	Unclassified			-0.169	0.468285321750199	0.468285321750199	0.635359021398741	unchanged
Meta_754	LPC O-18:1	Unclassified	Unclassified	Unclassified			-0.498333333	0.22911137189657	0.22911137189657	1.0605869728665	unchanged
Meta_755	LysoPC(P-18:0/0:0)	Lipids and lipid-like molecules	Glycerophospholipids	Glycerophosphocholines	HMDB0013122	C04230	0.439666666666666	0.280756940740025	0.280756940740025	1.02926983480745	unchanged
Meta_756	LysoPC(17:0/0:0)	Lipids and lipid-like molecules	Glycerophospholipids	Glycerophosphocholines	HMDB0012108	C04230	0.0510000000000002	0.868073911721715	0.868073911721715	0.189078504591578	unchanged
Meta_757	LysoPC(O-18:0/0:0)	Lipids and lipid-like molecules	Glycerophospholipids	Glycerophosphocholines	HMDB0011149		-0.453	0.0905316860669644	0.0905316860669644	1.3044978687372	unchanged
Meta_758	Betamethasone 17 Valerate	Lipids and lipid-like molecules	Steroids and steroid derivatives	Hydroxysteroids	HMDB0249157		-0.185666667	0.22043300050047	0.22043300050047	1.07290390060012	unchanged
Meta_759	Taurocholic acid	Lipids and lipid-like molecules	Steroids and steroid derivatives	Bile acids, alcohols and derivatives	HMDB0000036	C05122	-0.746666667	0.10882523334254	0.10882523334254	1.27690328952388	unchanged
Meta_760	Butyryl trihexyl citrate	Organic acids and derivatives	Carboxylic acids and derivatives	Tetracarboxylic acids and derivatives	HMDB0247498		0.0683333333333334	0.845513848070995	0.845513848070995	0.185006694995395	unchanged
Meta_761	6-(diaminomethylideneamino)-2-[[(4E,6E,12E,14E)-3-hydroxy-2-(hydroxymethyl)-8,10,16-trimethyloctadeca-4,6,12,14-tetraenoyl]amino]hexanoic acid	Unclassified	Unclassified	Unclassified			-0.131333333	0.467485778313021	0.467485778313021	0.616940842714881	unchanged
Meta_763	LysoPC(18:2(9Z,12Z)/0:0)	Lipids and lipid-like molecules	Glycerophospholipids	Glycerophosphocholines	HMDB0010386	C04100	0.118	0.66688741366653	0.66688741366653	0.439669036135798	unchanged
Meta_764	CAR 25:3	Unclassified	Unclassified	Unclassified			-0.293333333	0.181102603245613	0.181102603245613	1.12034812013401	unchanged
Meta_767	LysoPC(0:0/18:1(9Z))	Lipids and lipid-like molecules	Glycerophospholipids	Glycerophosphocholines	HMDB0061701		-0.026	0.896369998659814	0.896369998659814	0.0927003397736818	unchanged
Meta_768	Tridodecylamine	Organic nitrogen compounds	Organonitrogen compounds	Amines	HMDB0037822		-0.251	0.767360441423756	0.767360441423756	0.30997302777708	unchanged
Meta_77	L-Leucine	Organic acids and derivatives	Carboxylic acids and derivatives	Amino acids, peptides, and analogues	HMDB0000687	C00123	0.268666666666665	0.411516623400988	0.411516623400988	0.801437256926379	unchanged
Meta_770	LPC O-16:1	Unclassified	Unclassified	Unclassified			-0.352333333	0.2702284636416	0.2702284636416	0.986355947122643	unchanged
Meta_772	Medicagenic acid	Lipids and lipid-like molecules	Prenol lipids	Triterpenoids	HMDB0034551		-0.804	0.0576215834027456	0.0576215834027456	1.35132818082086	unchanged
Meta_773	LysoPE(22:6(4Z,7Z,10Z,13Z,16Z,19Z)/0:0)	Lipids and lipid-like molecules	Glycerophospholipids	Glycerophosphoethanolamines	HMDB0011526		-0.557333333	0.251788445803111	0.251788445803111	0.99914330370522	unchanged
Meta_774	LysoPE(22:5(4Z,7Z,10Z,13Z,16Z)/0:0)	Lipids and lipid-like molecules	Glycerophospholipids	Glycerophosphoethanolamines	HMDB0011524		-0.624	0.1805361332276	0.1805361332276	1.07222390700985	unchanged
Meta_775	LPE 22:4	Unclassified	Unclassified	Unclassified			-0.57	0.16144082058918	0.16144082058918	1.22546581627491	unchanged
Meta_776	LPC O-20:5	Unclassified	Unclassified	Unclassified			-1.066	0.071581578047341	0.071581578047341	1.30971608715128	unchanged
Meta_777	LysoPE(22:4(7Z,10Z,13Z,16Z)/0:0)	Lipids and lipid-like molecules	Glycerophospholipids	Glycerophosphoethanolamines	HMDB0011523		-1.046333333	0.248767363753132	0.248767363753132	1.00060814382564	unchanged

Meta_778	LPC O-20:4	Unclassified	Unclassified	Unclassified			-0.388	0.205721666009912	0.205721666009912	1.0630168547988	unchanged
Meta_779	Chenodeoxycholylarginine	Lipids and lipid-like molecules	Steroids and steroid derivatives	Bile acids, alcohols and derivatives	HMDB0242396		-0.082666667	0.496792429620329	0.496792429620329	0.634317503199203	unchanged
Meta_78	Dibutylamine	Organic nitrogen compounds	Organonitrogen compounds	Amines	HMDB0251174		-0.272	0.232183192447485	0.232183192447485	1.09487720987326	unchanged
Meta_781	LPC 19:1-SN1	Unclassified	Unclassified	Unclassified			0.124333333333333	0.699473578655234	0.699473578655234	0.366926212404244	unchanged
Meta_782	LPC O-20:1	Unclassified	Unclassified	Unclassified			0.714333333333334	0.361263786059765	0.361263786059765	0.895911924708309	unchanged
Meta_783	[1-(3,3-Dimethyloxiran-2-yl)-3-[(8S,10S,11S,14R)-11-hydroxy-4,4,8,10,14-pentamethyl-3-oxo-1,2,5,6,7,9,11,12,15,16-decahydrocyclopenta[a]phenanthren-17-yl]butyl] acetate	Lipids and lipid-like molecules	Prenol lipids	Triterpenoids	HMDB0248142		-0.660666667	0.390672095354892	0.390672095354892	0.782602845666527	unchanged
Meta_786	Fellavin; Flacoside	Unclassified	Unclassified	Unclassified			-0.852333333	0.0851692641767471	0.0851692641767471	1.28375269420731	unchanged
Meta_788	LysoPC(0:0/20:4(5Z,8Z,11Z,14Z))	Lipids and lipid-like molecules	Glycerophospholipids	Glycerophosphocholines	HMDB0061699		-0.380333333	0.236328866558362	0.236328866558362	1.01043996063208	unchanged
Meta_79	Ethylmalonic acid	Lipids and lipid-like molecules	Fatty Acyls	Fatty acids and conjugates	HMDB0000622		0.161666666666665	0.497609664668562	0.497609664668562	0.589333320729945	unchanged
Meta_791	Chenodeoxycholylmethionine	Lipids and lipid-like molecules	Steroids and steroid derivatives	Bile acids, alcohols and derivatives	HMDB0242403		-1.220666667	0.0506865494541853	0.0506865494541853	1.4096978331757	unchanged
Meta_792	6-bromo-4-phenyl-3-[3-phenyl-2-(pyridine-3-carbonyl)-3,4-dihydropyrazol-5-yl]-1H-quinolin-2-one	Unclassified	Unclassified	Unclassified			0.839666666666666	0.226924616733381	0.226924616733381	1.09608983780332	unchanged
Meta_794	Cerubidin	Phenylpropanoids and polyketides	Anthracyclines	Unclassified	HMDB0255779		0.212333333333332	0.125295971118433	0.125295971118433	1.21723194944282	unchanged
Meta_796	LysoPC(20:1(11Z)/0:0)	Lipids and lipid-like molecules	Glycerophospholipids	Glycerophosphocholines	HMDB0010391	C04230	0.292333333333334	0.37862928442587	0.37862928442587	0.822064076276438	unchanged
Meta_797	Lyoniside	Lignans, neolignans and related compounds	Lignan glycosides	Unclassified	HMDB0242693		-0.259666667	0.244191962116078	0.244191962116078	1.00558614218908	unchanged
Meta_8	1,3-Butadiene	Hydrocarbons	Unsaturated hydrocarbons	Olefins	HMDB0041792	C16450	0.0836666666666669 4	0.344217793019795	0.344217793019795	0.83308480987679	unchanged
Meta_80	Methylsuccinic acid	Lipids and lipid-like molecules	Fatty Acyls	Fatty acids and conjugates	HMDB0001844		-0.16	0.109161039513371	0.109161039513371	1.29114353475983	unchanged
Meta_800	Octaethyleneglycol monododecyl ether	Organic oxygen compounds	Organooxygen compounds	Ethers	HMDB0246126		-0.000666667	0.995260373388217	0.995260373388217	0.0113951915517143	unchanged
Meta_801	Telmisartan	Benzenoids	Benzene and substituted derivatives	Biphenyls and derivatives	HMDB0015101	C07710	-0.148666667	0.504228311381773	0.504228311381773	0.573296044938068	unchanged
Meta_802	DGCC 8:0_12:0	Unclassified	Unclassified	Unclassified			0.276999999999999	0.86595941206907	0.86595941206907	0.128348002459919	unchanged
Meta_803	SM 8:0;2O/16:2	Unclassified	Unclassified	Unclassified			0.227	0.625994435201118	0.625994435201118	0.47051041420846	unchanged
Meta_805	LysoPC(20:4(5Z,8Z,11Z,14Z)/0:0)	Lipids and lipid-like molecules	Glycerophospholipids	Glycerophosphocholines	HMDB0010395	C04230	0.0776666666666665 7	0.762961540001158	0.762961540001158	0.270188883001187	unchanged
Meta_806	LysoPC(18:1(9Z)/0:0)	Lipids and lipid-like molecules	Glycerophospholipids	Glycerophosphocholines	HMDB0002815		1.66166666666667	0.167960852621389	0.167960852621389	1.23141597784998	unchanged
Meta_807	LPC O-22:0	Unclassified	Unclassified	Unclassified			1.87266666666667	0.0906174390431951	0.0906174390431951	1.45742768127293	unchanged
Meta_81	Cinnamic acid	Phenylpropanoids and polyketides	Cinnamic acids and derivatives	Cinnamic acids	HMDB0000567	C10438	0.0783333333333336 7	0.564963365357483	0.564963365357483	0.549265436620162	unchanged
Meta_810	Inuline	Lipids and lipid-like molecules	Prenol lipids	Diterpenoids	HMDB0248466		-0.068666667	0.786267822572247	0.786267822572247	0.246982551610062	unchanged
Meta_811	MGDG O-13:1_7:0	Unclassified	Unclassified	Unclassified			-0.376333333	0.332836471378294	0.332836471378294	0.882044095632656	unchanged
Meta_812	SM 8:0;2O/17:2	Unclassified	Unclassified	Unclassified			-0.015666667	0.911874845185246	0.911874845185246	0.148955804593689	unchanged
Meta_813	DG(18:0/10:0/0:0)	Lipids and lipid-like molecules	Glycerolipids	Diradylglycerols	HMDB0093890		0.197666666666667	0.227309221962324	0.227309221962324	1.08402043607494	unchanged
Meta_814	LPS 22:1	Unclassified	Unclassified	Unclassified			1.34733333333333	0.0514784007607237	0.0514784007607237	1.49032216601079	unchanged
Meta_818	MGDG O-16:2_5:0	Unclassified	Unclassified	Unclassified			-0.613666667	0.440541110785801	0.440541110785801	0.715368022294192	unchanged
Meta_819	Methyl 3b-acetoxy-2,6-dihydroxy-8a,30a-epoxy-1-oxomeliacate	Unclassified	Unclassified	Unclassified			-0.772666667	0.0516416354154916	0.0516416354154916	1.36643000414963	unchanged
Meta_82	2-Hydroxycaproic acid	Lipids and lipid-like molecules	Fatty Acyls	Fatty acids and conjugates	HMDB0001624		0.253333333333332	0.208397786793469	0.208397786793469	1.14272794012639	unchanged
Meta_821	LysoPC(20:4(8Z,11Z,14Z,17Z)/0:0)	Lipids and lipid-like molecules	Glycerophospholipids	Glycerophosphocholines	HMDB0010396		-0.143	0.492741801273513	0.492741801273513	0.638402389491556	unchanged
Meta_822	Caffeoylcycloartenol	Lipids and lipid-like molecules	Steroids and steroid derivatives	Cycloartanols and derivatives	HMDB0040807		-0.286666667	0.205328431740786	0.205328431740786	1.10391210559923	unchanged
Meta_823	SMGDG O-16:4_2:0	Unclassified	Unclassified	Unclassified			-0.028	0.832618991288874	0.832618991288874	0.163913701727449	unchanged
Meta_824	Polidocanol	Organic oxygen compounds	Organooxygen compounds	Ethers	HMDB0256665	C13493	-0.069	0.545357600867636	0.545357600867636	0.547489688147189	unchanged
Meta_825	DG 34:7	Unclassified	Unclassified	Unclassified			0.413666666666666	0.326329875399227	0.326329875399227	0.877105654559377	unchanged
Meta_826	Uridine diphosphate-N-acetylglucosamine	Nucleosides, nucleotides, and analogues	Pyrimidine nucleotides	Pyrimidine nucleotide sugars	HMDB0000290	C00043	-0.084666667	0.889598476625145	0.889598476625145	0.162300263809803	unchanged
Meta_828	Austrobuxusin I	Unclassified	Unclassified	Unclassified			0.116999999999999	0.641276048094339	0.641276048094339	0.456405296006209	unchanged
Meta_829	DG 35:7	Unclassified	Unclassified	Unclassified			-0.13	0.187910803791915	0.187910803791915	1.17080060933014	unchanged
Meta_83	L-Aspartic acid	Organic acids and derivatives	Carboxylic acids and derivatives	Amino acids, peptides, and analogues	HMDB0000191	C00049	-0.409666667	0.290386899143662	0.290386899143662	0.977666685576261	unchanged
Meta_830	PE-Cer 12:0;2O/20:5	Unclassified	Unclassified	Unclassified			0.0830000000000000 2	0.738916343922062	0.738916343922062	0.331660066107848	unchanged
Meta_831	DG(14:1(9Z)/22:6 (4Z,7Z,10Z,13Z,16Z,19Z)/0:0)	Lipids and lipid-like molecules	Glycerolipids	Diradylglycerols	HMDB0007063		-0.123	0.666335824202041	0.666335824202041	0.437757716747322	unchanged
Meta_832	PC O-26:0	Unclassified	Unclassified	Unclassified			-0.226666667	0.321262089840093	0.321262089840093	0.889555557730406	unchanged
Meta_833	SM(d18:1/12:0)	Lipids and lipid-like molecules	Sphingolipids	Phosphosphingolipids	HMDB0012096	C00550	0.0233333333333333 4	0.886611290887402	0.886611290887402	0.148376689050529	unchanged
Meta_834	PE(14:1(9Z)/15:0)	Lipids and lipid-like molecules	Glycerophospholipids	Glycerophosphoethanolamines	HMDB0008856		-0.034	0.980805645834092	0.980805645834092	0.0575796459434757	unchanged
Meta_835	Dechlorane plus	Organohalogen compounds	Vinyl halides	Vinyl chlorides	HMDB0250920		-0.441666667	0.690367118676581	0.690367118676581	0.374638381533404	unchanged
Meta_836	Gossypetin 8-glucuronide 3-glucoside	Phenylpropanoids and polyketides	Flavonoids	Flavonoid glycosides	HMDB0039103		-0.227333333	0.597579187675364	0.597579187675364	0.481419854779491	unchanged
Meta_837	PE-Cer 12:0;2O/22:1	Unclassified	Unclassified	Unclassified			0.341333333333333	0.411449345393326	0.411449345393326	0.79548565846315	unchanged
Meta_838	PE(14:0/16:0)	Lipids and lipid-like molecules	Glycerophospholipids	Glycerophosphoethanolamines	HMDB0008824		-0.264	0.39578618173488	0.39578618173488	0.734517997363532	unchanged
Meta_839	PC O-28:1	Unclassified	Unclassified	Unclassified			-0.159	0.550015295800828	0.550015295800828	0.514701334104988	unchanged
Meta_84	Creatine	Organic acids and derivatives	Carboxylic acids and derivatives	Amino acids, peptides, and analogues	HMDB0000064	C00300	-0.005	0.964683799553405	0.964683799553405	0.0401138057319564	unchanged

Meta_840	PC O-28:0	Unclassified	Unclassified	Unclassified			0.07766666666666674	0.619194544943376	0.619194544943376	0.439160959798825	unchanged
Meta_841	Cer(d18:1/22:0)	Lipids and lipid-like molecules	Sphingolipids	Ceramides	HMDB0004952	C00195	-1.488333333	0.362563062682883	0.362563062682883	0.854428713878486	unchanged
Meta_842	Haploperozide	Unclassified	Unclassified	Unclassified			0.145333333333333	0.531841776516924	0.531841776516924	0.624548563590949	unchanged
Meta_843	PA(13:0/18:1(12Z)-O(9S,10R))	Unclassified	Unclassified	Unclassified	HMDB0262906		-0.643333333	0.44157073644333	0.44157073644333	0.71163180146769	unchanged
Meta_844	PE O-16:1_16:1	Unclassified	Unclassified	Unclassified			-0.198666667	0.268455446246456	0.268455446246456	0.976971024043862	unchanged
Meta_845	PE O-16:1_16:0	Unclassified	Unclassified	Unclassified			-0.629666667	0.123941069174183	0.123941069174183	1.22650355150891	unchanged
Meta_847	Veratroylzygadenine	Unclassified	Unclassified	Unclassified			-0.482333333	0.347624894216817	0.347624894216817	0.874194830155791	unchanged
Meta_848	PC O-12:0_14:0	Unclassified	Unclassified	Unclassified			0.716666666666667	0.498641813090624	0.498641813090624	0.687173334344038	unchanged
Meta_849	SM(d17:2(4E,8Z)/16:0)	Organic nitrogen compounds	Organonitrogen compounds	Quaternary ammonium salts	HMDB0240677		-0.175666667	0.289092992935814	0.289092992935814	0.877930138431244	unchanged
Meta_85	2-Methylindole	Organoheterocyclic compounds	Indoles and derivatives	Indoles	HMDB0245234		0.106333333333335	0.240774047885436	0.240774047885436	1.07312161973487	unchanged
Meta_850	Dephospho-CoA	Nucleosides, nucleotides, and analogues	Purine nucleotides	Purine ribonucleotides	HMDB0001373	C00882	0.381666666666666	0.12892141844185	0.12892141844185	1.19886741563278	unchanged
Meta_851	LysoPC(28:0/0:0)	Lipids and lipid-like molecules	Glycerophospholipids	Glycerophosphocholines	HMDB0029206		-0.064666667	0.869131544362816	0.869131544362816	0.192219507739337	unchanged
Meta_852	SM(d18:1/15:0)	Lipids and lipid-like molecules	Sphingolipids	Phosphosphingolipids	HMDB0240608		-0.033	0.697617402184894	0.697617402184894	0.343402985871044	unchanged
Meta_853	PC O-30:2	Unclassified	Unclassified	Unclassified			-0.909333333	0.292686216371072	0.292686216371072	0.876598004325603	unchanged
Meta_854	PE-Cer 12:0;2O/24:0	Unclassified	Unclassified	Unclassified			0.132333333333333	0.509291112243158	0.509291112243158	0.582457052602594	unchanged
Meta_855	PC O-30:1	Unclassified	Unclassified	Unclassified			-0.115	0.477263742194015	0.477263742194015	0.66352911511559	unchanged
Meta_856	PC O-30:0	Unclassified	Unclassified	Unclassified			0.000333333333333333741	0.99833997459304	0.99833997459304	0.00668206190578468	unchanged
Meta_857	Cer(d18:1/24:1(15Z))	Lipids and lipid-like molecules	Sphingolipids	Ceramides	HMDB0004953	C00195	-0.861	0.121506227991151	0.121506227991151	1.27071667083687	unchanged
Meta_858	PE O-16:1_18:2	Unclassified	Unclassified	Unclassified			-0.764666667	0.399108007421439	0.399108007421439	0.780880051738122	unchanged
Meta_86	L-Norleucine	Organic acids and derivatives	Carboxylic acids and derivatives	Amino acids, peptides, and analogues	HMDB0001645	C01933	-0.285666667	0.13063847218431	0.13063847218431	1.3280078773848	unchanged
Meta_861	NCGC00385360-01_C36H56O12_beta-D-Glucopyranose, 1-O-[(2alpha,3beta,5xi,9xi,18xi)-2,3,19,24-tetrahydroxy-24,28-dioxoolean-12-en-28-yl]-	Unclassified	Unclassified	Unclassified			-0.699	0.125414017563775	0.125414017563775	1.20150090805558	unchanged
Meta_862	SM 8:1;2O/23:0	Unclassified	Unclassified	Unclassified			0.352333333333334	0.690322654660319	0.690322654660319	0.426068594638268	unchanged
Meta_863	PC(14:0/16:0)	Lipids and lipid-like molecules	Glycerophospholipids	Glycerophosphocholines	HMDB0007869		0.251666666666665	0.0902557272464922	0.0902557272464922	1.40452458177062	unchanged
Meta_864	PC O-31:0	Unclassified	Unclassified	Unclassified			-0.002666667	0.978193727716479	0.978193727716479	0.0247921245971536	unchanged
Meta_865	Cer(d17:1/LTE4)	Unclassified	Unclassified	Unclassified	HMDB0289906		-0.521	0.395220734918068	0.395220734918068	0.807071423768182	unchanged
Meta_866	PC(14:0/14:1(9Z))	Lipids and lipid-like molecules	Glycerophospholipids	Glycerophosphocholines	HMDB0007867		0.203666666666667	0.729463009498667	0.729463009498667	0.292772090918463	unchanged
Meta_867	PC O-12:0_16:0	Unclassified	Unclassified	Unclassified			0.06499999999999995	0.344081842255833	0.344081842255833	0.819504523612258	unchanged
Meta_868	LPC 30:1	Unclassified	Unclassified	Unclassified			-0.36	0.618711695170199	0.618711695170199	0.489404476373196	unchanged
Meta_869	PC O-32:4	Unclassified	Unclassified	Unclassified			-0.143333333	0.54008541956641	0.54008541956641	0.533985696889227	unchanged
Meta_87	3H-1,2-Dithiole-3-thione	Organoheterocyclic compounds	Dithioles	1,2-dithioles	HMDB0035702		0.746000000000002	0.325759770232296	0.325759770232296	0.88849785528188	unchanged
Meta_870	PA(15:0/PGJ2)	Unclassified	Unclassified	Unclassified	HMDB0263194		-0.414333333	0.664870183387992	0.664870183387992	0.435956759534651	unchanged
Meta_871	Parthenocissin A	Lipids and lipid-like molecules	Glycerophospholipids	Glycerophosphocholines	HMDB0304716		-0.774666667	0.183372821610452	0.183372821610452	1.07180107381596	unchanged
Meta_872	PE(16:1(9Z)/18:1(9Z))	Lipids and lipid-like molecules	Glycerophospholipids	Glycerophosphoethanolamines	HMDB0008960		-0.521333333	0.0940567657776209	0.0940567657776209	1.42319954386331	unchanged
Meta_873	PC(14:1(9Z)/P-18:1(9Z))	Lipids and lipid-like molecules	Glycerophospholipids	Glycerophosphocholines	HMDB0007931		-0.082666667	0.769018133510544	0.769018133510544	0.291899794476131	unchanged
Meta_877	PC O-32:2	Unclassified	Unclassified	Unclassified			0.493000000000002	0.133131922306769	0.133131922306769	1.32275231517256	unchanged
Meta_878	SM 8:0;2O/24:2	Unclassified	Unclassified	Unclassified			0.09733333333333333	0.746697158204239	0.746697158204239	0.33899899434524	unchanged
Meta_879	PC O-32:1	Unclassified	Unclassified	Unclassified			-0.147333333	0.127530252124892	0.127530252124892	1.27840008264459	unchanged
Meta_88	Malic acid	Organic acids and derivatives	Hydroxy acids and derivatives	Beta hydroxy acids and derivatives	HMDB0000156	C00711	0.467666666666666	0.155834180478504	0.155834180478504	1.18774190788458	unchanged
Meta_880	SM 8:0;2O/24:1	Unclassified	Unclassified	Unclassified			0.06099999999999999	0.1236872207301	0.1236872207301	1.26548562359348	unchanged
Meta_881	PC 12:0_16:1	Unclassified	Unclassified	Unclassified			0.249333333333333	0.632249289580229	0.632249289580229	0.467836211149608	unchanged
Meta_882	PE O-16:1_20:5	Unclassified	Unclassified	Unclassified			-0.296333333	0.0562629541982519	0.0562629541982519	1.41951147957365	unchanged
Meta_887	PC 30:1	Unclassified	Unclassified	Unclassified			-0.218	0.128288443960509	0.128288443960509	1.36033587704065	unchanged
Meta_888	PC 30:0	Unclassified	Unclassified	Unclassified			-0.061666667	0.617307546999195	0.617307546999195	0.465388904497097	unchanged
Meta_889	LNAPE 16:1/N-19:1	Unclassified	Unclassified	Unclassified			-0.392666667	0.0780559073032574	0.0780559073032574	1.36279712938985	unchanged
Meta_89	3-Methyl sulfolene	Organoheterocyclic compounds	Dihydrothiophenes	Unclassified	HMDB0059667		0.100333333333332	0.467164115374413	0.467164115374413	0.706934126383609	unchanged
Meta_890	PE O-18:2_18:1	Unclassified	Unclassified	Unclassified			-0.874333333	0.0741424372083457	0.0741424372083457	1.48486399083628	unchanged
Meta_891	SM(d18:1/18:1(9Z))	Lipids and lipid-like molecules	Sphingolipids	Phosphosphingolipids	HMDB0012101		0.165999999999999	0.28443053233376	0.28443053233376	0.944683246485369	unchanged
Meta_892	LNAPE 18:1/N-17:0	Unclassified	Unclassified	Unclassified			-0.227666667	0.58636081975586	0.58636081975586	0.459845158567902	unchanged
Meta_893	PC O-33:2	Unclassified	Unclassified	Unclassified			-0.207	0.417150291927799	0.417150291927799	0.723760312849203	unchanged
Meta_894	SM(d18:1/18:0)	Lipids and lipid-like molecules	Sphingolipids	Phosphosphingolipids	HMDB0001348		0.265999999999998	0.0649579177899277	0.0649579177899277	1.43980400949007	unchanged
Meta_895	PS(16:0/16:1(9Z))	Lipids and lipid-like molecules	Glycerophospholipids	Glycerophosphoserines	HMDB0012355		-0.892666667	0.251180458992653	0.251180458992653	0.998139598381644	unchanged
Meta_896	PC(18:1(9Z)/14:0)	Lipids and lipid-like molecules	Glycerophospholipids	Glycerophosphocholines	HMDB0008097		-0.495666667	0.457129035923047	0.457129035923047	0.70221220175353	unchanged
Meta_897	PC O-33:1	Unclassified	Unclassified	Unclassified			-0.212666667	0.172654717365498	0.172654717365498	1.11128501544618	unchanged
Meta_899	PC O-16:1_14:0	Unclassified	Unclassified	Unclassified			-0.003	0.96580043329026	0.96580043329026	0.0620467269014704	unchanged
Meta_9	Pyrrolidine	Organoheterocyclic compounds	Pyrrolidines	Unclassified	HMDB0031641		0.270333333333333	0.0724934216339631	0.0724934216339631	1.35554285366047	unchanged

Meta_900	PC(14:0/18:0)	Lipids and lipid-like molecules	Glycerophospholipids	Glycerophosphocholines	HMDB0007871		0.103666666666665	0.308700851035352	0.308700851035352	0.897708561323102	unchanged
Meta_901	PC O-33:0	Unclassified	Unclassified	Unclassified			-0.682666667	0.135628343955415	0.135628343955415	1.23791731935293	unchanged
Meta_902	PE(16:1(9Z)/20:4(8Z,11Z,14Z,17Z))	Lipids and lipid-like molecules	Glycerophospholipids	Glycerophosphoethanolamines	HMDB0008971		-0.296	0.235521323788185	0.235521323788185	1.09476959149916	unchanged
Meta_903	PC(14:0/15:0)	Lipids and lipid-like molecules	Glycerophospholipids	Glycerophosphocholines	HMDB0007868		0.0120000000000005	0.920547788401834	0.920547788401834	0.112790599852497	unchanged
Meta_904	PS(16:0/16:0)	Lipids and lipid-like molecules	Glycerophospholipids	Glycerophosphoserines	HMDB0000614		-0.596666667	0.547143162108607	0.547143162108607	0.571298357723121	unchanged
Meta_905	PC O-16:0_14:0	Unclassified	Unclassified	Unclassified			-0.007666667	0.915521371532293	0.915521371532293	0.116087966051417	unchanged
Meta_906	PA(20:0/20:2(11Z,14Z))	Lipids and lipid-like molecules	Glycerophospholipids	Glycerophosphates	HMDB0115528		-0.181	0.237932344142113	0.237932344142113	0.997242104956161	unchanged
Meta_907	PE(16:0/20:4(8Z,11Z,14Z,17Z))	Lipids and lipid-like molecules	Glycerophospholipids	Glycerophosphoethanolamines	HMDB0008938		-0.602	0.0883569633779086	0.0883569633779086	1.29621256110134	unchanged
Meta_908	PC(14:0/P-18:1(11Z))	Lipids and lipid-like molecules	Glycerophospholipids	Glycerophosphocholines	HMDB0007897	C00157	0.0210000000000008	0.940382833178793	0.940382833178793	0.0737880640827092	unchanged
Meta_909	PC 31:1	Unclassified	Unclassified	Unclassified			-0.173	0.424688783217096	0.424688783217096	0.761970281898818	unchanged
Meta_91	Thioproline	Organic acids and derivatives	Carboxylic acids and derivatives	Amino acids, peptides, and analogues	HMDB0062164		-0.362	0.677635936883929	0.677635936883929	0.376425312075713	unchanged
Meta_910	PE 34:1	Unclassified	Unclassified	Unclassified			-0.261666667	0.325995290624852	0.325995290624852	0.892420439816327	unchanged
Meta_911	PE(18:1(9Z)/18:2(9Z,12Z))	Lipids and lipid-like molecules	Glycerophospholipids	Glycerophosphoethanolamines	HMDB0009060		-0.403	0.0744407832019468	0.0744407832019468	1.43239777191843	unchanged
Meta_912	PC(P-16:0/18:3(6Z,9Z,12Z))	Lipids and lipid-like molecules	Glycerophospholipids	Glycerophosphocholines	HMDB0011212		-0.110333333	0.768413471875772	0.768413471875772	0.231443627743258	unchanged
Meta_913	PE(18:1(9Z)/18:1(9Z))	Lipids and lipid-like molecules	Glycerophospholipids	Glycerophosphoethanolamines	HMDB0009059		-0.421	0.0571747905608505	0.0571747905608505	1.36095545199096	unchanged
Meta_914	PC(P-16:0/18:2(9Z,12Z))	Lipids and lipid-like molecules	Glycerophospholipids	Glycerophosphocholines	HMDB0011211		-0.188	0.448812008857601	0.448812008857601	0.682366346505239	unchanged
Meta_915	PC O-34:3	Unclassified	Unclassified	Unclassified			-0.070666667	0.82190639607437	0.82190639607437	0.180097444144797	unchanged
Meta_916	GlcCer(d18:1/16:0)	Lipids and lipid-like molecules	Sphingolipids	Glycosphingolipids	HMDB0004971		-0.011666667	0.969541788666359	0.969541788666359	0.00679107435576412	unchanged
Meta_917	PC(16:1(9Z)/P-18:0)	Lipids and lipid-like molecules	Glycerophospholipids	Glycerophosphocholines	HMDB0008028	C00157	-0.216	0.188921208120274	0.188921208120274	1.09548567433818	unchanged
Meta_919	PE O-16:1_22:6	Unclassified	Unclassified	Unclassified			-0.619333333	0.0645321063555394	0.0645321063555394	1.38911179608836	unchanged
Meta_92	Hydroxypropionic acid	Organic acids and derivatives	Hydroxy acids and derivatives	Beta hydroxy acids and derivatives	HMDB0000700	C01013	-0.089333333	0.785978651289535	0.785978651289535	0.285096571979955	unchanged
Meta_920	PE O-18:2_20:5	Unclassified	Unclassified	Unclassified			-0.610666667	0.203158755871573	0.203158755871573	1.04445709938613	unchanged
Meta_921	CerP(d18:1/22:0)	Lipids and lipid-like molecules	Sphingolipids	Phosphosphingolipids	HMDB0010703		1.17033333333333	0.0520361158110253	0.0520361158110253	1.47101938731647	unchanged
Meta_922	SM 8:0;2O/26:1	Unclassified	Unclassified	Unclassified			-0.189	0.0634529837242803	0.0634529837242803	1.32237477652663	unchanged
Meta_923	PC(14:0/16:1(9Z))	Lipids and lipid-like molecules	Glycerophospholipids	Glycerophosphocholines	HMDB0007870		0.240333333333332	0.184324743926525	0.184324743926525	1.14872783898552	unchanged
Meta_924	PC(15:0/P-16:0)	Lipids and lipid-like molecules	Glycerophospholipids	Glycerophosphocholines	HMDB0007961	C00157	-0.098666667	0.833682833189176	0.833682833189176	0.173658655348517	unchanged
Meta_925	SM 8:0;2O/26:0	Unclassified	Unclassified	Unclassified			0.0510000000000019	0.627184841208344	0.627184841208344	0.471134594869099	unchanged
Meta_926	PE O-18:1_20:4	Unclassified	Unclassified	Unclassified			-0.309	0.0611859836043309	0.0611859836043309	1.35540317162333	unchanged
Meta_928	PE O-16:1_22:4	Unclassified	Unclassified	Unclassified			-0.579	0.107596374928116	0.107596374928116	1.36176429140391	unchanged
Meta_929	PC 32:2	Unclassified	Unclassified	Unclassified			-0.006333333	0.987569523152007	0.987569523152007	0.033082065408164	unchanged
Meta_93	Adenine	Organoheterocyclic compounds	Imidazopyrimidines	Purines and purine derivatives	HMDB0000034	C00147	-0.020666667	0.911595843316553	0.911595843316553	0.102330126542366	unchanged
Meta_930	PC 32:1	Unclassified	Unclassified	Unclassified			-0.189333333	0.266913911483723	0.266913911483723	1.0035152284957	unchanged
Meta_931	PC(16:0/18:3(6Z,9Z,12Z))	Lipids and lipid-like molecules	Glycerophospholipids	Glycerophosphocholines	HMDB0007974		-0.536333333	0.157399745352364	0.157399745352364	1.16783403379175	unchanged
Meta_932	PC O-12:0_20:4	Unclassified	Unclassified	Unclassified			0.602	0.330524387932666	0.330524387932666	0.933484796999189	unchanged
Meta_933	PA(15:0/PGF1alpha)	Unclassified	Unclassified	Unclassified	HMDB0263258		-1.342666667	0.412269315542013	0.412269315542013	0.761060075865218	unchanged
Meta_934	PC(16:0/18:2(9Z,12Z))	Lipids and lipid-like molecules	Glycerophospholipids	Glycerophosphocholines	HMDB0007973		-0.004666667	0.965089407068988	0.965089407068988	0.0252604487774631	unchanged
Meta_935	PC O-35:2	Unclassified	Unclassified	Unclassified			-0.313666667	0.551230287382927	0.551230287382927	0.542676522962472	unchanged
Meta_936	PC(O-16:1(9Z)/16:1(9Z))	Lipids and lipid-like molecules	Glycerophospholipids	Glycerophosphocholines	HMDB0013411		-0.027333333	0.769938395986117	0.769938395986117	0.307068316294549	unchanged
Meta_937	PC(16:0/18:1(9Z))	Lipids and lipid-like molecules	Glycerophospholipids	Glycerophosphocholines	HMDB0007972	C13875	-0.149666667	0.431586166755814	0.431586166755814	0.705261595821493	unchanged
Meta_938	SM(d18:1/16:0)	Lipids and lipid-like molecules	Sphingolipids	Phosphosphingolipids	HMDB0010169		0.235333333333333	0.0972390594324936	0.0972390594324936	1.36237641655055	unchanged
Meta_939	PC(15:0/16:1(9Z))	Lipids and lipid-like molecules	Glycerophospholipids	Glycerophosphocholines	HMDB0007936		0	1	1	0.0262712237859584	unchanged
Meta_940	PC O-16:1_16:0	Unclassified	Unclassified	Unclassified			-0.028666667	0.846185273732151	0.846185273732151	0.179239273262921	unchanged
Meta_941	PC O-18:1_14:0	Unclassified	Unclassified	Unclassified			-0.206	0.0722109104048586	0.0722109104048586	1.34702039206107	unchanged
Meta_942	PC 9:0_26:6	Unclassified	Unclassified	Unclassified			-0.448666667	0.156219021170868	0.156219021170868	1.22480618085471	unchanged
Meta_943	PE(18:1(9Z)/20:4(8Z,11Z,14Z,17Z))	Lipids and lipid-like molecules	Glycerophospholipids	Glycerophosphoethanolamines	HMDB0009070		-0.434666667	0.0947871774615661	0.0947871774615661	1.25812009471781	unchanged
Meta_944	PC(15:0/16:0)	Lipids and lipid-like molecules	Glycerophospholipids	Glycerophosphocholines	HMDB0007935		-0.064	0.70782108640353	0.70782108640353	0.348068731024994	unchanged
Meta_945	PC O-16:0_16:0	Unclassified	Unclassified	Unclassified			-0.214666667	0.0550441802105573	0.0550441802105573	1.40939001308304	unchanged
Meta_946	PE(18:0/20:4(8Z,11Z,14Z,17Z))	Lipids and lipid-like molecules	Glycerophospholipids	Glycerophosphoethanolamines	HMDB0009004		-0.400666667	0.0879274352560403	0.0879274352560403	1.33210931121419	unchanged
Meta_947	PC(P-16:0/20:4(5Z,8Z,11Z,14Z))	Lipids and lipid-like molecules	Glycerophospholipids	Glycerophosphocholines	HMDB0011220		-0.058	0.761010125592521	0.761010125592521	0.331495226127014	unchanged
Meta_948	PC(O-16:0/20:5(5Z,8Z,11Z,14Z,17Z))	Lipids and lipid-like molecules	Glycerophospholipids	Glycerophosphocholines	HMDB0039528		0.0926666666666662	0.542406779008382	0.542406779008382	0.496628427479974	unchanged
Meta_949	PC(P-16:0/20:3(5Z,8Z,11Z))	Lipids and lipid-like molecules	Glycerophospholipids	Glycerophosphocholines	HMDB0011218		0.0223333333333322	0.890058938604542	0.890058938604542	0.0987547120816573	unchanged
Meta_95	p-Octopamine	Benzenoids	Phenols	1-hydroxy-2-unsubstituted benzenoids	HMDB0004825	C04227	0.0903333333333336	0.578531517883312	0.578531517883312	0.490704674599273	unchanged
Meta_950	PC O-36:3	Unclassified	Unclassified	Unclassified			-0.022	0.953700023208684	0.953700023208684	0.0964092895401587	unchanged
Meta_952	PC O-36:2	Unclassified	Unclassified	Unclassified			-0.257333333	0.172847626029675	0.172847626029675	1.11685366501826	unchanged
Meta_953	PG(18:1(9Z)/18:1(9Z))	Lipids and lipid-like molecules	Glycerophospholipids	Glycerophosphoglycerols	HMDB0010634		-0.383333333	0.394982977992768	0.394982977992768	0.736338477195668	unchanged
Meta_955	PC 16:0_16:2	Unclassified	Unclassified	Unclassified			-0.271	0.198028423093113	0.198028423093113	1.08889049275042	unchanged
Meta_956	SM 8:0;2O/28:1	Unclassified	Unclassified	Unclassified			0.107333333333333	0.441490404848191	0.441490404848191	0.69977407491843	unchanged
Meta_957	PC(14:0/18:1(9Z))	Lipids and lipid-like molecules	Glycerophospholipids	Glycerophosphocholines	HMDB0007873		-0.111666667	0.377375235422113	0.377375235422113	0.86837312243204	unchanged
Meta_958	PC(16:0/16:1(9Z))	Lipids and lipid-like molecules	Glycerophospholipids	Glycerophosphocholines	HMDB0007969		-0.324333333	0.104290442017018	0.104290442017018	1.32415832187347	unchanged
Meta_959	PC(P-16:0/18:1(12Z)-2OH(9,10))	Unclassified	Unclassified	Unclassified	HMDB0289479		-0.159666667	0.629222536013859	0.629222536013859	0.412731668896066	unchanged
Meta_96	Hypoxanthine	Organoheterocyclic compounds	Imidazopyrimidines	Purines and purine derivatives	HMDB0000157	C00262	-0.378	0.378747392181942	0.378747392181942	0.805466298371728	unchanged
Meta_960	PC O-16:0_17:1	Unclassified	Unclassified	Unclassified			-0.294666667	0.0851898144357111	0.0851898144357111	1.28955833591702	unchanged
Meta_961	SM 8:0;2O/28:0	Unclassified	Unclassified	Unclassified			-0.004	0.990701123957679	0.990701123957679	0.0248813793982961	unchanged
Meta_962	PC 34:3	Unclassified	Unclassified	Unclassified			0.430999999999999	0.224502987662332	0.224502987662332	0.998588848842649	unchanged
Meta_963	PC(16:0/16:0)	Lipids and lipid-like molecules	Glycerophospholipids	Glycerophosphocholines	HMDB0000564		0.163	0.388582240225028	0.388582240225028	0.813878543006877	unchanged

Meta_964	PC 34:2	Unclassified	Unclassified	Unclassified			0.268000000000001	0.493200274378759	0.493200274378759	0.642365284124757	unchanged
Meta_965	PC O-37:4	Unclassified	Unclassified	Unclassified			-0.382	0.523451486715565	0.523451486715565	0.591846804689493	unchanged
Meta_966	FAD	Nucleosides, nucleotides, and analogues	Flavin nucleotides	Unclassified	HMDB0001248	C00016	0.104666666666667	0.546519062963153	0.546519062963153	0.5950232464499	unchanged
Meta_967	PC 34:0	Unclassified	Unclassified	Unclassified			-0.148333333	0.693922576004274	0.693922576004274	0.351385468878116	unchanged
Meta_968	PC(O-16:1(9Z)/18:2(9Z,12Z))	Lipids and lipid-like molecules	Glycerophospholipids	Glycerophosphocholines	HMDB0013413		-0.502	0.189338815925166	0.189338815925166	1.11423841456239	unchanged
Meta_969	PC(18:0/18:2(9Z,12Z))	Lipids and lipid-like molecules	Glycerophospholipids	Glycerophosphocholines	HMDB0008039		-0.146666667	0.506687317590602	0.506687317590602	0.624712842953336	unchanged
Meta_970	PC 16:1_17:1	Unclassified	Unclassified	Unclassified			-0.123	0.863647836496529	0.863647836496529	0.131035561483114	unchanged
Meta_971	PS(18:0/18:1(9Z))	Lipids and lipid-like molecules	Glycerophospholipids	Glycerophosphoserines	HMDB0010163		-0.265666667	0.535494778604791	0.535494778604791	0.580457876757248	unchanged
Meta_972	PC(O-16:0/18:2(9Z,12Z))	Lipids and lipid-like molecules	Glycerophospholipids	Glycerophosphocholines	HMDB0011151		0.397666666666666	0.380410576697448	0.380410576697448	0.803526070930482	unchanged
Meta_974	PC 16:0_17:1	Unclassified	Unclassified	Unclassified			-0.503	0.107990594172529	0.107990594172529	1.26158655649367	unchanged
Meta_975	PC O-38:7	Unclassified	Unclassified	Unclassified			0.128666666666668	0.497895958275505	0.497895958275505	0.556566763787803	unchanged
Meta_976	PC O-16:0_18:1	Unclassified	Unclassified	Unclassified			-0.182666667	0.158190550757144	0.158190550757144	1.20085991967003	unchanged
Meta_977	PE(20:1(11Z)/20:4(8Z,11Z,14Z,17Z))	Lipids and lipid-like molecules	Glycerophospholipids	Glycerophosphoethanolamines	HMDB0009268		-0.406333333	0.247660517273713	0.247660517273713	1.01624209282737	unchanged
Meta_978	PC 16:0_17:0	Unclassified	Unclassified	Unclassified			-0.132333333	0.307416108994811	0.307416108994811	0.888734390433934	unchanged
Meta_979	PC O-38:6	Unclassified	Unclassified	Unclassified			0.193666666666665	0.646376455055808	0.646376455055808	0.380947775001746	unchanged
Meta_98	4-Nitrophenol	Benzenoids	Phenols	Nitrophenols	HMDB0001232	C00870	0.0499999999999998 9	0.896291340619006	0.896291340619006	0.104571603634583	unchanged
Meta_980	PC(P-16:0/22:4(7Z,10Z,13Z,16Z))	Lipids and lipid-like molecules	Glycerophospholipids	Glycerophosphocholines	HMDB0011226		-0.314333333	0.0614178797811043	0.0614178797811043	1.41695630207276	unchanged
Meta_981	PC O-38:5	Unclassified	Unclassified	Unclassified			0.0986666666666666 5	0.699153353513229	0.699153353513229	0.327026212152416	unchanged
Meta_982	PC O-38:4	Unclassified	Unclassified	Unclassified			-0.386333333	0.468207471606429	0.468207471606429	0.614575576290199	unchanged
Meta_983	PC(14:0/20:4(8Z,11Z,14Z,17Z))	Lipids and lipid-like molecules	Glycerophospholipids	Glycerophosphocholines	HMDB0007884		-0.534333333	0.0856064762782003	0.0856064762782003	1.31913928873077	unchanged
Meta_984	PC(16:1(9Z)/18:2(9Z,12Z))	Lipids and lipid-like molecules	Glycerophospholipids	Glycerophosphocholines	HMDB0008006		0.143333333333334	0.537936733163507	0.537936733163507	0.600083221672276	unchanged
Meta_985	PC 36:5	Unclassified	Unclassified	Unclassified			-0.386	0.17662648306421	0.17662648306421	1.06955618780344	unchanged
Meta_986	PC 36:3	Unclassified	Unclassified	Unclassified			-0.008666667	0.976562700655463	0.976562700655463	0.0255961911283984	unchanged
Meta_987	PC(16:0/18:0)	Lipids and lipid-like molecules	Glycerophospholipids	Glycerophosphocholines	HMDB0007970		0.0423333333333333 6	0.869277870384978	0.869277870384978	0.191018364951536	unchanged
Meta_988	PS(18:1(9Z)/18:1(9Z))	Lipids and lipid-like molecules	Glycerophospholipids	Glycerophosphoserines	HMDB0012390		1.08133333333333	0.0998508696683312	0.0998508696683312	1.30725489945221	unchanged
Meta_989	PC 36:2	Unclassified	Unclassified	Unclassified			0.104333333333337	0.384879041880907	0.384879041880907	0.827725994288741	unchanged
Meta_99	2,6-Dimethylbenzenethiol	Benzenoids	Thiophenols	Unclassified	HMDB0032019		0.166	0.458179333751562	0.458179333751562	0.65630353178932	unchanged
Meta_990	PC O-16:1_20:4	Unclassified	Unclassified	Unclassified			0.0809999999999999 5	0.304654515407815	0.304654515407815	0.835603735397847	unchanged
Meta_991	PC 36:1	Unclassified	Unclassified	Unclassified			0.425000000000001	0.278209745162823	0.278209745162823	0.951994232449455	unchanged
Meta_992	PC(O-16:0/20:4(8Z,11Z,14Z,17Z))	Lipids and lipid-like molecules	Glycerophospholipids	Glycerophosphocholines	HMDB0013407		-0.202333333	0.0928783271477495	0.0928783271477495	1.40618583271066	unchanged
Meta_993	PC 17:1_18:2	Unclassified	Unclassified	Unclassified			0.308333333333334	0.487002078988922	0.487002078988922	0.587531560247381	unchanged
Meta_994	PC O-18:2_18:1	Unclassified	Unclassified	Unclassified			-0.336	0.30866905184704	0.30866905184704	0.910018289574325	unchanged
Meta_995	PC(O-18:1(9Z)/18:2(9Z,12Z))	Lipids and lipid-like molecules	Glycerophospholipids	Glycerophosphocholines	HMDB0013429		-0.097666667	0.440368238019746	0.440368238019746	0.697277427533747	unchanged
Meta_996	PC 17:1_18:1	Unclassified	Unclassified	Unclassified			-0.101333333	0.372501917722377	0.372501917722377	0.792174466261493	unchanged
Meta_997	PC(P-18:1(11Z)/22:6(4Z,7Z,10Z,13Z,16Z,19Z))	Lipids and lipid-like molecules	Glycerophospholipids	Glycerophosphocholines	HMDB0011295		0.0296666666666667 4	0.918368477698581	0.918368477698581	0.0494763563359368	unchanged
Meta_998	PC O-40:8	Unclassified	Unclassified	Unclassified			0.0213333333333332 8	0.94785115972583	0.94785115972583	0.0459381877173074	unchanged
Meta_999	PC O-18:1_18:1	Unclassified	Unclassified	Unclassified			-0.047666667	0.788270589111591	0.788270589111591	0.284157602815339	unchanged
Meta_1014	PC(18:1(9Z)/18:1(9Z))	Lipids and lipid-like molecules	Glycerophospholipids	Glycerophosphocholines	HMDB0000593		0.297666666666665	0.0367950259279602	0.0367950259279602	1.41503237915873	up
Meta_106	Glucal	Organic oxygen compounds	Organooxygen compounds	Alcohols and polyols	HMDB0252763		0.368333333333334	0.0135847934797339	0.0135847934797339	1.55256071521225	up
Meta_122	L-Histidine	Organic acids and derivatives	Carboxylic acids and derivatives	Amino acids, peptides, and analogues	HMDB0000177	C00135	0.290000000000003	0.0129467741690886	0.0129467741690886	1.60993335274696	up
Meta_132	trans-S-(1-Propenyl)-L-cysteine	Organic acids and derivatives	Carboxylic acids and derivatives	Amino acids, peptides, and analogues	HMDB0029440		0.735999999999999	0.0256704201001688	0.0256704201001688	1.569357417	up
Meta_149	L-2,3-Dihydrodipicolinate	Organic acids and derivatives	Carboxylic acids and derivatives	Amino acids, peptides, and analogues	HMDB0012247	C03340	0.362666666666669	0.0473436742988139	0.0473436742988139	1.4495653292796	up
Meta_157	cis-Aconitic acid	Organic acids and derivatives	Carboxylic acids and derivatives	Tricarboxylic acids and derivatives	HMDB0000072	C00417	0.497666666666666	0.0434626894393383	0.0434626894393383	1.46344807462973	up
Meta_164	4-Methylumbelliferone	Phenylpropanoids and polyketides	Coumarins and derivatives	Hydroxycoumarins	HMDB0059622	C03081	0.452000000000002	0.0328171673570414	0.0328171673570414	1.52482629877083	up
Meta_165	L-Arginine	Organic acids and derivatives	Carboxylic acids and derivatives	Amino acids, peptides, and analogues	HMDB0000517	C00062	1.72866666666667	0.00718358462241495	0.00718358462241495	1.66158851430952	up
Meta_176	Hydroxyphenyllactic acid	Phenylpropanoids and polyketides	Phenylpropanoic acids	Unclassified	HMDB0000755	C03672	0.297333333333334	0.0117579100708491	0.0117579100708491	1.56593528557716	up
Meta_192	N-Lactoylvaline	Organic acids and derivatives	Carboxylic acids and derivatives	Amino acids, peptides, and analogues	HMDB0062181		0.566000000000001	0.0178940283009039	0.0178940283009039	1.57814568566588	up
Meta_194	N8-Acetylspermidine	Organic acids and derivatives	Carboximidic acids and derivatives	Carboximidic acids	HMDB0002189	C01029	1.954	0.0077233458430574	0.0077233458430574	1.66280444634722	up
Meta_200	2-Butylbenzothiazole	Organoheterocyclic compounds	Benzothiazoles	Unclassified	HMDB0040031		0.610333333333333	0.00907223968182823	0.00907223968182823	1.62951937312161	up
Meta_219	VASICINONE	Organoheterocyclic compounds	Diazanaphthalenes	Benzodiazines	HMDB0259770		0.785333333333334	0.0120397584272977	0.0120397584272977	1.58074528622513	up
Meta_22	Putrescine	Organic nitrogen compounds	Organonitrogen compounds	Amines	HMDB0001414	C00134	0.428666666666665	0.0160497672929229	0.0160497672929229	1.53766835282836	up
Meta_225	3-Indolebutyric acid	Organoheterocyclic compounds	Indoles and derivatives	Indoles	HMDB0002096	C11284	0.526333333333334	0.0142360825948962	0.0142360825948962	1.56459046957603	up

Meta_231	N-Acetyl-L-phenylalanine	Organic acids and derivatives	Carboxylic acids and derivatives	Amino acids, peptides, and analogues	HMDB0000512	C03519	0.509666666666668	0.00396093672554258	0.00396093672554258	1.63411231283737	up
Meta_238	Atipamezole	Benzenoids	Indanes	Unclassified	HMDB0248699		0.555	0.0421695497806763	0.0421695497806763	1.46764712484859	up
Meta_25	Toluene	Benzenoids	Benzene and substituted derivatives	Toluenes	HMDB0034168	C01455	0.150333333333332	0.0485412335988892	0.0485412335988892	1.42414312879913	up
Meta_257	1-Isothiocyanato-7-(methylsulfinyl)heptane	Organosulfur compounds	Sulfoxides	Unclassified	HMDB0038441		0.672999999999998	0.0329341494901409	0.0329341494901409	1.46069769619364	up
Meta_259	2-Hexylbenzothiazole	Organoheterocyclic compounds	Benzothiazoles	Unclassified	HMDB0040032		0.739333333333335	0.00957184037664051	0.00957184037664051	1.59873968860309	up
Meta_260	Bisphenol F	Benzenoids	Benzene and substituted derivatives	Diphenylmethanes	HMDB0240711	C14298	1.37533333333333	0.0335346228044257	0.0335346228044257	1.5072852689279	up
Meta_291	Pro-leu	Organic acids and derivatives	Carboxylic acids and derivatives	Amino acids, peptides, and analogues	HMDB0253028		1.934	0.04772006393394	0.04772006393394	1.53366745637062	up
Meta_293	N1,N8-Diacetylspermidine	Organic acids and derivatives	Carboxylic acids and derivatives	Carboxylic acid derivatives	HMDB0041947		3.49333333333333	1.79652420501638e-05	1.79652420501638e-05	1.70581625861213	up
Meta_302	1-Methoxypyrene	Benzenoids	Pyrenes	Unclassified	HMDB0060324	C18259	1.07466666666667	0.00975562946895078	0.00975562946895078	1.61833254371159	up
Meta_307	Salsoline-1-carboxylate	Organoheterocyclic compounds	Tetrahydroisoquinolines	Unclassified	HMDB0013067		0.370333333333335	0.0312921201065935	0.0312921201065935	1.47870948054411	up
Meta_320	Bisphenol B	Benzenoids	Benzene and substituted derivatives	Diphenylmethanes	HMDB0245327	C14225	1.035	0.00025195534117418	0.00025195534117418	1.69235979100319	up
Meta_341	Aspartyl-Leucine	Organic acids and derivatives	Carboxylic acids and derivatives	Amino acids, peptides, and analogues	HMDB0028757		0.990000000000002	0.00866135637531473	0.00866135637531473	1.64403094758105	up
Meta_342	8-hydroxy-3,6,9-trimethyl-3a,4,5,9b-tetrahydro-3H-benzo[g][1]benzofuran-2-one	Unclassified	Unclassified	Unclassified			0.391	0.0448598197135628	0.0448598197135628	1.4211705776581	up
Meta_371	Orbencarb	Unclassified	Unclassified	Unclassified		C11087	0.355666666666666	0.0264236133646833	0.0264236133646833	1.50989950391277	up
Meta_375	Cyclohexanones	Organic nitrogen compounds	Organonitrogen compounds	Amines	HMDB0302411		0.459333333333333	0.0392723673541612	0.0392723673541612	1.44249604445111	up
Meta_386	Thiamine	Organoheterocyclic compounds	Diazines	Pyrimidines and pyrimidine derivatives	HMDB0000235	C00378	1.266	0.00850778968471322	0.00850778968471322	1.66633087853254	up
Meta_387	Unii-73hdp58zfk	Unclassified	Unclassified	Unclassified			0.796333333333333	0.0051580401653192	0.0051580401653192	1.67480187807246	up
Meta_41	2-Acetyloxazole	Organic oxygen compounds	Organooxygen compounds	Carbonyl compounds	HMDB0038175		0.700999999999999	0.0076641944948765	0.0076641944948765	1.60098372850039	up
Meta_415	N-lactoyl-Tryptophan	Organic acids and derivatives	Carboxylic acids and derivatives	Amino acids, peptides, and analogues	HMDB0062178		0.735666666666667	0.0112998567354838	0.0112998567354838	1.63474604835811	up
Meta_418	methyl N-(3,4-dimethoxybenzoyl)glycinate	Unclassified	Unclassified	Unclassified			0.335666666666667	0.027962056301876	0.027962056301876	1.58884582564653	up
Meta_424	Monoethylhexyl phthalic acid	Benzenoids	Benzene and substituted derivatives	Benzoic acids and derivatives	HMDB0013248	C03343	0.402666666666665	0.0480903329580101	0.0480903329580101	1.51779377921546	up
Meta_430	Dibutyl phthalate	Benzenoids	Benzene and substituted derivatives	Benzoic acids and derivatives	HMDB0033244	C14214	0.651666666666666	0.0395802509493699	0.0395802509493699	1.46501725978218	up
Meta_451	N1,N12-Diacetylspermine	Organic acids and derivatives	Carboximidic acids and derivatives	Carboximidic acids	HMDB0002172	C03413	2.953	0.000223082176741015	0.000223082176741015	1.69279316750949	up
Meta_457	2-methoxy-6-methyl-5-(phenylmethoxy)-2H-3,4,5,6-tetrahydropyran-3,4-diol	Unclassified	Unclassified	Unclassified			0.933333333333332	0.0486354707812562	0.0486354707812562	1.47646667920714	up
Meta_470	Ricinoleic acid	Lipids and lipid-like molecules	Fatty Acyls	Fatty acids and conjugates	HMDB0034297	C08365	0.667666666666667	0.0395630205079024	0.0395630205079024	1.43024532972434	up
Meta_477	2-Pyrrolidineacetic acid	Organoheterocyclic compounds	Pyrrolidines	Unclassified	HMDB0029444		2.31166666666667	0.0149722622046254	0.0149722622046254	1.64261273803156	up
Meta_480	Arginylglutamine	Organic acids and derivatives	Carboxylic acids and derivatives	Amino acids, peptides, and analogues	HMDB0028707		0.757333333333333	0.0212172024618371	0.0212172024618371	1.59458685993336	up
Meta_50	1-Piperidinecarboxaldehyde	Organoheterocyclic compounds	Piperidines	Unclassified	HMDB0031702		3.177	0.0306325958152956	0.0306325958152956	1.58395918844998	up
Meta_518	Chloroquine	Organoheterocyclic compounds	Quinolines and derivatives	Aminoquinolines and derivatives	HMDB0014746	C07625	9.56766666666667	0.00039524541948243	0.00039524541948243	1.68011309902307	up
Meta_53	L-Proline	Organic acids and derivatives	Carboxylic acids and derivatives	Amino acids, peptides, and analogues	HMDB0000162	C00148	0.426666666666669	0.0105644925093831	0.0105644925093831	1.56680504559191	up
Meta_54	Methylmalonic acid	Organic acids and derivatives	Carboxylic acids and derivatives	Dicarboxylic acids and derivatives	HMDB0000202	C02170	0.389333333333333	0.0499001739488116	0.0499001739488116	1.46770687691794	up
Meta_540	Nivalenol	Lipids and lipid-like molecules	Prenol lipids	Sesquiterpenoids	HMDB0004304		0.670333333333332	0.0367277199659733	0.0367277199659733	1.46791208966683	up
Meta_553	Diprotin A	Organoheterocyclic compounds	Pyrroles	Substituted pyrroles	HMDB0251473		3.63866666666667	0.027685228746868	0.027685228746868	1.54312252098637	up
Meta_556	Aceprometazine	Organoheterocyclic compounds	Benzothiazines	Phenothiazines	HMDB0015553		1.83366666666667	0.00627530966464359	0.00627530966464359	1.64451615457956	up
Meta_591	MG(i-17:0/0:0/0:0)	Lipids and lipid-like molecules	Glycerolipids	Monoradylglycerols	HMDB0072846		1.245	0.0444693036283981	0.0444693036283981	1.41727716116542	up
Meta_617	Reichsteins Substance S	Lipids and lipid-like molecules	Steroids and steroid derivatives	Hydroxysteroids	HMDB0257139		0.361666666666666	0.0415975078783266	0.0415975078783266	1.55362288863035	up
Meta_642	N-Docosahexaenoyl Alanine	Organic acids and derivatives	Carboxylic acids and derivatives	Amino acids, peptides, and analogues	HMDB0242007		2.14866666666667	1.71323999699969e-05	1.71323999699969e-05	1.70214062417447	up
Meta_661	Tomatidine	Lipids and lipid-like molecules	Steroids and steroid derivatives	Steroidal alkaloids	HMDB0034731	C10826	1.80533333333333	0.000588228524263552	0.000588228524263552	1.69150236556649	up
Meta_667	O-Linoleoylcarnitine	Lipids and lipid-like molecules	Fatty Acyls	Fatty acid esters	HMDB0240780		1.112	0.0352345490506257	0.0352345490506257	1.44944073552915	up
Meta_674	Ingenol-3,4:5,20-diacetonide	Unclassified	Unclassified	Unclassified			1.515	0.0415829072068573	0.0415829072068573	1.4873510735794	up
Meta_696	10,14-Dihydroxy-11-methoxy-3,3-dimethyl-12-(3-methyl-2-butenyl)-[1]benzopyrano[5,6-b]xanthen-13(3H)-one	Organoheterocyclic compounds	Benzopyrans	1-benzopyrans	HMDB0304630		0.342666666666666	0.0392209284999056	0.0392209284999056	1.50527647281135	up
Meta_715	Folinic acid	Organoheterocyclic compounds	Pteridines and derivatives	Pterins and derivatives	HMDB0001562	C03479	1.14933333333333	0.00469288046979715	0.00469288046979715	1.64136768135554	up
Meta_73	N-Acetyl-beta-alanine	Organic acids and derivatives	Carboxylic acids and derivatives	Carboxylic acids	HMDB0061880	C01073	0.425333333333331	0.0274967786474381	0.0274967786474381	1.6192933534159	up
Meta_736	LPS 16:1	Unclassified	Unclassified	Unclassified			3.21066666666667	1.15266695023003e-05	1.15266695023003e-05	1.70582995026809	up
Meta_742	LysoPS(16:0/0:0)	Lipids and lipid-like molecules	Glycerophospholipids	Glycerophosphoserines	HMDB0240605		2.765	9.74222999864593e-05	9.74222999864593e-05	1.70161782620838	up
Meta_76	Pipecolic acid	Organic acids and derivatives	Carboxylic acids and derivatives	Amino acids, peptides, and analogues	HMDB0000070	C00408	0.245333333333335	0.0145879517499403	0.0145879517499403	1.56312156498714	up
Meta_766	1-Oleoylglycerophosphoserine	Lipids and lipid-like molecules	Glycerophospholipids	Glycerophosphoserines	HMDB0061694		2.00033333333334	0.0136886078362079	0.0136886078362079	1.63955976150476	up
Meta_769	1-Stearoylglycerophosphoserine	Lipids and lipid-like molecules	Glycerophospholipids	Glycerophosphoserines	HMDB0061698		1.78033333333333	0.00376618596290903	0.00376618596290903	1.6808015449702	up
Meta_771	LysoPC(0:0/18:0)	Lipids and lipid-like molecules	Glycerophospholipids	Glycerophosphocholines	HMDB0011128		1.28933333333334	0.00303539112060014	0.00303539112060014	1.67714513853168	up
Meta_780	LysoPE(22:1(13Z)/0:0)	Lipids and lipid-like molecules	Glycerophospholipids	Glycerophosphoethanolamines	HMDB0011521		0.447333333333335	0.0332910372198414	0.0332910372198414	1.46497027748747	up
Meta_784	LPC O-20:0	Unclassified	Unclassified	Unclassified			0.950000000000001	0.0367128644924915	0.0367128644924915	1.49136850370164	up
Meta_785	Cer(d18:1/16:0)	Lipids and lipid-like molecules	Sphingolipids	Ceramides	HMDB0004949	C00195	1.233	0.0427952388860117	0.0427952388860117	1.55691709938104	up
Meta_787	Glycocholic acid 3-sulfate	Lipids and lipid-like molecules	Steroids and steroid derivatives	Bile acids, alcohols and derivatives	HMDB0240728		3.58433333333333	0.0354390663399517	0.0354390663399517	1.59270409724425	up

Meta_789	homoharringtonine	Alkaloids and derivatives	Cephalotaxus alkaloids	Unclassified	HMDB0253207		2.96666666666667	0.00140192122053848	0.00140192122053848	1.69648870081153	up
Meta_790	LPS 20:3	Unclassified	Unclassified	Unclassified			1.804	0.00069095217097935	0.00069095217097935	1.67332472185451	up
Meta_793	Ergosine	Alkaloids and derivatives	Ergoline and derivatives	Lysergic acids and derivatives	HMDB0251913	C09167	1.94933333333333	0.000123996779791924	0.000123996779791924	1.69945351771169	up
Meta_795	LPS 20:1	Unclassified	Unclassified	Unclassified			3.01366666666667	0.00741538946540627	0.00741538946540627	1.68812082364016	up
Meta_798	LysoPC(20:0/0:0)	Lipids and lipid-like molecules	Glycerophospholipids	Glycerophosphocholines	HMDB0010390	C04230	1.98933333333333	0.0399858086801871	0.0399858086801871	1.57865858546637	up
Meta_799	Cer(d18:1/14:0)	Lipids and lipid-like molecules	Sphingolipids	Ceramides	HMDB0011773	C13916	1.61333333333334	0.00039551117640085	0.00039551117640085	1.68716975863375	up
Meta_804	Uridine diphosphate glucose	Nucleosides, nucleotides, and analogues	Pyrimidine nucleotides	Pyrimidine nucleotide sugars	HMDB0000286	C00029	0.532666666666666	0.00800958951036915	0.00800958951036915	1.6753958598409	up
Meta_808	LysoPC(18:0/0:0)	Lipids and lipid-like molecules	Glycerophospholipids	Glycerophosphocholines	HMDB0010384		1.15533333333334	0.00949177386771233	0.00949177386771233	1.67304064726039	up
Meta_809	Cer(d17:1/16:0)	Lipids and lipid-like molecules	Sphingolipids	Ceramides	HMDB0240685		1.54333333333333	0.00369910480906599	0.00369910480906599	1.63246669161124	up
Meta_815	LysoPC(22:1(13Z)/0:0)	Lipids and lipid-like molecules	Glycerophospholipids	Glycerophosphocholines	HMDB0010399		1.2	0.0241900302069271	0.0241900302069271	1.59750326390103	up
Meta_816	Uridine diphosphate glucuronic acid	Organoheterocyclic compounds	Lactones	Gamma butyrolactones	HMDB0000935	C00167	0.358333333333334	0.0169548341268308	0.0169548341268308	1.54875367176933	up
Meta_817	N-[(4E,8E)-1,3-dihydroxyoctadeca-4,8-dien-2-yl]hexadecanamide	Lipids and lipid-like molecules	Sphingolipids	Ceramides	HMDB0035480		1.14466666666667	0.00994019037886292	0.00994019037886292	1.66522542039847	up
Meta_820	Cer(d18:0/16:0)	Lipids and lipid-like molecules	Sphingolipids	Ceramides	HMDB0011760		2.02366666666667	0.00153227014930332	0.00153227014930332	1.69429641860066	up
Meta_827	Cer(d18:1/18:0)	Lipids and lipid-like molecules	Sphingolipids	Ceramides	HMDB0004950	C00195	2.29433333333333	0.0100253441281949	0.0100253441281949	1.65317030659589	up
Meta_876	HexCer 8:0;2O/24:1	Unclassified	Unclassified	Unclassified			1.73266666666667	0.0156944070424801	0.0156944070424801	1.59436793850113	up
Meta_883	SM 8:0;2O/24:0	Unclassified	Unclassified	Unclassified			0.562999999999999	0.023327362791386	0.023327362791386	1.52025731998324	up
Meta_90	2-Deoxy-D-ribose	Organic oxygen compounds	Organooxygen compounds	Carbonyl compounds	HMDB0245099	C01801	0.646333333333333	0.027860181499512	0.027860181499512	1.59823779321593	up
Meta_954	PC(16:1(9Z)/16:1(9Z))	Lipids and lipid-like molecules	Glycerophospholipids	Glycerophosphocholines	HMDB0008002		0.343666666666667	0.0389888803393933	0.0389888803393933	1.44428698557624	up
Meta_97	1-Methylnicotinamide	Organoheterocyclic compounds	Pyridines and derivatives	Pyridinecarboxylic acids and derivatives	HMDB0000699	C02918	1.373	0.000471983678751542	0.000471983678751542	1.68489278431413	up

Table S2. The metabolites in metabolomics analysis (MLT v.s. Ctrl)

Alignment ID	Metabolite name	HMDB_SuperClass	HMDB_Class	HMDB_SubClass	HMDB ID	cpd_id	log2fc	p.value	fdr	VIP	regulation
Meta_1011	PC 16:0_20:3	Unclassified	Unclassified	Unclassified			-0.758666667	0.0376049616778897	0.0376049616778897	1.26298692744225	down
Meta_1033	PC(18:1(9Z)/20:2(11Z,14Z))	Lipids and lipid-like molecules	Glycerophospholipids	Glycerophosphocholines	HMDB0008111		-0.463333333	0.00529148630714817	0.00529148630714817	1.45133595446147	down
Meta_1034	SM 8:0;2O/34:2	Unclassified	Unclassified	Unclassified			-0.791666667	0.00712076484927852	0.00712076484927852	1.39165381836366	down
Meta_110	Acetylcholine	Organic nitrogen compounds	Organonitrogen compounds	Quaternary ammonium salts	HMDB0000895	C01996	-0.433666667	0.00565354904393133	0.00565354904393133	1.40503458446996	down
Meta_117	Xanthine	Organoheterocyclic compounds	Imidazopyrimidines	Purines and purine derivatives	HMDB0000292	C00385	-0.829333333	0.0342419316418084	0.0342419316418084	1.33242125402033	down
Meta_134	L-Carnitine	Organic nitrogen compounds	Organonitrogen compounds	Quaternary ammonium salts	HMDB0000062	C00318	-0.705	0.00444884194265141	0.00444884194265141	1.43365164457845	down
Meta_139	Phosphohydroxypyruvic acid	Organic oxygen compounds	Organooxygen compounds	Carbonyl compounds	HMDB0001024	C03232	-0.268666667	0.0286405707071591	0.0286405707071591	1.36191837278046	down
Meta_141	1-Methylxanthine	Organoheterocyclic compounds	Imidazopyrimidines	Purines and purine derivatives	HMDB0010738	C16358	-0.898	0.0157146863275244	0.0157146863275244	1.3684695352886	down
Meta_146	p-Hydroxymandelic acid	Benzenoids	Phenols	1-hydroxy-2-unsubstituted benzenoids	HMDB0000822	C11527	-1.350666667	0.0183861451517578	0.0183861451517578	1.39488278971255	down
Meta_16	4-Hydroxy-2-butenoic acid gamma-lactone	Organoheterocyclic compounds	Dihydrofurans	Furanones	HMDB0032330	C17601	-0.494	0.0291617493376825	0.0291617493376825	1.26706718709156	down
Meta_162	4-(Dimethylamino)butyl carbamimidothioate	Organosulfur compounds	Isothioureas	Unclassified	HMDB0246305		-0.128666667	0.0364967513968892	0.0364967513968892	1.27750379379214	down
Meta_170	Methylthio 2-(propanoyloxy)propanoate	Organic acids and derivatives	Thiocarboxylic acids and derivatives	Thioesters	HMDB0040003		-0.361333333	0.0455475078668429	0.0455475078668429	1.26189348906012	down
Meta_174	Glycerol pyruvate	Lipids and lipid-like molecules	Glycerolipids	Monoradylglycerols	HMDB0252845		-1.456666667	0.036115036632524	0.036115036632524	1.34847320482325	down
Meta_186	Isoplumbagin	Benzenoids	Naphthalenes	Naphthoquinones	HMDB0035291		-0.970666667	0.0201926176290575	0.0201926176290575	1.38028338499832	down
Meta_19	L-Alanine	Organic acids and derivatives	Carboxylic acids and derivatives	Amino acids, peptides, and analogues	HMDB0000161	C00041	-0.465666667	0.0193192594272695	0.0193192594272695	1.32414481413836	down
Meta_195	4',5'-Dihydropsoralen	Phenylpropanoids and polyketides	Coumarins and derivatives	Furanocoumarins	HMDB0302123		-1.557666667	0.00337365107612963	0.00337365107612963	1.42698353121772	down
Meta_196	Leucyl-Glycine	Organic acids and derivatives	Carboxylic acids and derivatives	Amino acids, peptides, and analogues	HMDB0028929		-0.787333333	0.0206611996280402	0.0206611996280402	1.38868819570522	down
Meta_212	Caffeine	Organoheterocyclic compounds	Imidazopyrimidines	Purines and purine derivatives	HMDB0001847		-0.365333333	0.00436358014675236	0.00436358014675236	1.45666579380519	down
Meta_240	Dodecatrienoic acid	Lipids and lipid-like molecules	Fatty Acyls	Fatty acids and conjugates	HMDB0302622		-0.693666667	0.0200567626969067	0.0200567626969067	1.37149640890406	down
Meta_245	N-Nonanoylglycine	Organic acids and derivatives	Carboxylic acids and derivatives	Amino acids, peptides, and analogues	HMDB0013279		-0.486	0.0181079643830694	0.0181079643830694	1.35747599363121	down
Meta_246	4-(4-Nitrobenzyl)pyridine	Benzenoids	Benzene and substituted derivatives	Nitrobenzenes	HMDB0246294		-0.748333333	0.00271812372817807	0.00271812372817807	1.42104931392471	down
Meta_251	xi-5-Dodecanolide	Organoheterocyclic compounds	Lactones	Delta valerolactones	HMDB0037742		-0.419666667	0.0463129639749048	0.0463129639749048	1.20512484342352	down
Meta_255	(2R)-2-Amino-2-[[(1S)-1-carboxyethyl]amino]-4-methylpentanoic acid	Organic acids and derivatives	Carboxylic acids and derivatives	Amino acids, peptides, and analogues	HMDB0242765		-0.492	0.0198263647585013	0.0198263647585013	1.32397313365186	down
Meta_264	Serylleucine	Organic acids and derivatives	Carboxylic acids and derivatives	Amino acids, peptides, and analogues	HMDB0029043		-0.443	0.0460118354863791	0.0460118354863791	1.31988407354451	down
Meta_274	L-Threo-3-Phenylserine	Organic acids and derivatives	Carboxylic acids and derivatives	Amino acids, peptides, and analogues	HMDB0002184		-0.616	0.0151023998280202	0.0151023998280202	1.3791455151108	down
Meta_280	cis-Vitamin K1	Lipids and lipid-like molecules	Prenol lipids	Quinone and hydroquinone lipids	HMDB0250298		-0.516333333	0.0192724552670802	0.0192724552670802	1.32573023599169	down
Meta_282	Carnosine	Organic acids and derivatives	Peptidomimetics	Hybrid peptides	HMDB0000033	C00386	-0.469333333	0.00308225135134511	0.00308225135134511	1.42878131186158	down
Meta_283	Myristic acid	Lipids and lipid-like molecules	Fatty Acyls	Fatty acids and conjugates	HMDB0000806	C06424	-1.539666667	0.0132378345509995	0.0132378345509995	1.39928387332984	down
Meta_284	2-Chloro-1,3,5-triazine	Organoheterocyclic compounds	Triazines	1,3,5-triazines	HMDB0245050		-0.471	0.0225407727905568	0.0225407727905568	1.29418611445706	down
Meta_285	N-Decanoylglycine	Organic acids and derivatives	Carboxylic acids and derivatives	Amino acids, peptides, and analogues	HMDB0013267		-0.780333333	0.0300726386057328	0.0300726386057328	1.38275352590069	down
Meta_286	Methyl (2E,6Z)-dodecadienoate	Lipids and lipid-like molecules	Fatty Acyls	Fatty acid esters	HMDB0031014		-0.646666667	0.011037638491735	0.011037638491735	1.37605615427085	down
Meta_297	Leucyl-Valine	Organic acids and derivatives	Carboxylic acids and derivatives	Amino acids, peptides, and analogues	HMDB0028942		-0.839666667	0.033494350865325	0.033494350865325	1.30048662147102	down
Meta_298	Valylleucine	Organic acids and derivatives	Carboxylic acids and derivatives	Amino acids, peptides, and analogues	HMDB0029131		-0.681333333	0.0224717609357175	0.0224717609357175	1.37161738482359	down
Meta_301	Isobutyryl-L-carnitine	Lipids and lipid-like molecules	Fatty Acyls	Fatty acid esters	HMDB0000736		-0.414666667	0.0351274710453079	0.0351274710453079	1.31042653187882	down
Meta_304	Threonylleucine	Organic acids and derivatives	Carboxylic acids and derivatives	Amino acids, peptides, and analogues	HMDB0029065		-0.464666667	0.0396579132881712	0.0396579132881712	1.25213239520765	down
Meta_306	4-(2-Ethyl-6-methylphenyl)-5-methylmorpholin-3-one	Unclassified	Unclassified	Unclassified			-0.366333333	0.0284681178696544	0.0284681178696544	1.31039629602471	down
Meta_310	Phenylalanylalanine	Organic acids and derivatives	Carboxylic acids and derivatives	Amino acids, peptides, and analogues	HMDB0028988		-0.672333333	0.0402917569087325	0.0402917569087325	1.27814453862594	down
Meta_313	N,N-diethyl-3,4-dimethoxybenzamide	Unclassified	Unclassified	Unclassified			-0.697333333	0.020844655675055	0.020844655675055	1.30720451396381	down
Meta_314	alpha-D-Glucose	Organic oxygen compounds	Organooxygen compounds	Carbohydrates and carbohydrate conjugates	HMDB0003345	C00267	-0.322666667	0.0379836780070605	0.0379836780070605	1.28095149757308	down
Meta_337	Isovalerylcarnitine	Lipids and lipid-like molecules	Fatty Acyls	Fatty acid esters	HMDB0000688		-0.907666667	0.0107395057286273	0.0107395057286273	1.4155701867889	down
Meta_347	Pindolol	Organoheterocyclic compounds	Indoles and derivatives	Indoles	HMDB0015095	C07445	-0.643666667	0.047083520054447	0.047083520054447	1.25849735318355	down
Meta_351	3-(6,7-Dimethoxy-1,2,3,4-tetrahydroisoquinolin-1-yl)propan-1-ol	Unclassified	Unclassified	Unclassified			-1.193333333	0.0201919358012906	0.0201919358012906	1.38505102103306	down
Meta_359	10-Azabenzo(a)pyrene	Unclassified	Unclassified	Unclassified			-0.407666667	0.0205050800350741	0.0205050800350741	1.3685381748612	down
Meta_364	2-Hexyldecanoic acid	Unclassified	Unclassified	Unclassified			-1.682666667	0.0122554089562117	0.0122554089562117	1.38079552443032	down
Meta_37	Glyceric acid	Organic oxygen compounds	Organooxygen compounds	Carbohydrates and carbohydrate conjugates	HMDB0000139	C00258	-1.390333333	0.00389186110323479	0.00389186110323479	1.45407880550306	down
Meta_373	Furafylline	Organoheterocyclic compounds	Imidazopyrimidines	Purines and purine derivatives	HMDB0252528		-0.232333333	0.0050123190546207	0.0050123190546207	1.41068993787399	down
Meta_377	4-Methylpentanoylcarnitine	Lipids and lipid-like molecules	Fatty Acyls	Fatty acid esters	HMDB0241032		-0.97	0.0365041282971751	0.0365041282971751	1.2354903984689	down
Meta_385	Benzarone	Organic oxygen compounds	Organooxygen compounds	Carbonyl compounds	HMDB0248975	C14474	-0.966666667	0.00700206552502614	0.00700206552502614	1.39078403493085	down
Meta_388	Phenylalanylvaline	Organic acids and derivatives	Carboxylic acids and derivatives	Amino acids, peptides, and analogues	HMDB0029008		-0.490666667	0.0453337211915043	0.0453337211915043	1.30191206199958	down
Meta_390	3-(Diethylamino)-1-(3,4-dimethoxyphenyl)propan-1-one	Unclassified	Unclassified	Unclassified			-0.823333333	0.0409996134675226	0.0409996134675226	1.3014685164285	down
Meta_393	N-(4-Aminobenzoyl)-L-glutamic acid	Organic acids and derivatives	Carboxylic acids and derivatives	Amino acids, peptides, and analogues	HMDB0255007		-1.267333333	0.0151333891414132	0.0151333891414132	1.3811692245721	down
Meta_394	Threonylphenylalanine	Organic acids and derivatives	Carboxylic acids and derivatives	Amino acids, peptides, and analogues	HMDB0029068		-1.491333333	0.025713129738795	0.025713129738795	1.29127463782996	down
Meta_411	p(MeOx1-co-PheOx1-CH3) Na+	Unclassified	Unclassified	Unclassified			-1.208333333	0.0334480989067393	0.0334480989067393	1.30615534490153	down
Meta_419	4-Formylaminoantipyrine	Organoheterocyclic compounds	Azoles	Pyrazoles	HMDB0246429		-0.283	0.0144713595499218	0.0144713595499218	1.3962781830399	down
Meta_435	Linoleamide	Lipids and lipid-like molecules	Fatty Acyls	Fatty amides	HMDB0062656		-1.507	0.00251355824257019	0.00251355824257019	1.43779574842012	down
Meta_437	2'-O-Methylinosine	Unclassified	Unclassified	Unclassified			-0.452333333	0.0143910263069365	0.0143910263069365	1.4280416313168	down
Meta_441	Oleic acid	Lipids and lipid-like molecules	Fatty Acyls	Fatty acids and conjugates	HMDB0000207	C00712	-1.268333333	0.0114107008196815	0.0114107008196815	1.38741199281358	down
Meta_442	Hexanoylcarnitine	Lipids and lipid-like molecules	Fatty Acyls	Fatty acid esters	HMDB0000756		-2.070666667	0.009185812115429	0.009185812115429	1.44140481545436	down
Meta_443	N-Demethyltramadol	Benzenoids	Phenol ethers	Anisoles	HMDB0255112		-1.152	0.00301518951793249	0.00301518951793249	1.42252206211637	down
Meta_445	Sphingosine	Organic nitrogen compounds	Organonitrogen compounds	Amines	HMDB0000252	C00319	-1.005333333	0.0086811032105023	0.0086811032105023	1.41144048784434	down

Meta_45	2,4-Dimethylthiazole	Organoheterocyclic compounds	Azoles	Thiazoles	HMDB0032974		-0.540666667	0.011385146048982	0.011385146048982	1.34685038581414	down
Meta_452	Octanoylcarnitine	Lipids and lipid-like molecules	Fatty Acyls	Fatty acid esters	HMDB0000791	C02838	-2.647333333	0.000405583082764522	0.000405583082764522	1.4597404215949	down
Meta_457	2-methoxy-6-methyl-5-(phenylmethoxy)-2H-3,4,5,6-tetrahydropyran-3,4-diol	Unclassified	Unclassified	Unclassified			-2.233333333	0.00412945120249066	0.00412945120249066	1.43776782236118	down
Meta_463	Aspartame	Organic acids and derivatives	Carboxylic acids and derivatives	Amino acids, peptides, and analogues	HMDB0001894	C11045	-0.969333333	0.00190432839752756	0.00190432839752756	1.44234224963448	down
Meta_473	trans-4-Nonenal	Organic oxygen compounds	Organooxygen compounds	Carbonyl compounds	HMDB0032535		-1.512333333	0.0115881923266899	0.0115881923266899	1.4306324312624	down
Meta_508	Dehydrophytosphingosine	Organic nitrogen compounds	Organonitrogen compounds	Amines	HMDB0038057		-0.661	0.0438012177016027	0.0438012177016027	1.2667069813825	down
Meta_517	2-[4-(3,4-Methylenedioxyphenyl)butyl]-4(1H)-quinolinone	Organoheterocyclic compounds	Quinolines and derivatives	Quinolones and derivatives	HMDB0030382		-0.433666667	0.0251116916247414	0.0251116916247414	1.32138947432511	down
Meta_530	Heptaethylene glycol	Organic oxygen compounds	Organooxygen compounds	Ethers	HMDB0061835		-0.493666667	0.0315888913611511	0.0315888913611511	1.28381976855682	down
Meta_550	(3E,5E,8Z)-Deca-3,5,8-trienedioylcarnitine	Lipids and lipid-like molecules	Fatty Acyls	Fatty acid esters	HMDB0241127		-0.453666667	0.021372116902179	0.021372116902179	1.31296102145026	down
Meta_554	5-Dodecenoylcarnitine	Lipids and lipid-like molecules	Fatty Acyls	Fatty acid esters	HMDB0241212		-2.409666667	0.0031193802467515	0.0031193802467515	1.43971196830947	down
Meta_558	Dodecanoylcarnitine	Lipids and lipid-like molecules	Fatty Acyls	Fatty acid esters	HMDB0002250		-2.285666667	0.00061485191615633	0.00061485191615633	1.45264485280681	down
Meta_564	(+)-Calycanthine	Organoheterocyclic compounds	Quinolines and derivatives	Aminoquinolines and derivatives	HMDB0029561	C10573	-0.583	0.0400353335325799	0.0400353335325799	1.24622404721801	down
Meta_602	Myristoleoylcarnitine	Lipids and lipid-like molecules	Fatty Acyls	Fatty acid esters	HMDB0240588		-2.505	0.0107828523452188	0.0107828523452188	1.43248619958684	down
Meta_606	Tetradecanoylcarnitine	Lipids and lipid-like molecules	Fatty Acyls	Fatty acid esters	HMDB0005066		-1.397	0.00458222539509993	0.00458222539509993	1.39750265548497	down
Meta_612	(10Z,13Z,16Z)-N-(2-hydroxyethyl)docosa-10,13,16-trienamide	Unclassified	Unclassified	Unclassified			-0.843333333	0.0152105184394369	0.0152105184394369	1.42363862129383	down
Meta_617	Reichsteins Substance S	Lipids and lipid-like molecules	Steroids and steroid derivatives	Hydroxysteroids	HMDB0257139		-0.936	0.0254005789369829	0.0254005789369829	1.39771886237411	down
Meta_624	N-Palmitoyl Lysine	Organic acids and derivatives	Carboxylic acids and derivatives	Amino acids, peptides, and analogues	HMDB0241929		-1.467	0.0245970301518719	0.0245970301518719	1.29245739575928	down
Meta_631	3-[2-(2,3-dihydroxy-5,6,8a-trimethyloctahydro-2H-spiro[naphthalene-1,2'-oxiran]-5-yl)ethyl]-5-hydroxyfuran-2(5H)-one	Unclassified	Unclassified	Unclassified			-0.327	0.0101814995009753	0.0101814995009753	1.40055765977666	down
Meta_643	9-Hexadecenoylcarnitine	Lipids and lipid-like molecules	Fatty Acyls	Fatty acid esters	HMDB0013207		-1.294333333	0.00132321839468624	0.00132321839468624	1.44009074883154	down
Meta_645	Palmitoylcarnitine	Lipids and lipid-like molecules	Fatty Acyls	Fatty acid esters	HMDB0000222	C02990	-1.492	0.00240861103640588	0.00240861103640588	1.42221873037223	down
Meta_659	Heptadecanoyl carnitine	Lipids and lipid-like molecules	Fatty Acyls	Fatty acid esters	HMDB0006210		-2.391333333	0.0484044474349924	0.0484044474349924	1.32457794068587	down
Meta_67	Thymine	Organoheterocyclic compounds	Diazines	Pyrimidines and pyrimidine derivatives	HMDB0000262	C00178	-0.815666667	0.0365393497697579	0.0365393497697579	1.35638511538801	down
Meta_670	Oleoylcarnitine	Lipids and lipid-like molecules	Fatty Acyls	Fatty acid esters	HMDB0005065		-1.014	0.0173032510627545	0.0173032510627545	1.31432033266888	down
Meta_672	Stearoylcarnitine	Lipids and lipid-like molecules	Fatty Acyls	Fatty acid esters	HMDB0000848		-1.445666667	0.00506012798272088	0.00506012798272088	1.43773348636361	down
Meta_71	5,6-dihydrothymine	Organoheterocyclic compounds	Diazines	Pyrimidines and pyrimidine derivatives	HMDB0304210	C00906	-1.267333333	0.00593105162073538	0.00593105162073538	1.40502971098612	down
Meta_719	Crustecdysone	Lipids and lipid-like molecules	Steroids and steroid derivatives	Bile acids, alcohols and derivatives	HMDB0030180	C02633	-0.118333333	0.0176728453557055	0.0176728453557055	1.32903676965337	down
Meta_734	LPE O-20:1	Unclassified	Unclassified	Unclassified			-0.389666667	0.0163735046387548	0.0163735046387548	1.33025274912425	down
Meta_76	Pipecolic acid	Organic acids and derivatives	Carboxylic acids and derivatives	Amino acids, peptides, and analogues	HMDB0000070	C00408	-0.139	0.0212594378705121	0.0212594378705121	1.33078427765514	down
Meta_765	myo-Inositol 1,3,4,5-tetrakisphosphate	Organic oxygen compounds	Organooxygen compounds	Alcohols and polyols	HMDB0001059	C01272	-0.451333333	0.0367795541724407	0.0367795541724407	1.2989561358757	down
Meta_78	Dibutylamine	Organic nitrogen compounds	Organonitrogen compounds	Amines	HMDB0251174		-0.301	0.0339056481289376	0.0339056481289376	1.39048425952114	down
Meta_785	Cer(d18:1/16:0)	Lipids and lipid-like molecules	Sphingolipids	Ceramides	HMDB0004949	C00195	-0.413333333	0.032713776443852	0.032713776443852	1.29844049169457	down
Meta_816	Uridine diphosphate glucuronic acid	Organoheterocyclic compounds	Lactones	Gamma butyrolactones	HMDB0000935	C00167	-0.309666667	0.0269275899630454	0.0269275899630454	1.28362826142559	down
Meta_817	N-[(4E,8E)-1,3-dihydroxyoctadeca-4,8-dien-2-yl]hexadecanamide	Lipids and lipid-like molecules	Sphingolipids	Ceramides	HMDB0035480		-0.72	0.0169728438048153	0.0169728438048153	1.33128172420774	down
Meta_827	Cer(d18:1/18:0)	Lipids and lipid-like molecules	Sphingolipids	Ceramides	HMDB0004950	C00195	-1.491333333	0.000572286192873184	0.000572286192873184	1.45165094244657	down
Meta_828	Austrobuxusin I	Unclassified	Unclassified	Unclassified			-0.33	0.0480023813584839	0.0480023813584839	1.24035756584148	down
Meta_830	PE-Cer 12:0;2O/20:5	Unclassified	Unclassified	Unclassified			-1.539333333	0.00422837350879791	0.00422837350879791	1.41001112907193	down
Meta_832	PC O-26:0	Unclassified	Unclassified	Unclassified			-0.652	0.00942786740368436	0.00942786740368436	1.37680240846675	down
Meta_852	SM(d18:1/15:0)	Lipids and lipid-like molecules	Sphingolipids	Phosphosphingolipids	HMDB0240608		-0.202	0.0236746093738048	0.0236746093738048	1.31441868901531	down
Meta_856	PC O-30:0	Unclassified	Unclassified	Unclassified			-0.403	0.000476047002074358	0.000476047002074358	1.46471062541611	down
Meta_863	PC(14:0/16:0)	Lipids and lipid-like molecules	Glycerophospholipids	Glycerophosphocholines	HMDB0007869		-0.366666667	0.0275561101248568	0.0275561101248568	1.37147949594938	down
Meta_867	PC O-12:0_16:0	Unclassified	Unclassified	Unclassified			-0.441333333	0.0259604043710488	0.0259604043710488	1.37708814340592	down
Meta_869	PC O-32:4	Unclassified	Unclassified	Unclassified			-0.664666667	0.0214059327464869	0.0214059327464869	1.37420870458849	down
Meta_880	SM 8:0;2O/24:1	Unclassified	Unclassified	Unclassified			-0.153666667	0.0376014548865532	0.0376014548865532	1.27555925295652	down
Meta_882	PE O-16:1_20:5	Unclassified	Unclassified	Unclassified			-0.416	0.00809699863929273	0.00809699863929273	1.3688146232887	down
Meta_884	PE O-16:1_20:4	Unclassified	Unclassified	Unclassified			-0.286	0.0120072437767482	0.0120072437767482	1.38564592666928	down
Meta_886	PE O-16:1_20:3	Unclassified	Unclassified	Unclassified			-0.777333333	0.0301799254435483	0.0301799254435483	1.38120662875926	down
Meta_888	PC 30:0	Unclassified	Unclassified	Unclassified			-0.743	0.00242669762088202	0.00242669762088202	1.45079619741922	down
Meta_890	PE O-18:2_18:1	Unclassified	Unclassified	Unclassified			-0.414666667	0.0331017519779258	0.0331017519779258	1.37169044510114	down
Meta_891	SM(d18:1/18:1(9Z))	Lipids and lipid-like molecules	Sphingolipids	Phosphosphingolipids	HMDB0012101		-0.556666667	0.0427477430982251	0.0427477430982251	1.33102264517998	down
Meta_894	SM(d18:1/18:0)	Lipids and lipid-like molecules	Sphingolipids	Phosphosphingolipids	HMDB0001348		-0.446	0.0145260980384779	0.0145260980384779	1.36971185185162	down
Meta_898	SM 8:1;2O/25:0	Unclassified	Unclassified	Unclassified			-0.233666667	0.0315586186183051	0.0315586186183051	1.33353119977505	down
Meta_900	PC(14:0/18:0)	Lipids and lipid-like molecules	Glycerophospholipids	Glycerophosphocholines	HMDB0007871		-1.158666667	0.0053585395885508	0.0053585395885508	1.45134698000985	down
Meta_905	PC O-16:0_14:0	Unclassified	Unclassified	Unclassified			-0.391333333	0.0180198962840424	0.0180198962840424	1.38856046959733	down
Meta_914	PC(P-16:0/18:2(9Z,12Z))	Lipids and lipid-like molecules	Glycerophospholipids	Glycerophosphocholines	HMDB0011211		-0.890666667	0.0167564097782415	0.0167564097782415	1.39140084348106	down
Meta_916	GlcCer(d18:1/16:0)	Lipids and lipid-like molecules	Sphingolipids	Glycosphingolipids	HMDB0004971		-0.692666667	0.0271605575313037	0.0271605575313037	1.28459619298779	down
Meta_920	PE O-18:2_20:5	Unclassified	Unclassified	Unclassified			-0.930333333	0.027026154323841	0.027026154323841	1.29364854251371	down
Meta_922	SM 8:0;2O/26:1	Unclassified	Unclassified	Unclassified			-0.284666667	0.0176968845429074	0.0176968845429074	1.32459527915165	down
Meta_927	PE O-18:2_20:4	Unclassified	Unclassified	Unclassified			-0.480333333	0.00180162302025918	0.00180162302025918	1.45120211011375	down
Meta_931	PC(16:0/18:3(6Z,9Z,12Z))	Lipids and lipid-like molecules	Glycerophospholipids	Glycerophosphocholines	HMDB0007974		-1.858	0.0375367925421388	0.0375367925421388	1.35056405425948	down
Meta_937	PC(16:0/18:1(9Z))	Lipids and lipid-like molecules	Glycerophospholipids	Glycerophosphocholines	HMDB0007972	C13875	-0.393666667	0.0379052989756058	0.0379052989756058	1.24604280936898	down
Meta_938	SM(d18:1/16:0)	Lipids and lipid-like molecules	Sphingolipids	Phosphosphingolipids	HMDB0010169		-0.212333333	0.0455109165468593	0.0455109165468593	1.26614465454174	down
Meta_94	2-Phenylacetamide	Benzenoids	Benzene and substituted derivatives	Phenylacetamides	HMDB0010715	C02505	-0.396	0.0221530946181127	0.0221530946181127	1.4004049323005	down
Meta_944	PC(15:0/16:0)	Lipids and lipid-like molecules	Glycerophospholipids	Glycerophosphocholines	HMDB0007935		-0.864666667	0.00024954974547254	0.00024954974547254	1.46732001678343	down

Meta_945	PC O-16:0_16:0	Unclassified	Unclassified	Unclassified			-0.779666667	0.00138180418856167	0.00138180418856167	1.44295316938676	down
Meta_951	PC(14:1(9Z)/P-16:0)	Lipids and lipid-like molecules	Glycerophospholipids	Glycerophosphocholines	HMDB0007928		-0.636	0.04829730117121	0.04829730117121	1.21422566198745	down
Meta_956	SM 8:0;2O/28:1	Unclassified	Unclassified	Unclassified			-0.429333333	0.00424656525199521	0.00424656525199521	1.4121597176687	down
Meta_963	PC(16:0/16:0)	Lipids and lipid-like molecules	Glycerophospholipids	Glycerophosphocholines	HMDB0000564		-1.223333333	0.000213728806137118	0.000213728806137118	1.47839775657563	down
Meta_967	PC 34:0	Unclassified	Unclassified	Unclassified			-1.761333333	0.0252520036230184	0.0252520036230184	1.3819822443113	down
Meta_972	PC(O-16:0/18:2(9Z,12Z))	Lipids and lipid-like molecules	Glycerophospholipids	Glycerophosphocholines	HMDB0011151		-0.560333333	0.0365235951545372	0.0365235951545372	1.26557258115302	down
Meta_978	PC 16:0_17:0	Unclassified	Unclassified	Unclassified			-0.538	0.00532163407256971	0.00532163407256971	1.40937526029813	down
Meta_980	PC(P-16:0/22:4(7Z,10Z,13Z,16Z))	Lipids and lipid-like molecules	Glycerophospholipids	Glycerophosphocholines	HMDB0011226		-0.233666667	0.0191328988950474	0.0191328988950474	1.32088660713067	down
Meta_987	PC(16:0/18:0)	Lipids and lipid-like molecules	Glycerophospholipids	Glycerophosphocholines	HMDB0007970		-1.573666667	0.00755663042493419	0.00755663042493419	1.44504625123476	down
Meta_995	PC(O-18:1(9Z)/18:2(9Z,12Z))	Lipids and lipid-like molecules	Glycerophospholipids	Glycerophosphocholines	HMDB0013429		-0.471666667	0.00945932807045593	0.00945932807045593	1.36996882114952	down
Meta_1	Acetaldehyde oxime	Organic nitrogen compounds	Organonitrogen compounds	Oximes	HMDB0003656	C02658	5.31466666666667	0.149688627500128	0.149688627500128	1.12170451617981	unchanged
Meta_10	Glyoxylic acid	Organic acids and derivatives	Carboxylic acids and derivatives	Carboxylic acids	HMDB0000119	C00048	0.304666666666668	0.0971054702998163	0.0971054702998163	1.14537516818146	unchanged
Meta_100	Chloroform	Organohalogen compounds	Alkyl halides	Halomethanes	HMDB0029596	C13827	-0.358666667	0.246439307331524	0.246439307331524	0.943275891017049	unchanged
Meta_1000	PC 17:0_18:1	Unclassified	Unclassified	Unclassified			-0.002	0.995859123988936	0.995859123988936	0.024663660920555	unchanged
Meta_1001	PC O-40:7	Unclassified	Unclassified	Unclassified			-0.411	0.127177986707514	0.127177986707514	1.03002802227185	unchanged
Meta_1002	PC O-40:6	Unclassified	Unclassified	Unclassified			-0.425	0.197303673662855	0.197303673662855	0.901037586654649	unchanged
Meta_1003	PC(20:1(11Z)/P-18:0)	Lipids and lipid-like molecules	Glycerophospholipids	Glycerophosphocholines	HMDB0008325		-0.600666667	0.101385371182725	0.101385371182725	1.07881407925354	unchanged
Meta_1004	[5-Hydroxy-6-[2-(3-hydroxy-4-methoxyphenyl)ethoxy]-2-[[3,4,5-trihydroxy-6-(hydroxymethyl)oxan-2-yl]oxymethyl]-4-(3,4,5-trihydroxy-6-methyloxan-2-yl)oxyoxan-3-yl] 3-(3,4-dihydroxyphenyl)prop-2-enoate	Unclassified	Unclassified	Unclassified			0.23	0.687086177849088	0.687086177849088	0.291733500652198	unchanged
Meta_1006	PC(16:1(9Z)/20:4(8Z,11Z,14Z,17Z))	Lipids and lipid-like molecules	Glycerophospholipids	Glycerophosphocholines	HMDB0008016		-0.158333333	0.502608663646378	0.502608663646378	0.5525749512446	unchanged
Meta_1007	PC(16:0/20:4(8Z,11Z,14Z,17Z))	Lipids and lipid-like molecules	Glycerophospholipids	Glycerophosphocholines	HMDB0007983		-0.144	0.333083601914971	0.333083601914971	0.70769774573282	unchanged
Meta_1008	PC 16:1_20:3	Unclassified	Unclassified	Unclassified			0.549666666666667	0.0771759546910954	0.0771759546910954	1.21876165159676	unchanged
Meta_1009	PC 38:6	Unclassified	Unclassified	Unclassified			-0.462333333	0.065092036318381	0.065092036318381	1.24838064942539	unchanged
Meta_101	2-Acetyl-4-methylthiazole	Organic oxygen compounds	Organooxygen compounds	Carbonyl compounds	HMDB0302368		-0.762666667	0.188269706074788	0.188269706074788	0.964527257747572	unchanged
Meta_1010	PC(18:1(9Z)/18:2(9Z,12Z))	Lipids and lipid-like molecules	Glycerophospholipids	Glycerophosphocholines	HMDB0008105		-0.049333333	0.871945011591777	0.871945011591777	0.146567486653534	unchanged
Meta_1013	PC(20:4(5Z,8Z,11Z,14Z)/20:4(5Z,8Z,11Z,14Z))	Lipids and lipid-like molecules	Glycerophospholipids	Glycerophosphocholines	HMDB0008443		0.728666666666667	0.479435236546422	0.479435236546422	0.542541296786213	unchanged
Meta_1014	PC(18:1(9Z)/18:1(9Z))	Lipids and lipid-like molecules	Glycerophospholipids	Glycerophosphocholines	HMDB0000593		0.154999999999998	0.348694608584093	0.348694608584093	0.734646999188058	unchanged
Meta_1015	SM 8:0;2O/32:1	Unclassified	Unclassified	Unclassified			-1.512666667	0.204225167896303	0.204225167896303	0.955699172611519	unchanged
Meta_1016	PC(18:0/18:1(9Z))	Lipids and lipid-like molecules	Glycerophospholipids	Glycerophosphocholines	HMDB0008038		-0.236333333	0.308556247317931	0.308556247317931	0.795816662238051	unchanged
Meta_1017	PC O-16:1_22:6	Unclassified	Unclassified	Unclassified			0.457000000000001	0.294530018476389	0.294530018476389	0.803510452655935	unchanged
Meta_1019	PC O-18:2_20:4	Unclassified	Unclassified	Unclassified			0.417666666666667	0.280019049796634	0.280019049796634	0.865582255689552	unchanged
Meta_102	5-(2-Hydroxyethyl)-4-methylthiazole	Organoheterocyclic compounds	Azoles	Thiazoles	HMDB0032985	C04294	-0.143666667	0.933887064650354	0.933887064650354	0.0827111975204797	unchanged
Meta_1020	PC 38:2	Unclassified	Unclassified	Unclassified			-0.665666667	0.234982505661679	0.234982505661679	0.931699318434672	unchanged
Meta_1021	PC 17:1_20:4	Unclassified	Unclassified	Unclassified			0.399333333333335	0.201072284587165	0.201072284587165	0.925548660217407	unchanged
Meta_1022	PC O-18:1_20:4	Unclassified	Unclassified	Unclassified			-0.280333333	0.35502322393447	0.35502322393447	0.748029316811411	unchanged
Meta_1023	PC O-16:0_22:5	Unclassified	Unclassified	Unclassified			-0.129666667	0.500956932299761	0.500956932299761	0.520627216209051	unchanged
Meta_1024	PC O-16:0_22:4	Unclassified	Unclassified	Unclassified			-0.189	0.125731879849804	0.125731879849804	1.04136807441662	unchanged
Meta_1025	PC O-18:0_20:4	Unclassified	Unclassified	Unclassified			-0.313666667	0.323184311753596	0.323184311753596	0.792310134088814	unchanged
Meta_1026	PC 18:1_19:1	Unclassified	Unclassified	Unclassified			0.289666666666667	0.127386916406361	0.127386916406361	1.05631088139005	unchanged
Meta_1027	PG(i-22:0/18:1(12Z)-O(9S,10R))	Unclassified	Unclassified	Unclassified	HMDB0272143		0.00266666666666637	0.992131783912891	0.992131783912891	0.0132244255253333	unchanged
Meta_1028	PC(16:0/22:6(4Z,7Z,10Z,13Z,16Z,19Z))	Lipids and lipid-like molecules	Glycerophospholipids	Glycerophosphocholines	HMDB0007991		-0.107333333	0.347828403105253	0.347828403105253	0.698450561766201	unchanged
Meta_1029	PC(18:1(9Z)/20:5(5Z,8Z,11Z,14Z,17Z))	Lipids and lipid-like molecules	Glycerophospholipids	Glycerophosphocholines	HMDB0008116		0.843666666666668	0.0738267445481085	0.0738267445481085	1.22123091286479	unchanged
Meta_1031	PC(18:0/20:4(8Z,11Z,14Z,17Z))	Lipids and lipid-like molecules	Glycerophospholipids	Glycerophosphocholines	HMDB0008049		-0.004333333	0.981887965518081	0.981887965518081	0.0174827316195524	unchanged
Meta_1032	PC 18:1_20:3	Unclassified	Unclassified	Unclassified			-0.929666667	0.165368600745293	0.165368600745293	1.06971166263417	unchanged
Meta_1035	PC(18:1(9Z)/20:1(11Z))	Lipids and lipid-like molecules	Glycerophospholipids	Glycerophosphocholines	HMDB0008110		-0.270666667	0.12164065559701	0.12164065559701	1.02590074489997	unchanged
Meta_1036	SM 8:0;2O/34:1	Unclassified	Unclassified	Unclassified			-0.382	0.800435369106703	0.800435369106703	0.215763276783118	unchanged
Meta_1037	LacCer(d18:1/16:0)	Lipids and lipid-like molecules	Sphingolipids	Glycosphingolipids	HMDB0006750	C01290	-0.975	0.167286112904781	0.167286112904781	1.06795270300486	unchanged
Meta_1038	PC(18:1(9Z)/22:6(4Z,7Z,10Z,13Z,16Z,19Z))	Lipids and lipid-like molecules	Glycerophospholipids	Glycerophosphocholines	HMDB0008123		0.206666666666667	0.1707617956342	0.1707617956342	1.02221057414152	unchanged
Meta_1039	PC(18:1(9Z)/22:5(7Z,10Z,13Z,16Z,19Z))	Lipids and lipid-like molecules	Glycerophospholipids	Glycerophosphocholines	HMDB0008122		0.538333333333334	0.113064056093965	0.113064056093965	1.1341490755981	unchanged
Meta_105	Oxoglutaric acid	Organic acids and derivatives	Keto acids and derivatives	Gamma-keto acids and derivatives	HMDB0000208	C00026	1.23666666666667	0.0943496363314892	0.0943496363314892	1.23464826126762	unchanged
Meta_106	Glucal	Organic oxygen compounds	Organooxygen compounds	Alcohols and polyols	HMDB0252763		-0.064	0.791305070078683	0.791305070078683	0.214697320037704	unchanged
Meta_107	L-Glutamic acid	Organic acids and derivatives	Carboxylic acids and derivatives	Amino acids, peptides, and analogues	HMDB0000148	C00025	0.415666666666667	0.155453500093321	0.155453500093321	0.992300769740686	unchanged
Meta_11	2-Methyl-1-propylamine	Organic nitrogen compounds	Organonitrogen compounds	Amines	HMDB0034198	C02787	-0.303666667	0.572035161173679	0.572035161173679	0.445746637431717	unchanged
Meta_111	Spermidine	Organic nitrogen compounds	Organonitrogen compounds	Amines	HMDB0001257	C00315	-0.107666667	0.864069296032034	0.864069296032034	0.16486120973382	unchanged
Meta_113	Propyl 2-mercaptopropionate	Organic acids and derivatives	Carboxylic acids and derivatives	Carboxylic acid derivatives	HMDB0032495		0.443000000000001	0.203038275687972	0.203038275687972	0.934211246611997	unchanged
Meta_114	L-Lysine	Organic acids and derivatives	Carboxylic acids and derivatives	Amino acids, peptides, and analogues	HMDB0000182	C00047	-0.166666667	0.0721159087396922	0.0721159087396922	1.20571490395819	unchanged
Meta_118	Guanine	Organoheterocyclic compounds	Imidazopyrimidines	Purines and purine derivatives	HMDB0000132	C00242	0.466666666666665	0.190027177777566	0.190027177777566	0.968922520546038	unchanged
Meta_119	Dopamine	Benzenoids	Phenols	Benzenediols	HMDB0000073	C03758	-0.338333333	0.390513313942783	0.390513313942783	0.676110716449474	unchanged
Meta_12	Propionic acid	Organic acids and derivatives	Carboxylic acids and derivatives	Carboxylic acids	HMDB0000237	C00163	0.686666666666667	0.137174994054407	0.137174994054407	1.04544642257628	unchanged
Meta_120	2-Azaspiro[4.5]decan-3-one	Organoheterocyclic compounds	Azaspirodecane derivatives	Unclassified	HMDB0247166		-0.066666667	0.556150101110981	0.556150101110981	0.473369790018827	unchanged
Meta_121	4-Chlorobenzoic acid	Benzenoids	Benzene and substituted derivatives	Benzoic acids and derivatives	HMDB0246393	C02370	-0.181666667	0.115017159490277	0.115017159490277	1.14452540689737	unchanged
Meta_123	Dimethyl sulfoxide	Organosulfur compounds	Sulfoxides	Unclassified	HMDB0002151	C11143	0.536333333333332	0.0929452353605723	0.0929452353605723	1.17644092251098	unchanged
Meta_124	2,5-Dimethyl-4-ethoxy-3(2H)-furanone	Organoheterocyclic compounds	Dihydrofurans	Furanones	HMDB0032232		-0.393	0.22093266086526	0.22093266086526	0.920293208985271	unchanged
Meta_125	N-Acetylvaline	Organic acids and derivatives	Carboxylic acids and derivatives	Amino acids, peptides, and analogues	HMDB0011757		-0.046	0.859964308990746	0.859964308990746	0.153868324150761	unchanged
Meta_126	3a,6a,7b-Trihydroxy-5b-cholanoic acid	Lipids and lipid-like molecules	Steroids and steroid derivatives	Bile acids, alcohols and derivatives	HMDB0000364		-0.115333333	0.433791937713145	0.433791937713145	0.651066161138126	unchanged

Meta_127	Citiolone	Organic acids and derivatives	Carboxylic acids and derivatives	Amino acids, peptides, and analogues	HMDB0250301		0.00233333333333263	0.991680545049248	0.991680545049248	0.00226049236463395	unchanged
Meta_129	1-Propanol, 3,3,3-trichloro-	Organic oxygen compounds	Organooxygen compounds	Alcohols and polyols	HMDB0244002		-0.090333333	0.943637418886683	0.943637418886683	0.0423431543784087	unchanged
Meta_13	Glycine	Organic acids and derivatives	Carboxylic acids and derivatives	Amino acids, peptides, and analogues	HMDB0000123	C00037	0.577000000000002	0.11753411351547	0.11753411351547	1.09602563636821	unchanged
Meta_130	2-Naphthalenethiol	Benzenoids	Naphthalenes	Unclassified	HMDB0029689		-0.296666667	0.0824761864140932	0.0824761864140932	1.25889912641773	unchanged
Meta_132	trans-S-(1-Propenyl)-L-cysteine	Organic acids and derivatives	Carboxylic acids and derivatives	Amino acids, peptides, and analogues	HMDB0029440		0.0849999999999991	0.38525762889704	0.38525762889704	0.632311503804295	unchanged
Meta_133	Glutamic acid gamma-methyl ester	Organic acids and derivatives	Carboxylic acids and derivatives	Amino acids, peptides, and analogues	HMDB0061715		0.0246666666666684	0.934153881390657	0.934153881390657	0.0773511662374705	unchanged
Meta_135	1-Phenyl-1-pentanone	Organic oxygen compounds	Organooxygen compounds	Carbonyl compounds	HMDB0031208		-0.249666667	0.204617344248424	0.204617344248424	0.98785583369729	unchanged
Meta_136	(S1)-Methoxy-3-heptanethiol	Organic oxygen compounds	Organooxygen compounds	Ethers	HMDB0032380		-0.168	0.323852873396866	0.323852873396866	0.746727318946014	unchanged
Meta_137	Methionine methyl ester	Organic acids and derivatives	Carboxylic acids and derivatives	Amino acids, peptides, and analogues	HMDB0254602		-0.329	0.123597036300552	0.123597036300552	1.04605640410922	unchanged
Meta_138	Chloral hydrate	Organohalogen compounds	Halohydrins	Chlorohydrins	HMDB0060451	C06899	-0.179666667	0.741694165583345	0.741694165583345	0.276677349846327	unchanged
Meta_140	Phthalic acid	Benzenoids	Benzene and substituted derivatives	Benzoic acids and derivatives	HMDB0002107	C01606	-0.535333333	0.320977819982979	0.320977819982979	0.788344469655141	unchanged
Meta_142	L-3-Phenyllactic acid	Phenylpropanoids and polyketides	Phenylpropanoic acids	Unclassified	HMDB0000748	C05607	-0.332	0.154517073262392	0.154517073262392	1.01599112722841	unchanged
Meta_144	Dichloromethane	Organohalogen compounds	Alkyl halides	Halomethanes	HMDB0031548	C02271	-0.407333333	0.150345813725549	0.150345813725549	1.07266099396752	unchanged
Meta_145	Uric acid	Organoheterocyclic compounds	Imidazopyrimidines	Purines and purine derivatives	HMDB0000289	C00366	-0.244666667	0.171830415910122	0.171830415910122	0.952705733280973	unchanged
Meta_147	Pyridoxal	Organoheterocyclic compounds	Pyridines and derivatives	Pyridine carboxaldehydes	HMDB0001545	C00250	0.143666666666668	0.473343029856293	0.473343029856293	0.525087998030978	unchanged
Meta_148	Chloromethyl chloroformate	Organic acids and derivatives	Organic carbonic acids and derivatives	Unclassified	HMDB0250119		-0.381666667	0.432423601368345	0.432423601368345	0.58996346564513	unchanged
Meta_15	Dichloromethylene	Organohalogen compounds	Organochlorides	Unclassified	HMDB0251189		-0.232666667	0.524456147511201	0.524456147511201	0.522646628679994	unchanged
Meta_151	Pyridoxine	Organoheterocyclic compounds	Pyridines and derivatives	Pyridoxines	HMDB0000239	C00314	-0.008666667	0.976853532027758	0.976853532027758	0.0404226099855274	unchanged
Meta_152	Glycerophosphoric acid	Lipids and lipid-like molecules	Glycerophospholipids	Glycerophosphates	HMDB0252849	C03189	0.0086666666666666	0.98433745030054	0.98433745030054	0.033786259535844	unchanged
Meta_153	Capric acid	Lipids and lipid-like molecules	Fatty Acyls	Fatty acids and conjugates	HMDB0000511	C01571	-0.097666667	0.369864373935147	0.369864373935147	0.70754619185151	unchanged
Meta_154	Hexanoylglycine	Organic acids and derivatives	Carboxylic acids and derivatives	Amino acids, peptides, and analogues	HMDB0000701		-0.161333333	0.460825174805245	0.460825174805245	0.577796923490638	unchanged
Meta_155	Gabapentin hydrochloride	Unclassified	Unclassified	Unclassified			0.306999999999999	0.639681463754211	0.639681463754211	0.37610864630649	unchanged
Meta_156	Phenol sulphate	Organic acids and derivatives	Organic sulfuric acids and derivatives	Arylsulfates	HMDB0060015	C02180	0.0126666666666662	0.976489144853748	0.976489144853748	0.0374102624321344	unchanged
Meta_158	Suberic acid	Lipids and lipid-like molecules	Fatty Acyls	Fatty acids and conjugates	HMDB0000893	C08278	-0.308666667	0.257800873725705	0.257800873725705	0.87195233928602	unchanged
Meta_159	(卤)-3-Hydroxynonanoic acid	Organic acids and derivatives	Hydroxy acids and derivatives	Medium-chain hydroxy acids and derivatives	HMDB0031513		-0.115666667	0.336467770235652	0.336467770235652	0.742849520266489	unchanged
Meta_160	N-Acetyl-L-aspartic acid	Organic acids and derivatives	Carboxylic acids and derivatives	Amino acids, peptides, and analogues	HMDB0000812	C01042	0.137999999999998	0.642322572662991	0.642322572662991	0.342957853529034	unchanged
Meta_161	2-Oxoarginine	Organic acids and derivatives	Keto acids and derivatives	Short-chain keto acids and derivatives	HMDB0004225	C03771	-0.233333333	0.0617592681599147	0.0617592681599147	1.16471190451095	unchanged
Meta_163	Ureidosuccinic acid	Organic acids and derivatives	Carboxylic acids and derivatives	Amino acids, peptides, and analogues	HMDB0000828		1.50166666666667	0.126896995447388	0.126896995447388	1.10073368043541	unchanged
Meta_164	4-Methylumbelliferone	Phenylpropanoids and polyketides	Coumarins and derivatives	Hydroxycoumarins	HMDB0059622	C03081	0.0839999999999996	0.731903241194949	0.731903241194949	0.255458467748554	unchanged
Meta_165	L-Arginine	Organic acids and derivatives	Carboxylic acids and derivatives	Amino acids, peptides, and analogues	HMDB0000517	C00062	0.0240000000000009	0.912751235901199	0.912751235901199	0.0863757421608016	unchanged
Meta_167	L-Alloisoleucine	Organic acids and derivatives	Carboxylic acids and derivatives	Amino acids, peptides, and analogues	HMDB0000557		-0.192333333	0.578792481739826	0.578792481739826	0.456432877256638	unchanged
Meta_168	Gentianine	Organoheterocyclic compounds	Pyranopyridines	Unclassified	HMDB0303030	C06525	-0.383333333	0.298625492067629	0.298625492067629	0.856427987656223	unchanged
Meta_169	Indoleacetic acid	Organoheterocyclic compounds	Indoles and derivatives	Indolyl carboxylic acids and derivatives	HMDB0000197	C00954	0.437666666666667	0.371600930766114	0.371600930766114	0.706948871808225	unchanged
Meta_17	Piperidine	Organoheterocyclic compounds	Piperidines	Unclassified	HMDB0034301	C01746	0.229333333333337	0.354064277050568	0.354064277050568	0.72959519149185	unchanged
Meta_171	N,N-dimethylindoliumolate	Organoheterocyclic compounds	Indoles and derivatives	Unclassified	HMDB0060063		-1.234333333	0.103558384521515	0.103558384521515	1.18219958160737	unchanged
Meta_172	Cysteinylglycine	Organic acids and derivatives	Carboxylic acids and derivatives	Amino acids, peptides, and analogues	HMDB0000078	C01419	-0.815	0.296864167622858	0.296864167622858	0.760612860172988	unchanged
Meta_173	myo-Inositol	Organic oxygen compounds	Organooxygen compounds	Alcohols and polyols	HMDB0000211	C00137	-0.090666667	0.588199016830874	0.588199016830874	0.410815851569005	unchanged
Meta_175	Methyl l-phenylalaninate	Organic acids and derivatives	Carboxylic acids and derivatives	Amino acids, peptides, and analogues	HMDB0245704		-0.486	0.0616254088955137	0.0616254088955137	1.18418402941018	unchanged
Meta_177	Methyl Phenylglyoxalate	Benzenoids	Benzene and substituted derivatives	Benzoyl derivatives	HMDB0062605		0.0560000000000009	0.653197186598387	0.653197186598387	0.326031650993858	unchanged
Meta_179	2,4-Dinitrophenol	Benzenoids	Phenols	Nitrophenols	HMDB0245462	C02496	-0.082666667	0.770292001162494	0.770292001162494	0.263844633327347	unchanged
Meta_18	Pyruvic acid	Organic acids and derivatives	Keto acids and derivatives	Alpha-keto acids and derivatives	HMDB0000243	C00022	0.367333333333331	0.456797349239388	0.456797349239388	0.581363780495968	unchanged
Meta_180	Phosphorylcholine	Organic nitrogen compounds	Organonitrogen compounds	Quaternary ammonium salts	HMDB0001565	C00588	-0.188	0.5668194473758	0.5668194473758	0.435764473645763	unchanged
Meta_181	Normetanephrine	Benzenoids	Phenols	Methoxyphenols	HMDB0000819	C05589	0.316000000000003	0.0714689982614089	0.0714689982614089	1.23309344391914	unchanged
Meta_182	N-Heptanoylglycine	Organic acids and derivatives	Carboxylic acids and derivatives	Amino acids, peptides, and analogues	HMDB0013010		-0.104666667	0.355370159922716	0.355370159922716	0.69728340490561	unchanged
Meta_183	TRIBUTYLAMINE	Organic nitrogen compounds	Organonitrogen compounds	Amines	HMDB0259165		0.439666666666668	0.453255189606904	0.453255189606904	0.578214554229853	unchanged
Meta_184	Oxypurinol	Organoheterocyclic compounds	Imidazopyrimidines	Purines and purine derivatives	HMDB0000786	C07599	-0.932666667	0.0893333582921558	0.0893333582921558	1.23330711188977	unchanged
Meta_185	p-Cresol sulfate	Organic acids and derivatives	Organic sulfuric acids and derivatives	Arylsulfates	HMDB0011635		0.102	0.530438463626685	0.530438463626685	0.518361528321442	unchanged
Meta_187	Azelaic acid	Lipids and lipid-like molecules	Fatty Acyls	Fatty acids and conjugates	HMDB0000784	C08261	-0.161666667	0.328719178014294	0.328719178014294	0.789101160448241	unchanged
Meta_188	3-Hydroxycapric acid	Organic acids and derivatives	Hydroxy acids and derivatives	Medium-chain hydroxy acids and derivatives	HMDB0002203		-0.063	0.611233866199414	0.611233866199414	0.406754064447696	unchanged
Meta_189	N-Acetyl-L-glutamic acid	Organic acids and derivatives	Carboxylic acids and derivatives	Amino acids, peptides, and analogues	HMDB0001138	C00624	-0.110333333	0.568915515588406	0.568915515588406	0.433511626539124	unchanged
Meta_191	4-Methylquinoline-2-carboxylic acid	Unclassified	Unclassified	Unclassified			0.992000000000001	0.0678156196501727	0.0678156196501727	1.15683664862889	unchanged
Meta_193	Pivagabine	Organic acids and derivatives	Carboxylic acids and derivatives	Amino acids, peptides, and analogues	HMDB0246575		0.0583333333333336	0.547527383267818	0.547527383267818	0.485163981739055	unchanged
Meta_197	Glycylleucine	Organic acids and derivatives	Carboxylic acids and derivatives	Amino acids, peptides, and analogues	HMDB0000759	C02155	-0.086333333	0.371687789925419	0.371687789925419	0.660622051893761	unchanged
Meta_198	Glycyl-Isoleucine	Organic acids and derivatives	Carboxylic acids and derivatives	Amino acids, peptides, and analogues	HMDB0028844		-0.318666667	0.0717765685212994	0.0717765685212994	1.15362913578996	unchanged
Meta_199	N-Acetyl-L-methionine	Organic acids and derivatives	Carboxylic acids and derivatives	Amino acids, peptides, and analogues	HMDB0011745	C02712	0.523	0.0565308581373066	0.0565308581373066	1.32574852115809	unchanged
Meta_2	Thiirane	Organoheterocyclic compounds	Thiiranes	Unclassified	HMDB0258994	C19419	0.101666666666667	0.873820459220471	0.873820459220471	0.110783276561705	unchanged
Meta_20	Morpholine	Organoheterocyclic compounds	Oxazinanes	Morpholines	HMDB0031581	C14452	-1.002666667	0.203166948051031	0.203166948051031	0.922734777901812	unchanged
Meta_201	Menadione	Benzenoids	Naphthalenes	Naphthoquinones	HMDB0001892	C05377	0.240666666666668	0.718594552990628	0.718594552990628	0.27319204915325	unchanged
Meta_202	Citric acid	Organic acids and derivatives	Carboxylic acids and derivatives	Tricarboxylic acids and derivatives	HMDB0000094	C00158	1.06633333333333	0.0579359110321352	0.0579359110321352	1.2827117859068	unchanged
Meta_203	Phenylacetylglycine	Organic acids and derivatives	Carboxylic acids and derivatives	Amino acids, peptides, and analogues	HMDB0000821	C05598	0.239333333333335	0.247291368180561	0.247291368180561	0.897177272375871	unchanged
Meta_204	Hydroxycotinine	Organoheterocyclic compounds	Pyridines and derivatives	Pyrrolidinylpyridines	HMDB0001390		-0.151333333	0.450476890707369	0.450476890707369	0.578474690731263	unchanged
Meta_205	N-Acetylleucine	Organic acids and derivatives	Carboxylic acids and derivatives	Amino acids, peptides, and analogues	HMDB0011756	C02710	-0.070333333	0.492752353729619	0.492752353729619	0.562467364785067	unchanged
Meta_206	1,3-Dihydro-(2H)-indol-2-one	Organoheterocyclic compounds	Indoles and derivatives	Indolines	HMDB0061918	C12312	-0.717	0.0973220221601618	0.0973220221601618	1.08793952615413	unchanged
Meta_207	Gluconic acid	Organic oxygen compounds	Organooxygen compounds	Carbohydrates and carbohydrate conjugates	HMDB0000625	C00257	-0.476	0.102763413235343	0.102763413235343	1.06810976263583	unchanged
Meta_208	Ethyl Phenylglyoxylate	Benzenoids	Benzene and substituted derivatives	Benzoyl derivatives	HMDB0062729		-0.28	0.0985851669700765	0.0985851669700765	1.10616178300748	unchanged
Meta_209	Tyrosine methylester	Organic acids and derivatives	Carboxylic acids and derivatives	Amino acids, peptides, and analogues	HMDB0029217		-0.394666667	0.205050106103156	0.205050106103156	0.909343155366847	unchanged
Meta_21	L-Lactic acid	Organic acids and derivatives	Hydroxy acids and derivatives	Alpha hydroxy acids and derivatives	HMDB0000190	C00186	-0.620333333	0.198471012697535	0.198471012697535	0.994540839188026	unchanged
Meta_210	2-Hydroxyadipic acid	Lipids and lipid-like molecules	Fatty Acyls	Fatty acids and conjugates	HMDB0000321	C02360	-0.169666667	0.345547248093385	0.345547248093385	0.765490936223249	unchanged

Meta_211	methyl 2-(2-amino-4-hydroxy-6-methylpyrimidin-5-yl)acetate	Unclassified	Unclassified	Unclassified			0.507000000000001	0.132120625554743	0.132120625554743	1.06695263496343	unchanged
Meta_213	AICA-riboside	Nucleosides, nucleotides, and analogues	Imidazole ribonucleosides and ribonucleotides	Unclassified	HMDB0062179		-0.341666667	0.055678390484556	0.055678390484556	1.18876200918528	unchanged
Meta_214	Sebacic acid	Lipids and lipid-like molecules	Fatty Acyls	Fatty acids and conjugates	HMDB0000792	C08277	-0.051666667	0.829441231182482	0.829441231182482	0.179232300585692	unchanged
Meta_215	Simazine	Organoheterocyclic compounds	Triazines	1,3,5-triazines	HMDB0258294	C11172	0.299333333333333	0.424895815349301	0.424895815349301	0.608535864079309	unchanged
Meta_217	Benzenesulfonic acid	Benzenoids	Benzene and substituted derivatives	Benzenesulfonic acids and derivatives	HMDB0248986		-0.312333333	0.121189909577249	0.121189909577249	1.03480724545205	unchanged
Meta_219	VASICINONE	Organoheterocyclic compounds	Diazanaphthalenes	Benzodiazines	HMDB0259770		-0.588333333	0.508940420489623	0.508940420489623	0.552417745181586	unchanged
Meta_22	Putrescine	Organic nitrogen compounds	Organonitrogen compounds	Amines	HMDB0001414	C00134	0.118666666666666	0.268880812636922	0.268880812636922	0.85322258300179	unchanged
Meta_220	Alanylleucine	Organic acids and derivatives	Carboxylic acids and derivatives	Amino acids, peptides, and analogues	HMDB0028691		-0.571333333	0.110220919690126	0.110220919690126	1.05523107509234	unchanged
Meta_221	Leucylalanine	Organic acids and derivatives	Carboxylic acids and derivatives	Amino acids, peptides, and analogues	HMDB0028922		-0.473	0.087414575682876	0.087414575682876	1.13575467870263	unchanged
Meta_222	Spermine	Organic nitrogen compounds	Organonitrogen compounds	Amines	HMDB0001256	C00750	-0.335666667	0.715169676524247	0.715169676524247	0.308660939077468	unchanged
Meta_224	Indolelactic acid	Organoheterocyclic compounds	Indoles and derivatives	Indolyl carboxylic acids and derivatives	HMDB0000671	C02043	-0.097	0.668912272574272	0.668912272574272	0.351319969292936	unchanged
Meta_225	3-Indolebutyric acid	Organoheterocyclic compounds	Indoles and derivatives	Indoles	HMDB0002096	C11284	0.753666666666666	0.11932837327194	0.11932837327194	1.1286335966208	unchanged
Meta_226	L-Acetylcarnitine	Lipids and lipid-like molecules	Fatty Acyls	Fatty acid esters	HMDB0000201	C02571	-0.024333333	0.936729938444653	0.936729938444653	0.0412034739074479	unchanged
Meta_227	N-(3-Methylbut-2-EN-1-YL)-9H-purin-6-amine	Organoheterocyclic compounds	Imidazopyrimidines	Purines and purine derivatives	HMDB0245646	C04083	-0.590666667	0.064555935329366	0.064555935329366	1.18103775054186	unchanged
Meta_228	Galactitol	Organic oxygen compounds	Organooxygen compounds	Carbohydrates and carbohydrate conjugates	HMDB0000107	C01697	-0.117666667	0.46986639507625	0.46986639507625	0.58642157072349	unchanged
Meta_23	beta-Alanine	Organic acids and derivatives	Carboxylic acids and derivatives	Amino acids, peptides, and analogues	HMDB0000056	C00099	-0.113666667	0.6252523189103	0.6252523189103	0.376884957780799	unchanged
Meta_230	Pyridoxine hydrochloride	Organoheterocyclic compounds	Pyridines and derivatives	Pyridoxines	HMDB0301760		-0.166333333	0.489477273034063	0.489477273034063	0.542333053674765	unchanged
Meta_232	1-Naphthalenesulfonic acid	Benzenoids	Naphthalenes	Naphthalene sulfonic acids and derivatives	HMDB0243955	C16201	-0.444333333	0.503304555634342	0.503304555634342	0.510156072355899	unchanged
Meta_233	Diethyl L-malate	Organic acids and derivatives	Hydroxy acids and derivatives	Beta hydroxy acids and derivatives	HMDB0040220		-0.251333333	0.11836016249517	0.11836016249517	1.09342980468384	unchanged
Meta_234	N-Acetylglutamine	Organic acids and derivatives	Carboxylic acids and derivatives	Amino acids, peptides, and analogues	HMDB0006029		0.0106666666666673	0.982642021810887	0.982642021810887	0.00749100043091146	unchanged
Meta_235	Hydrocinnamic acid	Phenylpropanoids and polyketides	Phenylpropanoic acids	Unclassified	HMDB0000764	C05629	-0.026333333	0.875088678550477	0.875088678550477	0.135254821312197	unchanged
Meta_237	Talipexole	Organoheterocyclic compounds	Azepines	Unclassified	HMDB0258691		0.0906666666666656	0.339628190243791	0.339628190243791	0.747366797909733	unchanged
Meta_238	Atipamezole	Benzenoids	Indanes	Unclassified	HMDB0248699		-0.107666667	0.836893431049717	0.836893431049717	0.185568558692979	unchanged
Meta_239	Varenicline	Organoheterocyclic compounds	Benzazepines	Unclassified	HMDB0015398		-0.343333333	0.0755188551196952	0.0755188551196952	1.16861268448323	unchanged
Meta_24	2-(Methylthio)ethanol	Organosulfur compounds	Thioethers	Dialkylthioethers	HMDB0032425		-0.031333333	0.90078234886171	0.90078234886171	0.111793066985168	unchanged
Meta_241	(+)-1,2-Epoxyneomenthyl acetate	Organoheterocyclic compounds	Oxepanes	Unclassified	HMDB0302747		-0.060666667	0.794894088301434	0.794894088301434	0.218966751449883	unchanged
Meta_242	3-Oxododecanoic acid	Organic acids and derivatives	Keto acids and derivatives	Medium-chain keto acids and derivatives	HMDB0010727	C02367	0.225	0.340038737757998	0.340038737757998	0.770800928765988	unchanged
Meta_243	1,3,5-Trinitrobenzene	Benzenoids	Benzene and substituted derivatives	Nitrobenzenes	HMDB0244187		0.404	0.0773201484867351	0.0773201484867351	1.2451112658036	unchanged
Meta_247	1-Pyrenemethanol	Benzenoids	Pyrenes	Unclassified	HMDB0244007		-0.078	0.847146238936218	0.847146238936218	0.150980723968176	unchanged
Meta_248	Undecanedioic acid	Lipids and lipid-like molecules	Fatty Acyls	Fatty acids and conjugates	HMDB0000888		-0.126666667	0.685792745968622	0.685792745968622	0.327428520444448	unchanged
Meta_249	3-Hydroxydodecanoic acid	Organic acids and derivatives	Hydroxy acids and derivatives	Medium-chain hydroxy acids and derivatives	HMDB0000387		0.363	0.214068560520667	0.214068560520667	0.895616053724879	unchanged
Meta_250	N-Formylphenylalanine	Organic acids and derivatives	Carboxylic acids and derivatives	Amino acids, peptides, and analogues	HMDB0240317		0.184999999999999	0.165286186478414	0.165286186478414	0.969268758900122	unchanged
Meta_252	Glyceraldehyde phosphate	Organic acids and derivatives	Organic phosphoric acids and	Unclassified	HMDB0252837		-0.143666667	0.470100952901515	0.470100952901515	0.56895214231763	unchanged
Meta_254	1-Methylpyrene	Benzenoids	Pyrenes	Unclassified	HMDB0243945		-0.616666667	0.333616339897572	0.333616339897572	0.779137903155979	unchanged
Meta_256	Valylvaline	Organic acids and derivatives	Carboxylic acids and derivatives	Amino acids, peptides, and analogues	HMDB0029140		-0.603666667	0.0595528024819667	0.0595528024819667	1.20718311692488	unchanged
Meta_257	1-Isothiocyanato-7-(methylsulfinyl)heptane	Organosulfur compounds	Sulfoxides	Unclassified	HMDB0038441		0.423333333333332	0.0692748110676446	0.0692748110676446	1.15772293200518	unchanged
Meta_258	Pantothenic acid	Organic oxygen compounds	Organooxygen compounds	Alcohols and polyols	HMDB0000210	C00864	0.166666666666668	0.320616296569388	0.320616296569388	0.76884240384543	unchanged
Meta_260	Bisphenol F	Benzenoids	Benzene and substituted derivatives	Diphenylmethanes	HMDB0240711	C14298	0.591333333333333	0.200237488721994	0.200237488721994	0.969166844104022	unchanged
Meta_261	Propionylcarnitine	Lipids and lipid-like molecules	Fatty Acyls	Fatty acid esters	HMDB0000824		0.355666666666664	0.161027229170292	0.161027229170292	1.0176731243811	unchanged
Meta_262	Alanylmethionine	Organic acids and derivatives	Carboxylic acids and derivatives	Amino acids, peptides, and analogues	HMDB0028693		0.319666666666667	0.365385866793795	0.365385866793795	0.720698297172853	unchanged
Meta_263	Methyl 2-amino-3-(1H-indol-3-yl)propanoate	Organic acids and derivatives	Carboxylic acids and derivatives	Amino acids, peptides, and analogues	HMDB0254563		0.222333333333333	0.197512257078324	0.197512257078324	0.902148388826978	unchanged
Meta_265	6-Chloro-3-cyano-7-methylchromone	Unclassified	Unclassified	Unclassified			0.122	0.711899592002838	0.711899592002838	0.302397843962086	unchanged
Meta_266	(5-Methyl-1,3-dioxo-1,3-dihydro-2H-isoindol-2-yl)acetic acid	Unclassified	Unclassified	Unclassified			0.481	0.401683408205917	0.401683408205917	0.65411011934836	unchanged
Meta_268	ID11614	Organoheterocyclic compounds	Diazinanes	Piperazines	HMDB0060827		-0.199	0.276126497011168	0.276126497011168	0.856999495566299	unchanged
Meta_269	Diethyl phthalic acid	Benzenoids	Benzene and substituted derivatives	Benzoic acids and derivatives	HMDB0094660	C14175	0.169666666666666	0.49521365798638	0.49521365798638	0.550437229298564	unchanged
Meta_27	Phenol	Benzenoids	Phenols	1-hydroxy-4-unsubstituted benzenoids	HMDB0000228	C15584; C00146	0.308333333333334	0.176766817825502	0.176766817825502	0.96358302109251	unchanged
Meta_270	5-Hydroxy-L-tryptophan	Organoheterocyclic compounds	Indoles and derivatives	Tryptamines and derivatives	HMDB0000472	C00643	0.312666666666665	0.508250515577556	0.508250515577556	0.536812856917404	unchanged
Meta_271	2,5-Di-tert-butylhydroquinone	Benzenoids	Benzene and substituted derivatives	Phenylpropanes	HMDB0245492		-0.085	0.74747229702403	0.74747229702403	0.262044406959036	unchanged
Meta_272	Methyl 4-chloro-1H-indole-3-acetate	Organoheterocyclic compounds	Indoles and derivatives	Indolyl carboxylic acids and derivatives	HMDB0032937		0.414000000000002	0.0880972807215707	0.0880972807215707	1.16959704604068	unchanged
Meta_273	L-Cystathionine	Organic acids and derivatives	Carboxylic acids and derivatives	Amino acids, peptides, and analogues	HMDB0000099	C02291	0.891333333333334	0.0551015758286215	0.0551015758286215	1.23763939440941	unchanged
Meta_275	Glycyl-Phenylalanine	Organic acids and derivatives	Carboxylic acids and derivatives	Amino acids, peptides, and analogues	HMDB0028848		-0.143	0.393948715102405	0.393948715102405	0.663238188138946	unchanged
Meta_277	Chlorquinaldol	Organoheterocyclic compounds	Quinolines and derivatives	Haloquinolines	HMDB0250139		0.123000000000001	0.623911980628258	0.623911980628258	0.401312595732389	unchanged
Meta_278	L-Glutamic acid 5-phosphate	Organic acids and derivatives	Carboxylic acids and derivatives	Amino acids, peptides, and analogues	HMDB0001228	C03287	0.306666666666667	0.226473578239625	0.226473578239625	0.882543297235612	unchanged
Meta_279	(R)-1,2-dimethyl-5,6-dihydroxy-tetrahydroisoquinoline	Organoheterocyclic compounds	Tetrahydroisoquinolines	Unclassified	HMDB0012484		-1.006333333	0.297902413858104	0.297902413858104	0.766442129115585	unchanged
Meta_28	Succinic anhydride	Organic acids and derivatives	Carboxylic acids and derivatives	Dicarboxylic acids and derivatives	HMDB0032523	C19524	-0.042666667	0.726098157835323	0.726098157835323	0.316223969279793	unchanged
Meta_281	Trifluoroacetic acid	Organic acids and derivatives	Carboxylic acids and derivatives	Alpha-halocarboxylic acids and derivatives	HMDB0014118		-0.34	0.0637963806241451	0.0637963806241451	1.16787783069805	unchanged
Meta_287	Pentadecylamine	Organic nitrogen compounds	Organonitrogen compounds	Amines	HMDB0243828		-0.393666667	0.446086128412505	0.446086128412505	0.590896210729431	unchanged
Meta_288	Ibudilast	Organoheterocyclic compounds	Pyrazolopyridines	Unclassified	HMDB0015614		0.0256666666666661	0.9325495425262	0.9325495425262	0.052193837837776	unchanged
Meta_289	Pro-Ile	Organic acids and derivatives	Carboxylic acids and derivatives	Amino acids, peptides, and analogues	HMDB0304810		-0.561666667	0.236866108461938	0.236866108461938	0.876730719866636	unchanged
Meta_29	2-Piperidinone	Organoheterocyclic compounds	Piperidines	Piperidinones	HMDB0011749		-0.4	0.121714900914462	0.121714900914462	1.07467110748375	unchanged

Meta_290	Dodecanedioic acid	Lipids and lipid-like molecules	Fatty Acyls	Fatty acids and conjugates	HMDB0000623	C02678	-0.089666667	0.626699331046939	0.626699331046939	0.392960195299348	unchanged
Meta_291	Pro-leu	Organic acids and derivatives	Carboxylic acids and derivatives	Amino acids, peptides, and analogues	HMDB0253028		-0.267	0.628557597982152	0.628557597982152	0.408138651100381	unchanged
Meta_292	Isoleucyl-Valine	Organic acids and derivatives	Carboxylic acids and derivatives	Amino acids, peptides, and analogues	HMDB0028920		-0.420666667	0.152823907568195	0.152823907568195	1.09415157995627	unchanged
Meta_294	Lauramine oxide	Unclassified	Unclassified	Unclassified			-0.092666667	0.346000821189941	0.346000821189941	0.746001244439538	unchanged
Meta_295	Cassiachromone	Organoheterocyclic compounds	Benzopyrans	1-benzopyrans	HMDB0030813		0.327999999999999	0.225951427	0.225951427	0.904544931172843	unchanged
Meta_296	Benzanthrone	Benzenoids	Phenanthrenes and derivatives	Unclassified	HMDB0248974		-0.324333333	0.586683923384903	0.586683923384903	0.430920607467782	unchanged
Meta_3	Urea	Organic acids and derivatives	Organic carbonic acids and derivatives	Ureas	HMDB0000294	C00086	-0.434666667	0.197534450370042	0.197534450370042	0.985454898891962	unchanged
Meta_30	Cyclohexylamine	Organic nitrogen compounds	Organonitrogen compounds	Cyclohexylamines	HMDB0031404	C00571	-0.204666667	0.0502958068125862	0.0502958068125862	1.2984369965158	unchanged
Meta_300	7-Diethylamino-4-methylcoumarin	Unclassified	Unclassified	Unclassified			0.625333333333332	0.246191740890546	0.246191740890546	0.847193662269151	unchanged
Meta_302	1-Methoxypyrene	Benzenoids	Pyrenes	Unclassified	HMDB0060324	C18259	-0.224333333	0.33644493487978	0.33644493487978	0.720668431567156	unchanged
Meta_305	Dehydrocurdione	Lipids and lipid-like molecules	Prenol lipids	Sesquiterpenoids	HMDB0035407	C16949	0.0386666666666677	0.818836003632886	0.818836003632886	0.210552097216722	unchanged
Meta_308	Cyheptamide	Benzenoids	Dibenzocycloheptenes	Unclassified	HMDB0250690		-0.060666667	0.65318885917393	0.65318885917393	0.352174570544735	unchanged
Meta_31	2-Ketobutyric acid	Organic acids and derivatives	Keto acids and derivatives	Short-chain keto acids and derivatives	HMDB0000005	C00109	1.109	0.151029069715158	0.151029069715158	1.01628883772068	unchanged
Meta_311	Alanylphenylalanine	Organic acids and derivatives	Carboxylic acids and derivatives	Amino acids, peptides, and analogues	HMDB0028694		-0.448333333	0.0576531912492897	0.0576531912492897	1.27105605581078	unchanged
Meta_312	1,4-Bisdesoxycyadox	Organoheterocyclic compounds	Diazanaphthalenes	Benzodiazines	HMDB0244200		-0.391333333	0.288132038024407	0.288132038024407	0.81617473817266	unchanged
Meta_315	Pimelic acid	Lipids and lipid-like molecules	Fatty Acyls	Fatty acids and conjugates	HMDB0000857	C02656	-0.242333333	0.124907607540669	0.124907607540669	1.04833204634381	unchanged
Meta_316	Pentaethylene glycol	Organic oxygen compounds	Organooxygen compounds	Ethers	HMDB0256256		-0.483666667	0.614708202796634	0.614708202796634	0.40136205673852	unchanged
Meta_317	5-Acetyl-1-benzylpyrrolidin-2-one	Unclassified	Unclassified	Unclassified			-0.327	0.19583044266835	0.19583044266835	0.95459496027177	unchanged
Meta_318	2,4,5,6-Phenanthrenetetrol	Benzenoids	Phenanthrenes and derivatives	Phenanthrols	HMDB0032866		-0.074333333	0.741678413205817	0.741678413205817	0.234313942737822	unchanged
Meta_319	Croscarmellose	Organic oxygen compounds	Organooxygen compounds	Carbohydrates and carbohydrate conjugates	HMDB0303567		0.162666666666667	0.496160796968934	0.496160796968934	0.518535934912912	unchanged
Meta_32	2-Aminoisobutyric acid	Organic acids and derivatives	Carboxylic acids and derivatives	Amino acids, peptides, and analogues	HMDB0001906	C03665	0.187666666666669	0.641496555425722	0.641496555425722	0.372468260522305	unchanged
Meta_321	3-Oxotetradecanoic acid	Lipids and lipid-like molecules	Fatty Acyls	Fatty acids and conjugates	HMDB0010730		0.466333333333333	0.314440811052766	0.314440811052766	0.774786507093308	unchanged
Meta_322	N-Undecanoylglycine	Organic acids and derivatives	Carboxylic acids and derivatives	Amino acids, peptides, and analogues	HMDB0013286		-0.326666667	0.124874911359027	0.124874911359027	1.04760700169615	unchanged
Meta_323	Canthin-6-one	Alkaloids and derivatives	Indolonaphthyridine alkaloids	Unclassified	HMDB0249593	C09098	1.08633333333333	0.140151430119922	0.140151430119922	1.12173512869791	unchanged
Meta_324	Pseudouridine	Nucleosides, nucleotides, and	Nucleoside and nucleotide analogues	Unclassified	HMDB0000767	C02067	-0.816666667	0.0546543213753292	0.0546543213753292	1.22888024268084	unchanged
Meta_325	Lumichrome	Organoheterocyclic compounds	Pteridines and derivatives	Alloxazines and isoalloxazines	HMDB0254199	C01727	0.0360000000000014	0.859505273054656	0.859505273054656	0.14096566083355	unchanged
Meta_326	Pyroglutamyl isoleucine	Unclassified	Unclassified	Unclassified			0.0990000000000002	0.583618223364635	0.583618223364635	0.468039129961904	unchanged
Meta_327	1,11-Undecanedicarboxylic acid	Lipids and lipid-like molecules	Fatty Acyls	Fatty acids and conjugates	HMDB0002327		0.274333333333335	0.374968574636219	0.374968574636219	0.655547029838269	unchanged
Meta_328	Falcarinone	Organic oxygen compounds	Organooxygen compounds	Carbonyl compounds	HMDB0033693	C08445	-0.659666667	0.0717138481920076	0.0717138481920076	1.1436065231825	unchanged
Meta_329	(R)-3-Hydroxy-tetradecanoic acid	Lipids and lipid-like molecules	Fatty Acyls	Fatty acids and conjugates	HMDB0010731		0.132999999999999	0.544404935913879	0.544404935913879	0.498922892089705	unchanged
Meta_33	L-Homoserine	Organic acids and derivatives	Carboxylic acids and derivatives	Amino acids, peptides, and analogues	HMDB0000719	C00263	0.274333333333331	0.177033058075358	0.177033058075358	0.984183159763282	unchanged
Meta_330	N-Acetylmannosamine	Organic oxygen compounds	Organooxygen compounds	Carbohydrates and carbohydrate conjugates	HMDB0001129	C00645	0.213333333333333	0.405655067741454	0.405655067741454	0.624162793384289	unchanged
Meta_331	Isopentenyl pyrophosphate	Lipids and lipid-like molecules	Prenol lipids	Isoprenoid phosphates	HMDB0001347	C00129	-0.324333333	0.106267428119545	0.106267428119545	1.14222125321425	unchanged
Meta_332	Glycerophosphoglycerol	Organic acids and derivatives	Organic phosphoric acids and	Phosphate esters	HMDB0240316		0.233333333333331	0.708189555582119	0.708189555582119	0.295674531788079	unchanged
Meta_333	Isoleucyl-Leucine	Organic acids and derivatives	Carboxylic acids and derivatives	Amino acids, peptides, and analogues	HMDB0028911		-0.744666667	0.0696599727860012	0.0696599727860012	1.23722710596266	unchanged
Meta_334	Leucylleucine	Organic acids and derivatives	Carboxylic acids and derivatives	Amino acids, peptides, and analogues	HMDB0028933	C11332	-0.688	0.215552966920993	0.215552966920993	0.879012502071883	unchanged
Meta_335	Tetraacetylethylenediamine	Organic acids and derivatives	Carboxylic acids and derivatives	Carboxylic acid derivatives	HMDB0040573		-1.01	0.0942128002723796	0.0942128002723796	1.13234182857676	unchanged
Meta_336	Bisphenol A	Benzenoids	Benzene and substituted derivatives	Diphenylmethanes	HMDB0032133	C13624	0.531666666666666	0.21086392213377	0.21086392213377	0.98184574154775	unchanged
Meta_338	Calcium galactarate	Organic oxygen compounds	Organooxygen compounds	Carbohydrates and carbohydrate conjugates	HMDB0303726		-0.060333333	0.704473042764756	0.704473042764756	0.319540862094201	unchanged
Meta_34	alpha-Hydroxyisobutyric acid	Organic acids and derivatives	Hydroxy acids and derivatives	Alpha hydroxy acids and derivatives	HMDB0000729		0.555333333333334	0.0682585225374049	0.0682585225374049	1.2380951676607	unchanged
Meta_341	Aspartyl-Leucine	Organic acids and derivatives	Carboxylic acids and derivatives	Amino acids, peptides, and analogues	HMDB0028757		-0.164666667	0.367247969177149	0.367247969177149	0.704882479591166	unchanged
Meta_342	8-hydroxy-3,6,9-trimethyl-3a,4,5,9b-tetrahydro-3H-benzo[g][1]benzofuran-2-one	Unclassified	Unclassified	Unclassified			-0.359333333	0.0682735431240501	0.0682735431240501	1.16573750579332	unchanged
Meta_344	3-Hydroxybutyrylcarnitine	Lipids and lipid-like molecules	Fatty Acyls	Fatty acid esters	HMDB0013127		0.128666666666668	0.719293329266716	0.719293329266716	0.296980116664242	unchanged
Meta_345	3,4-Dichloroisocoumarin	Phenylpropanoids and polyketides	Isocoumarins and derivatives	Unclassified	HMDB0246026		0.620999999999999	0.0566493663695258	0.0566493663695258	1.21520897117388	unchanged
Meta_346	2,3 or 10-Mercaptopinane	Lipids and lipid-like molecules	Prenol lipids	Monoterpenoids	HMDB0032377		-0.168666667	0.668892362388122	0.668892362388122	0.355460338739575	unchanged
Meta_348	2,6-Di-tert-butyl-4-nitrophenol	Unclassified	Unclassified	Unclassified			0.101666666666667	0.428332142544922	0.428332142544922	0.639577186407396	unchanged
Meta_350	L-Serine	Organic acids and derivatives	Carboxylic acids and derivatives	Amino acids, peptides, and analogues	HMDB0000187	C00065	-0.142666667	0.413644885470301	0.413644885470301	0.65719094476513	unchanged
Meta_352	Ritalinic acid	Organic nitrogen compounds	Organonitrogen compounds	Amines	HMDB0042008		-0.417333333	0.478905792586902	0.478905792586902	0.538551164522456	unchanged
Meta_353	cis-4-Carboxymethylenebut-2-en-4-olide	Organoheterocyclic compounds	Dihydrofurans	Furanones	HMDB0060459	C04431	-0.095	0.488103689023034	0.488103689023034	0.549143266313604	unchanged
Meta_354	Serylphenylalanine	Organic acids and derivatives	Carboxylic acids and derivatives	Amino acids, peptides, and analogues	HMDB0029046		-0.422	0.207771462328369	0.207771462328369	0.888039481341976	unchanged
Meta_355	Methyl (S)-2-Methylbutanoate	Lipids and lipid-like molecules	Fatty Acyls	Fatty acid esters	HMDB0029762		-0.051666667	0.870913630671388	0.870913630671388	0.154308596206775	unchanged
Meta_356	Palmitoleic acid	Lipids and lipid-like molecules	Fatty Acyls	Fatty acids and conjugates	HMDB0003229	C08362	-1.840333333	0.0890264112653744	0.0890264112653744	1.19148937971981	unchanged
Meta_357	6-Chloro-4-hydroxy-7-methyl-3-nitrocoumarin	Unclassified	Unclassified	Unclassified			-0.206333333	0.531409800875031	0.531409800875031	0.493620618395221	unchanged
Meta_358	Ketorolac	Organic oxygen compounds	Organooxygen compounds	Carbonyl compounds	HMDB0014608	C07062	-1.685333333	0.111478820116997	0.111478820116997	1.06038977843824	unchanged
Meta_36	Choline	Organic nitrogen compounds	Organonitrogen compounds	Quaternary ammonium salts	HMDB0000097	C00114	-0.411666667	0.263782325351476	0.263782325351476	0.82093237005686	unchanged
Meta_360	5-Methoxy-3,4-methylenedioxyamphetamine	Organoheterocyclic compounds	Benzodioxoles	Unclassified	HMDB0254817		-0.147333333	0.592166479664291	0.592166479664291	0.422741380045291	unchanged
Meta_361	O-Demethylmetoprolol	Benzenoids	Phenols	Tyrosols and derivatives	HMDB0255850		-0.32	0.187662210185503	0.187662210185503	0.96566402141025	unchanged
Meta_362	Liquiritigenin	Phenylpropanoids and polyketides	Flavonoids	Flavans	HMDB0029519	C09762	-0.107	0.363788585619648	0.363788585619648	0.721187880906776	unchanged
Meta_363	7,12-Dimethylbenz[a]anthracene	Benzenoids	Phenanthrenes and derivatives	Unclassified	HMDB0062429	C19488	-0.199333333	0.673595136522776	0.673595136522776	0.360014977744198	unchanged
Meta_365	Palmitic acid	Lipids and lipid-like molecules	Fatty Acyls	Fatty acids and conjugates	HMDB0000220	C00249	0.451000000000001	0.0549727590943763	0.0549727590943763	1.18971360005953	unchanged
Meta_366	Palmitic amide	Lipids and lipid-like molecules	Fatty Acyls	Fatty amides	HMDB0012273		-2.359666667	0.0647684401526135	0.0647684401526135	1.20673008083964	unchanged
Meta_367	Alternariol	Phenylpropanoids and polyketides	Coumarins and derivatives	Unclassified	HMDB0030831	C16838	0.0606666666666662	0.517619880617651	0.517619880617651	0.504359183543302	unchanged
Meta_368	2'-O-Methyluridine	Nucleosides, nucleotides, and	Pyrimidine nucleosides	Unclassified	HMDB0240328		-0.153	0.450438593619853	0.450438593619853	0.571930078867457	unchanged
Meta_369	Lumiflavin	Organoheterocyclic compounds	Pteridines and derivatives	Alloxazines and isoalloxazines	HMDB0254201		1.09066666666667	0.0551162629018779	0.0551162629018779	1.28764274611857	unchanged
Meta_370	Tetradecanedioic acid	Lipids and lipid-like molecules	Fatty Acyls	Fatty acids and conjugates	HMDB0000872		0.0793333333333344	0.849831989865517	0.849831989865517	0.145698089895813	unchanged

Meta_372	Mannose 6-phosphate	Organic oxygen compounds	Organooxygen compounds	Carbohydrates and carbohydrate conjugates	HMDB0001078		-0.401333333	0.334404234420001	0.334404234420001	0.744278899768134	unchanged
Meta_376	Glutaminylisoleucine	Organic acids and derivatives	Carboxylic acids and derivatives	Amino acids, peptides, and analogues	HMDB0028800		-0.512333333	0.178058487627128	0.178058487627128	0.963985110730065	unchanged
Meta_378	Mannitol 1-phosphate	Organic oxygen compounds	Organooxygen compounds	Carbohydrates and carbohydrate conjugates	HMDB0001530	C00644	0.405333333333335	0.0612940064180637	0.0612940064180637	1.17554983580875	unchanged
Meta_379	Celereoin	Phenylpropanoids and polyketides	Coumarins and derivatives	Furanocoumarins	HMDB0039027		0.931333333333335	0.0945437291015907	0.0945437291015907	1.17198647082493	unchanged
Meta_38	4-Hydroxybenzaldehyde	Organic oxygen compounds	Organooxygen compounds	Carbonyl compounds	HMDB0011718	C00633	-0.023333333	0.817462815777859	0.817462815777859	0.194578827083653	unchanged
Meta_380	2-(4-oxoquinazolin-3(4H)-yl)ethyl isobutyrate	Unclassified	Unclassified	Unclassified			-0.537666667	0.460274619020273	0.460274619020273	0.575574094326239	unchanged
Meta_381	Imazapyr	Organic acids and derivatives	Carboxylic acids and derivatives	Amino acids, peptides, and analogues	HMDB0253405	C18864	-0.521666667	0.0643284568342504	0.0643284568342504	1.247515959	unchanged
Meta_382	3-Hydroxyisovalerylcarnitine	Lipids and lipid-like molecules	Fatty Acyls	Fatty acid esters	HMDB0061189		-0.617666667	0.286629091302657	0.286629091302657	0.770284283138449	unchanged
Meta_383	methyl 2-[(4-methyl-2-oxo-2H-chromen-7-yl)oxy]propanoate	Unclassified	Unclassified	Unclassified			-0.047	0.851035965309035	0.851035965309035	0.123839113180409	unchanged
Meta_384	Phosphatidylglycerol	Lipids and lipid-like molecules	Glycerophospholipids	Glycerophosphates	HMDB0302468		0.0806666666666676	0.791895628329157	0.791895628329157	0.194728990093917	unchanged
Meta_389	Valylphenylalanine	Organic acids and derivatives	Carboxylic acids and derivatives	Amino acids, peptides, and analogues	HMDB0029134		-0.333666667	0.350501954166374	0.350501954166374	0.741353532126983	unchanged
Meta_391	3'-HPPH	Organoheterocyclic compounds	Azolidines	Imidazolidines	HMDB0013896		-0.274333333	0.0834411455713151	0.0834411455713151	1.16458765563738	unchanged
Meta_392	Inosine	Nucleosides, nucleotides, and	Purine nucleosides	Unclassified	HMDB0000195	C00294	0.233999999999998	0.170443635472914	0.170443635472914	0.953489948158939	unchanged
Meta_396	3-Oxohexadecanoic acid	Lipids and lipid-like molecules	Fatty Acyls	Fatty acids and conjugates	HMDB0010733		0.341333333333333	0.133146739702708	0.133146739702708	1.03402582270889	unchanged
Meta_397	Pemirolast	Organoheterocyclic compounds	Pyridopyrimidines	Unclassified	HMDB0015023		-0.225666667	0.0508889611611872	0.0508889611611872	1.21087954363611	unchanged
Meta_398	Octadecylamine	Organic nitrogen compounds	Organonitrogen compounds	Amines	HMDB0029586		-0.020333333	0.958602734599443	0.958602734599443	0.0531864529210822	unchanged
Meta_4	Trifluoromethane	Organohalogen compounds	Alkyl halides	Halomethanes	HMDB0252378		-0.11	0.656833871988017	0.656833871988017	0.341219952659393	unchanged
Meta_40	m-Cresol	Benzenoids	Phenols	Cresols	HMDB0002048	C01467	-0.125333333	0.141567970790919	0.141567970790919	1.11091917812431	unchanged
Meta_400	lysine phosphoester	Organic acids and derivatives	Carboxylic acids and derivatives	Amino acids, peptides, and analogues	HMDB0254265		0.292333333333332	0.215192477535587	0.215192477535587	0.876136457104175	unchanged
Meta_401	2-(4-Fluorophenyl)-3-hydroxy-7-methylchromen-4-one	Unclassified	Unclassified	Unclassified			0.41	0.623907477894141	0.623907477894141	0.41189121840629	unchanged
Meta_403	2-Hydroxyhexadecanoic acid	Lipids and lipid-like molecules	Fatty Acyls	Fatty acids and conjugates	HMDB0031057		-0.287	0.323025903741513	0.323025903741513	0.762919318926711	unchanged
Meta_404	16-Hydroxyhexadecanoic acid	Lipids and lipid-like molecules	Fatty Acyls	Fatty acids and conjugates	HMDB0006294	C18218	-0.467666667	0.665890984194598	0.665890984194598	0.326638995156773	unchanged
Meta_405	L-Thyronine	Organic acids and derivatives	Carboxylic acids and derivatives	Amino acids, peptides, and analogues	HMDB0000667		-0.374333333	0.471933083161656	0.471933083161656	0.585822625296284	unchanged
Meta_406	Hept-3-enoylcarnitine	Lipids and lipid-like molecules	Fatty Acyls	Fatty acid esters	HMDB0241681		-1.618666667	0.164677023519771	0.164677023519771	1.07934301231907	unchanged
Meta_407	N-(2-hydroxyethyl)tetradecanamide	Unclassified	Unclassified	Unclassified			0.611000000000001	0.086234410685677	0.086234410685677	1.12901656889213	unchanged
Meta_408	beta-naphthoflavone	Phenylpropanoids and polyketides	Flavonoids	Flavones	HMDB0249144		0.323666666666664	0.140722580316525	0.140722580316525	1.03023891551382	unchanged
Meta_409	Imazamethabenz	Organic acids and derivatives	Carboxylic acids and derivatives	Amino acids, peptides, and analogues	HMDB0034885		0.0650000000000013	0.821935105551481	0.821935105551481	0.166077899845797	unchanged
Meta_41	2-Acetyloxazole	Organic oxygen compounds	Organooxygen compounds	Carbonyl compounds	HMDB0038175		0.846666666666666	0.192078303337151	0.192078303337151	1.03456483706603	unchanged
Meta_410	3-Hydroxylidocaine	Organic acids and derivatives	Carboxylic acids and derivatives	Amino acids, peptides, and analogues	HMDB0060655	C16560	-0.016333333	0.938439974816327	0.938439974816327	0.0636939454839706	unchanged
Meta_412	Lorcaserin sulfamate	Organoheterocyclic compounds	Benzazepines	Unclassified	HMDB0061153		-0.036333333	0.663675552459158	0.663675552459158	0.366353966599987	unchanged
Meta_413	Cordycepin	Nucleosides, nucleotides, and	Purine nucleosides	Purine 3'-deoxyribonucleosides	HMDB0250451		0.0760000000000005	0.471658407764948	0.471658407764948	0.555713086611374	unchanged
Meta_416	Bakers yeast extract	Benzenoids	Fluorenes	Unclassified	HMDB0032173		-0.144666667	0.673964910184162	0.673964910184162	0.344802835154975	unchanged
Meta_417	Cyclobrassinin	Organoheterocyclic compounds	Indoles and derivatives	Indoles	HMDB0033352		-0.545333333	0.144568669310993	0.144568669310993	1.03673498117817	unchanged
Meta_42	Methylphosphate	Organic acids and derivatives	Organic phosphoric acids and	Phosphate esters	HMDB0061711		-0.316	0.115282201718549	0.115282201718549	1.05868183550234	unchanged
Meta_421	Fenthion	Organic acids and derivatives	Organic thiophosphoric acids and derivatives	Thiophosphoric acid esters	HMDB0033209	C14420	-0.005333333	0.974504568326062	0.974504568326062	0.0387326998734105	unchanged
Meta_422	Pyr-phe-OH	Unclassified	Unclassified	Unclassified			2.65066666666667	0.0555794372839582	0.0555794372839582	1.33057958302929	unchanged
Meta_423	Pantetheine	Organic acids and derivatives	Carboxylic acids and derivatives	Amino acids, peptides, and analogues	HMDB0003426	C00831	-0.520666667	0.275751767963098	0.275751767963098	0.842558935947583	unchanged
Meta_425	Longistylin C	Phenylpropanoids and polyketides	Stilbenes	Unclassified	HMDB0038544		-0.474333333	0.250025478106396	0.250025478106396	0.86615230752585	unchanged
Meta_426	Fexinidazole	Organoheterocyclic compounds	Azoles	Imidazoles	HMDB0252240		0.168333333333333	0.32973738987707	0.32973738987707	0.734526297793501	unchanged
Meta_427	1-Benzyl-3-ethyl-4-hydroxyquinolin-2-one	Unclassified	Unclassified	Unclassified			-0.34	0.0872550490251435	0.0872550490251435	1.1229592516012	unchanged
Meta_429	(E)-2-(Hydroxymethyl)-3-[(1S,2R,5S)-5-(hydroxymethyl)-2-propan-2-ylcyclohexyl]prop-2-enoic acid	Unclassified	Unclassified	Unclassified			-0.557666667	0.0672301134199591	0.0672301134199591	1.22897799192979	unchanged
Meta_43	3-Furoic acid	Organoheterocyclic compounds	Furans	Furoic acid and derivatives	HMDB0000444		1.576	0.0520606480628411	0.0520606480628411	1.24940626277763	unchanged
Meta_430	Dibutyl phthalate	Benzenoids	Benzene and substituted derivatives	Benzoic acids and derivatives	HMDB0033244	C14214	0.712	0.131210371351316	0.131210371351316	1.09908379332003	unchanged
Meta_431	Phenylalanylleucine	Organic acids and derivatives	Carboxylic acids and derivatives	Amino acids, peptides, and analogues	HMDB0253025		-0.633	0.292881642014184	0.292881642014184	0.836516910129224	unchanged
Meta_432	Leucylphenylalanine	Organic acids and derivatives	Carboxylic acids and derivatives	Amino acids, peptides, and analogues	HMDB0013243		-0.409333333	0.40913353786215	0.40913353786215	0.626030435703172	unchanged
Meta_433	Glycerophosphocholine	Lipids and lipid-like molecules	Glycerophospholipids	Glycerophosphocholines	HMDB0000086	C00670	-0.142333333	0.688059478546842	0.688059478546842	0.303610250025758	unchanged
Meta_434	Asparaginyl-Phenylalanine	Organic acids and derivatives	Carboxylic acids and derivatives	Amino acids, peptides, and analogues	HMDB0028738		-0.313	0.184486931493013	0.184486931493013	0.924206655948899	unchanged
Meta_436	Hexadecenal	Lipids and lipid-like molecules	Fatty Acyls	Fatty aldehydes	HMDB0060482	C06123	-0.206333333	0.388972766877629	0.388972766877629	0.673070015784962	unchanged
Meta_438	Methyl 5-(2-oxohexahydro-1H-thieno[3,4-d]imidazol-4-yl)pentanoate	Unclassified	Unclassified	Unclassified			1.88933333333333	0.425126147825132	0.425126147825132	0.621219921831832	unchanged
Meta_439	L-beta-aspartyl-L-phenylalanine	Organic acids and derivatives	Peptidomimetics	Hybrid peptides	HMDB0011167		-0.313	0.346141291575673	0.346141291575673	0.75019467793178	unchanged
Meta_44	Uracil	Organoheterocyclic compounds	Diazines	Pyrimidines and pyrimidine derivatives	HMDB0000300	C00106	-0.453333333	0.128770986153999	0.128770986153999	1.02634402550372	unchanged
Meta_444	Oleamide	Lipids and lipid-like molecules	Fatty Acyls	Fatty amides	HMDB0002117	C19670	-0.354333333	0.0982169059302085	0.0982169059302085	1.22409398059083	unchanged
Meta_446	Myristoylglycine	Organic acids and derivatives	Carboxylic acids and derivatives	Amino acids, peptides, and analogues	HMDB0013250		-0.028	0.908875287804216	0.908875287804216	0.104588259183746	unchanged
Meta_447	Altretamine	Organic nitrogen compounds	Organonitrogen compounds	Amines	HMDB0014631		-2.593	0.0920158464862901	0.0920158464862901	1.15132228901984	unchanged
Meta_448	Isovalerylalanine	Organic acids and derivatives	Carboxylic acids and derivatives	Amino acids, peptides, and analogues	HMDB0000747		0.233999999999998	0.128988309374246	0.128988309374246	1.07189740073649	unchanged
Meta_449	N-(2-Hydroxyethyl)pentadecanamide	Unclassified	Unclassified	Unclassified			-0.009	0.919779437075926	0.919779437075926	0.103682923739667	unchanged
Meta_453	Lauroyl diethanolamide	Lipids and lipid-like molecules	Fatty Acyls	Fatty amides	HMDB0032358		-0.401333333	0.0631747117967033	0.0631747117967033	1.19932445520624	unchanged
Meta_454	C17-Sphinganine	Unclassified	Unclassified	Unclassified			0.233000000000001	0.261671597985263	0.261671597985263	0.808115507549307	unchanged
Meta_455	Diazoxon	Organic acids and derivatives	Organic phosphoric acids and	Phosphate esters	HMDB0251156		0.968333333333334	0.126077936451845	0.126077936451845	1.16024978633568	unchanged
Meta_458	Androsterone	Lipids and lipid-like molecules	Steroids and steroid derivatives	Androstane steroids	HMDB0000031	C00523	0.0410000000000004	0.764482999581594	0.764482999581594	0.236851386677568	unchanged
Meta_46	3-Hydroxyproline	Organic acids and derivatives	Carboxylic acids and derivatives	Amino acids, peptides, and analogues	HMDB0245903		0.0463333333333331	0.889162029445213	0.889162029445213	0.119924278770736	unchanged
Meta_460	Gingerol	Benzenoids	Phenols	Methoxyphenols	HMDB0005783	C10462	-0.03	0.87722864367868	0.87722864367868	0.115647391466364	unchanged

Meta_461	Brassica oleracea Alkaloid	Organic acids and derivatives	Carboxylic acids and derivatives	Amino acids, peptides, and analogues	HMDB0033602		-0.500333333	0.0743485868242243	0.0743485868242243	1.26702073454318	unchanged
Meta_465	Isoleucyl-Cysteine	Organic acids and derivatives	Carboxylic acids and derivatives	Amino acids, peptides, and analogues	HMDB0028904		-0.262333333	0.425302841151946	0.425302841151946	0.6267076477168	unchanged
Meta_466	Tyrosyl-Leucine	Organic acids and derivatives	Carboxylic acids and derivatives	Amino acids, peptides, and analogues	HMDB0029109		-0.278333333	0.272209005463418	0.272209005463418	0.842733957274579	unchanged
Meta_467	2'-O-Methylguanosine	Unclassified	Unclassified	Unclassified			-0.691333333	0.117856995365348	0.117856995365348	1.06425266075578	unchanged
Meta_468	(9Z,12Z)-N-(2-hydroxyethyl)hexadeca-9,12-dienamide	Unclassified	Unclassified	Unclassified			-0.060333333	0.803454334790606	0.803454334790606	0.223098573871835	unchanged
Meta_469	3-Oxooctadecanoic acid	Lipids and lipid-like molecules	Fatty Acyls	Fatty acids and conjugates	HMDB0010736		0.189333333333334	0.711445117669204	0.711445117669204	0.305508954376794	unchanged
Meta_47	Acetylenedicarboxylic acid	Organic acids and derivatives	Carboxylic acids and derivatives	Dicarboxylic acids and derivatives	HMDB0247933	C03248	-0.286666667	0.111256458161167	0.111256458161167	1.19216953527693	unchanged
Meta_470	Ricinoleic acid	Lipids and lipid-like molecules	Fatty Acyls	Fatty acids and conjugates	HMDB0034297	C08365	-0.190333333	0.56516173726226	0.56516173726226	0.470167570678808	unchanged
Meta_471	5'-Methylthioadenosine	Nucleosides, nucleotides, and	5'-deoxyribonucleosides	5'-deoxy-5'-thionucleosides	HMDB0001173	C00170	-0.323	0.33789577159715	0.33789577159715	0.778837182103465	unchanged
Meta_472	3'-O-Methylguanosine	Nucleosides, nucleotides, and	Purine nucleosides	Unclassified	HMDB0006038		-0.571333333	0.0953169536845982	0.0953169536845982	1.21330116378422	unchanged
Meta_474	N,N-Dimethyloctadecylamine	Organic nitrogen compounds	Organonitrogen compounds	Amines	HMDB0255266		0.274666666666665	0.6089552181836	0.6089552181836	0.397949632720558	unchanged
Meta_475	5,7-Dihydroxy-4'-methoxy-8-methylflavanone	Phenylpropanoids and polyketides	Flavonoids	O-methylated flavonoids	HMDB0041321		0.299999999999999	0.218633615365606	0.218633615365606	0.897817061369354	unchanged
Meta_476	12-Hydroxystearic acid	Lipids and lipid-like molecules	Fatty Acyls	Fatty acids and conjugates	HMDB0061706		-0.202333333	0.400528332302582	0.400528332302582	0.626004966312284	unchanged
Meta_477	2-Pyrrolidineacetic acid	Organoheterocyclic compounds	Pyrrolidines	Unclassified	HMDB0029444		-0.349	0.429188273763161	0.429188273763161	0.599234056500526	unchanged
Meta_479	Oxadixyl	Unclassified	Unclassified	Unclassified		C18753	-0.447333333	0.308294317470817	0.308294317470817	0.757732732792395	unchanged
Meta_48	Creatinine	Organic acids and derivatives	Carboxylic acids and derivatives	Amino acids, peptides, and analogues	HMDB0000562	C00791	0.0199999999999996	0.885539489983353	0.885539489983353	0.118243793275989	unchanged
Meta_480	Arginylglutamine	Organic acids and derivatives	Carboxylic acids and derivatives	Amino acids, peptides, and analogues	HMDB0028707		0.285333333333334	0.197424466095272	0.197424466095272	0.916108669560602	unchanged
Meta_481	""""\4-hydroxy-5,7-dimethyl-3-benzylhydroquinolin-2-one CollisionEnergy:102040\""""""""""""""""	Unclassified	Unclassified	Unclassified			-0.373	0.111091763715949	0.111091763715949	1.09608372201405	unchanged
Meta_482	(4-oxido-2,3,5,6,7,8-hexahydro-1H-pyrrolizin-4-ium-1-yl)methyl 2-hydroxy-2-(1-hydroxyethyl)-3-methylbutanoate	Unclassified	Unclassified	Unclassified			0.697333333333335	0.188277679446113	0.188277679446113	0.952388478454613	unchanged
Meta_484	N-Acetylaspartylglutamic acid	Organic acids and derivatives	Carboxylic acids and derivatives	Amino acids, peptides, and analogues	HMDB0001067	C12270	0.437333333333335	0.215644445609953	0.215644445609953	0.951058804568371	unchanged
Meta_485	Eicosapentaenoic acid	Lipids and lipid-like molecules	Fatty Acyls	Fatty acids and conjugates	HMDB0001999	C06428	1.841	0.0987378920996237	0.0987378920996237	1.21067521089137	unchanged
Meta_486	N-Acetyldehydroanonaine	Alkaloids and derivatives	Aporphines	Unclassified	HMDB0041537		0.774666666666667	0.0841378057714636	0.0841378057714636	1.14778475890975	unchanged
Meta_487	Benzododecinium	Unclassified	Unclassified	Unclassified			0.0426666666666655	0.801876479819867	0.801876479819867	0.188688472795691	unchanged
Meta_488	4-Oxo-1,2,3,4-tetrahydrocyclopenta[c]chromen-7-yl benzoate	Unclassified	Unclassified	Unclassified			-0.203333333	0.373966089523383	0.373966089523383	0.70227758115098	unchanged
Meta_489	Hexaethylene glycol	Organic oxygen compounds	Organooxygen compounds	Ethers	HMDB0061822		-0.038666667	0.847552749595699	0.847552749595699	0.163687484325124	unchanged
Meta_49	Butanal	Organic oxygen compounds	Organooxygen compounds	Carbonyl compounds	HMDB0003543	C01412	-0.284666667	0.0884170368304022	0.0884170368304022	1.18192183248516	unchanged
Meta_490	Glutathione	Organic acids and derivatives	Carboxylic acids and derivatives	Amino acids, peptides, and analogues	HMDB0000125	C00051	-0.325	0.531078323392601	0.531078323392601	0.509464945114497	unchanged
Meta_491	3-Hydroxyimino-10,13-dimethyl-1,2,4,5,6,7,8,9,11,12,14,15,16,17-tetradecahydrocyclopenta[a]phenanthren-17-ol	Unclassified	Unclassified	Unclassified			-0.348333333	0.119485333754486	0.119485333754486	1.05374799934531	unchanged
Meta_492	Oxidized glutathione	Organic acids and derivatives	Carboxylic acids and derivatives	Amino acids, peptides, and analogues	HMDB0003337	C00127	0.696333333333335	0.216917997157938	0.216917997157938	0.905829245047436	unchanged
Meta_493	N-Acetylneuraminic acid	Organic oxygen compounds	Organooxygen compounds	Carbohydrates and carbohydrate conjugates	HMDB0000230	C19910	-0.711	0.0699233172484235	0.0699233172484235	1.16181831909515	unchanged
Meta_494	methyl 2-ethyl-4-[(3R,4R,5S)-5-hydroxy-4,5-dimethyl-2-oxooxolan-3-yl]-2-methyl-3-oxobutanoate	Unclassified	Unclassified	Unclassified			0.285333333333334	0.308766094252944	0.308766094252944	0.745535710717085	unchanged
Meta_495	Melophlin M	Unclassified	Unclassified	Unclassified			0.059333333333334	0.823636139960208	0.823636139960208	0.16085008935981	unchanged
Meta_496	Oleyl alcohol	Lipids and lipid-like molecules	Fatty Acyls	Fatty alcohols	HMDB0029632		-0.634666667	0.324698588813632	0.324698588813632	0.775090900775089	unchanged
Meta_499	2-hydroxyethinylestradiol	Lipids and lipid-like molecules	Steroids and steroid derivatives	Estrane steroids	HMDB0061027		-0.550666667	0.254843884957076	0.254843884957076	0.844938112506845	unchanged
Meta_500	Mestranol	Lipids and lipid-like molecules	Steroids and steroid derivatives	Estrane steroids	HMDB0015446	C07618	0.181666666666667	0.715539870342248	0.715539870342248	0.26563700366463	unchanged
Meta_501	Phenylalanylphenylalanine	Organic acids and derivatives	Carboxylic acids and derivatives	Amino acids, peptides, and analogues	HMDB0013302		-1.462	0.0728281130763864	0.0728281130763864	1.18159702772731	unchanged
Meta_502	3-Palmitoyl-sn-glycerol	Lipids and lipid-like molecules	Glycerolipids	Monoradylglycerols	HMDB0245964		0.725333333333333	0.0795601774551571	0.0795601774551571	1.20639226713565	unchanged
Meta_503	9,10-Dihydroxystearic acid	Lipids and lipid-like molecules	Fatty Acyls	Fatty acids and conjugates	HMDB0302281	C19622	-0.129666667	0.641147116719775	0.641147116719775	0.360591996180357	unchanged
Meta_504	Phosmet	Organoheterocyclic compounds	Isoindoles and derivatives	Isoindolines	HMDB0256463	C18756	0.115	0.691885277541004	0.691885277541004	0.323460667954096	unchanged
Meta_505	(2Z)-6-Hydroxynon-2-enoylcarnitine	Lipids and lipid-like molecules	Fatty Acyls	Fatty acid esters	HMDB0241750		0.449333333333334	0.462679582406418	0.462679582406418	0.579245752362471	unchanged
Meta_506	Val-Val-Val	Organic acids and derivatives	Carboxylic acids and derivatives	Amino acids, peptides, and analogues	HMDB0094676		-0.36	0.215885852380006	0.215885852380006	0.984547615826269	unchanged
Meta_507	Decanoylcarnitine	Lipids and lipid-like molecules	Fatty Acyls	Fatty acid esters	HMDB0000651		-1.814333333	0.0922519232043835	0.0922519232043835	1.14545317708074	unchanged
Meta_509	8-(2,6-Diethyl-4-methylphenyl)-1,2,4,5-tetrahydropyrazolo[1,2-d][1,4,5]oxadiazepine-7,9-dione	Unclassified	Unclassified	Unclassified			-0.437666667	0.253912728033628	0.253912728033628	0.821601238727797	unchanged
Meta_51	Maleic acid	Organic acids and derivatives	Carboxylic acids and derivatives	Dicarboxylic acids and derivatives	HMDB0000176	C01384	-0.176333333	0.702313944712264	0.702313944712264	0.284765384856069	unchanged
Meta_510	Cocaethylene	Benzenoids	Benzene and substituted derivatives	Benzoic acids and derivatives	HMDB0041860		-0.227666667	0.157622338371569	0.157622338371569	1.03241617961234	unchanged
Meta_511	Leucyl-Tryptophan	Organic acids and derivatives	Carboxylic acids and derivatives	Amino acids, peptides, and analogues	HMDB0028940		-1.031666667	0.473172253970132	0.473172253970132	0.53071584893871	unchanged
Meta_512	(3Z,6Z,9Z,12Z,15Z)-N-(2-hydroxyethyl)octadeca-3,6,9,12,15-pentaenamide	Unclassified	Unclassified	Unclassified			-0.226	0.481987542834592	0.481987542834592	0.544073519755413	unchanged
Meta_514	2,3-Dihydro-2,3-dihydroxy-4-(4-methoxyphenyl)-1H-phenalen-1-one	Benzenoids	Naphthalenes	Phenylnaphthalenes	HMDB0041463		0.0439999999999987	0.811658082149334	0.811658082149334	0.203108118793506	unchanged
Meta_515	Cryptotanshinone	Lipids and lipid-like molecules	Prenol lipids	Diterpenoids	HMDB0035220		-0.095	0.577271178523789	0.577271178523789	0.455832860689641	unchanged
Meta_518	Chloroquine	Organoheterocyclic compounds	Quinolines and derivatives	Aminoquinolines and derivatives	HMDB0014746	C07625	-0.813333333	0.521555793395848	0.521555793395848	0.513424149637311	unchanged
Meta_519	5-Thymidylic acid	Nucleosides, nucleotides, and	Pyrimidine nucleotides	Pyrimidine deoxyribonucleotides	HMDB0001227	C00364	0.313333333333334	0.225809892464419	0.225809892464419	0.853684628163967	unchanged
Meta_520	5alpha-Pregnane	Lipids and lipid-like molecules	Steroids and steroid derivatives	Pregnane steroids	HMDB0247017		-1.114	0.099838524986949	0.099838524986949	1.22250590847636	unchanged
Meta_521	Uridine 5'-monophosphate	Nucleosides, nucleotides, and	Pyrimidine nucleotides	Pyrimidine ribonucleotides	HMDB0000288	C00105	0.0626666666666651	0.851291956036622	0.851291956036622	0.160850997951273	unchanged
Meta_522	Trihexyphenidyl	Organic nitrogen compounds	Organonitrogen compounds	Amines	HMDB0014520	C07171	-0.209	0.287879235833054	0.287879235833054	0.785966899184503	unchanged

Meta_524	TRIPHENYL PHOSPHATE	Organic acids and derivatives	Organic phosphoric acids and	Phosphate esters	HMDB0259262	C14235	0.0416666666666679	0.811039880191164	0.811039880191164	0.196107205042382	unchanged
Meta_525	Denatonium	Unclassified	Unclassified	Unclassified			-0.234	0.245176782504986	0.245176782504986	0.873096169466789	unchanged
Meta_526	Octadecanamide	Organic acids and derivatives	Carboximidic acids and derivatives	Carboximidic acids	HMDB0034146	C13846	-1.999	0.0864358651053489	0.0864358651053489	1.22041464772061	unchanged
Meta_527	Oleoylethanolamide	Organic nitrogen compounds	Organonitrogen compounds	Amines	HMDB0002088		0.233333333333334	0.575801051835161	0.575801051835161	0.418036590783079	unchanged
Meta_528	(R)-Heraclenol	Phenylpropanoids and polyketides	Coumarins and derivatives	Furanocoumarins	HMDB0030745		0.121000000000002	0.631654929989438	0.631654929989438	0.384751248751789	unchanged
Meta_529	10-(4-Sulfophenyl)decanoic acid	Unclassified	Unclassified	Unclassified			0.439999999999998	0.309433172613049	0.309433172613049	0.732289759218286	unchanged
Meta_531	Avocadyne 4-acetate	Lipids and lipid-like molecules	Fatty Acyls	Fatty alcohols	HMDB0031049		0.306000000000001	0.569390044930881	0.569390044930881	0.427278356331153	unchanged
Meta_532	N-(1-Deoxy-1-fructosyl)phenylalanine	Organic acids and derivatives	Carboxylic acids and derivatives	Amino acids, peptides, and analogues	HMDB0037846		0.494666666666667	0.265182415301261	0.265182415301261	0.798256725170047	unchanged
Meta_533	Stearoylethanolamide	Organic nitrogen compounds	Organonitrogen compounds	Amines	HMDB0013078		0.0409999999999986	0.909848982456537	0.909848982456537	0.0984294364348067	unchanged
Meta_534	Phenylalanyltyrosine	Organic acids and derivatives	Carboxylic acids and derivatives	Amino acids, peptides, and analogues	HMDB0029007		-1.560666667	0.0760057324606818	0.0760057324606818	1.1915275774407	unchanged
Meta_535	(15Z)-9,12,13-Trihydroxy-15-octadecenoic acid	Unclassified	Unclassified	Unclassified			-0.496333333	0.419436098564007	0.419436098564007	0.624457939353555	unchanged
Meta_536	Rhodamine 110	Organoheterocyclic compounds	Benzopyrans	1-benzopyrans	HMDB0257198		0.0886666666666667	0.749987394229789	0.749987394229789	0.245397763036918	unchanged
Meta_537	Bexarotene	Lipids and lipid-like molecules	Prenol lipids	Retinoids	HMDB0014452		-0.230666667	0.526961553331187	0.526961553331187	0.498107489726717	unchanged
Meta_539	2,4,12-Octadecatrienoic acid isobutylamide	Lipids and lipid-like molecules	Fatty Acyls	Fatty amides	HMDB0032033		-2.066	0.0570287008276993	0.0570287008276993	1.199758426	unchanged
Meta_54	Methylmalonic acid	Organic acids and derivatives	Carboxylic acids and derivatives	Dicarboxylic acids and derivatives	HMDB0000202	C02170	0.0706666666666678	0.380775252686907	0.380775252686907	0.646127648920161	unchanged
Meta_540	Nivalenol	Lipids and lipid-like molecules	Prenol lipids	Sesquiterpenoids	HMDB0004304		0.203333333333333	0.470414790394598	0.470414790394598	0.538078509801829	unchanged
Meta_541	Pentids	Organic acids and derivatives	Carboxylic acids and derivatives	Amino acids, peptides, and analogues	HMDB0256229		-0.101666667	0.831302338636133	0.831302338636133	0.18469750917878	unchanged
Meta_542	Pipericine	Lipids and lipid-like molecules	Fatty Acyls	Fatty amides	HMDB0031678		-1.510333333	0.116787682478066	0.116787682478066	1.17909932549139	unchanged
Meta_543	8-Hydroxy-9,10-epoxystearic acid	Unclassified	Unclassified	Unclassified			-0.731333333	0.183339474040895	0.183339474040895	0.937992220777948	unchanged
Meta_544	20-Hydroxyeicosatetraenoic acid	Lipids and lipid-like molecules	Fatty Acyls	Fatty acids and conjugates	HMDB0005998	C14748	-0.183	0.227056128330208	0.227056128330208	0.89941036132276	unchanged
Meta_545	13-Docosenamide	Lipids and lipid-like molecules	Fatty Acyls	Fatty amides	HMDB0244507		-1.067333333	0.125086024626134	0.125086024626134	1.16133658848413	unchanged
Meta_546	Fructose 1,6-bisphosphate	Organic oxygen compounds	Organooxygen compounds	Carbohydrates and carbohydrate conjugates	HMDB0001058	C05378	0.0493333333333332	0.821182543498319	0.821182543498319	0.17222415646693	unchanged
Meta_547	Canrenone	Lipids and lipid-like molecules	Steroids and steroid derivatives	Steroid lactones	HMDB0003033		0.990666666666666	0.171064796222737	0.171064796222737	1.00638902783516	unchanged
Meta_548	12-KETE	Lipids and lipid-like molecules	Fatty Acyls	Fatty acids and conjugates	HMDB0013633	C14807	-0.918333333	0.369364216370526	0.369364216370526	0.670071448771051	unchanged
Meta_549	Monoelaidin	Lipids and lipid-like molecules	Glycerolipids	Monoradylglycerols	HMDB0254854		-0.182666667	0.228829508186913	0.228829508186913	0.87576866403197	unchanged
Meta_551	Docosanamide	Lipids and lipid-like molecules	Fatty Acyls	Fatty amides	HMDB0000583		-1.480666667	0.207843424080617	0.207843424080617	0.948472694619735	unchanged
Meta_552	12-[(Cyclohexylcarbamoyl)amino]dodecanoic acid	Unclassified	Unclassified	Unclassified			0.0709999999999997	0.552058157093104	0.552058157093104	0.467458730964897	unchanged
Meta_553	Diprotin A	Organoheterocyclic compounds	Pyrroles	Substituted pyrroles	HMDB0251473		-0.543333333	0.618124983880475	0.618124983880475	0.401712911458338	unchanged
Meta_555	[2,2'-Biquinoline]-4,4'-dicarboxylic acid	Organoheterocyclic compounds	Quinolines and derivatives	Quinoline carboxylic acids	HMDB0244480		0.0226666666666659	0.937597308749777	0.937597308749777	0.0661549445823616	unchanged
Meta_556	Aceprometazine	Organoheterocyclic compounds	Benzothiazines	Phenothiazines	HMDB0015553		-0.303	0.344188519859868	0.344188519859868	0.748229795671783	unchanged
Meta_557	Aritmina	Alkaloids and derivatives	Ajmaline-sarpagine alkaloids	Unclassified	HMDB0248601		0.104666666666667	0.555171608644375	0.555171608644375	0.452517814208537	unchanged
Meta_559	3-[2-(2,3-dihydro-1,4-benzodioxin-6-yl)-2-oxoethyl]quinazolin-4(3H)-one	Unclassified	Unclassified	Unclassified			-0.073	0.589460702064039	0.589460702064039	0.435275277217584	unchanged
Meta_56	2-Hydroxy-2-methylbutyric acid	Lipids and lipid-like molecules	Fatty Acyls	Fatty acids and conjugates	HMDB0001987		0.386333333333333	0.184727795402821	0.184727795402821	0.926736478563319	unchanged
Meta_560	2-Methoxy-4-vinylphenol	Benzenoids	Phenols	Methoxyphenols	HMDB0013744	C17883	0.795	0.0524434862636163	0.0524434862636163	1.25254182568695	unchanged
Meta_561	Tyrosyl-Tyrosine	Organic acids and derivatives	Carboxylic acids and derivatives	Amino acids, peptides, and analogues	HMDB0029117		-1.782333333	0.0868984281366923	0.0868984281366923	1.13824946483782	unchanged
Meta_562	Cytidine monophosphate	Nucleosides, nucleotides, and	Pyrimidine nucleotides	Pyrimidine ribonucleotides	HMDB0000095	C00055	0.156333333333333	0.70703963560398	0.70703963560398	0.311424944565354	unchanged
Meta_563	Adenosine monophosphate	Nucleosides, nucleotides, and	Purine nucleotides	Purine ribonucleotides	HMDB0000045	C00020	0.0420000000000016	0.864132868027923	0.864132868027923	0.146267895365797	unchanged
Meta_565	3(2'-Chlorophenyl)-7-hydroxy-4-phenylcoumarin	Unclassified	Unclassified	Unclassified			0.0946666666666669	0.534789169979057	0.534789169979057	0.507136622931321	unchanged
Meta_566	hydroquinidine	Alkaloids and derivatives	Cinchona alkaloids	Unclassified	HMDB0242615		-0.217666667	0.147617854645657	0.147617854645657	1.07608804710116	unchanged
Meta_567	NA-Dopamine 13:0	Unclassified	Unclassified	Unclassified			-0.243	0.470662330797755	0.470662330797755	0.570721857733756	unchanged
Meta_569	N-(4-fluorophenyl)-2-(4-hydroxyquinazolin-2-ylthio)acetamide CollisionEnergy:102040	Unclassified	Unclassified	Unclassified			-0.657	0.0985234470834741	0.0985234470834741	1.16210840165492	unchanged
Meta_57	2,5-Dimethyltetrahydrothiophene	Organoheterocyclic compounds	Thiolanes	Unclassified	HMDB0302173		0.281333333333336	0.152605349250682	0.152605349250682	1.04728305259907	unchanged
Meta_570	Prostaglandin B2	Lipids and lipid-like molecules	Fatty Acyls	Eicosanoids	HMDB0004236	C05954	-0.332333333	0.252529359200586	0.252529359200586	0.920360098680955	unchanged
Meta_571	Dutasteride	Lipids and lipid-like molecules	Steroids and steroid derivatives	Androstane steroids	HMDB0015258		-0.412	0.395020982617975	0.395020982617975	0.667687778215636	unchanged
Meta_572	THIODICARB	Organic acids and derivatives	Organic carbonic acids and derivatives	Unclassified	HMDB0259010		-0.181333333	0.519029564155431	0.519029564155431	0.503272537648352	unchanged
Meta_573	Penicilloic acid	Organic acids and derivatives	Carboxylic acids and derivatives	Amino acids, peptides, and analogues	HMDB0060617		0.225666666666669	0.475574440294574	0.475574440294574	0.545111155326666	unchanged
Meta_574	5,7-Dihydroxy-2-(4-methoxyphenyl)-6-(3-methylbut-2-enyl)-2,3-dihydrochromen-4-one	Unclassified	Unclassified	Unclassified			0.640333333333334	0.229858077896046	0.229858077896046	0.901735446676516	unchanged
Meta_575	Phenolsulfonphthalein	Unclassified	Unclassified	Unclassified		C12600	-0.049666667	0.859755042612584	0.859755042612584	0.154962761794886	unchanged
Meta_576	Gingerenone A	Phenylpropanoids and polyketides	Diarylheptanoids	Linear diarylheptanoids	HMDB0035403	C10460	0.208333333333332	0.656269842565884	0.656269842565884	0.356554815459147	unchanged
Meta_577	MG(0:0/18:2(9Z,12Z)/0:0)	Lipids and lipid-like molecules	Fatty Acyls	Lineolic acids and derivatives	HMDB0011538		-0.118333333	0.455782747397663	0.455782747397663	0.575992394797734	unchanged
Meta_578	Cinacalcet	Benzenoids	Naphthalenes	Unclassified	HMDB0015147		0.258333333333333	0.231474260639584	0.231474260639584	0.86896821623293	unchanged
Meta_579	5,4'-Dihydroxy-3,3'-dimethoxy-6:7-methylenedioxyflavone	Phenylpropanoids and polyketides	Flavonoids	O-methylated flavonoids	HMDB0029272		-0.202	0.368160578283564	0.368160578283564	0.674662609066305	unchanged
Meta_580	2-[(3,5-dioxo-2H-1,2,4-triazin-6-yl)amino]-N-[(4-hydroxy-3-methoxyphenyl)methylideneamino]acetamide	Unclassified	Unclassified	Unclassified			-0.105	0.701160301563518	0.701160301563518	0.325277376177271	unchanged
Meta_581	Pantetheine 4'-phosphate	Organic acids and derivatives	Carboxylic acids and derivatives	Amino acids, peptides, and analogues	HMDB0001416	C01134	0.176	0.803761320771046	0.803761320771046	0.184413655938088	unchanged
Meta_582	Dopexamine	Benzenoids	Phenols	Benzenediols	HMDB0041882		-0.197333333	0.729377410634239	0.729377410634239	0.283260479540511	unchanged
Meta_583	Arachidic acid	Lipids and lipid-like molecules	Fatty Acyls	Fatty acids and conjugates	HMDB0002212	C06425	0.852333333333332	0.611515382944055	0.611515382944055	0.410463644130971	unchanged
Meta_584	Tetracosapentaenoic acid (24:5n-6)	Lipids and lipid-like molecules	Fatty Acyls	Fatty acids and conjugates	HMDB0006322		0.0499999999999972	0.714488446645797	0.714488446645797	0.30168203013871	unchanged

Meta_585	Meta-Tyrosine	Organic acids and derivatives	Carboxylic acids and derivatives	Amino acids, peptides, and analogues	HMDB0059720		0.00833333333333286	0.933660349048419	0.933660349048419	0.0667381688199897	unchanged
Meta_586	O-Arachidonoyl Glycidol	Unclassified	Unclassified	Unclassified			-0.585333333	0.276948509944494	0.276948509944494	0.845540815937581	unchanged
Meta_587	Guanosine monophosphate	Nucleosides, nucleotides, and	Purine nucleotides	Purine ribonucleotides	HMDB0001397	C00144	-0.005333333	0.991986028880417	0.991986028880417	0.0022087808505457	unchanged
Meta_588	Carbocromen	Phenylpropanoids and polyketides	Coumarins and derivatives	Unclassified	HMDB0249632		-0.830666667	0.285991977020767	0.285991977020767	0.81077082257551	unchanged
Meta_589	(+)-Butaclamol hydrochloride	Unclassified	Unclassified	Unclassified			-0.088	0.670135968209698	0.670135968209698	0.327625340779193	unchanged
Meta_59	Betaine	Organic acids and derivatives	Carboxylic acids and derivatives	Amino acids, peptides, and analogues	HMDB0000043	C00719	0.537333333333336	0.13327649228236	0.13327649228236	1.12998811485568	unchanged
Meta_590	9,10-Epoxystearic acid	Lipids and lipid-like molecules	Fatty Acyls	Lineolic acids and derivatives	HMDB0247617	C19418	0.414	0.184807308481325	0.184807308481325	0.956766198798446	unchanged
Meta_592	Perfluoroheptanoic acid	Organohalogen compounds	Alkyl halides	Alkyl fluorides	HMDB0256322		-0.790333333	0.351991825597536	0.351991825597536	0.768639205903323	unchanged
Meta_593	(4-Benzylpiperazin-1-yl)(3,5-dimethoxyphenyl)methanone	Unclassified	Unclassified	Unclassified			-0.288666667	0.302490191079764	0.302490191079764	0.770889845785501	unchanged
Meta_594	Docosanal	Lipids and lipid-like molecules	Fatty Acyls	Fatty aldehydes	HMDB0251560		-1.067333333	0.060530315310139	0.060530315310139	1.30537543615034	unchanged
Meta_596	N-(3-chlorophenyl)-2-(4-hydroxyquinazolin-2-ylthio)acetamide CollisionEnergy:102040	Unclassified	Unclassified	Unclassified			0.350000000000001	0.507087029632291	0.507087029632291	0.556300398445948	unchanged
Meta_597	3-(3-Methyl-5-pentylfuran-2-yl)propanoylcarnitine	Lipids and lipid-like molecules	Fatty Acyls	Fatty acid esters	HMDB0241844		-0.087666667	0.583884124804511	0.583884124804511	0.452932171440942	unchanged
Meta_598	Methyltricaprylylammonium	Unclassified	Unclassified	Unclassified			-0.234	0.655644781019481	0.655644781019481	0.372631254821082	unchanged
Meta_599	Octadecyl fumarate	Lipids and lipid-like molecules	Fatty Acyls	Fatty acid esters	HMDB0038073		0.0563333333333347	0.911488899858381	0.911488899858381	0.0896439020065381	unchanged
Meta_6	Pyrroline	Organoheterocyclic compounds	Pyrrolines	Unclassified	HMDB0245392		0.280666666666665	0.125676147826511	0.125676147826511	1.03948212064945	unchanged
Meta_60	L-Valine	Organic acids and derivatives	Carboxylic acids and derivatives	Amino acids, peptides, and analogues	HMDB0000883	C00183	0.926666666666669	0.259752107243938	0.259752107243938	0.796313702811332	unchanged
Meta_600	Cholesterol	Lipids and lipid-like molecules	Steroids and steroid derivatives	Cholestane steroids	HMDB0000067	C00187	-0.153666667	0.283415714117465	0.283415714117465	0.774463658283626	unchanged
Meta_601	Adenosine 2'-phosphate	Organic oxygen compounds	Organooxygen compounds	Carbohydrates and carbohydrate conjugates	HMDB0011617	C00946	0.164000000000001	0.476678611952802	0.476678611952802	0.558780570501132	unchanged
Meta_603	Angelicin	Phenylpropanoids and polyketides	Coumarins and derivatives	Furanocoumarins	HMDB0033930	C09060	-0.458666667	0.450551547986309	0.450551547986309	0.614851763152946	unchanged
Meta_604	(-)-Arctigenin	Lignans, neolignans and related compounds	Furanoid lignans	Tetrahydrofuran lignans	HMDB0030087		-0.166333333	0.595592177082455	0.595592177082455	0.429283868056963	unchanged
Meta_605	Docosahexaenoyl Ethanolamide	Organic nitrogen compounds	Organonitrogen compounds	Amines	HMDB0013658		0.632666666666667	0.14492655065677	0.14492655065677	1.11818122449163	unchanged
Meta_607	(7Z,10Z,13Z,16Z,19Z)-N-(2-Hydroxyethyl)docosa-7,10,13,16,19-pentaenamide	Unclassified	Unclassified	Unclassified			-0.550333333	0.171826649259208	0.171826649259208	1.06261351854421	unchanged
Meta_608	(10Z,13Z,16Z,19Z)-N-(2-hydroxyethyl)docosa-10,13,16,19-tetraenamide	Unclassified	Unclassified	Unclassified			-0.895666667	0.0575139105278886	0.0575139105278886	1.17943717961523	unchanged
Meta_609	Riboflavin	Organoheterocyclic compounds	Pteridines and derivatives	Alloxazines and isoalloxazines	HMDB0000244	C00255	0.378333333333332	0.472842260369841	0.472842260369841	0.578063212205767	unchanged
Meta_610	7a-Hydroxy-5b-cholanic acid	Lipids and lipid-like molecules	Steroids and steroid derivatives	Bile acids, alcohols and derivatives	HMDB0002492		0.0536666666666683	0.711548487620501	0.711548487620501	0.302477276102203	unchanged
Meta_611	S-Lactoylglutathione	Organic acids and derivatives	Carboxylic acids and derivatives	Amino acids, peptides, and analogues	HMDB0001066	C03451	-0.774	0.092144743972934	0.092144743972934	1.10940263524961	unchanged
Meta_614	N-[2-(1H-indol-3-yl)ethyl]-2,2-diphenylcyclopropane-1-carboxamide	Unclassified	Unclassified	Unclassified			1.44766666666667	0.193153706663989	0.193153706663989	0.971907597111621	unchanged
Meta_615	Tetraethylene glycol monododecyl ether	Organic oxygen compounds	Organooxygen compounds	Ethers	HMDB0246913		0.194666666666667	0.297136331983339	0.297136331983339	0.753938428059382	unchanged
Meta_616	Ochrephilone	Unclassified	Unclassified	Unclassified			0.898666666666667	0.149418485112241	0.149418485112241	1.02763163852256	unchanged
Meta_619	(6beta,7alpha,12beta,13beta)-7-Hydroxy-11,16-dioxo-8,14-apianadien-22,6-olide	Lipids and lipid-like molecules	Prenol lipids	Terpene lactones	HMDB0031380		0.222000000000001	0.4930253169069	0.4930253169069	0.516816252209124	unchanged
Meta_62	Phenylacetaldehyde	Benzenoids	Benzene and substituted derivatives	Phenylacetaldehydes	HMDB0006236	C00601	0.784000000000001	0.351272911615407	0.351272911615407	0.740741295884853	unchanged
Meta_620	Corynoxeine	Organoheterocyclic compounds	Indolizidines	Unclassified	HMDB0250480		-0.341333333	0.137053383237755	0.137053383237755	1.06786368024402	unchanged
Meta_621	Cholesta-4,6-dien-3-one	Lipids and lipid-like molecules	Steroids and steroid derivatives	Cholestane steroids	HMDB0002394		0.474	0.489451226984875	0.489451226984875	0.520508170756213	unchanged
Meta_623	(10Z)-Pentadec-10-enoylcarnitine	Lipids and lipid-like molecules	Fatty Acyls	Fatty acid esters	HMDB0241436		1.48933333333333	0.125434765758565	0.125434765758565	1.13568716820537	unchanged
Meta_625	Fingolimod phosphate ester, S-	Organic acids and derivatives	Organic phosphoric acids and	Phosphate esters	HMDB0243705		-0.800666667	0.109855253282815	0.109855253282815	1.05190304699344	unchanged
Meta_626	Pentadecanoylcarnitine	Lipids and lipid-like molecules	Fatty Acyls	Fatty acid esters	HMDB0062517		-2.412333333	0.0627566643240859	0.0627566643240859	1.29372351878042	unchanged
Meta_627	ethyl 2-amino-5-oxo-4-(pyridin-3-yl)-4H,5H-pyrano[3,2-c]chromene-3-carboxylate	Unclassified	Unclassified	Unclassified			0.0896666666666661	0.808125289051743	0.808125289051743	0.199367622665951	unchanged
Meta_628	Tcmdc-142530	Unclassified	Unclassified	Unclassified			0.218666666666667	0.498210740038612	0.498210740038612	0.539628410000371	unchanged
Meta_63	2-Methyl-1,4-benzoquinone	Unclassified	Unclassified	Unclassified			-0.307	0.200413555617281	0.200413555617281	0.942660855278555	unchanged
Meta_630	3-Hydroxydodec-6-enedioylcarnitine	Lipids and lipid-like molecules	Fatty Acyls	Fatty acid esters	HMDB0241231		-0.904333333	0.0983827383279373	0.0983827383279373	1.22456498631834	unchanged
Meta_632	1-(3,4-dihydroisoquinolin-2(1H)-yl)-2-(4-iodo-1H-pyrazol-1-yl)ethanone	Unclassified	Unclassified	Unclassified			0.108999999999998	0.622428632742762	0.622428632742762	0.400213214457489	unchanged
Meta_633	Bis(2-ethylhexyl) phthalate	Benzenoids	Benzene and substituted derivatives	Benzoic acids and derivatives	HMDB0249243	C03690	0.952333333333332	0.145090882153159	0.145090882153159	1.00220801086236	unchanged
Meta_634	Deoxycholic acid	Lipids and lipid-like molecules	Steroids and steroid derivatives	Bile acids, alcohols and derivatives	HMDB0000626	C04483	0.33	0.807931002799599	0.807931002799599	0.210462272314757	unchanged
Meta_635	MG(0:0/16:0/0:0)	Lipids and lipid-like molecules	Glycerolipids	Monoradylglycerols	HMDB0011533		-0.331666667	0.271164504617708	0.271164504617708	0.816230541930283	unchanged
Meta_636	Octaethylene glycol	Organic oxygen compounds	Organooxygen compounds	Ethers	HMDB0094680		-0.195333333	0.0819396246118162	0.0819396246118162	1.14110698125626	unchanged
Meta_637	N-Linoleoyl Isoleucine	Unclassified	Unclassified	Unclassified	HMDB0241978		0.0180000000000007	0.952176926774463	0.952176926774463	0.0323038593942057	unchanged
Meta_638	1-hexadecyl-glycero-3-phosphate	Lipids and lipid-like molecules	Glycerophospholipids	Glycerophosphates	HMDB0062325		0.935	0.0787491916300832	0.0787491916300832	1.12022637603363	unchanged
Meta_639	Petromyzonol	Unclassified	Unclassified	Unclassified		C16258	0.0546666666666678	0.539410142583321	0.539410142583321	0.507710036870483	unchanged
Meta_64	Niacinamide	Organoheterocyclic compounds	Pyridines and derivatives	Pyridinecarboxylic acids and derivatives	HMDB0001406	C00153	-0.138666667	0.785269643897531	0.785269643897531	0.222477517627806	unchanged
Meta_640	Kanzonol M	Phenylpropanoids and polyketides	Isoflavonoids	O-methylated isoflavonoids	HMDB0041101		0.870666666666667	0.202446650341461	0.202446650341461	0.935064034266984	unchanged
Meta_641	Kyotorphin	Organic acids and derivatives	Carboxylic acids and derivatives	Amino acids, peptides, and analogues	HMDB0005768	C02993	-0.451333333	0.346298644433084	0.346298644433084	0.745379306198873	unchanged
Meta_642	N-Docosahexaenoyl Alanine	Organic acids and derivatives	Carboxylic acids and derivatives	Amino acids, peptides, and analogues	HMDB0242007		-0.843666667	0.255181773300401	0.255181773300401	0.935384308315406	unchanged
Meta_644	S-Adenosylmethionine	Nucleosides, nucleotides, and	5'-deoxyribonucleosides	5'-deoxy-5'-thionucleosides	HMDB0001185		0.856333333333332	0.0696845571891549	0.0696845571891549	1.171345088	unchanged
Meta_646	L-Gulonolactone	Organoheterocyclic compounds	Lactones	Gamma butyrolactones	HMDB0003466	C01040	-0.663	0.360402867825627	0.360402867825627	0.716554676785499	unchanged
Meta_647	7a-Hydroxy-cholestene-3-one	Lipids and lipid-like molecules	Steroids and steroid derivatives	Cholestane steroids	HMDB0001993	C05455	-0.106666667	0.643261940056608	0.643261940056608	0.372637694213053	unchanged
Meta_648	7-Ketocholesterol	Lipids and lipid-like molecules	Steroids and steroid derivatives	Cholestane steroids	HMDB0000501		-0.314666667	0.0991848478812283	0.0991848478812283	1.17164450849337	unchanged

Meta_649	Uridine 5'-diphosphate	Nucleosides, nucleotides, and	Pyrimidine nucleotides	Pyrimidine ribonucleotides	HMDB0000295	C00015	-0.087666667	0.631959302794135	0.631959302794135	0.367660353398573	unchanged
Meta_65	Taurine	Organic acids and derivatives	Organic sulfonic acids and derivatives	Organosulfonic acids and derivatives	HMDB0000251	C00245	0.372	0.363768860868342	0.363768860868342	0.680796472292456	unchanged
Meta_650	Osajin	Unclassified	Unclassified	Unclassified			1.38666666666667	0.0522024460705606	0.0522024460705606	1.28167519550904	unchanged
Meta_651	N-[2-(3,4-dihydroxyphenyl)ethyl]heptadecanamide	Unclassified	Unclassified	Unclassified			-0.100333333	0.681490652908009	0.681490652908009	0.343353167817002	unchanged
Meta_652	Isorhynchophylline	Organoheterocyclic compounds	Indolizidines	Unclassified	HMDB0253688		-0.438666667	0.259590193555474	0.259590193555474	0.825895323630568	unchanged
Meta_653	Heliocide H3	Benzenoids	Anthracenes	Anthraquinones	HMDB0030741		0.565999999999999	0.183436824654265	0.183436824654265	0.935121099532451	unchanged
Meta_654	N-Oleoyl phenylalanine	Organic acids and derivatives	Carboxylic acids and derivatives	Amino acids, peptides, and analogues	HMDB0062336		-0.032	0.83031617316662	0.83031617316662	0.189927566615726	unchanged
Meta_655	6-Amino-2-(octadec-9-enoylamino)hexanoic acid	Unclassified	Unclassified	Unclassified			-0.937666667	0.101612813126784	0.101612813126784	1.0693380545646	unchanged
Meta_657	Benzethonium	Unclassified	Unclassified	Unclassified			-0.154666667	0.699560270956126	0.699560270956126	0.319178363004585	unchanged
Meta_658	Echimidine N-oxide	Unclassified	Unclassified	Unclassified			-0.396666667	0.430043102164236	0.430043102164236	0.664267839872373	unchanged
Meta_66	Nicotinic acid	Organoheterocyclic compounds	Pyridines and derivatives	Pyridinecarboxylic acids and derivatives	HMDB0001488	C00253	-0.238666667	0.438953307958983	0.438953307958983	0.597812899417621	unchanged
Meta_660	Gelomulide N	Unclassified	Unclassified	Unclassified			3.87966666666667	0.0759909916253488	0.0759909916253488	1.16429876242905	unchanged
Meta_661	Tomatidine	Lipids and lipid-like molecules	Steroids and steroid derivatives	Steroidal alkaloids	HMDB0034731	C10826	0.289999999999999	0.2858653695665	0.2858653695665	0.827308067701205	unchanged
Meta_663	Syringic acid glucuronide	Phenylpropanoids and polyketides	Coumarins and derivatives	Unclassified	HMDB0240562		0.0750000000000011	0.80177184064384	0.80177184064384	0.190532461275742	unchanged
Meta_664	1,1,2-Trimethyl-1H-benzo[E]indole	Benzenoids	Naphthalenes	Unclassified	HMDB0246655		-0.287	0.362556944044396	0.362556944044396	0.731018973434788	unchanged
Meta_665	LPE O-15:1	Unclassified	Unclassified	Unclassified			1.083	0.235010232996197	0.235010232996197	0.9404213997492	unchanged
Meta_667	O-Linoleoylcarnitine	Lipids and lipid-like molecules	Fatty Acyls	Fatty acid esters	HMDB0240780		-1.585	0.0514221830994122	0.0514221830994122	1.21902330126868	unchanged
Meta_668	Adenosine 3',5'-diphosphate	Nucleosides, nucleotides, and	Purine nucleotides	Purine ribonucleotides	HMDB0000061	C00054	-0.050666667	0.848108475731274	0.848108475731274	0.161977204916509	unchanged
Meta_669	LPC O-12:0	Unclassified	Unclassified	Unclassified			0.218999999999999	0.390857898195415	0.390857898195415	0.639002221909636	unchanged
Meta_671	14-Hydroxy-6,10,23-trimethyl-4-azahexacyclo [12.11.0.02,11.04,9.015,24.018,23]pentacosane-17,20-dione	Unclassified	Unclassified	Unclassified			-0.929	0.428264771053603	0.428264771053603	0.630456755329675	unchanged
Meta_673	Derhamnosylmaysin	Phenylpropanoids and polyketides	Flavonoids	Flavonoid glycosides	HMDB0301877		-0.231333333	0.26230359853799	0.26230359853799	0.808782542266566	unchanged
Meta_674	Ingenol-3,4:5,20-diacetonide	Unclassified	Unclassified	Unclassified			1.07333333333333	0.0987637152647549	0.0987637152647549	1.09134780236267	unchanged
Meta_675	Desmosterol	Lipids and lipid-like molecules	Steroids and steroid derivatives	Cholestane steroids	HMDB0002719	C01802	-0.503333333	0.0507681190303721	0.0507681190303721	1.20222146210231	unchanged
Meta_676	7-Ketologanin	Unclassified	Unclassified	Unclassified			0.528666666666667	0.150522446950618	0.150522446950618	0.996942988325606	unchanged
Meta_677	LPE O-16:2	Unclassified	Unclassified	Unclassified			1.023	0.101536829349673	0.101536829349673	1.10274062513705	unchanged
Meta_678	DG 21:1	Unclassified	Unclassified	Unclassified			-0.101666667	0.55973044177352	0.55973044177352	0.459423905341165	unchanged
Meta_68	Pyroglutamic acid	Organic acids and derivatives	Carboxylic acids and derivatives	Amino acids, peptides, and analogues	HMDB0000267	C01879	0.231999999999999	0.174467847794242	0.174467847794242	0.952371272409914	unchanged
Meta_680	Dinonyl phthalate	Benzenoids	Benzene and substituted derivatives	Benzoic acids and derivatives	HMDB0251424		0.907666666666668	0.123232552649172	0.123232552649172	1.04231505426555	unchanged
Meta_681	gamma-Glutamylalanine	Organic acids and derivatives	Carboxylic acids and derivatives	Amino acids, peptides, and analogues	HMDB0006248	C03740	3.06266666666667	0.125049026703713	0.125049026703713	1.15337332730108	unchanged
Meta_682	Nonaethylene glycol	Unclassified	Unclassified	Unclassified			-0.286333333	0.228211994409506	0.228211994409506	0.873659193570379	unchanged
Meta_683	Myosmine	Organoheterocyclic compounds	Pyridines and derivatives	Unclassified	HMDB0030386	C10160	-0.229333333	0.747265155140584	0.747265155140584	0.289524647254759	unchanged
Meta_684	27-Nor-5b-cholestane-3a,7a,12a,24,25-pentol	Lipids and lipid-like molecules	Steroids and steroid derivatives	Bile acids, alcohols and derivatives	HMDB0002126		-0.116	0.809675099173597	0.809675099173597	0.207566014679283	unchanged
Meta_685	Folic acid	Benzenoids	Phenols	Methoxyphenols	HMDB0000121	C00504	0.257	0.496196591018177	0.496196591018177	0.514282490219531	unchanged
Meta_686	Cycloart-23-ene-3beta,25-diol	Unclassified	Unclassified	Unclassified			-0.066333333	0.480092575283395	0.480092575283395	0.527516649665522	unchanged
Meta_687	Glycoursodeoxycholic acid	Lipids and lipid-like molecules	Steroids and steroid derivatives	Bile acids, alcohols and derivatives	HMDB0000708		-0.217333333	0.44560211575576	0.44560211575576	0.61738749896581	unchanged
Meta_689	LysoPE(16:1(9Z)/0:0)	Lipids and lipid-like molecules	Glycerophospholipids	Glycerophosphoethanolamines	HMDB0011504		0.907333333333334	0.0663480066464939	0.0663480066464939	1.15212140890774	unchanged
Meta_69	S-2-Propenyl propanethioate	Organic acids and derivatives	Thiocarboxylic acids and derivatives	Thioesters	HMDB0037493		0.253666666666664	0.309993190656078	0.309993190656078	0.741879642048292	unchanged
Meta_691	1-[1-(4-Methoxyphenyl)-1,3,4,9-tetrahydropyrido[3,4-b]indol-2-yl]-3-phenylprop-2-en-1-one	Unclassified	Unclassified	Unclassified			0.163	0.250865052763463	0.250865052763463	0.837943741263016	unchanged
Meta_692	Dioctyl phthalate	Benzenoids	Benzene and substituted derivatives	Benzoic acids and derivatives	HMDB0251427	C14227	1.03	0.17058142755908	0.17058142755908	0.989148296534616	unchanged
Meta_695	Flavin mononucleotide	Nucleosides, nucleotides, and	Flavin nucleotides	Unclassified	HMDB0001520	C00061	0.734	0.105353977163184	0.105353977163184	1.20834802787915	unchanged
Meta_697	Citranaxanthin	Lipids and lipid-like molecules	Prenol lipids	Triterpenoids	HMDB0036883		-0.401333333	0.496511375949804	0.496511375949804	0.511091247473197	unchanged
Meta_7	2-Methyl-1-butene	Hydrocarbons	Unsaturated hydrocarbons	Branched unsaturated hydrocarbons	HMDB0245210		-0.163666667	0.140380122082763	0.140380122082763	1.09128172645752	unchanged
Meta_700	LPE O-18:2	Unclassified	Unclassified	Unclassified			0.657	0.0528196408414762	0.0528196408414762	1.21889564555753	unchanged
Meta_704	Acetildenafil	Organic oxygen compounds	Organooxygen compounds	Carbonyl compounds	HMDB0247908		-0.448333333	0.221763538950604	0.221763538950604	0.8783270499902	unchanged
Meta_705	LysoSM(d18:1)	Lipids and lipid-like molecules	Sphingolipids	Phosphosphingolipids	HMDB0006482		-0.878	0.0623236116716223	0.0623236116716223	1.20895466615285	unchanged
Meta_707	1-Heptadecanoylglycerophosphoethanolamine	Lipids and lipid-like molecules	Glycerophospholipids	Glycerophosphoethanolamines	HMDB0061691		0.546666666666667	0.0655899956383332	0.0655899956383332	1.16090690015163	unchanged
Meta_710	histamine-C23:1	Unclassified	Unclassified	Unclassified			-0.114666667	0.759721626353422	0.759721626353422	0.21853622531117	unchanged
Meta_711	Glycitin	Phenylpropanoids and polyketides	Isoflavonoids	Isoflavonoid O-glycosides	HMDB0002219		-0.384666667	0.200938367845313	0.200938367845313	0.949175988140965	unchanged
Meta_712	Withaferin A	Lipids and lipid-like molecules	Steroids and steroid derivatives	Steroid lactones	HMDB0259897	C08841	-0.140333333	0.281348540096569	0.281348540096569	0.796895770011268	unchanged
Meta_713	Raloxifene	Organic oxygen compounds	Organooxygen compounds	Carbonyl compounds	HMDB0014624	C07228	-0.198333333	0.794232297758151	0.794232297758151	0.227647773236304	unchanged
Meta_714	Nutriacholic acid	Lipids and lipid-like molecules	Steroids and steroid derivatives	Bile acids, alcohols and derivatives	HMDB0000467		-0.104	0.812187671610874	0.812187671610874	0.171216329878156	unchanged
Meta_715	Folinic acid	Organoheterocyclic compounds	Pteridines and derivatives	Pterins and derivatives	HMDB0001562	C03479	-0.160666667	0.421184153319441	0.421184153319441	0.644856979410747	unchanged
Meta_716	1-[1-[4-(Diethylamino)phenyl]-4a-hydroxy-1,3,4,5,6,7,8,8a-octahydroisoquinolin-2-yl]-3-(4-propan-2-ylphenyl)prop-2-en-1-one	Unclassified	Unclassified	Unclassified			-0.320666667	0.139497730096988	0.139497730096988	1.02784762990668	unchanged
Meta_717	N-(3-chloro-4-methoxyphenyl)-2-[4-(morpholin-4-ylcarbonyl)-2-oxoquinolin-1(2H)-yl]acetamide	Unclassified	Unclassified	Unclassified			0.719000000000001	0.402488955925938	0.402488955925938	0.695766706734329	unchanged
Meta_718	LysoPE(18:2(9Z,12Z)/0:0)	Lipids and lipid-like molecules	Glycerophospholipids	Glycerophosphoethanolamines	HMDB0011507		0.702999999999999	0.236867208074801	0.236867208074801	0.951340536502222	unchanged
Meta_72	QUINUCLIDINE	Organoheterocyclic compounds	Quinuclidines	Unclassified	HMDB0257057		-0.178666667	0.802919845292443	0.802919845292443	0.22239782450125	unchanged

Meta_723	(E)-6,10-Dimethyl-9-methylene-5-undecen-2-one	Organic oxygen compounds	Organooxygen compounds	Carbonyl compounds	HMDB0035862		-0.311333333	0.322708684116629	0.322708684116629	0.786365016119073	unchanged
Meta_725	17-(Furan-3-yl)-6-hydroxy-4,4,8-trimethyl-3,7-dioxo-14,15-epoxyandrosta-1,5-dien-11-yl acetate	Unclassified	Unclassified	Unclassified			0.514333333333333	0.278481279644058	0.278481279644058	0.816608736729659	unchanged
Meta_726	LPG 16:1	Unclassified	Unclassified	Unclassified			-0.144333333	0.759841830587924	0.759841830587924	0.237179712760081	unchanged
Meta_727	Decaethylene glycol	Unclassified	Unclassified	Unclassified			-0.069333333	0.724038286627642	0.724038286627642	0.294879010831799	unchanged
Meta_73	N-Acetyl-beta-alanine	Organic acids and derivatives	Carboxylic acids and derivatives	Carboxylic acids	HMDB0061880	C01073	-0.259333333	0.0555514853727206	0.0555514853727206	1.18720945526014	unchanged
Meta_730	22-Hydoxy-2-hopen-1-one	Unclassified	Unclassified	Unclassified			-0.182333333	0.247867703182015	0.247867703182015	0.849795633541243	unchanged
Meta_731	3b,18b-3-Methoxy-11-oxo-12-oleanen-30-oic acid	Lipids and lipid-like molecules	Prenol lipids	Triterpenoids	HMDB0034516		-0.461	0.107882926947105	0.107882926947105	1.10665788053354	unchanged
Meta_732	""""""\2-[9-(4-methylphenyl)-6-oxohydropurin-8-ylthio]-N-(2,3,5,6-tetrafluorophenyl)a cetamide CollisionEnergy:205060\""""""""""""""""""""""""	Unclassified	Unclassified	Unclassified			2.35033333333333	0.0824597303750416	0.0824597303750416	1.25798173878811	unchanged
Meta_733	Arg-C22:5	Unclassified	Unclassified	Unclassified			-0.339666667	0.223975880459907	0.223975880459907	0.919790520732302	unchanged
Meta_735	1-[1-[4-(Diethylamino)phenyl]-4a-hydroxy-1,3,4,5,6,7,8,8a-octahydroisoquinolin-2-yl]-3-(3,4-dimethoxyphenyl)prop-2-en-1-one	Unclassified	Unclassified	Unclassified			0.271666666666667	0.815041082125113	0.815041082125113	0.209260846171341	unchanged
Meta_736	LPS 16:1	Unclassified	Unclassified	Unclassified			0.427666666666667	0.138545514896064	0.138545514896064	1.04691664279322	unchanged
Meta_737	LysoPC(16:1(9Z)/0:0)	Lipids and lipid-like molecules	Glycerophospholipids	Glycerophosphocholines	HMDB0010383	C04230	0.287333333333333	0.32418063690346	0.32418063690346	0.722602379930852	unchanged
Meta_739	CerP(d15:0/10:0)	Unclassified	Unclassified	Unclassified			-0.318666667	0.208466783949925	0.208466783949925	0.900832184057222	unchanged
Meta_741	Amppd	Organic acids and derivatives	Organic phosphoric acids and	Phosphate esters	HMDB0248363		0.904333333333334	0.222994064572358	0.222994064572358	0.873640879358382	unchanged
Meta_743	LysoPC(0:0/16:0)	Lipids and lipid-like molecules	Glycerophospholipids	Glycerophosphocholines	HMDB0240262		0.367666666666667	0.307801359974715	0.307801359974715	0.737203630050838	unchanged
Meta_744	LysoPC(16:0/0:0)	Lipids and lipid-like molecules	Glycerophospholipids	Glycerophosphocholines	HMDB0010382	C04230	0.423666666666666	0.125928989785253	0.125928989785253	1.08941620887465	unchanged
Meta_746	Lobarin	Unclassified	Unclassified	Unclassified			-0.224666667	0.610252861994009	0.610252861994009	0.408865453237857	unchanged
Meta_747	LysoPE(20:4(8Z,11Z,14Z,17Z)/0:0)	Lipids and lipid-like molecules	Glycerophospholipids	Glycerophosphoethanolamines	HMDB0011518		-0.301333333	0.439781751046975	0.439781751046975	0.614278343507911	unchanged
Meta_748	Frangulanine	Organic acids and derivatives	Carboxylic acids and derivatives	Amino acids, peptides, and analogues	HMDB0030199	C10003	-1.130666667	0.183159543848525	0.183159543848525	1.06401744721697	unchanged
Meta_749	Methocarbamol	Benzenoids	Phenol ethers	Anisoles	HMDB0014567		0.356666666666666	0.112890815734814	0.112890815734814	1.18779837084274	unchanged
Meta_75	Isoquinoline	Organoheterocyclic compounds	Isoquinolines and derivatives	Unclassified	HMDB0034244	C06323	0.623333333333331	0.0520484604720249	0.0520484604720249	1.2143430507654	unchanged
Meta_750	LysoPE(20:1(11Z)/0:0)	Lipids and lipid-like molecules	Glycerophospholipids	Glycerophosphoethanolamines	HMDB0011512		0.492666666666667	0.0897821147192955	0.0897821147192955	1.10147913454979	unchanged
Meta_751	LPC O-18:2	Unclassified	Unclassified	Unclassified			0.949333333333332	0.107063992161234	0.107063992161234	1.17691192376196	unchanged
Meta_752	[(1S,9S,13R,14R)-14-hydroxy-9-(hydroxymethyl)-5,5-dimethyltetracyclo[11.2.1.0,?.0?,?]hexadecan-14-yl]methyl (2E)-3-(3,4-dihydroxyphenyl)prop-2-enoate	Unclassified	Unclassified	Unclassified			-0.243333333	0.162073417676188	0.162073417676188	0.983346156124123	unchanged
Meta_756	LysoPC(17:0/0:0)	Lipids and lipid-like molecules	Glycerophospholipids	Glycerophosphocholines	HMDB0012108	C04230	0.506333333333334	0.145865082055506	0.145865082055506	1.08434535212404	unchanged
Meta_758	Betamethasone 17 Valerate	Lipids and lipid-like molecules	Steroids and steroid derivatives	Hydroxysteroids	HMDB0249157		0.00733333333333341	0.977596790956546	0.977596790956546	0.00763732788205154	unchanged
Meta_759	Taurocholic acid	Lipids and lipid-like molecules	Steroids and steroid derivatives	Bile acids, alcohols and derivatives	HMDB0000036	C05122	-0.366666667	0.122069697135774	0.122069697135774	1.0285656461976	unchanged
Meta_760	Butyryl trihexyl citrate	Organic acids and derivatives	Carboxylic acids and derivatives	Tetracarboxylic acids and derivatives	HMDB0247498		-0.272666667	0.406863007712719	0.406863007712719	0.610831543561601	unchanged
Meta_761	6-(diaminomethylideneamino)-2-[[(4E,6E,12E,14E)-3-hydroxy-2-(hydroxymethyl)-8,10,16-trimethyloctadeca-4,6,12,14-tetraenoyl]amino]hexanoic acid	Unclassified	Unclassified	Unclassified			0.252666666666666	0.290320607961213	0.290320607961213	0.825124257821768	unchanged
Meta_763	LysoPC(18:2(9Z,12Z)/0:0)	Lipids and lipid-like molecules	Glycerophospholipids	Glycerophosphocholines	HMDB0010386	C04100	0.386333333333335	0.222485151899181	0.222485151899181	0.916140099509653	unchanged
Meta_764	CAR 25:3	Unclassified	Unclassified	Unclassified			-0.075666667	0.703106073702002	0.703106073702002	0.328637748700118	unchanged
Meta_766	1-Oleoylglycerophosphoserine	Lipids and lipid-like molecules	Glycerophospholipids	Glycerophosphoserines	HMDB0061694		0.523666666666669	0.210691612930866	0.210691612930866	0.967336233504586	unchanged
Meta_768	Tridodecylamine	Organic nitrogen compounds	Organonitrogen compounds	Amines	HMDB0037822		0.806000000000001	0.452622499873651	0.452622499873651	0.554263934668479	unchanged
Meta_77	L-Leucine	Organic acids and derivatives	Carboxylic acids and derivatives	Amino acids, peptides, and analogues	HMDB0000687	C00123	0.572999999999997	0.171608325826062	0.171608325826062	0.964539599869109	unchanged
Meta_772	Medicagenic acid	Lipids and lipid-like molecules	Prenol lipids	Triterpenoids	HMDB0034551		0.200000000000001	0.487773766061212	0.487773766061212	0.529355732607655	unchanged
Meta_773	LysoPE(22:6 (4Z,7Z,10Z,13Z,16Z,19Z)/0:0)	Lipids and lipid-like molecules	Glycerophospholipids	Glycerophosphoethanolamines	HMDB0011526		-0.021	0.920529627069984	0.920529627069984	0.0827116411896597	unchanged
Meta_774	LysoPE(22:5(4Z,7Z,10Z,13Z,16Z)/0:0)	Lipids and lipid-like molecules	Glycerophospholipids	Glycerophosphoethanolamines	HMDB0011524		0.418999999999999	0.38153271723946	0.38153271723946	0.65473457145673	unchanged
Meta_775	LPE 22:4	Unclassified	Unclassified	Unclassified			-0.234333333	0.145224050386563	0.145224050386563	1.00354454962864	unchanged
Meta_777	LysoPE(22:4(7Z,10Z,13Z,16Z)/0:0)	Lipids and lipid-like molecules	Glycerophospholipids	Glycerophosphoethanolamines	HMDB0011523		-0.629666667	0.400886033888919	0.400886033888919	0.658522608142728	unchanged
Meta_779	Chenodeoxycholylarginine	Lipids and lipid-like molecules	Steroids and steroid derivatives	Bile acids, alcohols and derivatives	HMDB0242396		-0.425666667	0.302063162319328	0.302063162319328	0.810848030686456	unchanged
Meta_782	LPC O-20:1	Unclassified	Unclassified	Unclassified			-0.441666667	0.545510729105216	0.545510729105216	0.487040009583064	unchanged
Meta_783	[1-(3,3-Dimethyloxiran-2-yl)-3-[(8S,10S,11S,14R)-11-hydroxy-4,4,8,10,14-pentamethyl-3-oxo-1,2,5,6,7,9,11,12,15,16-decahydrocyclopenta[a]phenanthren-17-yl]butyl] acetate	Lipids and lipid-like molecules	Prenol lipids	Triterpenoids	HMDB0248142		0.195333333333334	0.868661962095822	0.868661962095822	0.153778481054437	unchanged
Meta_786	Fellavin; Flacoside	Unclassified	Unclassified	Unclassified			-1.434333333	0.0551248839658525	0.0551248839658525	1.20540236322155	unchanged
Meta_787	Glycocholic acid 3-sulfate	Lipids and lipid-like molecules	Steroids and steroid derivatives	Bile acids, alcohols and derivatives	HMDB0240728		1.54966666666667	0.166506931989211	0.166506931989211	1.07383420432162	unchanged
Meta_788	LysoPC(0:0/20:4(5Z,8Z,11Z,14Z))	Lipids and lipid-like molecules	Glycerophospholipids	Glycerophosphocholines	HMDB0061699		-0.137666667	0.69951971142668	0.69951971142668	0.313094076636178	unchanged
Meta_79	Ethylmalonic acid	Lipids and lipid-like molecules	Fatty Acyls	Fatty acids and conjugates	HMDB0000622		0.165333333333333	0.494528918795466	0.494528918795466	0.512612210570272	unchanged
Meta_791	Chenodeoxycholylmethionine	Lipids and lipid-like molecules	Steroids and steroid derivatives	Bile acids, alcohols and derivatives	HMDB0242403		-0.917666667	0.0947853516977952	0.0947853516977952	1.09852088478939	unchanged

Meta_792	6-bromo-4-phenyl-3-[3-phenyl-2-(pyridine-3-carbonyl)-3,4-dihydropyrazol-5-yl]-1H-quinolin-2-one	Unclassified	Unclassified	Unclassified			-0.169333333	0.783355769927222	0.783355769927222	0.208863973470289	unchanged
Meta_794	Cerubidin	Phenylpropanoids and polyketides	Anthracyclines	Unclassified	HMDB0255779		-0.304666667	0.076640145359824	0.076640145359824	1.16294804928471	unchanged
Meta_797	Lyoniside	Lignans, neolignans and related compounds	Lignan glycosides	Unclassified	HMDB0242693		-0.380333333	0.146556162866453	0.146556162866453	0.988077842524593	unchanged
Meta_798	LysoPC(20:0/0:0)	Lipids and lipid-like molecules	Glycerophospholipids	Glycerophosphocholines	HMDB0010390	C04230	1.07366666666667	0.120710154426865	0.120710154426865	1.14564873208166	unchanged
Meta_799	Cer(d18:1/14:0)	Lipids and lipid-like molecules	Sphingolipids	Ceramides	HMDB0011773	C13916	0.142000000000001	0.4370802829545	0.4370802829545	0.589396248784781	unchanged
Meta_80	Methylsuccinic acid	Lipids and lipid-like molecules	Fatty Acyls	Fatty acids and conjugates	HMDB0001844		0.137333333333334	0.201797069905405	0.201797069905405	0.953321492081747	unchanged
Meta_800	Octaethyleneglycol monododecyl ether	Organic oxygen compounds	Organooxygen compounds	Ethers	HMDB0246126		0.212333333333333	0.619908389764569	0.619908389764569	0.43532043790867	unchanged
Meta_801	Telmisartan	Benzenoids	Benzene and substituted derivatives	Biphenyls and derivatives	HMDB0015101	C07710	-0.088	0.549126305721724	0.549126305721724	0.476603496382896	unchanged
Meta_802	DGCC 8:0_12:0	Unclassified	Unclassified	Unclassified			1.28533333333333	0.506722955571642	0.506722955571642	0.527231179526303	unchanged
Meta_803	SM 8:0;2O/16:2	Unclassified	Unclassified	Unclassified			-0.176333333	0.318136126409614	0.318136126409614	0.772322634505736	unchanged
Meta_804	Uridine diphosphate glucose	Nucleosides, nucleotides, and	Pyrimidine nucleotides	Pyrimidine nucleotide sugars	HMDB0000286	C00029	-0.125333333	0.291866536268255	0.291866536268255	0.856054145000066	unchanged
Meta_805	LysoPC(20:4(5Z,8Z,11Z,14Z)/0:0)	Lipids and lipid-like molecules	Glycerophospholipids	Glycerophosphocholines	HMDB0010395	C04230	0.135666666666666	0.667957196768599	0.667957196768599	0.314418406345142	unchanged
Meta_806	LysoPC(18:1(9Z)/0:0)	Lipids and lipid-like molecules	Glycerophospholipids	Glycerophosphocholines	HMDB0002815		2.025	0.21814103333309	0.21814103333309	0.952592477642757	unchanged
Meta_807	LPC O-22:0	Unclassified	Unclassified	Unclassified			1.931	0.0789915148640883	0.0789915148640883	1.24999380347427	unchanged
Meta_808	LysoPC(18:0/0:0)	Lipids and lipid-like molecules	Glycerophospholipids	Glycerophosphocholines	HMDB0010384		0.270333333333333	0.254838011340809	0.254838011340809	0.809727984255142	unchanged
Meta_809	Cer(d17:1/16:0)	Lipids and lipid-like molecules	Sphingolipids	Ceramides	HMDB0240685		-0.439	0.128024108907124	0.128024108907124	1.09990792004936	unchanged
Meta_810	Inuline	Lipids and lipid-like molecules	Prenol lipids	Diterpenoids	HMDB0248466		-0.384333333	0.193620791951663	0.193620791951663	0.962433480798312	unchanged
Meta_811	MGDG O-13:1_7:0	Unclassified	Unclassified	Unclassified			-0.056666667	0.827739175099412	0.827739175099412	0.185243018380037	unchanged
Meta_812	SM 8:0;2O/17:2	Unclassified	Unclassified	Unclassified			-0.471	0.27028691978479	0.27028691978479	0.844259133912694	unchanged
Meta_813	DG(18:0/10:0/0:0)	Lipids and lipid-like molecules	Glycerolipids	Diradylglycerols	HMDB0093890		-0.097	0.522164822429705	0.522164822429705	0.527922273049048	unchanged
Meta_818	MGDG O-16:2_5:0	Unclassified	Unclassified	Unclassified			0.0570000000000004	0.964628639813282	0.964628639813282	0.0575362821349727	unchanged
Meta_819	Methyl 3b-acetoxy-2,6-dihydroxy-8a,30a-epoxy-1-oxomeliacate	Unclassified	Unclassified	Unclassified			-0.683333333	0.0778669925559628	0.0778669925559628	1.14809193917007	unchanged
Meta_821	LysoPC(20:4(8Z,11Z,14Z,17Z)/0:0)	Lipids and lipid-like molecules	Glycerophospholipids	Glycerophosphocholines	HMDB0010396		0.424999999999999	0.0959572382967054	0.0959572382967054	1.12053121747505	unchanged
Meta_822	Caffeoylcycloartenol	Lipids and lipid-like molecules	Steroids and steroid derivatives	Cycloartanols and derivatives	HMDB0040807		-0.386333333	0.130764725107005	0.130764725107005	1.07816944467492	unchanged
Meta_823	SMGDG O-16:4_2:0	Unclassified	Unclassified	Unclassified			0.198	0.188887053383109	0.188887053383109	0.961461336498046	unchanged
Meta_824	Polidocanol	Organic oxygen compounds	Organooxygen compounds	Ethers	HMDB0256665	C13493	-0.103333333	0.393779161036507	0.393779161036507	0.684659674342566	unchanged
Meta_825	DG 34:7	Unclassified	Unclassified	Unclassified			-0.134333333	0.422950658218966	0.422950658218966	0.601343562553966	unchanged
Meta_826	Uridine diphosphate-N-acetylglucosamine	Nucleosides, nucleotides, and analogues	Pyrimidine nucleotides	Pyrimidine nucleotide sugars	HMDB0000290	C00043	0.324666666666669	0.605026429790003	0.605026429790003	0.408907371922376	unchanged
Meta_829	DG 35:7	Unclassified	Unclassified	Unclassified			-0.196666667	0.376870359132636	0.376870359132636	0.734767032828856	unchanged
Meta_83	L-Aspartic acid	Organic acids and derivatives	Carboxylic acids and derivatives	Amino acids, peptides, and analogues	HMDB0000191	C00049	-0.438	0.357647741596586	0.357647741596586	0.673914857225716	unchanged
Meta_831	DG(14:1(9Z)/22:6(4Z,7Z,10Z,13Z,16Z,19Z)/0:0)	Lipids and lipid-like molecules	Glycerolipids	Diradylglycerols	HMDB0007063		0.257	0.290676202158786	0.290676202158786	0.84045765792499	unchanged
Meta_833	SM(d18:1/12:0)	Lipids and lipid-like molecules	Sphingolipids	Phosphosphingolipids	HMDB0012096	C00550	-0.237333333	0.213473075738621	0.213473075738621	0.901856905279339	unchanged
Meta_834	PE(14:1(9Z)/15:0)	Lipids and lipid-like molecules	Glycerophospholipids	Glycerophosphoethanolamines	HMDB0008856		0.775666666666666	0.652789195130254	0.652789195130254	0.368195344921068	unchanged
Meta_835	Dechlorane plus	Organohalogen compounds	Vinyl halides	Vinyl chlorides	HMDB0250920		1.00233333333333	0.4267232318281	0.4267232318281	0.600762503530657	unchanged
Meta_836	Gossypetin 8-glucuronide 3-glucoside	Phenylpropanoids and polyketides	Flavonoids	Flavonoid glycosides	HMDB0039103		-0.018666667	0.981891948659053	0.981891948659053	0.00945348041363979	unchanged
Meta_837	PE-Cer 12:0;2O/22:1	Unclassified	Unclassified	Unclassified			0.0173333333333332	0.96318126297877	0.96318126297877	0.0375244202725317	unchanged
Meta_838	PE(14:0/16:0)	Lipids and lipid-like molecules	Glycerophospholipids	Glycerophosphoethanolamines	HMDB0008824		-0.445333333	0.405126920570818	0.405126920570818	0.680889177114756	unchanged
Meta_839	PC O-28:1	Unclassified	Unclassified	Unclassified			-0.473333333	0.157749289223801	0.157749289223801	0.983134661253404	unchanged
Meta_84	Creatine	Organic acids and derivatives	Carboxylic acids and derivatives	Amino acids, peptides, and analogues	HMDB0000064	C00300	-0.566333333	0.174948182083388	0.174948182083388	1.03564051757579	unchanged
Meta_840	PC O-28:0	Unclassified	Unclassified	Unclassified			-0.327333333	0.104120509649631	0.104120509649631	1.17059962120055	unchanged
Meta_841	Cer(d18:1/22:0)	Lipids and lipid-like molecules	Sphingolipids	Ceramides	HMDB0004952	C00195	-1.635666667	0.146747900583432	0.146747900583432	1.01080506762693	unchanged
Meta_842	Haploperozide	Unclassified	Unclassified	Unclassified			0.427666666666667	0.144031757731291	0.144031757731291	1.07283638428276	unchanged
Meta_843	PA(13:0/18:1(12Z)-O(9S,10R))	Unclassified	Unclassified	Unclassified	HMDB0262906		0.115	0.93047969052261	0.93047969052261	0.0917425676241517	unchanged
Meta_844	PE O-16:1_16:1	Unclassified	Unclassified	Unclassified			0.0586666666666655	0.721961273457721	0.721961273457721	0.261091645738862	unchanged
Meta_845	PE O-16:1_16:0	Unclassified	Unclassified	Unclassified			-0.928333333	0.176539269084428	0.176539269084428	1.04214363020556	unchanged
Meta_847	Veratroylzygadenine	Unclassified	Unclassified	Unclassified			-0.203666667	0.689336065798976	0.689336065798976	0.325366818458189	unchanged
Meta_848	PC O-12:0_14:0	Unclassified	Unclassified	Unclassified			-0.071333333	0.94359178723186	0.94359178723186	0.0595033434012063	unchanged
Meta_849	SM(d17:2(4E,8Z)/16:0)	Organic nitrogen compounds	Organonitrogen compounds	Quaternary ammonium salts	HMDB0240677		-0.004333333	0.970971892163068	0.970971892163068	0.0356155562809669	unchanged
Meta_850	Dephospho-CoA	Nucleosides, nucleotides, and	Purine nucleotides	Purine ribonucleotides	HMDB0001373	C00882	-0.018666667	0.919035456063014	0.919035456063014	0.0839207326065801	unchanged
Meta_851	LysoPC(28:0/0:0)	Lipids and lipid-like molecules	Glycerophospholipids	Glycerophosphocholines	HMDB0029206		-0.162	0.589596149330127	0.589596149330127	0.423043835961473	unchanged
Meta_853	PC O-30:2	Unclassified	Unclassified	Unclassified			-0.224666667	0.644447605348766	0.644447605348766	0.374342270550601	unchanged
Meta_854	PE-Cer 12:0;2O/24:0	Unclassified	Unclassified	Unclassified			-0.002666667	0.995542153799336	0.995542153799336	0.00261928495707218	unchanged
Meta_855	PC O-30:1	Unclassified	Unclassified	Unclassified			0.242333333333331	0.0504577649782769	0.0504577649782769	1.21088872564342	unchanged
Meta_857	Cer(d18:1/24:1(15Z))	Lipids and lipid-like molecules	Sphingolipids	Ceramides	HMDB0004953	C00195	-1.495666667	0.186724636201514	0.186724636201514	1.03373856032004	unchanged
Meta_858	PE O-16:1_18:2	Unclassified	Unclassified	Unclassified			-0.717	0.18903921909129	0.18903921909129	0.968895620269671	unchanged
Meta_86	L-Norleucine	Organic acids and derivatives	Carboxylic acids and derivatives	Amino acids, peptides, and analogues	HMDB0001645	C01933	0.145999999999997	0.132761925186399	0.132761925186399	1.10683253702541	unchanged
Meta_860	PE O-16:1_18:1	Unclassified	Unclassified	Unclassified			-0.353333333	0.0888015570733363	0.0888015570733363	1.24066724690881	unchanged
Meta_861	NCGC00385360-01_C36H56O12_beta-D-Glucopyranose, 1-O-[(2alpha,3beta,5xi,9xi,18xi)-2,3,19,24-tetrahydroxy-24,28-dioxoolean-12-en-28-yl]-	Unclassified	Unclassified	Unclassified			-0.633	0.106195387162796	0.106195387162796	1.08262029618395	unchanged
Meta_862	SM 8:1;2O/23:0	Unclassified	Unclassified	Unclassified			0.534000000000001	0.557233960034141	0.557233960034141	0.493714417854306	unchanged
Meta_864	PC O-31:0	Unclassified	Unclassified	Unclassified			-0.368	0.0592799091346926	0.0592799091346926	1.29130901908314	unchanged

Meta_865	Cer(d17:1/LTE4)	Unclassified	Unclassified	Unclassified	HMDB0289906		-0.147333333	0.677126397204398	0.677126397204398	0.331460916202635	unchanged
Meta_866	PC(14:0/14:1(9Z))	Lipids and lipid-like molecules	Glycerophospholipids	Glycerophosphocholines	HMDB0007867		-0.427	0.48359432317047	0.48359432317047	0.562116575982377	unchanged
Meta_868	LPC 30:1	Unclassified	Unclassified	Unclassified			0.394333333333332	0.220067743590764	0.220067743590764	0.858806231014491	unchanged
Meta_87	3H-1,2-Dithiole-3-thione	Organoheterocyclic compounds	Dithioles	1,2-dithioles	HMDB0035702		-0.293	0.759595310990019	0.759595310990019	0.234895589783302	unchanged
Meta_870	PA(15:0/PGJ2)	Unclassified	Unclassified	Unclassified	HMDB0263194		0.136333333333333	0.926198497674333	0.926198497674333	0.0940383181839416	unchanged
Meta_871	Parthenocissin A	Lipids and lipid-like molecules	Glycerophospholipids	Glycerophosphocholines	HMDB0304716		-0.399333333	0.276036433631762	0.276036433631762	0.871967812555269	unchanged
Meta_872	PE(16:1(9Z)/18:1(9Z))	Lipids and lipid-like molecules	Glycerophospholipids	Glycerophosphoethanolamines	HMDB0008960		-0.007333333	0.915852685466952	0.915852685466952	0.0835077885490986	unchanged
Meta_873	PC(14:1(9Z)/P-18:1(9Z))	Lipids and lipid-like molecules	Glycerophospholipids	Glycerophosphocholines	HMDB0007931		-0.681666667	0.0509662791576288	0.0509662791576288	1.2483185526661	unchanged
Meta_874	LNAPE 16:1/N-18:0	Unclassified	Unclassified	Unclassified			-0.570666667	0.0877723455846163	0.0877723455846163	1.10297380959071	unchanged
Meta_875	PE(16:0/18:1(9Z))	Lipids and lipid-like molecules	Glycerophospholipids	Glycerophosphoethanolamines	HMDB0008927	C13877	-0.123666667	0.136540364180262	0.136540364180262	1.03376164113562	unchanged
Meta_876	HexCer 8:0;2O/24:1	Unclassified	Unclassified	Unclassified			-0.273	0.648238185583966	0.648238185583966	0.364851231554611	unchanged
Meta_877	PC O-32:2	Unclassified	Unclassified	Unclassified			0.15366666666667	0.716993059210266	0.716993059210266	0.29474333120698	unchanged
Meta_878	SM 8:0;2O/24:2	Unclassified	Unclassified	Unclassified			-0.076	0.796083388888679	0.796083388888679	0.203279081965419	unchanged
Meta_879	PC O-32:1	Unclassified	Unclassified	Unclassified			0.0463333333333367	0.621808005814724	0.621808005814724	0.391072035972917	unchanged
Meta_881	PC 12:0_16:1	Unclassified	Unclassified	Unclassified			0.781666666666666	0.205213950159631	0.205213950159631	1.00187223763131	unchanged
Meta_885	PE(20:4(5Z,8Z,11Z,14Z)/P-16:0)	Lipids and lipid-like molecules	Glycerophospholipids	Glycerophosphoethanolamines	HMDB0009411		-0.244666667	0.385577633771095	0.385577633771095	0.681040317094602	unchanged
Meta_889	LNAPE 16:1/N-19:1	Unclassified	Unclassified	Unclassified			-0.303666667	0.137604019221846	0.137604019221846	1.09945159530703	unchanged
Meta_892	LNAPE 18:1/N-17:0	Unclassified	Unclassified	Unclassified			0.202333333333334	0.647099153451974	0.647099153451974	0.360670596922271	unchanged
Meta_893	PC O-33:2	Unclassified	Unclassified	Unclassified			0.567333333333334	0.0733492431927683	0.0733492431927683	1.23971604067846	unchanged
Meta_895	PS(16:0/16:1(9Z))	Lipids and lipid-like molecules	Glycerophospholipids	Glycerophosphoserines	HMDB0012355		-0.402666667	0.590195521387084	0.590195521387084	0.445765960967629	unchanged
Meta_896	PC(18:1(9Z)/14:0)	Lipids and lipid-like molecules	Glycerophospholipids	Glycerophosphocholines	HMDB0008097		-0.671666667	0.561304026384233	0.561304026384233	0.478386314252083	unchanged
Meta_899	PC O-16:1_14:0	Unclassified	Unclassified	Unclassified			0.173666666666668	0.0904368790106029	0.0904368790106029	1.1634503371448	unchanged
Meta_9	Pyrrolidine	Organoheterocyclic compounds	Pyrrolidines	Unclassified	HMDB0031641		0.151	0.134409786283597	0.134409786283597	1.0192620451867	unchanged
Meta_901	PC O-33:0	Unclassified	Unclassified	Unclassified			-0.036	0.934368703155396	0.934368703155396	0.0599680962631852	unchanged
Meta_902	PE(16:1(9Z)/20:4(8Z,11Z,14Z,17Z))	Lipids and lipid-like molecules	Glycerophospholipids	Glycerophosphoethanolamines	HMDB0008971		-0.200333333	0.111059813972281	0.111059813972281	1.10360440855188	unchanged
Meta_903	PC(14:0/15:0)	Lipids and lipid-like molecules	Glycerophospholipids	Glycerophosphocholines	HMDB0007868		0.310000000000001	0.268087668808176	0.268087668808176	0.890897664681002	unchanged
Meta_904	PS(16:0/16:0)	Lipids and lipid-like molecules	Glycerophospholipids	Glycerophosphoserines	HMDB0000614		-0.527	0.782792996102888	0.782792996102888	0.201487851998422	unchanged
Meta_906	PA(20:0/20:2(11Z,14Z))	Lipids and lipid-like molecules	Glycerophospholipids	Glycerophosphates	HMDB0115528		-0.330666667	0.10314637068499	0.10314637068499	1.07952886756376	unchanged
Meta_907	PE(16:0/20:4(8Z,11Z,14Z,17Z))	Lipids and lipid-like molecules	Glycerophospholipids	Glycerophosphoethanolamines	HMDB0008938		-0.003666667	0.984260111022517	0.984260111022517	0.0143526520350702	unchanged
Meta_908	PC(14:0/P-18:1(11Z))	Lipids and lipid-like molecules	Glycerophospholipids	Glycerophosphocholines	HMDB0007897	C00157	0.193666666666667	0.347972630840553	0.347972630840553	0.721622349500241	unchanged
Meta_909	PC 31:1	Unclassified	Unclassified	Unclassified			0.261999999999999	0.360064981220997	0.360064981220997	0.657790807498437	unchanged
Meta_91	Thioproline	Organic acids and derivatives	Carboxylic acids and derivatives	Amino acids, peptides, and analogues	HMDB0062164		0.265333333333334	0.750258476382465	0.750258476382465	0.252627245120141	unchanged
Meta_910	PE 34:1	Unclassified	Unclassified	Unclassified			0.0463333333333331	0.733329154119794	0.733329154119794	0.247244945094606	unchanged
Meta_911	PE(18:1(9Z)/18:2(9Z,12Z))	Lipids and lipid-like molecules	Glycerophospholipids	Glycerophosphoethanolamines	HMDB0009060		0.198	0.354125417735363	0.354125417735363	0.768561272620015	unchanged
Meta_912	PC(P-16:0/18:3(6Z,9Z,12Z))	Lipids and lipid-like molecules	Glycerophospholipids	Glycerophosphocholines	HMDB0011212		0.276333333333334	0.320839209046478	0.320839209046478	0.794000241831159	unchanged
Meta_913	PE(18:1(9Z)/18:1(9Z))	Lipids and lipid-like molecules	Glycerophospholipids	Glycerophosphoethanolamines	HMDB0009059		-0.132333333	0.525829903063772	0.525829903063772	0.482186412401453	unchanged
Meta_915	PC O-34:3	Unclassified	Unclassified	Unclassified			-0.004666667	0.989049350926558	0.989049350926558	0.00710163252387023	unchanged
Meta_917	PC(16:1(9Z)/P-18:0)	Lipids and lipid-like molecules	Glycerophospholipids	Glycerophosphocholines	HMDB0008028	C00157	-0.109666667	0.252557080184422	0.252557080184422	0.925134202358552	unchanged
Meta_918	SM 8:0;2O/26:2	Unclassified	Unclassified	Unclassified			-0.246666667	0.228376984610746	0.228376984610746	0.971324640214612	unchanged
Meta_919	PE O-16:1_22:6	Unclassified	Unclassified	Unclassified			-0.149666667	0.415446565580173	0.415446565580173	0.606274071158942	unchanged
Meta_92	Hydroxypropionic acid	Organic acids and derivatives	Hydroxy acids and derivatives	Beta hydroxy acids and derivatives	HMDB0000700	C01013	-0.215666667	0.564981299322937	0.564981299322937	0.433402601144217	unchanged
Meta_924	PC(15:0/P-16:0)	Lipids and lipid-like molecules	Glycerophospholipids	Glycerophosphocholines	HMDB0007961	C00157	-1.011666667	0.176205506155473	0.176205506155473	0.959364374781878	unchanged
Meta_926	PE O-18:1_20:4	Unclassified	Unclassified	Unclassified			-0.146	0.316593236994077	0.316593236994077	0.766925147643924	unchanged
Meta_928	PE O-16:1_22:4	Unclassified	Unclassified	Unclassified			-0.309333333	0.0865597974943401	0.0865597974943401	1.17503309564752	unchanged
Meta_929	PC 32:2	Unclassified	Unclassified	Unclassified			-0.182333333	0.615366527393578	0.615366527393578	0.405102805806023	unchanged
Meta_93	Adenine	Organoheterocyclic compounds	Imidazopyrimidines	Purines and purine derivatives	HMDB0000034	C00147	-0.343666667	0.160007994674842	0.160007994674842	0.992189932411374	unchanged
Meta_930	PC 32:1	Unclassified	Unclassified	Unclassified			-0.277666667	0.153702458170316	0.153702458170316	1.096428283	unchanged
Meta_932	PC O-12:0_20:4	Unclassified	Unclassified	Unclassified			-0.79	0.255480994834069	0.255480994834069	0.817169481799651	unchanged
Meta_933	PA(15:0/PGF1alpha)	Unclassified	Unclassified	Unclassified	HMDB0263258		0.182	0.897545526529924	0.897545526529924	0.121490887214577	unchanged
Meta_934	PC(16:0/18:2(9Z,12Z))	Lipids and lipid-like molecules	Glycerophospholipids	Glycerophosphocholines	HMDB0007973		-0.602	0.213178021004996	0.213178021004996	0.964072689383275	unchanged
Meta_935	PC O-35:2	Unclassified	Unclassified	Unclassified			0.382333333333333	0.227565464007904	0.227565464007904	0.893291924701173	unchanged
Meta_936	PC(O-16:1(9Z)/16:1(9Z))	Lipids and lipid-like molecules	Glycerophospholipids	Glycerophosphocholines	HMDB0013411		-0.178333333	0.387642319616568	0.387642319616568	0.6887024511764	unchanged
Meta_939	PC(15:0/16:1(9Z))	Lipids and lipid-like molecules	Glycerophospholipids	Glycerophosphocholines	HMDB0007936		0.280333333333335	0.259747764781047	0.259747764781047	0.841292607811077	unchanged
Meta_940	PC O-16:1_16:0	Unclassified	Unclassified	Unclassified			-0.399333333	0.104789965111161	0.104789965111161	1.19715958099218	unchanged
Meta_941	PC O-18:1_14:0	Unclassified	Unclassified	Unclassified			0.0596666666666685	0.486336635095602	0.486336635095602	0.526299693060722	unchanged
Meta_943	PE(18:1(9Z)/20:4(8Z,11Z,14Z,17Z))	Lipids and lipid-like molecules	Glycerophospholipids	Glycerophosphoethanolamines	HMDB0009070		0.0539999999999985	0.790974718565392	0.790974718565392	0.209463380990602	unchanged
Meta_946	PE(18:0/20:4(8Z,11Z,14Z,17Z))	Lipids and lipid-like molecules	Glycerophospholipids	Glycerophosphoethanolamines	HMDB0009004		-0.098666667	0.370119371320991	0.370119371320991	0.680424501097551	unchanged
Meta_947	PC(P-16:0/20:4(5Z,8Z,11Z,14Z))	Lipids and lipid-like molecules	Glycerophospholipids	Glycerophosphocholines	HMDB0011220		-0.056666667	0.823574397607939	0.823574397607939	0.170359970966904	unchanged
Meta_948	PC(O-16:0/20:5(5Z,8Z,11Z,14Z,17Z))	Lipids and lipid-like molecules	Glycerophospholipids	Glycerophosphocholines	HMDB0039528		0.312333333333335	0.079407960700167	0.079407960700167	1.26044845716454	unchanged
Meta_949	PC(P-16:0/20:3(5Z,8Z,11Z))	Lipids and lipid-like molecules	Glycerophospholipids	Glycerophosphocholines	HMDB0011218		-0.182	0.619508493040863	0.619508493040863	0.418771778740232	unchanged
Meta_950	PC O-36:3	Unclassified	Unclassified	Unclassified			0.0453333333333355	0.89698988822168	0.89698988822168	0.0911315002072249	unchanged
Meta_952	PC O-36:2	Unclassified	Unclassified	Unclassified			-0.012	0.961620014964875	0.961620014964875	0.0232727033827606	unchanged
Meta_953	PG(18:1(9Z)/18:1(9Z))	Lipids and lipid-like molecules	Glycerophospholipids	Glycerophosphoglycerols	HMDB0010634		-0.201	0.457895962522449	0.457895962522449	0.62315341338426	unchanged
Meta_954	PC(16:1(9Z)/16:1(9Z))	Lipids and lipid-like molecules	Glycerophospholipids	Glycerophosphocholines	HMDB0008002		0.236000000000001	0.150909408595059	0.150909408595059	1.02482442072173	unchanged
Meta_955	PC 16:0_16:2	Unclassified	Unclassified	Unclassified			-0.077333333	0.649618129374363	0.649618129374363	0.382277384257173	unchanged
Meta_957	PC(14:0/18:1(9Z))	Lipids and lipid-like molecules	Glycerophospholipids	Glycerophosphocholines	HMDB0007873		-0.086	0.481164188727148	0.481164188727148	0.585051392268503	unchanged
Meta_958	PC(16:0/16:1(9Z))	Lipids and lipid-like molecules	Glycerophospholipids	Glycerophosphocholines	HMDB0007969		-0.086666667	0.522617136114907	0.522617136114907	0.484424737764137	unchanged
Meta_959	PC(P-16:0/18:1(12Z)-2OH(9,10))	Unclassified	Unclassified	Unclassified	HMDB0289479		-0.553666667	0.165557419619543	0.165557419619543	1.03528994446606	unchanged
Meta_96	Hypoxanthine	Organoheterocyclic compounds	Imidazopyrimidines	Purines and purine derivatives	HMDB0000157	C00262	0.0793333333333344	0.835702716011503	0.835702716011503	0.164407706889911	unchanged
Meta_960	PC O-16:0_17:1	Unclassified	Unclassified	Unclassified			0.174333333333333	0.348217100974367	0.348217100974367	0.715205136673462	unchanged

Meta_961	SM 8:0;2O/28:0	Unclassified	Unclassified	Unclassified			-0.115	0.801205676211263	0.801205676211263	0.194901986946246	unchanged
Meta_962	PC 34:3	Unclassified	Unclassified	Unclassified			0.67	0.102635496005683	0.102635496005683	1.18231517586532	unchanged
Meta_964	PC 34:2	Unclassified	Unclassified	Unclassified			0.219333333333331	0.571892379707442	0.571892379707442	0.471384483145548	unchanged
Meta_965	PC O-37:4	Unclassified	Unclassified	Unclassified			-0.305666667	0.302724600680236	0.302724600680236	0.760405051294485	unchanged
Meta_966	FAD	Nucleosides, nucleotides, and	Flavin nucleotides	Unclassified	HMDB0001248	C00016	0.281000000000002	0.177394109471412	0.177394109471412	0.980457512921887	unchanged
Meta_968	PC(O-16:1(9Z)/18:2(9Z,12Z))	Lipids and lipid-like molecules	Glycerophospholipids	Glycerophosphocholines	HMDB0013413		-0.547	0.166463052814878	0.166463052814878	1.04874454737847	unchanged
Meta_969	PC(18:0/18:2(9Z,12Z))	Lipids and lipid-like molecules	Glycerophospholipids	Glycerophosphocholines	HMDB0008039		-0.244666667	0.351105946795793	0.351105946795793	0.687879733323437	unchanged
Meta_970	PC 16:1_17:1	Unclassified	Unclassified	Unclassified			0.572333333333333	0.406030665649466	0.406030665649466	0.689778766346873	unchanged
Meta_971	PS(18:0/18:1(9Z))	Lipids and lipid-like molecules	Glycerophospholipids	Glycerophosphoserines	HMDB0010163		-0.234	0.681921249134161	0.681921249134161	0.335725230056008	unchanged
Meta_973	PC O-16:1_18:1	Unclassified	Unclassified	Unclassified			-0.092333333	0.24052186056544	0.24052186056544	0.845454919441788	unchanged
Meta_974	PC 16:0_17:1	Unclassified	Unclassified	Unclassified			-0.413	0.156232429713947	0.156232429713947	1.08419287626253	unchanged
Meta_975	PC O-38:7	Unclassified	Unclassified	Unclassified			0.0553333333333335	0.822892346646276	0.822892346646276	0.187687986194867	unchanged
Meta_976	PC O-16:0_18:1	Unclassified	Unclassified	Unclassified			-0.210666667	0.169147731228473	0.169147731228473	1.036433618	unchanged
Meta_977	PE(20:1(11Z)/20:4(8Z,11Z,14Z,17Z))	Lipids and lipid-like molecules	Glycerophospholipids	Glycerophosphoethanolamines	HMDB0009268		0.191666666666666	0.601082693445328	0.601082693445328	0.451042714556635	unchanged
Meta_979	PC O-38:6	Unclassified	Unclassified	Unclassified			0.354333333333333	0.35269285184405	0.35269285184405	0.729743744322499	unchanged
Meta_98	4-Nitrophenol	Benzenoids	Phenols	Nitrophenols	HMDB0001232	C00870	0.114333333333333	0.678818310259602	0.678818310259602	0.336894389956339	unchanged
Meta_981	PC O-38:5	Unclassified	Unclassified	Unclassified			-0.240333333	0.41818121239795	0.41818121239795	0.62128808841315	unchanged
Meta_982	PC O-38:4	Unclassified	Unclassified	Unclassified			-0.044333333	0.921374061510781	0.921374061510781	0.0689159931200548	unchanged
Meta_983	PC(14:0/20:4(8Z,11Z,14Z,17Z))	Lipids and lipid-like molecules	Glycerophospholipids	Glycerophosphocholines	HMDB0007884		0.291666666666666	0.155785303092841	0.155785303092841	0.964982307215482	unchanged
Meta_984	PC(16:1(9Z)/18:2(9Z,12Z))	Lipids and lipid-like molecules	Glycerophospholipids	Glycerophosphocholines	HMDB0008006		-0.316	0.23041564124253	0.23041564124253	0.89115549047275	unchanged
Meta_985	PC 36:5	Unclassified	Unclassified	Unclassified			0.305999999999999	0.192201051989184	0.192201051989184	0.981910846248576	unchanged
Meta_986	PC 36:3	Unclassified	Unclassified	Unclassified			0.247	0.227122570298201	0.227122570298201	0.965323085737253	unchanged
Meta_988	PS(18:1(9Z)/18:1(9Z))	Lipids and lipid-like molecules	Glycerophospholipids	Glycerophosphoserines	HMDB0012390		0.631666666666666	0.409271431534778	0.409271431534778	0.647049002717329	unchanged
Meta_989	PC 36:2	Unclassified	Unclassified	Unclassified			0.0136666666666692	0.893989907866639	0.893989907866639	0.126643225228375	unchanged
Meta_99	2,6-Dimethylbenzenethiol	Benzenoids	Thiophenols	Unclassified	HMDB0032019		-0.189666667	0.450366532609566	0.450366532609566	0.570932871631401	unchanged
Meta_991	PC 36:1	Unclassified	Unclassified	Unclassified			-0.587333333	0.291949747136368	0.291949747136368	0.774378081453294	unchanged
Meta_992	PC(O-16:0/20:4(8Z,11Z,14Z,17Z))	Lipids and lipid-like molecules	Glycerophospholipids	Glycerophosphocholines	HMDB0013407		-0.066	0.659941813899596	0.659941813899596	0.355817848407379	unchanged
Meta_993	PC 17:1_18:2	Unclassified	Unclassified	Unclassified			0.530333333333333	0.246402636911314	0.246402636911314	0.912494119785924	unchanged
Meta_994	PC O-18:2_18:1	Unclassified	Unclassified	Unclassified			-0.231666667	0.264691267515802	0.264691267515802	0.842118074292674	unchanged
Meta_996	PC 17:1_18:1	Unclassified	Unclassified	Unclassified			-0.247333333	0.0634822465765867	0.0634822465765867	1.23805311222864	unchanged
Meta_997	PC(P-18:1(11Z)/22:6(4Z,7Z,10Z,13Z,16Z,19Z))	Lipids and lipid-like molecules	Glycerophospholipids	Glycerophosphocholines	HMDB0011295		-0.28	0.342919617859191	0.342919617859191	0.756725596158104	unchanged
Meta_998	PC O-40:8	Unclassified	Unclassified	Unclassified			0.125	0.500160824620251	0.500160824620251	0.566588822231323	unchanged
Meta_999	PC O-18:1_18:1	Unclassified	Unclassified	Unclassified			-0.188333333	0.215595310363136	0.215595310363136	0.895380473352962	unchanged
Meta_1005	PC(16:0/20:5(5Z,8Z,11Z,14Z,17Z))	Lipids and lipid-like molecules	Glycerophospholipids	Glycerophosphocholines	HMDB0007984		0.491333333333333	0.0094656938347705	0.0094656938347705	1.36646841375972	up
Meta_1012	PC 38:5	Unclassified	Unclassified	Unclassified			0.376666666666667	0.0293661360211476	0.0293661360211476	1.35586361127983	up
Meta_1018	PC(18:0/18:0)	Lipids and lipid-like molecules	Glycerophospholipids	Glycerophosphocholines	HMDB0008036		0.539000000000002	0.00101874342875417	0.00101874342875417	1.45003226903409	up
Meta_103	2-Aminoadipic acid	Organic acids and derivatives	Carboxylic acids and derivatives	Amino acids, peptides, and analogues	HMDB0302754	C00956	0.377333333333334	0.0223475701094956	0.0223475701094956	1.31138677543969	up
Meta_1030	PC(18:1(9Z)/20:4(8Z,11Z,14Z,17Z))	Lipids and lipid-like molecules	Glycerophospholipids	Glycerophosphocholines	HMDB0008115		0.350999999999999	0.00889068263263447	0.00889068263263447	1.38351110212322	up
Meta_104	1-Naphthylamine	Benzenoids	Naphthalenes	Unclassified	HMDB0243964	C14790	0.827999999999999	0.00639855335002019	0.00639855335002019	1.42750406031699	up
Meta_108	Indole-3-carboxaldehyde	Organoheterocyclic compounds	Indoles and derivatives	Indoles	HMDB0029737	C08493	0.696000000000002	0.0385827762782468	0.0385827762782468	1.33106906423735	up
Meta_109	2-Aminoheptanoate	Organic acids and derivatives	Carboxylic acids and derivatives	Amino acids, peptides, and analogues	HMDB0094649		0.906333333333333	0.00563850652208841	0.00563850652208841	1.43623946887485	up
Meta_112	L-2-Hydroxyglutaric acid	Organic acids and derivatives	Hydroxy acids and derivatives	Short-chain hydroxy acids and derivatives	HMDB0000694	C03196	0.537666666666667	0.00728657495067402	0.00728657495067402	1.38872102633881	up
Meta_115	Phthalic anhydride	Organoheterocyclic compounds	Benzofurans	Benzofuranones	HMDB0256501		0.855666666666666	0.0208085320476164	0.0208085320476164	1.38317417335152	up
Meta_116	L-Methionine	Organic acids and derivatives	Carboxylic acids and derivatives	Amino acids, peptides, and analogues	HMDB0000696	C00073	0.552	0.0409240889547814	0.0409240889547814	1.3257526837661	up
Meta_122	L-Histidine	Organic acids and derivatives	Carboxylic acids and derivatives	Amino acids, peptides, and analogues	HMDB0000177	C00135	0.320666666666668	0.0114205492114132	0.0114205492114132	1.36290193114304	up
Meta_128	8-Hydroxy-2-methylquinoline	Unclassified	Unclassified	Unclassified			0.752000000000001	0.0484823866982416	0.0484823866982416	1.28638167498106	up
Meta_131	3-Hydroxymethylglutaric acid	Lipids and lipid-like molecules	Fatty Acyls	Fatty acids and conjugates	HMDB0000355	C03761	1.9	0.00368744496544155	0.00368744496544155	1.45395151296071	up
Meta_14	Cyclohexa-1,2,4,5-tetraene	Allenes	Cyclic allenes	Unclassified	HMDB0248980		0.507666666666664	0.0150369840099139	0.0150369840099139	1.38483836887278	up
Meta_143	L-Phenylalanine	Organic acids and derivatives	Carboxylic acids and derivatives	Amino acids, peptides, and analogues	HMDB0000159	C00079	0.430999999999997	0.0439457704576287	0.0439457704576287	1.25242444731125	up
Meta_149	L-2,3-Dihydrodipicolinate	Organic acids and derivatives	Carboxylic acids and derivatives	Amino acids, peptides, and analogues	HMDB0012247	C03340	0.388000000000002	0.0235274578003883	0.0235274578003883	1.32742843175161	up
Meta_150	1-Naphthyl isocyanate	Benzenoids	Naphthalenes	Unclassified	HMDB0243960		0.800000000000001	0.00264966913465489	0.00264966913465489	1.43279511228043	up
Meta_157	cis-Aconitic acid	Organic acids and derivatives	Carboxylic acids and derivatives	Tricarboxylic acids and derivatives	HMDB0000072	C00417	1.159	0.00699347502088262	0.00699347502088262	1.39587639260841	up
Meta_166	N-Formyl-L-methionine	Organic acids and derivatives	Carboxylic acids and derivatives	Amino acids, peptides, and analogues	HMDB0001015	C03145	0.813666666666666	0.0146726181746672	0.0146726181746672	1.41601313517816	up
Meta_176	Hydroxyphenyllactic acid	Phenylpropanoids and polyketides	Phenylpropanoic acids	Unclassified	HMDB0000755	C03672	1.69266666666667	2.10172050500233e-05	2.10172050500233e-05	1.47728731652957	up
Meta_178	L-Tyrosine	Organic acids and derivatives	Carboxylic acids and derivatives	Amino acids, peptides, and analogues	HMDB0000158	C00082	0.531000000000002	0.0176320060822873	0.0176320060822873	1.3683548634688	up
Meta_190	4-Hydroxy-4-(pyridin-2-yl)butan-2-one	Unclassified	Unclassified	Unclassified			0.410999999999998	0.00748609623714338	0.00748609623714338	1.37252909642567	up
Meta_192	N-Lactoylvaline	Organic acids and derivatives	Carboxylic acids and derivatives	Amino acids, peptides, and analogues	HMDB0062181		1.82733333333333	0.000314697847224579	0.000314697847224579	1.46699894884303	up
Meta_194	N8-Acetylspermidine	Organic acids and derivatives	Carboximidic acids and derivatives	Carboximidic acids	HMDB0002189	C01029	0.920333333333332	0.00680382440026812	0.00680382440026812	1.40751566493829	up
Meta_200	2-Butylbenzothiazole	Organoheterocyclic compounds	Benzothiazoles	Unclassified	HMDB0040031		1.13766666666667	0.0067229588742096	0.0067229588742096	1.41776061284818	up
Meta_216	N-Lactoylleucine	Organic acids and derivatives	Carboxylic acids and derivatives	Amino acids, peptides, and analogues	HMDB0062176		1.74	8.59522282503779e-05	8.59522282503779e-05	1.47254800637751	up
Meta_218	Theophylline	Organoheterocyclic compounds	Imidazopyrimidines	Purines and purine derivatives	HMDB0001889	C07130	1.069	0.0300734136604564	0.0300734136604564	1.25709754708658	up
Meta_223	Monosodium glutamate	Organic acids and derivatives	Carboxylic acids and derivatives	Amino acids, peptides, and analogues	HMDB0303331		0.460000000000001	0.0450309208413138	0.0450309208413138	1.28132436979426	up
Meta_229	L-Tryptophan	Organoheterocyclic compounds	Indoles and derivatives	Indolyl carboxylic acids and derivatives	HMDB0000929	C00078	0.576666666666664	0.00731586231157787	0.00731586231157787	1.39499867736101	up
Meta_231	N-Acetyl-L-phenylalanine	Organic acids and derivatives	Carboxylic acids and derivatives	Amino acids, peptides, and analogues	HMDB0000512	C03519	1.44733333333333	9.6948868026056e-05	9.6948868026056e-05	1.47452562629329	up
Meta_236	L-Kynurenine	Organic oxygen compounds	Organooxygen compounds	Carbonyl compounds	HMDB0000684	C00328	0.752000000000001	0.00247325091547257	0.00247325091547257	1.46287521646364	up
Meta_244	5-Hydroxyindoleacetic acid	Organoheterocyclic compounds	Indoles and derivatives	Indolyl carboxylic acids and derivatives	HMDB0000763	C05635	0.564666666666668	0.00830152427704419	0.00830152427704419	1.39012749014295	up
Meta_25	Toluene	Benzenoids	Benzene and substituted derivatives	Toluenes	HMDB0034168	C01455	0.484999999999999	0.0017580709064491	0.0017580709064491	1.42960539048067	up
Meta_253	Alanylglutamic acid	Organic acids and derivatives	Carboxylic acids and derivatives	Amino acids, peptides, and analogues	HMDB0028686		1.296	0.0239912272497554	0.0239912272497554	1.39842863942131	up
Meta_259	2-Hexylbenzothiazole	Organoheterocyclic compounds	Benzothiazoles	Unclassified	HMDB0040032		1.321	0.00145282167011473	0.00145282167011473	1.45244712014743	up

Meta_26	Cytosine	Organoheterocyclic compounds	Diazines	Pyrimidines and pyrimidine derivatives	HMDB0000630	C00380	0.381666666666666	0.026247176536307	0.026247176536307	1.27657649297203	up
Meta_267	N-lactoyl-Methionine	Organic acids and derivatives	Carboxylic acids and derivatives	Amino acids, peptides, and analogues	HMDB0062182		1.65333333333333	0.000816302804304709	0.000816302804304709	1.44554254467516	up
Meta_276	Dinitolmide	Benzenoids	Benzene and substituted derivatives	Toluenes	HMDB0032558		0.586666666666668	0.0161727089372015	0.0161727089372015	1.38875948434277	up
Meta_293	N1,N8-Diacetylspermidine	Organic acids and derivatives	Carboxylic acids and derivatives	Carboxylic acid derivatives	HMDB0041947		2.31566666666667	3.07236880070557e-05	3.07236880070557e-05	1.4799497863883	up
Meta_299	N-Carboxyacetyl-D-phenylalanine	Organic acids and derivatives	Carboxylic acids and derivatives	Amino acids, peptides, and analogues	HMDB0039102		0.426666666666666	0.0179950022038045	0.0179950022038045	1.43553175808538	up
Meta_307	Salsoline-1-carboxylate	Organoheterocyclic compounds	Tetrahydroisoquinolines	Unclassified	HMDB0013067		1.97733333333333	0.0010347078282976	0.0010347078282976	1.477739351	up
Meta_309	15(R)-hydroperoxy-EPE	Organoheterocyclic compounds	Diazinanes	Piperazines	HMDB0062295		2.49933333333333	0.00762541310290855	0.00762541310290855	1.44327154702409	up
Meta_320	Bisphenol B	Benzenoids	Benzene and substituted derivatives	Diphenylmethanes	HMDB0245327	C14225	0.652000000000001	0.00234775520640868	0.00234775520640868	1.42736366962794	up
Meta_339	3-Indolehydracrylic acid	Organoheterocyclic compounds	Indoles and derivatives	Indolyl carboxylic acids and derivatives	HMDB0059765		4.50933333333333	0.0231391616018998	0.0231391616018998	1.41414250954456	up
Meta_340	gamma-Glutamylvaline	Organic acids and derivatives	Carboxylic acids and derivatives	Amino acids, peptides, and analogues	HMDB0011172		1.50766666666667	0.00680099284751323	0.00680099284751323	1.45432065793419	up
Meta_343	Zederone	Lipids and lipid-like molecules	Prenol lipids	Triterpenoids	HMDB0036767		0.193333333333333	0.0412673674659488	0.0412673674659488	1.28320051841877	up
Meta_349	N-lactoyl-Tyrosine	Organic acids and derivatives	Carboxylic acids and derivatives	Amino acids, peptides, and analogues	HMDB0062177		1.904	0.00115945541651585	0.00115945541651585	1.46856062388023	up
Meta_35	gamma-Aminobutyric acid	Organic acids and derivatives	Carboxylic acids and derivatives	Amino acids, peptides, and analogues	HMDB0000112	C00334	0.458	0.0227452450647606	0.0227452450647606	1.30565101163078	up
Meta_371	Orbencarb	Unclassified	Unclassified	Unclassified		C11087	0.529	0.00158453173142039	0.00158453173142039	1.43266732683569	up
Meta_374	gamma-Glutamylleucine	Organic acids and derivatives	Carboxylic acids and derivatives	Amino acids, peptides, and analogues	HMDB0011171		1.66266666666667	0.0159928069587487	0.0159928069587487	1.41757659342303	up
Meta_375	Cyclohexanones	Organic nitrogen compounds	Organonitrogen compounds	Amines	HMDB0302411		2.63733333333333	0.00837670493922845	0.00837670493922845	1.4393532452553	up
Meta_386	Thiamine	Organoheterocyclic compounds	Diazines	Pyrimidines and pyrimidine derivatives	HMDB0000235	C00378	0.853666666666667	0.000329405863638472	0.000329405863638472	1.47315699817567	up
Meta_387	Unii-73hdp58zfk	Unclassified	Unclassified	Unclassified			0.525333333333334	0.0134071291036749	0.0134071291036749	1.34645288909638	up
Meta_39	Guaiacol	Benzenoids	Phenols	Methoxyphenols	HMDB0001398	C15572; C01502	0.489666666666668	0.00978565241805766	0.00978565241805766	1.40815674803289	up
Meta_395	Phenol glucuronide	Organic oxygen compounds	Organooxygen compounds	Carbohydrates and carbohydrate conjugates	HMDB0060014		1.524	0.00719423899583119	0.00719423899583119	1.44947221717993	up
Meta_399	3-(2-Chlorophenyl)-7-hydroxychromen-2-one	Unclassified	Unclassified	Unclassified			0.527333333333333	0.0315382834397812	0.0315382834397812	1.29152666437244	up
Meta_414	Lauryldiethanolamine	Unclassified	Unclassified	Unclassified			0.606666666666666	0.0191448662653202	0.0191448662653202	1.35945815609912	up
Meta_415	N-lactoyl-Tryptophan	Organic acids and derivatives	Carboxylic acids and derivatives	Amino acids, peptides, and analogues	HMDB0062178		1.54333333333333	0.000833496085792578	0.000833496085792578	1.44774218890459	up
Meta_418	methyl N-(3,4-dimethoxybenzoyl)glycinate	Unclassified	Unclassified	Unclassified			1.65533333333333	0.000212363557089679	0.000212363557089679	1.48140527706101	up
Meta_420	(卤)-Ribaline	Organoheterocyclic compounds	Quinolines and derivatives	Dihydrofuranoquinolines	HMDB0033345		0.506333333333332	0.0264363828431521	0.0264363828431521	1.3566983089668	up
Meta_424	Monoethylhexyl phthalic acid	Benzenoids	Benzene and substituted derivatives	Benzoic acids and derivatives	HMDB0013248	C03343	0.593666666666668	0.015502388610942	0.015502388610942	1.37579756210551	up
Meta_428	1-[(2R,3S,5R)-3,4-Dihydroxy-5-(hydroxymethyl)oxolan-2-yl]pyrimidine-2,4-dione	Nucleosides, nucleotides, and analogues	Pyrimidine nucleosides	Unclassified	HMDB0246131		0.638666666666666	0.000245924815381651	0.000245924815381651	1.46689492362062	up
Meta_450	4'-Methoxymucidin	Benzenoids	Phenol ethers	Anisoles	HMDB0030019		1.27666666666667	0.00757434502703685	0.00757434502703685	1.43753587889125	up
Meta_451	N1,N12-Diacetylspermine	Organic acids and derivatives	Carboximidic acids and derivatives	Carboximidic acids	HMDB0002172	C03413	1.233	0.00603426746941857	0.00603426746941857	1.41825467861536	up
Meta_456	N-gamma-Glutamyl-S-(1-propenyl)cysteine	Organic acids and derivatives	Carboxylic acids and derivatives	Amino acids, peptides, and analogues	HMDB0038553		1.394	0.00186890584879096	0.00186890584879096	1.4497265773735	up
Meta_459	Glutamylphenylalanine	Organic acids and derivatives	Carboxylic acids and derivatives	Amino acids, peptides, and analogues	HMDB0029156		1.721	0.013895908455662	0.013895908455662	1.43852318546099	up
Meta_464	gamma-Glutamylphenylalanine	Organic acids and derivatives	Carboxylic acids and derivatives	Amino acids, peptides, and analogues	HMDB0000594		1.835	0.0195036056201033	0.0195036056201033	1.40927499670381	up
Meta_478	Fludiazepam	Organoheterocyclic compounds	Benzodiazepines	1,4-benzodiazepines	HMDB0015513		1.152	0.00101681617527941	0.00101681617527941	1.4614032746642	up
Meta_483	4-Hydroxy-3',6-dimethoxy-5'-methylspiro[1-benzouran-2,4'-cyclohex-2-ene]-1',3-dione	Unclassified	Unclassified	Unclassified			0.419333333333334	0.0405479155093193	0.0405479155093193	1.31857480613968	up
Meta_497	Zaluzanin D	Lipids and lipid-like molecules	Prenol lipids	Terpene lactones	HMDB0302712		1.11666666666667	0.0117299028925898	0.0117299028925898	1.34924327252474	up
Meta_498	gamma-Glutamyltyrosine	Organic acids and derivatives	Carboxylic acids and derivatives	Amino acids, peptides, and analogues	HMDB0011741		1.96866666666667	0.0086465849001721	0.0086465849001721	1.45331019597781	up
Meta_5	ISOPRENE	Hydrocarbons	Unsaturated hydrocarbons	Branched unsaturated hydrocarbons	HMDB0253673	C16521	0.452999999999999	0.0355140035882137	0.0355140035882137	1.32280955617185	up
Meta_50	1-Piperidinecarboxaldehyde	Organoheterocyclic compounds	Piperidines	Unclassified	HMDB0031702		3.01533333333333	0.0329498831187476	0.0329498831187476	1.37260490635263	up
Meta_513	Phytosphingosine	Organic nitrogen compounds	Organonitrogen compounds	Amines	HMDB0004610	C12144	0.998999999999999	0.0180805898403845	0.0180805898403845	1.31967350450981	up
Meta_516	(5epsilon,9epsilon,16epsilon)-17-Hydroxykauran-19-oic acid	Unclassified	Unclassified	Unclassified			1.12833333333333	0.0122653603914948	0.0122653603914948	1.33961541059937	up
Meta_52	Levulinic acid	Organic acids and derivatives	Keto acids and derivatives	Gamma-keto acids and derivatives	HMDB0000720		1.148	0.00236092829210437	0.00236092829210437	1.42772176924353	up
Meta_523	Ssr180711	Unclassified	Unclassified	Unclassified			1.66966666666667	0.0321375912392776	0.0321375912392776	1.36124884085211	up
Meta_53	L-Proline	Organic acids and derivatives	Carboxylic acids and derivatives	Amino acids, peptides, and analogues	HMDB0000162	C00148	0.686	0.00459067656365632	0.00459067656365632	1.41046848049823	up
Meta_538	gamma-Glutamyltryptophan	Organic acids and derivatives	Carboxylic acids and derivatives	Amino acids, peptides, and analogues	HMDB0029160		2.13733333333333	0.0116608983762337	0.0116608983762337	1.44611351499547	up
Meta_55	3-Hydroxyisovaleric acid	Lipids and lipid-like molecules	Fatty Acyls	Fatty acids and conjugates	HMDB0000754		0.539333333333333	0.0146344590628042	0.0146344590628042	1.39138931337796	up
Meta_568	3-(1H-Indol-3-YL)-2-[2-(2-methylphenoxy)acetamido]propanoic acid	Unclassified	Unclassified	Unclassified			0.956	0.00598808382725583	0.00598808382725583	1.44385917863394	up
Meta_58	Indole	Organoheterocyclic compounds	Indoles and derivatives	Indoles	HMDB0000738	C00463	0.719000000000001	0.0287588831332831	0.0287588831332831	1.35055379592717	up
Meta_591	MG(i-17:0/0:0/0:0)	Lipids and lipid-like molecules	Glycerolipids	Monoradylglycerols	HMDB0072846		1.31466666666667	0.0331864009829872	0.0331864009829872	1.31711527132169	up
Meta_595	Tryptophan 2-C-mannoside	Organoheterocyclic compounds	Indoles and derivatives	Indolyl carboxylic acids and derivatives	HMDB0240296		0.487666666666668	0.0311025367664313	0.0311025367664313	1.26028302637478	up
Meta_61	L-Threonine	Organic acids and derivatives	Carboxylic acids and derivatives	Amino acids, peptides, and analogues	HMDB0000167	C00188	0.544999999999998	0.0140292847381803	0.0140292847381803	1.34630164646476	up
Meta_613	Mangostinone	Organoheterocyclic compounds	Benzopyrans	1-benzopyrans	HMDB0041292		1.06866666666667	0.0458679870523976	0.0458679870523976	1.31542469719993	up
Meta_618	2-[[1-(4-Nitrobenzoyl)pyrrolidine-2-carbonyl]amino]benzoic acid	Unclassified	Unclassified	Unclassified			1.25533333333333	0.000248357735523562	0.000248357735523562	1.47336583336511	up
Meta_622	Succinyladenosine	Nucleosides, nucleotides, and	Purine nucleosides	Unclassified	HMDB0000912		1.09933333333333	0.0209874138262652	0.0209874138262652	1.3599531338923	up
Meta_629	2-Methoxyethyl 5-hydroxy-2-methyl-1-(3-methylphenyl)benzo[g]indole-3-carboxylate	Unclassified	Unclassified	Unclassified			0.888333333333332	0.0289708557068321	0.0289708557068321	1.31051407478921	up
Meta_656	N6-Carbamoyl-L-threonyladenosine	Unclassified	Unclassified	Unclassified			0.596666666666666	0.00447869693633502	0.00447869693633502	1.42488329559286	up

Meta_662	N-(3-(dimethylamino)propyl)-2-((3,4,8,8-tetramethyl-2-oxo-2,8,9,10-tetrahydropyrano[2,3-f]chromen-5-yl)oxy)acetamide	Unclassified	Unclassified	Unclassified			0.657333333333334	0.00828778953599419	0.00828778953599419	1.41734726040853	up
Meta_666	LysoPE(14:0/0:0)	Lipids and lipid-like molecules	Glycerophospholipids	Glycerophosphoethanolamines	HMDB0011500		1.23866666666667	0.00747653654588725	0.00747653654588725	1.38738643504751	up
Meta_679	LPE O-16:1	Unclassified	Unclassified	Unclassified			0.795666666666666	0.0134754286285177	0.0134754286285177	1.35614817358877	up
Meta_688	LPE O-17:1	Unclassified	Unclassified	Unclassified			0.950333333333333	0.00399828343166121	0.00399828343166121	1.40728019103464	up
Meta_690	Peiminine	Lipids and lipid-like molecules	Steroids and steroid derivatives	Steroidal alkaloids	HMDB0256206		1.26566666666667	0.00479430282600859	0.00479430282600859	1.40167024036908	up
Meta_693	LysoPE(16:0/0:0)	Lipids and lipid-like molecules	Glycerophospholipids	Glycerophosphoethanolamines	HMDB0011503		0.780000000000001	0.0213694067995044	0.0213694067995044	1.36604553169684	up
Meta_694	LPC O-14:0	Unclassified	Unclassified	Unclassified			1.5	0.00908334705229952	0.00908334705229952	1.39470534942419	up
Meta_696	10,14-Dihydroxy-11-methoxy-3,3-dimethyl-12-(3-methyl-2-butenyl)-[1]benzopyrano[5,6-b]xanthen-13(3H)-one	Organoheterocyclic compounds	Benzopyrans	1-benzopyrans	HMDB0304630		1.13966666666667	0.000194266948510673	0.000194266948510673	1.46914814627023	up
Meta_698	(2R,3S,4S,5R,6R)-2-[[(2S,3R,4R)-3,4-dihydroxy-4-(hydroxymethyl)oxolan-2-yl]oxymethyl]-6-[4-(4-hydroxyphenyl)butan-2-yloxy]oxane-3,4,5-triol	Unclassified	Unclassified	Unclassified			0.782666666666668	0.0223767888849266	0.0223767888849266	1.39809320425812	up
Meta_699	Adenylosuccinic acid	Nucleosides, nucleotides, and	Purine nucleotides	Purine ribonucleotides	HMDB0248010		1.22966666666667	0.0159156118639259	0.0159156118639259	1.40115797102566	up
Meta_70	Ketoleucine	Organic acids and derivatives	Keto acids and derivatives	Short-chain keto acids and derivatives	HMDB0000695	C00233	1.03966666666667	0.00512576895768344	0.00512576895768344	1.41914147940531	up
Meta_701	N-[2-(3,4-dihydroxyphenyl)ethyl]docosa-4,7,10,13,16,19-hexaenamide	Unclassified	Unclassified	Unclassified			0.765000000000001	0.0341240861157753	0.0341240861157753	1.24396003207677	up
Meta_702	LysoPE(P-18:0/0:0)	Lipids and lipid-like molecules	Glycerophospholipids	Glycerophosphoethanolamines	HMDB0240598		1.00066666666667	0.0291715182624936	0.0291715182624936	1.352326367	up
Meta_703	LPE O-18:1	Unclassified	Unclassified	Unclassified			1.13266666666667	0.0147764555045086	0.0147764555045086	1.39317549719071	up
Meta_706	LPE O-18:0	Unclassified	Unclassified	Unclassified			1.101	0.0243186892835233	0.0243186892835233	1.39949144812677	up
Meta_708	LysoPC(14:0/0:0)	Lipids and lipid-like molecules	Glycerophospholipids	Glycerophosphocholines	HMDB0010379		1.008	0.043153342741678	0.043153342741678	1.28348814057275	up
Meta_709	LPC O-15:0	Unclassified	Unclassified	Unclassified			1.267	0.00767371462381875	0.00767371462381875	1.40837548052783	up
Meta_720	LysoPE(18:1(9Z)/0:0)	Lipids and lipid-like molecules	Glycerophospholipids	Glycerophosphoethanolamines	HMDB0011506		0.771000000000001	0.00510096351162537	0.00510096351162537	1.39358696023926	up
Meta_721	LysoPE(18:0/0:0)	Lipids and lipid-like molecules	Glycerophospholipids	Glycerophosphoethanolamines	HMDB0011130		0.844999999999999	0.0113563067755361	0.0113563067755361	1.43096573808367	up
Meta_722	LysoPC(P-16:0/0:0)	Lipids and lipid-like molecules	Glycerophospholipids	Glycerophosphocholines	HMDB0010407		1.289	0.00743505031999853	0.00743505031999853	1.40613186	up
Meta_724	Peldesine	Organoheterocyclic compounds	Pyrrolopyrimidines	Unclassified	HMDB0256208		1.83333333333333	0.0418843938359354	0.0418843938359354	1.2418940827204	up
Meta_728	LysoPC(15:0/0:0)	Lipids and lipid-like molecules	Glycerophospholipids	Glycerophosphocholines	HMDB0010381	C04230	0.888000000000002	0.0345143889580201	0.0345143889580201	1.26718882790017	up
Meta_729	1-O-Hexadecyl-sn-glycero-3-phosphocholine	Lipids and lipid-like molecules	Glycerophospholipids	Glycerophosphocholines	HMDB0243890		1.22566666666667	0.00692823547555827	0.00692823547555827	1.37961340746396	up
Meta_738	LPE O-20:0	Unclassified	Unclassified	Unclassified			1.11066666666667	0.000771032930419167	0.000771032930419167	1.46184746505125	up
Meta_74	N-Acetyl-L-alanine	Organic acids and derivatives	Carboxylic acids and derivatives	Amino acids, peptides, and analogues	HMDB0000766		0.613333333333333	0.0201818603480446	0.0201818603480446	1.349304101	up
Meta_740	LPC O-17:1	Unclassified	Unclassified	Unclassified			1.40633333333333	0.0104039229966568	0.0104039229966568	1.41786476515292	up
Meta_742	LysoPS(16:0/0:0)	Lipids and lipid-like molecules	Glycerophospholipids	Glycerophosphoserines	HMDB0240605		0.946333333333333	0.0050898396635074	0.0050898396635074	1.41515152097534	up
Meta_745	LPC O-17:0	Unclassified	Unclassified	Unclassified			1.54666666666667	0.0234713883692836	0.0234713883692836	1.39429284001649	up
Meta_753	LPC 17:1-SN1	Unclassified	Unclassified	Unclassified			0.938333333333334	0.00411620209395544	0.00411620209395544	1.41492574268466	up
Meta_754	LPC O-18:1	Unclassified	Unclassified	Unclassified			1.12966666666667	0.0405347783934632	0.0405347783934632	1.24561757640731	up
Meta_755	LysoPC(P-18:0/0:0)	Lipids and lipid-like molecules	Glycerophospholipids	Glycerophosphocholines	HMDB0013122	C04230	1.676	0.0131993437274457	0.0131993437274457	1.34998782085759	up
Meta_757	LysoPC(O-18:0/0:0)	Lipids and lipid-like molecules	Glycerophospholipids	Glycerophosphocholines	HMDB0011149		1.34333333333333	0.00354023607448538	0.00354023607448538	1.41044187448009	up
Meta_767	LysoPC(0:0/18:1(9Z))	Lipids and lipid-like molecules	Glycerophospholipids	Glycerophosphocholines	HMDB0061701		0.903666666666666	0.0172711168219458	0.0172711168219458	1.35835300861963	up
Meta_769	1-Stearoylglycerophosphoserine	Lipids and lipid-like molecules	Glycerophospholipids	Glycerophosphoserines	HMDB0061698		0.672333333333331	0.0322736738491013	0.0322736738491013	1.27071950672607	up
Meta_770	LPC O-16:1	Unclassified	Unclassified	Unclassified			1.271	0.0235876441968	0.0235876441968	1.37876471402561	up
Meta_771	LysoPC(0:0/18:0)	Lipids and lipid-like molecules	Glycerophospholipids	Glycerophosphocholines	HMDB0011128		0.577333333333336	0.0236475371484475	0.0236475371484475	1.28566483941103	up
Meta_776	LPC O-20:5	Unclassified	Unclassified	Unclassified			1.47766666666667	0.0250859526564981	0.0250859526564981	1.31522694222764	up
Meta_778	LPC O-20:4	Unclassified	Unclassified	Unclassified			1.21866666666667	0.0127062963974008	0.0127062963974008	1.36009560695602	up
Meta_780	LysoPE(22:1(13Z)/0:0)	Lipids and lipid-like molecules	Glycerophospholipids	Glycerophosphoethanolamines	HMDB0011521		0.627333333333334	0.0148806331566859	0.0148806331566859	1.43384469983148	up
Meta_781	LPC 19:1-SN1	Unclassified	Unclassified	Unclassified			1.25866666666666	0.0183163262660213	0.0183163262660213	1.31204792948986	up
Meta_784	LPC O-20:0	Unclassified	Unclassified	Unclassified			1.13166666666667	0.0232963936569779	0.0232963936569779	1.3415294180048	up
Meta_789	homoharringtonine	Alkaloids and derivatives	Cephalotaxus alkaloids	Unclassified	HMDB0253207		1.16566666666667	0.0119043851744684	0.0119043851744684	1.35527572747735	up
Meta_790	LPS 20:3	Unclassified	Unclassified	Unclassified			0.807	0.014858398106287	0.014858398106287	1.3832893689263	up
Meta_793	Ergosine	Alkaloids and derivatives	Ergoline and derivatives	Lysergic acids and derivatives	HMDB0251913	C09167	0.802333333333333	0.0429143375883672	0.0429143375883672	1.28025180629799	up
Meta_795	LPS 20:1	Unclassified	Unclassified	Unclassified			1.881	0.0100506498536874	0.0100506498536874	1.4146592353741	up
Meta_796	LysoPC(20:1(11Z)/0:0)	Lipids and lipid-like molecules	Glycerophospholipids	Glycerophosphocholines	HMDB0010391	C04230	1.03633333333333	0.037576537232508	0.037576537232508	1.29193924324676	up
Meta_8	1,3-Butadiene	Hydrocarbons	Unsaturated hydrocarbons	Olefins	HMDB0041792	C16450	0.472333333333335	0.000757822298437582	0.000757822298437582	1.45105301909248	up
Meta_81	Cinnamic acid	Phenylpropanoids and polyketides	Cinnamic acids and derivatives	Cinnamic acids	HMDB0000567	C10438	0.474333333333338	0.012825433515409	0.012825433515409	1.35119442232655	up
Meta_814	LPS 22:1	Unclassified	Unclassified	Unclassified			1.45933333333333	0.0493815498535278	0.0493815498535278	1.31107473296969	up
Meta_815	LysoPC(22:1(13Z)/0:0)	Lipids and lipid-like molecules	Glycerophospholipids	Glycerophosphocholines	HMDB0010399		1.229	0.0186341755015647	0.0186341755015647	1.37496733224353	up
Meta_82	2-Hydroxycaproic acid	Lipids and lipid-like molecules	Fatty Acyls	Fatty acids and conjugates	HMDB0001624		0.465	0.0178295768741821	0.0178295768741821	1.39230266524822	up
Meta_820	Cer(d18:0/16:0)	Lipids and lipid-like molecules	Sphingolipids	Ceramides	HMDB0011760		1.67733333333333	0.000800325040553896	0.000800325040553896	1.46755521589268	up
Meta_846	PC(14:0/14:0)	Lipids and lipid-like molecules	Glycerophospholipids	Glycerophosphocholines	HMDB0007866		0.569333333333336	0.0452855770408853	0.0452855770408853	1.32615594444707	up
Meta_85	2-Methylindole	Organoheterocyclic compounds	Indoles and derivatives	Indoles	HMDB0245234		0.761333333333337	0.0175253466657193	0.0175253466657193	1.42128682643807	up
Meta_859	PC 28:0	Unclassified	Unclassified	Unclassified			0.468333333333334	0.030592253892936	0.030592253892936	1.30954977961015	up
Meta_88	Malic acid	Organic acids and derivatives	Hydroxy acids and derivatives	Beta hydroxy acids and derivatives	HMDB0000156	C00711	0.580333333333332	0.012062882524552	0.012062882524552	1.35292741446605	up
Meta_883	SM 8:0;2O/24:0	Unclassified	Unclassified	Unclassified			0.874666666666666	0.00772166867445639	0.00772166867445639	1.36866050546955	up
Meta_887	PC 30:1	Unclassified	Unclassified	Unclassified			0.658666666666665	0.00729420111453334	0.00729420111453334	1.38140580502199	up
Meta_89	3-Methyl sulfolene	Organoheterocyclic compounds	Dihydrothiophenes	Unclassified	HMDB0059667		0.673333333333336	0.0109473058750157	0.0109473058750157	1.38783634618289	up
Meta_897	PC O-33:1	Unclassified	Unclassified	Unclassified			0.414666666666666	0.00904498784103963	0.00904498784103963	1.36411804701193	up

Meta_90	2-Deoxy-D-ribose	Organic oxygen compounds	Organooxygen compounds	Carbonyl compounds	HMDB0245099	C01801	1.40833333333333	0.00124494374783441	0.00124494374783441	1.47215383048803	up
Meta_921	CerP(d18:1/22:0)	Lipids and lipid-like molecules	Sphingolipids	Phosphosphingolipids	HMDB0010703		0.956333333333333	0.00655637395858641	0.00655637395858641	1.4023355450796	up
Meta_923	PC(14:0/16:1(9Z))	Lipids and lipid-like molecules	Glycerophospholipids	Glycerophosphocholines	HMDB0007870		0.376666666666665	0.00292142279366223	0.00292142279366223	1.44841276419308	up
Meta_925	SM 8:0;2O/26:0	Unclassified	Unclassified	Unclassified			0.369666666666667	0.0263116467435538	0.0263116467435538	1.35964932497852	up
Meta_942	PC 9:0_26:6	Unclassified	Unclassified	Unclassified			0.224666666666666	0.0359788419678797	0.0359788419678797	1.26509116336309	up
Meta_95	p-Octopamine	Benzenoids	Phenols	1-hydroxy-2-unsubstituted benzenoids	HMDB0004825	C04227	0.657333333333334	0.00292500758748926	0.00292500758748926	1.43367741283668	up
Meta_97	1-Methylnicotinamide	Organoheterocyclic compounds	Pyridines and derivatives	Pyridinecarboxylic acids and derivatives	HMDB0000699	C02918	1.311	0.00301782150279785	0.00301782150279785	1.43462298935609	up
Meta_990	PC O-16:1_20:4	Unclassified	Unclassified	Unclassified			0.353666666666667	0.0107898650936352	0.0107898650936352	1.35237908272729	up

Table S3. The potential bioavailable targets of Clofazimine predicted by SwissTargetPrediction and TargetNet

Database	Targets	Number
SwissTargetPrediction	PPIA CNR1 PGR PFKFB3 MAPK14 CRHR1 MET GCGR TSPO GPR39 CNR2 GRK2 EIF2AK2 HTR2B HTR2A HTR2C HCRTR2 HCRTR1 VCP CHRM4 PDE5A CHRM5 CHRM2 CHRM1 CHRM3 HSD17B3 IMPDH2 P2RX3 KCNJ5 KCNJ6 SSTR2 SSTR4 KCNH2 CCKBR GCK FFAR1 MDM4 MDM2 SSTR3 GRM5 DGAT1 CXCR3 PTGS2 OPRM1 OPRD1 OPRK1 GSK3B ALK CCNE1 NAAA TGFBR1 ADORA3 SLC5A2 MAP3K8 SLC5A1 NR3C1 PDE6D ASAH1 PTAFR ADRB2 SCN10A ERBB2 EGFR P2RY1 TACR1 PLA2G7 PDE10A SMO IDH1 FNTA FNTB MAPK11 TLR9 TRPM8 GPR55 NR3C2 PSEN2 ALOX5 ACHE MAPK8 MAPK10 NR1H3 INCENP NR1H2 MAPK9 AURKA ABL1 AVPR1A OXTR SRC KDR HSD11B1 JAK3 JAK2 GRIA2 TRPV1 FLT1 ADK ALOX5AP KIT PDGFRA	100
TargetNet	CRHR1 CYP11B2 PGR LRRK2 PRKCQ RELA CLK1 EGFR Grm1 TGFBR1 NOS3 NOS1 MAOA MAPK11 EGFR KDR NOS2 JAK2 SRC MAP3K8 AHR MAPK10 P2rx7 HTR5A DYRK1A Grm5 CYP11B1 IMPDH ADK Grm2 Mapk14 IRAK4 GRM4 P2RX7 MAPK14 BRAF Pdgfrb PDE7A PDGFRB MAOB TEK CYP17A1 GSK3B CASP9 LCK Dyrk1a PIM1 ABL1 XDH APOBEC3G RPS6KB1 CSNK2A1 KIF11 RET PIM3 RPS6KA3 AKT1 LYN PDE10A ROCK1 CDK1 NR3C2 ATP4A AR PIM2 EPHB4 ADRA2A Cnr1 NR2E3 PDE4A CNR1 FGFR1 PDGFRA PTGES CLK4 Hrh1 ABCG2 PTK2 DRD1 ADRA2B PDE2A CCNA1 BCL2A1 ADRA2C TLR9 Crhr1 GPBAR1 GALR3 PRCP ACHE ADORA1 SLC6A3 ERBB2 DRD5 CNR2 HTR2C Adra2c KCNA5 BTK CYP1A2 HSD11B1 AXL PDE4D CCNE2 CYP19A1 MET PDE4B SLC6A2 GRM2 APP ATP4A HRH4 RORA SIGMAR1 SLC6A4 PLK1 CDK2 PDPK1 MAPK9 NPY5R FLT1 Tspo DHODH NTRK1 CSF1R FLT3 CDC7 PTAFR CDK4 MAPK8 HTR2A Taar1 TERT GPR35 MAPKAPK2 CDK7 HCK Chrna7 Trpv1 TNK2 APOBEC3A RIPK2 Hsd11b1 FYN Drd4 PAK4 FNTB HSD11B2 Nos2 CDK9 Htr2c MAP2K1 CYP3A4 ALK HTR1E IGF1R CHRFAM7A RAC1 TYK2 RAF1 Prcp CHRNA7 DUSP3 CACNA1H PIK3CD FNTB Slc6a3 JAK1 ACHE TAAR1 PDE3A HNF4A PIK3CB PLAU CHRNA4 FLT4 PRKCD AURKA MDM2 ache Chrna4 Slc6a4 SCN5A ICAM1 KCNH2 SMO CASR SYK MIF GSK3A AKT3 Htr2a ADORA3 TMIGD3 NPBWR1 S1PR3 CDK5 PIK3CA GRIN2B CHRM4 MPL Rac1 HTR3A HCRTR1 OPRL1 GCGR DRD1 Ptgs2 ache PLIN5 Maoa CYP2D6 Drd1 JAK3 F2R Htr6 PTPN7 CDC42 ALPL DHFR PTGS1 AKT2 IKBKB ADORA1 ADORA2A Maob SLC9A1 ITK PLA2G1B PLIN1 CCR4 PIK3CG Nos1 PGGT1B CACNA1B CHEK2 MGLL PTGS1 HTR7 Ache ROCK2	273
Overlapping targets	CNR1 PGR MAPK14 CRHR1 MET GCGR CNR2 HTR2A HTR2C HCRTR1 CHRM4 KCNH2 MDM2 GSK3B ALK TGFBR1 ADORA3 MAP3K8 PTAFR ERBB2 EGFR PDE10A SMO MAPK11 TLR9 NR3C2 ACHE MAPK8 MAPK10 MAPK9 AURKA ABL1 SRC KDR HSD11B1 JAK3 JAK2 FLT1 ADK PDGFRA	40

Table S4. The metabolites in metabolomics analysis (CFZ v.s. Ctrl)

Alignment ID	Metabolite name	Category1	Category2	HMDB ID	log2fc	p.value	fdr	VIP	regulation
Meta_1	Urea	Amino Acid	Urea cycle; Arginine and Proline Metabolism	HMDB0000294	-1.759	0.00451916740133313	0.00451916740133313	1.26586352141864	down
Meta_100	Ectoine	Xenobiotics	Natural Product/Food/Plant	METPA0797	-2.116	0.00437234106318107	0.00437234106318107	1.25583427972071	down
Meta_106	4-Guanidinobutanoate	Amino Acid	Urea cycle; Arginine and Proline Metabolism	HMDB0003464	-1.490666667	0.00156927377946948	0.00156927377946948	1.26492296527263	down
Meta_124	Xanthine	Nucleotide	Purine Metabolism, (Hypo)Xanthine/Inosine containing	HMDB0000292	-0.814	0.00291897741069794	0.00291897741069794	1.24687037626961	down
Meta_133	Homostachydrine	Xenobiotics	Natural Product/Food/Plant	HMDB0004827	-0.922666667	0.011117998646262	0.011117998646262	1.22253067929591	down
Meta_14	L-Alanine	Amino Acid	Alanine, Aspartate and Glutamate Metabolism	HMDB0000161	-1.214666667	0.00251763135799214	0.00251763135799214	1.25003895809646	down
Meta_141	Ala-Ala	Peptide	Dipeptide	HMDB0003459	-2.338666667	0.0454178569210274	0.0454178569210274	1.13446520833075	down
Meta_152	Phenyllactic Acid	Amino Acid	Phenylalanine Metabolism	HMDB0000779	-0.909333333	0.0326290007291713	0.0326290007291713	1.13521679349717	down
Meta_154	2-Mercaptobenzothiazole	Xenobiotics	Natural Product/Food/Plant	HMDB0030524	-0.694	0.044229988271091	0.044229988271091	1.05809832428213	down
Meta_159	Phosphoenolpyruvate	Carbohydrate	Glycolysis, Gluconeogenesis, and Pyruvate Metabolism	HMDB0000263	-2.057333333	0.0129110823137227	0.0129110823137227	1.24813042767371	down
Meta_162	Pyridoxal	Cofactors and Vitamins	Vitamin B6 Metabolism	HMDB0001545	-0.990666667	0.00509314606245588	0.00509314606245588	1.20898963457747	down
Meta_174	Gly-Val	Peptide	Dipeptide	HMDB0028854	-2.408666667	0.00931126644847571	0.00931126644847571	1.25843560193815	down
Meta_175	Theanine	Xenobiotics	Natural Product/Food/Plant	HMDB0034365	-2.782666667	0.00207224766523337	0.00207224766523337	1.26289707234157	down
Meta_186	Mannitol/Sorbitol	Carbohydrate	Polysaccharides and Oligosaccharides	HMDB0000247	-1.244666667	0.000399317182185706	0.000399317182185706	1.2675189652159	down
Meta_193	3-Phosphoglyceric Acid	Carbohydrate	Glycolysis, Gluconeogenesis, and Pyruvate Metabolism	HMDB0000807	-1.77	0.000697023596488636	0.000697023596488636	1.27066737061304	down
Meta_196	Azelaic acid	Lipid	Medium Chain Fatty Acid	HMDB0000784	-0.679333333	0.0340637804444784	0.0340637804444784	1.14472921496767	down
Meta_197	Azelate (C9-DC)	Lipid	Fatty Acid, Dicarboxylate	HMDB0000784	-0.514	0.0144399809301589	0.0144399809301589	1.16485630499685	down
Meta_199	10-Hydroxydecanoic acid	Lipid	Fatty Acid, Monohydroxy	HMDB0094656	-0.825	0.0187027747489113	0.0187027747489113	1.168283889	down
Meta_20	Cyclohexanamine	Xenobiotics	Chemical	HMDB0031404	-1.056333333	0.00461144757667569	0.00461144757667569	1.21762164760369	down
Meta_204	Ala-Val	Peptide	Dipeptide	HMDB0028700	-3.145	0.00281573804119961	0.00281573804119961	1.27650011353902	down
Meta_205	Gly-Leu	Peptide	Dipeptide	HMDB0000759	-2.307666667	0.000687316622152624	0.000687316622152624	1.27637921359119	down
Meta_206	Gly-Ile	Peptide	Dipeptide	HMDB0028844	-2.648	0.000255566302469098	0.000255566302469098	1.27366154941471	down
Meta_208	N6,N6,N6-Trimethyllysine	Amino Acid	Lysine Metabolism	HMDB0001325	-1.005666667	0.0133602798302995	0.0133602798302995	1.22667843231814	down
Meta_214	Phenylacetylglycine	Lipid	Fatty Acid Metabolism (Acyl glycines)	HMDB0000821	-1.296333333	0.00914778968594565	0.00914778968594565	1.21894655837682	down
Meta_216	Quinic Acid	Amino Acid	Shikimic acid pathway	HMDB0003072	-1.819	0.0250512490610962	0.0250512490610962	1.21475602290941	down
Meta_217	3-Hydroxycotinine	Xenobiotics	Tobaco Metabolite	HMDB0001390	-0.555	0.0389604012142518	0.0389604012142518	1.13665336602759	down
Meta_220	4-Aminohippuric acid	Xenobiotics	Benzoate Metabolism	HMDB0001867	-1.494666667	0.00110344899115587	0.00110344899115587	1.24897554596304	down
Meta_229	Ala-Ile	Peptide	Dipeptide	HMDB0028690	-3.070333333	0.00436892869506908	0.00436892869506908	1.27115786149137	down
Meta_230	Leu-Ala	Peptide	Dipeptide	HMDB0028922	-3.419	0.00634775003515667	0.00634775003515667	1.26325177280511	down
Meta_231	Ala-Leu	Peptide	Dipeptide	HMDB0028691	-3.177333333	0.00300472827294661	0.00300472827294661	1.27219811810613	down
Meta_232	Ile-Ala	Peptide	Dipeptide	HMDB0028900	-2.482333333	0.0189176355357957	0.0189176355357957	1.23320293580775	down
Meta_239	Ser-Val	Peptide	Dipeptide	HMDB0029052	-3.641	0.00299011893547245	0.00299011893547245	1.27439229667857	down
Meta_248	Creatine phosphate	Amino Acid	Urea cycle; Arginine and Proline Metabolism	HMDB0001511	-1.197666667	0.00329547274378926	0.00329547274378926	1.22817584274281	down
Meta_25	3-Hydroxyisobutyrate	Amino Acid	Leucine, Isoleucine and Valine Metabolism	HMDB0000336	-1.285666667	0.0246772695692064	0.0246772695692064	1.18387357121802	down
Meta_252	Undecanedioate (C11-DC)	Lipid	Fatty Acid, Dicarboxylate	HMDB0000888	-0.594	0.0145835244553313	0.0145835244553313	1.18625820998866	down
Meta_254	OMEGA-HYDROXYDODECANOIC ACID	Lipid	Fatty Acid, Monohydroxy	HMDB0002059	-2.159333333	0.0203978943234542	0.0203978943234542	1.18970666821537	down
Meta_256	Malonylglycine	Lipid	Fatty Acid Metabolism (Acyl glycines)	HMDB0240730	-1.048	0.00138431574079194	0.00138431574079194	1.24600216649403	down
Meta_257	Val-Val	Peptide	Dipeptide	HMDB0029140	-2.964333333	0.000754896663919676	0.000754896663919676	1.28310188958818	down
Meta_258	Propionylcarnitine	Lipid	Fatty Acid Metabolism (Acyl Carnitine)	HMDB0000824	-3.686	0.000246590815520878	0.000246590815520878	1.2735479385497	down
Meta_26	Cadaverine	Amino Acid	Lysine Metabolism	HMDB0002322	-1.573666667	0.016993327483895	0.016993327483895	1.23454013279208	down
Meta_261	Ser-Ile	Peptide	Dipeptide	HMDB0029042	-3.047333333	0.0151302545727818	0.0151302545727818	1.24221665958573	down
Meta_262	Ser-Leu	Peptide	Dipeptide	HMDB0029043	-2.977333333	0.00310157232458008	0.00310157232458008	1.27470771331293	down
Meta_266	Phe-Gly	Peptide	Dipeptide	HMDB0028995	-3.866666667	0.00505128357411276	0.00505128357411276	1.26695766082071	down
Meta_267	Gly-Phe	Peptide	Dipeptide	HMDB0028848	-3.108	0.00757172886420005	0.00757172886420005	1.26391272917382	down
Meta_277	D-Ribose 5-Phosphate	Carbohydrate	Pentose phosphate pathway	HMDB0001548	-0.685333333	0.0314275316739218	0.0314275316739218	1.12798701405198	down
Meta_281	Pro-Ile	Peptide	Dipeptide	HMDB0304810	-1.849333333	0.0208544103779848	0.0208544103779848	1.16762865738711	down
Meta_282	Leu-Val	Peptide	Dipeptide	HMDB0028942	-2.947	0.00123446943541814	0.00123446943541814	1.27517983296978	down
Meta_283	Ile-Val	Peptide	Dipeptide	HMDB0028920	-3.115333333	0.00458460542655634	0.00458460542655634	1.26629009315501	down
Meta_284	Val-Leu	Peptide	Dipeptide	HMDB0029131	-3.14	0.00864404697065837	0.00864404697065837	1.25573071898571	down
Meta_285	Isobutyrylcarnitine (C4)	Amino Acid	Leucine, Isoleucine and Valine Metabolism	HMDB0000736	-1.759666667	0.00107301222544344	0.00107301222544344	1.25110739172948	down
Meta_287	Thr-Leu	Peptide	Dipeptide	HMDB0029065	-2.788	0.000155176526301327	0.000155176526301327	1.27645377608436	down
Meta_29	Glyceric Acid	Carbohydrate	Pentose phosphate pathway	HMDB0000139	-4.239333333	0.0027966791039399	0.0027966791039399	1.27451435311513	down
Meta_297	Uridine	Nucleotide	Pyrimidine Metabolism, Uracil containing	HMDB0000296	-1.442666667	0.00487827237753753	0.00487827237753753	1.21798889234582	down
Meta_298	Lumichrome	Xenobiotics	Natural Product/Food/Plant	HMDB0254199	-1.448333333	0.0217940039761472	0.0217940039761472	1.2232407656784	down
Meta_301	2-Hydroxymyristate	Lipid	Fatty Acid, Monohydroxy	HMDB0002261	-0.931	0.0172484957583969	0.0172484957583969	1.16308267528892	down
Meta_304	Leu-Leu	Peptide	Dipeptide	HMDB0028933	-2.873666667	0.00638785252243909	0.00638785252243909	1.25399022791391	down
Meta_305	Ile-Leu	Peptide	Dipeptide	HMDB0028911	-2.514	0.00388374242459977	0.00388374242459977	1.24052548017193	down
Meta_306	Ile-Ile	Peptide	Dipeptide	HMDB0028910	-3.174	0.00877364025308906	0.00877364025308906	1.25042084192223	down
Meta_309	Ile-Asn	Peptide	Dipeptide	HMDB0028902	-2.762	0.0033286232670424	0.0033286232670424	1.27000829748157	down
Meta_310	Leu-Gly-Gly	Peptide	Small peptide		-1.360666667	0.0125163723292953	0.0125163723292953	1.17475996229907	down
Meta_312	Isovalerylcarnitine (C5)	Amino Acid	Leucine, Isoleucine and Valine Metabolism	HMDB0000688	-0.446333333	0.0281398941101919	0.0281398941101919	1.15953102500721	down

Meta_313	2-Methylbutyrylcarnitine (C5)	Amino Acid	Leucine, Isoleucine and Valine Metabolism	HMDB0000378	-0.975333333	0.0105722684842631	0.0105722684842631	1.18645272122207	down
Meta_319	Met-Val	Peptide	Dipeptide	HMDB0028986	-3.359	7.09989048084934e-06	7.09989048084934e-06	1.28402265606126	down
Meta_320	Val-Met	Peptide	Dipeptide	HMDB0259742	-2.956333333	0.00782172586021225	0.00782172586021225	1.259072609	down
Meta_323	Ser-Phe	Peptide	Dipeptide	HMDB0029046	-3.657333333	0.019666315448814	0.019666315448814	1.23694531685453	down
Meta_327	N-Acetylglucosamine	Carbohydrate	Amino sugar and Nucleotide sugar Metabolism	HMDB0000215	-1.289666667	0.00180477952727052	0.00180477952727052	1.25269156556744	down
Meta_336	Glucose 6-Phosphate/Fructose 6-Phosphate	Carbohydrate	Glycolysis, Gluconeogenesis, and Pyruvate Metabolism	HMDB0001401	-0.895666667	0.00769452648355298	0.00769452648355298	1.20655164951806	down
Meta_338	2'-O-Methyluridine	Nucleotide	Pyrimidine Metabolism, Uracil containing	HMDB0240328	-1.720333333	0.0159899697512928	0.0159899697512928	1.1482005855972	down
Meta_34	S-1-Pyrroline-5-Carboxylate	Amino Acid	Alanine, Aspartate and Glutamate Metabolism	HMDB0001301	-1.699333333	0.0339475796648097	0.0339475796648097	1.14722653550613	down
Meta_340	Lys-Ile	Peptide	Dipeptide	HMDB0028954	-0.620666667	0.00488952365031993	0.00488952365031993	1.25282693604876	down
Meta_341	Ile-Lys	Peptide	Dipeptide	HMDB0028912	-3.154666667	0.017757446113148	0.017757446113148	1.23508890950076	down
Meta_342	Lys-Leu	Peptide	Dipeptide	HMDB0028955	-2.557666667	0.0160790682823563	0.0160790682823563	1.20912684315084	down
Meta_344	Leu-Glu	Peptide	Dipeptide	HMDB0028928	-3.771333333	0.0455622385720205	0.0455622385720205	1.17158182064576	down
Meta_345	Ile-Glu	Peptide	Dipeptide	HMDB0028906	-1.938333333	0.00231764032199027	0.00231764032199027	1.2633865625204	down
Meta_346	Glu-Leu	Peptide	Dipeptide	HMDB0028823	-1.822	0.0106937759669834	0.0106937759669834	1.25023518074184	down
Meta_353	Ile-Met	Peptide	Dipeptide	HMDB0028913	-3.596333333	0.0245997367029181	0.0245997367029181	1.22147124904688	down
Meta_354	Met-Leu	Peptide	Dipeptide	HMDB0028977	-3.245333333	0.0468683843191016	0.0468683843191016	1.141313212	down
Meta_357	Phe-Val	Peptide	Dipeptide	HMDB0029008	-3.633	0.0117681109169115	0.0117681109169115	1.24205155118431	down
Meta_358	Val-Phe	Peptide	Dipeptide	HMDB0029134	-3.269333333	1.88871675050268e-05	1.88871675050268e-05	1.28074044377061	down
Meta_360	Thr-Phe	Peptide	Dipeptide	HMDB0029068	-3.544	0.0462711616767289	0.0462711616767289	1.17003563534626	down
Meta_366	Ser-Tyr	Peptide	Dipeptide	HMDB0029051	-3.982	0.00598103507688072	0.00598103507688072	1.25187251570808	down
Meta_367	His-Leu	Peptide	Dipeptide	HMDB0028889	-3.606666667	0.0171925319294534	0.0171925319294534	1.23987366832143	down
Meta_370	2-Hydroxypalmitate	Lipid	Fatty Acid, Monohydroxy	HMDB0031057	-0.633666667	0.0431122487073346	0.0431122487073346	1.11094892306079	down
Meta_372	6-Phosphogluconic Acid	Carbohydrate	Pentose phosphate pathway	HMDB0001316	-1.049333333	0.0379225675133818	0.0379225675133818	1.07915080109912	down
Meta_374	Gamma-Glutamylglutamate	Peptide	Gamma-glutamyl Amino Acid	HMDB0011737	-1.566	0.00935691507229562	0.00935691507229562	1.24593144230882	down
Meta_378	Phe-Ile	Peptide	Dipeptide	HMDB0028998	-2.992333333	0.00913703347003124	0.00913703347003124	1.23754250589843	down
Meta_379	Phe-Leu	Peptide	Dipeptide	HMDB0253025	-3.046	0.0190049089693291	0.0190049089693291	1.22679884071363	down
Meta_384	N1-Methyladenosine	Nucleotide	Purine Metabolism, Adenine containing	HMDB0003331	-0.98	0.0251478793506411	0.0251478793506411	1.20432480711947	down
Meta_388	Myristoylglycine	Lipid	Fatty Acid Metabolism (Acyl glycines)	HMDB0013250	-1.813	0.00362232250481551	0.00362232250481551	1.24855921589348	down
Meta_393	Ile-Arg	Peptide	Dipeptide	HMDB0028901	-1.33	0.012873010527604	0.012873010527604	1.21674440699523	down
Meta_394	Lauryl diethanolamide	Lipid	Endocannabinoid (N-acylethanolamine)	HMDB0032358	-1.135333333	0.000591532998664308	0.000591532998664308	1.26857125782033	down
Meta_401	Phe-Glu	Peptide	Dipeptide	HMDB0028994	-3.161666667	0.0100584602177233	0.0100584602177233	1.25605101540717	down
Meta_403	Ile-Tyr	Peptide	Dipeptide	HMDB0028919	-4.053333333	0.00225682217790413	0.00225682217790413	1.27255199786465	down
Meta_407	N1-Methylguanosine	Nucleotide	Purine Metabolism, Guanine containing	HMDB0001563	-0.434333333	0.00417479157098915	0.00417479157098915	1.24181228455505	down
Meta_409	2-Hydroxyoleate	Lipid	Fatty Acid, Monohydroxy		-1.547666667	0.0360162203962962	0.0360162203962962	1.19771452082093	down
Meta_41	L-Proline	Amino Acid	Urea cycle; Arginine and Proline Metabolism	HMDB0000162	-1.224	0.00446452333610102	0.00446452333610102	1.26710636189064	down
Meta_431	N-Acetylneuraminic Acid	Carbohydrate	Amino sugar and Nucleotide sugar Metabolism	HMDB0000230	-1.45	0.0230059210100834	0.0230059210100834	1.2039508369526	down
Meta_438	N2,N2-Dimethylguanosine	Nucleotide	Purine Metabolism, Guanine containing	HMDB0004824	-3.156333333	0.000651926087025154	0.000651926087025154	1.28152770103031	down
Meta_440	Phe-Phe	Peptide	Dipeptide	HMDB0013302	-3.808666667	0.00441066740492984	0.00441066740492984	1.266130622	down
Meta_443	Ile-Trp	Peptide	Dipeptide	HMDB0028918	-1.797333333	0.0253000536841914	0.0253000536841914	1.19735202180749	down
Meta_45	2-Hydroxy-2-methylbutyric acid	Lipid	Fatty Acid, Monohydroxy	HMDB0001987	-2.508	0.0128974524863478	0.0128974524863478	1.18970687837761	down
Meta_454	Phe-Tyr	Peptide	Dipeptide	HMDB0029007	-3.074666667	0.00530397879493114	0.00530397879493114	1.25733468907488	down
Meta_459	b-Nicotinamide Mononucleotide	Cofactors and Vitamins	Nicotinate and Nicotinamide Metabolism	HMDB0000229	-1.154333333	0.0258738775665856	0.0258738775665856	1.11624291973041	down
Meta_47	3-Hydroxyisovaleric Acid	Amino Acid	Leucine, Isoleucine and Valine Metabolism	HMDB0000754	-2.514	0.00018292719185504	0.00018292719185504	1.27009224250419	down
Meta_470	Tyr-Tyr	Peptide	Dipeptide	HMDB0029117	-3.758333333	0.0111072902295046	0.0111072902295046	1.25503938368547	down
Meta_483	S-Lactoylglutathione	Carbohydrate	Glycolysis, Gluconeogenesis, and Pyruvate Metabolism	HMDB0001066	-2.736666667	0.0131321290364919	0.0131321290364919	1.18870813879495	down
Meta_553	CTP	Nucleotide	Pyrimidine Metabolism, Cytidine containing	HMDB0000082	-2.784666667	0.00687296834122961	0.00687296834122961	1.24284172373999	down
Meta_556	UTP	Nucleotide	Pyrimidine Metabolism, Uracil containing	HMDB0000285	-1.683	0.00747767615061716	0.00747767615061716	1.21338019375794	down
Meta_567	dGTP	Nucleotide	Purine Metabolism, Guanine containing	HMDB0001440	-1.829666667	0.0151441403744779	0.0151441403744779	1.18370352995485	down
Meta_607	UDP-N-Acetyl-Glucosamine	Carbohydrate	Amino sugar and Nucleotide sugar Metabolism	HMDB0000290	-0.725666667	0.0204376584552455	0.0204376584552455	1.15784462376602	down
Meta_616	PE 32:0|PE 16:0_16:0	Lipid	Glycerophospholipid (phosphatidylethanolamine)	HMDB0008923	-3.567333333	0.0269056798257592	0.0269056798257592	1.14804029965279	down
Meta_620	PC 30:0|PC 14:0_16:0	Lipid	Glycerophospholipid (phosphatidylcholine)	HMDB0007869	-0.464333333	0.0206874317855171	0.0206874317855171	1.15925563991515	down
Meta_623	PE 34:2|PE 16:0_18:2	Lipid	Glycerophospholipid (phosphatidylethanolamine)	HMDB0008928	-1.091	0.0025132642435758	0.0025132642435758	1.2335836428545	down
Meta_625	PE 34:1|PE 16:0_18:1	Lipid	Glycerophospholipid (phosphatidylethanolamine)	HMDB0008927	-1.094333333	0.00863489449258123	0.00863489449258123	1.22090881772359	down
Meta_626	PC 31:0	Lipid	Glycerophospholipid (phosphatidylcholine)	HMDB0007935	-0.523333333	0.0461020945415856	0.0461020945415856	1.05880480807638	down
Meta_632	PC 32:0|PC 16:0_16:0	Lipid	Glycerophospholipid (phosphatidylcholine)	HMDB0000564	-1.437666667	0.00342153553442	0.00342153553442	1.24175516405484	down
Meta_636	PE 36:3|PE 18:1_18:2	Lipid	Glycerophospholipid (phosphatidylethanolamine)	HMDB0009060	-0.393	0.0164762176342018	0.0164762176342018	1.23700183018636	down
Meta_669	PE 40:6|PE 18:0_22:6	Lipid	Glycerophospholipid (phosphatidylethanolamine)	HMDB0009012	-2.074666667	0.000776117858839003	0.000776117858839003	1.25641270796515	down
Meta_85	Deoxyribose	Carbohydrate	Pentose phosphate pathway	HMDB0003224	-1.065333333	0.0219341479857265	0.0219341479857265	1.13755616247443	down
Meta_90	Erythronic Acid	Carbohydrate	Amino sugar and Nucleotide sugar Metabolism	HMDB0000613	-1.515333333	0.00107377252360636	0.00107377252360636	1.2522747749169	down
Meta_94	Hypoxanthine	Nucleotide	Purine Metabolism, (Hypo)Xanthine/Inosine containing	HMDB0000157	-1.689	0.00953502548329998	0.00953502548329998	1.24989568641341	down
Meta_96	Trigonelline (N'-Methylnicotinate)	Cofactors and Vitamins	Nicotinate and Nicotinamide Metabolism	HMDB0000875	-0.690666667	0.0397137068494318	0.0397137068494318	1.17316476282754	down
Meta_99	Methylimidazoleacetic acid	Amino Acid	Histidine Metabolism	HMDB0002820	-1.385333333	0.00743155265573813	0.00743155265573813	1.25335043146638	down
Meta_10	Pyruvic Acid	Carbohydrate	Glycolysis, Gluconeogenesis, and Pyruvate Metabolism	HMDB0000243	0.162000000000001	0.645708966429026	0.645708966429026	0.308719073041911	unchanged
Meta_101	Octanoic Acid (C8:0)	Lipid	Medium Chain Fatty Acid	HMDB0000482	-0.066666667	0.600309645546748	0.600309645546748	0.352055186779172	unchanged

Meta_102	2-Naphthylamine	Xenobiotics	Natural Product/Food/Plant	HMDB0243964	0.620333333333335	0.0787753176807693	0.0787753176807693	1.0325977391391	unchanged
Meta_103	Stachydrine	Xenobiotics	Natural Product/Food/Plant	HMDB0004827	-0.691333333	0.0857040559970515	0.0857040559970515	0.959845307470779	unchanged
Meta_105	Adipic acid	Xenobiotics	Degradation of aromatic compounds	HMDB0000448	-0.047	0.825780757166131	0.825780757166131	0.146286047329072	unchanged
Meta_107	N-Methylisoleucine	Amino Acid	Leucine, Isoleucine and Valine Metabolism	HMDB0341355	-0.806333333	0.166188651929983	0.166188651929983	0.869802024826173	unchanged
Meta_108	N-Methylleucine	Amino Acid	Leucine, Isoleucine and Valine Metabolism		-0.813333333	0.209667495578467	0.209667495578467	0.809368585679488	unchanged
Meta_11	Sarcosine	Amino Acid	Glycine, Serine and Threonine Metabolism	HMDB0000271	-0.420333333	0.151940550911307	0.151940550911307	0.86240988157736	unchanged
Meta_110	Spermidine	Amino Acid	Polyamine Metabolism	HMDB0001257	0.606333333333335	0.350534168377024	0.350534168377024	0.622994463000899	unchanged
Meta_112	P-Coumaraldehyde	Xenobiotics	Natural Product/Food/Plant	HMDB0037254	0.305666666666665	0.60640845998141	0.60640845998141	0.35819976673085	unchanged
Meta_115	L-Glutamic Acid	Amino Acid	Alanine, Aspartate and Glutamate Metabolism	HMDB0000148	0.263000000000002	0.0947458584344778	0.0947458584344778	0.951572202422231	unchanged
Meta_116	Tartaric Acid	Carbohydrate	Glyoxylate and dicarboxylate metabolism	HMDB0000956	-1.59	0.383312613250502	0.383312613250502	0.60696029662913	unchanged
Meta_119	3-Phenylpropionic Acid	Xenobiotics	Benzoate Metabolism	HMDB0000764	-0.578333333	0.638732781386696	0.638732781386696	0.310783682044504	unchanged
Meta_121	2-Hydroxyphenylacetic Acid	Amino Acid	Phenylalanine Metabolism	HMDB0000669	-0.360666667	0.39824634315151	0.39824634315151	0.573813745950598	unchanged
Meta_123	Guanine	Nucleotide	Purine Metabolism, Guanine containing	HMDB0000132	0.587666666666667	0.0757120808943735	0.0757120808943735	1.00564682537729	unchanged
Meta_126	N-Acetylhistamine	Amino Acid	Histidine Metabolism	HMDB0013253	-0.090666667	0.797366215048342	0.797366215048342	0.175174826647085	unchanged
Meta_128	4,4'-Bipyridine	Xenobiotics	Chemical	HMDB0246599	0.230666666666664	0.490137978908686	0.490137978908686	0.471990210499399	unchanged
Meta_129	2-ketocaprylate	Amino Acid	Leucine, Isoleucine and Valine Metabolism	HMDB0013211	-0.220666667	0.684062331325117	0.684062331325117	0.286436630241879	unchanged
Meta_13	Putrescine	Amino Acid	Polyamine Metabolism	HMDB0001414	0.395333333333333	0.148348196891999	0.148348196891999	0.867561065888168	unchanged
Meta_130	Nonanoic Acid (C9:0)	Lipid	Medium Chain Fatty Acid	HMDB0000847	-0.036666667	0.751494561427748	0.751494561427748	0.218599146795622	unchanged
Meta_131	N-Acetylproline	Amino Acid	Urea cycle; Arginine and Proline Metabolism	HMDB0094701	-1.159	0.0564430107057958	0.0564430107057958	1.04026734009288	unchanged
Meta_132	Isovalerylglycine	Amino Acid	Leucine, Isoleucine and Valine Metabolism	HMDB0000678	0.206333333333333	0.565395714035001	0.565395714035001	0.412367695262683	unchanged
Meta_134	Pimelate (C7-DC)	Lipid	Fatty Acid, Dicarboxylate	HMDB0000857	-0.630333333	0.130935859794485	0.130935859794485	0.891667921537852	unchanged
Meta_136	2-HYDROXYOCTANOIC ACID	Lipid	Fatty Acid, Monohydroxy	HMDB0000711	-0.439666667	0.170400696365367	0.170400696365367	0.837830791750698	unchanged
Meta_137	Aminoadipic Acid	Amino Acid	Lysine Metabolism	HMDB0000510	-0.145333333	0.820209625573277	0.820209625573277	0.165307426165089	unchanged
Meta_138	N,N,N-Trimethyl-5-Aminovalerate	Amino Acid	Lysine Metabolism	HMDB0240732	0.198333333333334	0.169311814619255	0.169311814619255	0.841606870417212	unchanged
Meta_139	3-Hydroxy-3-Methylglutaric Acid	Amino Acid	Leucine, Isoleucine and Valine Metabolism	HMDB0029169	0.787333333333333	0.172916953098194	0.172916953098194	0.815869377122187	unchanged
Meta_140	Oxoadipic Acid	Amino Acid	Lysine Metabolism	HMDB0000225	0.209999999999999	0.316158471324643	0.316158471324643	0.648818974822488	unchanged
Meta_142	Tryptamine	Amino Acid	Tryptophan Metabolism	HMDB0000303	-0.196333333	0.782311342087516	0.782311342087516	0.195360490560423	unchanged
Meta_143	N6-Methyllysine	Amino Acid	Lysine Metabolism	HMDB0002038	0.159666666666666	0.748649451793011	0.748649451793011	0.227547244752883	unchanged
Meta_144	L-Beta-homolysine	Amino Acid	Other Amino Acid Metabolism		0.183999999999999	0.147439187082016	0.147439187082016	0.865675638041258	unchanged
Meta_145	Acetylcysteine	Amino Acid	Methionine, Cysteine, SAM and Taurine Metabolism	HMDB0001890	0.286333333333333	0.382403655127919	0.382403655127919	0.573722731147139	unchanged
Meta_146	N-Acetylthreonine	Amino Acid	Glycine, Serine and Threonine Metabolism	HMDB0062557	0.173	0.475615925599239	0.475615925599239	0.470394693852982	unchanged
Meta_149	L-Allothreonine	Amino Acid	Other Amino Acid Metabolism	HMDB0004041	-0.365	0.261082400379166	0.261082400379166	0.779401246979616	unchanged
Meta_15	Glycerol	Lipid	Glycerolipid and Phospholipid Metabolism	HMDB0000131	-0.035666667	0.868285455055758	0.868285455055758	0.120004687333721	unchanged
Meta_150	Methionine Methyl Ester	Xenobiotics	Chemical	HMDB0254602	-0.06	0.642820745874606	0.642820745874606	0.321958198655247	unchanged
Meta_155	Quinolinate	Cofactors and Vitamins	Nicotinate and Nicotinamide Metabolism	HMDB0000232	-0.766333333	0.316569345379457	0.316569345379457	0.656280804331929	unchanged
Meta_156	L-methionine sulfoxide	Amino Acid	Methionine, Cysteine, SAM and Taurine Metabolism	HMDB0002005	0.194333333333331	0.152857391684698	0.152857391684698	0.856784514368042	unchanged
Meta_158	Hordenine	Xenobiotics	Natural Product/Food/Plant	HMDB0004366	0.542999999999999	0.185856981202968	0.185856981202968	0.904463446972693	unchanged
Meta_160	Perillic acid	Xenobiotics	Natural Product/Food/Plant	HMDB0004586	-0.294333333	0.758507537933762	0.758507537933762	0.22111532642675	unchanged
Meta_163	Glyceraldehyde 3-Phosphate	Carbohydrate	Glycolysis, Gluconeogenesis, and Pyruvate Metabolism	HMDB0001112	-0.136	0.181395855117598	0.181395855117598	0.824836264666419	unchanged
Meta_167	Decanoic Acid (C10:0)	Lipid	Medium Chain Fatty Acid	HMDB0000511	0.0640000000000001	0.637564031185678	0.637564031185678	0.329680974350934	unchanged
Meta_168	Phenol Sulfate	Amino Acid	Tyrosine Metabolism	HMDB0060015	-0.786666667	0.437309916734416	0.437309916734416	0.526499593850863	unchanged
Meta_169	Aconitic Acid	Carbohydrate	Citrate cycle (TCA cycle)	HMDB0000072	0.0410000000000004	0.871565935232168	0.871565935232168	0.119620793720678	unchanged
Meta_17	3-Hydroxypyridine	Xenobiotics	Chemical	HMDB0240652	0.411666666666665	0.386877512983064	0.386877512983064	0.606600804313833	unchanged
Meta_170	Suberate (C8-DC)	Lipid	Fatty Acid, Dicarboxylate	HMDB0000893	-0.342333333	0.206717001501078	0.206717001501078	0.816688763084252	unchanged
Meta_173	Shikimic Acid	Amino Acid	Shikimic acid pathway	HMDB0003070	0.721	0.0635099718388425	0.0635099718388425	1.09588741932121	unchanged
Meta_177	N-Formylmethionine	Amino Acid	Methionine, Cysteine, SAM and Taurine Metabolism	HMDB0001015	-0.01	0.957389384827848	0.957389384827848	0.0363271847869604	unchanged
Meta_179	Citrulline	Amino Acid	Urea cycle; Arginine and Proline Metabolism	HMDB0000904	-0.127	0.394014123376557	0.394014123376557	0.579844972233206	unchanged
Meta_18	Phosphate	Energy	Oxidative Phosphorylation	HMDB0001429	0.319333333333333	0.0921013651455683	0.0921013651455683	0.966082436001581	unchanged
Meta_184	Caffeic Acid	Xenobiotics	Natural Product/Food/Plant	HMDB0001964	1.207	0.221058592133888	0.221058592133888	0.790349577543704	unchanged
Meta_187	GALACTITOL	Carbohydrate	Polysaccharides and Oligosaccharides	HMDB0000107	-1.199333333	0.0530371285041324	0.0530371285041324	1.08754522385918	unchanged
Meta_188	Pyridoxate	Cofactors and Vitamins	Vitamin B6 Metabolism	HMDB0000017	0.596333333333334	0.196006207566239	0.196006207566239	0.799499628321471	unchanged
Meta_19	2-Piperidinone	Xenobiotics	Natural Product/Food/Plant	HMDB0011749	0.262999999999998	0.418100525135873	0.418100525135873	0.558613847878921	unchanged
Meta_190	Phosphoserine	Amino Acid	Glycine, Serine and Threonine Metabolism	HMDB0000272	1.54366666666667	0.120932624776442	0.120932624776442	0.901113610116824	unchanged
Meta_194	Undecanoic Acid (C11:0)	Lipid	Medium Chain Fatty Acid	HMDB0000947	-0.093666667	0.62614048224166	0.62614048224166	0.348380020161908	unchanged
Meta_198	3-Hydroxydecanoate	Lipid	Fatty Acid, Monohydroxy	HMDB0002203	-0.284333333	0.57129175058252	0.57129175058252	0.385225378500267	unchanged
Meta_201	N1/N8-Acetylspermidine	Amino Acid	Polyamine Metabolism	HMDB0001276	-0.500333333	0.179568218809502	0.179568218809502	0.846552084416837	unchanged
Meta_202	2,5-Dihydroxybenzenesulfonate	Xenobiotics	Chemical	HMDB0245498	0.573	0.185399087270706	0.185399087270706	0.805022606805555	unchanged
Meta_209	N-Acetylmethionine	Amino Acid	Methionine, Cysteine, SAM and Taurine Metabolism	HMDB0011745	0.372333333333334	0.1562223548121	0.1562223548121	0.856340398437833	unchanged
Meta_21	5-Valerolactone	Xenobiotics	Chemical		-0.069666667	0.707668743043897	0.707668743043897	0.249786055856413	unchanged
Meta_211	Lactoylvaline	Peptide	Pseudodipeptides (N-Lactoyl-amino acids)	HMDB0062181	0.169666666666666	0.230638986985297	0.230638986985297	0.803759962094603	unchanged
Meta_212	Citric Acid/Isocitric Acid	Carbohydrate	Citrate cycle (TCA cycle)	HMDB0000193	0.0493333333333332	0.848161431087389	0.848161431087389	0.130803756215621	unchanged
Meta_213	Citric Acid	Carbohydrate	Citrate cycle (TCA cycle)	HMDB0000094	0.196000000000002	0.439892319624367	0.439892319624367	0.506959629826599	unchanged
Meta_215	Glucuronate	Carbohydrate	Amino sugar and Nucleotide sugar Metabolism	HMDB0000127	-0.03	0.903528168821949	0.903528168821949	0.091654736225293	unchanged
Meta_218	m-Cumenyl methylcarbamate	Unclassified	Unclassified		0.0919999999999987	0.663828483376807	0.663828483376807	0.306661225465475	unchanged

Meta_22	Isovaleric Acid	Amino Acid	Leucine, Isoleucine and Valine Metabolism	HMDB0000718	0.856333333333332	0.156977667060397	0.156977667060397	0.923568864945084	unchanged
Meta_222	5-Dodecenoic Acid (C12:1n7)	Lipid	Medium Chain Fatty Acid	HMDB0000529	0.334000000000001	0.226076590191192	0.226076590191192	0.763850459419621	unchanged
Meta_223	Erythrose-4-Phosphate	Carbohydrate	Pentose phosphate pathway	HMDB0001321	1.229	0.286011781222729	0.286011781222729	0.693989072759342	unchanged
Meta_224	Dodecanoic Acid (C12:0)	Lipid	Medium Chain Fatty Acid	HMDB0000638	0.239000000000001	0.0885218348818936	0.0885218348818936	0.997538746000051	unchanged
Meta_225	Cysteine S-sulfate	Amino Acid	Methionine, Cysteine, SAM and Taurine Metabolism	HMDB0000731	-0.465666667	0.384582154926525	0.384582154926525	0.582449698944296	unchanged
Meta_226	Sebacate (C10-DC)	Lipid	Fatty Acid, Dicarboxylate	HMDB0000792	-0.234666667	0.268578688323221	0.268578688323221	0.72527335475943	unchanged
Meta_227	1-Carboxyethylisoleucine	Amino Acid	Leucine, Isoleucine and Valine Metabolism	HMDB0242158	0.137666666666666	0.736714887662434	0.736714887662434	0.229747082741979	unchanged
Meta_228	25-HYDROXYCHOLESTEROL	Lipid	Sterol	HMDB0006247	-0.367	0.0749031741531224	0.0749031741531224	1.08785759458052	unchanged
Meta_23	Gamma-Aminobutyric Acid	Amino Acid	Alanine, Aspartate and Glutamate Metabolism	HMDB0000112	-0.097	0.619401757253791	0.619401757253791	0.346537336782829	unchanged
Meta_234	Spermine	Unclassified	Unclassified		-0.207333333	0.788686503660893	0.788686503660893	0.179929550441405	unchanged
Meta_235	L-Acetylcarnitine	Lipid	Fatty Acid Metabolism (Acyl Carnitine)	HMDB0000201	0.00233333333333263	0.989516329123352	0.989516329123352	0.0142464666243961	unchanged
Meta_236	Lactoylleucine	Peptide	Pseudodipeptides (N-Lactoyl-amino acids)	HMDB0062176	0.0156666666666681	0.931855034025094	0.931855034025094	0.0653464309896408	unchanged
Meta_237	2-Methylcitrate/Homocitrate	Carbohydrate	Citrate cycle (TCA cycle)	HMDB0003518	-3.257	0.0805077850482409	0.0805077850482409	1.09086876501191	unchanged
Meta_238	L-Tryptophan	Amino Acid	Tryptophan Metabolism	HMDB0000929	0.587000000000003	0.0692311541952582	0.0692311541952582	1.02894998490298	unchanged
Meta_240	4-Octylphenol	Xenobiotics	Chemical	HMDB0246557	0.0396666666666654	0.318727056745804	0.318727056745804	0.658968966875849	unchanged
Meta_242	Indolelactate	Amino Acid	Tryptophan Metabolism	HMDB0000671	-1.401	0.166698169738454	0.166698169738454	0.927726625779803	unchanged
Meta_245	Gly-Met	Peptide	Dipeptide	HMDB0028847	-0.092333333	0.787875316515675	0.787875316515675	0.185358714433905	unchanged
Meta_246	L-Kynurenine	Amino Acid	Tryptophan Metabolism	HMDB0000684	0.0690000000000008	0.836624451308222	0.836624451308222	0.146452747064217	unchanged
Meta_249	1,3-Diphenylguanidine	Xenobiotics	Chemical		-0.235666667	0.592768306898782	0.592768306898782	0.363989050674613	unchanged
Meta_250	Perfluorobutyric Acid	Xenobiotics	Chemical	HMDB0253102	0.624333333333334	0.344880835359207	0.344880835359207	0.627966877276758	unchanged
Meta_253	3-Hydroxylaurate	Lipid	Fatty Acid, Monohydroxy	HMDB0000387	-0.824	0.253448805349998	0.253448805349998	0.786710962181001	unchanged
Meta_259	Gamma-Glutamylalanine	Peptide	Gamma-glutamyl Amino Acid	HMDB0006248	-1.924	0.117136286771475	0.117136286771475	1.01436564296903	unchanged
Meta_260	N-Acetylserotonin	Amino Acid	Tryptophan Metabolism	HMDB0001238	0.336000000000002	0.145883653855802	0.145883653855802	0.885238116620647	unchanged
Meta_264	5-Hydroxy-L-Tryptophan	Amino Acid	Tryptophan Metabolism	HMDB0000472	0.214	0.26372718300058	0.26372718300058	0.702689498890945	unchanged
Meta_268	Tetradecadienoate (14:2)	Lipid	Medium Chain Fatty Acid	HMDB0340799	-0.037666667	0.972790902557165	0.972790902557165	0.0318348194202646	unchanged
Meta_269	Androstenediol (3Beta,17Beta) Disulfate (2)	Lipid	Androgenic Steroids	HMDB0240313	-0.303	0.652483179342354	0.652483179342354	0.309272037991949	unchanged
Meta_27	L-Serine	Amino Acid	Glycine, Serine and Threonine Metabolism	HMDB0000187	0.668666666666667	0.0949483239058976	0.0949483239058976	0.982663001980731	unchanged
Meta_273	Dicyclohexylurea	Xenobiotics	Chemical	HMDB0244166	-1.204	0.0645083406714942	0.0645083406714942	1.04203608994336	unchanged
Meta_274	Vitamin K1	Cofactors and Vitamins	Vitamin K Metabolism	HMDB0003555	-0.830333333	0.117142220622313	0.117142220622313	0.991162991398632	unchanged
Meta_275	Traumatic Acid	Lipid	Fatty Acid, Dicarboxylate	HMDB0000933	-0.152666667	0.690394674125541	0.690394674125541	0.273494162502801	unchanged
Meta_279	Dodecanedioate (C12-DC)	Lipid	Fatty Acid, Dicarboxylate	HMDB0000623	-0.076333333	0.483807754524712	0.483807754524712	0.47477748042527	unchanged
Meta_286	Butyrylcarnitine	Lipid	Fatty Acid Metabolism (Acyl Carnitine)	HMDB0002013	-0.596	0.0986801612020981	0.0986801612020981	0.956979649892749	unchanged
Meta_288	Formylkynurenine	Amino Acid	Tryptophan Metabolism	HMDB0001200	-0.377	0.316509628612309	0.316509628612309	0.687990195047023	unchanged
Meta_289	Lactoylphenylalanine	Peptide	Pseudodipeptides (N-Lactoyl-amino acids)	HMDB0062175	0.292	0.104914808653163	0.104914808653163	0.991016979091763	unchanged
Meta_290	Pentadecenoic Acid (C15:1n5)	Lipid	Long Chain Fatty Acid	HMDB0302623	0.528666666666668	0.297335193352014	0.297335193352014	0.674213098456898	unchanged
Meta_291	7,8-Dihydrobiopterin	Cofactors and Vitamins	Folate Metabolism	HMDB0000038	0.835666666666667	0.173246457472647	0.173246457472647	0.887788721759392	unchanged
Meta_292	Thymidine	Nucleotide	Pyrimidine Metabolism, Thymine containing	HMDB0000273	1.662	0.0663277407312969	0.0663277407312969	1.02053558557764	unchanged
Meta_293	Pyroglutamylleucine	Peptide	Dipeptide	HMDB0341382	0.566666666666666	0.599507791877094	0.599507791877094	0.358660015011554	unchanged
Meta_294	N,N-Dimethyl-Proline-Proline Betaine	Amino Acid	Urea cycle; Arginine and Proline Metabolism		0.810000000000001	0.0981914513714646	0.0981914513714646	0.951851556087659	unchanged
Meta_295	Pentadecanoic Acid (C15:0)	Lipid	Long Chain Fatty Acid	HMDB0000826	0.0143333333333349	0.89971360464234	0.89971360464234	0.0884561876141792	unchanged
Meta_296	Pseudouridine	Nucleotide	Pyrimidine Metabolism, Uracil containing	HMDB0000767	-0.959	0.074752538066481	0.074752538066481	0.991298398806188	unchanged
Meta_3	Propanoic Acid	Carbohydrate	Propanoate Metabolism	HMDB0000237	0.443000000000001	0.285828829118855	0.285828829118855	0.708780808010627	unchanged
Meta_30	Guaiacol	Xenobiotics	Natural Product/Food/Plant	HMDB0001398	0.599999999999998	0.062224505609369	0.062224505609369	1.08305277716086	unchanged
Meta_300	Tridecanedioic acid	Lipid	Long Chain Fatty Acid	HMDB0002327	-0.516	0.0941739813120576	0.0941739813120576	0.969224544645538	unchanged
Meta_302	Glycerophosphoglycerol	Lipid	Glycerolipid and Phospholipid Metabolism	HMDB0240316	0.64533333333333	0.111588313358826	0.111588313358826	1.01928519864396	unchanged
Meta_303	Citicoline	Xenobiotics	Drug	HMDB0001413	0.0683333333333334	0.805627649147693	0.805627649147693	0.175432799476528	unchanged
Meta_307	Leu-Ile	Peptide	Dipeptide	HMDB0028932	-1.008	0.717124087412831	0.717124087412831	0.265940805804736	unchanged
Meta_31	2-AMINOPHENOL	Amino Acid	Tryptophan Metabolism	HMDB0001169	0.276666666666666	0.195859612237269	0.195859612237269	0.876906431592101	unchanged
Meta_311	Valerylcarnitine (C5)	Amino Acid	Leucine, Isoleucine and Valine Metabolism	HMDB0013128	-0.539	0.272427373574797	0.272427373574797	0.770802908271701	unchanged
Meta_314	5,6-Dihydrouridine	Nucleotide	Pyrimidine Metabolism, Uracil containing	HMDB0000497	-0.904666667	0.0868439592226531	0.0868439592226531	1.07302148133377	unchanged
Meta_316	Pyridoxal 5'-Phosphate	Cofactors and Vitamins	Vitamin B6 Metabolism	HMDB0001491	0.181666666666667	0.513912567705927	0.513912567705927	0.432457544162071	unchanged
Meta_317	3-Hydroxybutyrylcarnitine	Lipid	Fatty Acid Metabolism (Acyl Carnitine)	HMDB0013127	0.110000000000001	0.81216873163466	0.81216873163466	0.166522628808139	unchanged
Meta_318	Gamma-Glutamylthreonine	Peptide	Gamma-glutamyl Amino Acid	HMDB0029159	-0.131333333	0.698685406289864	0.698685406289864	0.260553588765829	unchanged
Meta_324	Palmitoleic Acid (C16:1)	Lipid	Long Chain Fatty Acid	HMDB0003229	0.195666666666664	0.253697606422089	0.253697606422089	0.713115350490131	unchanged
Meta_325	Nicotinamide riboside (NR)	Cofactors and Vitamins	Nicotinate and Nicotinamide Metabolism	HMDB0000855	0.409000000000001	0.237364223860886	0.237364223860886	0.761452327590032	unchanged
Meta_328	Lauroylglycine	Lipid	Fatty Acid Metabolism (Acyl glycines)	HMDB0013272	0.109999999999999	0.881958457600453	0.881958457600453	0.114982206454807	unchanged
Meta_329	Palmitamide	Lipid	Fatty Acid Metabolism (Fatty Amide)	HMDB0012273	0.0296666666666638	0.812479955139677	0.812479955139677	0.168602134376226	unchanged
Meta_33	Pyrrole-2-Carboxylic Acid	Xenobiotics	Benzoate Metabolism	HMDB0004230	-0.503666667	0.211753553665024	0.211753553665024	0.816423332773726	unchanged
Meta_331	Tetradecanedioate (C14-DC)	Lipid	Fatty Acid, Dicarboxylate	HMDB0000872	-0.035666667	0.85310382628709	0.85310382628709	0.119444034915111	unchanged
Meta_332	Glucosamine 6-Phosphate	Carbohydrate	Amino sugar and Nucleotide sugar Metabolism	HMDB0001254	-0.154333333	0.422780940620194	0.422780940620194	0.53333306197806	unchanged
Meta_335	N,N-Dimethyltetradecylamine-N-Oxide	Xenobiotics	Natural Product/Food/Plant		3.08733333333333	0.240712784596315	0.240712784596315	0.811867013692239	unchanged
Meta_343	Glucose 1-Phosphate	Carbohydrate	Glycolysis, Gluconeogenesis, and Pyruvate Metabolism	HMDB0001586	-0.428333333	0.207301399538879	0.207301399538879	0.800285010620185	unchanged
Meta_349	Gly-Trp	Peptide	Dipeptide	HMDB0028852	-2.305333333	0.0852674019615898	0.0852674019615898	1.07756162201238	unchanged
Meta_35	Uracil	Nucleotide	Pyrimidine Metabolism, Uracil containing	HMDB0000300	0.0106666666666655	0.979259692136147	0.979259692136147	0.0135650840184279	unchanged

Meta_350	3-Hydroxyisovalerylcarnitine	Amino Acid	Leucine, Isoleucine and Valine Metabolism	HMDB0062555	0.713000000000001	0.0614957769432118	0.0614957769432118	1.04505949988882	unchanged
Meta_352	Thiamine	Cofactors and Vitamins	Vitamin B1 Metabolism	HMDB0000235	-0.764666667	0.25609155341275	0.25609155341275	0.764561251454665	unchanged
Meta_355	1,3-Diphopshateglycerate	Carbohydrate	Glycolysis, Gluconeogenesis, and Pyruvate Metabolism	HMDB0001294	1.02366666666667	0.112194696897584	0.112194696897584	0.98648969015254	unchanged
Meta_356	Lauryl Sulfate	Xenobiotics	Natural Product/Food/Plant		0.321666666666669	0.47885791499306	0.47885791499306	0.467165119928348	unchanged
Meta_359	Deoxyguanosine	Nucleotide	Purine Metabolism, Guanine containing	HMDB0000085	0.0233333333333334	0.877327591858191	0.877327591858191	0.105439803014149	unchanged
Meta_36	Fumaric Acid	Carbohydrate	Citrate cycle (TCA cycle)	HMDB0000134	0.306666666666668	0.446950132252002	0.446950132252002	0.515262723505147	unchanged
Meta_363	Metoprolol acid	Xenobiotics	Drug	HMDB0254682	-1.418	0.133924838442116	0.133924838442116	0.962009960982997	unchanged
Meta_365	Inosine	Nucleotide	Purine Metabolism, (Hypo)Xanthine/Inosine containing	HMDB0000195	-0.276	0.450871745718229	0.450871745718229	0.534106640588975	unchanged
Meta_369	16-Hydroxyhexadecanoic acid	Lipid	Fatty Acid, Monohydroxy	HMDB0006294	-0.204	0.390326230824065	0.390326230824065	0.585164405618848	unchanged
Meta_37	Maleic Acid (C4:1-DC)	Lipid	Fatty Acid, Dicarboxylate	HMDB0000176	0.471666666666668	0.171426723654711	0.171426723654711	0.884344271180631	unchanged
Meta_371	Lauryl diethanolamine	Lipid	Endocannabinoid (N-acylethanolamine)		-0.275666667	0.623947018810206	0.623947018810206	0.334003259483101	unchanged
Meta_376	Terbucarb	Xenobiotics	Chemical		0.0116666666666667	0.981151382250218	0.981151382250218	0.021774286647207	unchanged
Meta_377	Gamma-Glutamylmethionine	Peptide	Gamma-glutamyl Amino Acid	HMDB0034367	-0.319333333	0.386204629053963	0.386204629053963	0.585937547686431	unchanged
Meta_38	Ketoisovaleric Acid	Amino Acid	Leucine, Isoleucine and Valine Metabolism	HMDB0000019	0.462666666666667	0.137214627951952	0.137214627951952	0.945231627731572	unchanged
Meta_382	Asp-Phe	Peptide	Dipeptide	HMDB0000706	-0.315	0.200058341832127	0.200058341832127	0.868561696627147	unchanged
Meta_385	9-Octadecenamide	Lipid	Fatty Acid Metabolism (Fatty Amide)	HMDB0002117	0.277333333333335	0.0615584958380503	0.0615584958380503	1.12189507650871	unchanged
Meta_386	Stearic Acid (C18:0)	Lipid	Long Chain Fatty Acid	HMDB0000827	0.131666666666668	0.112383786726336	0.112383786726336	0.916356217838808	unchanged
Meta_389	Stearamide	Lipid	Fatty Acid Metabolism (Fatty Amide)	HMDB0034146	0.00633333333333397	0.974184059174294	0.974184059174294	0.0302030630019314	unchanged
Meta_39	3-Methyl-2-Oxobutyrate	Amino Acid	Leucine, Isoleucine and Valine Metabolism	HMDB0000019	1.48066666666667	0.172323025651731	0.172323025651731	0.928188812299249	unchanged
Meta_390	Xanthosine	Nucleotide	Purine Metabolism, (Hypo)Xanthine/Inosine containing	HMDB0000299	1.412	0.0630060620090929	0.0630060620090929	1.03598699226703	unchanged
Meta_391	Hexadecanedioate (C16-DC)	Lipid	Fatty Acid, Dicarboxylate	HMDB0000672	0.0876666666666672	0.729267550486812	0.729267550486812	0.249096843068865	unchanged
Meta_392	Arg-Leu	Peptide	Dipeptide	HMDB0028713	-1.401666667	0.0815633772715569	0.0815633772715569	0.998828544862049	unchanged
Meta_398	Argininosuccinic acid	Amino Acid	Urea cycle; Arginine and Proline Metabolism	HMDB0000052	1.08833333333333	0.0534773311221745	0.0534773311221745	1.13439302467573	unchanged
Meta_399	6-Gingerol	Xenobiotics	Natural Product/Food/Plant	HMDB0005783	-0.126	0.49207336763054	0.49207336763054	0.456362156239593	unchanged
Meta_4	Glycolic Acid	Carbohydrate	Glyoxylate and dicarboxylate metabolism	HMDB0000115	0.199000000000002	0.271003414981576	0.271003414981576	0.708337776818279	unchanged
Meta_40	Caproic Acid (C6:0)/4-methylvaleric acid	Lipid	Medium Chain Fatty Acid	HMDB0000535	1.507	0.176688233090922	0.176688233090922	0.899428312521013	unchanged
Meta_400	Tetradecylsulfate	Xenobiotics	Chemical		0.00766666666666538	0.974597582558365	0.974597582558365	0.0255517075681005	unchanged
Meta_404	13-HODE + 9-HODE	Lipid	Fatty Acid, Monohydroxy	HMDB0004667	0.232666666666665	0.267200741404403	0.267200741404403	0.721397333068223	unchanged
Meta_405	9-HODE	Lipid	Fatty Acid Metabolism (Linolenic acid metabolism)	HMDB0004670	-0.082333333	0.341533800016475	0.341533800016475	0.627463941228273	unchanged
Meta_408	Ricinoleic Acid	Lipid	Fatty Acid, Monohydroxy	HMDB0034297	0.389999999999999	0.0546937429035302	0.0546937429035302	1.1100878414084	unchanged
Meta_410	(9Z,12R)-12-Hydroxyoctadec-9-enoic acid	Lipid	Fatty Acid, Monohydroxy	HMDB0034297	0.343	0.167993851775867	0.167993851775867	0.897474107823581	unchanged
Meta_411	5-Methylthioadenosine (MTA)	Amino Acid	Polyamine Metabolism	HMDB0001173	0.625	0.237942105943785	0.237942105943785	0.736795954948343	unchanged
Meta_413	Palmitoleoyl ethanolamide	Lipid	Endocannabinoid (N-acylethanolamine)	HMDB0013648	0.310666666666666	0.312103922625028	0.312103922625028	0.674984407393545	unchanged
Meta_414	Perfluorobutane Sulfonic Acid	Xenobiotics	Chemical	HMDB0246220	-0.203666667	0.0556297533696033	0.0556297533696033	1.1062305590267	unchanged
Meta_415	12-Hydroxyoctadecanoic acid	Lipid	Fatty Acid, Monohydroxy	HMDB0061706	-0.038666667	0.755189136605993	0.755189136605993	0.217437434548822	unchanged
Meta_416	2-Hydroxystearate	Lipid	Fatty Acid, Monohydroxy	HMDB0062549	0.0396666666666672	0.833434681523452	0.833434681523452	0.137141382762105	unchanged
Meta_417	N-Acetylglucosamine 1-phosphate	Carbohydrate	Amino sugar and Nucleotide sugar Metabolism	HMDB0001367	-1.386	0.0600778023886833	0.0600778023886833	1.02946415780842	unchanged
Meta_418	Palmitoyl ethanolamide	Lipid	Endocannabinoid (N-acylethanolamine)	HMDB0002100	0.917666666666667	0.178073590207974	0.178073590207974	0.83195518331992	unchanged
Meta_42	L-Norvaline	Amino Acid	Other Amino Acid Metabolism	HMDB0013716	0.281333333333333	0.545574384834964	0.545574384834964	0.424571126245933	unchanged
Meta_421	Tetradecyl diethanolamine	Lipid	Endocannabinoid (N-acylethanolamine)		0.137666666666668	0.483282324704341	0.483282324704341	0.469893550715602	unchanged
Meta_422	N-Acetyl-Aspartyl-Glutamate (NAAG)	Amino Acid	Alanine, Aspartate and Glutamate Metabolism	HMDB0001067	0.731666666666666	0.0513483391260581	0.0513483391260581	1.05900942442681	unchanged
Meta_423	12-HETE	Lipid	Eicosanoid	HMDB0006111	0.821	0.452812135310694	0.452812135310694	0.506928985794919	unchanged
Meta_427	dCMP	Nucleotide	Pyrimidine Metabolism, Cytidine containing	HMDB0001202	-1.333333333	0.0954687461550794	0.0954687461550794	1.06058225219784	unchanged
Meta_428	Epigallocatechin	Xenobiotics	Natural Product/Food/Plant	HMDB0001871	0.391999999999999	0.283626003962967	0.283626003962967	0.746477192752133	unchanged
Meta_429	Eicosadienoic Acid (C20:2n6)	Lipid	Long Chain Fatty Acid	HMDB0005060	0.726333333333333	0.0582051165551219	0.0582051165551219	1.12581551034485	unchanged
Meta_432	D-Ribulose 1,5-Bisphosphate	Carbohydrate	Pentose phosphate pathway	HMDB0304322	-0.159666667	0.299124574204366	0.299124574204366	0.661122346603935	unchanged
Meta_433	Fructosylysine	Xenobiotics	Natural Product/Food/Plant	HMDB0252495	0.335333333333333	0.1068714636111	0.1068714636111	0.964714930442244	unchanged
Meta_434	Mestranol	Xenobiotics	Drug	HMDB0015446	-0.217666667	0.490872599009602	0.490872599009602	0.470118204840445	unchanged
Meta_435	Eicosenoic Acid (C20:1)	Lipid	Long Chain Fatty Acid	HMDB0062436	0.612666666666668	0.125403506482594	0.125403506482594	0.900468289021511	unchanged
Meta_437	Arachidate (C20:0)	Lipid	Long Chain Fatty Acid	HMDB0002212	0.110000000000001	0.291679190522493	0.291679190522493	0.668045584418999	unchanged
Meta_439	Palmitoylglycine	Lipid	Fatty Acid Metabolism (Acyl glycines)	HMDB0013034	0.135666666666667	0.545952649353446	0.545952649353446	0.40895642061803	unchanged
Meta_44	Erythronolactone	Xenobiotics	Natural Product/Food/Plant	HMDB0000349	0.660666666666666	0.316662883877417	0.316662883877417	0.650844009271405	unchanged
Meta_441	Octadecanedioate (C18-DC)	Lipid	Fatty Acid, Dicarboxylate	HMDB0000782	-0.606	0.0932493814174398	0.0932493814174398	1.00185606624641	unchanged
Meta_442	Leu-Trp	Peptide	Dipeptide	HMDB0028940	-2.104333333	0.130090651062682	0.130090651062682	0.96619386880035	unchanged
Meta_444	D-Ribo-Phytosphingosine	Lipid	Sphingolipid Metabolism	HMDB0004610	-0.089666667	0.875462207279111	0.875462207279111	0.111359463835149	unchanged
Meta_446	Beta-Citrylglutamate	Amino Acid	Alanine, Aspartate and Glutamate Metabolism	HMDB0013220	0.431999999999999	0.241200316892229	0.241200316892229	0.765297156184759	unchanged
Meta_447	CMP	Nucleotide	Pyrimidine Metabolism, Cytidine containing	HMDB0000095	-0.135666667	0.140535001859787	0.140535001859787	0.876851419654769	unchanged
Meta_450	Dodecylbenzenesulfonic acid	Xenobiotics	Chemical	HMDB0031031	0.160666666666666	0.268659267296463	0.268659267296463	0.691515988995381	unchanged
Meta_451	Oleoyl ethanolamide	Lipid	Endocannabinoid (N-acylethanolamine)	HMDB0002088	0.898000000000002	0.056879338772902	0.056879338772902	1.03992679448243	unchanged
Meta_453	Docosahexaenoic Acid (C22:6n3 DHA)	Lipid	Long Chain Fatty Acid	HMDB0002183	0.859999999999999	0.0870712033083891	0.0870712033083891	1.02111066524935	unchanged
Meta_458	Trp-Glu	Peptide	Dipeptide	HMDB0029082	-0.970333333	0.257250108044224	0.257250108044224	0.767699486698479	unchanged
Meta_46	2-Hydroxyisovaleric acid	Amino Acid	Leucine, Isoleucine and Valine Metabolism	HMDB0000407	-0.066	0.63203385348246	0.63203385348246	0.320461315960644	unchanged
Meta_460	13,16-Docosadienoic Acid (C22:2n6)	Lipid	Long Chain Fatty Acid	HMDB0061714	0.637000000000001	0.20502119334349	0.20502119334349	0.786559151445264	unchanged
Meta_461	Erucate (22:1n9)	Lipid	Long Chain Fatty Acid	HMDB0002068	0.804333333333334	0.207675695052615	0.207675695052615	0.799287730776359	unchanged

Meta_462	Oleoylglycine	Lipid	Fatty Acid Metabolism (Acyl glycines)	HMDB0013631	0.0326666666666657	0.967788474406518	0.967788474406518	0.0324611934848423	unchanged
Meta_464	Fructose 1,6-Bisphosphate	Carbohydrate	Glycolysis, Gluconeogenesis, and Pyruvate Metabolism	HMDB0001058	-0.184333333	0.187662276823879	0.187662276823879	0.820814398078962	unchanged
Meta_465	Canrenone	Xenobiotics	Drug	HMDB0003033	0.171333333333333	0.122256248025563	0.122256248025563	0.919220951359101	unchanged
Meta_466	Stearoylglycine	Lipid	Fatty Acid Metabolism (Acyl glycines)	HMDB0013308	-0.415666667	0.269101498667094	0.269101498667094	0.739130910059287	unchanged
Meta_467	Sucrose	Carbohydrate	Polysaccharides and Oligosaccharides	HMDB0000258	0.124666666666668	0.471085743800934	0.471085743800934	0.507396073754943	unchanged
Meta_469	His-Trp	Peptide	Dipeptide	HMDB0028896	-0.529	0.184853616161485	0.184853616161485	0.850305723296265	unchanged
Meta_471	dGMP	Nucleotide	Purine Metabolism, Guanine containing	HMDB0001044	0.306666666666667	0.451979126506552	0.451979126506552	0.529410376057101	unchanged
Meta_472	AMP	Nucleotide	Purine Metabolism, Adenine containing	HMDB0000045	0.634	0.164124872516218	0.164124872516218	0.901110356632791	unchanged
Meta_473	IMP	Nucleotide	Purine Metabolism, (Hypo)Xanthine/Inosine containing	HMDB0000175	0.451333333333333	0.328675445646821	0.328675445646821	0.619606744517574	unchanged
Meta_475	TRICOSANOIC ACID	Xenobiotics	Natural Product/Food/Plant	HMDB0001160	-0.081	0.940415624590801	0.940415624590801	0.0482336379534236	unchanged
Meta_478	Cortisol	Lipid	Corticosteroids	HMDB0000063	0.379	0.407721300565825	0.407721300565825	0.561366879702003	unchanged
Meta_48	2-Hydroxyvaleric acid	Amino Acid	Leucine, Isoleucine and Valine Metabolism	HMDB0001863	0.214666666666666	0.318939234835428	0.318939234835428	0.666453099720262	unchanged
Meta_481	melibiose	Carbohydrate	Polysaccharides and Oligosaccharides	HMDB0000048	-0.163666667	0.621630464185522	0.621630464185522	0.350655929015626	unchanged
Meta_486	N6-Succinyladenosine	Nucleotide	Purine Metabolism, Adenine containing	HMDB0000912	2.564	0.0553724907970361	0.0553724907970361	1.15190085009667	unchanged
Meta_487	S-Adenosylhomocysteine	Amino Acid	Methionine, Cysteine, SAM and Taurine Metabolism	HMDB0000939	-1.066	0.116777952372538	0.116777952372538	1.00047830720408	unchanged
Meta_488	4-Cholesten-3-One	Lipid	Sterol	HMDB0000921	0.0199999999999996	0.981925709393034	0.981925709393034	0.0165639485467682	unchanged
Meta_489	dCDP	Nucleotide	Pyrimidine Metabolism, Cytidine containing	HMDB0001245	0.294333333333334	0.141062049170741	0.141062049170741	0.901228632743875	unchanged
Meta_492	Hyodeoxycholic Acid	Lipid	Bile Acid	HMDB0000733	0.452333333333334	0.416924238520366	0.416924238520366	0.583831413395833	unchanged
Meta_493	Deoxycholic Acid	Lipid	Bile Acid	HMDB0000626	-0.106333333	0.546801968389666	0.546801968389666	0.429796452624275	unchanged
Meta_497	dTDP	Nucleotide	Pyrimidine Metabolism, Thymine containing	HMDB0001274	0.588666666666667	0.256229698764788	0.256229698764788	0.725291812411001	unchanged
Meta_498	7-Oxocholesterol	Lipid	Sterol	HMDB0000501	0.346666666666666	0.353978906924057	0.353978906924057	0.619140925323331	unchanged
Meta_499	CDP	Nucleotide	Pyrimidine Metabolism, Cytidine containing	HMDB0001546	-0.837	0.0525169632876117	0.0525169632876117	1.13331565974361	unchanged
Meta_5	Trimethylamine N-Oxide	Lipid	Glycerolipid and Phospholipid Metabolism	HMDB0000925	0.331	0.425611377285109	0.425611377285109	0.536193928581913	unchanged
Meta_50	ALLOTHREONINE	Amino Acid	Glycine, Serine and Threonine Metabolism	HMDB00004041	-0.339	0.318460796293567	0.318460796293567	0.674438916793801	unchanged
Meta_500	UDP	Nucleotide	Pyrimidine Metabolism, Uracil containing	HMDB0000295	-0.250333333	0.52550492074885	0.52550492074885	0.452421431432102	unchanged
Meta_501	LPA 16:1_sn2	Lipid	Glycerophospholipid (lysophosphatidic acid)		-0.025	0.967732035221826	0.967732035221826	0.0190605988512951	unchanged
Meta_502	LPA 16:1_sn1	Lipid	Glycerophospholipid (lysophosphatidic acid)		1.64066666666667	0.0581500574754169	0.0581500574754169	1.07841497596859	unchanged
Meta_503	b-Muricholic Acid	Lipid	Bile Acid	HMDB0000415	-1.329666667	0.0893195844375215	0.0893195844375215	1.06414691789918	unchanged
Meta_504	Cholic Acid	Lipid	Bile Acid	HMDB0000619	1.34766666666667	0.116103896159967	0.116103896159967	1.02156311034909	unchanged
Meta_505	Leu-Leu-Tyr	Peptide	Small peptide	HMDB0304804	0.712333333333332	0.143807686504912	0.143807686504912	0.893809501980181	unchanged
Meta_513	dGDP	Nucleotide	Purine Metabolism, Guanine containing	HMDB0000960	-0.801	0.205483536014304	0.205483536014304	0.869611194803828	unchanged
Meta_514	ADP	Nucleotide	Purine Metabolism, Adenine containing	HMDB0001341	-1.562666667	0.130146654265377	0.130146654265377	0.967113572713313	unchanged
Meta_517	LPA 18:1_sn2	Lipid	Glycerophospholipid (lysophosphatidic acid)	HMDB0007855	3.293	0.0543590138523376	0.0543590138523376	1.14978899034772	unchanged
Meta_52	Betaine	Amino Acid	Glycine, Serine and Threonine Metabolism	HMDB0000043	0.391000000000002	0.143032498548489	0.143032498548489	0.917981058773283	unchanged
Meta_531	5-O-Methylvisammioside	Xenobiotics	Natural Product/Food/Plant		0.0953333333333326	0.76594142133863	0.76594142133863	0.21274461260903	unchanged
Meta_534	Flavin Mononucleotide	Cofactors and Vitamins	Vitamin B2 Metabolism	HMDB0001520	0.363333333333335	0.26014558962517	0.26014558962517	0.747108449727626	unchanged
Meta_54	1H-Benzotriazole	Xenobiotics	Chemical	HMDB0244873	-0.488666667	0.459332895499876	0.459332895499876	0.493849574996479	unchanged
Meta_542	Cholesteryl Sulfate	Lipid	Sterol	HMDB0000653	1.03066666666667	0.208651943282868	0.208651943282868	0.861458234804169	unchanged
Meta_546	LPE 17:0	Lipid	Glycerophospholipid (lysophosphatidylethanolamine)		1.853	0.0880547082197618	0.0880547082197618	1.08090180709062	unchanged
Meta_549	Thr-HDCA	Lipid	Bile Acid, amino acid conjungate		-0.919333333	0.10581878742328	0.10581878742328	0.940163173805873	unchanged
Meta_55	L-Threonine	Amino Acid	Glycine, Serine and Threonine Metabolism	HMDB0000167	-0.227333333	0.183445852815774	0.183445852815774	0.881063784414702	unchanged
Meta_550	LPE 18:2_sn1	Lipid	Glycerophospholipid (lysophosphatidylethanolamine)	HMDB0011507	0.962333333333333	0.125961080266401	0.125961080266401	0.986051210237676	unchanged
Meta_557	LPG 16:0	Lipid	Glycerophospholipid (lysophosphatidylglycerol)	HMDB0240601	-0.091666667	0.437718994047699	0.437718994047699	0.527183109372888	unchanged
Meta_565	LPE 20:5_sn2	Lipid	Glycerophospholipid (lysophosphatidylethanolamine)	HMDB0011519	0.464333333333334	0.335700939775478	0.335700939775478	0.62303780227704	unchanged
Meta_568	ATP	Nucleotide	Purine Metabolism, Adenine containing	HMDB0000538	-1.830333333	0.0905410571794103	0.0905410571794103	1.00760439746128	unchanged
Meta_569	LPE 20:2_sn2	Lipid	Glycerophospholipid (lysophosphatidylethanolamine)	HMDB0011483	2.01866666666667	0.0550350711764414	0.0550350711764414	1.03544678693834	unchanged
Meta_578	GTP	Nucleotide	Purine Metabolism, Guanine containing	HMDB0001273	-0.245333333	0.362566678237672	0.362566678237672	0.636421685990017	unchanged
Meta_579	LPS 18:1	Lipid	Glycerophospholipid (lysophosphatidylserine)	HMDB0240603	0.364666666666668	0.119409823791527	0.119409823791527	0.959586430516954	unchanged
Meta_58	Phenylacetaldehyde	Amino Acid	Phenylalanine Metabolism	HMDB0006236	0.248000000000001	0.160715618942414	0.160715618942414	0.852654403471084	unchanged
Meta_59	Benzoic acid	Xenobiotics	Benzoate Metabolism	HMDB0001870	0.455333333333332	0.0845236323241385	0.0845236323241385	1.08270866430673	unchanged
Meta_593	UDP-Glucose	Carbohydrate	Amino sugar and Nucleotide sugar Metabolism	HMDB0000286	-0.521333333	0.123749479096452	0.123749479096452	0.950614399834468	unchanged
Meta_599	Uridine 5'-Diphosphoglucuronic Acid	Carbohydrate	Amino sugar and Nucleotide sugar Metabolism	HMDB0000935	0.835000000000001	0.074056798516971	0.074056798516971	1.10263962928575	unchanged
Meta_60	Nicotinamide	Cofactors and Vitamins	Nicotinate and Nicotinamide Metabolism	HMDB0001406	-0.539	0.0531163555857949	0.0531163555857949	1.03723969451171	unchanged
Meta_601	ADP-Glucose	Nucleotide	Purine Metabolism, Adenine containing	HMDB0006557	-0.961333333	0.405206312757057	0.405206312757057	0.55753191975995	unchanged
Meta_606	Guanosine 5'-Diphosphate-D-Mannose	Carbohydrate	Amino sugar and Nucleotide sugar Metabolism	HMDB0001163	0.871333333333334	0.280361140067075	0.280361140067075	0.690577158072189	unchanged
Meta_61	Taurine	Amino Acid	Methionine, Cysteine, SAM and Taurine Metabolism	HMDB0000251	0.579666666666666	0.193258883673081	0.193258883673081	0.890562967815382	unchanged
Meta_610	Glutathione Disulfide (GSSG)	Amino Acid	Glutathione Metabolism	HMDB0003337	0.614333333333331	0.107570886141151	0.107570886141151	0.995340483562818	unchanged
Meta_611	LPI 20:4	Lipid	Glycerophospholipid (lysophosphatidylinositiol)	HMDB0061690	2.02333333333333	0.0594012571360716	0.0594012571360716	1.09510771051708	unchanged
Meta_613	SM 32:2;O2	Lipid	Sphingolipid Metabolism		-0.689333333	0.0856558610842681	0.0856558610842681	0.97089819920931	unchanged
Meta_614	SM 32:1;O2	Lipid	Sphingolipid Metabolism		0.419999999999998	0.107370923769942	0.107370923769942	0.969487343492392	unchanged
Meta_615	SM 33:1;O2|SM 17:1;O2/16:0	Lipid	Sphingolipid Metabolism		0.442	0.194962254189216	0.194962254189216	0.866221150753501	unchanged
Meta_617	SM 34:2;O2|SM 18:2;O2/16:0	Lipid	Sphingolipid Metabolism	HMDB0240638	0.135666666666669	0.593588627244688	0.593588627244688	0.366023173156995	unchanged
Meta_618	SM 34:1;O2|SM 18:1;O2/16:0	Lipid	Sphingolipid Metabolism	HMDB0010169	0.238333333333333	0.146530304807698	0.146530304807698	0.927620672172289	unchanged
Meta_619	SM 34:0;O2	Lipid	Sphingolipid Metabolism		0.637666666666664	0.0523831047460818	0.0523831047460818	1.06036792233293	unchanged

Meta_62	Picolinic Acid	Amino Acid	Tryptophan Metabolism	HMDB0002243	0.0820000000000007	0.66816241741209	0.66816241741209	0.295912478864314	unchanged
Meta_621	PE 34:3|PE 16:1_18:2	Lipid	Glycerophospholipid (phosphatidylethanolamine)	HMDB0008961	-0.545	0.152855165746758	0.152855165746758	0.867641812278342	unchanged
Meta_622	LNAPE 18:2/N-16:0	Lipid	Glycerophospholipid (N-acyl-lysophosphatidylethanolamines)		-0.588	0.49360250061286	0.49360250061286	0.460032761121834	unchanged
Meta_627	PC 32:3	Lipid	Glycerophospholipid (phosphatidylcholine)		-0.234333333	0.591235799913122	0.591235799913122	0.36859015032843	unchanged
Meta_629	PC 32:2|PC 14:0_18:2	Lipid	Glycerophospholipid (phosphatidylcholine)	HMDB0007874	-0.126333333	0.705351597192579	0.705351597192579	0.261729804976189	unchanged
Meta_63	5-Methylcytosine	Nucleotide	Pyrimidine Metabolism, Cytidine containing	HMDB0002894	-0.864666667	0.0739869727186681	0.0739869727186681	1.05647773167187	unchanged
Meta_631	PC 32:1|PC 16:0_16:1	Lipid	Glycerophospholipid (phosphatidylcholine)	HMDB0007969	-0.105333333	0.336615116826037	0.336615116826037	0.651729205435593	unchanged
Meta_633	PE 36:5	Lipid	Glycerophospholipid (phosphatidylethanolamine)		-0.214	0.462704481161448	0.462704481161448	0.483447125017238	unchanged
Meta_634	PE 36:4|PE 18:2_18:2	Lipid	Glycerophospholipid (phosphatidylethanolamine)	HMDB0009093	0.0429999999999993	0.816834423628714	0.816834423628714	0.168795360199266	unchanged
Meta_635	PE 36:4|PE 16:0_20:4	Lipid	Glycerophospholipid (phosphatidylethanolamine)	HMDB0008937	-0.152	0.202026125044356	0.202026125044356	0.862344687800331	unchanged
Meta_637	LNAPE 18:2/N-18:0	Lipid	Glycerophospholipid (N-acyl-lysophosphatidylethanolamines)		0.24	0.560245351178978	0.560245351178978	0.39087027508583	unchanged
Meta_638	PE 36:2|PE 18:0_18:2	Lipid	Glycerophospholipid (phosphatidylethanolamine)	HMDB0008994	-0.479	0.0570289156251261	0.0570289156251261	1.08840117695352	unchanged
Meta_639	PC 33:2|PC 15:0_18:2	Lipid	Glycerophospholipid (phosphatidylcholine)	HMDB0007940	0.234333333333332	0.466841640409282	0.466841640409282	0.504025094187031	unchanged
Meta_64	1-Methylcytosine	Nucleotide	Pyrimidine Metabolism, Cytidine containing	HMDB0243939	0.512999999999998	0.163950250908217	0.163950250908217	0.897428241244504	unchanged
Meta_640	LNAPE 18:1/N-18:0	Lipid	Glycerophospholipid (N-acyl-lysophosphatidylethanolamines)		-0.267666667	0.214826516917158	0.214826516917158	0.843696152635525	unchanged
Meta_641	PC 33:1	Lipid	Glycerophospholipid (phosphatidylcholine)		0.280999999999999	0.282597028834049	0.282597028834049	0.696202560876596	unchanged
Meta_642	PE 36:1	Lipid	Glycerophospholipid (phosphatidylethanolamine)		1.20966666666667	0.313586015429831	0.313586015429831	0.712217453354224	unchanged
Meta_643	PC 33:0_2	Lipid	Glycerophospholipid (phosphatidylcholine)		-0.052333333	0.84449139930791	0.84449139930791	0.134265461793594	unchanged
Meta_644	PC 33:0	Lipid	Glycerophospholipid (phosphatidylcholine)		0.830333333333334	0.532492193930664	0.532492193930664	0.444319183518275	unchanged
Meta_646	PC 34:3|PC 16:1_18:2	Lipid	Glycerophospholipid (phosphatidylcholine)	HMDB0008006	0.534333333333336	0.168993800520062	0.168993800520062	0.88081152342386	unchanged
Meta_647	PC 34:2|PC 16:1_18:1	Lipid	Glycerophospholipid (phosphatidylcholine)	HMDB0008005	-0.012666667	0.895291601205589	0.895291601205589	0.0866194798601735	unchanged
Meta_649	PC 34:1|PC 16:0_18:1	Lipid	Glycerophospholipid (phosphatidylcholine)	HMDB0007972	0.182333333333332	0.182797781807359	0.182797781807359	0.813381866683838	unchanged
Meta_65	4-Imidazoleacetic Acid	Amino Acid	Histidine Metabolism	HMDB0002024	-0.641333333	0.320525682656716	0.320525682656716	0.681656625483478	unchanged
Meta_650	PC 34:0	Lipid	Glycerophospholipid (phosphatidylcholine)		2.252	0.219252520127658	0.219252520127658	0.832136390021555	unchanged
Meta_651	PE 38:6|PE 18:2_20:4	Lipid	Glycerophospholipid (phosphatidylethanolamine)	HMDB0009102	-0.556333333	0.107619474844215	0.107619474844215	0.929047157405881	unchanged
Meta_652	PE 38:5|PE 18:1_20:4	Lipid	Glycerophospholipid (phosphatidylethanolamine)	HMDB0009069	0.075333333333333	0.561483155925652	0.561483155925652	0.398354399408172	unchanged
Meta_654	PE 38:4|PE 18:0_20:4	Lipid	Glycerophospholipid (phosphatidylethanolamine)	HMDB0009003	-0.195	0.504286899195903	0.504286899195903	0.456300946576099	unchanged
Meta_656	PC 35:3	Lipid	Glycerophospholipid (phosphatidylcholine)		0.679	0.122276590334017	0.122276590334017	0.953168272241656	unchanged
Meta_657	LNAPE 18:2/N-20:0	Lipid	Glycerophospholipid (N-acyl-lysophosphatidylethanolamines)		1.10033333333333	0.163945315820162	0.163945315820162	0.945043425197569	unchanged
Meta_659	PC 36:5|PC 16:1_20:4	Lipid	Glycerophospholipid (phosphatidylcholine)	HMDB0008015	0.443666666666669	0.244585635073979	0.244585635073979	0.724177454188359	unchanged
Meta_662	Flavin Adenine Dinucleotide	Cofactors and Vitamins	Vitamin B2 Metabolism	HMDB0001248	-0.157333333	0.343854842694325	0.343854842694325	0.650747586491957	unchanged
Meta_663	PC 36:3|PC 16:0_20:3	Lipid	Glycerophospholipid (phosphatidylcholine)	HMDB0007981	-0.767333333	0.520725545204057	0.520725545204057	0.456337982691399	unchanged
Meta_664	SM 40:2;O2|SM 18:2;O2/22:0	Lipid	Sphingolipid Metabolism	HMDB0240633	1.31766666666666	0.0953497441885905	0.0953497441885905	1.0213893065145	unchanged
Meta_665	PC 36:2|PC 18:0_18:2	Lipid	Glycerophospholipid (phosphatidylcholine)	HMDB0008039	0.242000000000001	0.111979819395954	0.111979819395954	0.920382745706823	unchanged
Meta_668	PE 40:7|PE 18:1_22:6	Lipid	Glycerophospholipid (phosphatidylethanolamine)	HMDB0009078	-0.445	0.246865367367911	0.246865367367911	0.759370965143476	unchanged
Meta_67	Dl-Coniine	Xenobiotics	Natural Product/Food/Plant	HMDB0030285	0.390666666666668	0.716170545655594	0.716170545655594	0.249289023550372	unchanged
Meta_670	PC 35:2	Lipid	Glycerophospholipid (phosphatidylcholine)		-0.031	0.895479094047654	0.895479094047654	0.0862608049169679	unchanged
Meta_671	PC 37:2|PC 19:0_18:2	Lipid	Glycerophospholipid (phosphatidylcholine)	HMDB0256163	0.464666666666666	0.755633755583368	0.755633755583368	0.218172785848499	unchanged
Meta_672	PC 38:7_2	Lipid	Glycerophospholipid (phosphatidylcholine)		0.407666666666666	0.729275260620026	0.729275260620026	0.236828218762493	unchanged
Meta_673	PC 38:6|PC 16:0_22:6	Lipid	Glycerophospholipid (phosphatidylcholine)	HMDB0007991	0.870666666666665	0.0661769039923719	0.0661769039923719	1.07775357500166	unchanged
Meta_674	PC 38:6|PC 18:2_20:4	Lipid	Glycerophospholipid (phosphatidylcholine)	HMDB0008147	0.174	0.642062713749815	0.642062713749815	0.324103419168811	unchanged
Meta_676	PC 38:4|PC 18:0_20:4	Lipid	Glycerophospholipid (phosphatidylcholine)	HMDB0008048	1.10466666666667	0.164561131889615	0.164561131889615	0.930147662762287	unchanged
Meta_677	PC 38:3|PC 18:0_20:3	Lipid	Glycerophospholipid (phosphatidylcholine)	HMDB0008047	0.113999999999997	0.8519131147139	0.8519131147139	0.125840468903707	unchanged
Meta_678	PC 38:2	Lipid	Glycerophospholipid (phosphatidylcholine)		-0.722	0.557565181511193	0.557565181511193	0.415311477925664	unchanged
Meta_679	PE 40:5	Lipid	Glycerophospholipid (phosphatidylethanolamine)		0.878666666666666	0.177855190050814	0.177855190050814	0.875331491292708	unchanged
Meta_68	Citraconic Acid	Lipid	Fatty Acid, Dicarboxylate	HMDB0000634	0.00633333333333219	0.98875583859202	0.98875583859202	0.0167390984454755	unchanged
Meta_682	PC 40:8|PC 20:4_20:4	Lipid	Glycerophospholipid (phosphatidylcholine)	HMDB0008443	1.18066666666667	0.117021072661614	0.117021072661614	1.00656270363238	unchanged
Meta_683	PC 40:7|PC 18:1_22:6	Lipid	Glycerophospholipid (phosphatidylcholine)	HMDB0008123	0.515000000000002	0.237886950032114	0.237886950032114	0.78968660482522	unchanged
Meta_685	PC 40:5	Lipid	Glycerophospholipid (phosphatidylcholine)	HMDB0008056	0.462333333333334	0.206354653568297	0.206354653568297	0.830286167813227	unchanged
Meta_686	PC 40:4|PC 18:0_22:4	Lipid	Glycerophospholipid (phosphatidylcholine)	HMDB0008054	3.53566666666667	0.0966085851181684	0.0966085851181684	1.05417970520565	unchanged
Meta_687	Eplerenone	Xenobiotics	Drug	HMDB0014838	0.486333333333333	0.33800315231666	0.33800315231666	0.623018830372774	unchanged
Meta_689	PI 36:4	Lipid	Glycerophospholipid (phosphatidylinositol)	HMDB0009790	1.38033333333333	0.0991958250250554	0.0991958250250554	0.939169028975315	unchanged
Meta_69	Pyroglutamine	Amino Acid	Alanine, Aspartate and Glutamate Metabolism	HMDB0062558	-0.095	0.351328264483106	0.351328264483106	0.605762422006382	unchanged
Meta_7	2-Pyrrolidinone	Xenobiotics	Chemical	HMDB0002039	0.233666666666664	0.599777426240275	0.599777426240275	0.361286582304018	unchanged
Meta_72	L-Pipecolic Acid	Amino Acid	Lysine Metabolism	HMDB0000716	0.0353333333333339	0.869285844475381	0.869285844475381	0.119552884294267	unchanged
Meta_74	Methylsuccinic Acid	Amino Acid	Leucine, Isoleucine and Valine Metabolism	HMDB0001844	-0.506333333	0.0895167705644626	0.0895167705644626	1.0202822636537	unchanged
Meta_76	3-Hydroxyhexanoate	Lipid	Fatty Acid, Monohydroxy	HMDB0061652	-0.989666667	0.0925866580336477	0.0925866580336477	1.02820835830481	unchanged
Meta_77	2-hydroxy-3-methylvalerate	Amino Acid	Leucine, Isoleucine and Valine Metabolism	HMDB0000317	-0.285666667	0.100688301280866	0.100688301280866	0.96037016181495	unchanged
Meta_79	Trans-4-Hydroxyproline	Amino Acid	Urea cycle; Arginine and Proline Metabolism	HMDB0000725	1.26166666666667	0.0879059059722763	0.0879059059722763	0.981352343902455	unchanged
Meta_81	3-Methylindole	Xenobiotics	Natural Product/Food/Plant	HMDB0000466	0.596666666666668	0.0641185135845191	0.0641185135845191	1.04556151491632	unchanged
Meta_84	Malic Acid	Carbohydrate	Citrate cycle (TCA cycle)	HMDB0000156	0.434999999999999	0.367428963398786	0.367428963398786	0.622771509216092	unchanged
Meta_86	Trans-Cinnamaldehyde	Xenobiotics	Natural Product/Food/Plant	HMDB0003441	0.314666666666668	0.302215378599928	0.302215378599928	0.670663333075031	unchanged
Meta_87	cuminyl alcohol	Xenobiotics	Degradation of aromatic compounds	HMDB0031817	0.0116666666666667	0.943842288761938	0.943842288761938	0.0567831038937739	unchanged
Meta_88	Thiaproline	Xenobiotics	Natural Product/Food/Plant	HMDB0258979	-0.715666667	0.266150302344169	0.266150302344169	0.779597983053349	unchanged

Meta_92	CARVEOL	Xenobiotics	Natural Product/Food/Plant	HMDB0036083	-0.085	0.768462055607822	0.768462055607822	0.200480054280831	unchanged
Meta_97	Phenylethanolamine	Xenobiotics	Chemical	HMDB0001065	-0.029	0.93713548903166	0.93713548903166	0.0533847942007738	unchanged
Meta_98	Urocanate	Amino Acid	Histidine Metabolism	HMDB0062562	0.171333333333333	0.743917827451757	0.743917827451757	0.225032020114355	unchanged
Meta_104	Oxoglutaric Acid	Carbohydrate	Citrate cycle (TCA cycle)	HMDB0000208	1.83766666666667	0.00507426373056897	0.00507426373056897	1.20866767249707	up
Meta_109	Acetylcholine	Lipid	Fatty Acid Metabolism (Acyl Choline)	HMDB0000895	0.716333333333331	0.0221266065565337	0.0221266065565337	1.22632354459312	up
Meta_111	2-Hydroxyglutaric Acid	Carbohydrate	Citrate cycle (TCA cycle)	HMDB0059655	3.33	0.0115427898012081	0.0115427898012081	1.22732527102856	up
Meta_113	L-Glutamine	Amino Acid	Alanine, Aspartate and Glutamate Metabolism	HMDB0000641	2.492	0.000775270134585988	0.000775270134585988	1.26599194821197	up
Meta_114	L-Lysine	Amino Acid	Lysine Metabolism	HMDB0000182	0.827000000000002	0.0069384420814516	0.0069384420814516	1.26103489388878	up
Meta_117	Hydroxyasparagine	Amino Acid	Alanine, Aspartate and Glutamate Metabolism	HMDB0032332	0.654999999999999	0.0169824089928284	0.0169824089928284	1.14447666034039	up
Meta_118	Cinnamic Acid	Amino Acid	Phenylalanine Metabolism	HMDB0000567	0.687333333333335	0.0306455426692605	0.0306455426692605	1.1452355750302	up
Meta_12	Lactic Acid	Carbohydrate	Glycolysis, Gluconeogenesis, and Pyruvate Metabolism	HMDB0000190	0.102	0.0334808743522189	0.0334808743522189	1.10823038055062	up
Meta_120	L-Methionine	Amino Acid	Methionine, Cysteine, SAM and Taurine Metabolism	HMDB0000696	0.604666666666667	0.0116142561565276	0.0116142561565276	1.23563373670666	up
Meta_122	Cysteine sulfinic Acid	Amino Acid	Methionine, Cysteine, SAM and Taurine Metabolism	HMDB0000996	2.375	0.000337981469894375	0.000337981469894375	1.27254956501116	up
Meta_127	Dihydroorotic acid	Nucleotide	Pyrimidine Metabolism, Orotic acid containing	HMDB0003349	6.30066666666667	0.018806431318511	0.018806431318511	1.2356584803726	up
Meta_135	1,5-Naphthalenediamine	Xenobiotics	Chemical	HMDB0244231	0.655333333333331	0.0402814924792979	0.0402814924792979	1.08631673265885	up
Meta_147	Carnitine	Lipid	Fatty Acid Metabolism (Acyl Carnitine)	HMDB0000062	1.15266666666667	0.00159170915068611	0.00159170915068611	1.26569300190537	up
Meta_148	5-hydroxylysine	Amino Acid	Lysine Metabolism	HMDB0000450	1.20433333333333	0.000840508218949514	0.000840508218949514	1.26278805890465	up
Meta_151	P-Coumaric Acid	Amino Acid	Tyrosine Metabolism	HMDB0002035	0.518333333333331	0.0282122156449506	0.0282122156449506	1.16518278942122	up
Meta_157	L-Phenylalanine	Amino Acid	Phenylalanine Metabolism	HMDB0000159	0.688666666666666	0.0378232398064207	0.0378232398064207	1.11462250903551	up
Meta_164	Pyridoxamine	Cofactors and Vitamins	Vitamin B6 Metabolism	HMDB0001431	0.984	0.00532864517407295	0.00532864517407295	1.26633978992023	up
Meta_165	Pyridoxine	Cofactors and Vitamins	Vitamin B6 Metabolism	HMDB0000239	1.09766666666667	0.0162363583034667	0.0162363583034667	1.24169483292528	up
Meta_166	Glycerol 3-Phosphate	Lipid	Glycerolipid and Phospholipid Metabolism	HMDB0000126	1.36833333333333	0.01221649495408	0.01221649495408	1.1727084213791	up
Meta_171	N-Acetyl-L-Aspartic Acid	Amino Acid	Alanine, Aspartate and Glutamate Metabolism	HMDB0000812	1.854	0.00362425558403325	0.00362425558403325	1.22350086274038	up
Meta_172	N-Carbamoyl-L-Aspartate	Nucleotide	Purine and Pyrimidine Metabolism	HMDB0000828	3.52466666666667	0.000162226432742622	0.000162226432742622	1.27367143024662	up
Meta_176	L-Arginine	Amino Acid	Urea cycle; Arginine and Proline Metabolism	HMDB0000517	0.664000000000002	0.00249948569551178	0.00249948569551178	1.24015003077344	up
Meta_178	Guanidinosuccinic Acid	Amino Acid	Urea cycle; Arginine and Proline Metabolism	HMDB0003157	0.715666666666667	0.0441754590043157	0.0441754590043157	1.16968607975961	up
Meta_181	Cysteinylglycine	Amino Acid	Glutathione Metabolism	HMDB0000078	0.802	0.0357835746805734	0.0357835746805734	1.11860803785195	up
Meta_182	Glucose	Carbohydrate	Glycolysis, Gluconeogenesis, and Pyruvate Metabolism	HMDB0000122	0.976333333333333	0.013342049658197	0.013342049658197	1.22608424778397	up
Meta_183	Mannose	Carbohydrate	Polysaccharides and Oligosaccharides	HMDB0000169	1.03	0.00360224457325669	0.00360224457325669	1.22387963037864	up
Meta_185	Hydroxyphenyllactic Acid	Amino Acid	Tyrosine Metabolism	HMDB0000755	1.12	0.0114319439845015	0.0114319439845015	1.19541234426304	up
Meta_189	L-Tyrosine	Amino Acid	Tyrosine Metabolism	HMDB0000158	0.537999999999997	0.0378945171845422	0.0378945171845422	1.15437063127506	up
Meta_191	Choline Phosphate	Lipid	Glycerolipid and Phospholipid Metabolism	HMDB0001565	2.43766666666667	0.00131318565963027	0.00131318565963027	1.25872276150942	up
Meta_2	Pyrrolidine	Xenobiotics	Natural Product/Food/Plant	HMDB0031641	0.532666666666668	0.0458971713844361	0.0458971713844361	1.14048929310861	up
Meta_200	Glutarylglycine	Lipid	Fatty Acid Metabolism (Acyl glycines)	HMDB0000590	0.754999999999999	0.00883787826564133	0.00883787826564133	1.18494160235556	up
Meta_203	N-Acetylglutamine	Amino Acid	Alanine, Aspartate and Glutamate Metabolism	HMDB0006029	1.119	0.0464307342357645	0.0464307342357645	1.08421838781497	up
Meta_207	N6-Acetyllysine	Amino Acid	Lysine Metabolism	HMDB0000206	1.07133333333333	0.00200559228165847	0.00200559228165847	1.27778791366994	up
Meta_210	N-Acetylglutamate	Amino Acid	Alanine, Aspartate and Glutamate Metabolism	HMDB0001138	0.568333333333333	0.0220018781348826	0.0220018781348826	1.14826292949904	up
Meta_219	D-Gluconic Acid	Carbohydrate	Pentose phosphate pathway	HMDB0000625	1.67733333333334	0.00489783614263348	0.00489783614263348	1.230592916	up
Meta_221	3-Methoxy-4-Hydroxymandelate	Xenobiotics	Natural Product/Food/Plant	HMDB0000291	1.976	0.00484008514720832	0.00484008514720832	1.2440096116157	up
Meta_233	N2-Acetyl,N6-Methyllysine	Amino Acid	Lysine Metabolism	HMDB0242186	0.930333333333333	0.0408902085907367	0.0408902085907367	1.16227397940928	up
Meta_24	2-Hydroxybutyric Acid	Carbohydrate	Propanoate Metabolism	HMDB0000008	0.526999999999999	0.0492012757365633	0.0492012757365633	1.08359930695749	up
Meta_243	N-Acetylphenylalanine	Amino Acid	Phenylalanine Metabolism	HMDB0000512	1.948	0.00201259012153757	0.00201259012153757	1.26733056438528	up
Meta_251	Glycerophosphoethanolamine	Lipid	Glycerolipid and Phospholipid Metabolism	HMDB0000114	1.76	0.0364977461441974	0.0364977461441974	1.14269258563403	up
Meta_255	Tyramine O-Sulfate	Amino Acid	Tyrosine Metabolism	HMDB0006409	1.448	0.0488459101672422	0.0488459101672422	1.09835228706589	up
Meta_263	Pantothenate	Cofactors and Vitamins	Pantothenate and CoA Metabolism	HMDB0000210	1.361	0.00275346029167124	0.00275346029167124	1.24057754664852	up
Meta_272	Myristoleic Acid (C14:1n5)	Lipid	Long Chain Fatty Acid	HMDB0002000	0.894	0.0238081892396477	0.0238081892396477	1.13157777060009	up
Meta_276	Myristic Acid (C14:0)	Lipid	Long Chain Fatty Acid	HMDB0000806	0.524999999999999	0.00187962166772804	0.00187962166772804	1.24528319516367	up
Meta_278	Pro-Hydroxy-Pro	Amino Acid	Urea cycle; Arginine and Proline Metabolism	HMDB0006695	1.64233333333333	0.00673717566113161	0.00673717566113161	1.22696924072033	up
Meta_28	Choline	Lipid	Glycerolipid and Phospholipid Metabolism	HMDB0000097	0.334666666666667	0.0302434269275689	0.0302434269275689	1.1963164644042	up
Meta_280	L-Prolyl-L-isoleucine	Peptide	Dipeptide		1.09933333333333	0.0226208857057551	0.0226208857057551	1.18303518602298	up
Meta_299	Lys-C6:1	Lipid	Fatty Acid Metabolism (Fatty amides)		1.87266666666667	0.0030076630346413	0.0030076630346413	1.25720232083918	up
Meta_315	Gamma-Glutamylvaline	Peptide	Gamma-glutamyl Amino Acid	HMDB0011172	1.53066666666667	0.00685551709099311	0.00685551709099311	1.226597973	up
Meta_32	BENZYL ALCOHOL	Xenobiotics	Degradation of aromatic compounds	HMDB0003119	0.441333333333333	0.0332758135744052	0.0332758135744052	1.16257445731425	up
Meta_321	Gamma-Glutamyl-Cysteine	Peptide	Gamma-glutamyl Amino Acid	HMDB0001049	2.87066666666667	0.000110795488423446	0.000110795488423446	1.28403951436061	up
Meta_322	Hexadecadienoate (16:2n6)	Lipid	Long Chain Fatty Acid	HMDB0000477	1.16133333333333	0.0339733712175701	0.0339733712175701	1.09532437216073	up
Meta_326	Palmitic Acid (C16:0)	Lipid	Long Chain Fatty Acid	HMDB0000220	0.317000000000004	0.00456691124704161	0.00456691124704161	1.24588454038487	up
Meta_330	1-Methylpseudouridine	Xenobiotics	Natural Product/Food/Plant		0.880666666666667	0.00399772736567524	0.00399772736567524	1.23444501755669	up
Meta_333	Glycerophosphorylcholine	Lipid	Glycerolipid and Phospholipid Metabolism	HMDB0000086	1.932	0.00771679008828334	0.00771679008828334	1.26255860898549	up
Meta_334	2-Methylcytidine	Nucleotide	Pyrimidine Metabolism, Cytidine containing	HMDB0242132	2.56966666666667	0.0209269063417267	0.0209269063417267	1.18743300265241	up
Meta_337	5-Methyluridine	Nucleotide	Pyrimidine Metabolism, Uracil containing	HMDB0000884	1.08133333333333	0.0143934810661136	0.0143934810661136	1.20475978313408	up
Meta_339	Hexanoylcarnitine	Lipid	Fatty Acid Metabolism (Acyl Carnitine)	HMDB0000756	1.64833333333333	0.0174828999103017	0.0174828999103017	1.22604874332387	up
Meta_347	Gamma-Glutamylleucine	Peptide	Gamma-glutamyl Amino Acid	HMDB0011171	1.89466666666666	0.0052026815155205	0.0052026815155205	1.24855536332844	up
Meta_348	Gamma-Glutamylisoleucine	Peptide	Gamma-glutamyl Amino Acid	HMDB0011170	1.66966666666667	0.00441717763772737	0.00441717763772737	1.24767090577513	up
Meta_361	10Z-Heptadecenoic Acid (C17:1n7)	Lipid	Long Chain Fatty Acid	HMDB0060038	0.807	0.0118355208026071	0.0118355208026071	1.17158878317534	up

Meta_362	Adenosine	Nucleotide	Purine Metabolism, Adenine containing	HMDB0000050	1.49966666666667	0.000940957581190222	0.000940957581190222	1.25615817013859	up
Meta_368	Heptadecanoic Acid (C17:0)	Lipid	Long Chain Fatty Acid	HMDB0002259	0.183999999999999	0.0421220083915681	0.0421220083915681	1.0660968322105	up
Meta_373	Hydroxyhexanoycarnitine	Lipid	Fatty Acid Metabolism (Acyl Carnitine)	HMDB0013131	2.05266666666667	0.0329067079025139	0.0329067079025139	1.10024942109159	up
Meta_375	Alpha/Gamma-Linolenic Acid (C18:3n3 or n6)	Lipid	Long Chain Fatty Acid	HMDB0001388	0.714333333333334	0.012980276830595	0.012980276830595	1.20198374092943	up
Meta_380	Linoleic Acid (C18:2n6)	Lipid	Long Chain Fatty Acid	HMDB0000673	0.572666666666667	0.018106817475629	0.018106817475629	1.16552277562816	up
Meta_383	Oleic Acid (C18:1)	Lipid	Long Chain Fatty Acid	HMDB0000207	0.783999999999999	0.0306973261119967	0.0306973261119967	1.09997740237185	up
Meta_387	Guanosine	Nucleotide	Purine Metabolism, Guanine containing	HMDB0000133	0.517333333333333	0.0325523669292818	0.0325523669292818	1.1868049523816	up
Meta_395	Sedoheptulose 7-Phosphate	Carbohydrate	Pentose phosphate pathway	HMDB0001068	1.25733333333333	0.00345225657603777	0.00345225657603777	1.27499585769372	up
Meta_402	Gamma-Glutamylphenylalanine	Peptide	Gamma-glutamyl Amino Acid	HMDB0000594	1.982	0.00168059354844132	0.00168059354844132	1.26147371275927	up
Meta_419	Eicosapentaenoic Acid (C20:5n3 EPA)	Lipid	Long Chain Fatty Acid	HMDB0001999	1.01166666666667	0.0487973803792549	0.0487973803792549	1.06001727949557	up
Meta_420	Sphinganine	Lipid	Sphingolipid Metabolism	HMDB0000269	1.10266666666667	0.00153336530398136	0.00153336530398136	1.2687824661388	up
Meta_424	Arachidonic Acid (C20:4n6)	Lipid	Long Chain Fatty Acid	HMDB0001043	2.68466666666667	0.0012709081610913	0.0012709081610913	1.25194378338675	up
Meta_425	Asp-Gly-Leu	Peptide	Small peptide		1.91333333333333	0.0284299259020922	0.0284299259020922	1.12054516744358	up
Meta_426	Eicosatrienoic Acid (C20:3n3)	Lipid	Long Chain Fatty Acid	HMDB0002925	1.74666666666667	0.0172232189149798	0.0172232189149798	1.21481365599887	up
Meta_43	Succinic Acid	Carbohydrate	Citrate cycle (TCA cycle)	HMDB0000254	0.825333333333333	0.0274498838613931	0.0274498838613931	1.10887963224931	up
Meta_430	Glutathione	Amino Acid	Glutathione Metabolism	HMDB0000125	0.852333333333334	0.0121529158500559	0.0121529158500559	1.18464787934549	up
Meta_436	Gamma-Glutamyltyrosine	Peptide	Gamma-glutamyl Amino Acid	HMDB0011741	2.00866666666667	0.0230237562596169	0.0230237562596169	1.16890236464689	up
Meta_445	11-HETE	Unclassified	Unclassified		2.12033333333333	0.00471920731199822	0.00471920731199822	1.21698333710373	up
Meta_449	UMP	Nucleotide	Pyrimidine Metabolism, Uracil containing	HMDB0000288	0.603333333333332	0.0479259032709127	0.0479259032709127	1.04802420325785	up
Meta_455	Docosapentaenoic Acid (C22:5n6 DPA)	Lipid	Long Chain Fatty Acid	HMDB0006528	1.51733333333334	0.0193221676093085	0.0193221676093085	1.15405802151651	up
Meta_456	Glycerophosphoinositol	Lipid	Glycerolipid and Phospholipid Metabolism	HMDB0011649	2.34266666666667	0.000782399534116992	0.000782399534116992	1.26974975440706	up
Meta_457	13,16,19-Docosatrienoic Acid (C22:3n3)	Lipid	Long Chain Fatty Acid	HMDB0002823	1.489	0.0300055127586645	0.0300055127586645	1.11413169769457	up
Meta_463	Erucamide	Lipid	Fatty Acid Metabolism (Fatty Amide)	HMDB0244507	2.331	0.0317893434243676	0.0317893434243676	1.09716647470413	up
Meta_476	GMP	Nucleotide	Purine Metabolism, Guanine containing	HMDB0001397	1.38966666666667	0.000953790145988599	0.000953790145988599	1.25469811012117	up
Meta_477	Xanthosine-5-Phosphate(XMP)	Nucleotide	Purine Metabolism, (Hypo)Xanthine/Inosine containing	HMDB0001554	1.40166666666667	0.0355131232970517	0.0355131232970517	1.12754669895041	up
Meta_479	C-Glycosyltryptophan	Amino Acid	Tryptophan Metabolism	HMDB0240296	1.65333333333333	0.0457138023042379	0.0457138023042379	1.12412841832173	up
Meta_480	Myristoylcarnitine	Lipid	Fatty Acid Metabolism (Acyl Carnitine)	HMDB0005066	2.77533333333333	9.86039879225602e-06	9.86039879225602e-06	1.28237206112407	up
Meta_482	Riboflavin	Cofactors and Vitamins	Vitamin B2 Metabolism	HMDB0000244	0.909000000000001	0.030124602379906	0.030124602379906	1.16517263763646	up
Meta_484	LPA 14:0_sn2	Lipid	Glycerophospholipid (lysophosphatidic acid)	HMDB0114765	3.03866666666667	0.00441149798868315	0.00441149798868315	1.26864949162002	up
Meta_485	Cholest-4,6-Dien-3-One	Lipid	Sterol	HMDB0002394	1.49333333333333	0.0344601794638359	0.0344601794638359	1.18238187466489	up
Meta_49	Aminomalonic Acid	Amino Acid	Other Amino Acid Metabolism	HMDB0001147	0.833666666666668	0.0397971149404326	0.0397971149404326	1.16385179428072	up
Meta_490	dUDP	Nucleotide	Pyrimidine Metabolism, Uracil containing	HMDB0001000	1.883	0.00308185714035411	0.00308185714035411	1.24165497936863	up
Meta_494	Palmitoleylcarnitine	Lipid	Fatty Acid Metabolism (Acyl Carnitine)	HMDB0240782	1.546	2.09968970880736e-05	2.09968970880736e-05	1.28142380209632	up
Meta_495	S-Adenosylmethionine	Amino Acid	Methionine, Cysteine, SAM and Taurine Metabolism	HMDB0001185	1.43866666666667	0.0110938416827345	0.0110938416827345	1.23885262887051	up
Meta_496	Palmitoylcarnitine	Lipid	Fatty Acid Metabolism (Acyl Carnitine)	HMDB0000222	4.082	7.12792615516833e-05	7.12792615516833e-05	1.28452545797529	up
Meta_506	LPA 16:0_sn1	Lipid	Glycerophospholipid (lysophosphatidic acid)	HMDB0007853	2.026	0.0012799729438666	0.0012799729438666	1.2779976143307	up
Meta_507	LPA 16:0_sn2	Lipid	Glycerophospholipid (lysophosphatidic acid)	HMDB0007849	3.783	0.00761161458820548	0.00761161458820548	1.24294672325123	up
Meta_509	N6-Threonylcarbamoyladenosine	Unclassified	Unclassified		1.59833333333333	0.00275044331283931	0.00275044331283931	1.24277910711123	up
Meta_51	Guanidinoacetic acid	Amino Acid	Creatine Metabolism	HMDB0000128	0.655999999999999	0.0352692179964535	0.0352692179964535	1.16283063102579	up
Meta_510	DEHP	Xenobiotics	Chemical	HMDB0249243	1.05633333333333	0.0443791526720315	0.0443791526720315	1.11278018788386	up
Meta_516	LPA 18:3	Lipid	Glycerophospholipid (lysophosphatidic acid)	HMDB0114743	2.913	0.0186186849154615	0.0186186849154615	1.23160978342155	up
Meta_518	LPA 18:1_sn1	Lipid	Glycerophospholipid (lysophosphatidic acid)	HMDB0007855	2.24666666666667	0.0016875446174579	0.0016875446174579	1.26915980638774	up
Meta_519	LPE O-16:1	Lipid	Glycerophospholipid (lysophosphatidylethanolamine)		2.48133333333334	0.000777188819962413	0.000777188819962413	1.26763899544942	up
Meta_520	LPA 18:0_sn2	Lipid	Glycerophospholipid (lysophosphatidic acid)	HMDB0007850	2.678	0.00156252605712021	0.00156252605712021	1.24442357942163	up
Meta_521	LPE P-16:0	Lipid	Glycerophospholipid (lysophosphatidylethanolamine)	HMDB0011152	2.46533333333333	0.000627287990205216	0.000627287990205216	1.26934279549837	up
Meta_522	Folic acid	Cofactors and Vitamins	Folate Metabolism	HMDB0000121	1.17366666666667	0.0128647991367046	0.0128647991367046	1.24535315133547	up
Meta_523	LPE O-16:0	Lipid	Glycerophospholipid (lysophosphatidylethanolamine)		4.35	0.000613875375411502	0.000613875375411502	1.28240988487172	up
Meta_524	GDP	Nucleotide	Purine Metabolism, Guanine containing	HMDB0001201	1.07533333333333	0.0458533035355149	0.0458533035355149	1.13104238529797	up
Meta_525	CDP-Ethanolamine	Lipid	Glycerolipid and Phospholipid Metabolism	HMDB0001564	3.27366666666667	0.00292330179151368	0.00292330179151368	1.24732304488314	up
Meta_526	Glycochenodeoxycholic Acid	Lipid	Bile Acid, glycine conjungate	HMDB0000637	2.467	7.56855055597678e-05	7.56855055597678e-05	1.28239899410025	up
Meta_529	LPE 16:1_sn1	Lipid	Glycerophospholipid (lysophosphatidylethanolamine)	HMDB0011504	0.924666666666665	0.0149700714869122	0.0149700714869122	1.17341355741387	up
Meta_53	L-Valine	Amino Acid	Leucine, Isoleucine and Valine Metabolism	HMDB0000883	0.581	0.0299209301201851	0.0299209301201851	1.17884716027446	up
Meta_530	LPE P-17:0	Lipid	Glycerophospholipid (lysophosphatidylethanolamine)		2.22533333333333	0.00195544612032479	0.00195544612032479	1.24197114337189	up
Meta_532	LPE 16:0_sn1	Lipid	Glycerophospholipid (lysophosphatidylethanolamine)	HMDB0011503	1.285	0.00560314771583239	0.00560314771583239	1.21049903728216	up
Meta_536	LPA 20:2_sn2	Lipid	Glycerophospholipid (lysophosphatidic acid)	HMDB0114758	3.93166666666667	0.000979968144170104	0.000979968144170104	1.27258120042586	up
Meta_537	LPE P-18:2	Lipid	Glycerophospholipid (lysophosphatidylethanolamine)		2.00166666666667	0.0152245310526232	0.0152245310526232	1.15413594526414	up
Meta_540	Glycocholic Acid	Lipid	Bile Acid, glycine conjungate	HMDB0000138	2.406	0.0134677787303131	0.0134677787303131	1.24796834075405	up
Meta_541	LPE P-18:1	Lipid	Glycerophospholipid (lysophosphatidylethanolamine)	HMDB0240599	2.53166666666667	0.000353355440423312	0.000353355440423312	1.26786043386131	up
Meta_543	LPE O-18:1	Lipid	Glycerophospholipid (lysophosphatidylethanolamine)		4.87833333333334	0.00925904999322303	0.00925904999322303	1.25430777470891	up
Meta_544	LPE P-18:0	Lipid	Glycerophospholipid (lysophosphatidylethanolamine)	HMDB0240598	2.63133333333333	0.0070134529166324	0.0070134529166324	1.25021242234746	up
Meta_545	LPC 14:0_sn1	Lipid	Glycerophospholipid (lysophosphatidylcholine)	HMDB0010379	1.71266666666667	0.00145512338825229	0.00145512338825229	1.26415142892634	up
Meta_547	LPE O-18:0	Lipid	Glycerophospholipid (lysophosphatidylethanolamine)		4.32666666666667	0.0268961388442509	0.0268961388442509	1.20275572469402	up
Meta_551	LPE 18:1_sn1	Lipid	Glycerophospholipid (lysophosphatidylethanolamine)	HMDB0011505	1.64	0.00666574697667468	0.00666574697667468	1.2278336749152	up
Meta_554	LPC 15:0_sn1	Lipid	Glycerophospholipid (lysophosphatidylcholine)	HMDB0010381	3.035	0.030162687420079	0.030162687420079	1.20644057012474	up

Meta_555	LPE 18:0_sn1	Lipid	Glycerophospholipid (lysophosphatidylethanolamine)	HMDB0011130	1.99466666666666	0.0070081126646917	0.0070081126646917	1.22278823432994	up
Meta_558	LPE O-20:5	Lipid	Glycerophospholipid (lysophosphatidylethanolamine)		2.459	0.000852591159135863	0.000852591159135863	1.26424911799843	up
Meta_559	LPE O-20:4	Lipid	Glycerophospholipid (lysophosphatidylethanolamine)		2.731	0.00406071570240676	0.00406071570240676	1.25171511491206	up
Meta_561	LPC 16:1_sn1	Lipid	Glycerophospholipid (lysophosphatidylcholine)	HMDB0010383	1.449	0.000785012908059083	0.000785012908059083	1.26715237169548	up
Meta_562	LPS 16:0	Lipid	Glycerophospholipid (lysophosphatidylserine)	HMDB0240605	1.303	0.000557137458690145	0.000557137458690145	1.25902810627785	up
Meta_563	LPC 16:0_sn1	Lipid	Glycerophospholipid (lysophosphatidylcholine)	HMDB0010382	2.164	0.00124314998793699	0.00124314998793699	1.2688306627065	up
Meta_564	LPE 20:5_sn1	Lipid	Glycerophospholipid (lysophosphatidylethanolamine)	HMDB0011519	0.646333333333333	0.0440171772992654	0.0440171772992654	1.1001169002962	up
Meta_566	LPE 20:4_sn2	Lipid	Glycerophospholipid (lysophosphatidylethanolamine)	HMDB0011487	1.16466666666667	0.0105594904135421	0.0105594904135421	1.18362775934037	up
Meta_571	LPE 20:1_sn1	Lipid	Glycerophospholipid (lysophosphatidylethanolamine)	HMDB0011512	1.72266666666667	0.0113194925392985	0.0113194925392985	1.21307810559613	up
Meta_572	LPC 17:1_(1)	Lipid	Glycerophospholipid (lysophosphatidylcholine)		1.06466666666667	0.0263073720108537	0.0263073720108537	1.19895919238321	up
Meta_573	LPG 18:1	Lipid	Glycerophospholipid (lysophosphatidylglycerol)	HMDB0240602	1.95666666666667	0.00196975988096922	0.00196975988096922	1.24628448092135	up
Meta_574	LPC 17:0_(2)	Lipid	Glycerophospholipid (lysophosphatidylcholine)	HMDB0012108	2.444	0.00804611076023014	0.00804611076023014	1.25342024731081	up
Meta_575	LPG 18:0	Lipid	Glycerophospholipid (lysophosphatidylglycerol)		2.141	0.000211535158267272	0.000211535158267272	1.27577670524578	up
Meta_576	LPC 18:3	Lipid	Glycerophospholipid (lysophosphatidylcholine)	HMDB0010387	2.333	0.0196616662845085	0.0196616662845085	1.14419409654153	up
Meta_577	LPC 18:2_sn2	Lipid	Glycerophospholipid (lysophosphatidylcholine)	HMDB0061700	1.268	0.0123516149826047	0.0123516149826047	1.25072808109478	up
Meta_580	LPC 18:1_sn1	Lipid	Glycerophospholipid (lysophosphatidylcholine)	HMDB0002815	2.35766666666667	0.000133757672589919	0.000133757672589919	1.27350168471648	up
Meta_581	LPS 18:0	Lipid	Glycerophospholipid (lysophosphatidylserine)	HMDB0240606	2.50866666666667	0.00483745557413528	0.00483745557413528	1.26737100627256	up
Meta_582	LPC 18:0_sn1	Lipid	Glycerophospholipid (lysophosphatidylcholine)	HMDB0010384	3.19666666666667	0.000394058631089122	0.000394058631089122	1.27121129926415	up
Meta_583	LPE 22:6_sn2	Lipid	Glycerophospholipid (lysophosphatidylethanolamine)	HMDB0011496	1.75366666666667	0.00867384811451938	0.00867384811451938	1.2346743520798	up
Meta_584	LPE 22:5_(1)	Lipid	Glycerophospholipid (lysophosphatidylethanolamine)	HMDB0011524	1.51066666666667	0.024166850134057	0.024166850134057	1.19954489087475	up
Meta_585	LPE 22:4_sn1	Lipid	Glycerophospholipid (lysophosphatidylethanolamine)	HMDB0011523	1.86133333333333	0.00523493426932037	0.00523493426932037	1.22841670962957	up
Meta_586	LPC 19:1_sn2	Lipid	Glycerophospholipid (lysophosphatidylcholine)		3.599	0.00275240157438989	0.00275240157438989	1.25543040356154	up
Meta_587	LPC 20:4_sn2	Lipid	Glycerophospholipid (lysophosphatidylcholine)	HMDB0061699	2.39533333333334	0.00161626095873468	0.00161626095873468	1.24962350069357	up
Meta_589	LPC 20:3_sn1	Lipid	Glycerophospholipid (lysophosphatidylcholine)	HMDB0010393	2.746	0.00694202426582561	0.00694202426582561	1.24297612491058	up
Meta_590	LPC 20:2_sn1	Lipid	Glycerophospholipid (lysophosphatidylcholine)	HMDB0010392	3.732	0.00441280855897457	0.00441280855897457	1.24926883973599	up
Meta_591	LPC 20:1_sn1	Lipid	Glycerophospholipid (lysophosphatidylcholine)	HMDB0010391	2.55066666666667	0.000528116180836765	0.000528116180836765	1.2643233197409	up
Meta_592	LPC 20:0_sn1	Lipid	Glycerophospholipid (lysophosphatidylcholine)	HMDB0010390	1.596	0.0257843892236956	0.0257843892236956	1.12013964100109	up
Meta_595	LPC 22:5_(1)	Lipid	Glycerophospholipid (lysophosphatidylcholine)	HMDB0010402	3.27733333333334	0.0137268479802073	0.0137268479802073	1.21905826143318	up
Meta_596	LPI 16:0	Lipid	Glycerophospholipid (lysophosphatidylinositiol)	HMDB0061695	3.24266666666667	0.0198293375457203	0.0198293375457203	1.20938447821866	up
Meta_597	LPC 22:4_sn2	Lipid	Glycerophospholipid (lysophosphatidylcholine)	HMDB0010401	3.136	0.00369413491046376	0.00369413491046376	1.2517822	up
Meta_598	LPC 22:1	Lipid	Glycerophospholipid (lysophosphatidylcholine)	HMDB0010399	2.34333333333333	0.00065707032077312	0.00065707032077312	1.2591322772212	up
Meta_6	Dimethyl Sulfoxide	Xenobiotics	Chemical	HMDB0002151	8.92833333333334	8.47574591618068e-06	8.47574591618068e-06	1.28282681672186	up
Meta_603	LPC 24:6	Unclassified	Unclassified		1.01833333333333	0.0329180091925488	0.0329180091925488	1.15739900357726	up
Meta_604	LPI 18:1	Lipid	Glycerophospholipid (lysophosphatidylinositiol)	HMDB0061693	1.644	0.00926561574641114	0.00926561574641114	1.2534654418113	up
Meta_605	LPI 18:0	Lipid	Glycerophospholipid (lysophosphatidylinositiol)	HMDB0240261	3.90466666666667	0.00390626588522745	0.00390626588522745	1.26247225470929	up
Meta_608	LPC 24:1	Lipid	Glycerophospholipid (lysophosphatidylcholine)	HMDB0010406	2.34166666666667	0.00132527256475777	0.00132527256475777	1.25006189546724	up
Meta_624	SM 35:1;O2	Lipid	Sphingolipid Metabolism		0.641	0.0125305078123834	0.0125305078123834	1.16937262299157	up
Meta_628	SM 36:2;O2|SM 18:2;O2/18:0	Lipid	Sphingolipid Metabolism	HMDB0240631	1.03166666666667	0.00161190117912774	0.00161190117912774	1.25483732929478	up
Meta_630	SM 36:1;O2|SM 18:1;O2/18:0	Lipid	Sphingolipid Metabolism		0.643333333333334	0.0328287225497588	0.0328287225497588	1.10286458138989	up
Meta_645	PC 34:4	Lipid	Glycerophospholipid (phosphatidylcholine)		0.82	0.028939976920393	0.028939976920393	1.13771227874539	up
Meta_653	PC 35:4|PC 15:0_20:4	Lipid	Glycerophospholipid (phosphatidylcholine)	HMDB0007949	1.57533333333333	0.0426840583429119	0.0426840583429119	1.16539741062792	up
Meta_655	LNAPE 20:3/N-18:0	Lipid	Glycerophospholipid (N-acyl-lysophosphatidylethanolamines)		0.908333333333335	0.0122709250375234	0.0122709250375234	1.17487934622601	up
Meta_658	PC 35:1	Lipid	Glycerophospholipid (phosphatidylcholine)		0.880666666666663	0.0279746372847138	0.0279746372847138	1.16207832202892	up
Meta_66	Thymine	Nucleotide	Pyrimidine Metabolism, Thymine containing	HMDB0000262	0.908000000000001	0.0165355131850896	0.0165355131850896	1.19395187920447	up
Meta_660	PC 36:4|PC 18:2_18:2	Lipid	Glycerophospholipid (phosphatidylcholine)	HMDB0008138	0.937000000000001	0.048812638308412	0.048812638308412	1.09926333453379	up
Meta_661	PC 36:4|PC 16:0_20:4	Lipid	Glycerophospholipid (phosphatidylcholine)	HMDB0007982	0.670999999999999	0.000496120522684259	0.000496120522684259	1.26166461651145	up
Meta_667	PC 36:1|PC 18:0_18:1	Lipid	Glycerophospholipid (phosphatidylcholine)	HMDB0008038	0.970000000000002	0.0145343306735739	0.0145343306735739	1.20674813173021	up
Meta_675	PC 38:5|PC 18:1_20:4	Lipid	Glycerophospholipid (phosphatidylcholine)	HMDB0008114	0.706666666666667	0.048047748570454	0.048047748570454	1.14350447372107	up
Meta_681	PC 39:5	Lipid	Glycerophospholipid (phosphatidylcholine)		2.058	0.00752214174449564	0.00752214174449564	1.25311658283198	up
Meta_684	PC 40:6|PC 18:0_22:6	Lipid	Glycerophospholipid (phosphatidylcholine)	HMDB0008057	2.39566666666667	0.00588399626655318	0.00588399626655318	1.24127607692856	up
Meta_70	Pyroglutamic Acid	Amino Acid	Glutathione Metabolism	HMDB0000267	0.585666666666665	0.0105019904146693	0.0105019904146693	1.17663308368143	up
Meta_71	Propionylglycine	Lipid	Fatty Acid Metabolism (Acyl glycines)	HMDB0000783	0.460333333333331	0.0434360462012866	0.0434360462012866	1.13390622548503	up
Meta_73	N-Methylproline	Amino Acid	Urea cycle; Arginine and Proline Metabolism	HMDB0094696	0.524666666666665	0.0391202940451055	0.0391202940451055	1.17993877351169	up
Meta_75	Glutarate (C5-DC)	Lipid	Fatty Acid, Dicarboxylate	HMDB0000661	1.011	0.0109029474240036	0.0109029474240036	1.20975673611784	up
Meta_78	N-Acetylputrescine	Amino Acid	Polyamine Metabolism	HMDB0002064	1.918	0.000392620773966106	0.000392620773966106	1.26484887241231	up
Meta_8	Piperidine	Xenobiotics	Natural Product/Food/Plant	HMDB0034301	0.586666666666666	0.0139021225702511	0.0139021225702511	1.19650116804011	up
Meta_80	Creatine	Amino Acid	Creatine Metabolism	HMDB0000064	0.518666666666668	0.00416268086346767	0.00416268086346767	1.21776138291354	up
Meta_82	L-Leucine	Amino Acid	Leucine, Isoleucine and Valine Metabolism	HMDB0000687	0.602333333333334	0.0424669388422852	0.0424669388422852	1.11378507246549	up
Meta_83	L-Isoleucine	Amino Acid	Leucine, Isoleucine and Valine Metabolism	HMDB0000172	0.729333333333333	0.0240129250972687	0.0240129250972687	1.19260163781066	up
Meta_89	Threonic Acid	Cofactors and Vitamins	Ascorbate and Aldarate Metabolism	HMDB0000943	3.13466666666667	0.000477106433852252	0.000477106433852252	1.26777573372438	up
Meta_9	2-Methylpyrrolidine	Xenobiotics	Natural Product/Food/Plant	HMDB0341433	0.726999999999997	0.027281480329971	0.027281480329971	1.14070669953048	up
Meta_91	Phenylacetic Acid	Amino Acid	Phenylalanine Metabolism	HMDB0000209	0.665333333333333	0.0312705015036264	0.0312705015036264	1.09375137452435	up
Meta_93	Adenine	Nucleotide	Purine Metabolism, Adenine containing	HMDB0000034	2.09466666666667	0.00228846272417836	0.00228846272417836	1.25375795168481	up
Meta_95	1-Methylnicotinamide	Cofactors and Vitamins	Nicotinate and Nicotinamide Metabolism	HMDB0000699	1.96533333333333	2.76214045190846e-05	2.76214045190846e-05	1.2810631839526	up
