# Supplementary material for: Autophagic flux inhibition potentiates disulfidptosis susceptibility in human hepatocellular carcinoma
Source: Cell Death Dis. 2026 Aug 11;17(1):948. doi: 10.1038/s41419-026-09135-9 (PMC13745592; doi:10.1038/s41419-026-09135-9)

Original images of western blots

Figure 1A
MHCC97-H ATG7

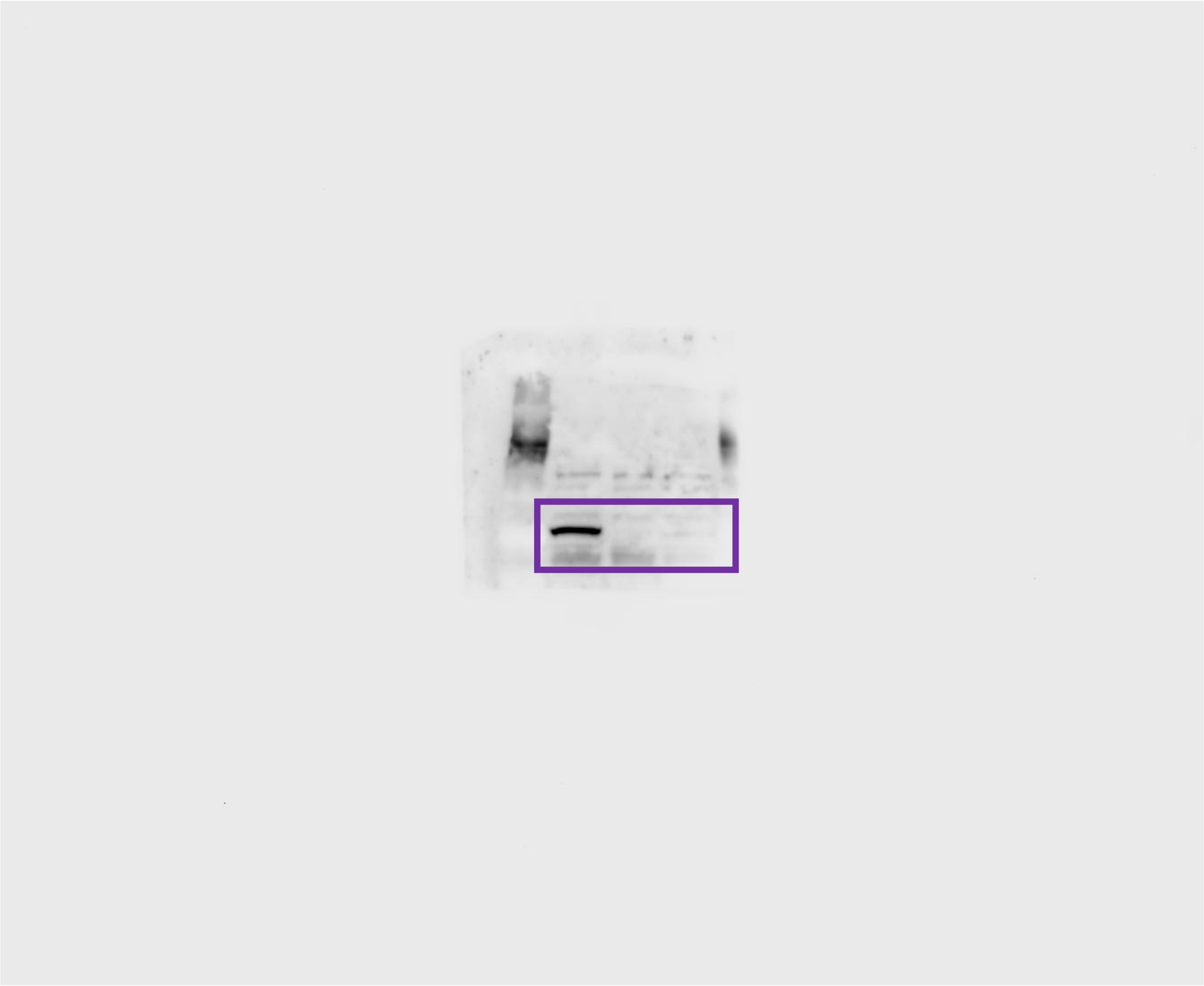

Figure 1A
MHCC97-H p62

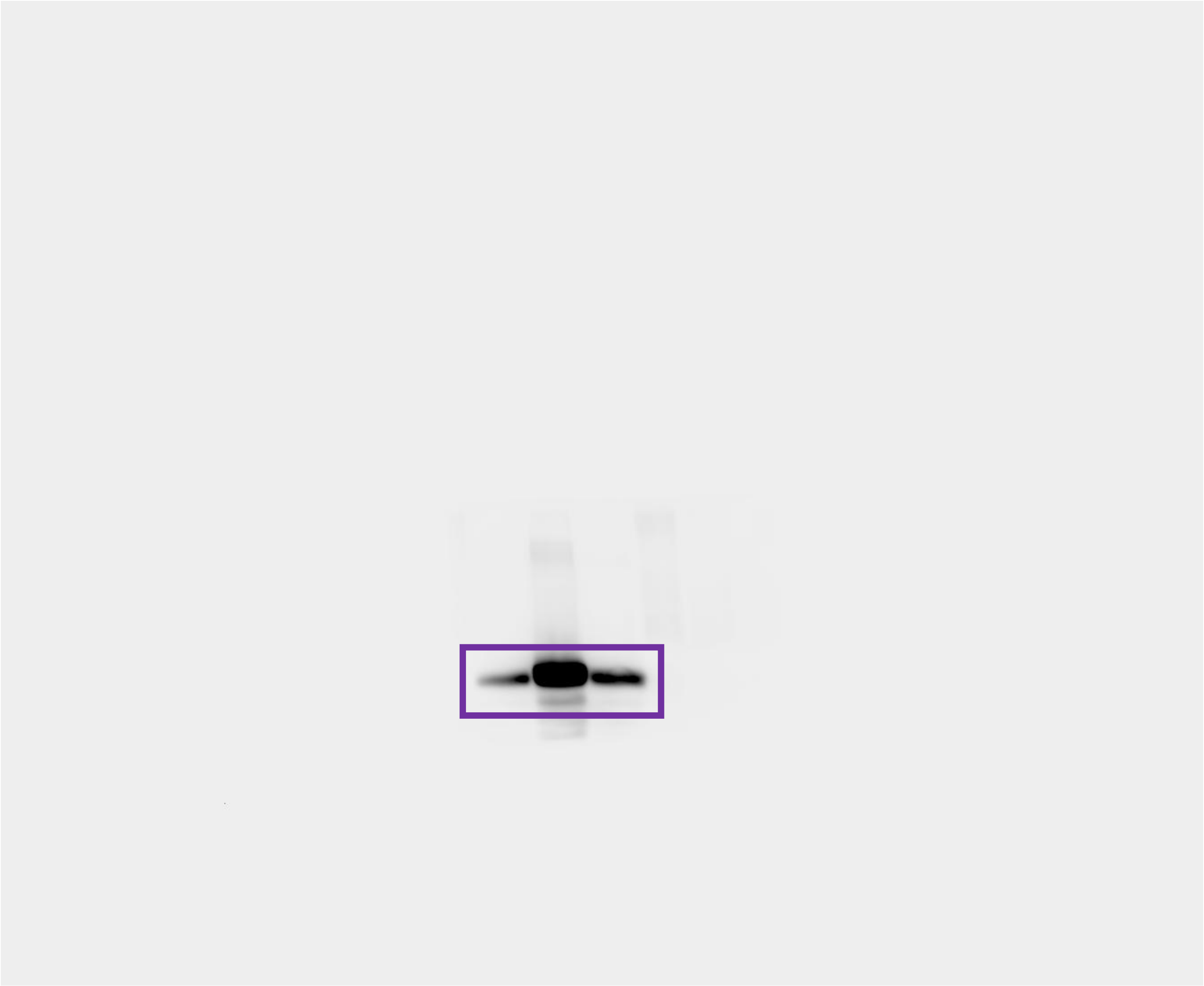

Figure 1A
MHCC97-H BECLIN1

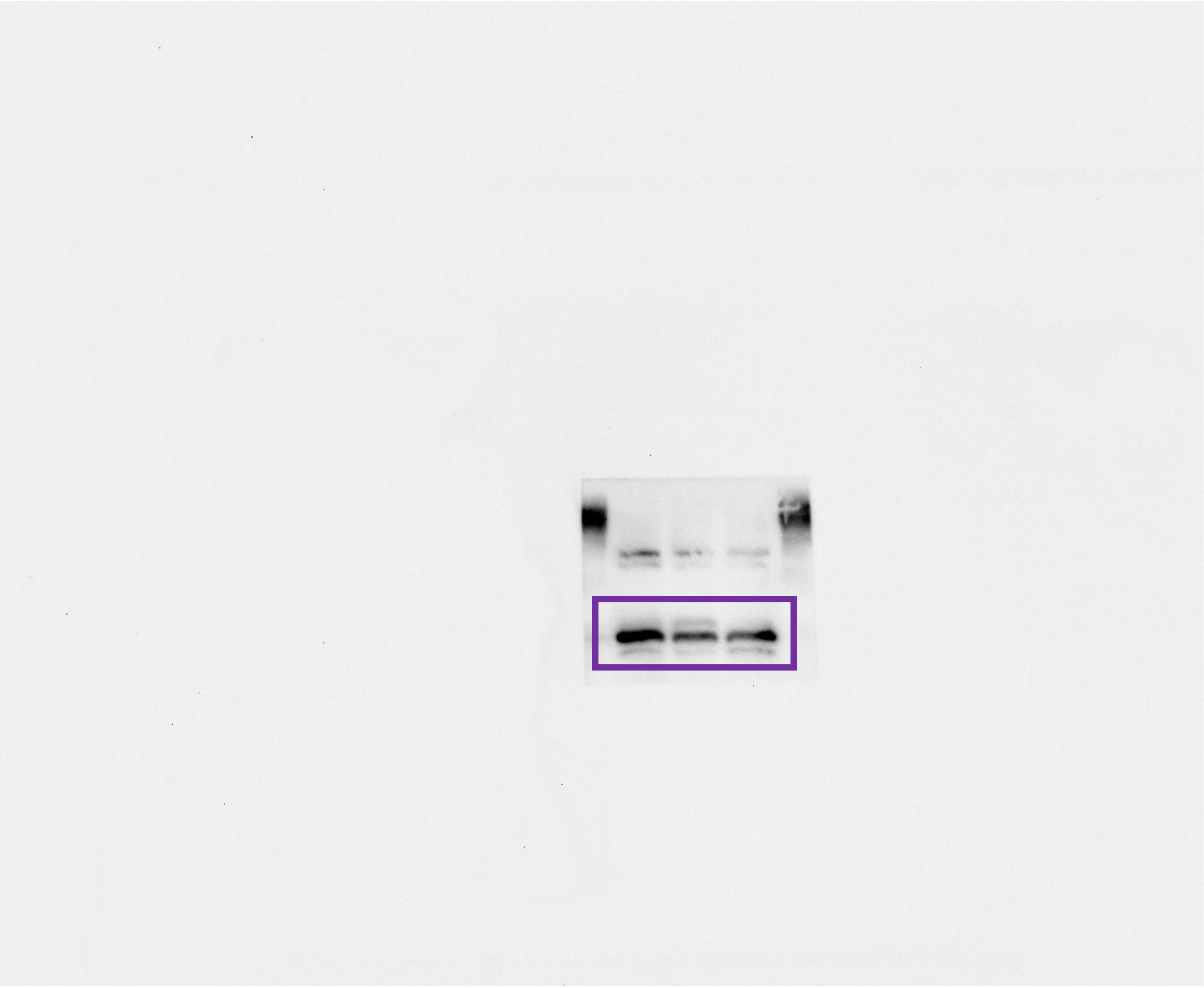

Figure 1A
MHCC97-H LC3

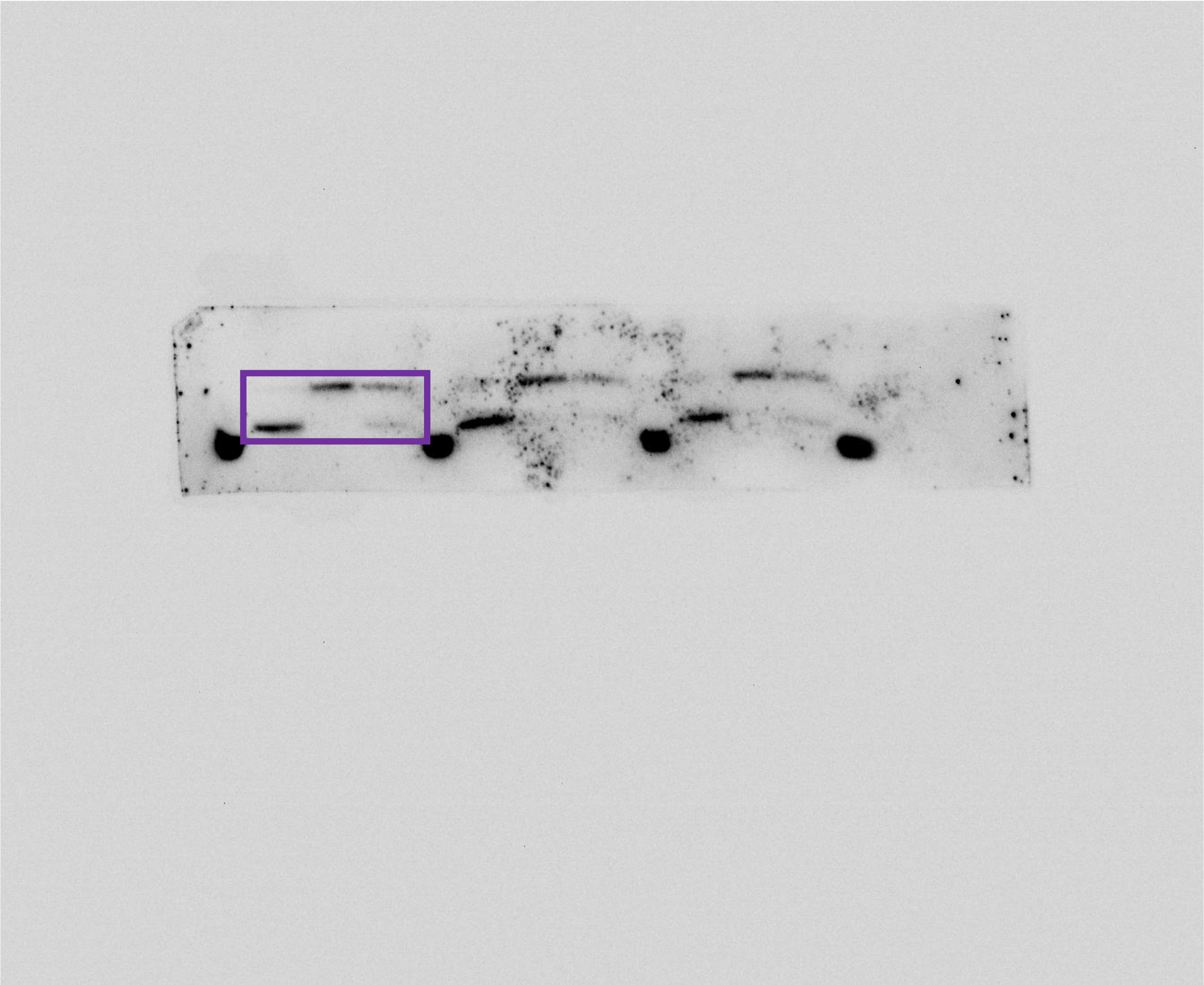

Figure 1A
MHCC97-H GAPDH

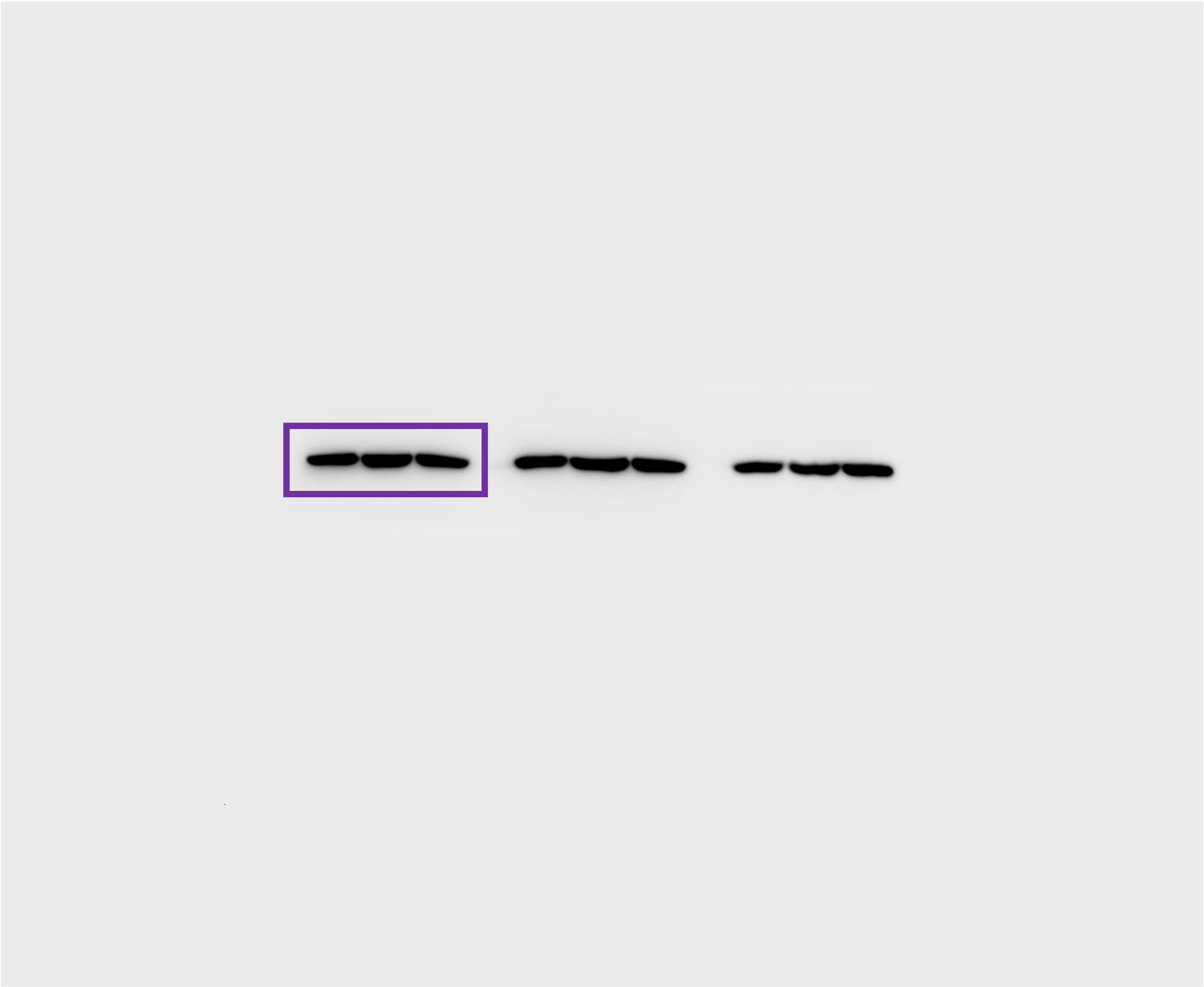

Figure 1A
Hep 3B ATG7

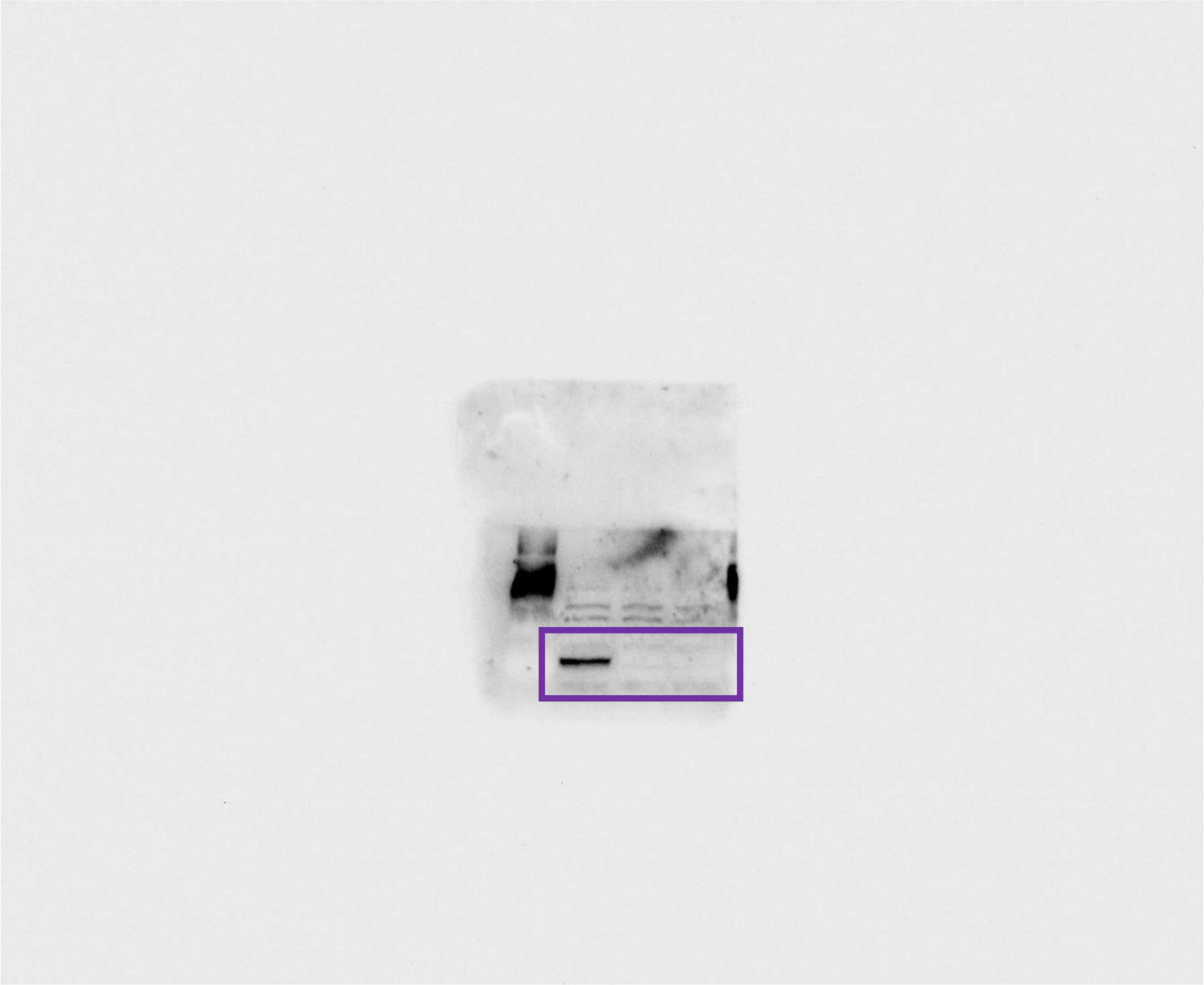

Figure 1A
Hep 3B p62

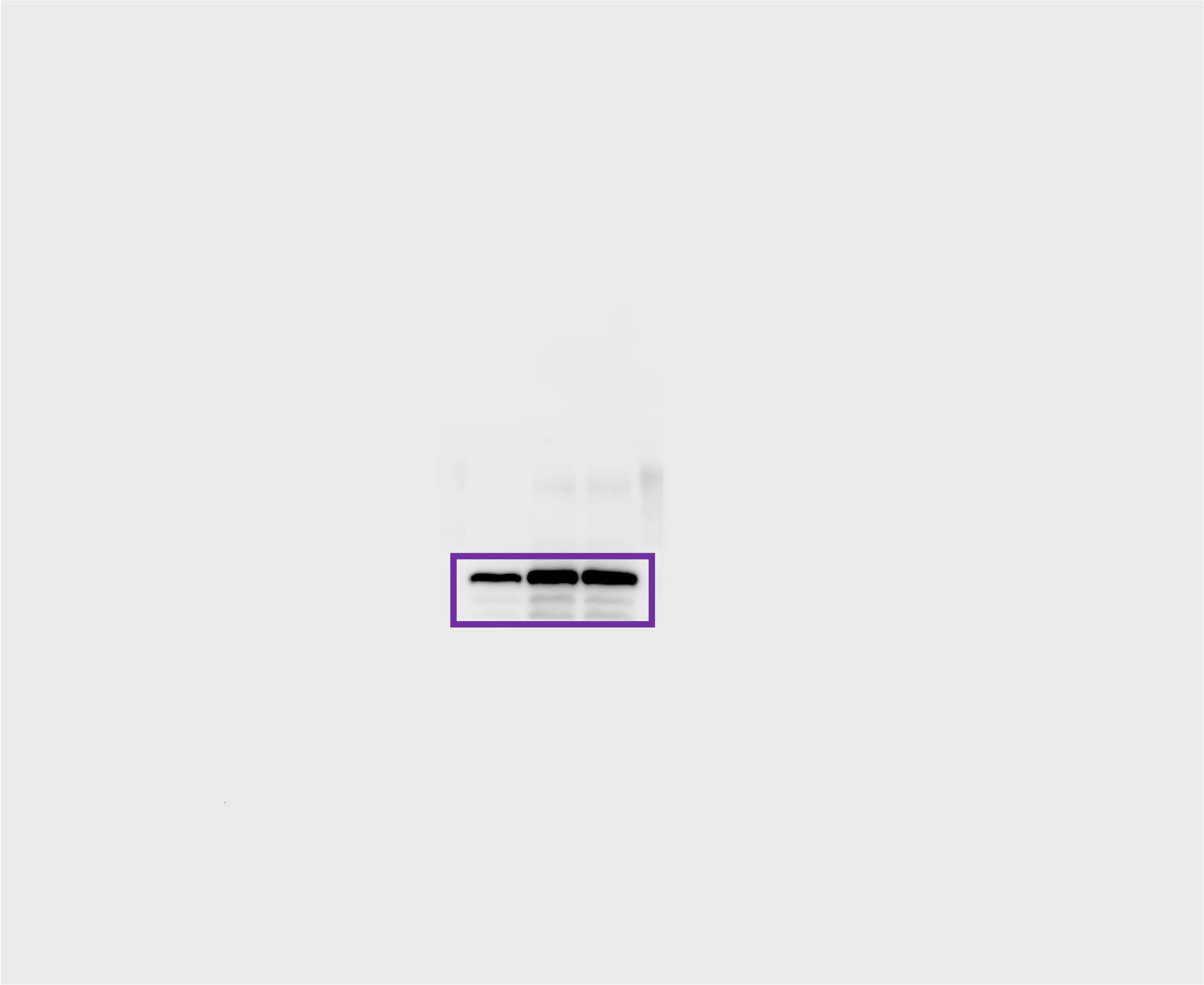

Figure 1A
Hep 3B BECLIN1

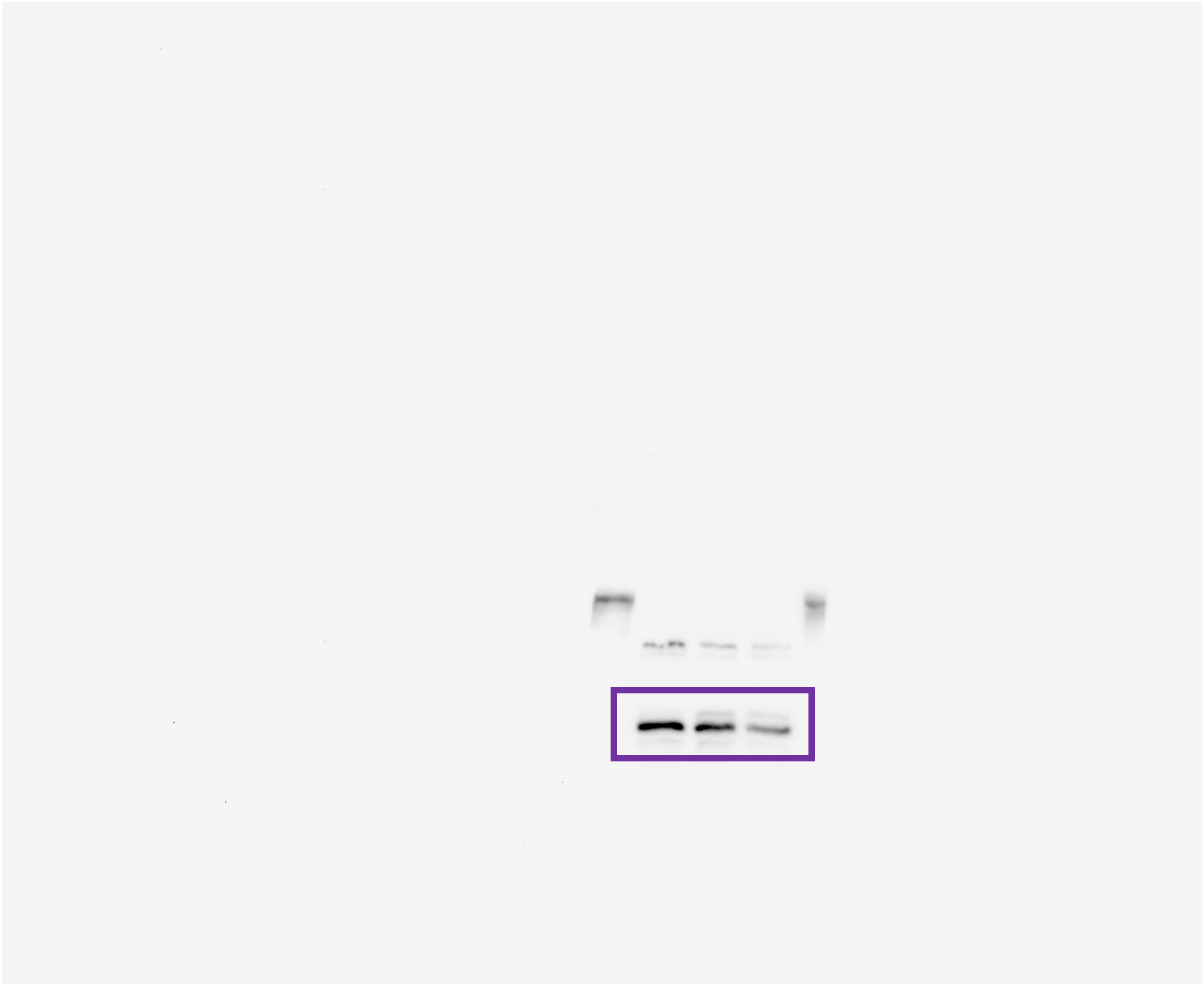

Figure 1A
Hep 3B LC3

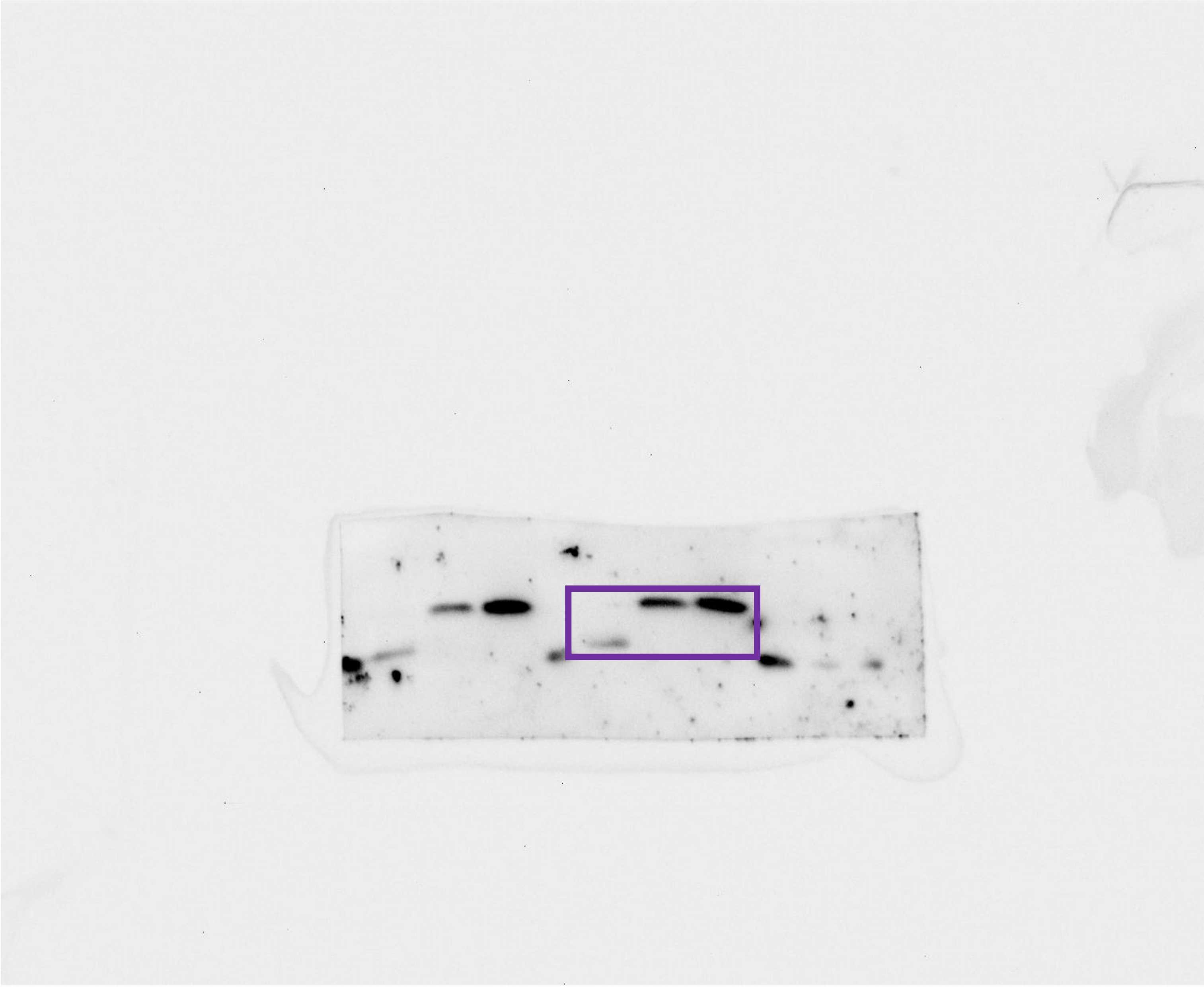

Figure 1A
Hep 3B GAPDH

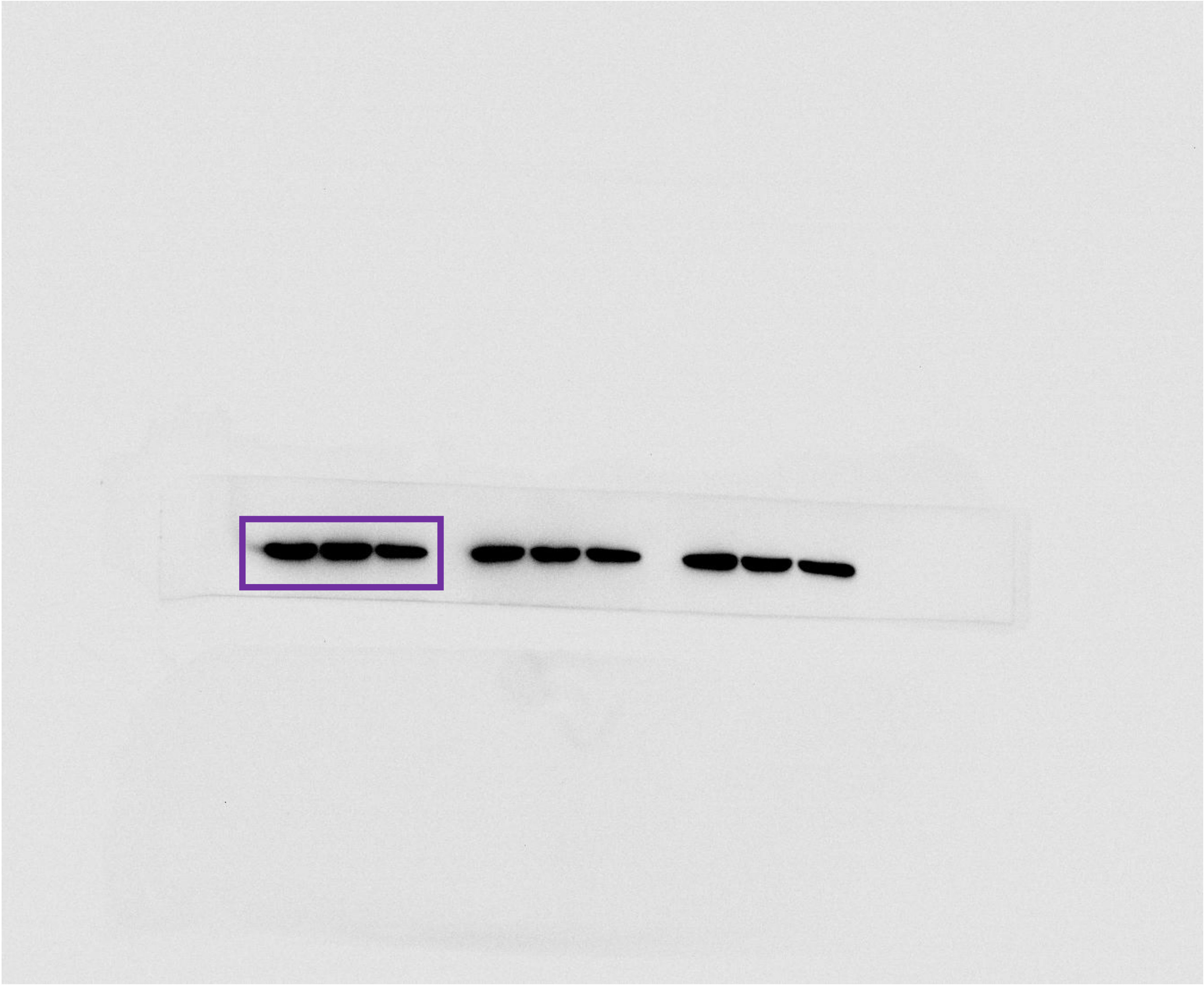

Figure 1B
MHCC97-H FLNA

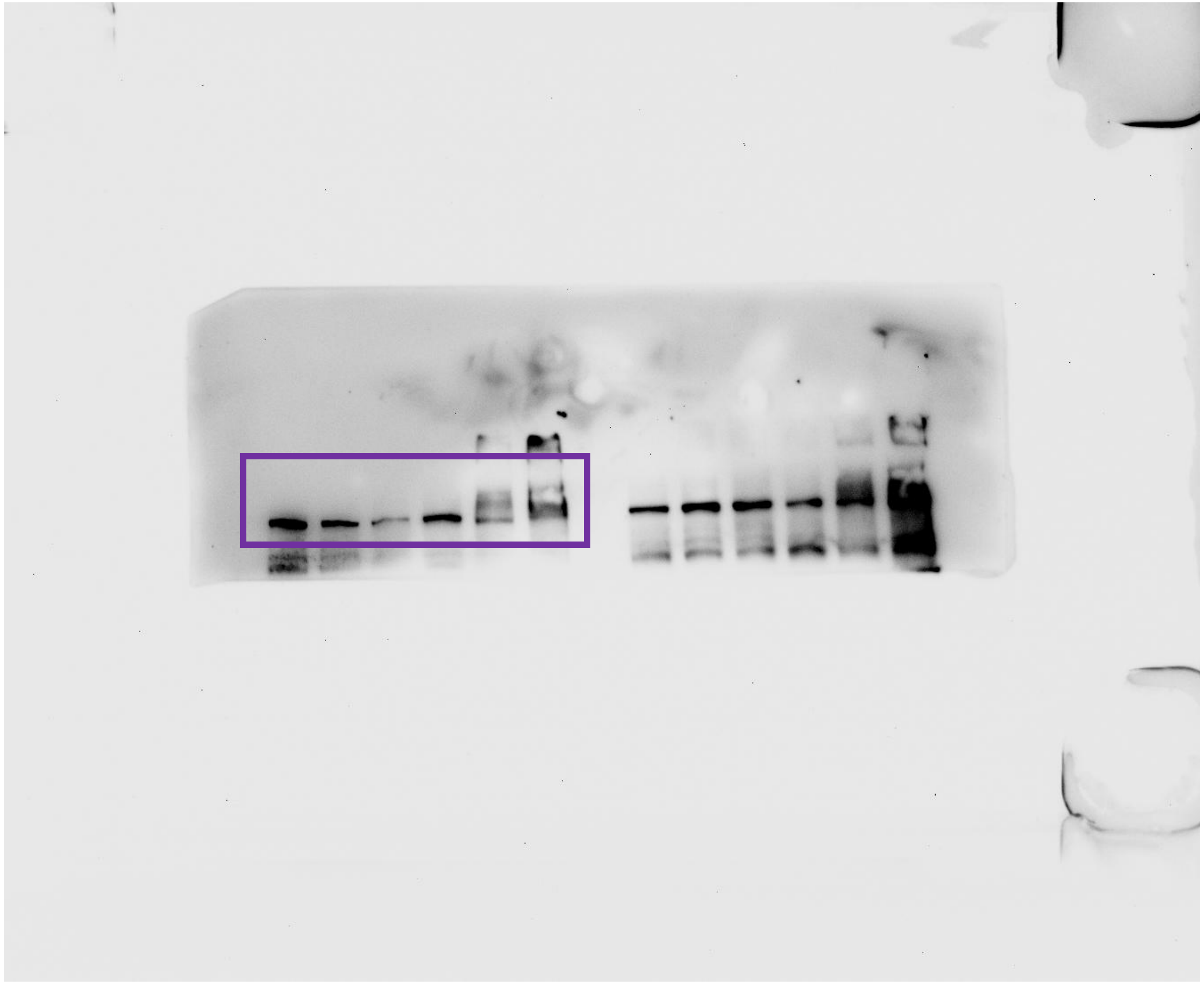

Figure 1B
MHCC97-H DREBRIN

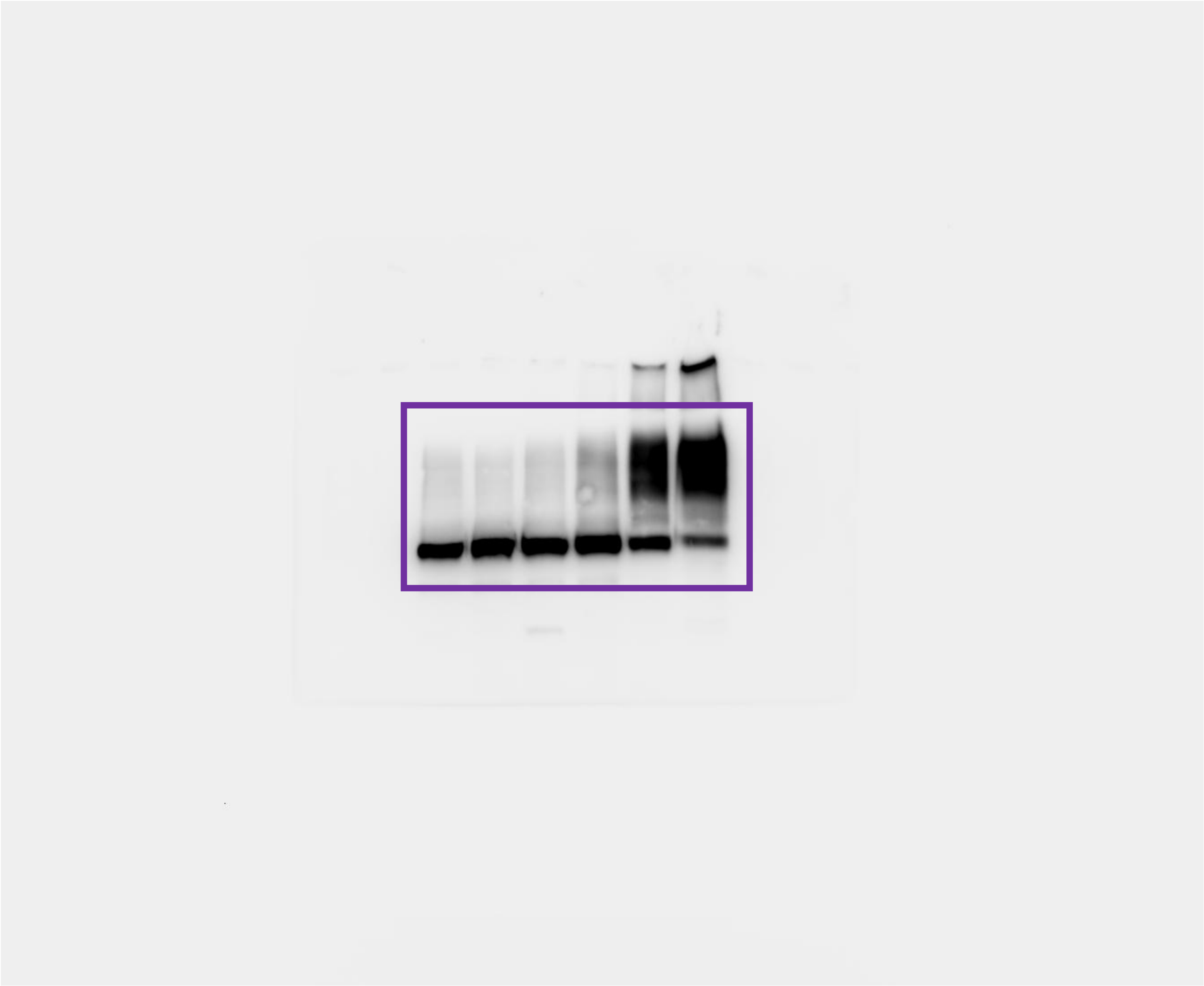

Figure 1B
MHCC97-H GAPDH

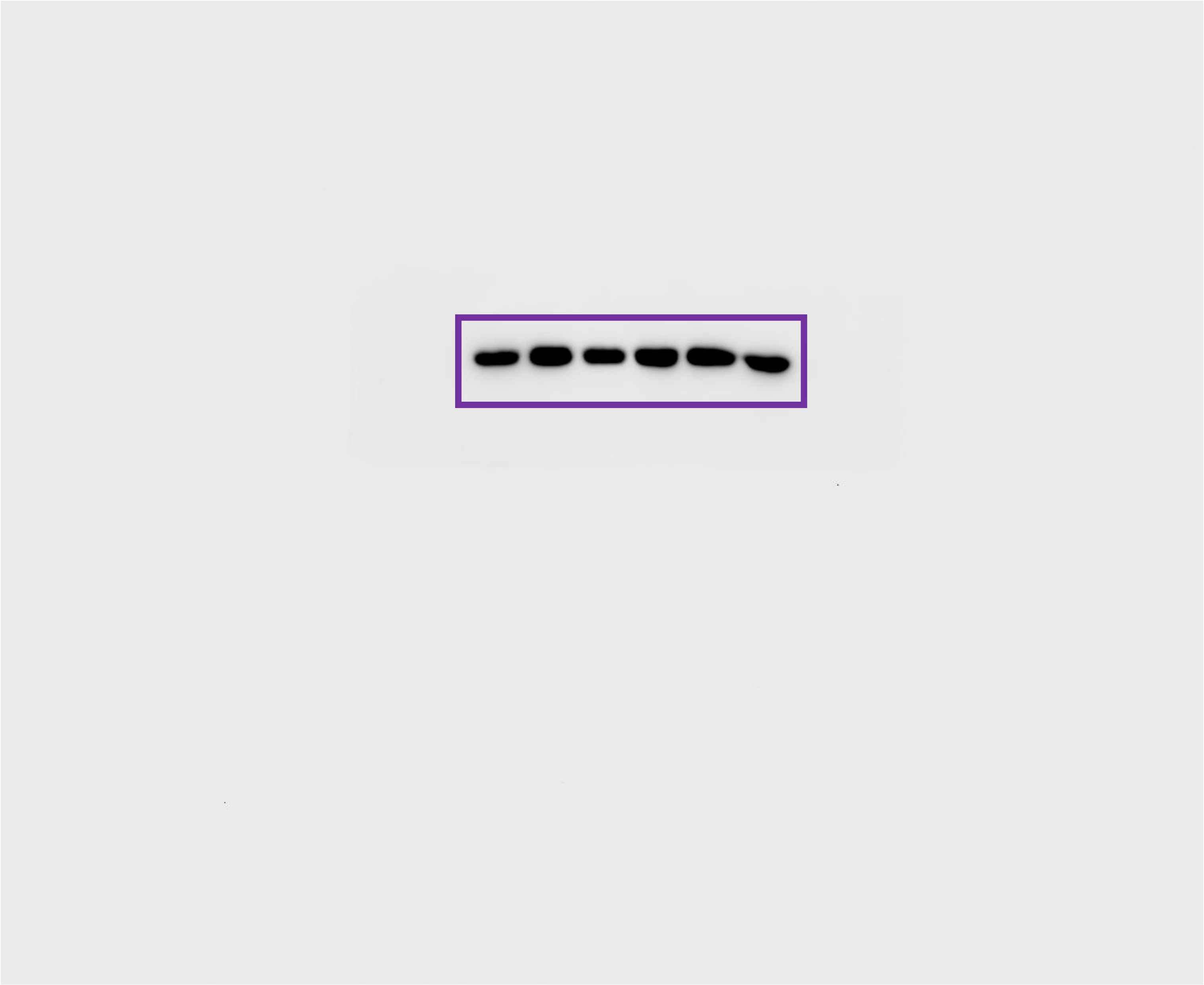

Figure 1B
Hep 3B FLNA

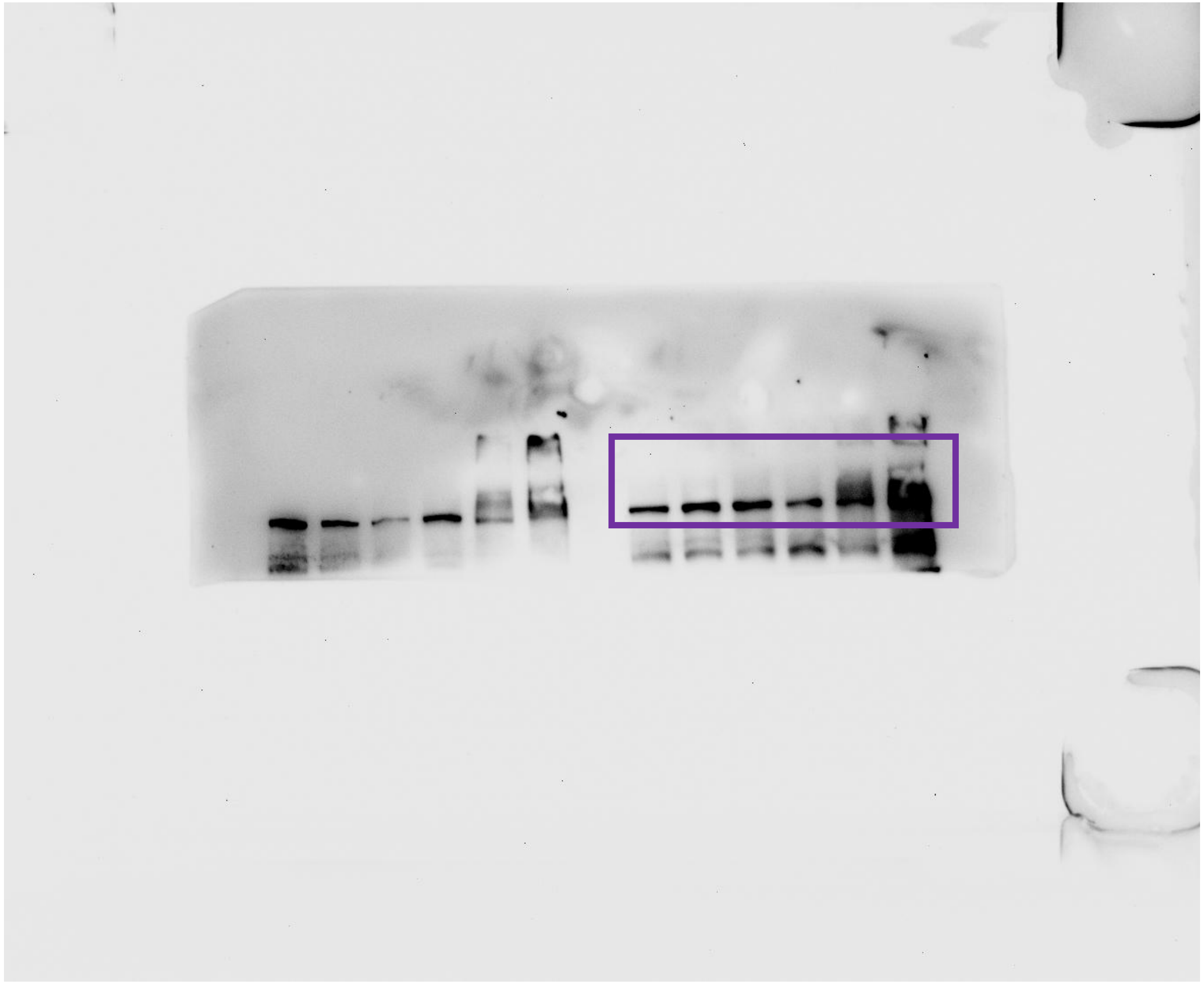

Figure 1B
Hep 3B DREBRIN

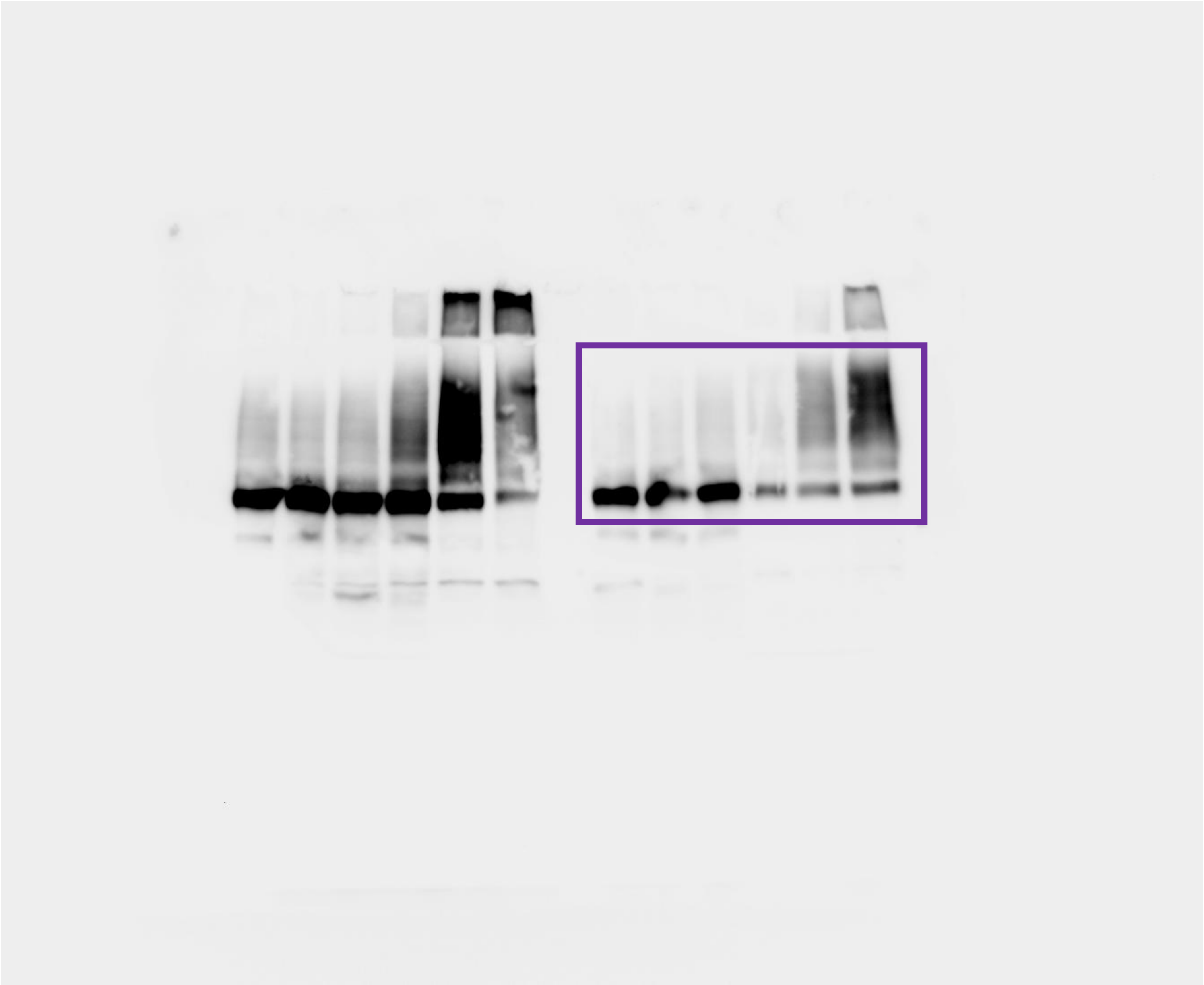

Figure 1B
Hep 3B GAPDH

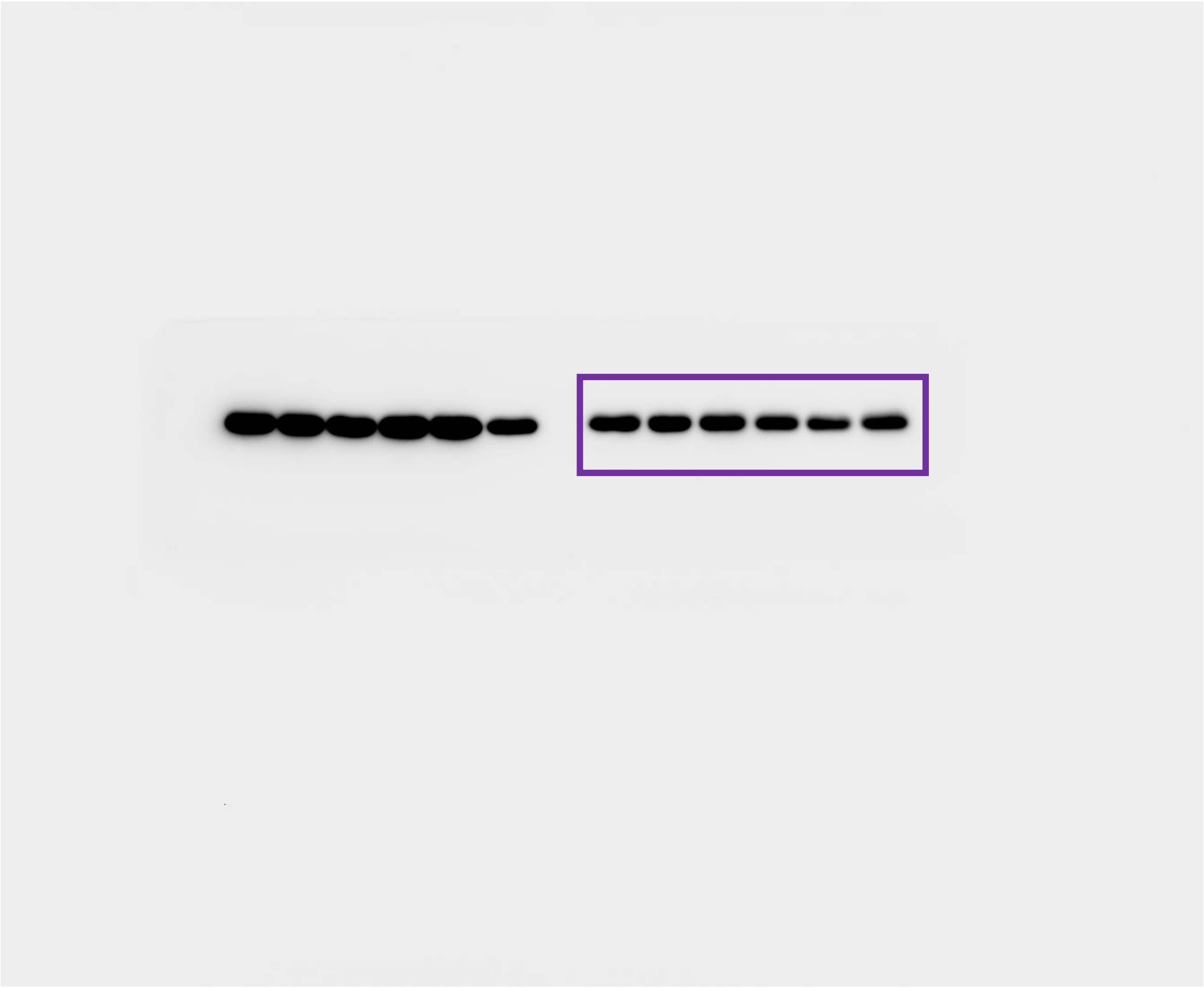

Figure 2A
MHCC97-H APN ATG7

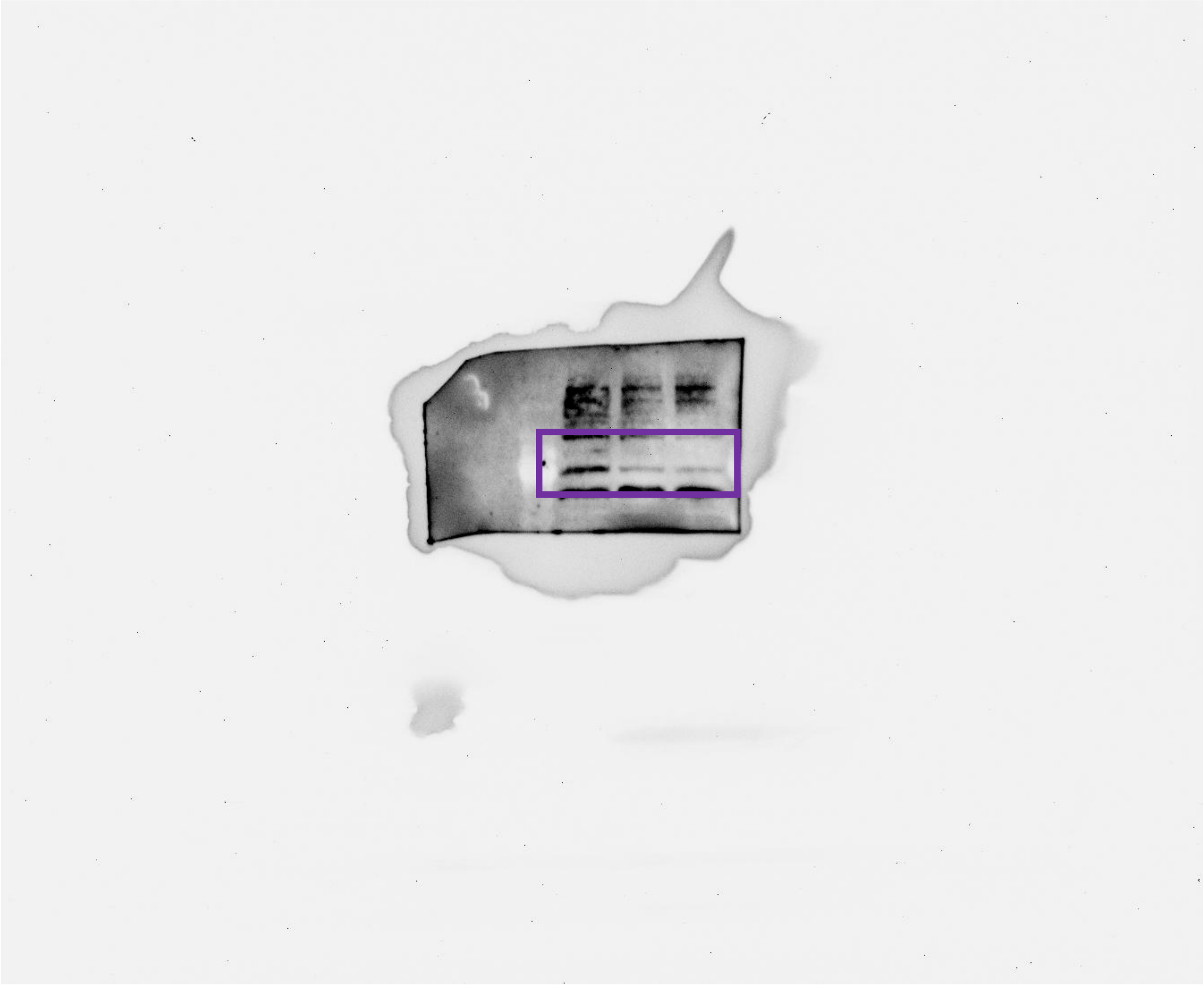

Figure 2A
MHCC97-H APN p62

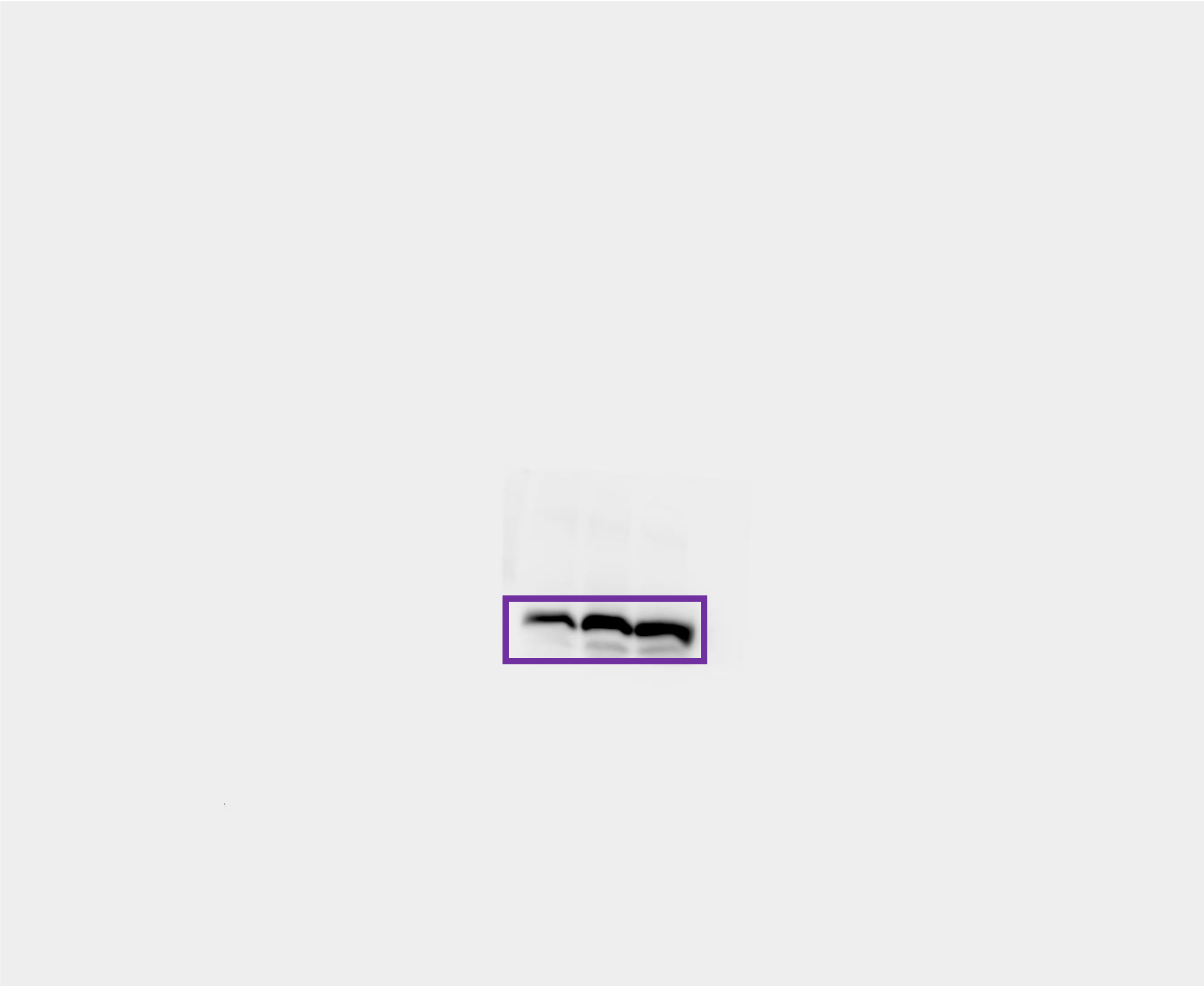

Figure 2A
MHCC97-H APN BECLIN1

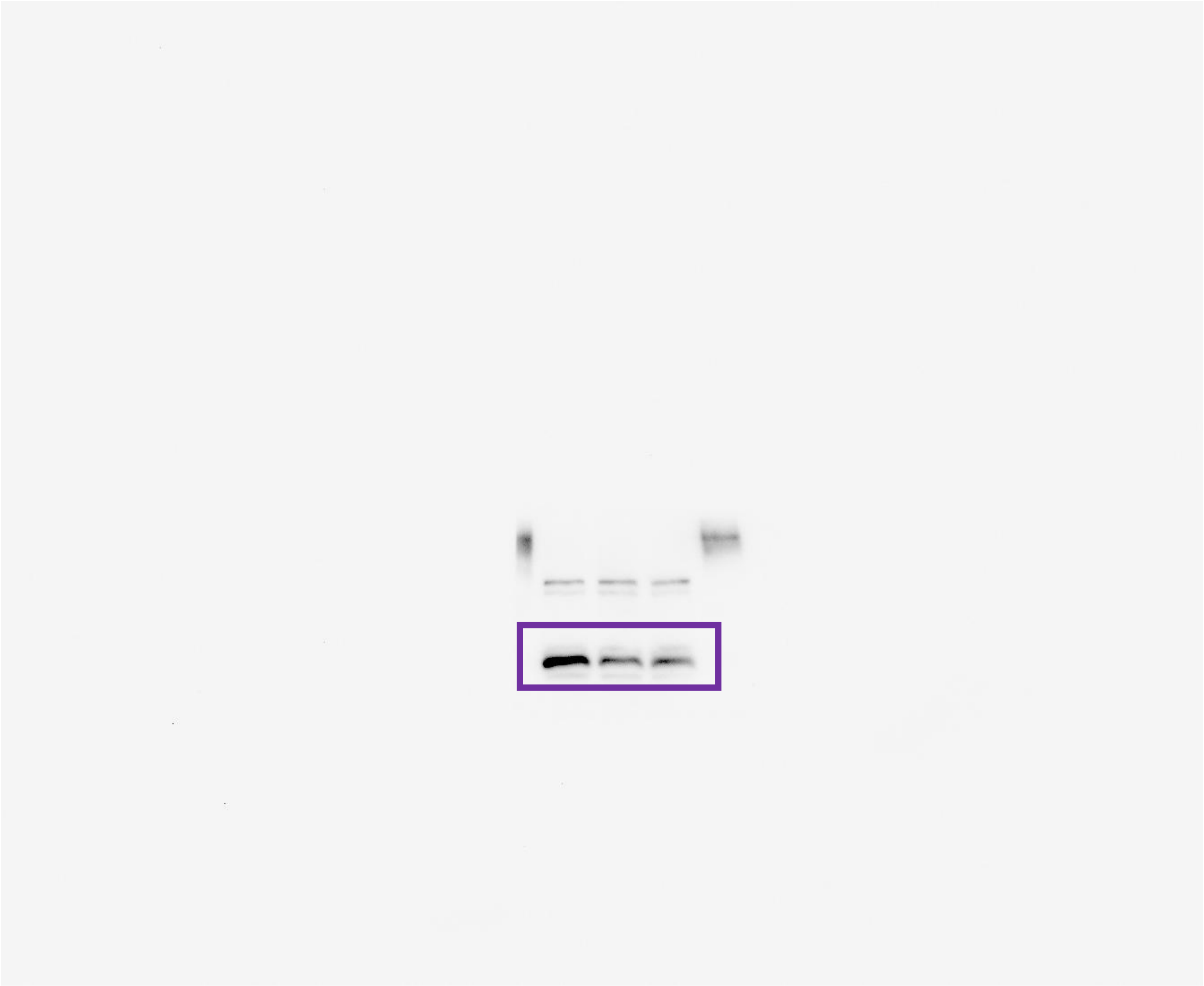

Figure 2A

MHCC97-H APN LC3

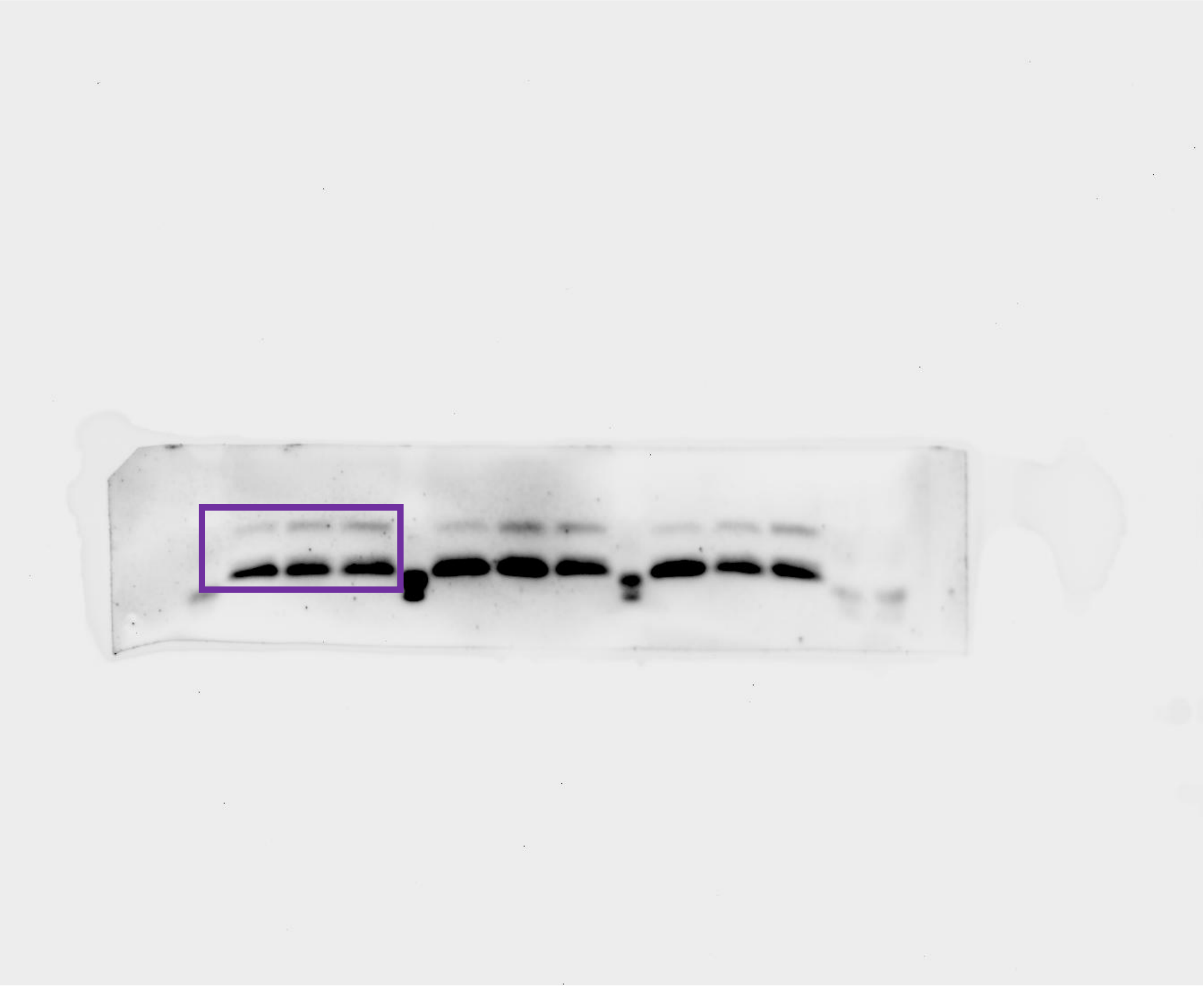

Figure 2A

MHCC97-H APN GAPDH

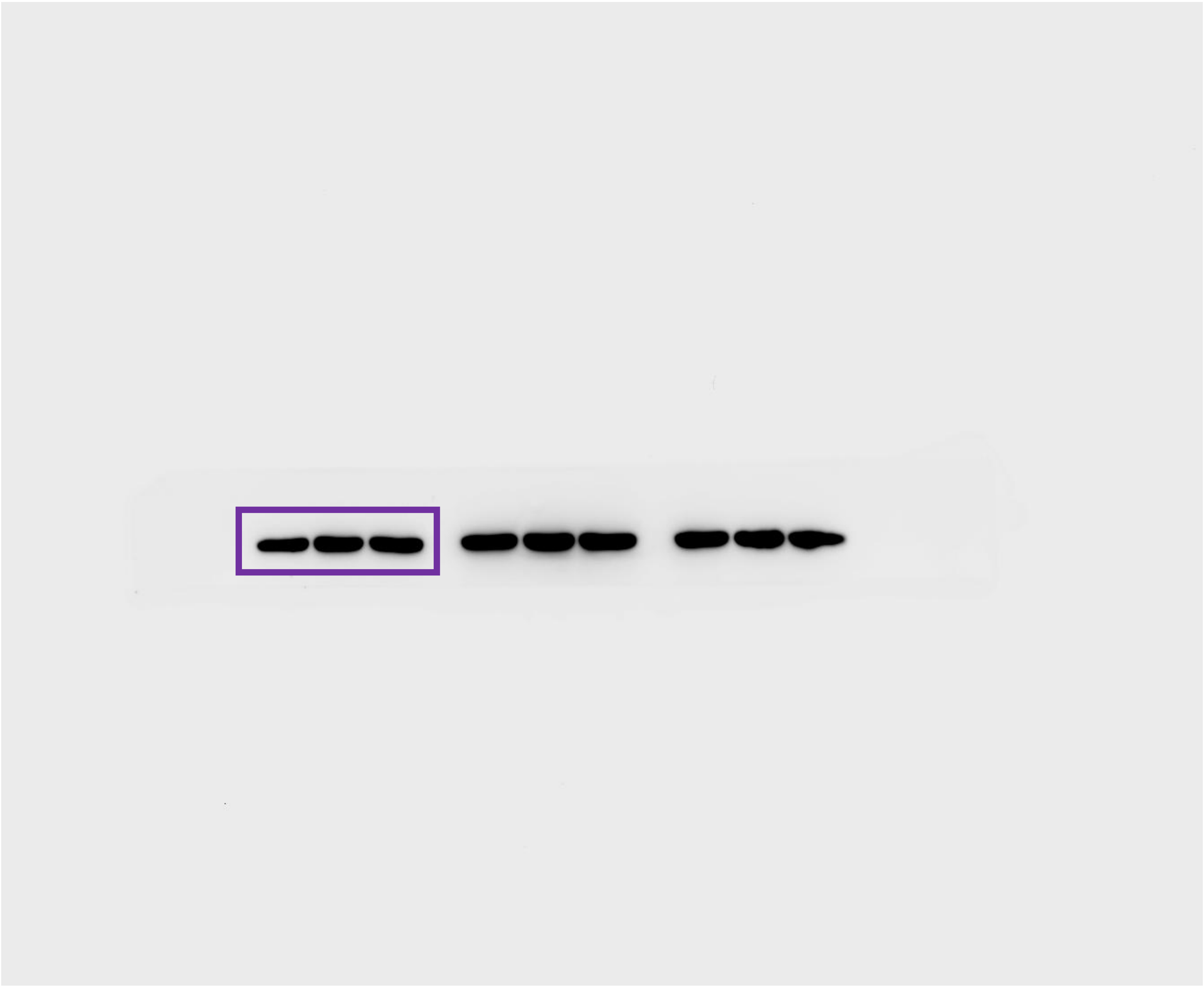

Figure 2A

MHCC97-H   CQ ATG7

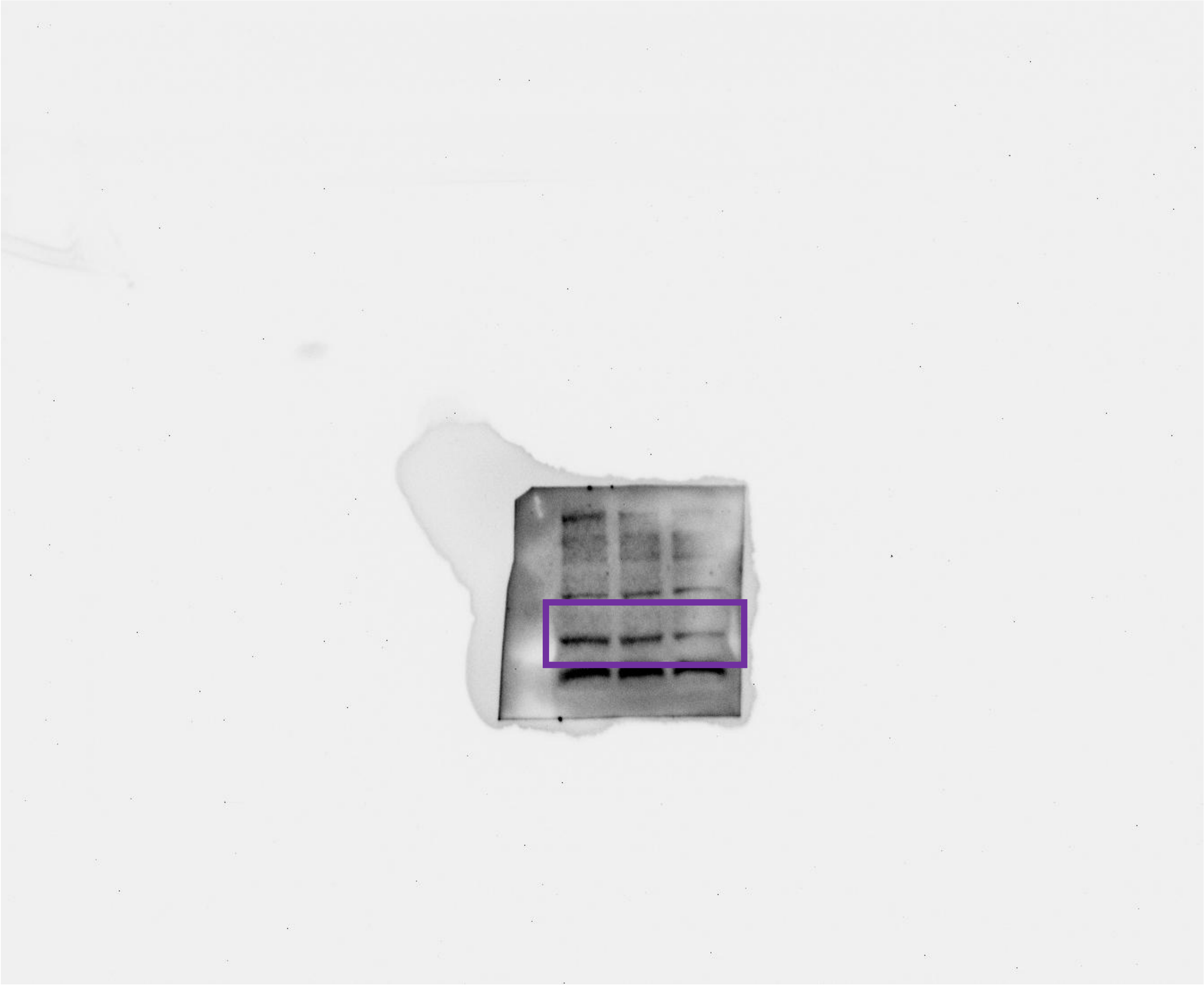

Figure 2A
MHCC97-H CQ p62

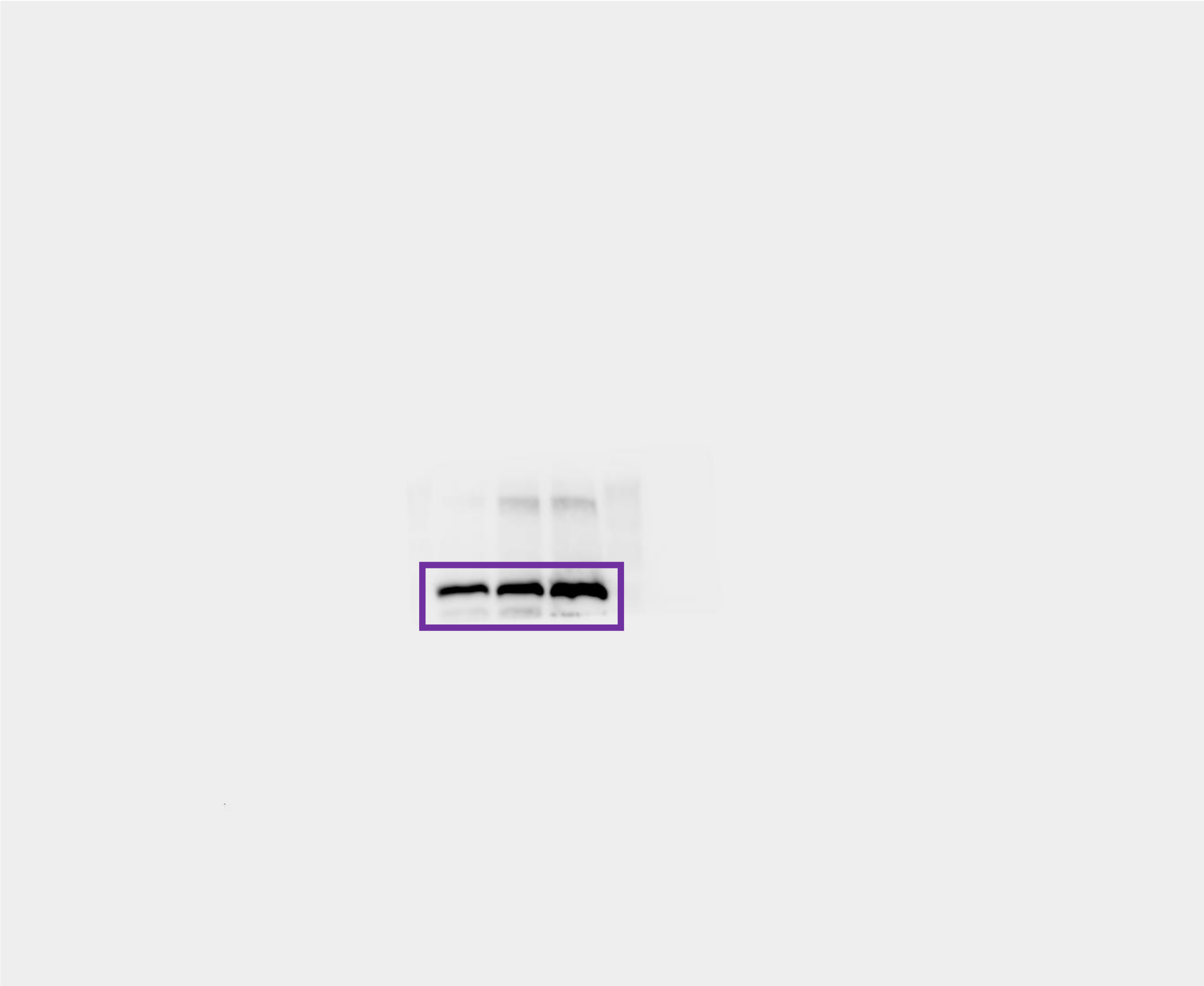

Figure 2A
MHCC97-H CQ BECLIN1

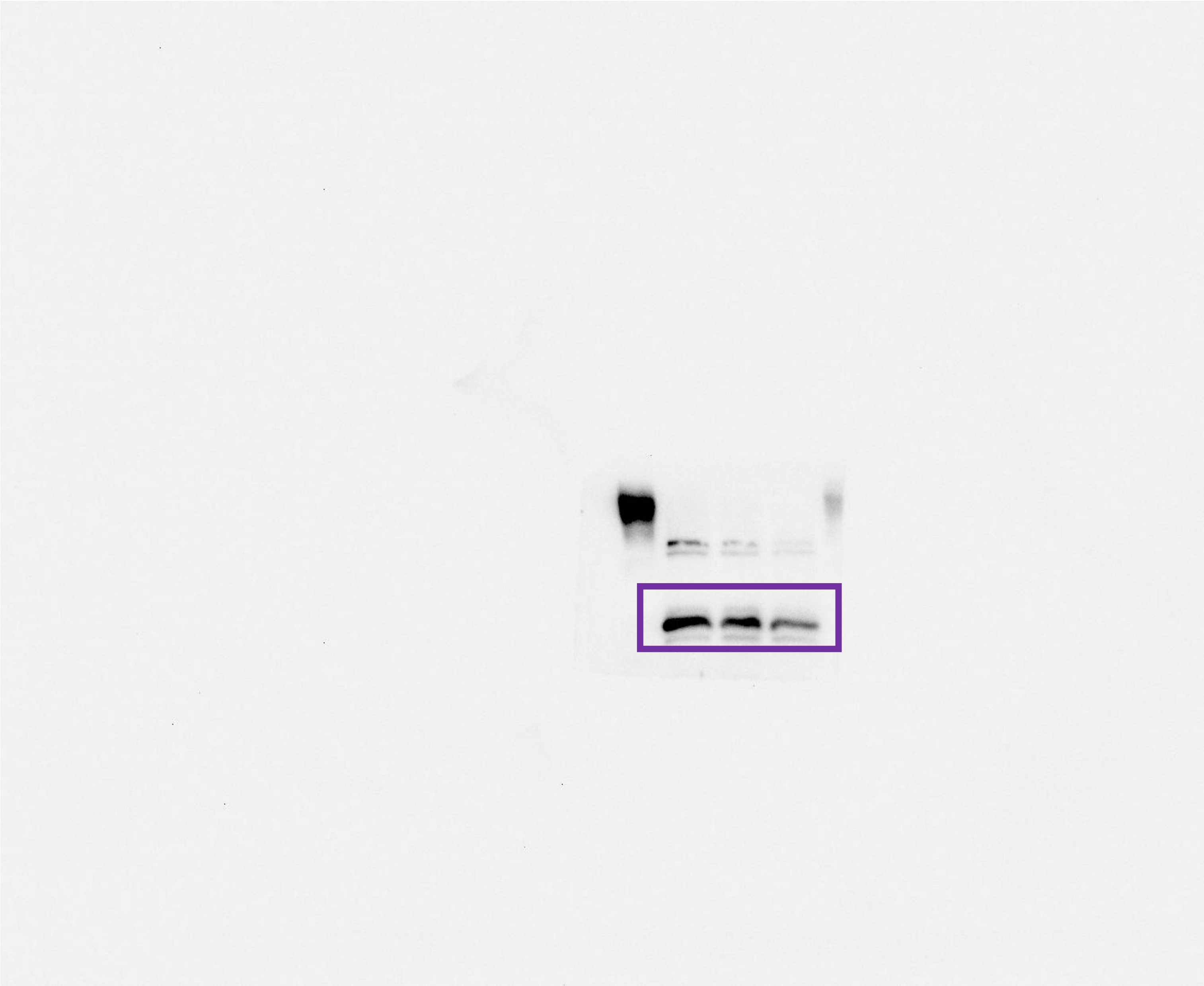

Figure 2A
MHCC97-H CQ LC3

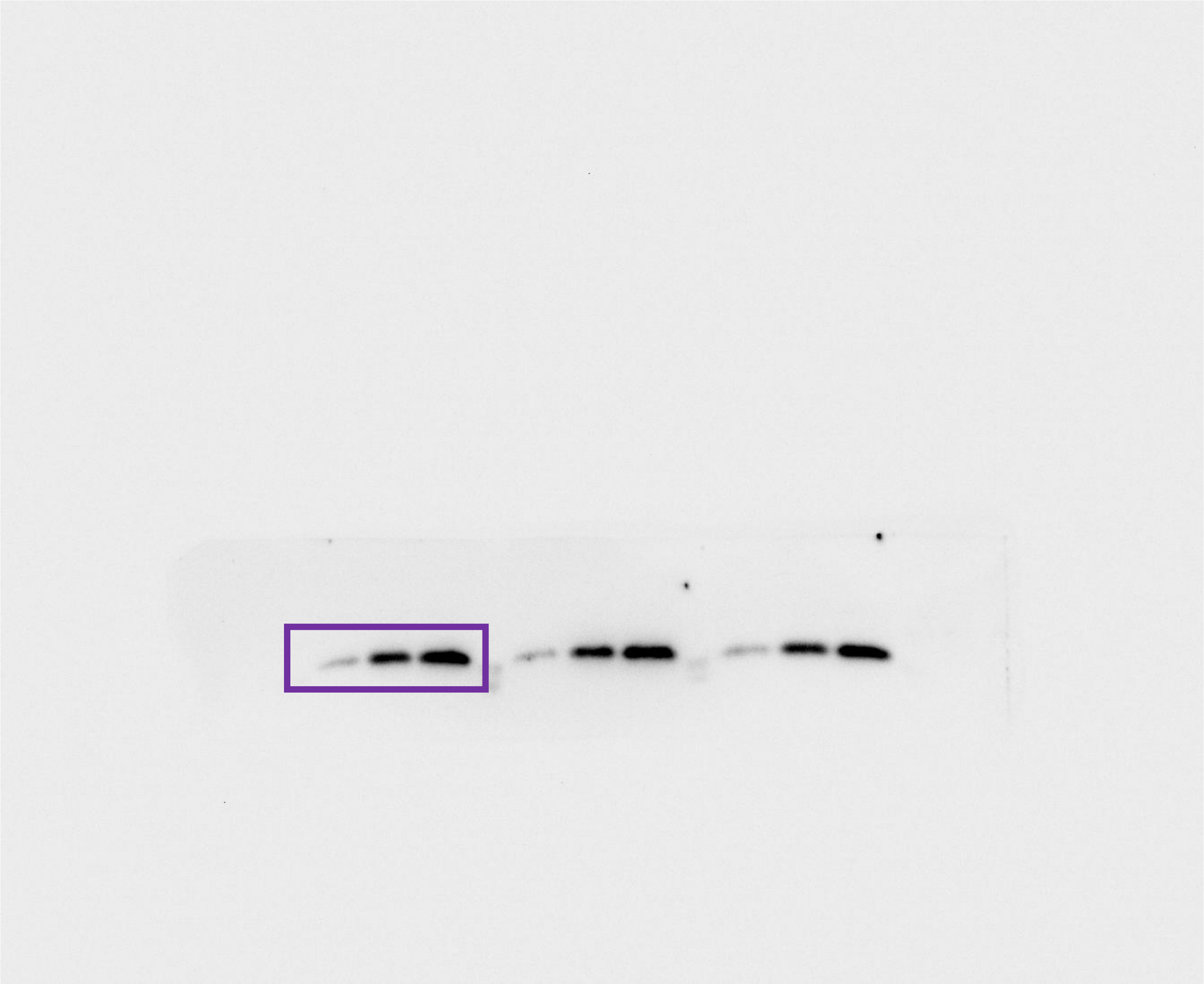

Figure 2A
MHCC97-H CQ GAPDH

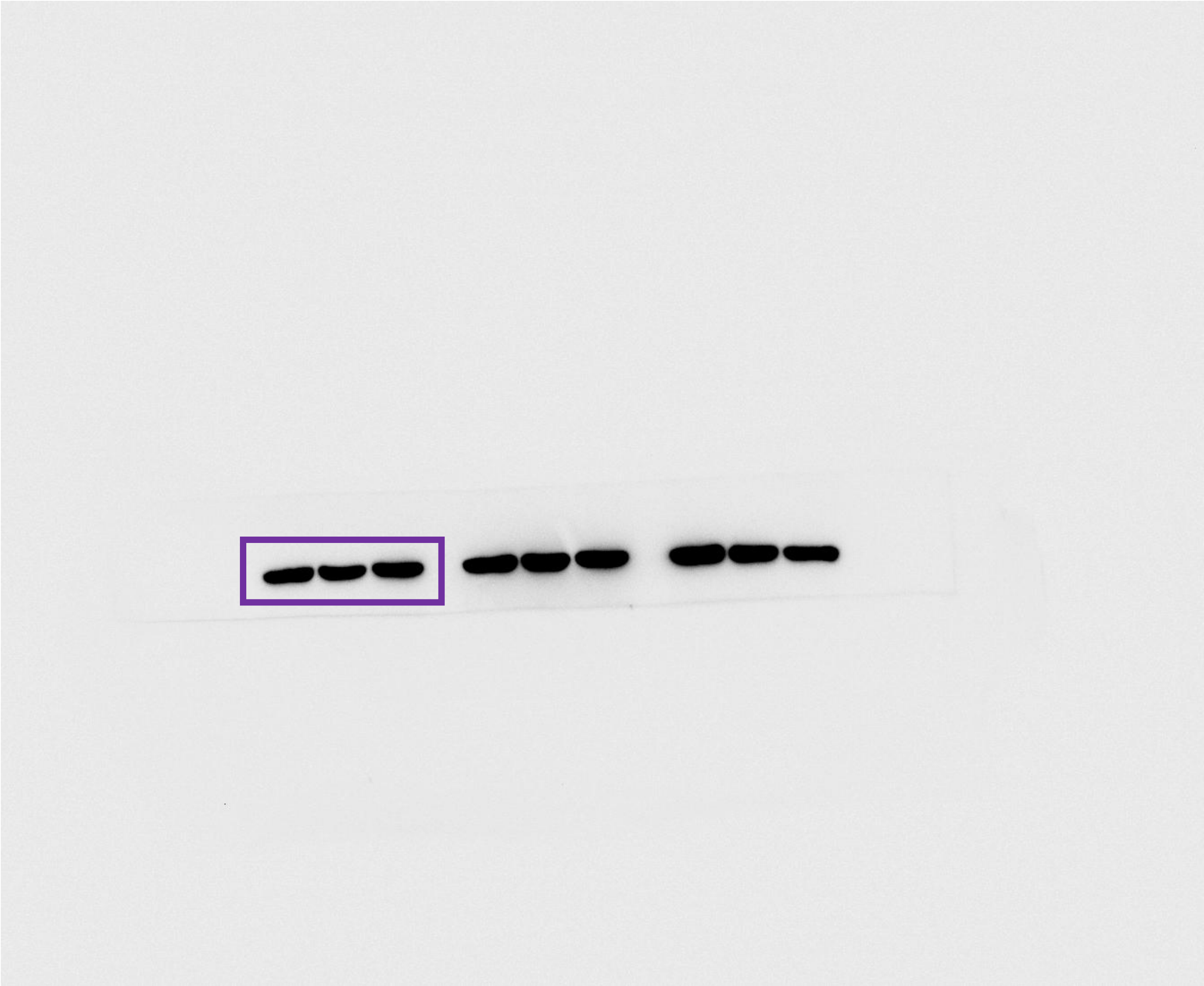

Figure 2B
Hep 3B APN ATG7

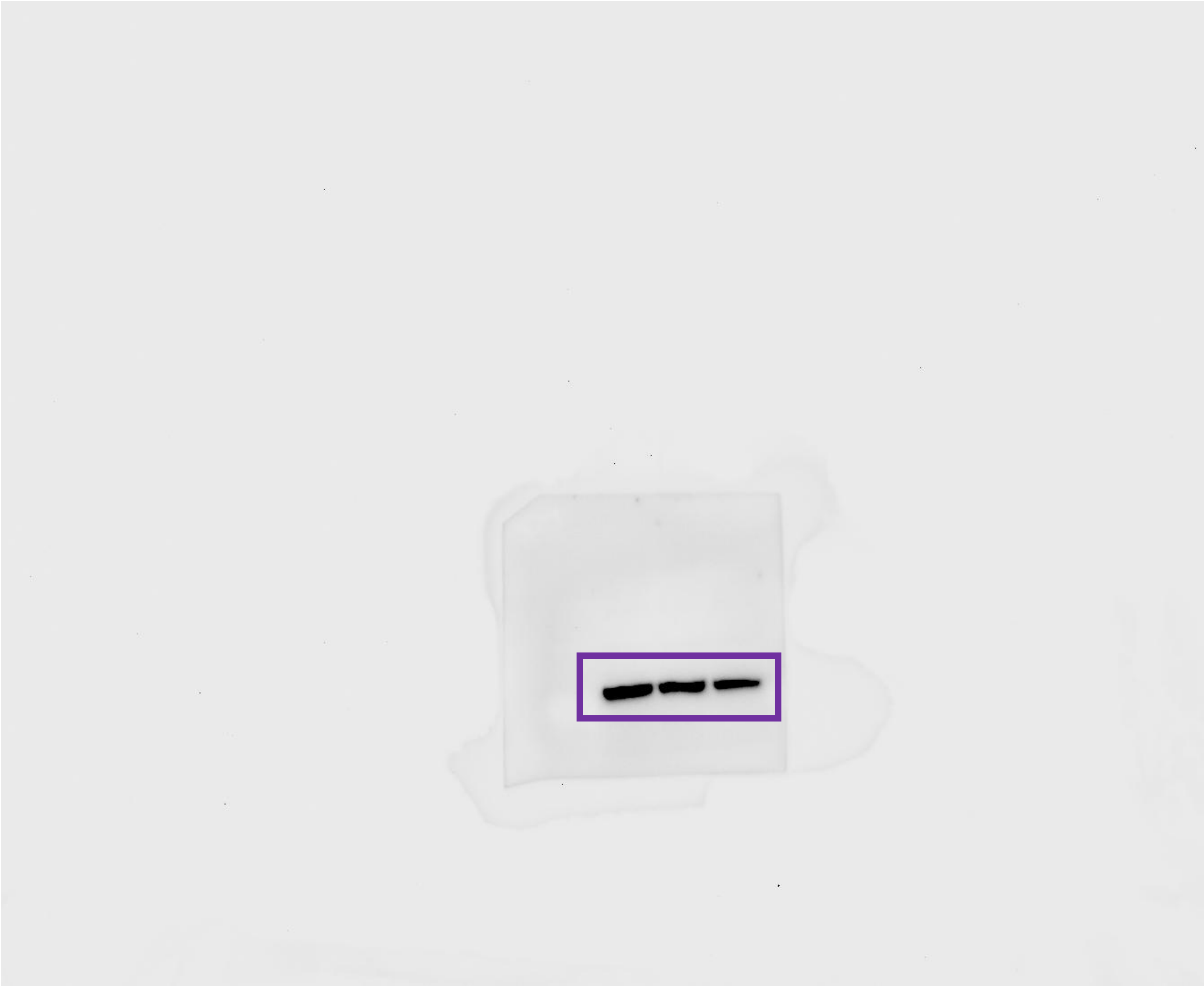

Figure 2B
Hep 3B APN p62

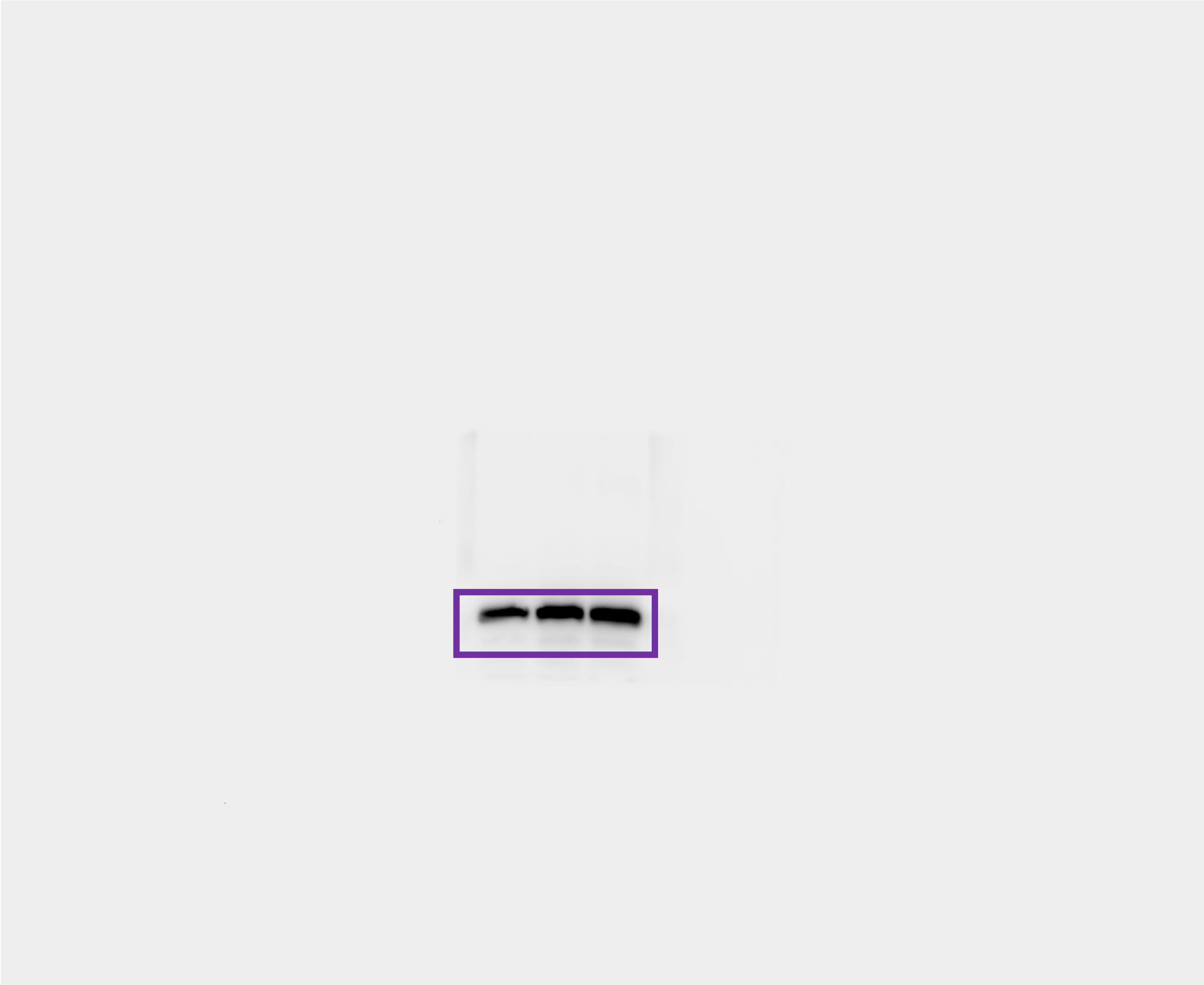

Figure 2B
Hep 3B APN BECLIN1

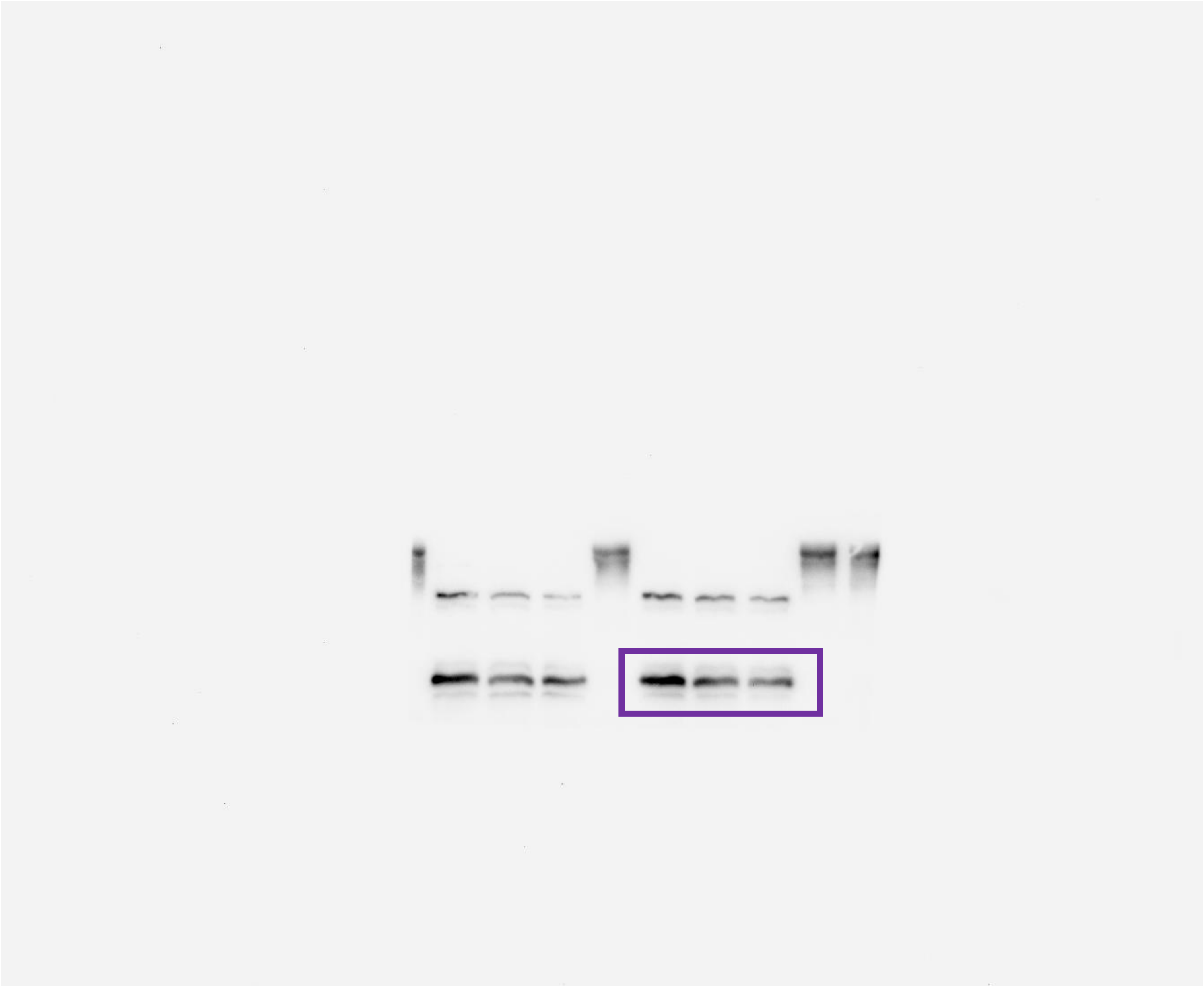

Figure 2B
Hep 3B APN LC3

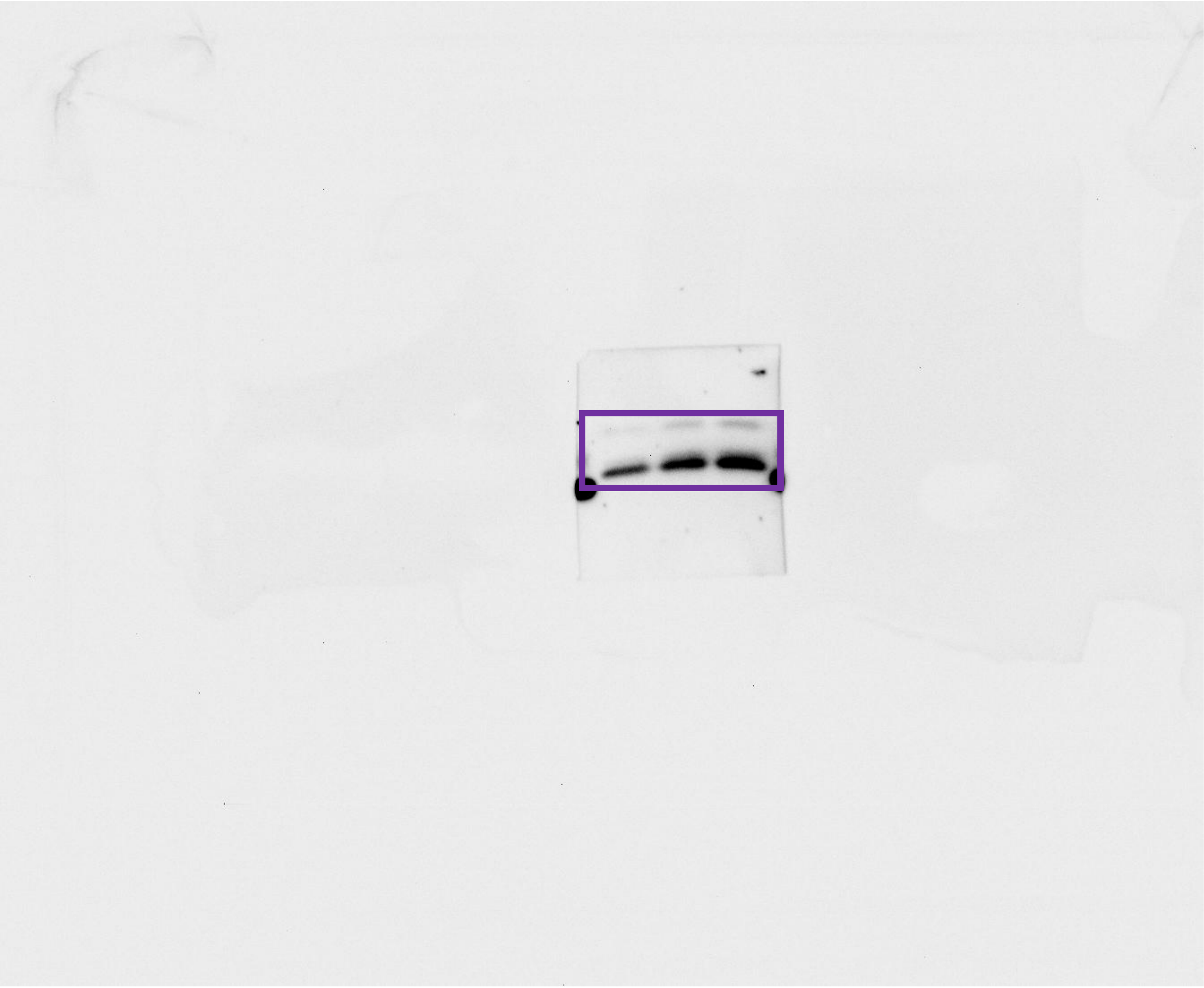

Figure 2B
Hep 3B APN GAPDH

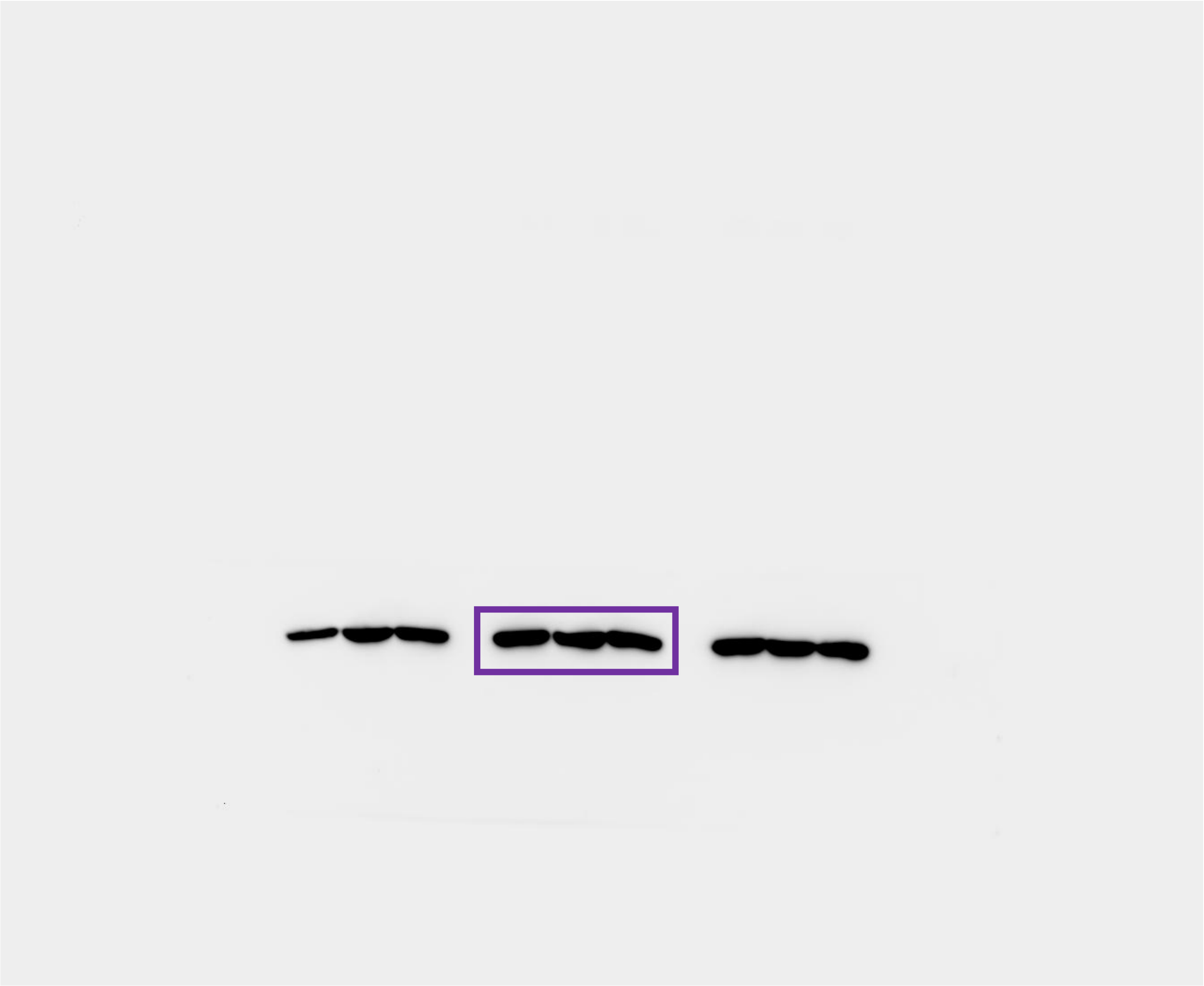

Figure 2B
Hep 3B CQ ATG7

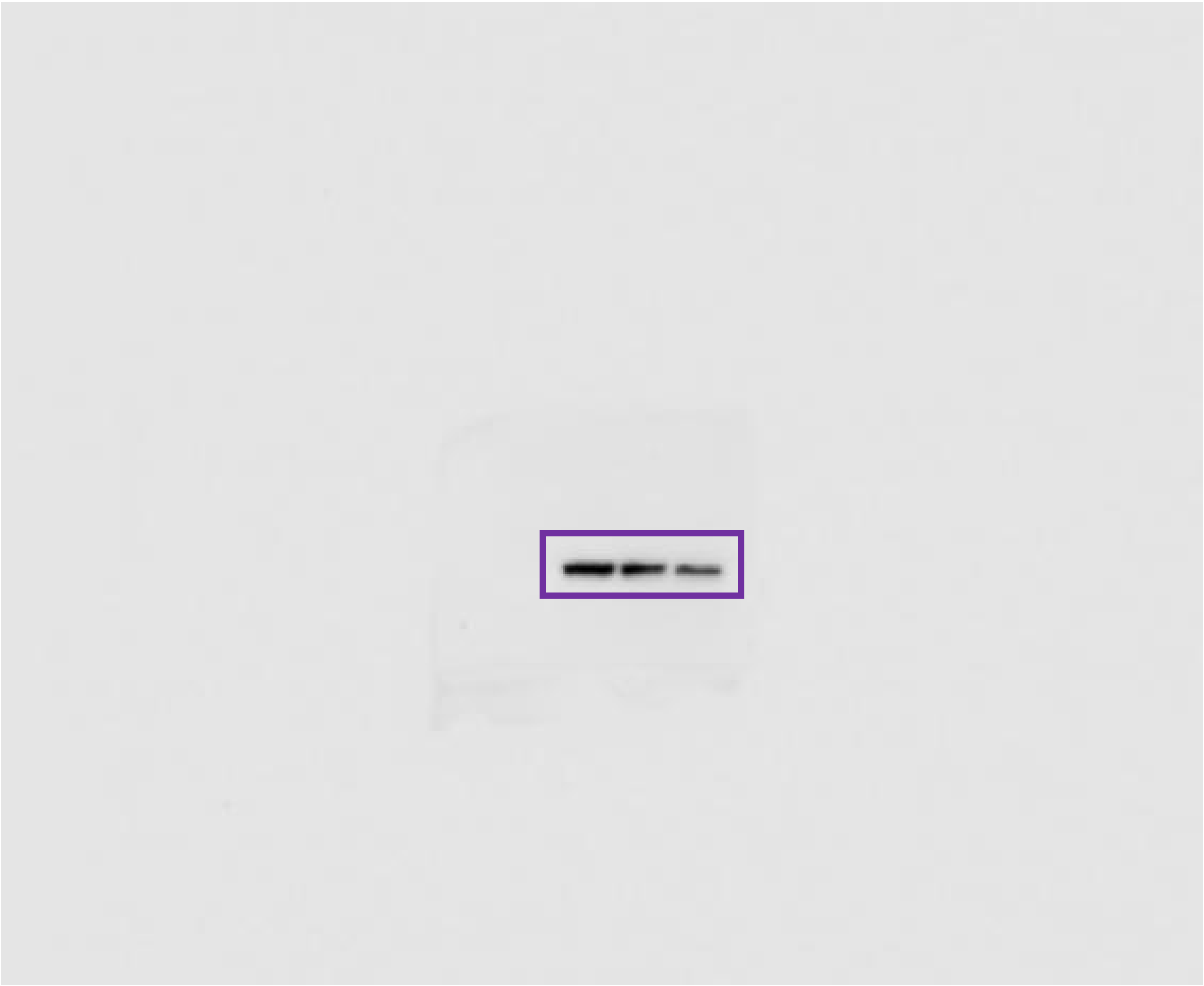

Figure 2B
Hep 3B CQ p62

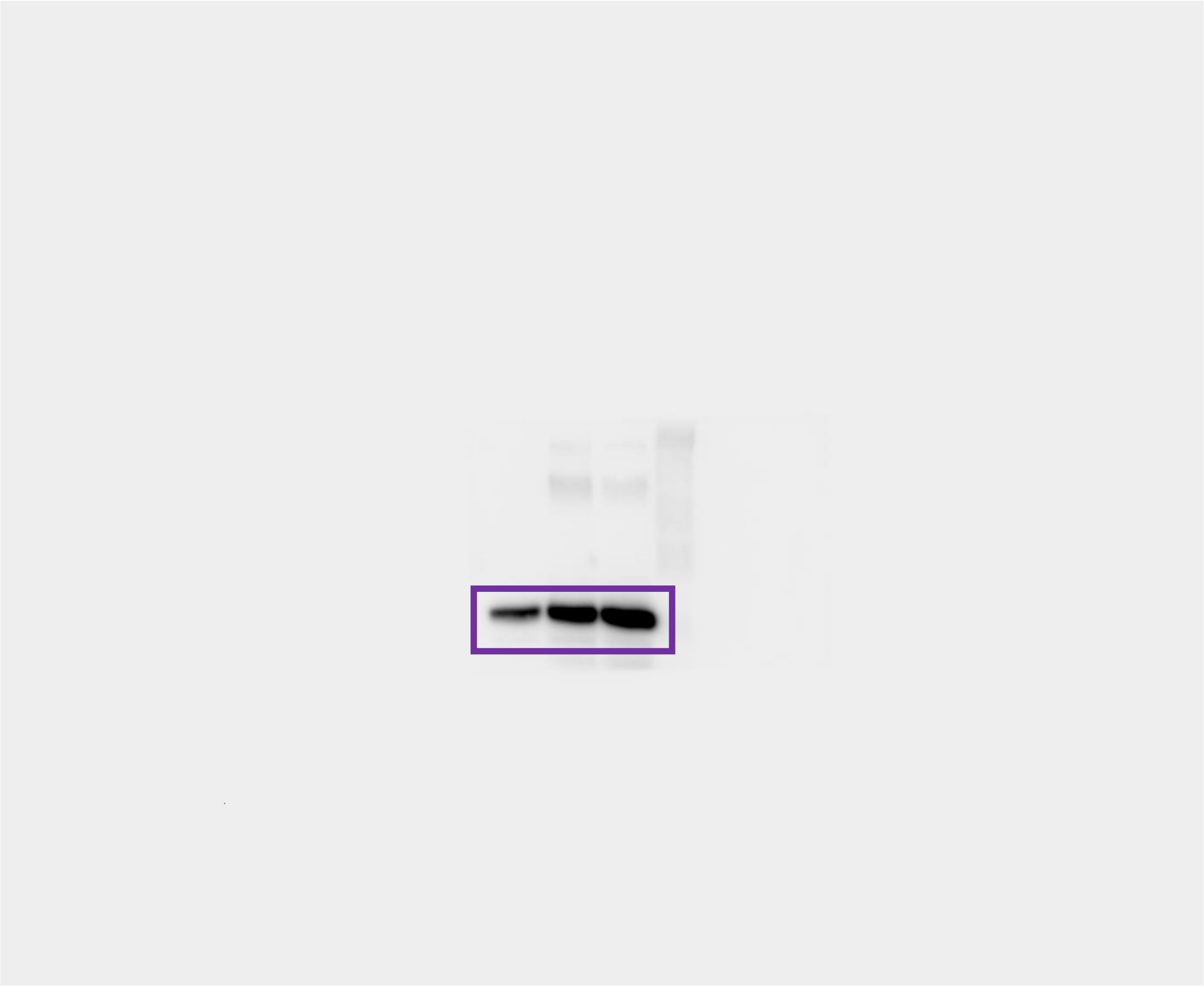

Figure 2B
Hep 3B CQ BECLIN1

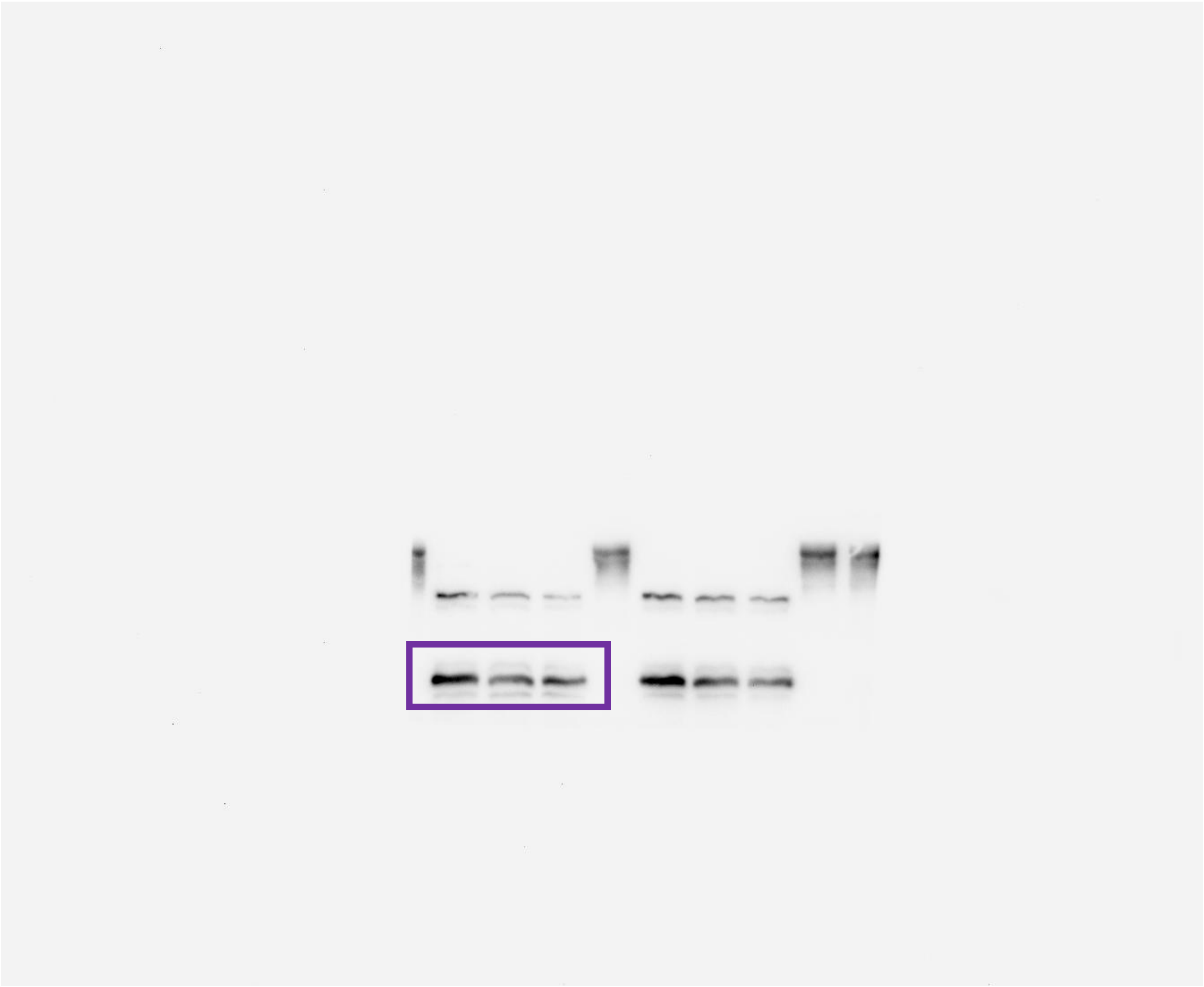

Figure 2B
Hep 3B CQ LC3

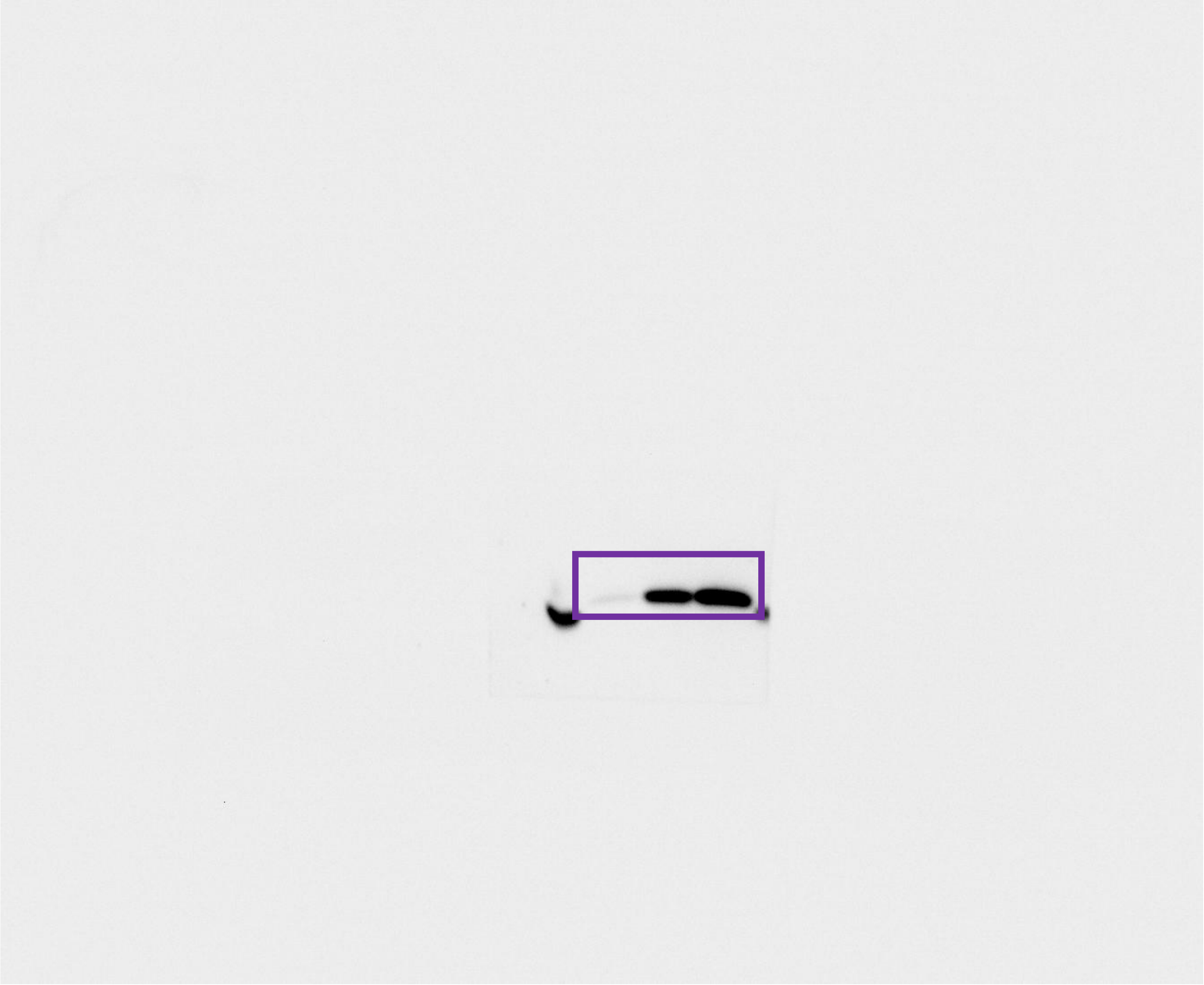

Figure 2B
Hep 3B CQ GAPDH

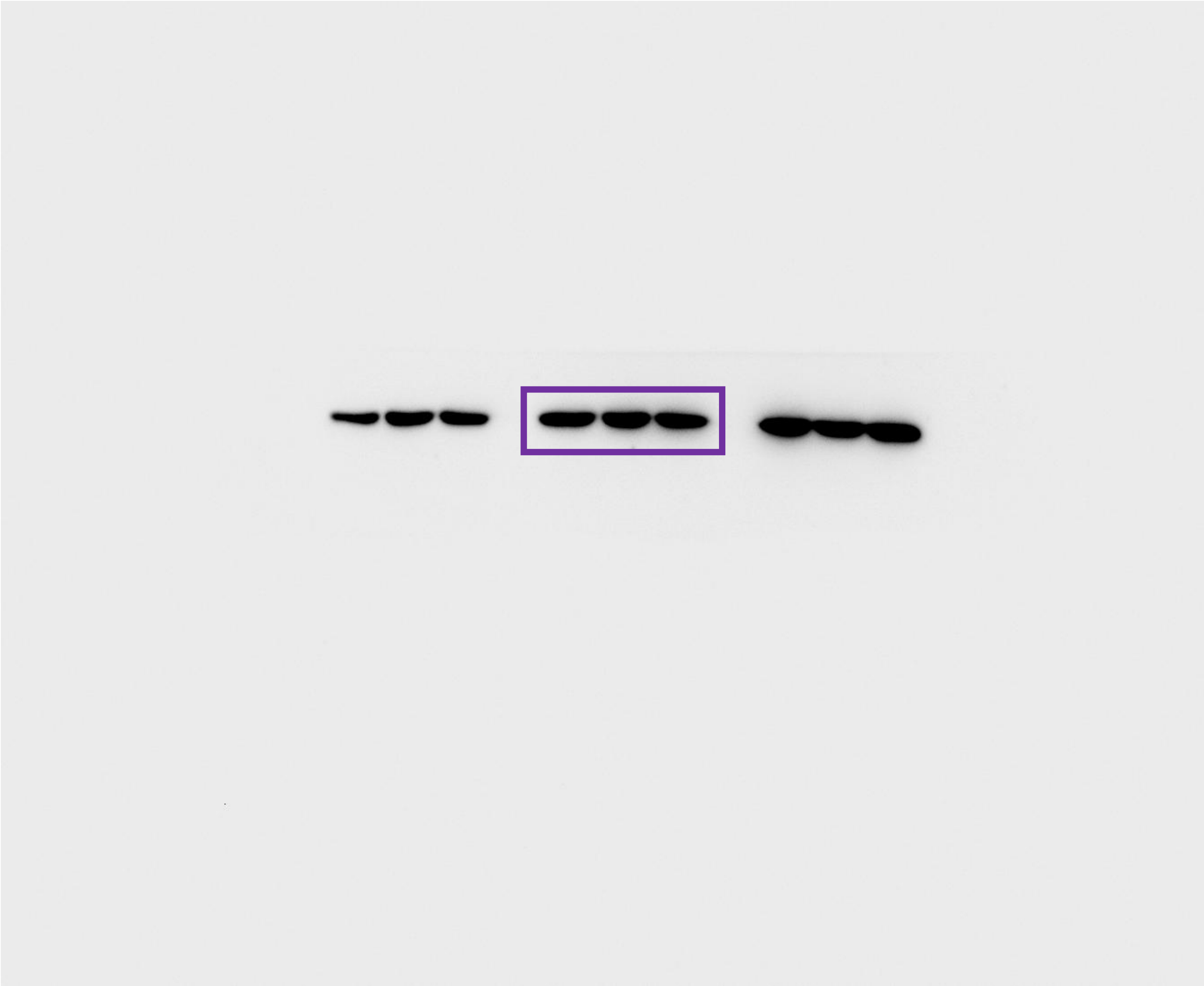

Figure 2D
MHCC97-H FLNA

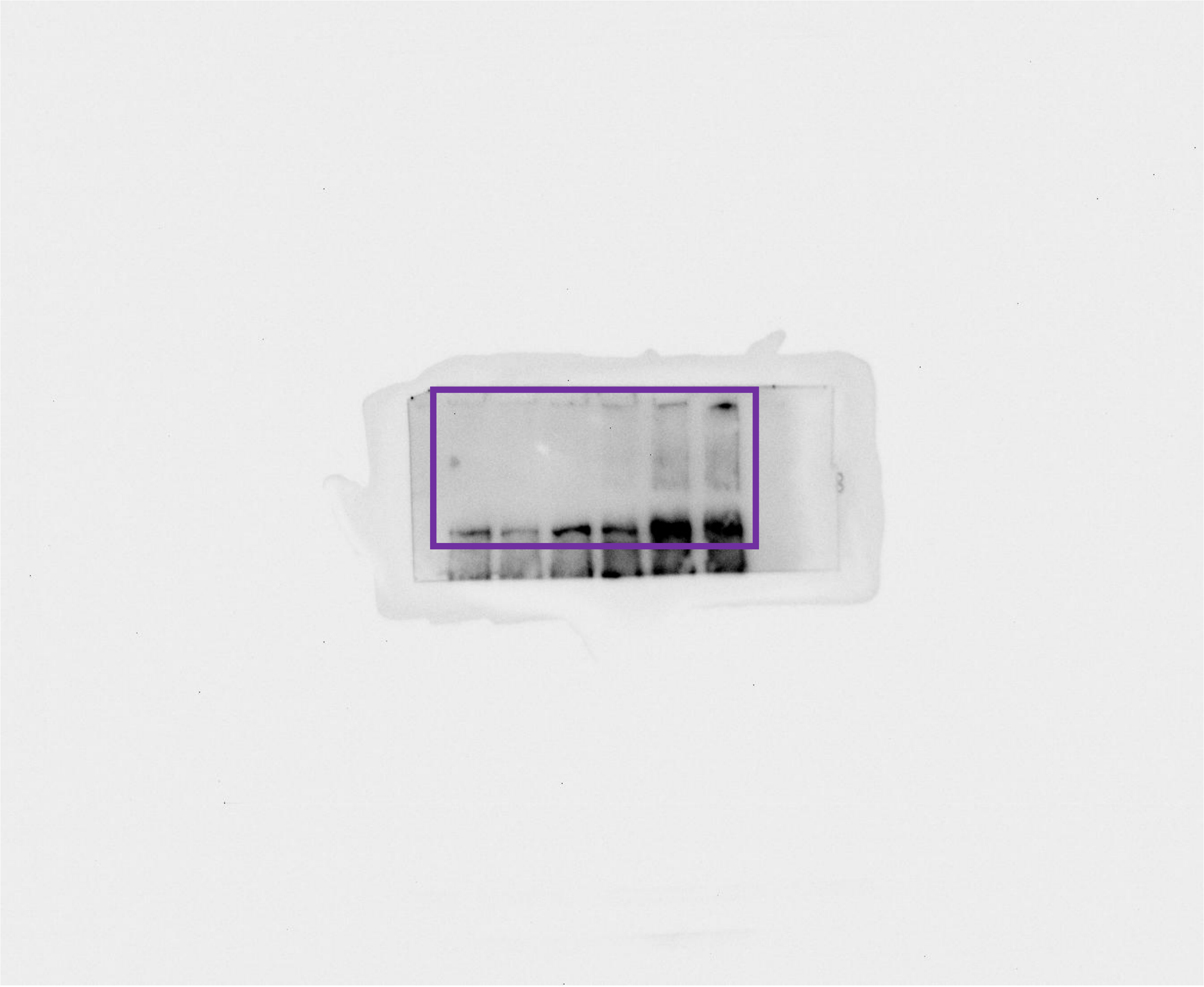

Figure 2D
MHCC97-H DREBRIN

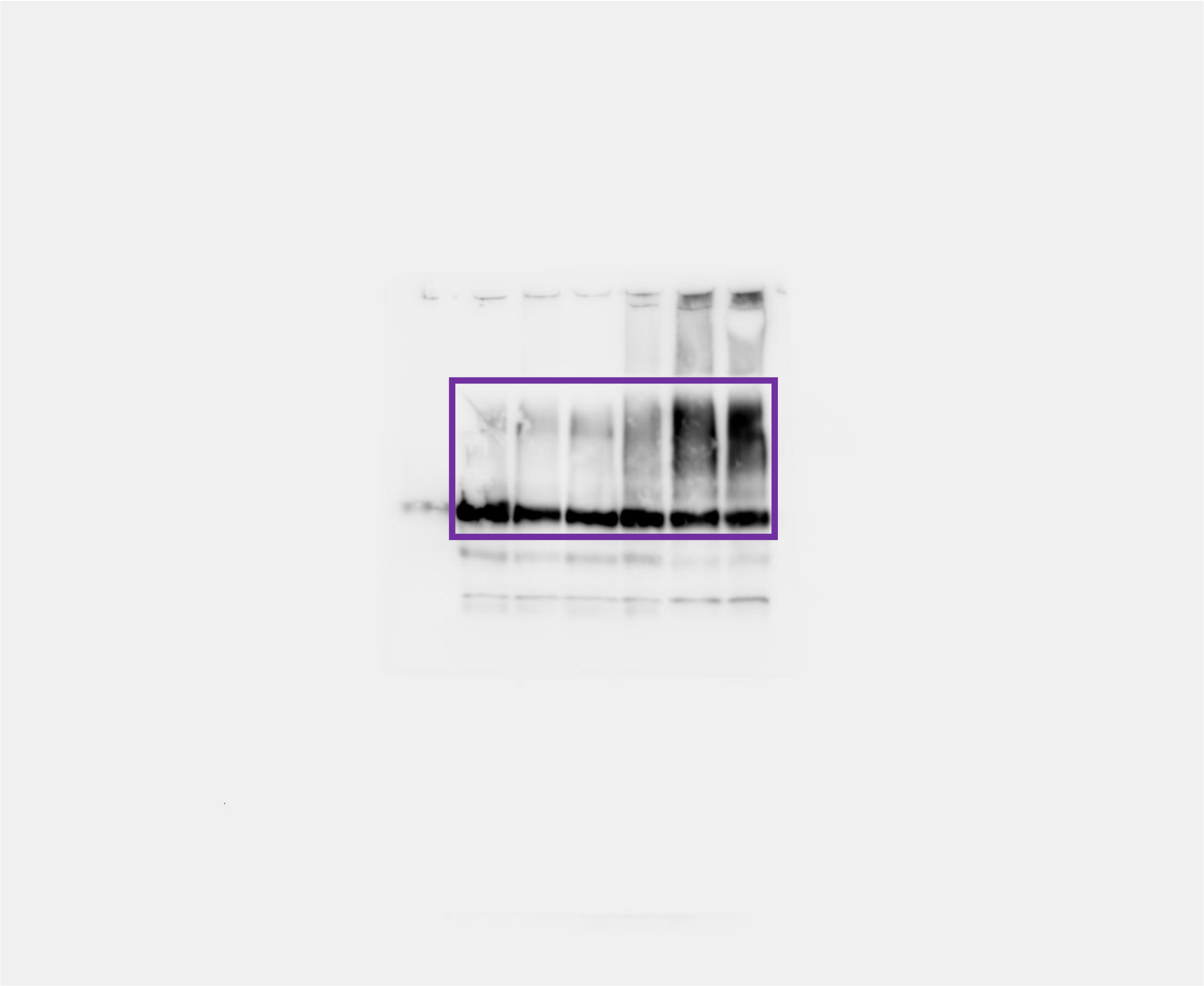

Figure 2D
MHCC97-H GAPDH

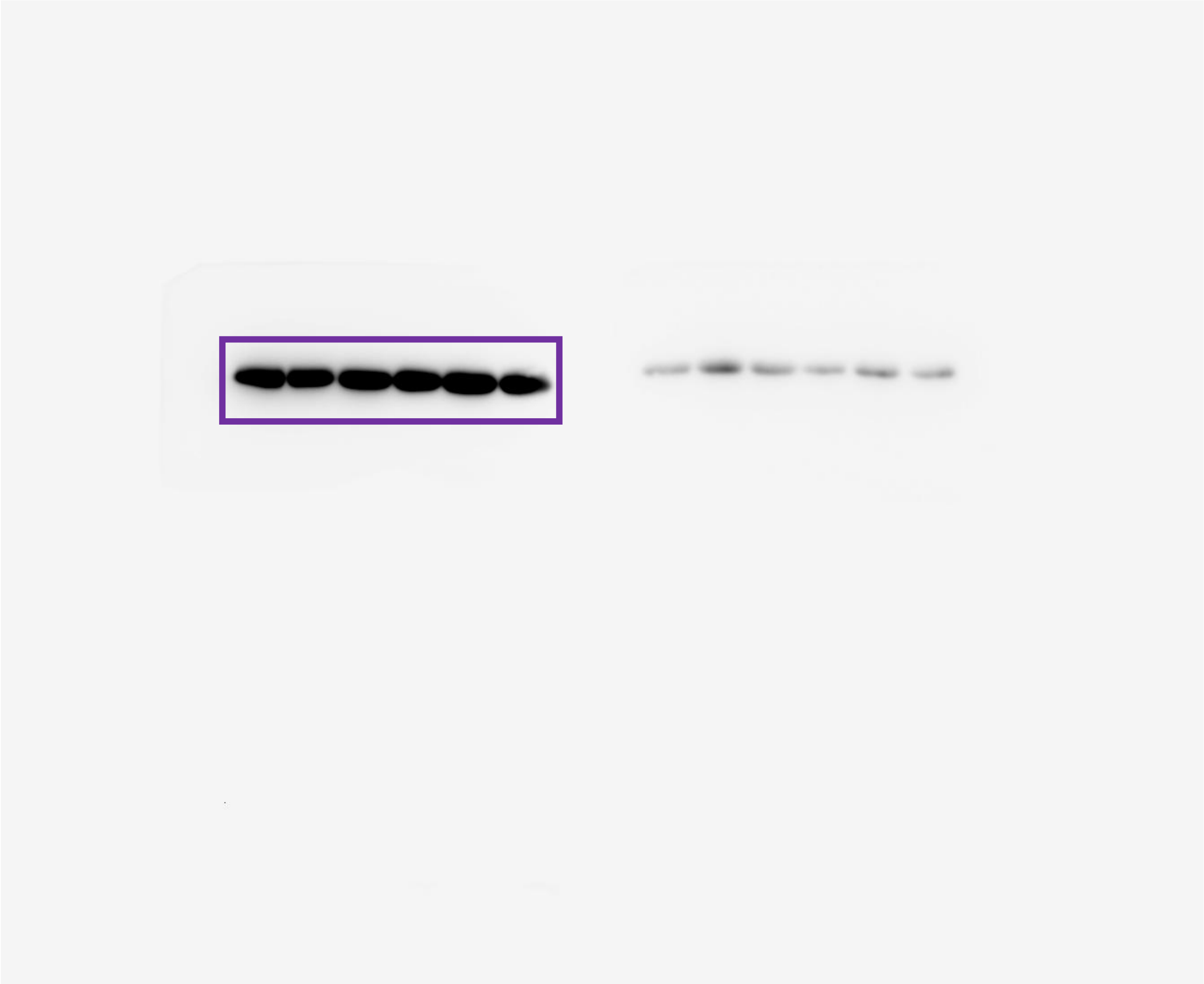

Figure 2D
Hep 3B FLNA

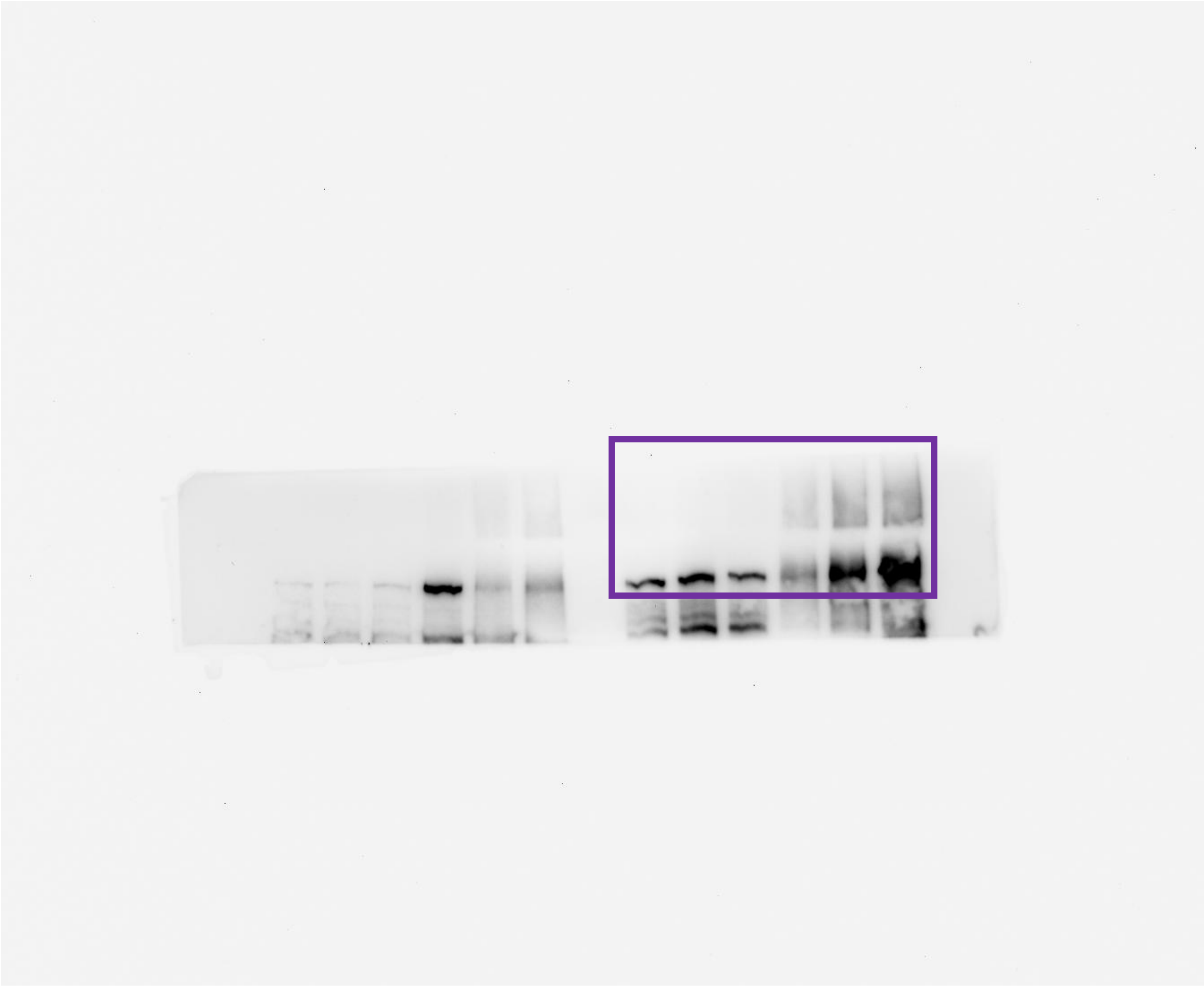

Figure 2D
Hep 3B DREBRIN

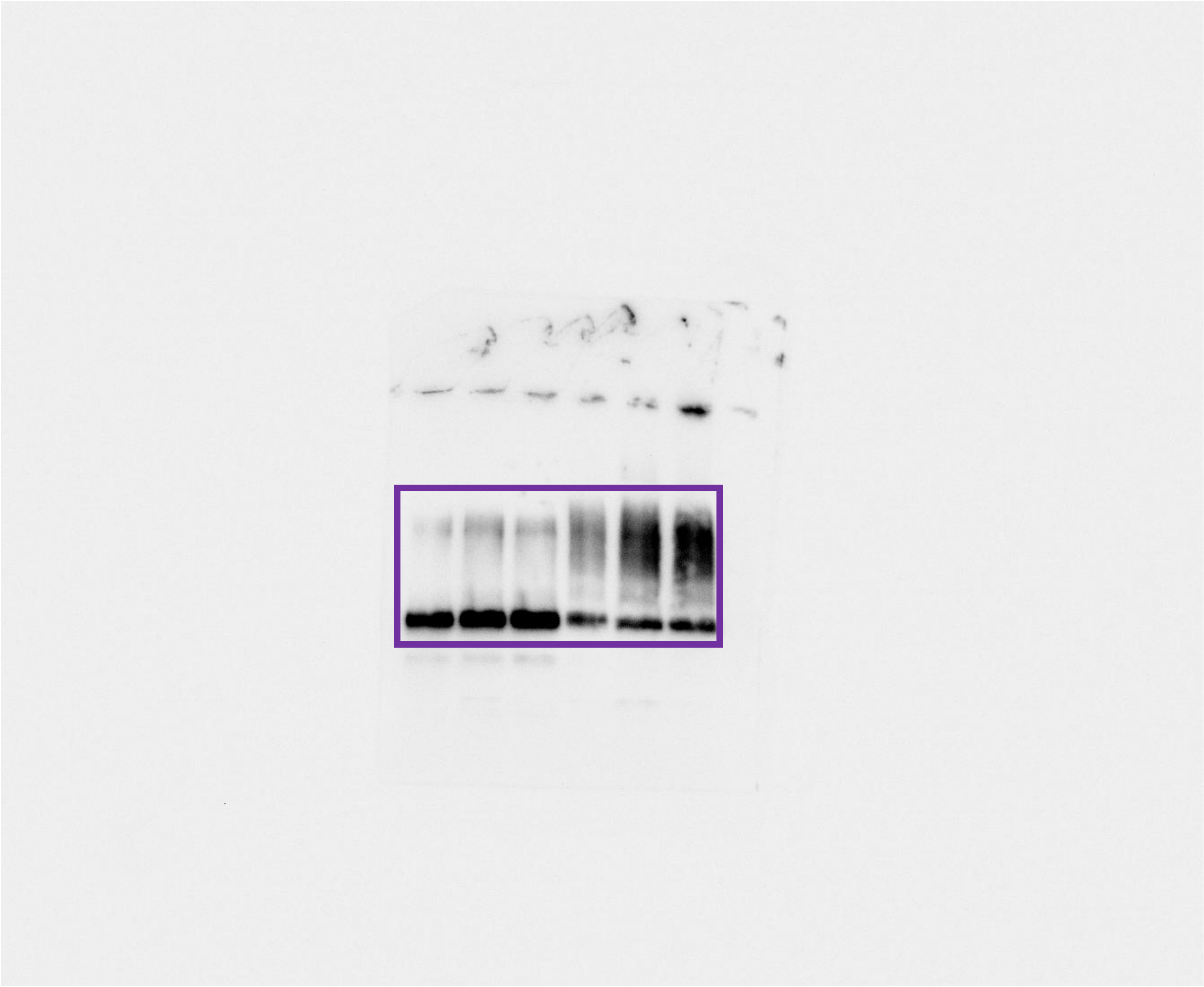

Figure 2D
Hep 3B GAPDH

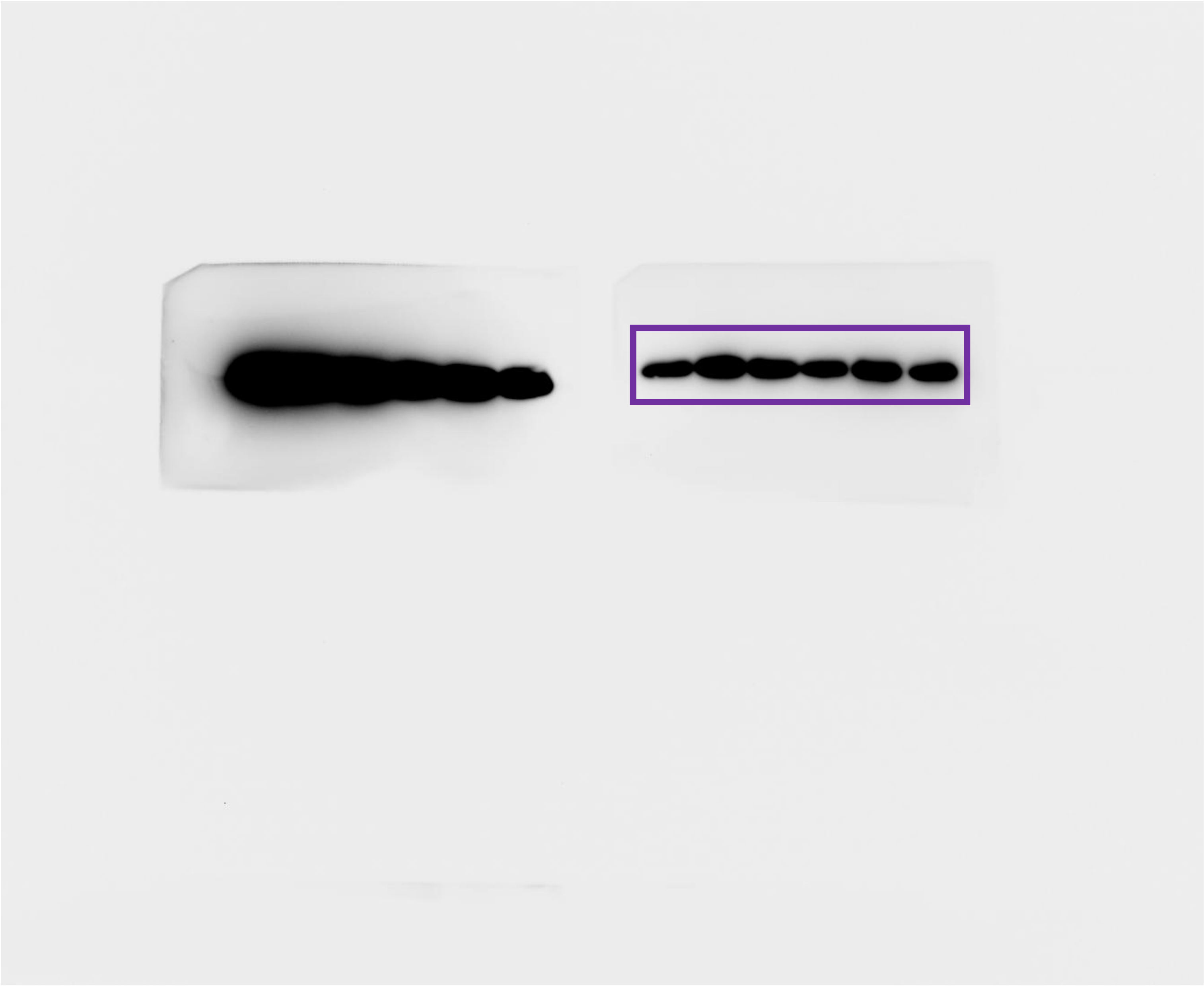

Figure 3A
MHCC97-H APN G6PD

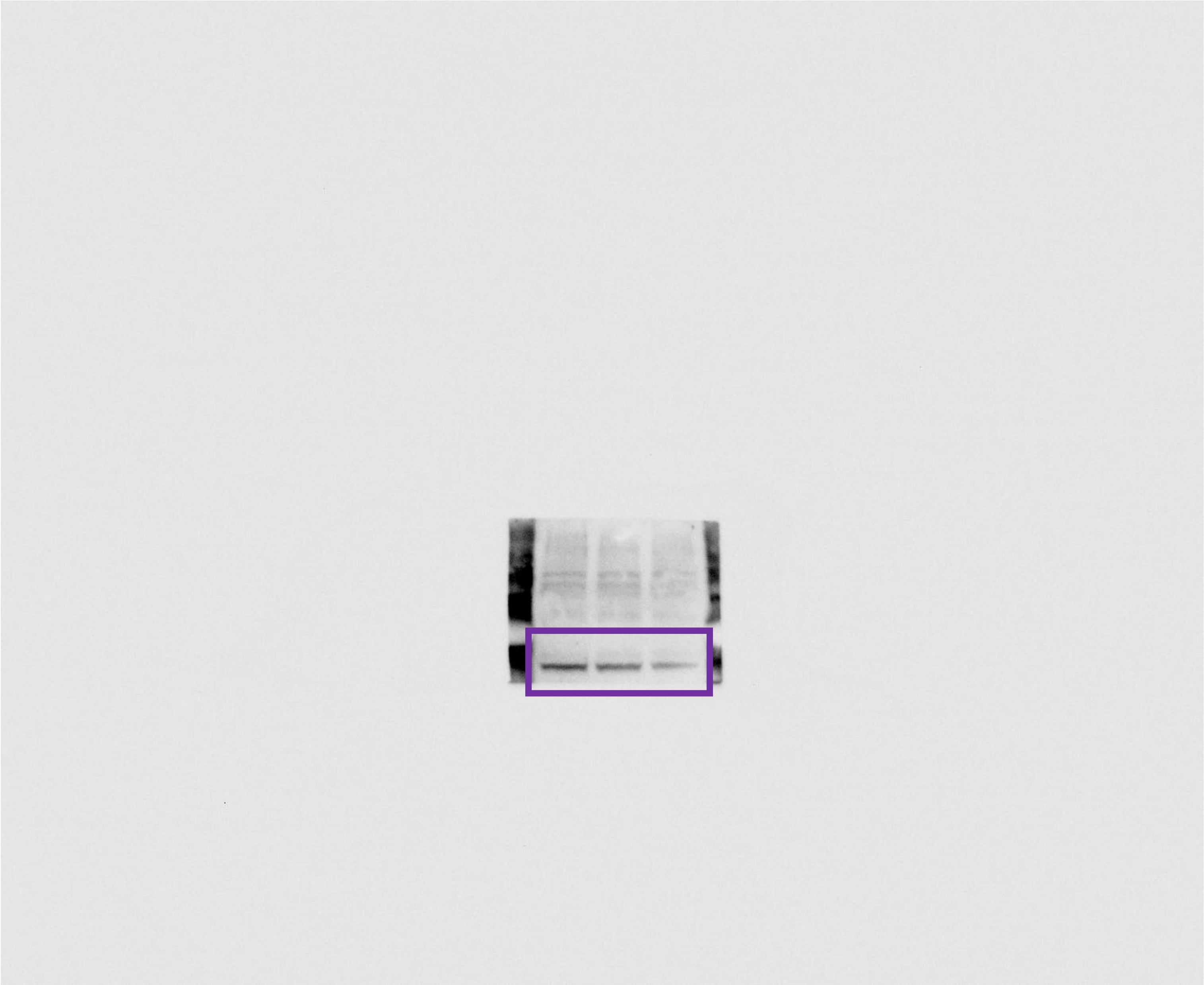

Figure 3A
MHCC97-H APN LDHA

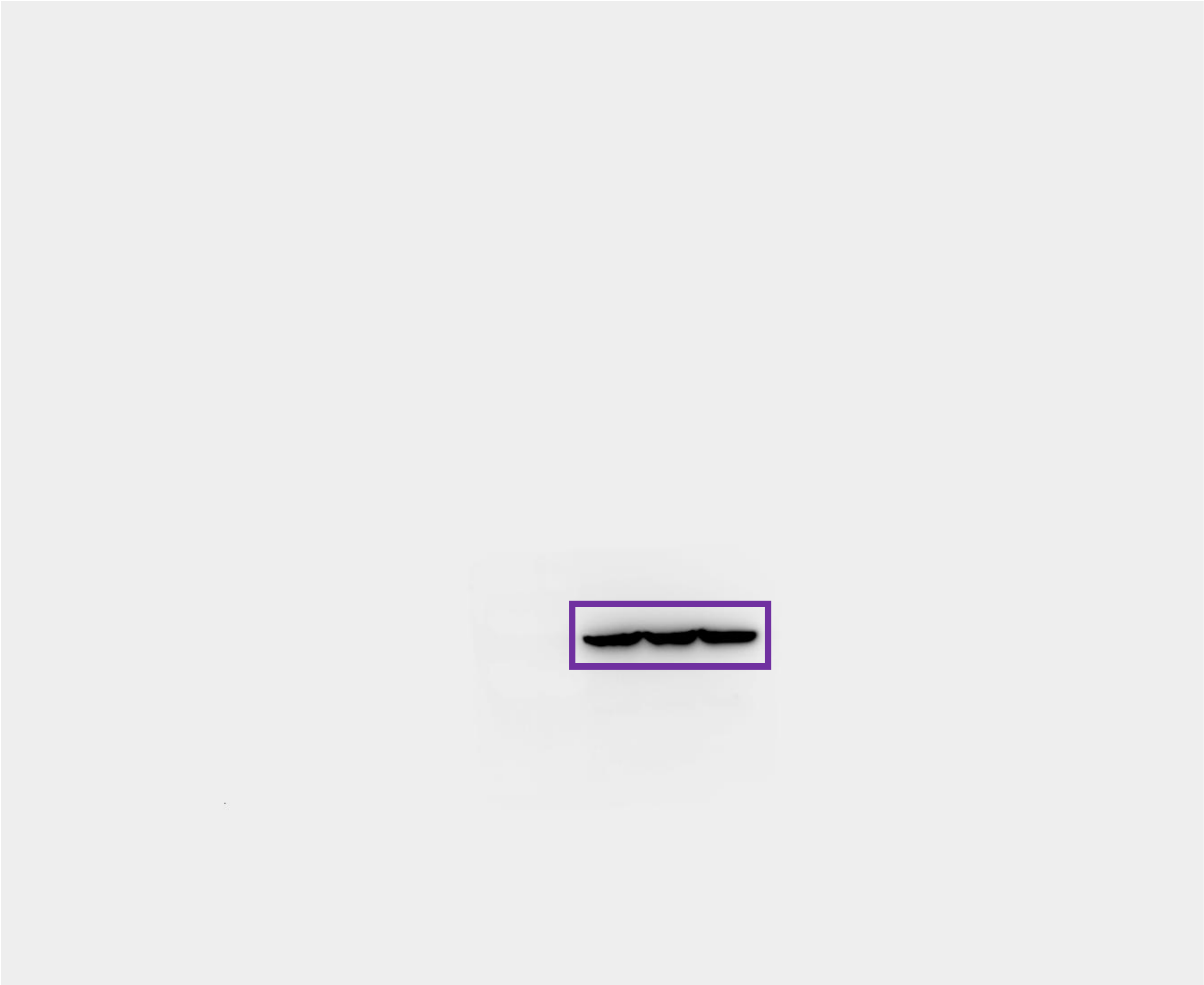

Figure 3A

MHCC97-H APN MCT4

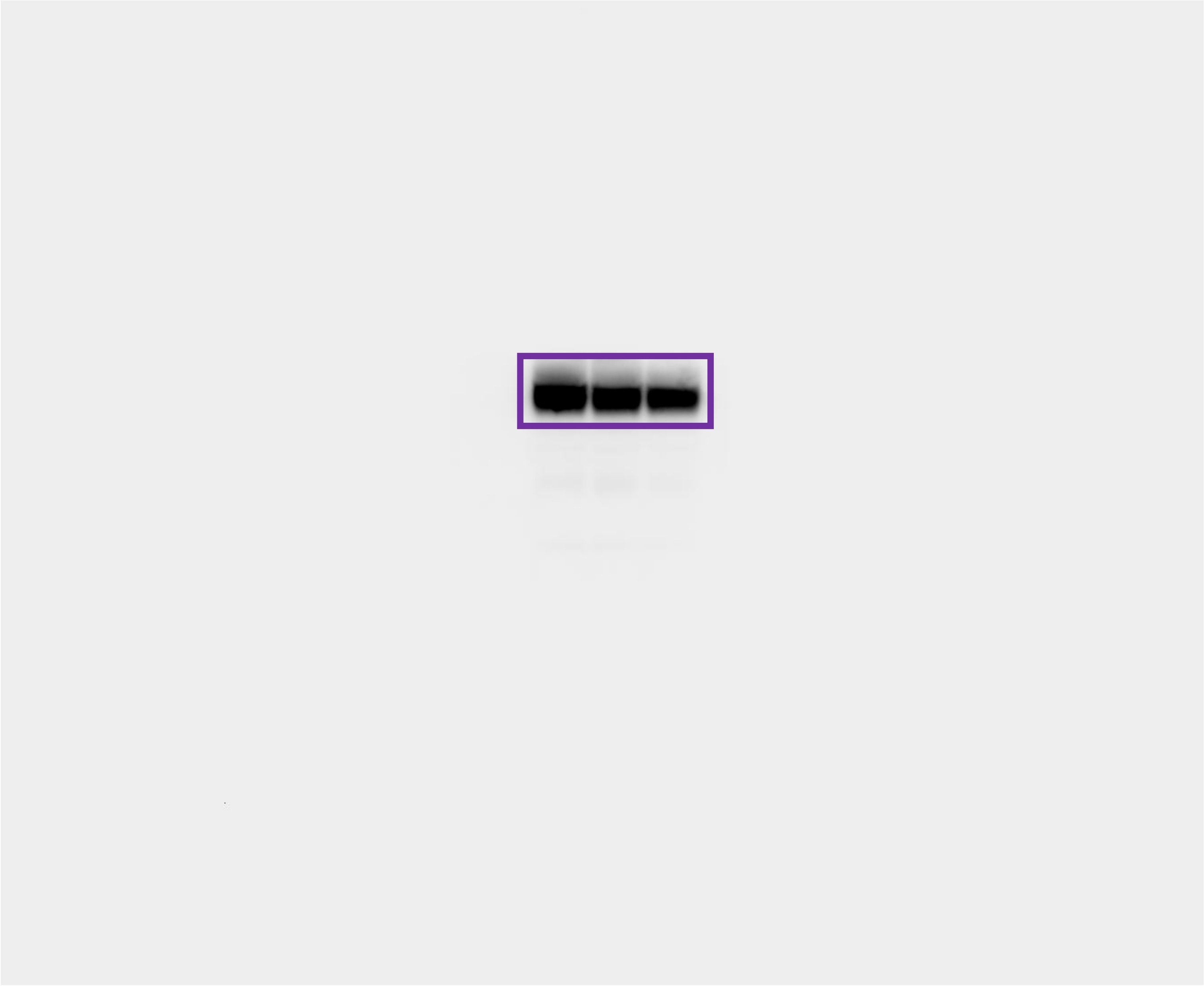

Figure 3A

MHCC97-H APN GAPDH

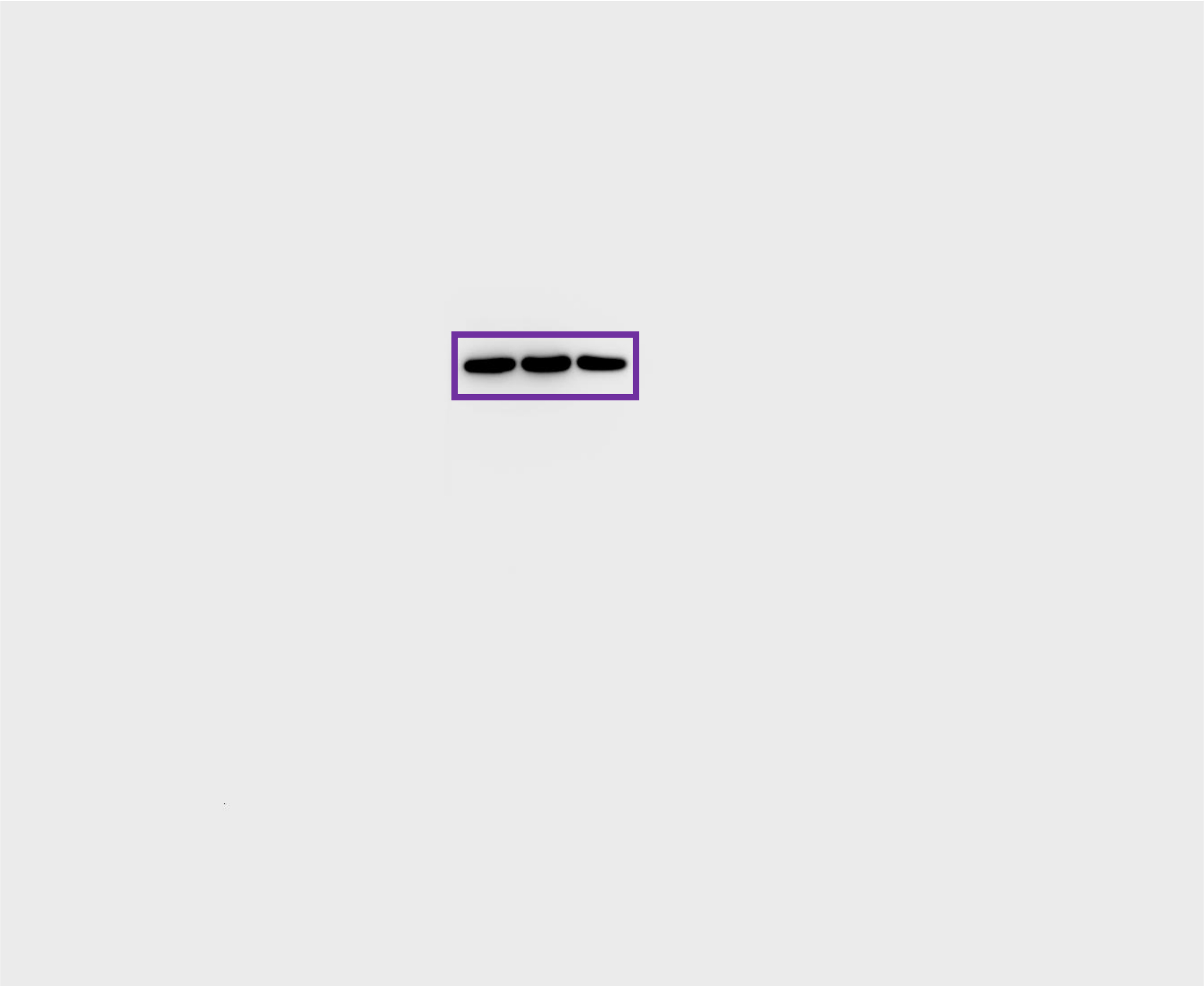

Figure 3A
MHCC97-H CQ G6PD

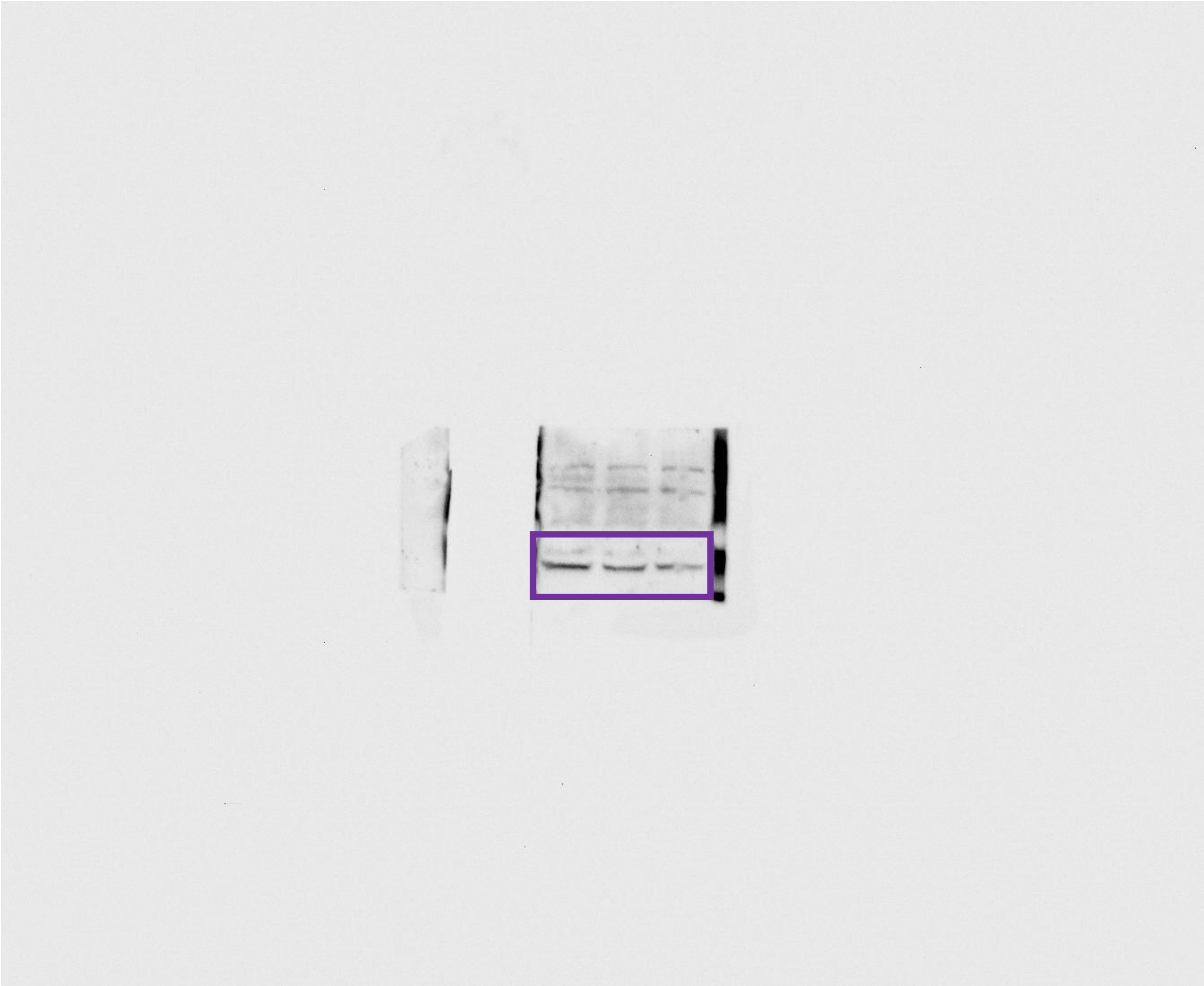

Figure 3A
MHCC97-H CQ LDHA

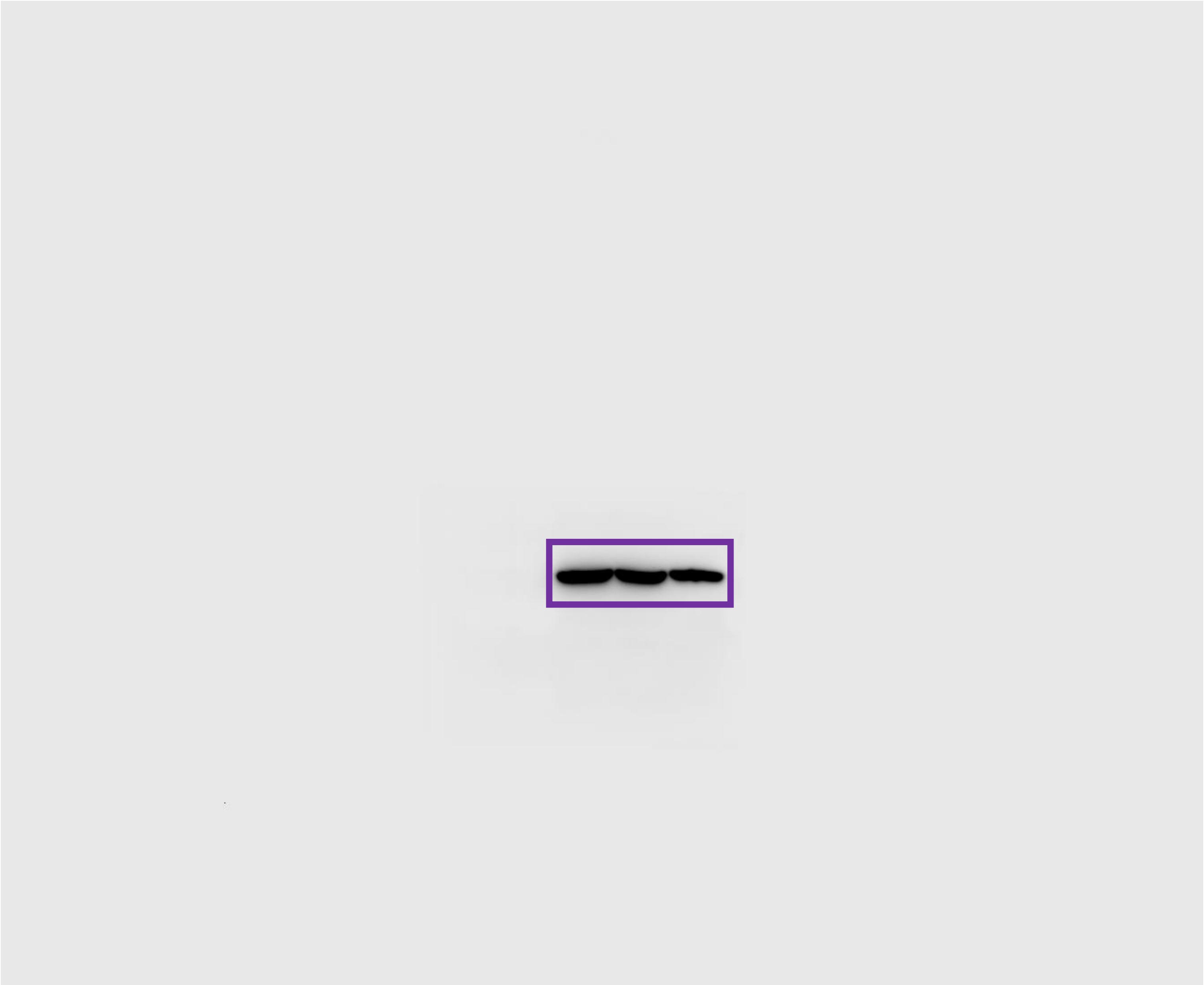

Figure 3A
MHCC97-H CQ MCT4

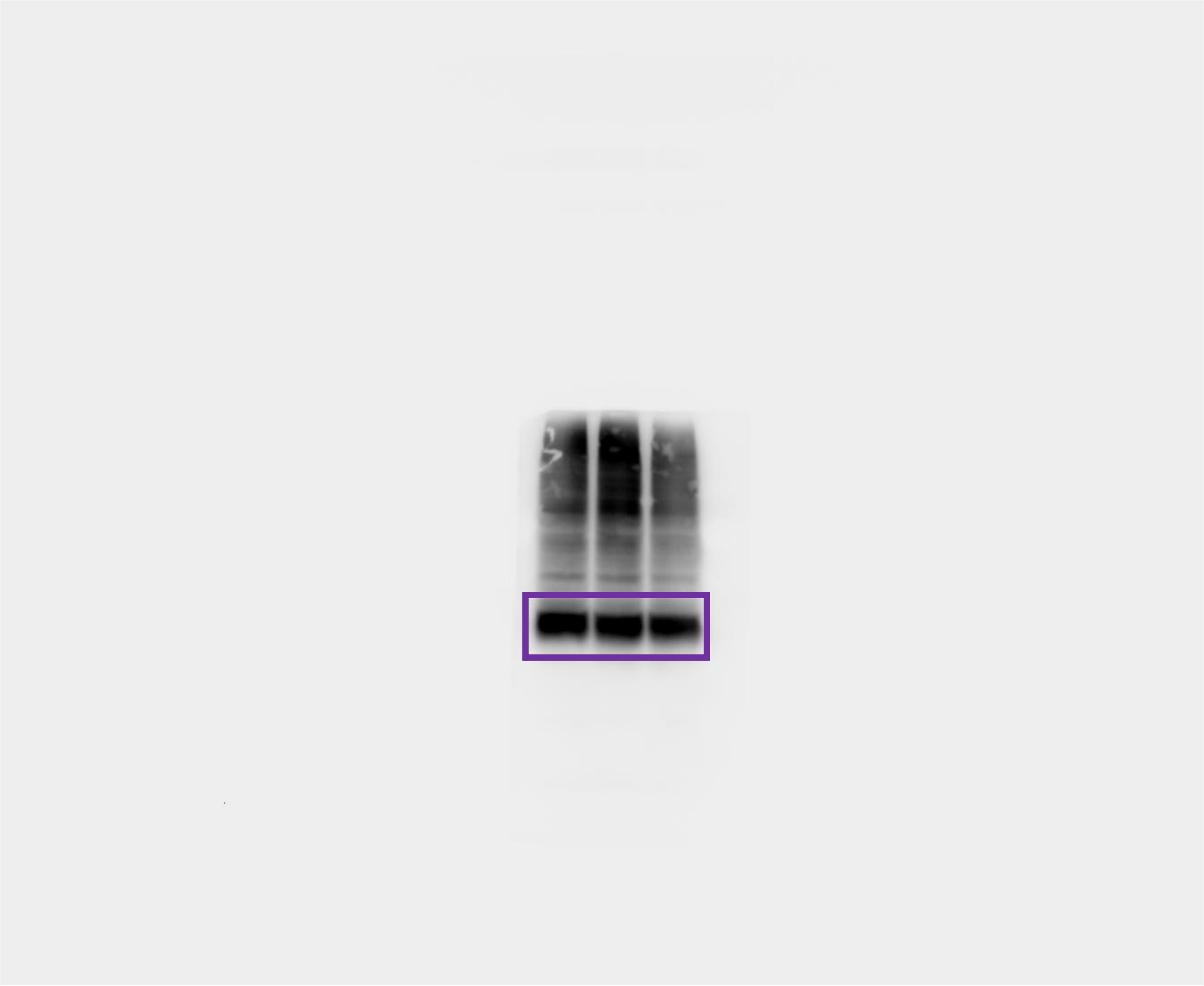

Figure 3A
MHCC97-H CQ GAPDH

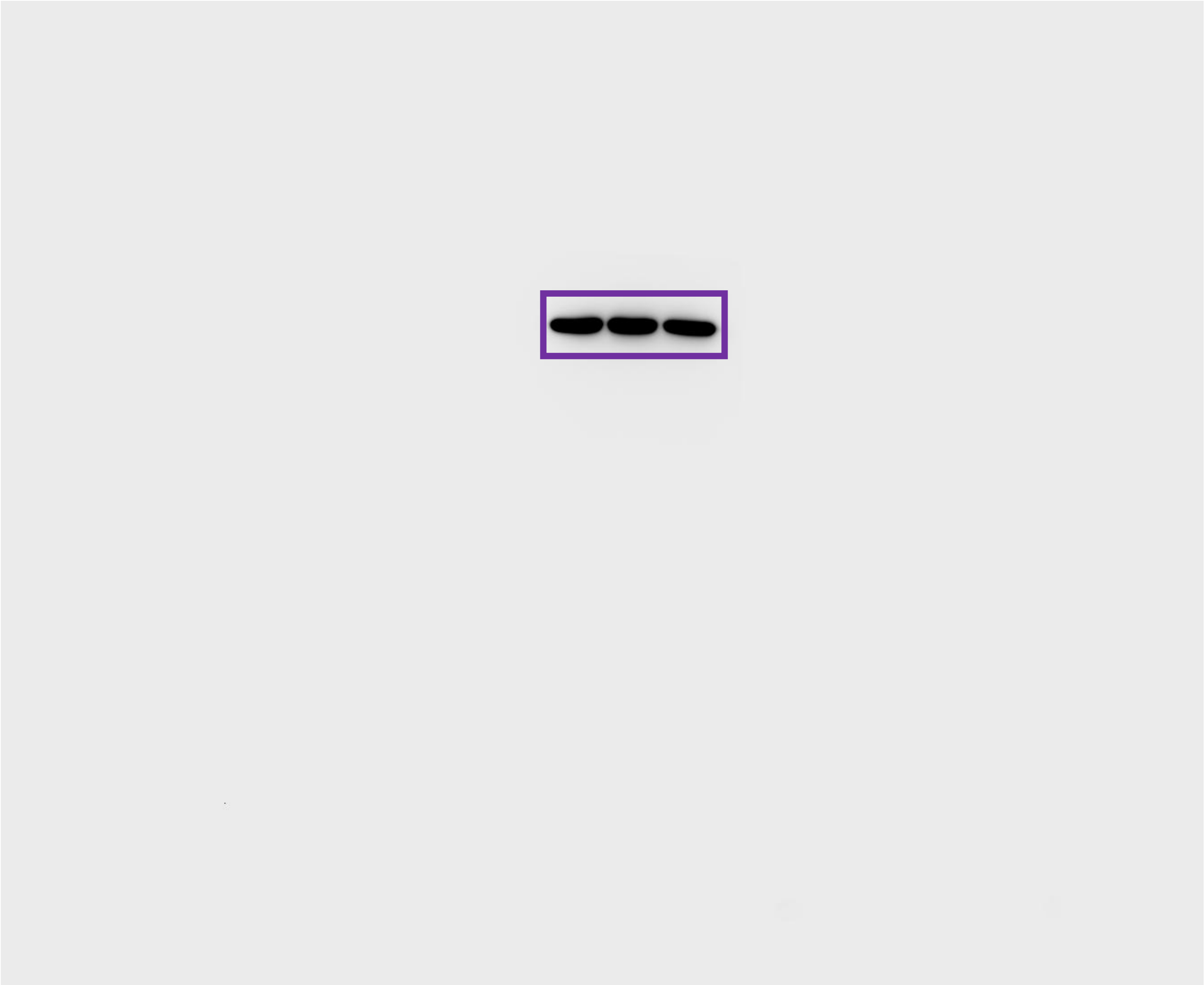

Figure 3B
Hep 3B APN G6PD

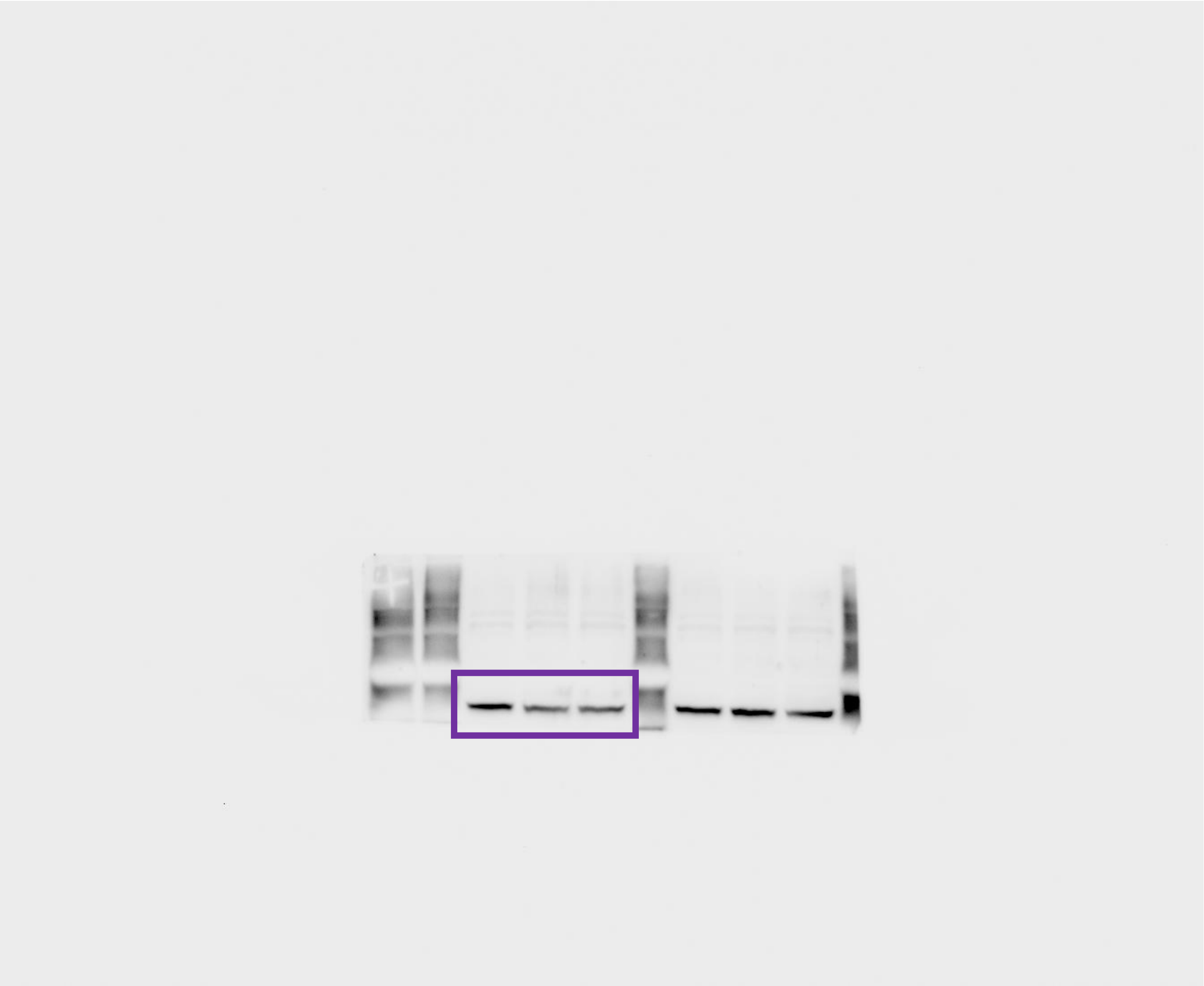

Figure 3B
Hep 3B APN LDHA

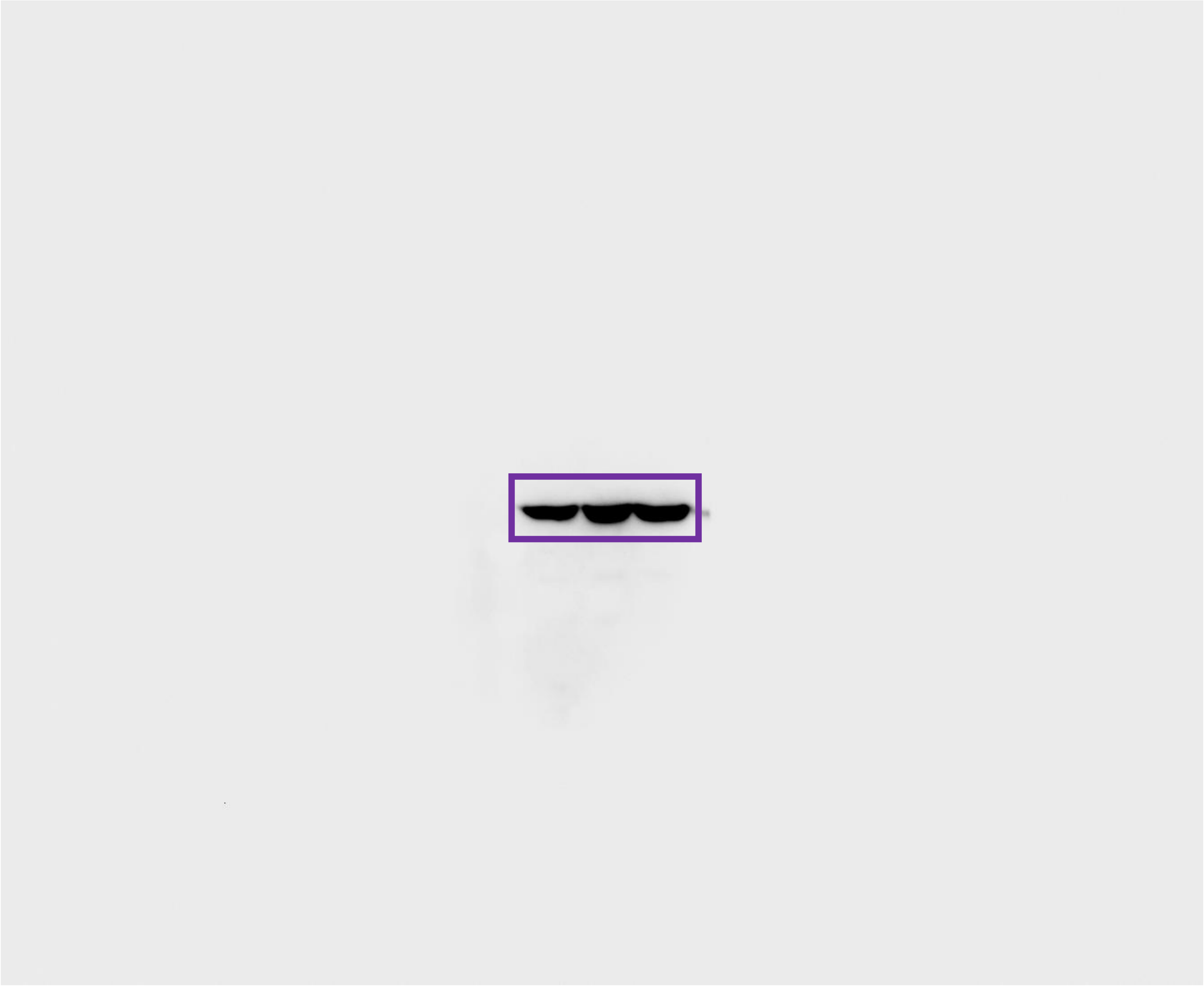

Figure 3B
Hep 3B APN MCT4

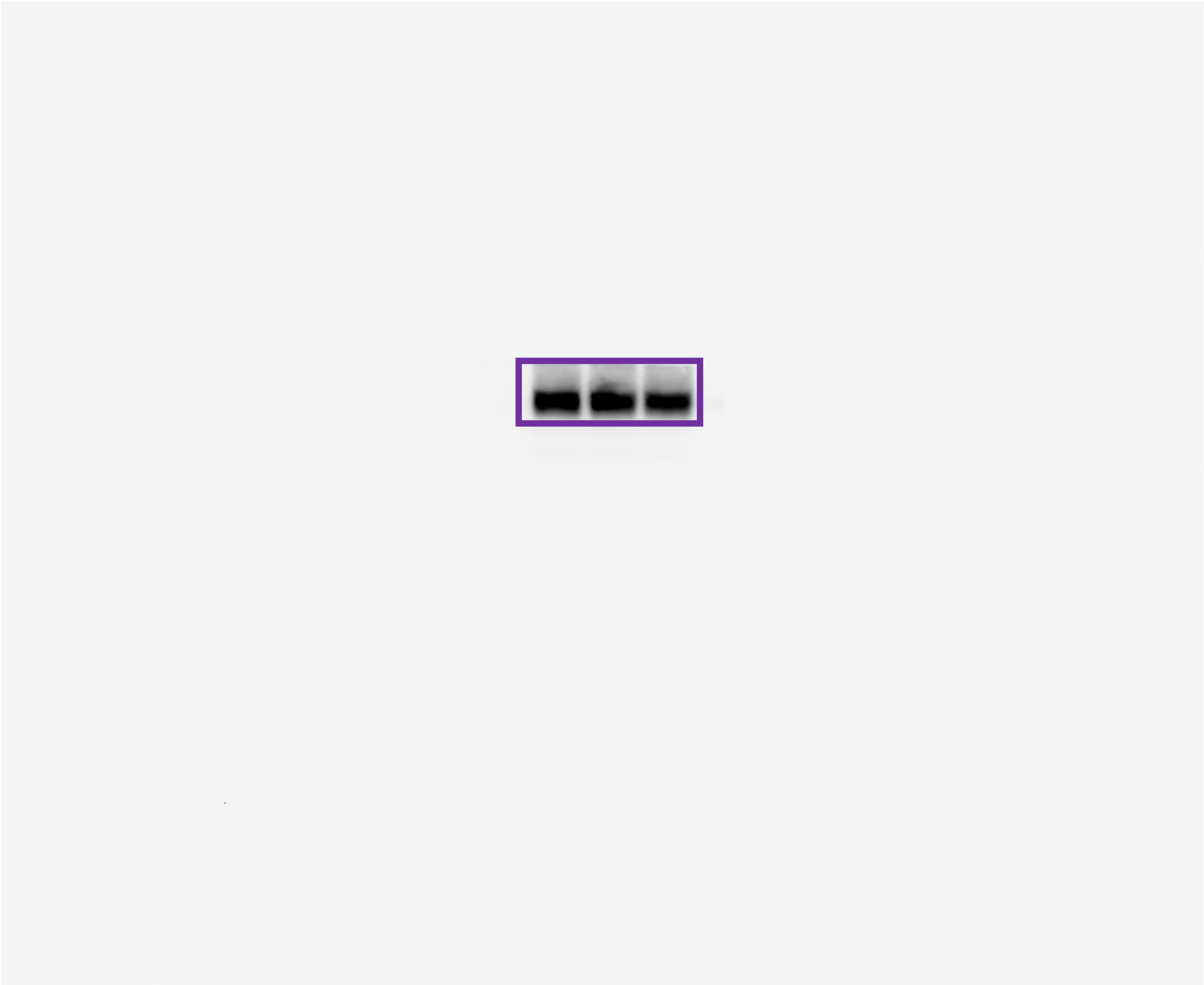

Figure 3B
Hep 3B APN GAPDH

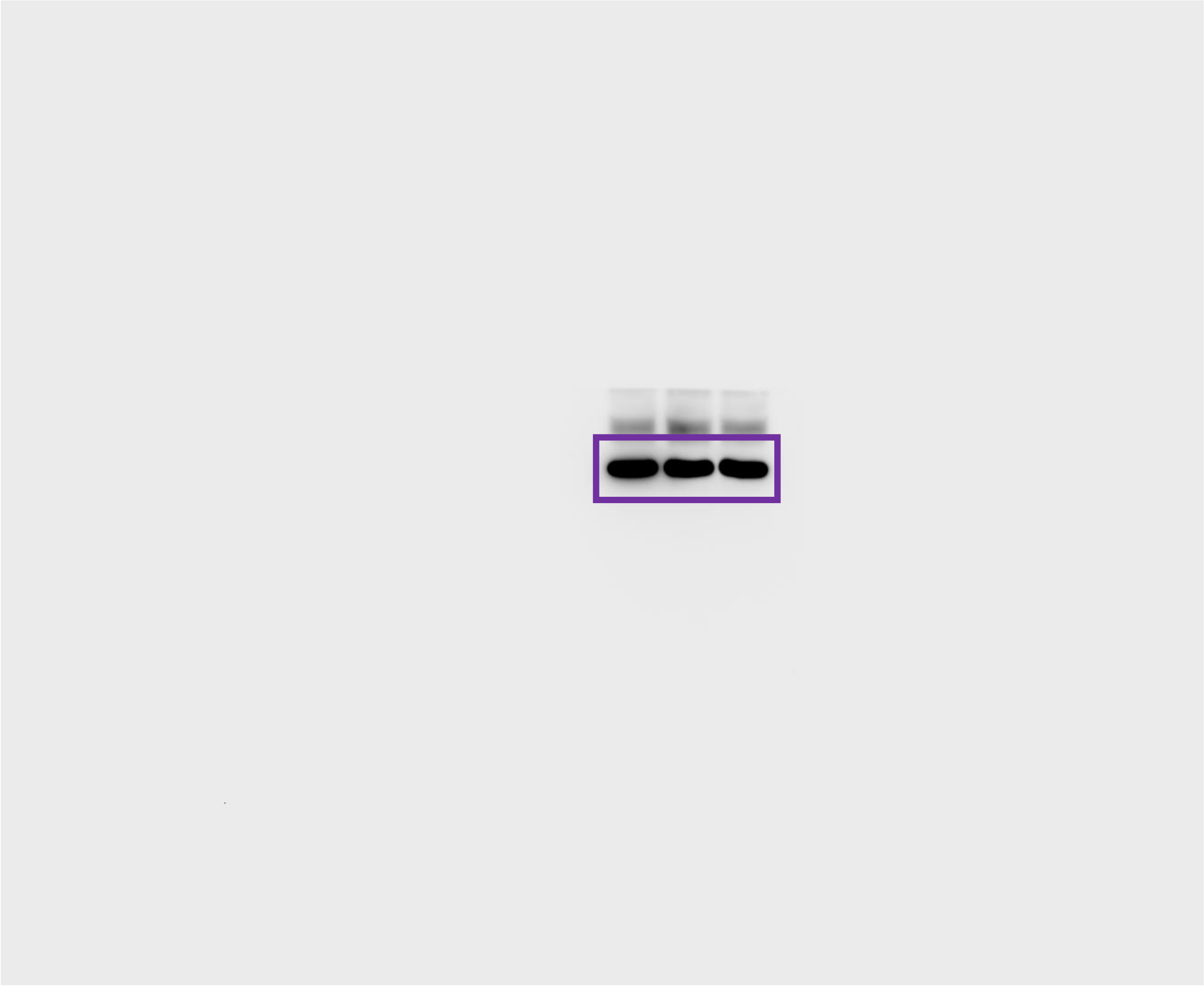

Figure 3B
Hep 3B CQ G6PD

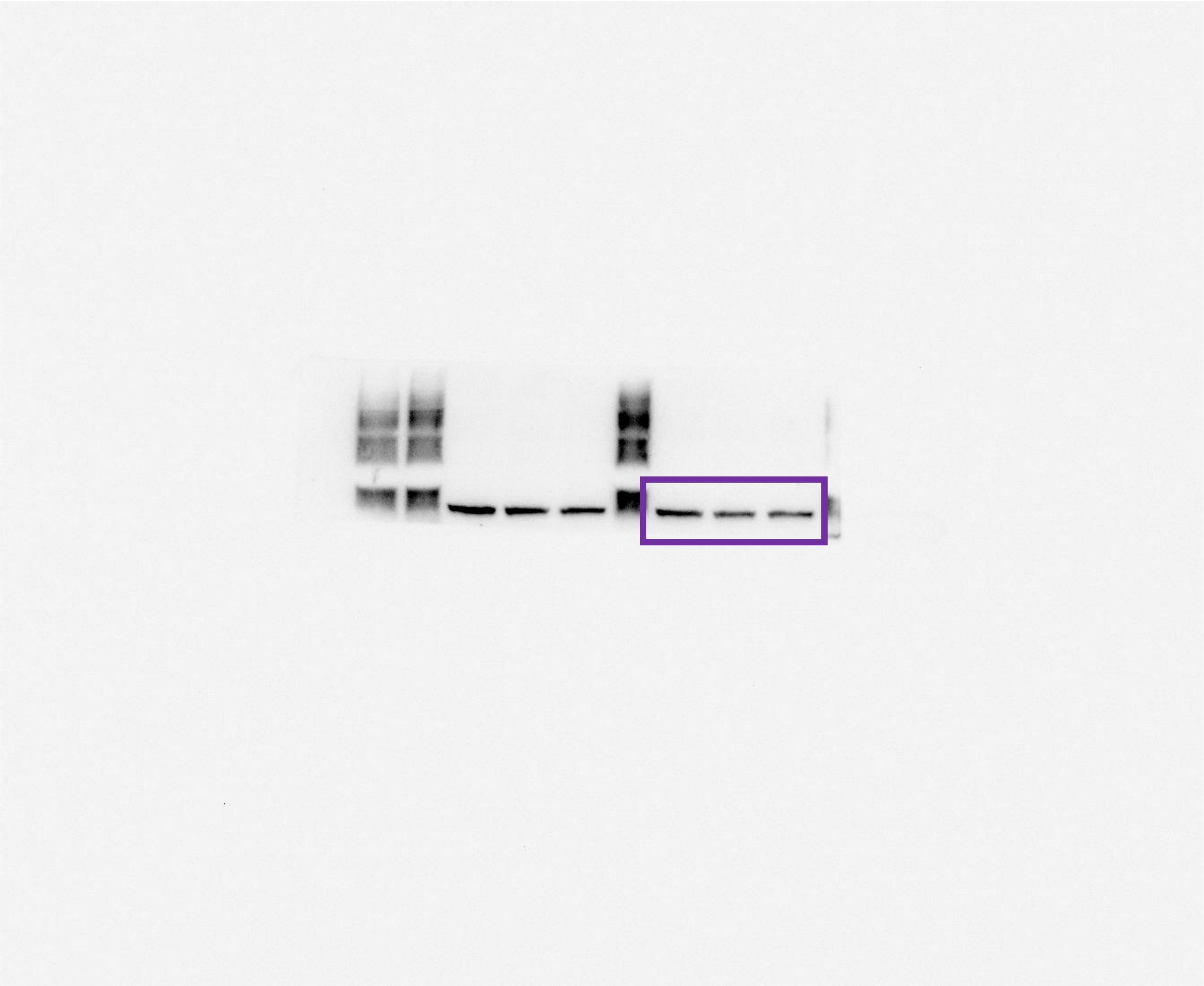

Figure 3B
Hep 3B CQ LDHA

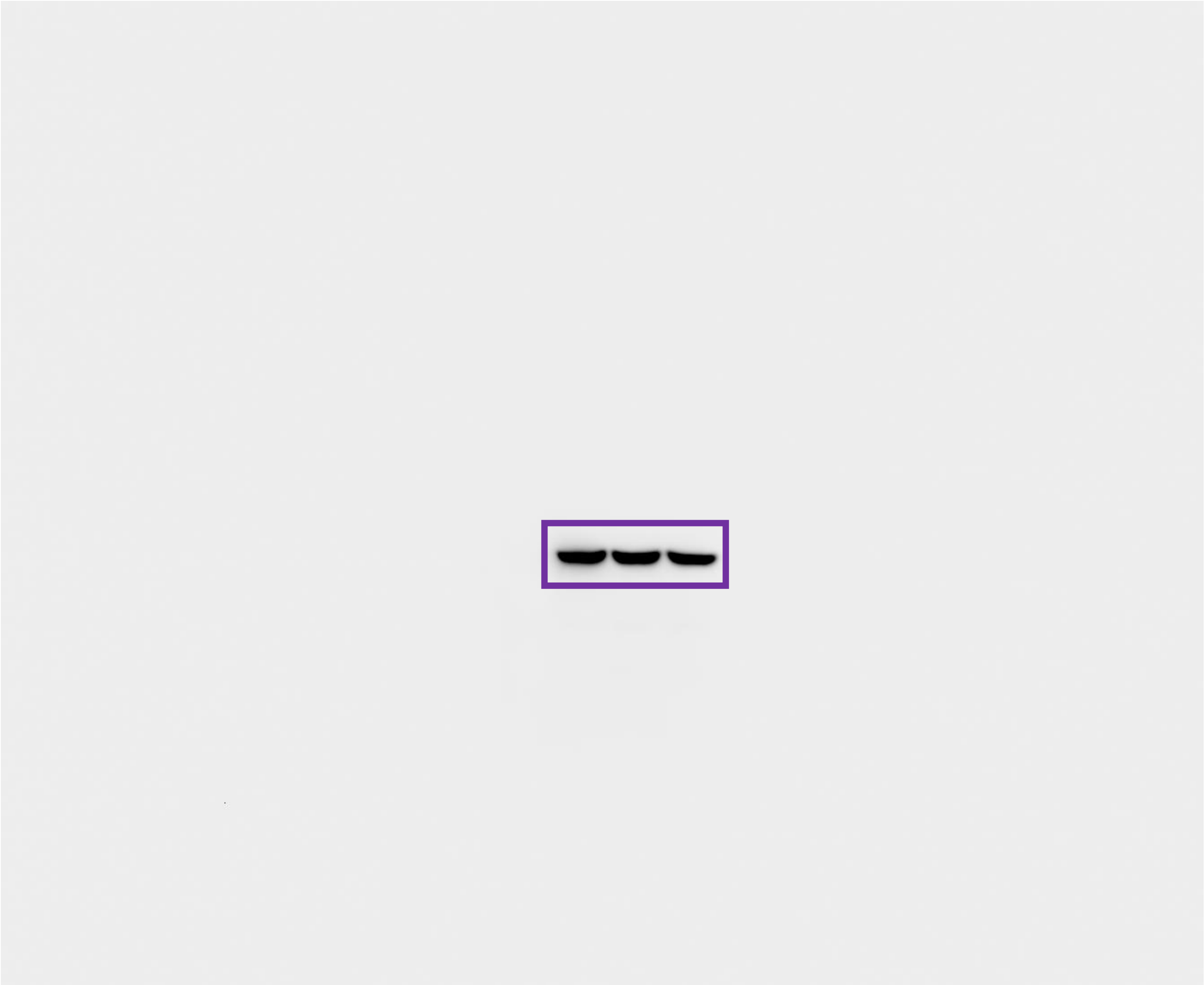

Figure 3B
Hep 3B CQ MCT4

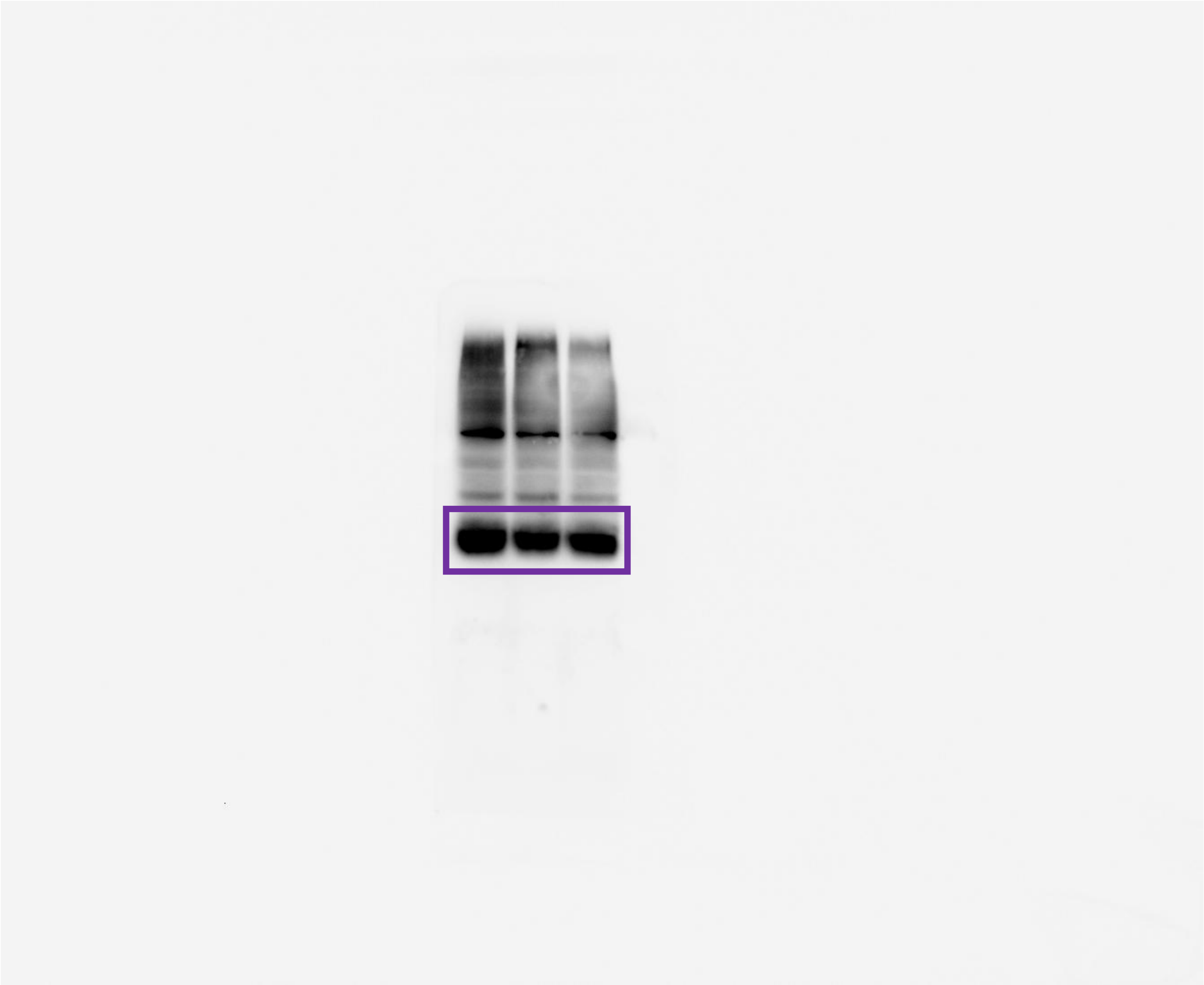

Figure 3B
Hep 3B CQ GAPDH

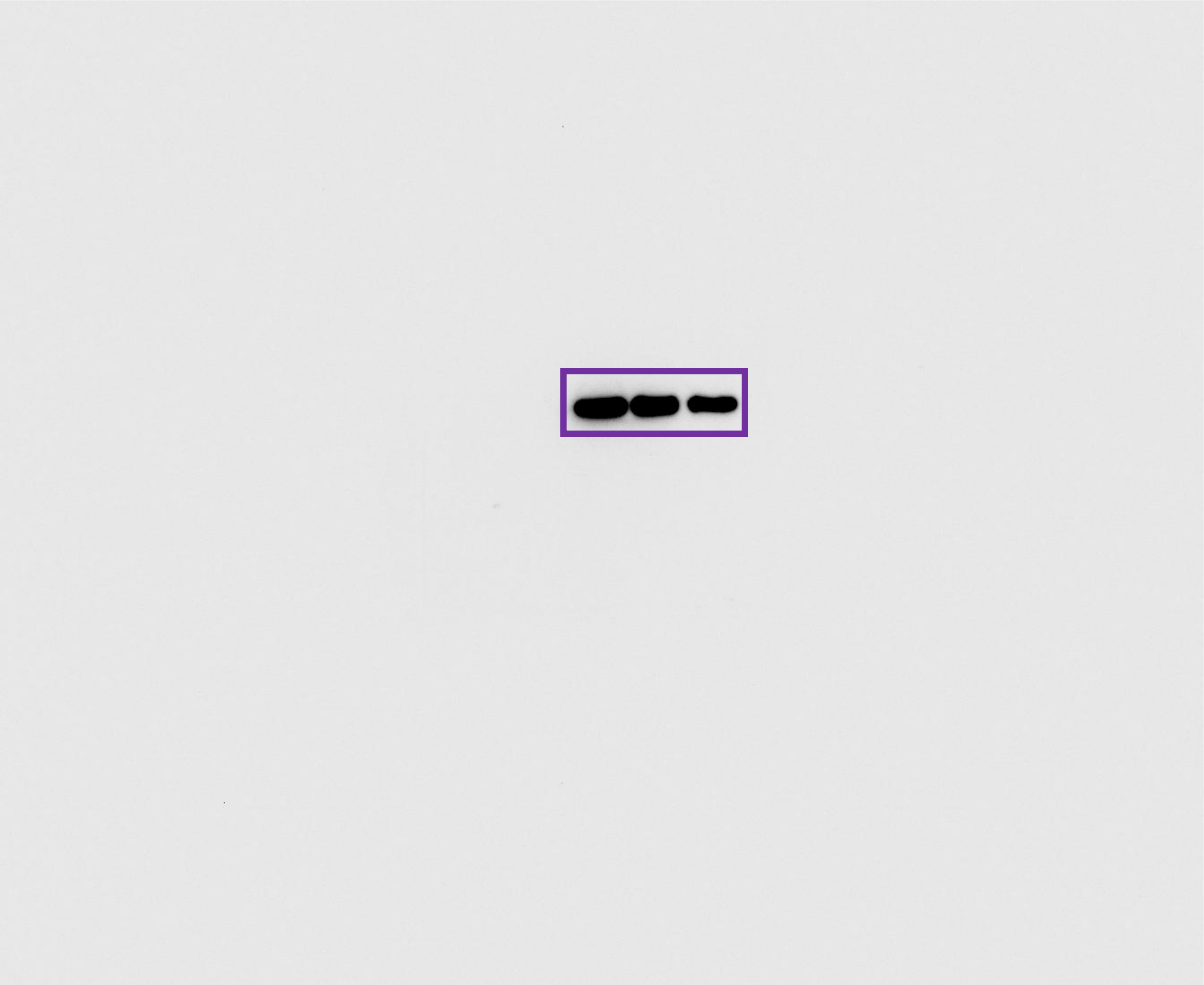

Figure 3C
MHCC97-H G6PD

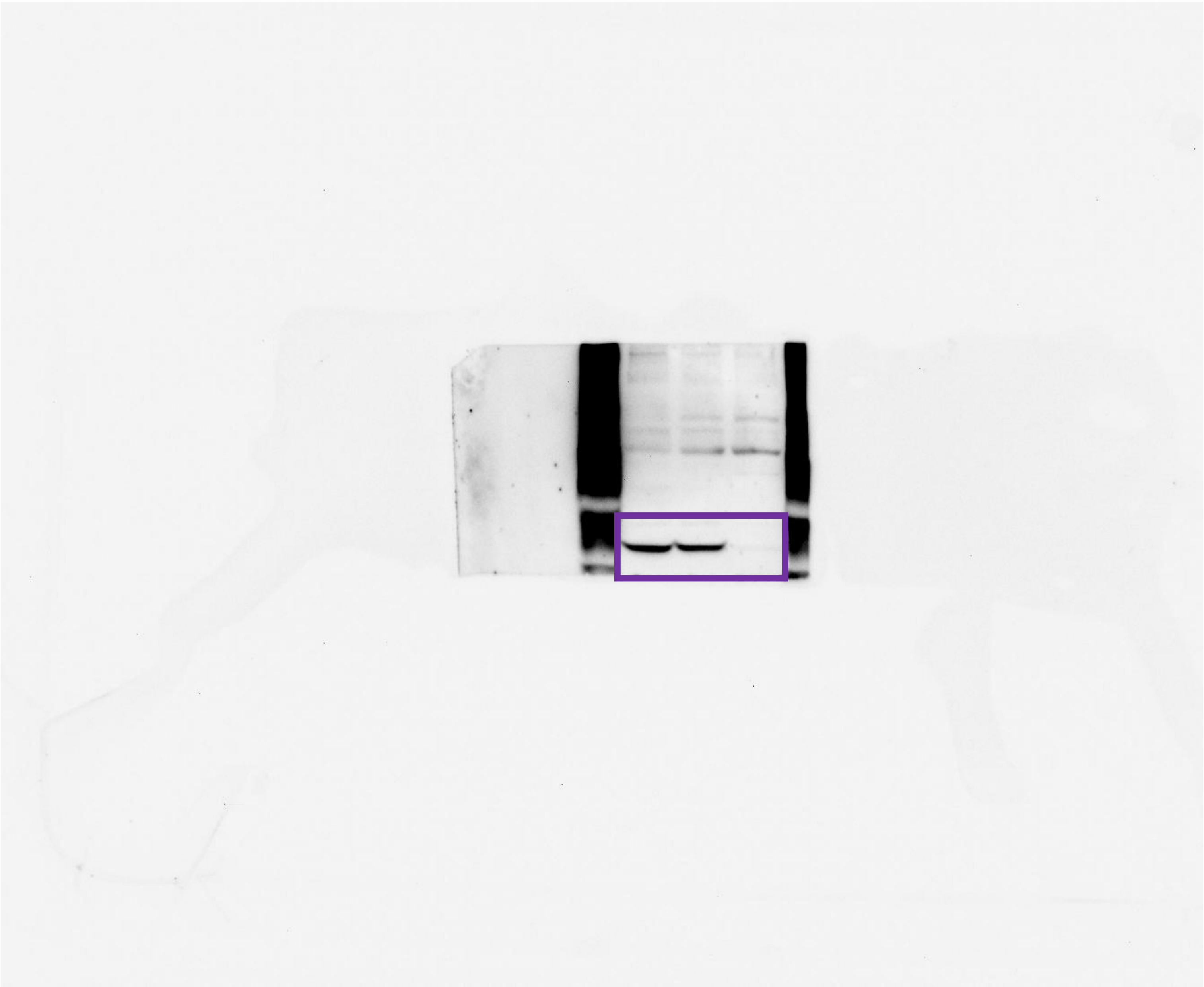

Figure 3D
Hep 3B G6PD

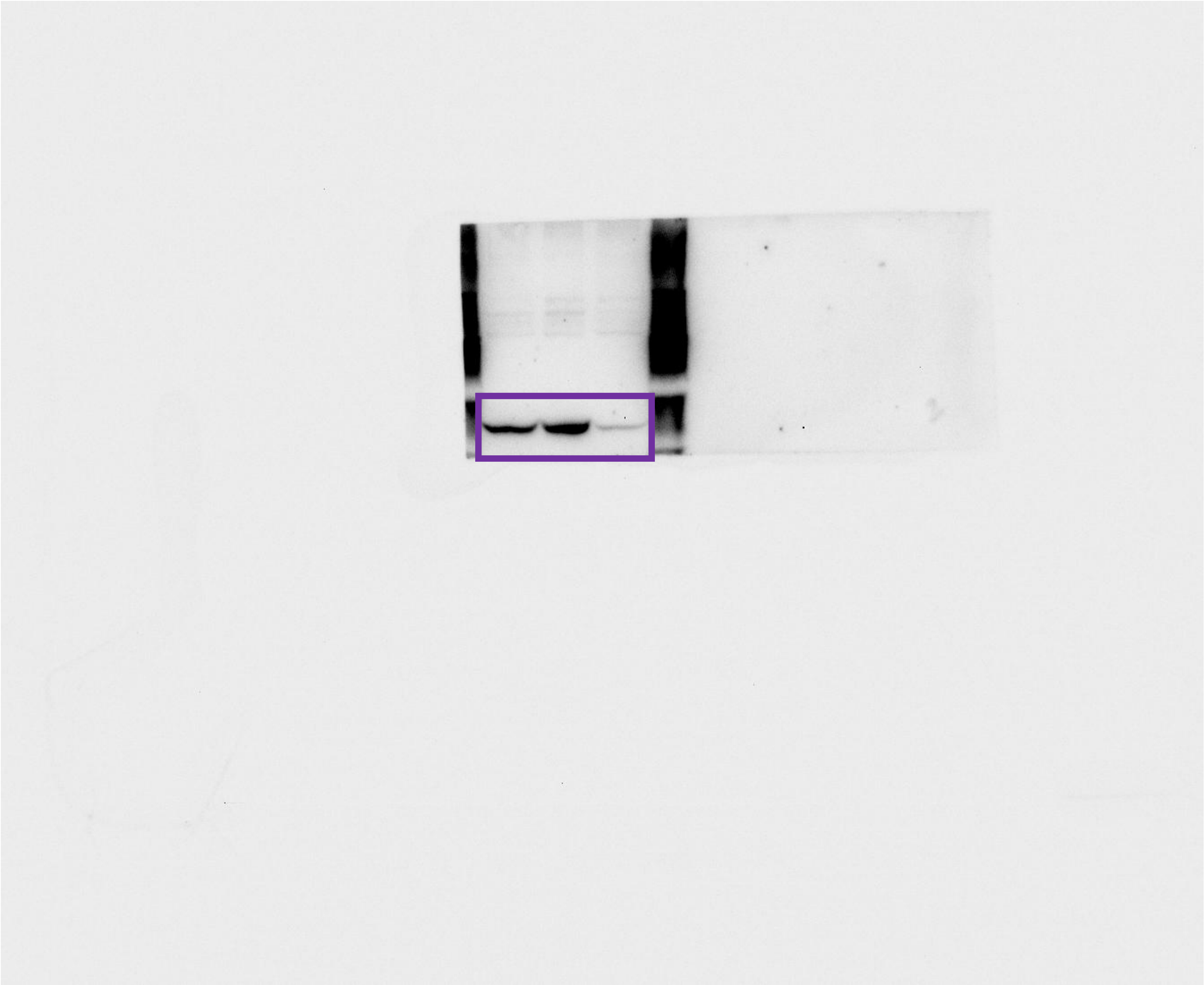

Figure 3C and 3D
MHCC97-H Hep 3B
GAPDH

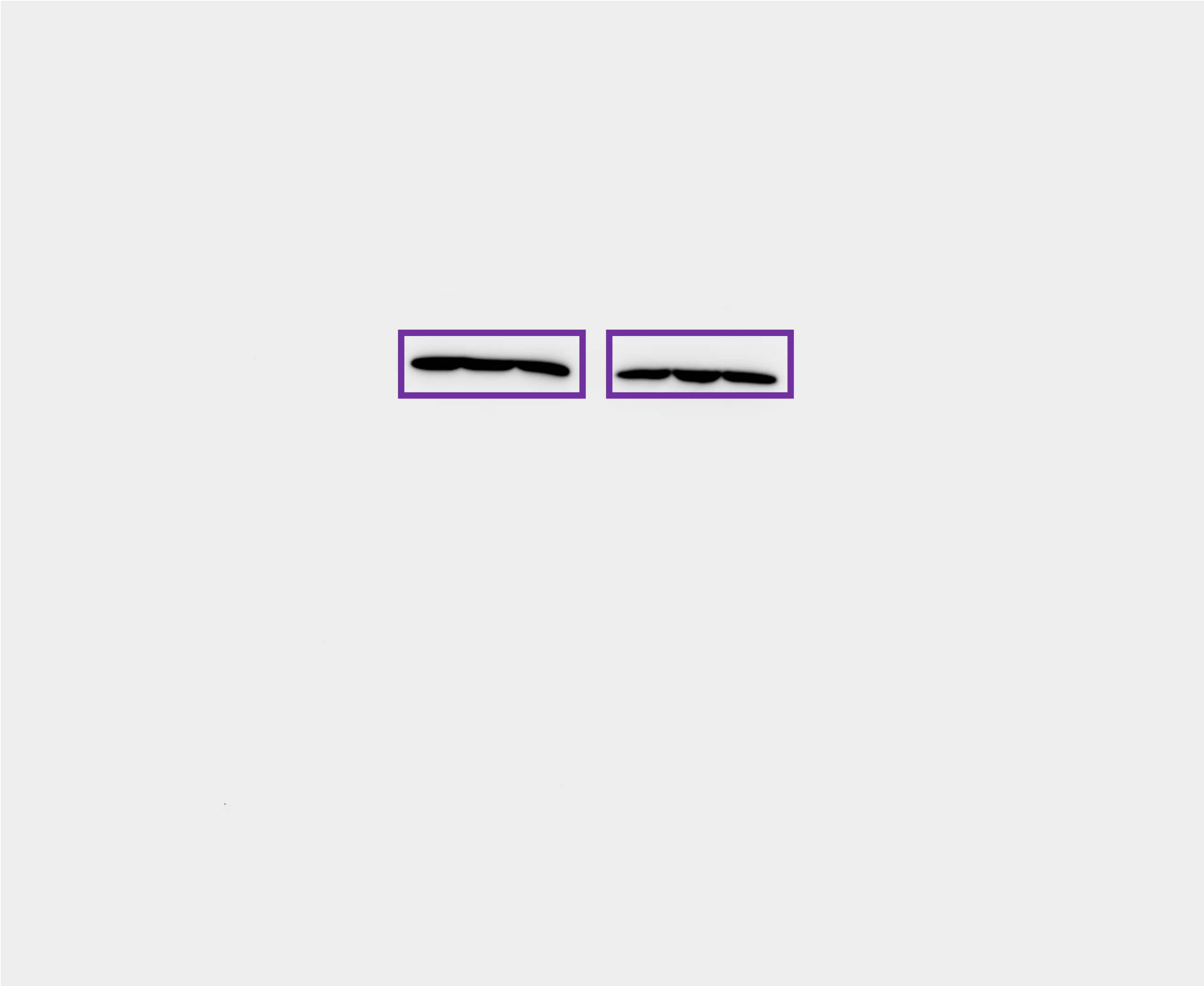

Figure 3G
MHCC97-H
Negative siRNA G6PD siRNA
FLNA

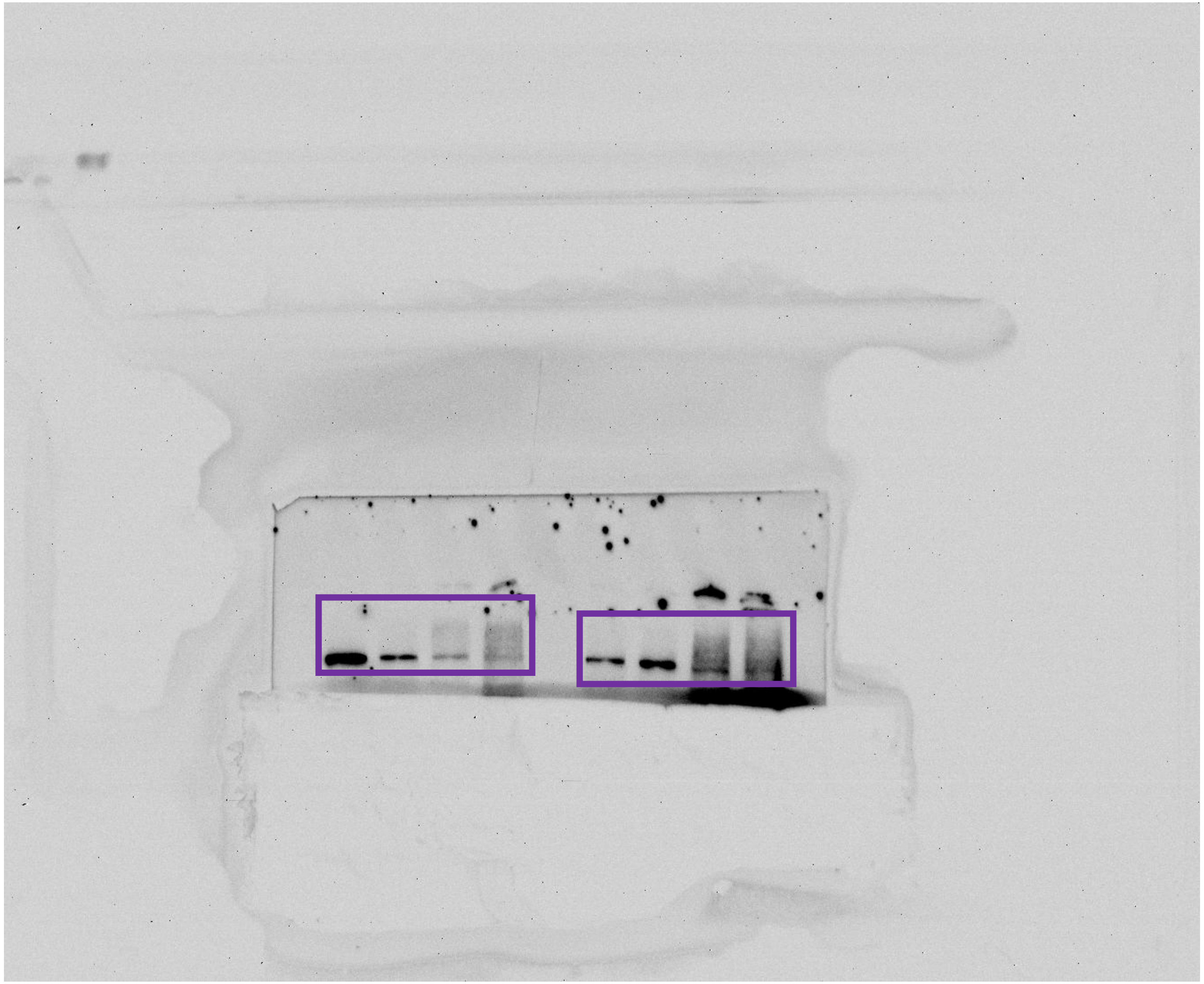

Figure 3G
MHCC97-H Negative siRNA
DREBRIN

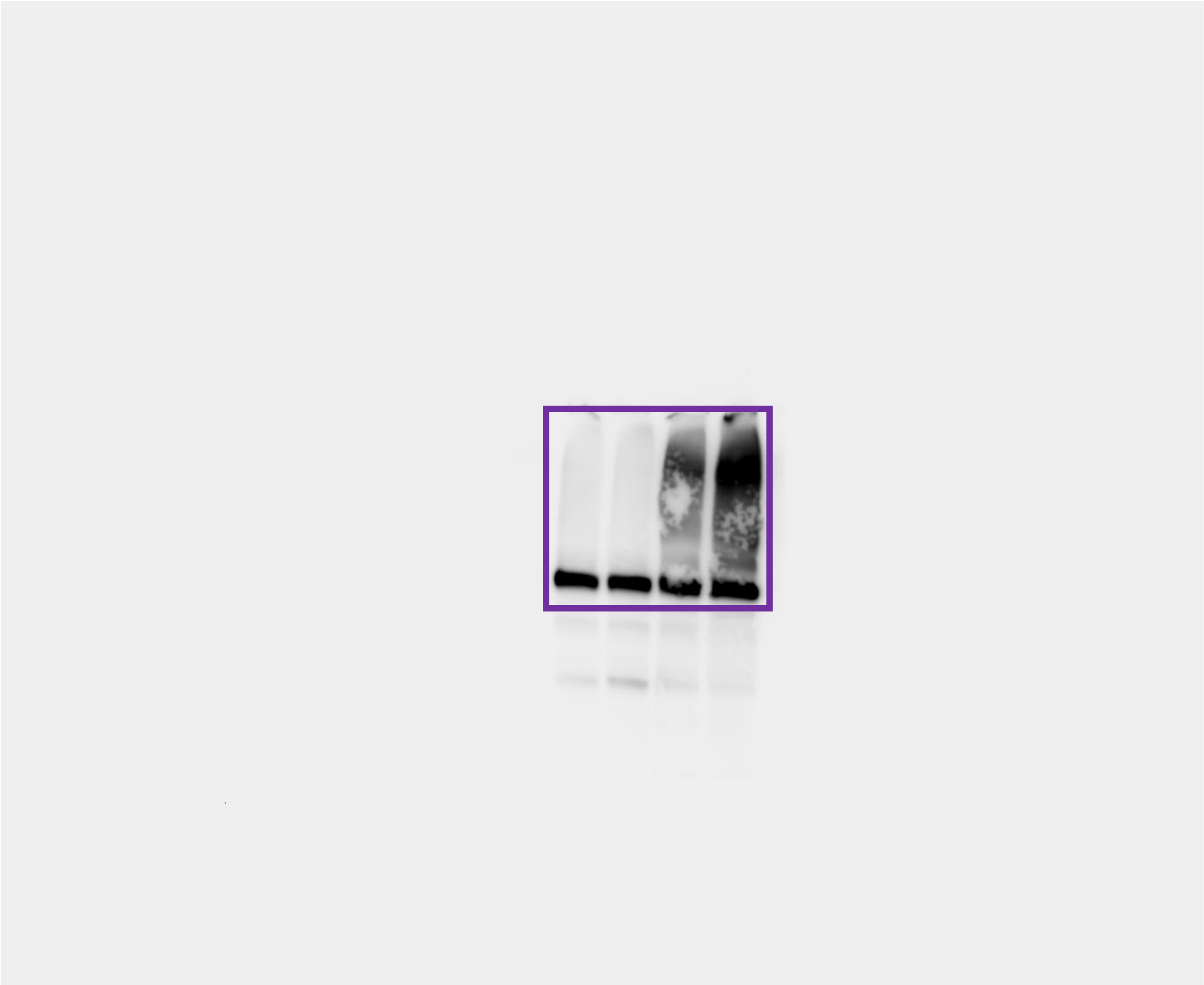

Figure 3G
MHCC97-H G6PD siRNA
DREBRIN

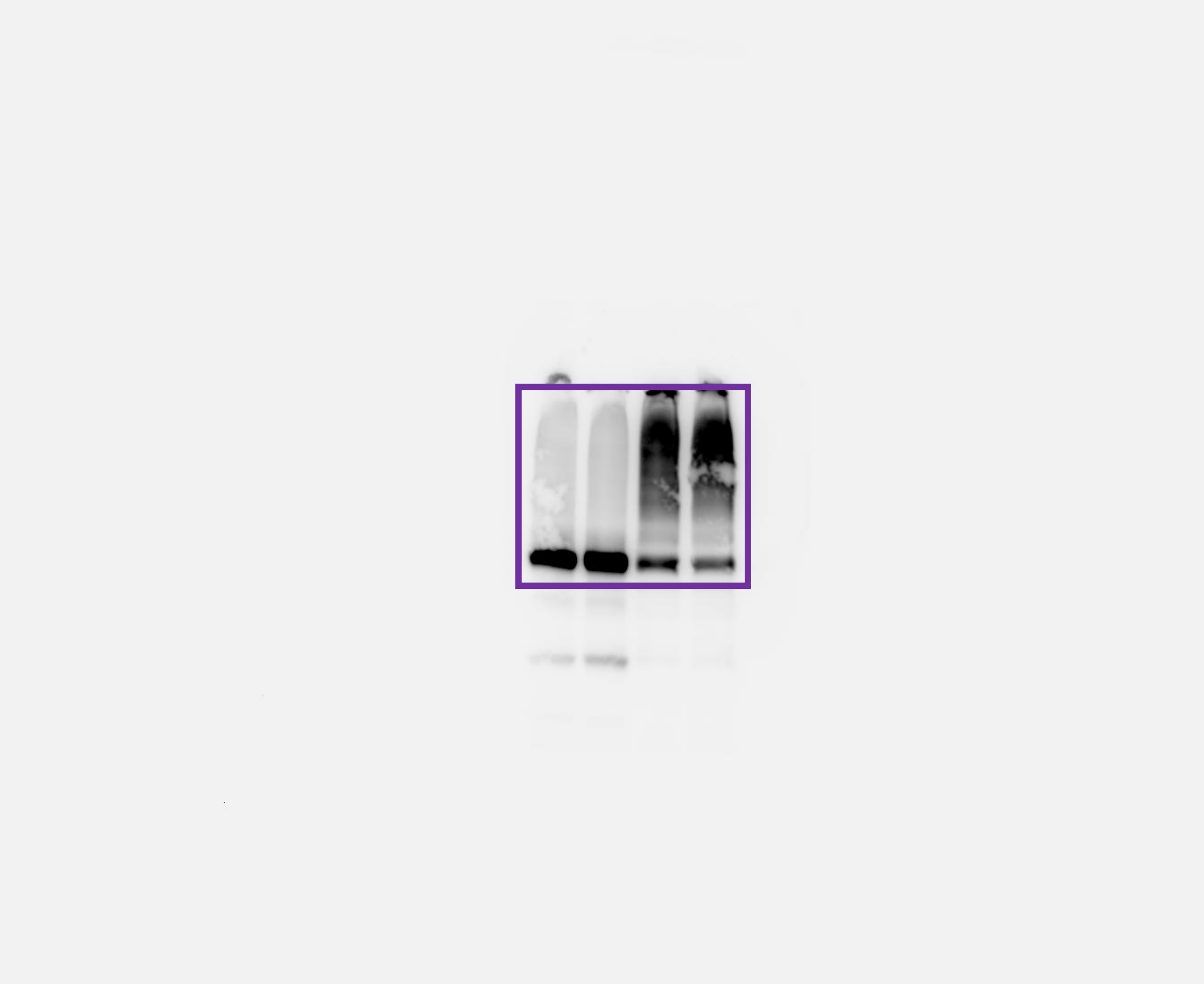

Figure 3G
MHCC97-H
Negative siRNA G6PD siRNA
GAPDH

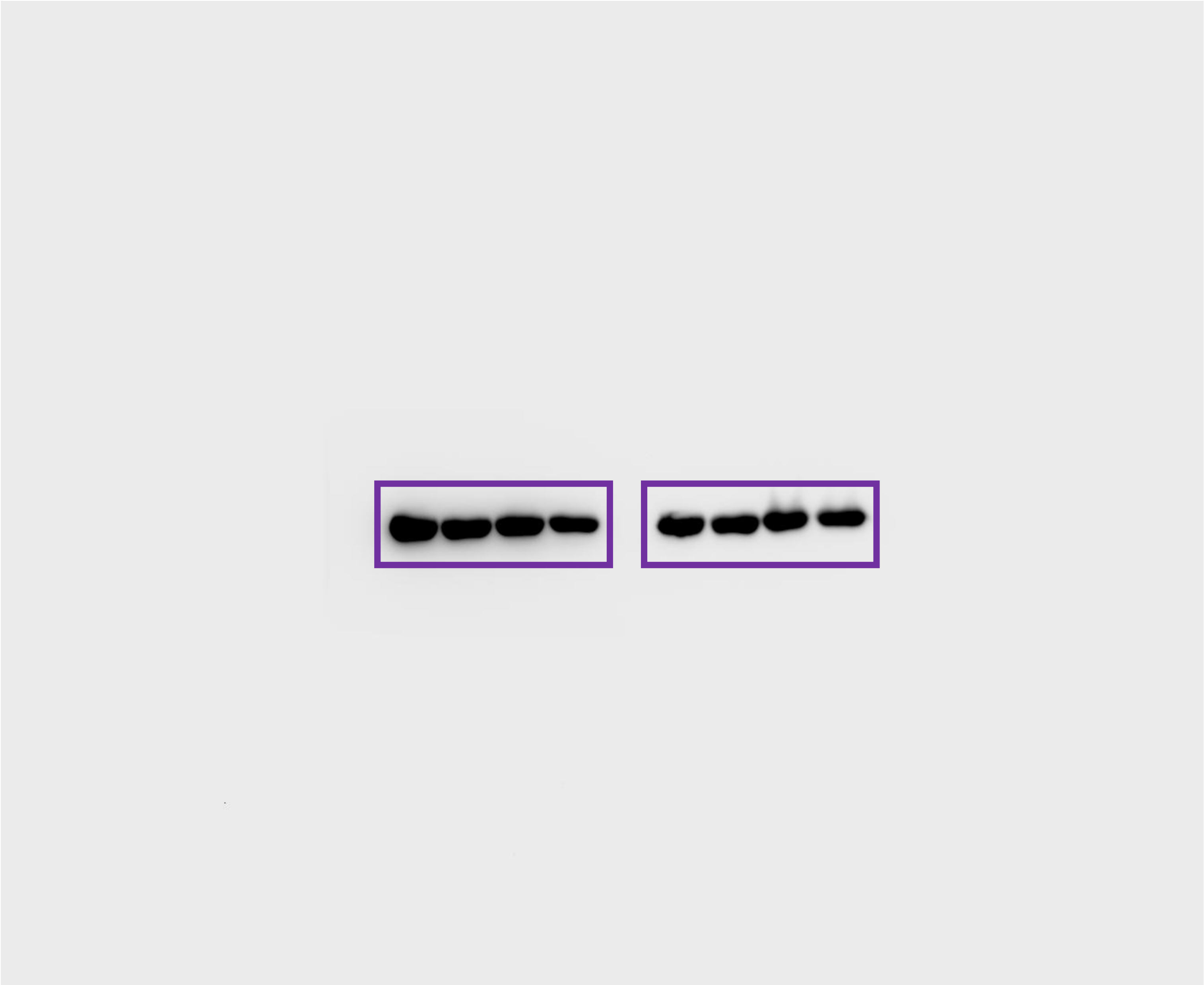

Figure 3H
Hep 3B Negative siRNA
FLNA

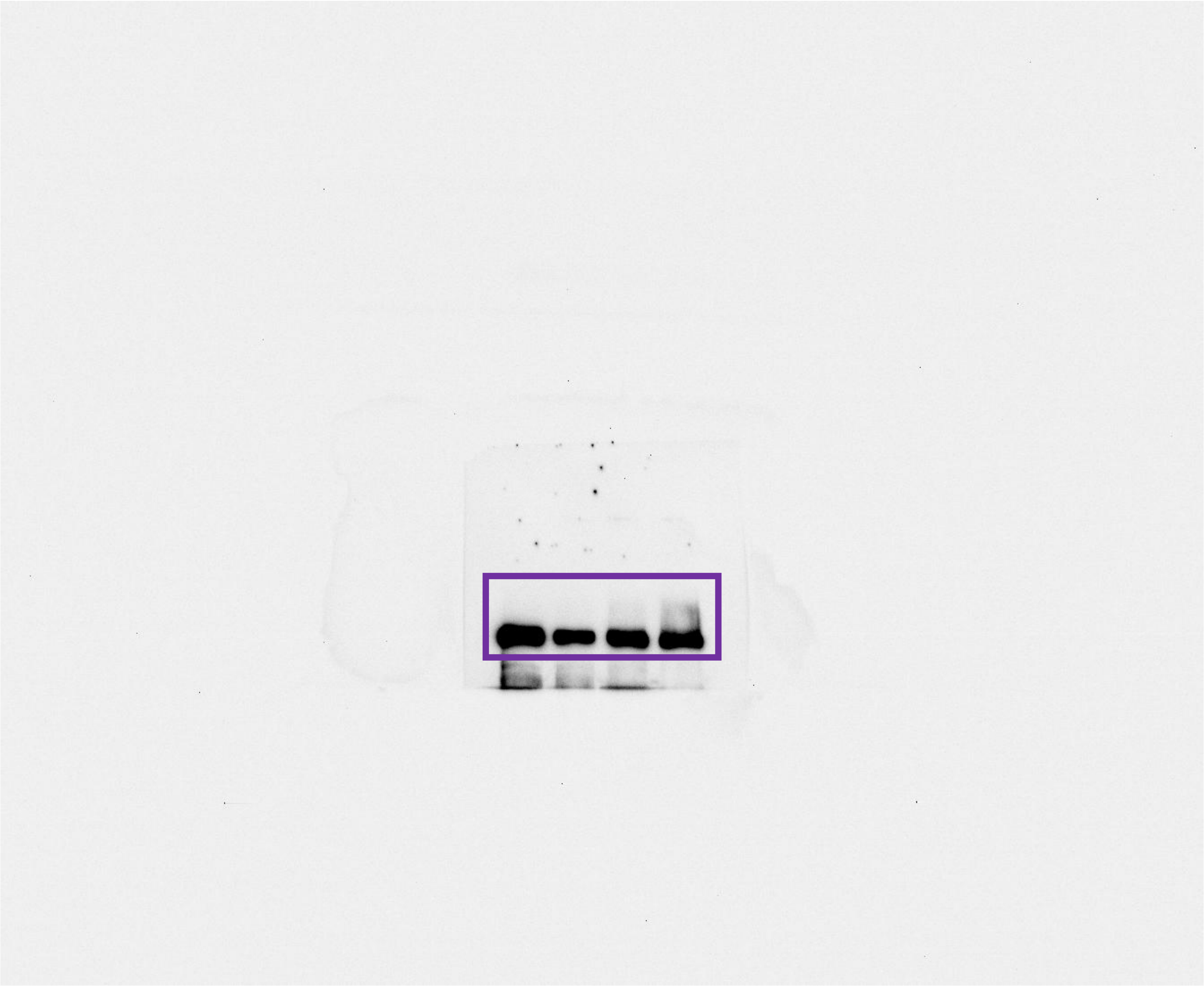

Figure 3H
Hep 3B G6PD siRNA
FLNA

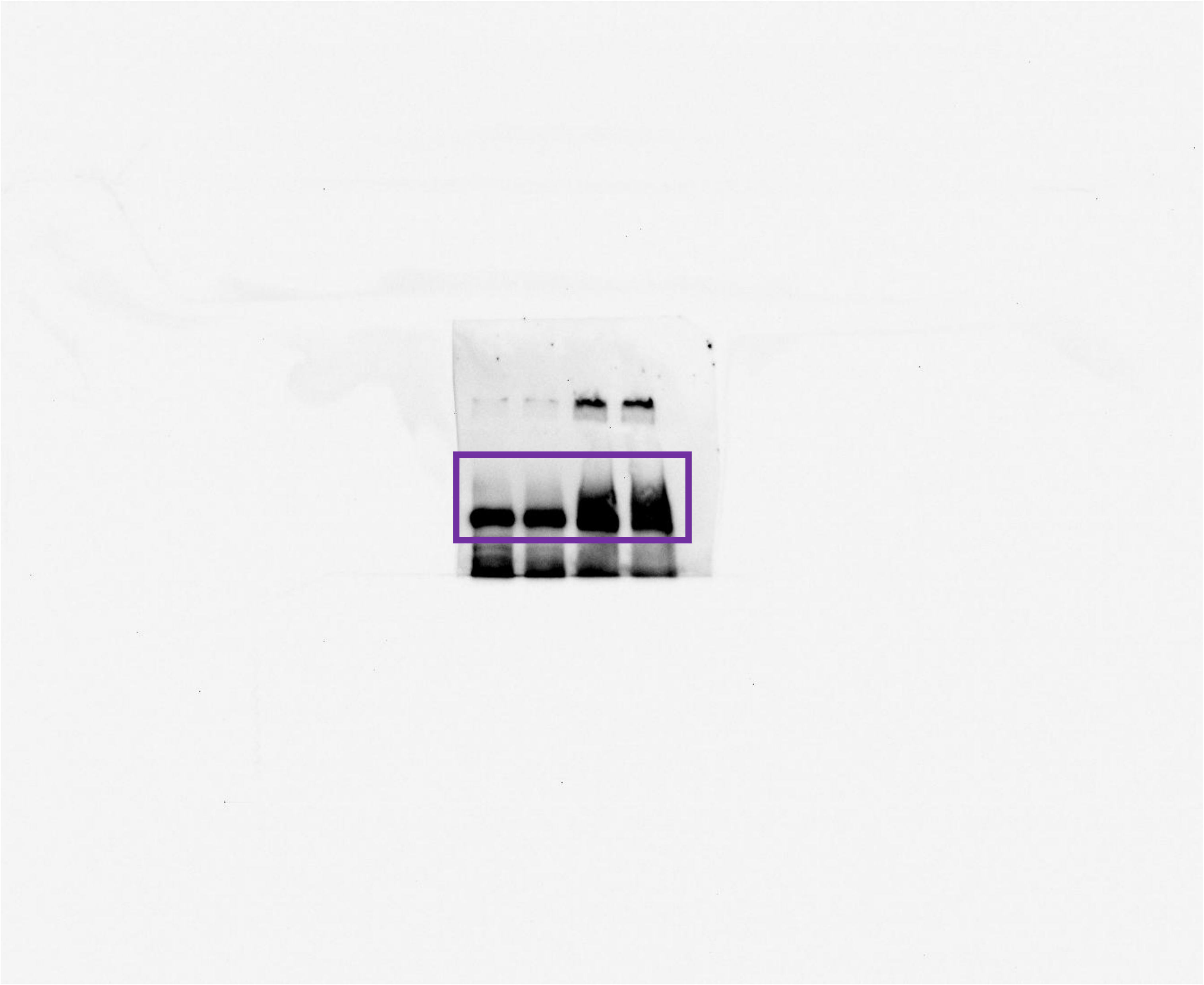

Figure 3H
Hep 3B Negative siRNA
DREBRIN

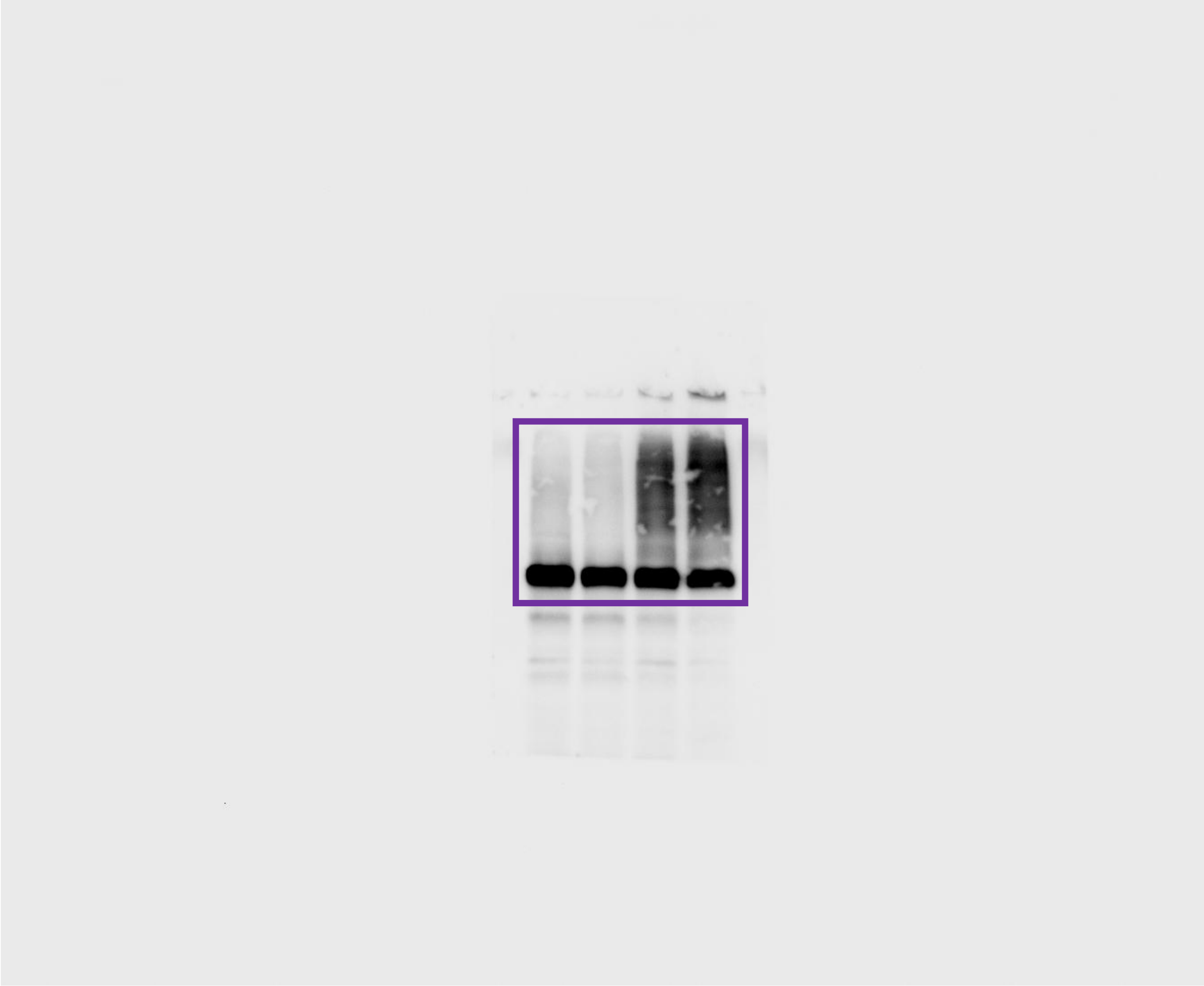

Figure 3H
Hep 3B G6PD siRNA
DREBRIN

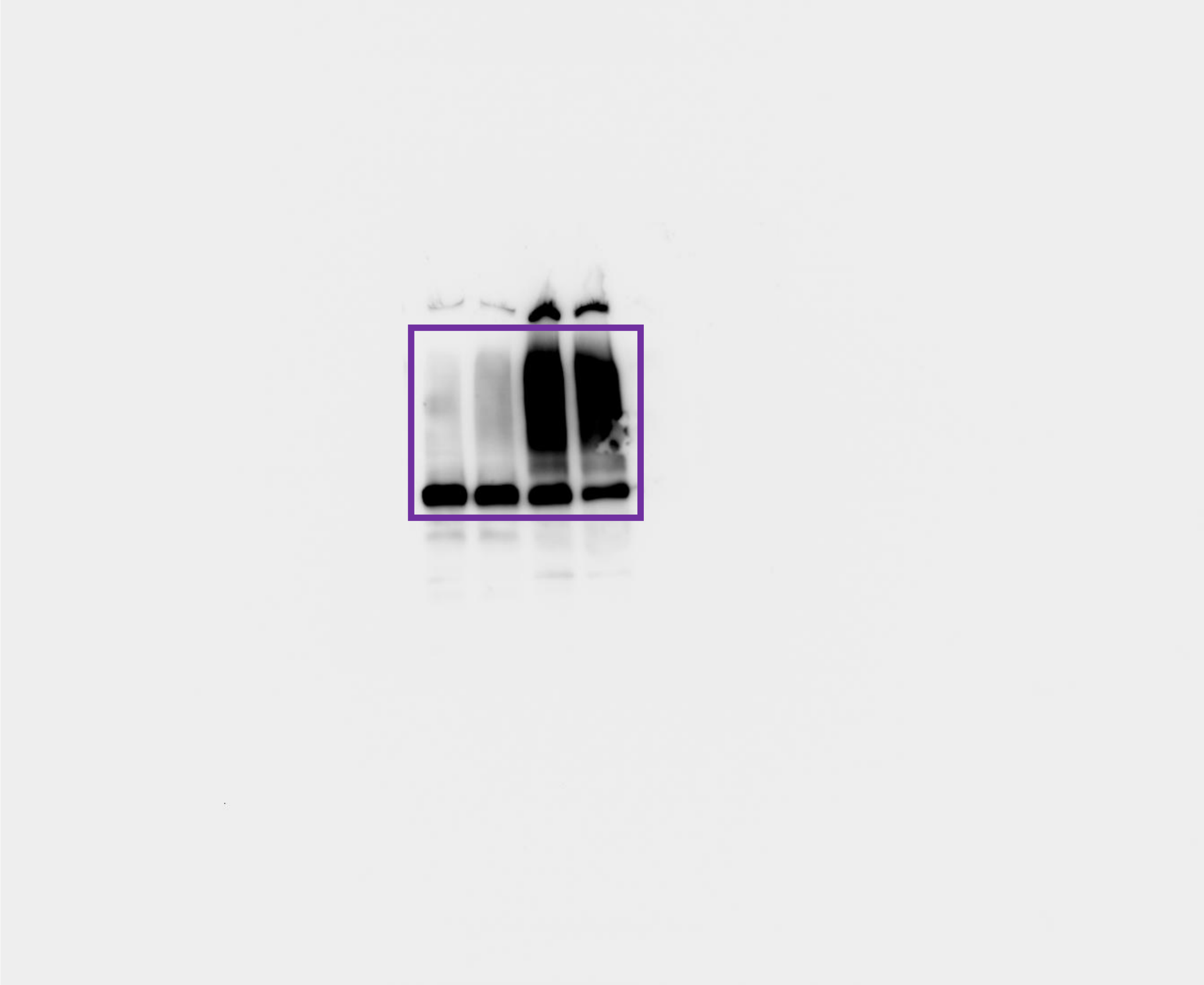

Figure 3G
Hep 3B
Negative siRNA G6PD siRNA
GAPDH

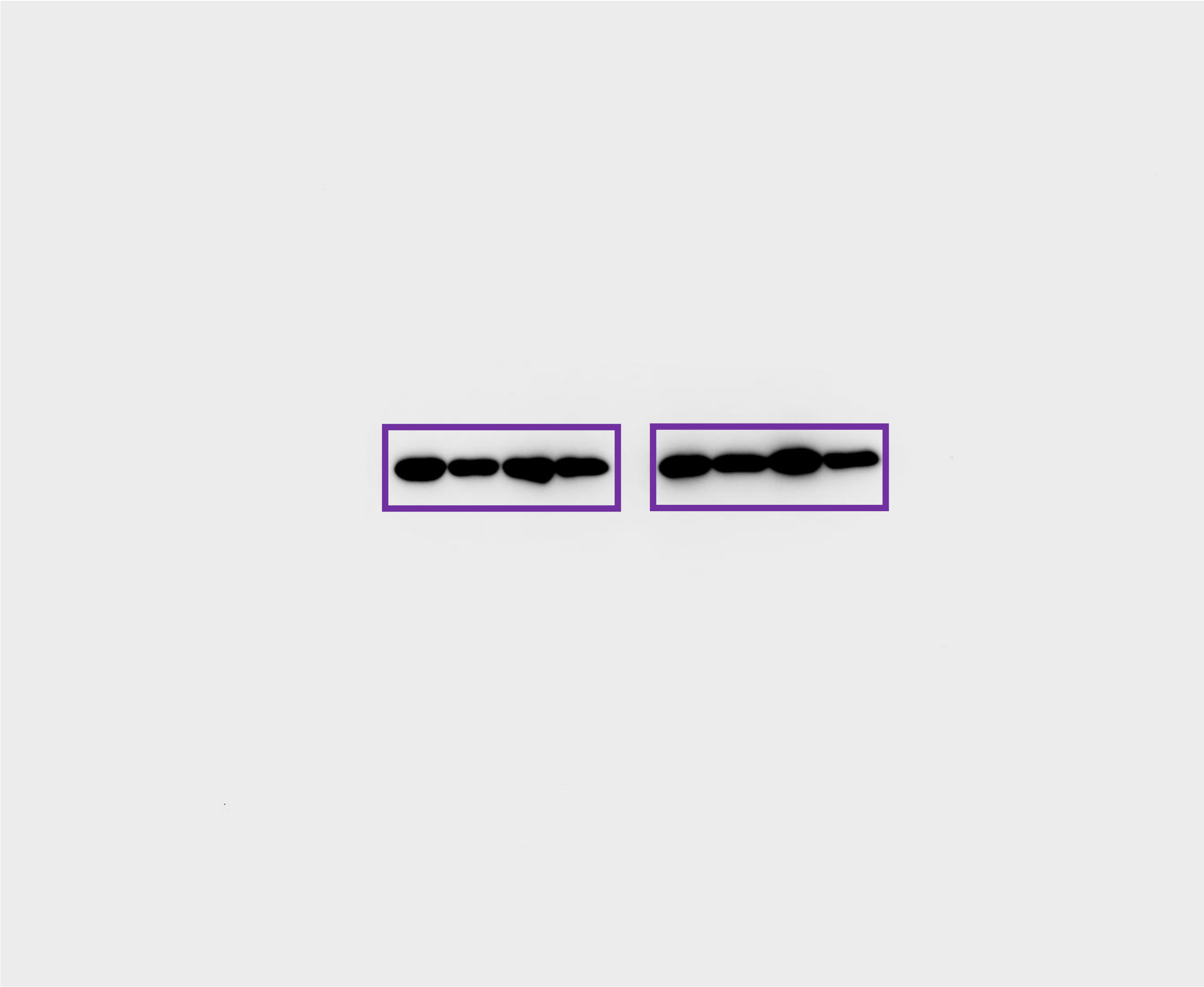

Figure 4V
Ctrl FLNA

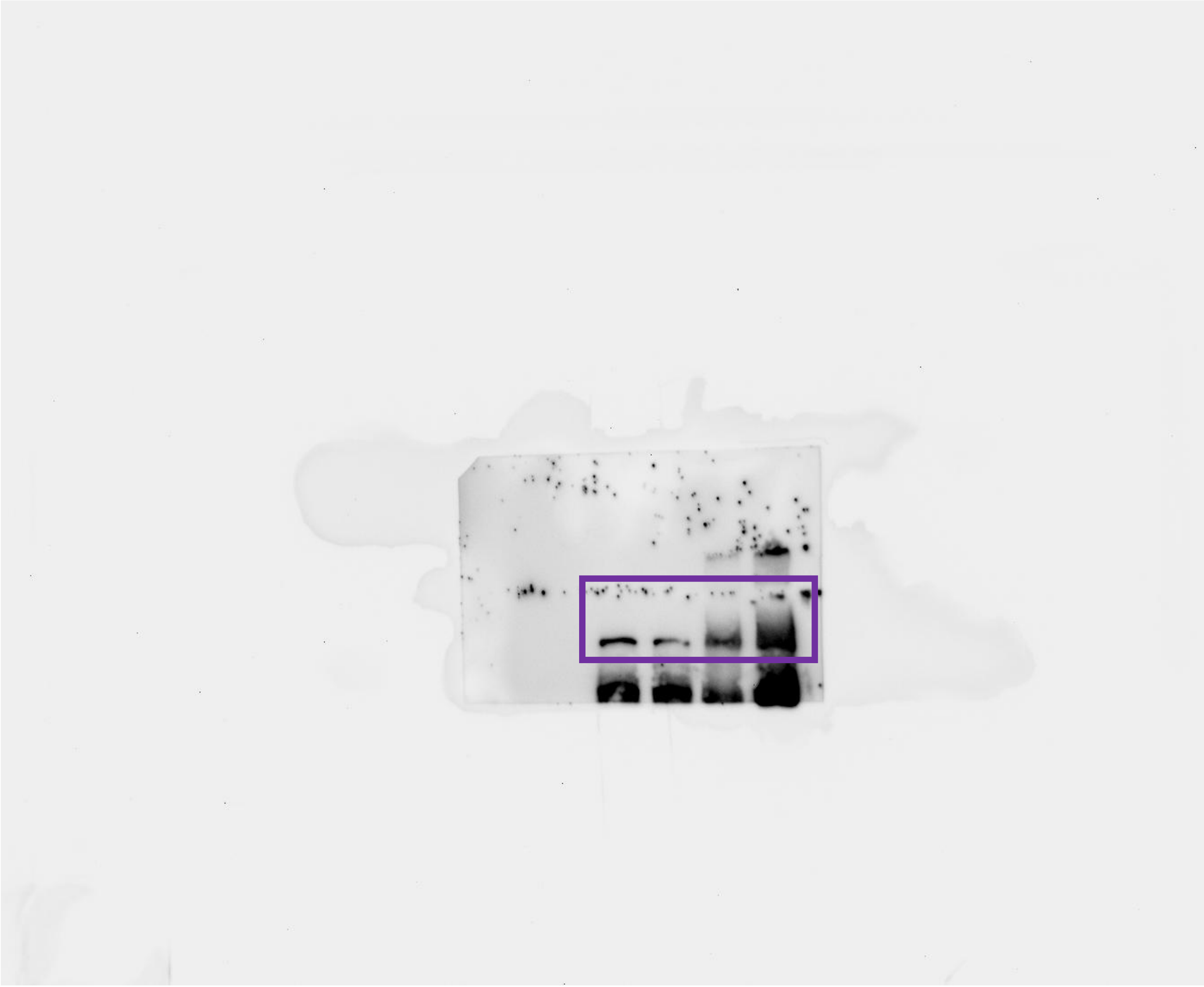

Figure 4V
GSH FLNA

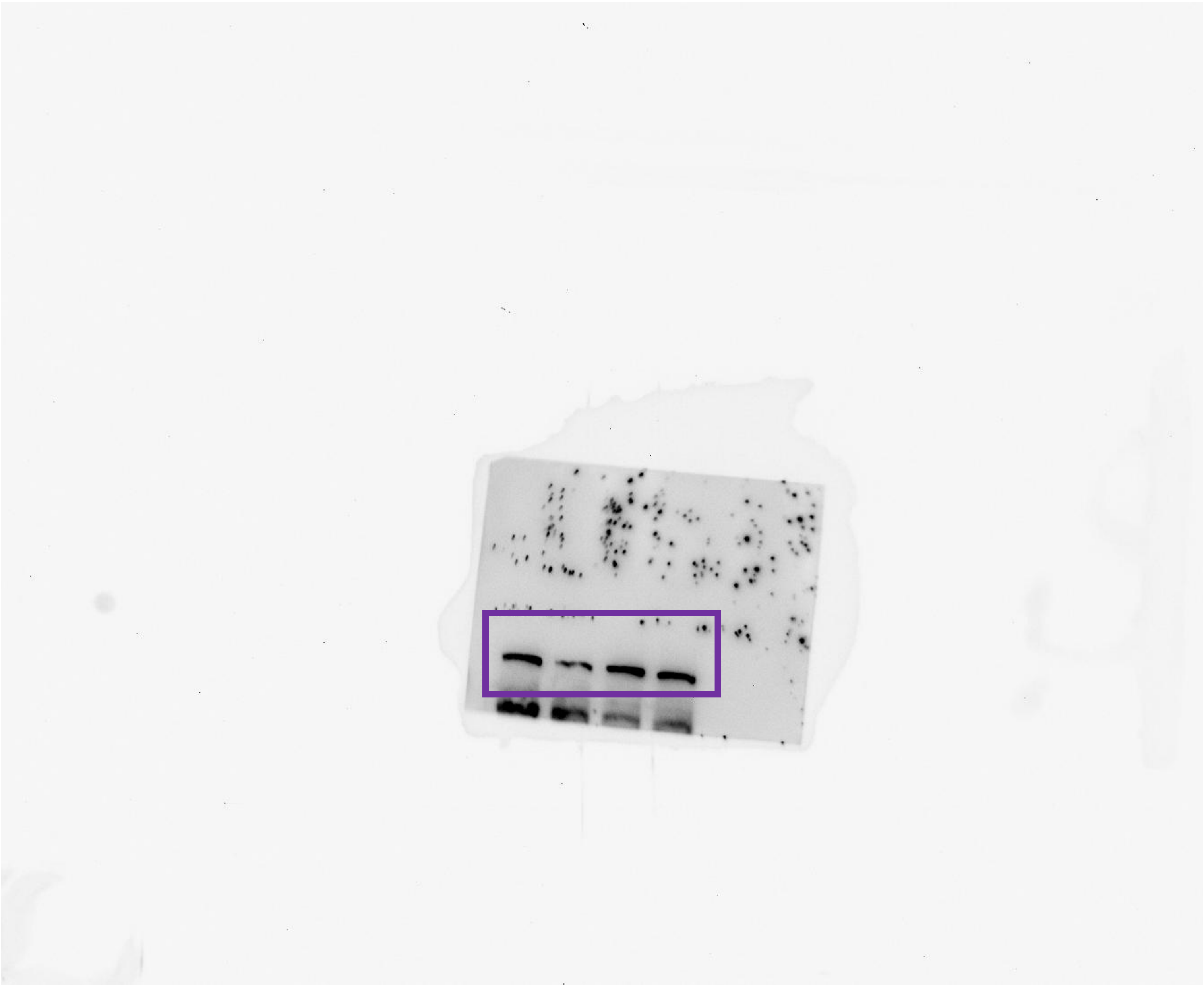

Figure 4V
Ctrl DREBRIN

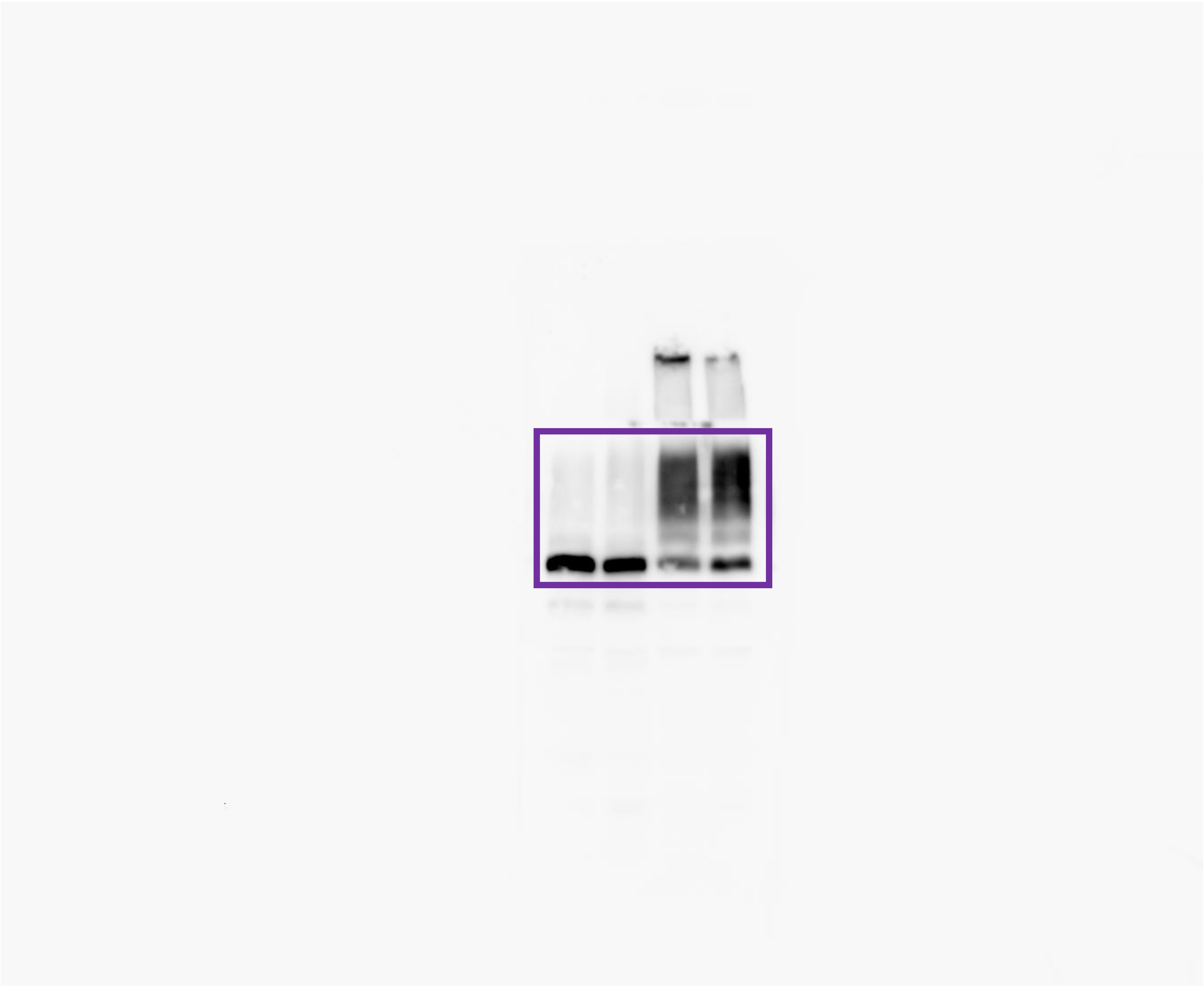

Figure 4V
GSH DREBRIN

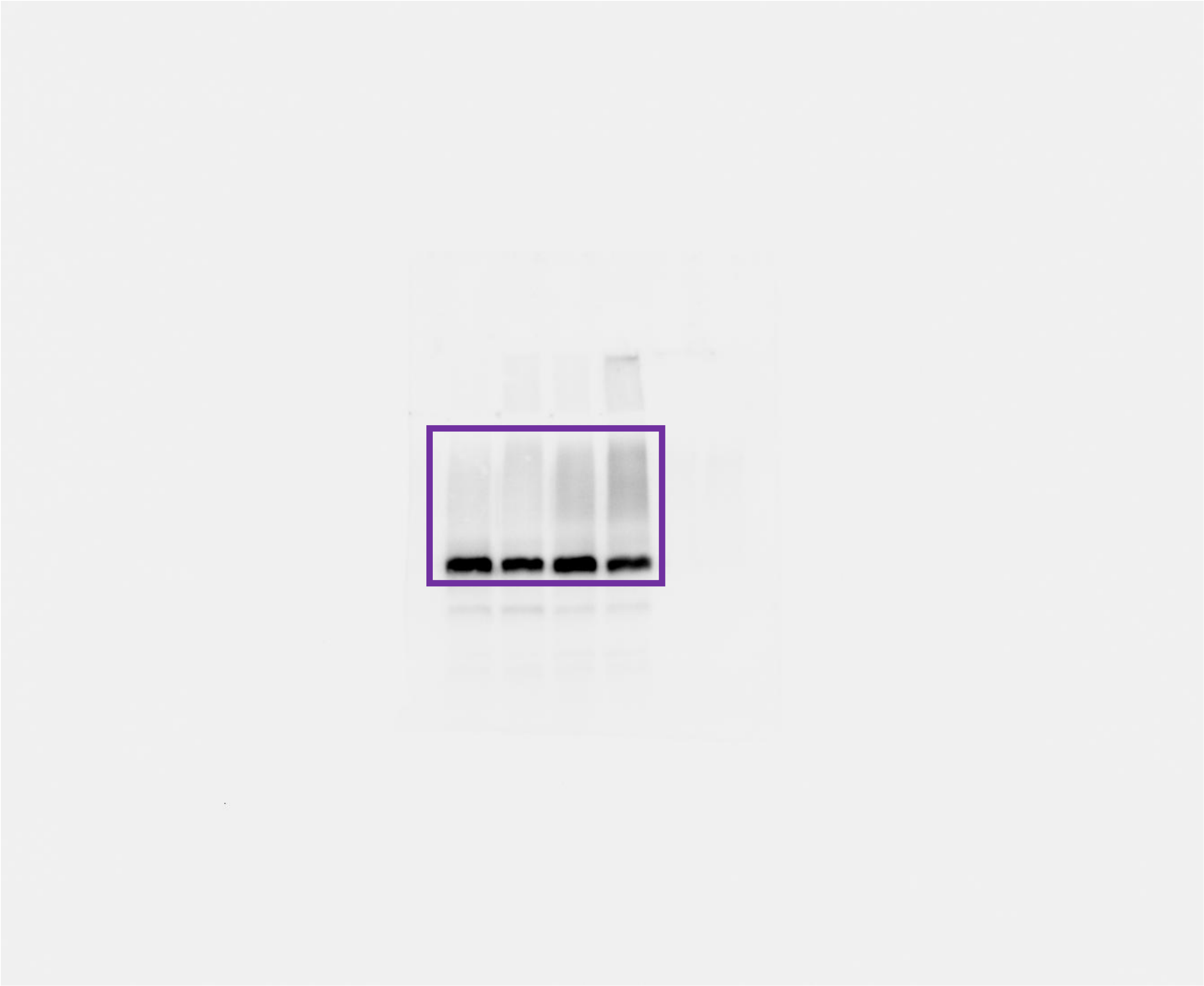

Figure 4V
Ctrl GSH GAPDH

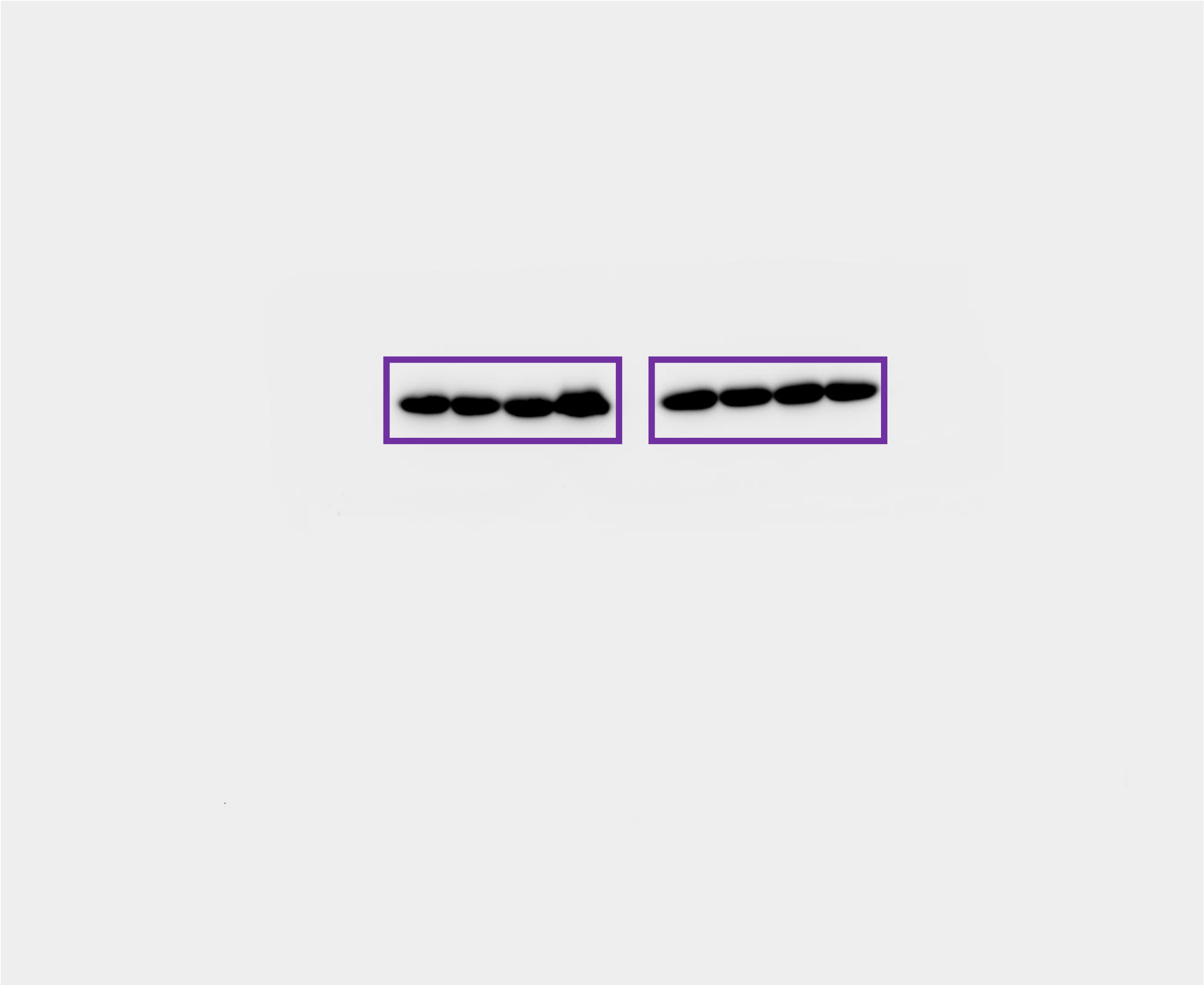

Figure 5C
MHCC97-H CQ PINK

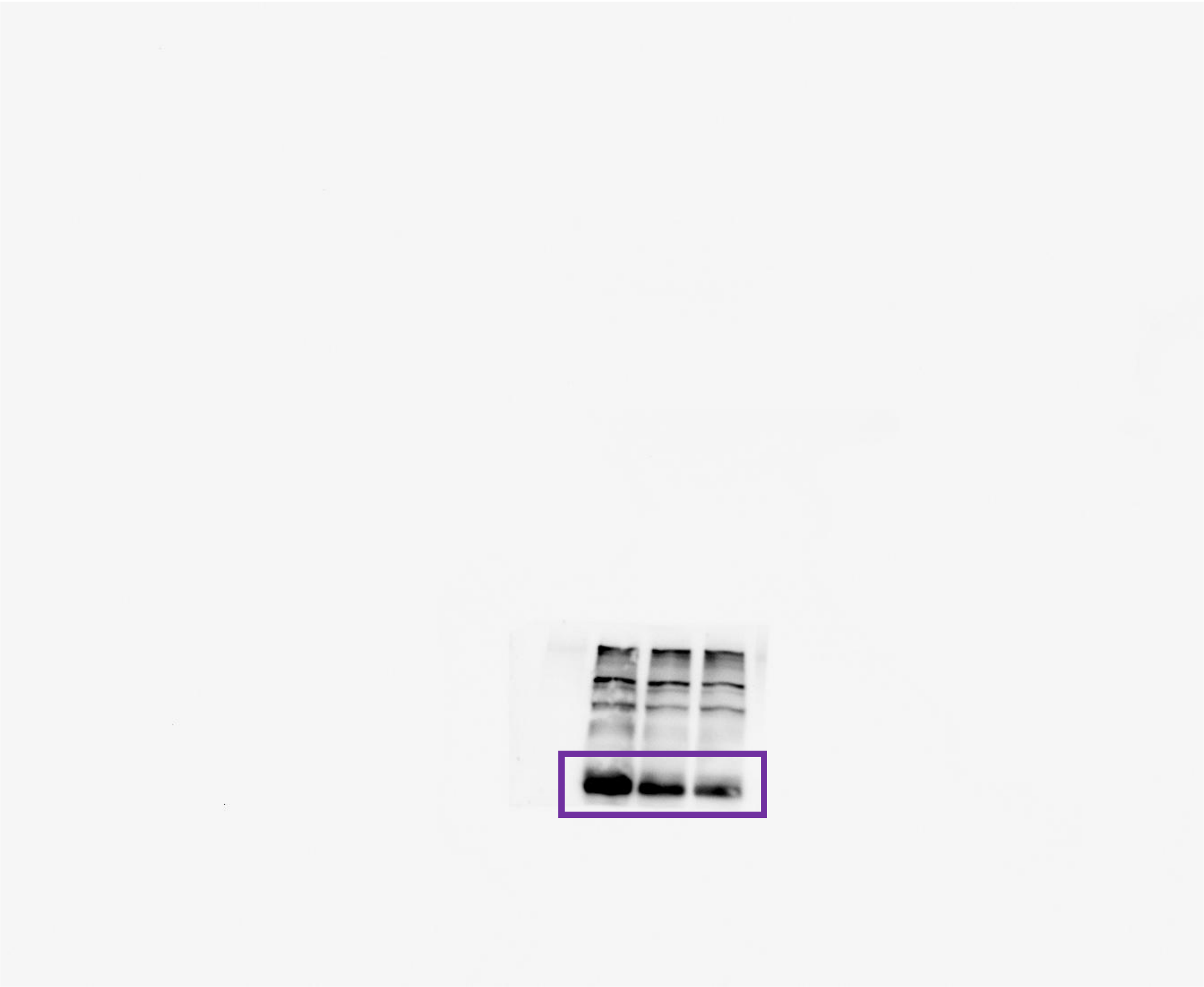

Figure 5C
MHCC97-H CQ PARK2

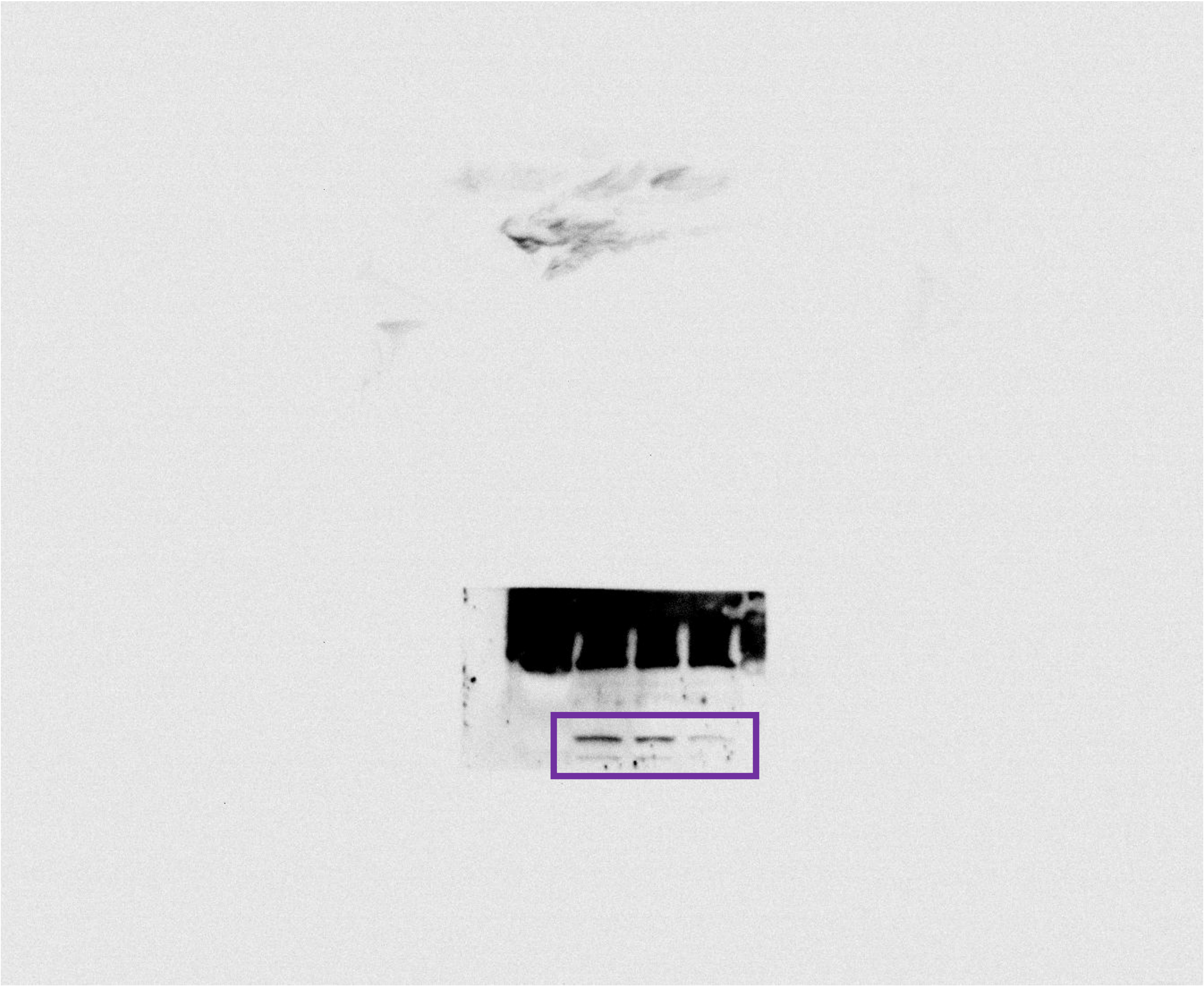

Figure 5C
MHCC97-H CQ GAPDH

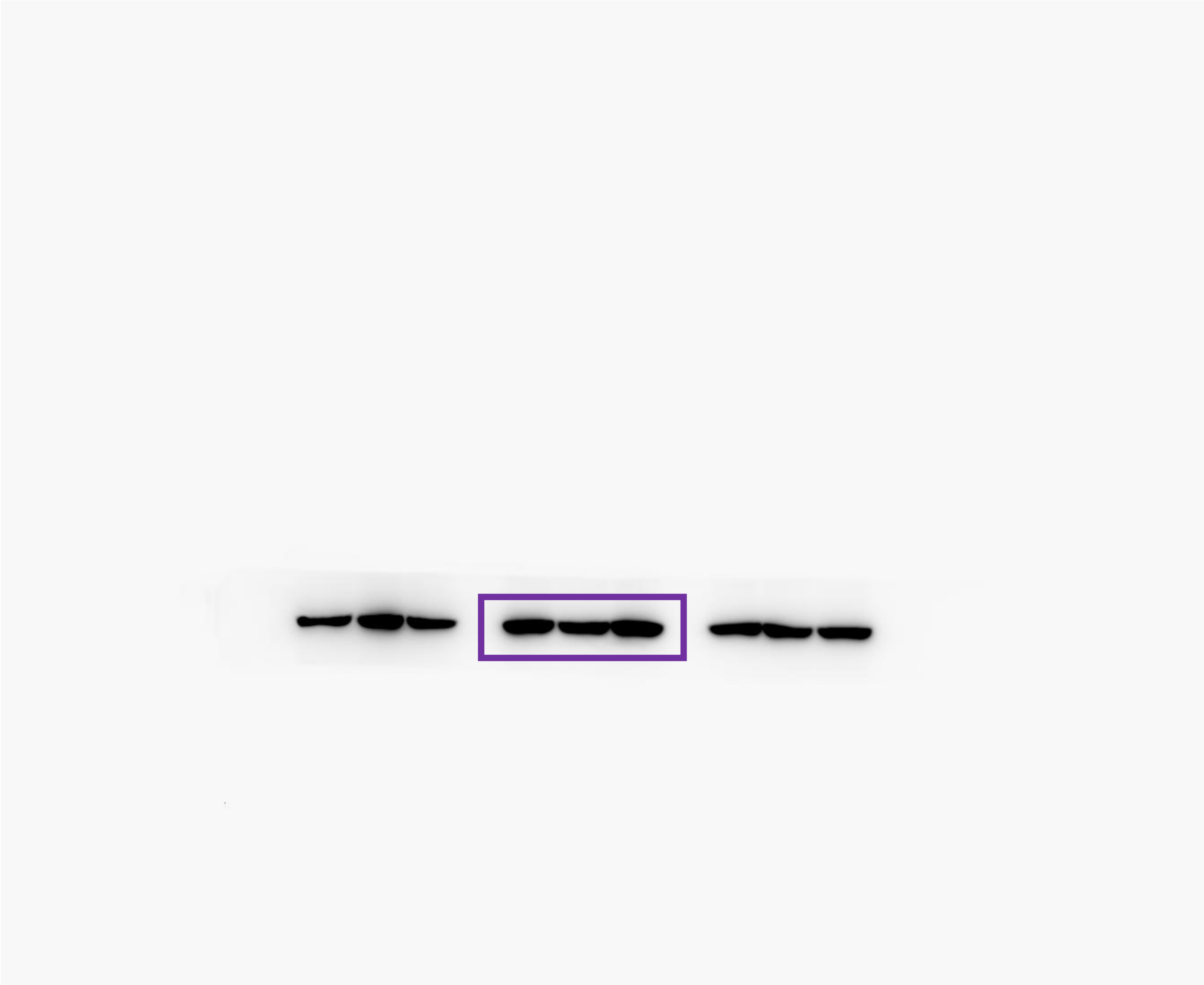

Figure 5C
MHCC97-H APN PINK

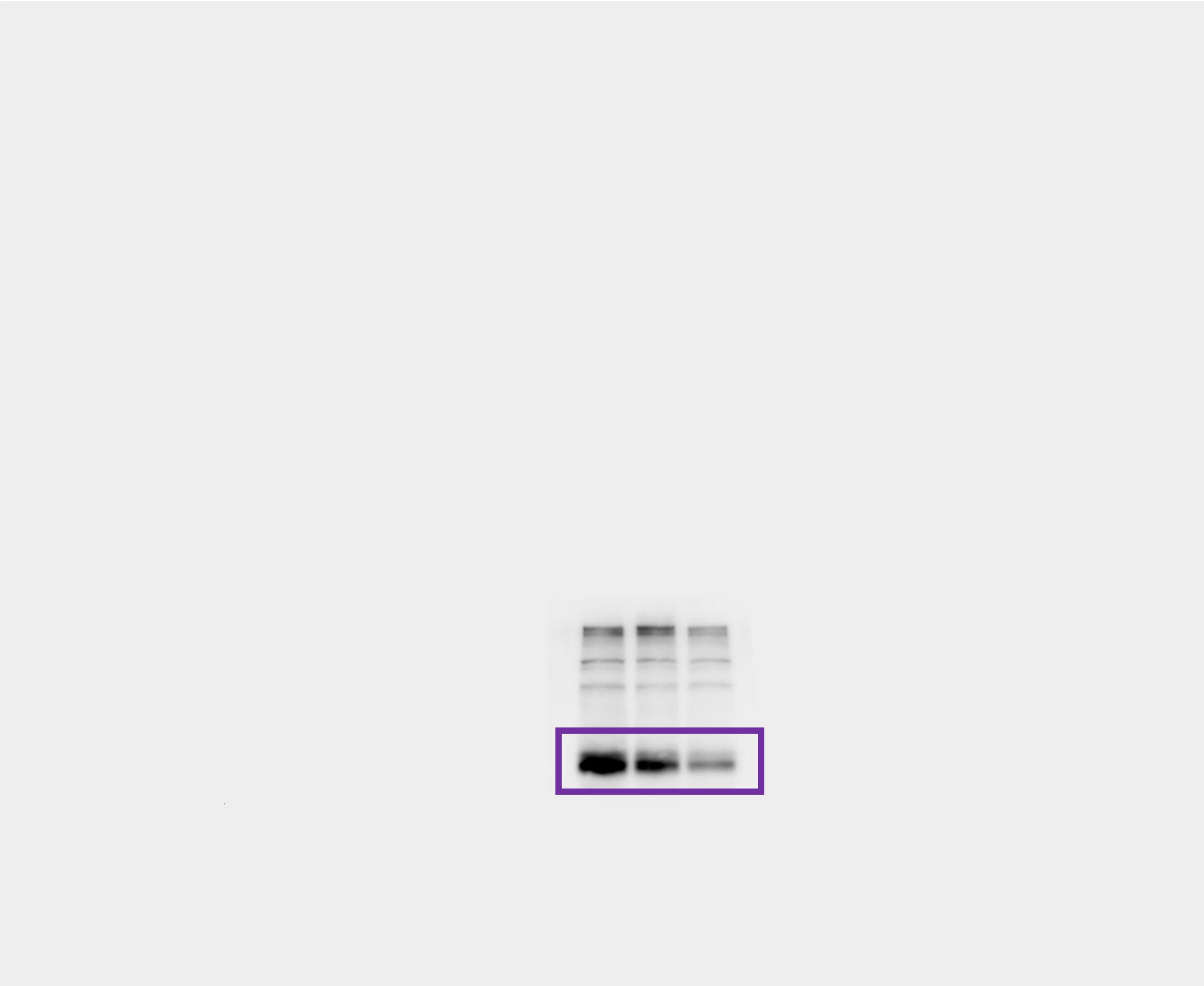

Figure 5C
MHCC97-H APN PARK2

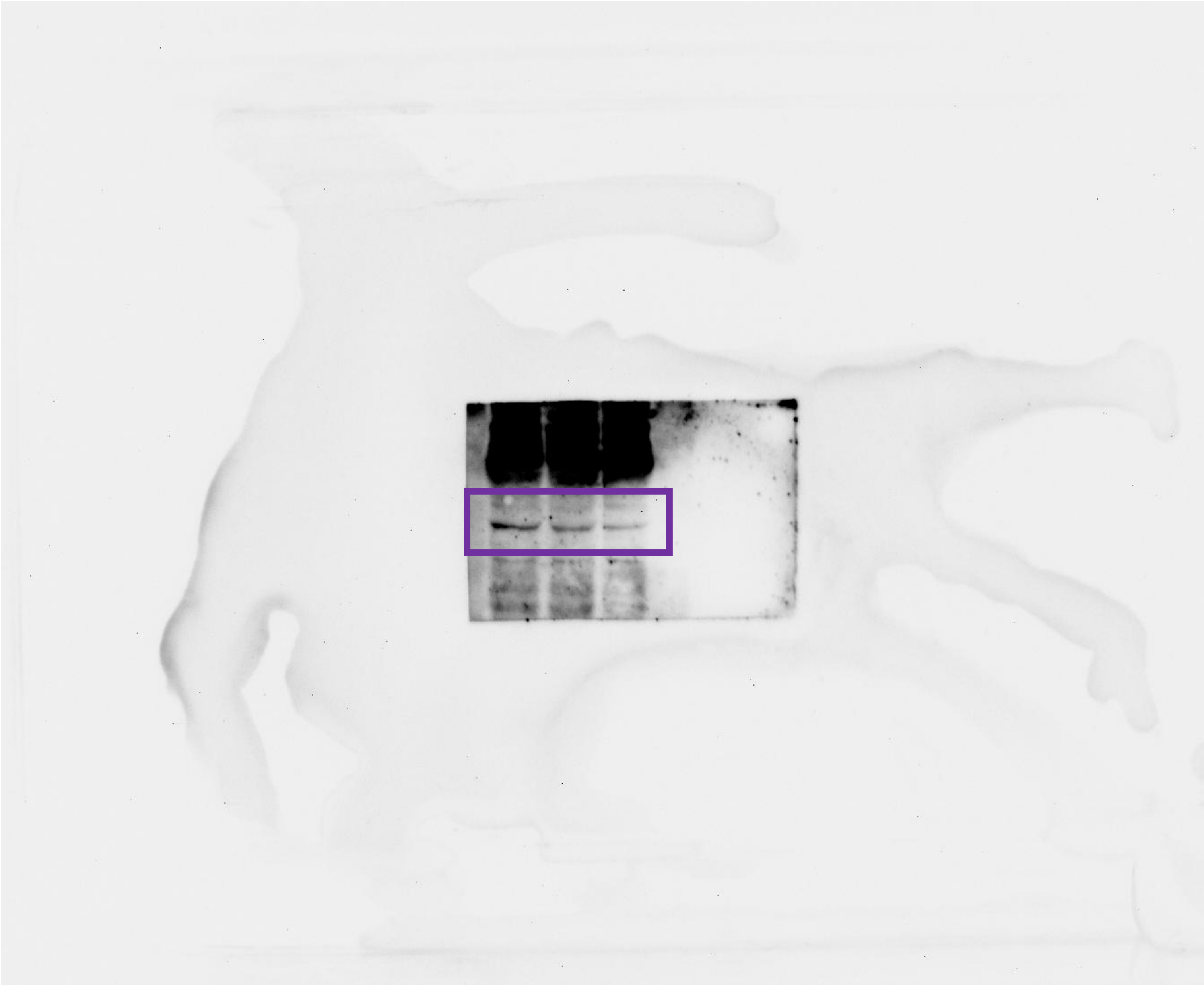

Figure 5C
MHCC97-H APN GAPDH

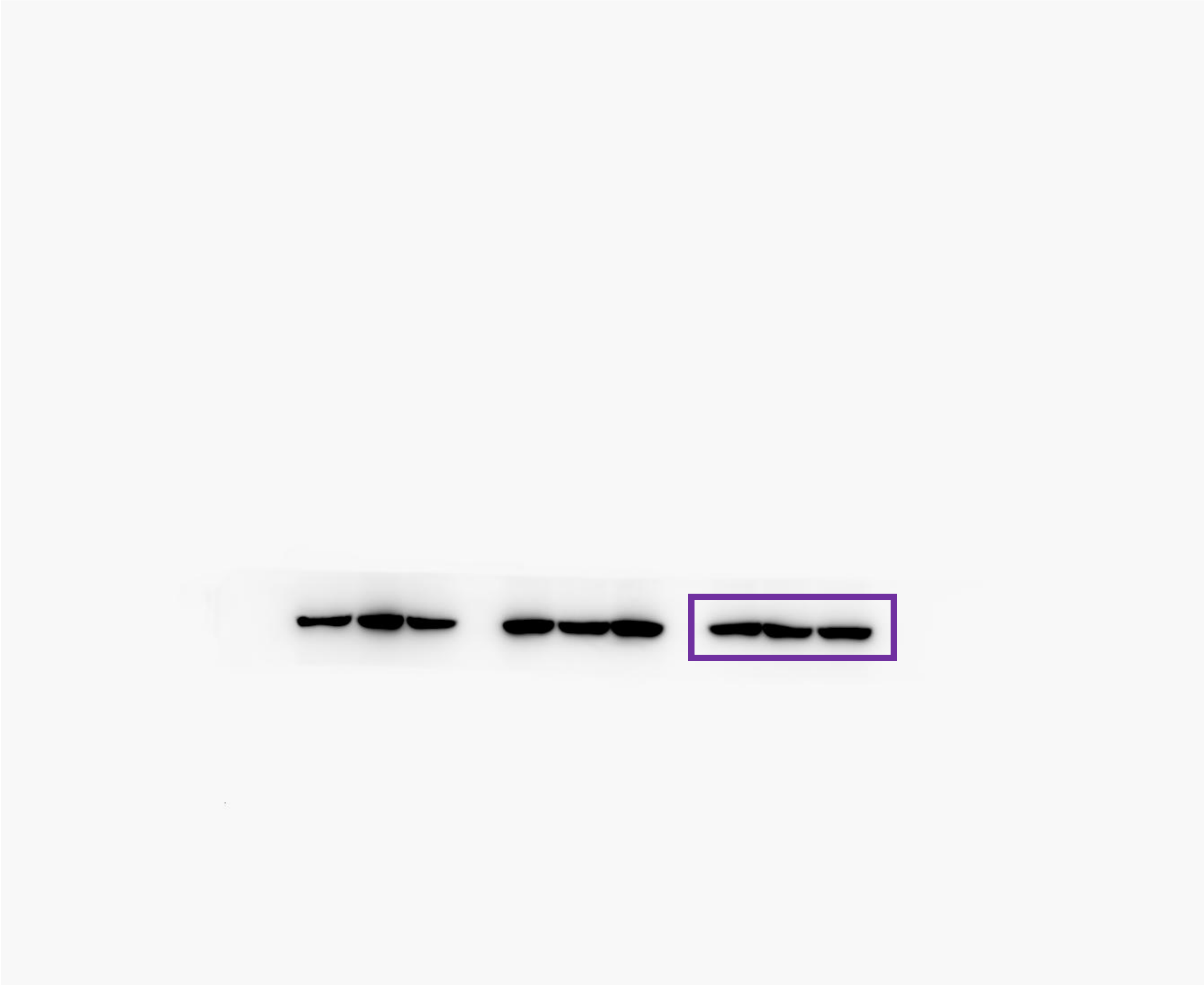

Figure 5D
Hep 3B CQ PINK

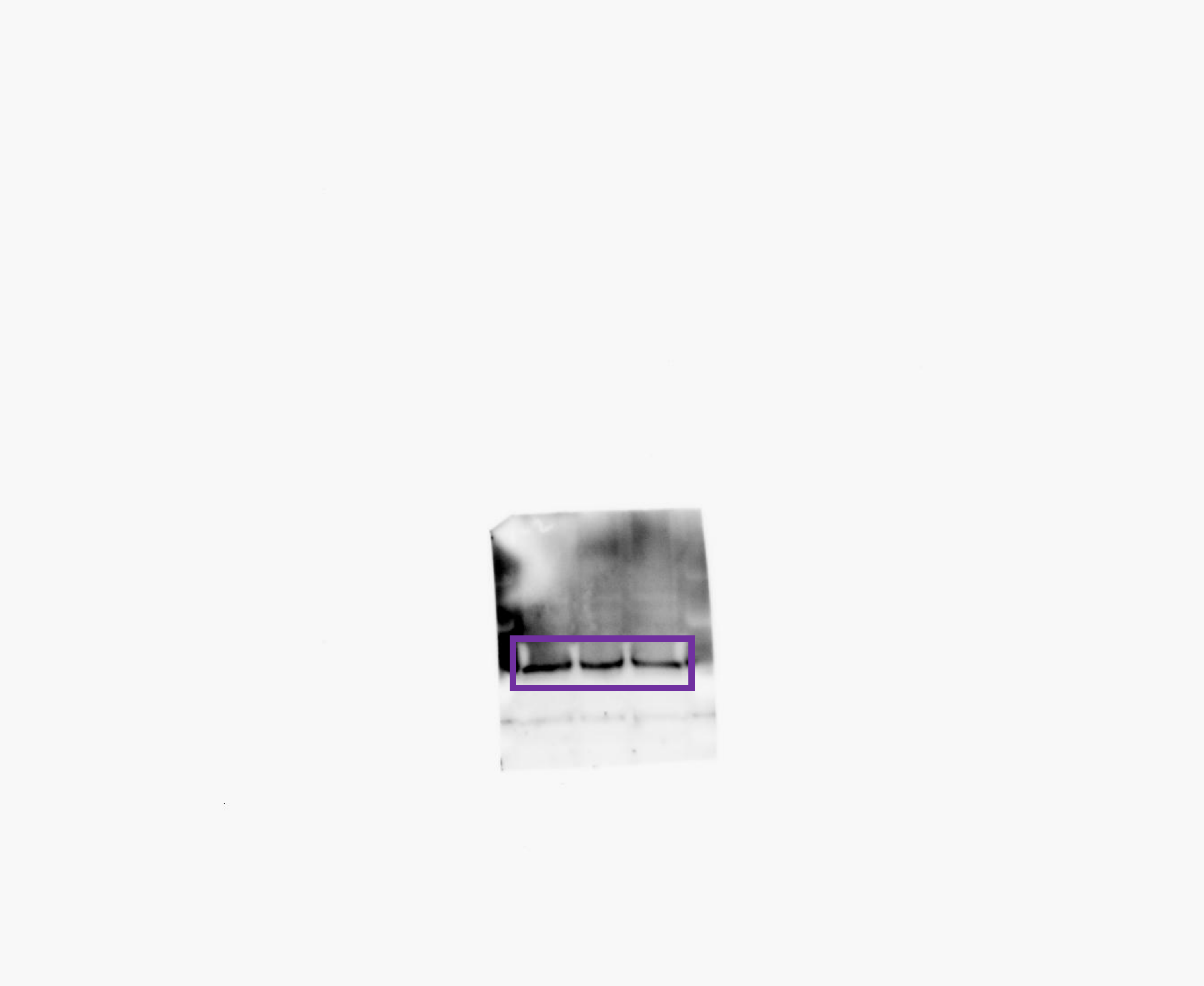

Figure 5D
Hep 3B CQ PARK2

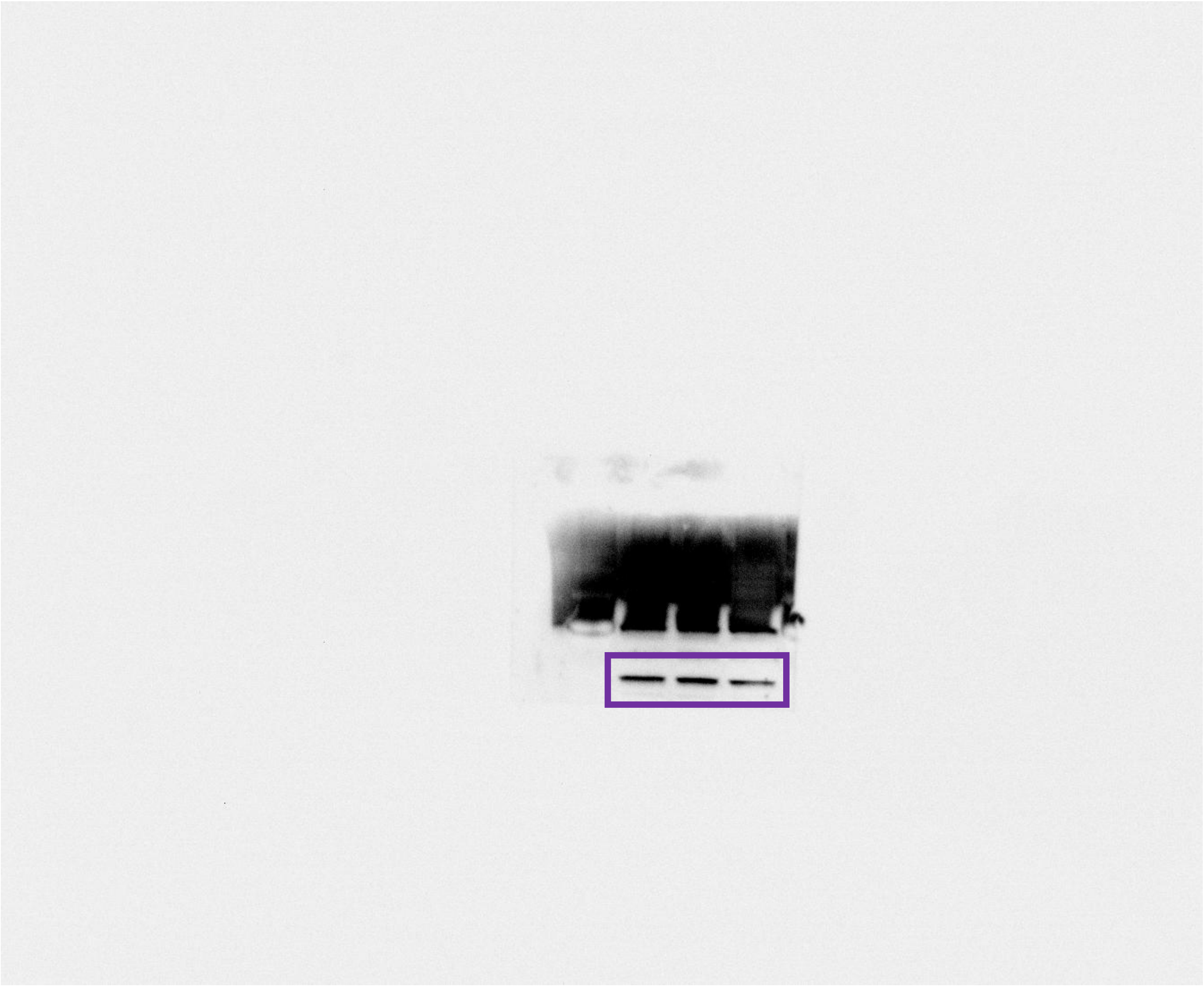

Figure 5D

Hep 3B CQ GAPDH

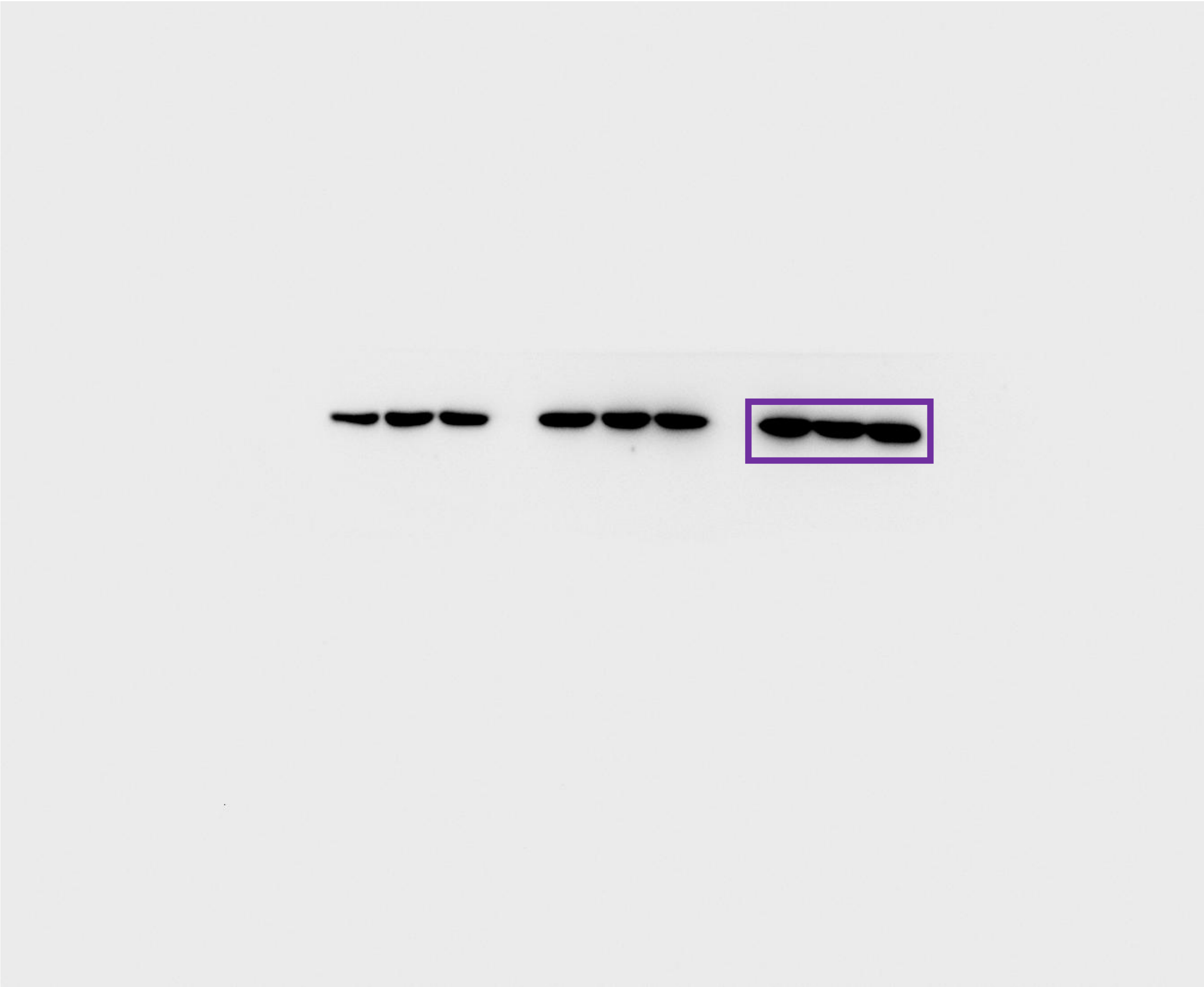

Figure 5D
Hep 3B APN PINK

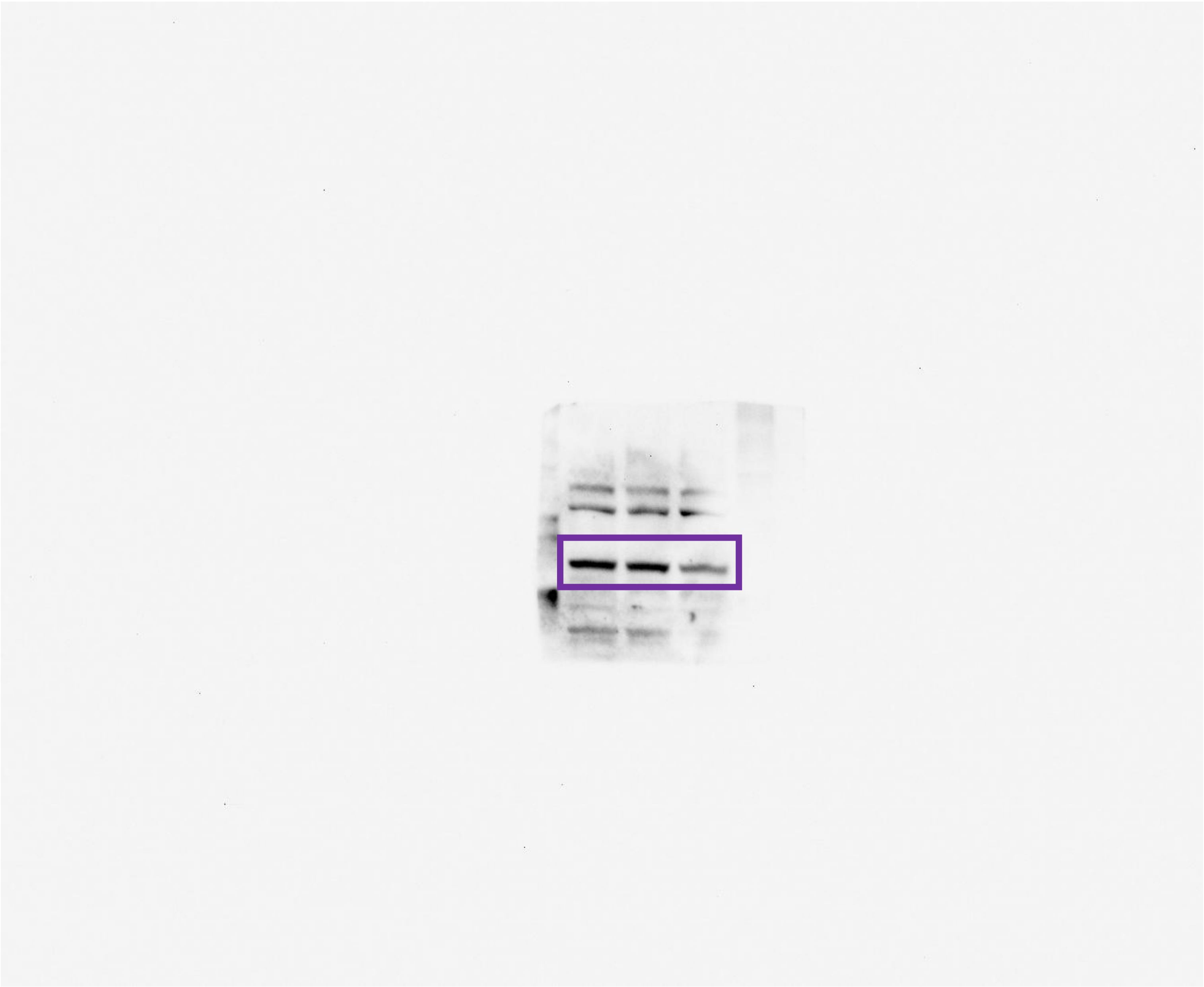

Figure 5D
Hep 3B APN PARK2

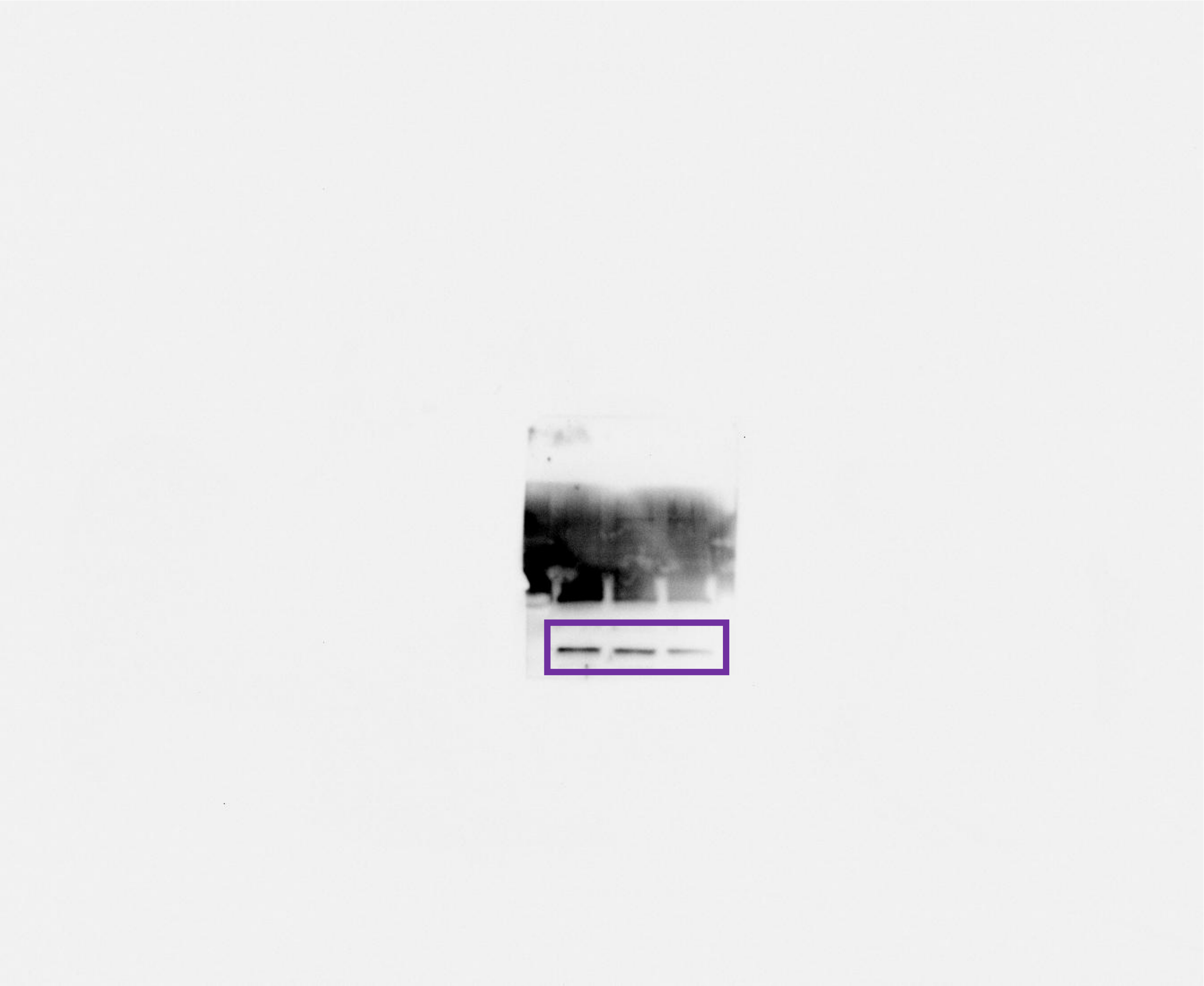

Figure 5D
Hep 3B APN GAPDH

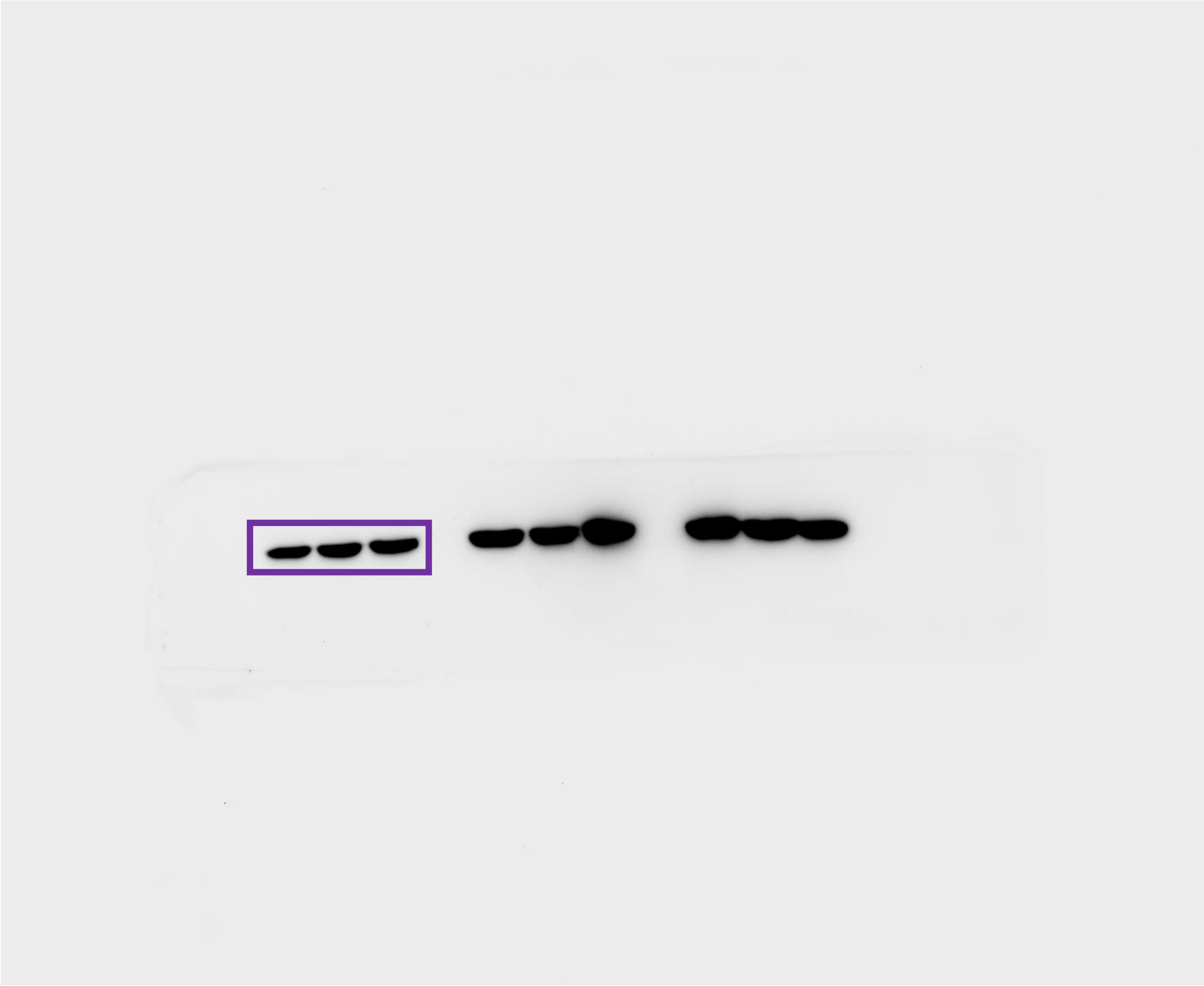

Figure 5I
MHCC97-H Ctrl Elamipretide
FLNA

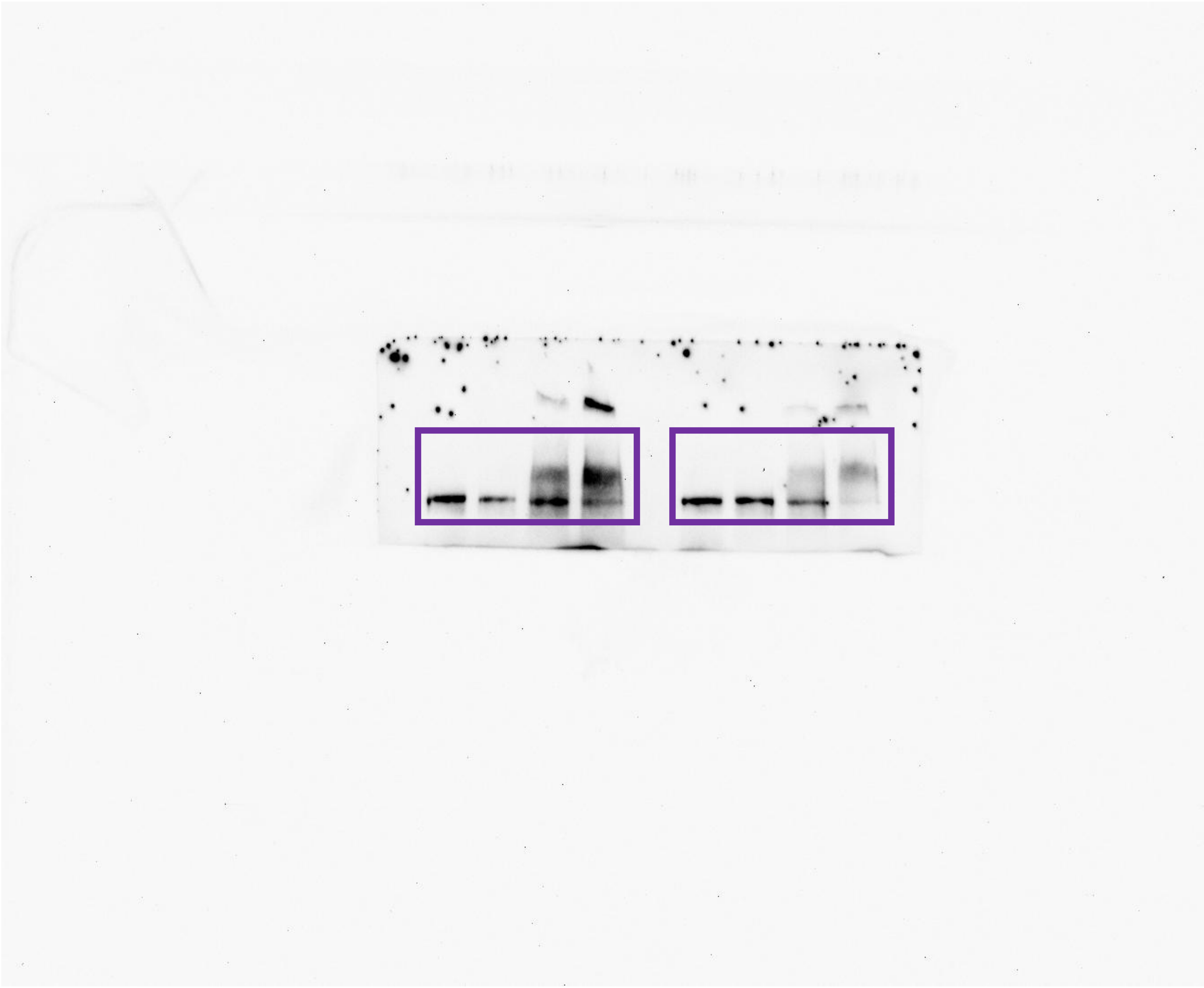

Figure 5I
MHCC97-H Ctrl
DREBRIN

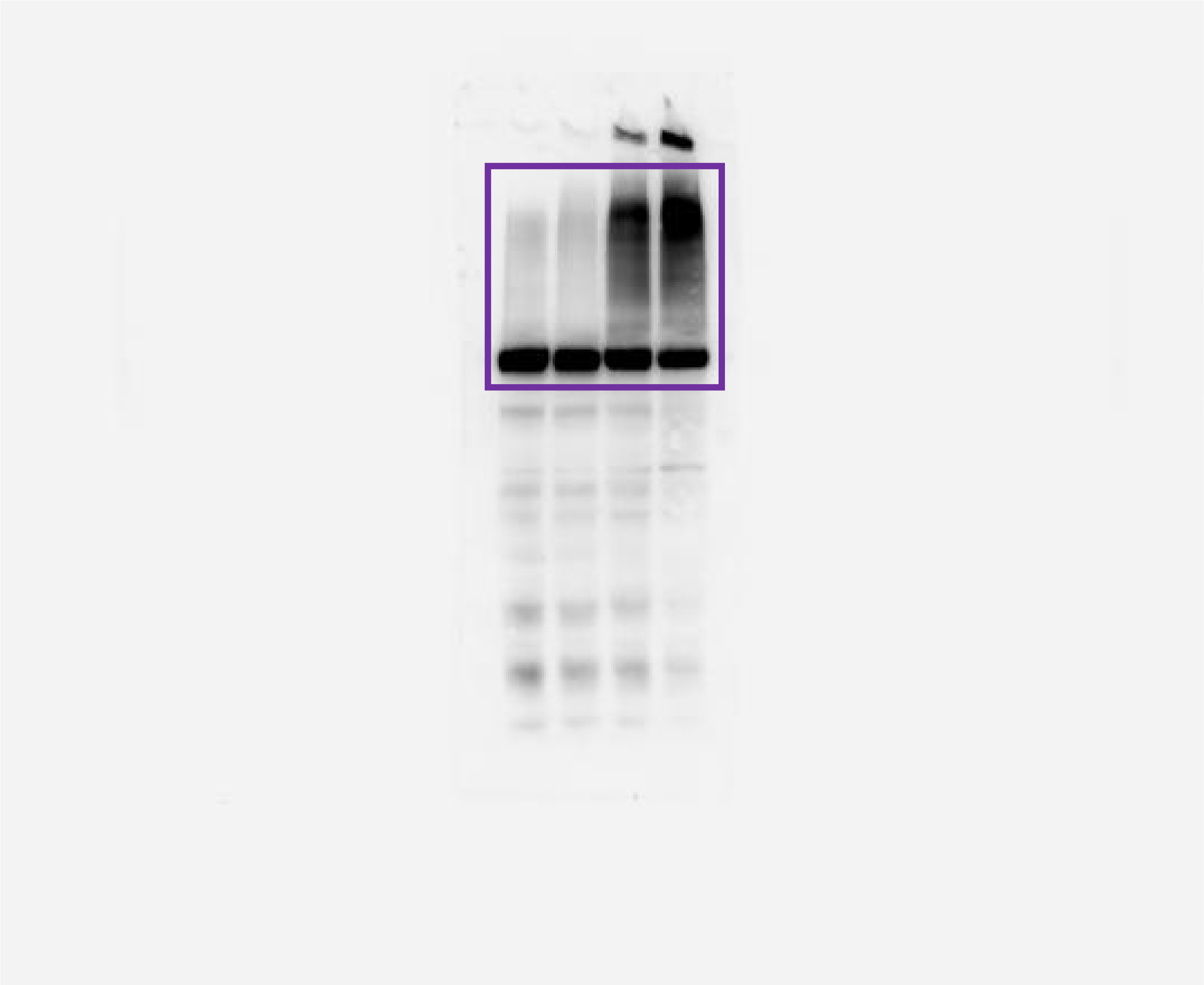

Figure 5I
MHCC97-H Elamipretide
DREBRIN

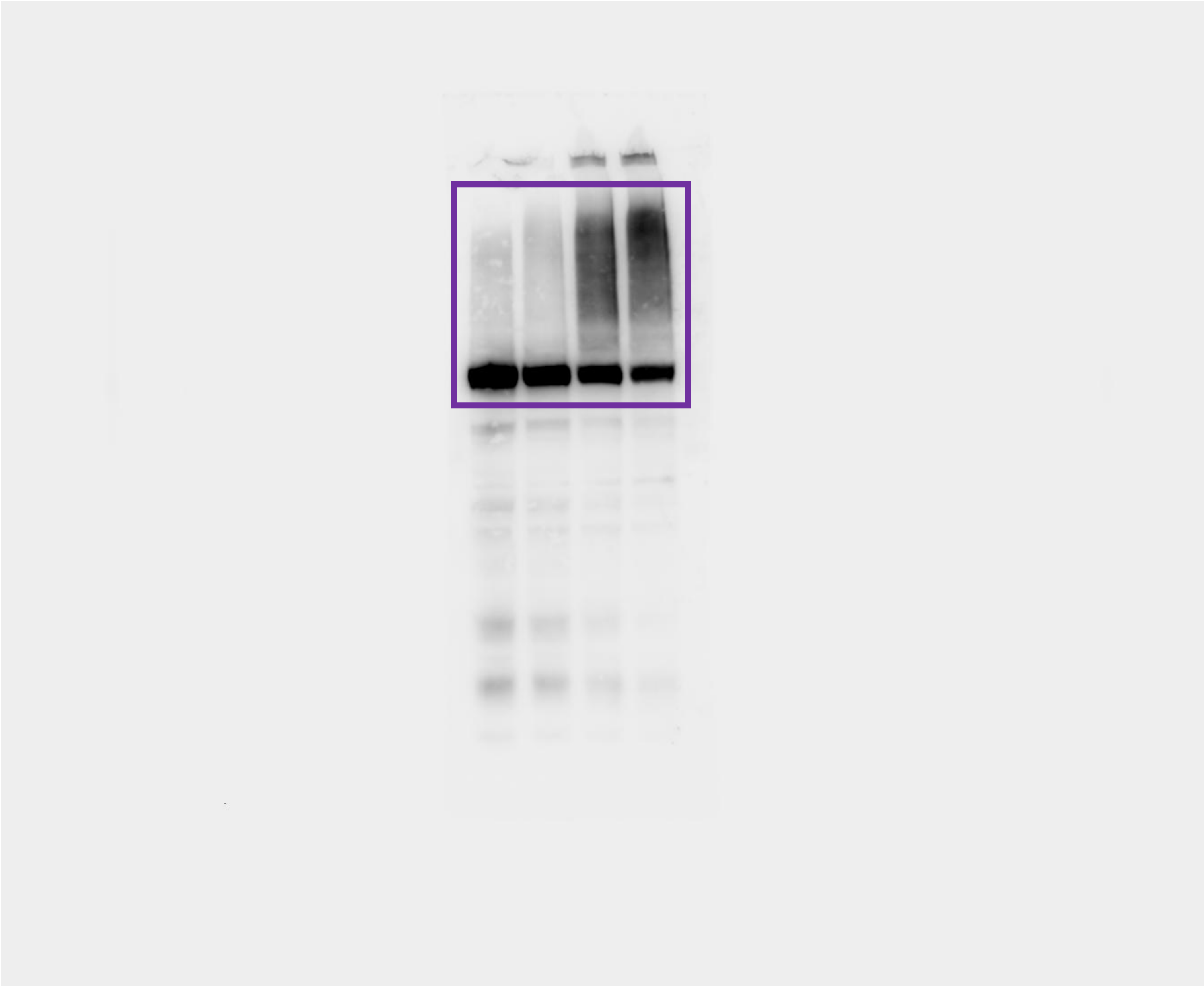

Figure 5I
MHCC97-H Ctrl Elamipretide
GAPDH

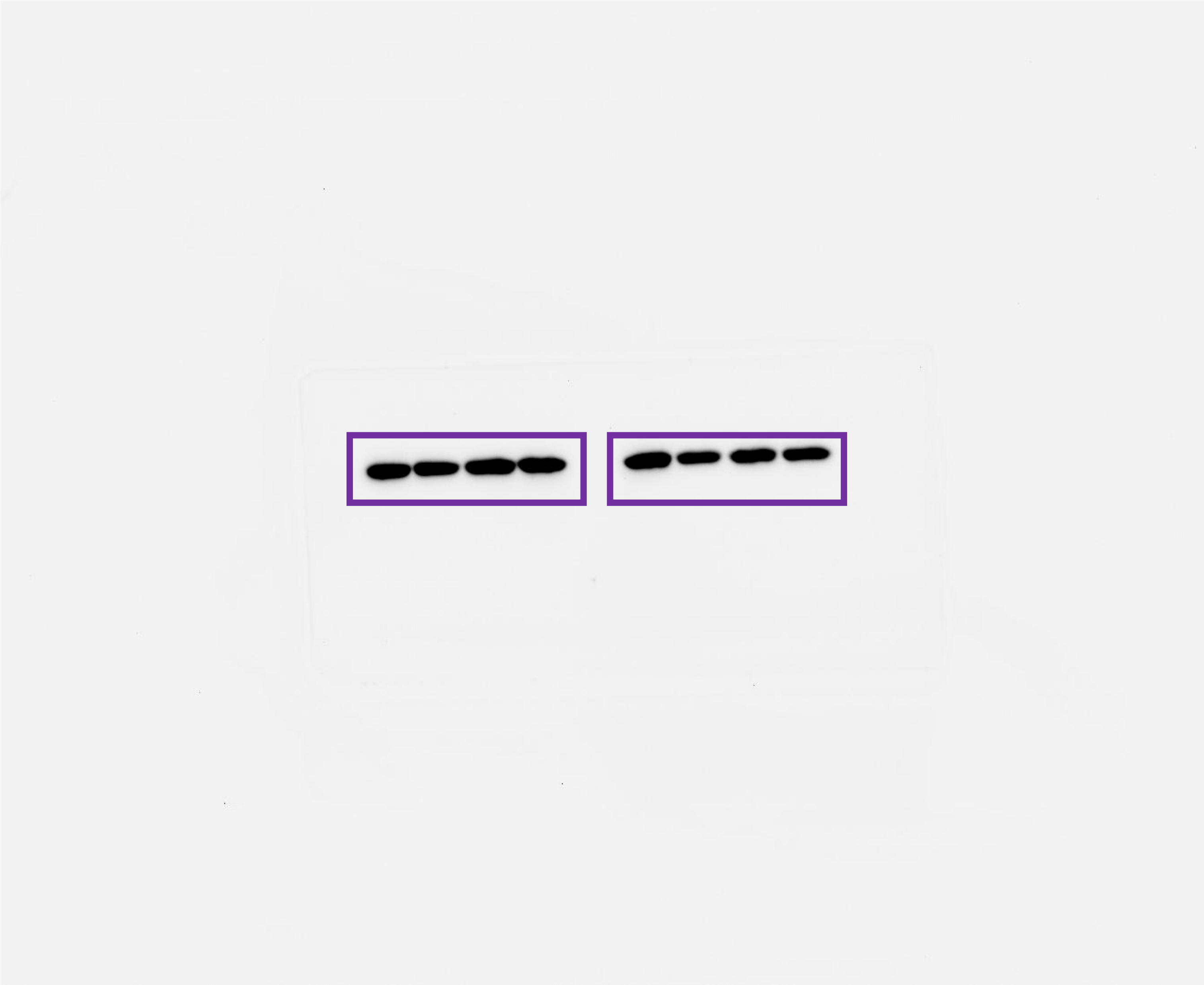

Figure 5J

Hep 3B Ctrl Elamipretide

FLNA

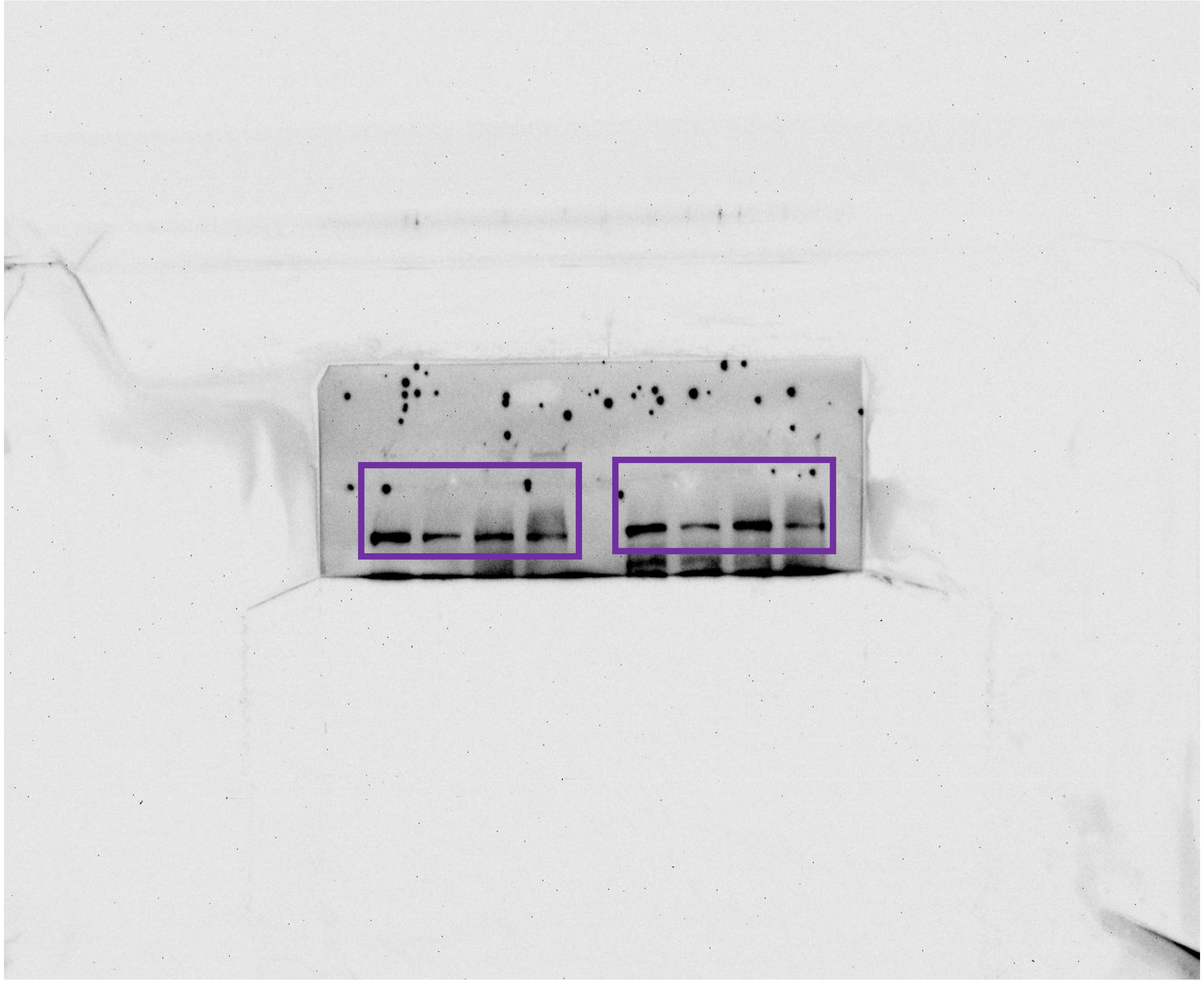

Figure 5J
Hep 3B Ctrl Elamipretide
DREBRIN

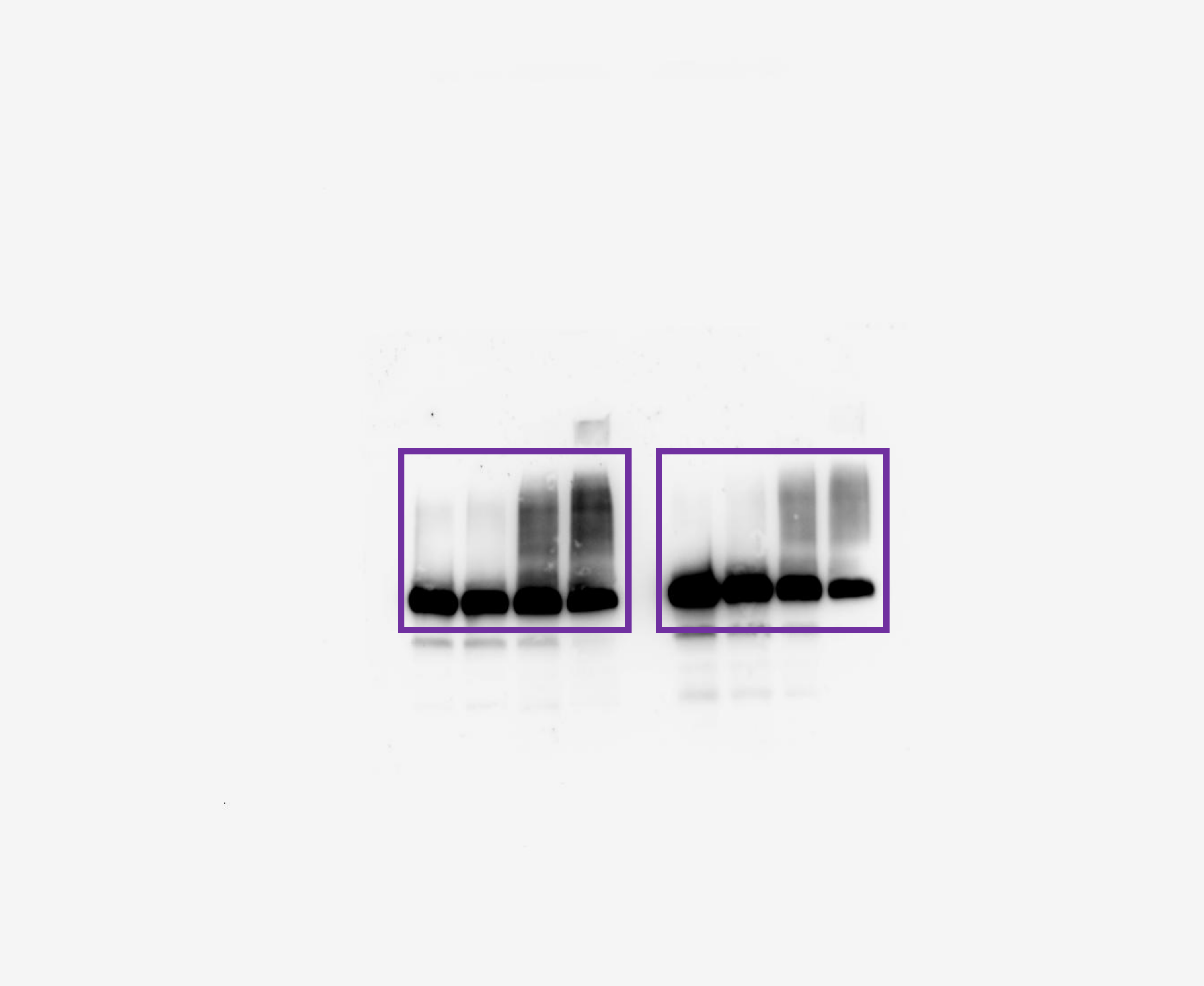

Figure 5J
Hep 3B Ctrl Elamipretide
GAPDH

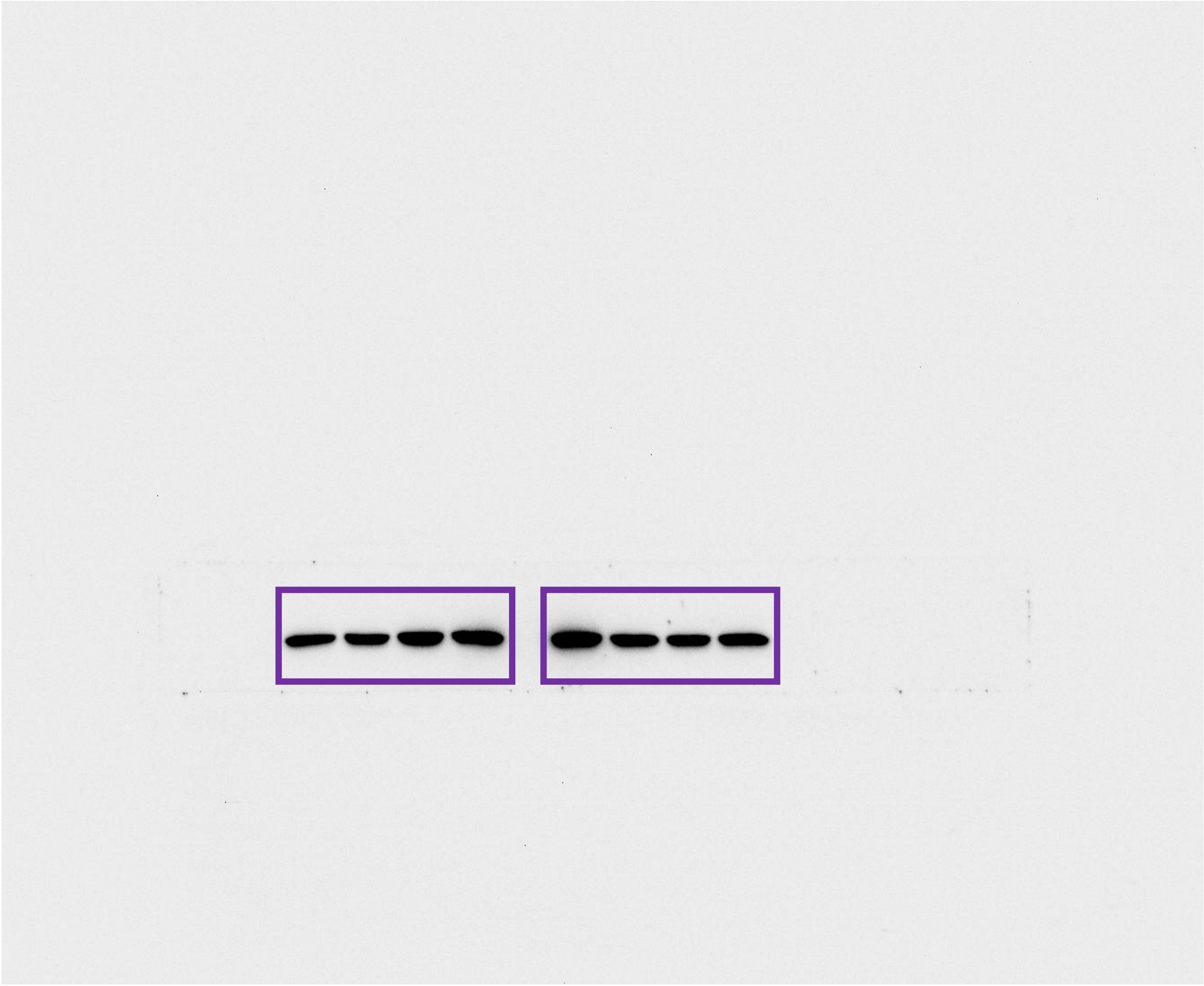

Figure 6C

MHCC97-H MLT FLNA

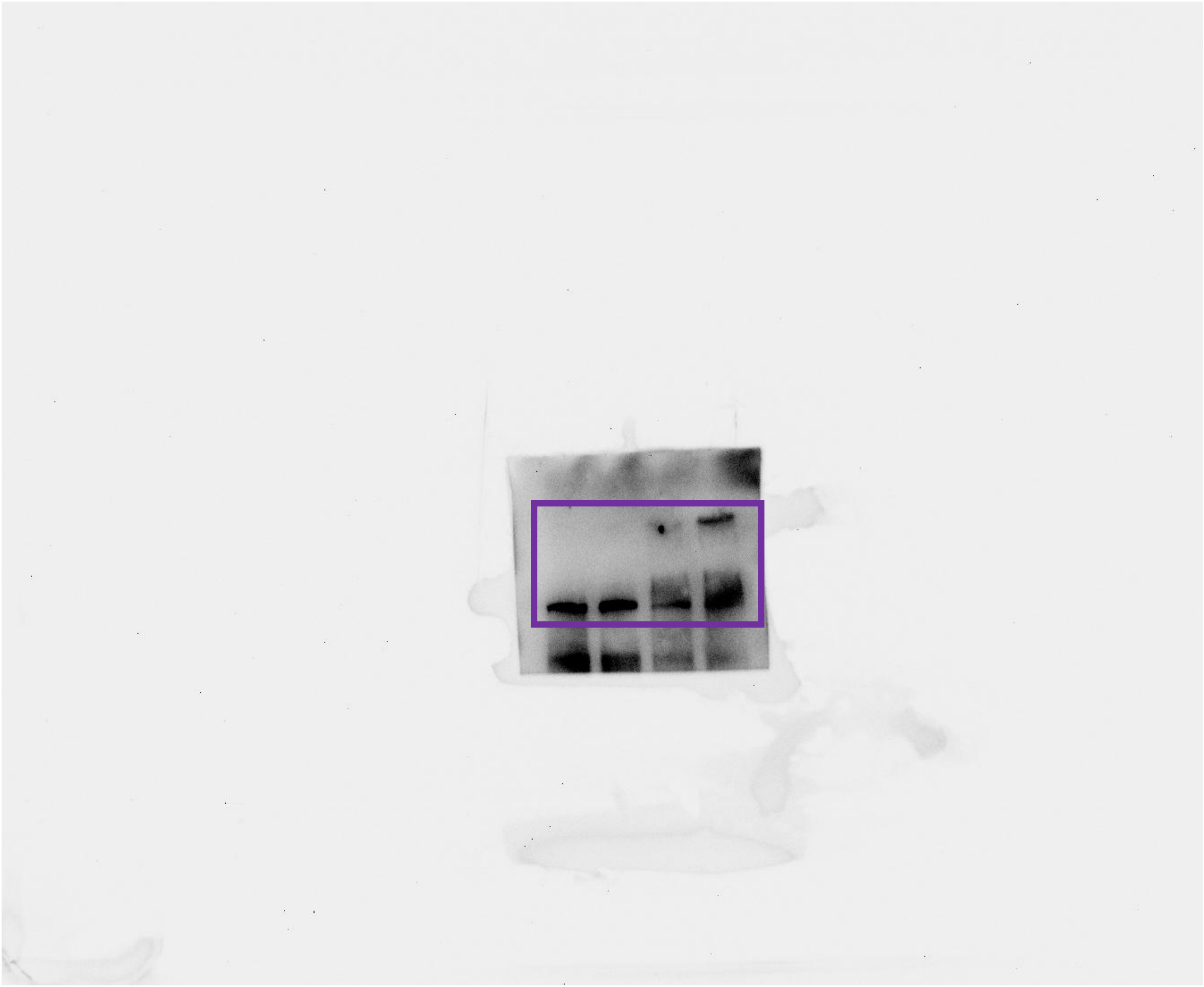

Figure 6C
MHCC97-H 4PA FLNA

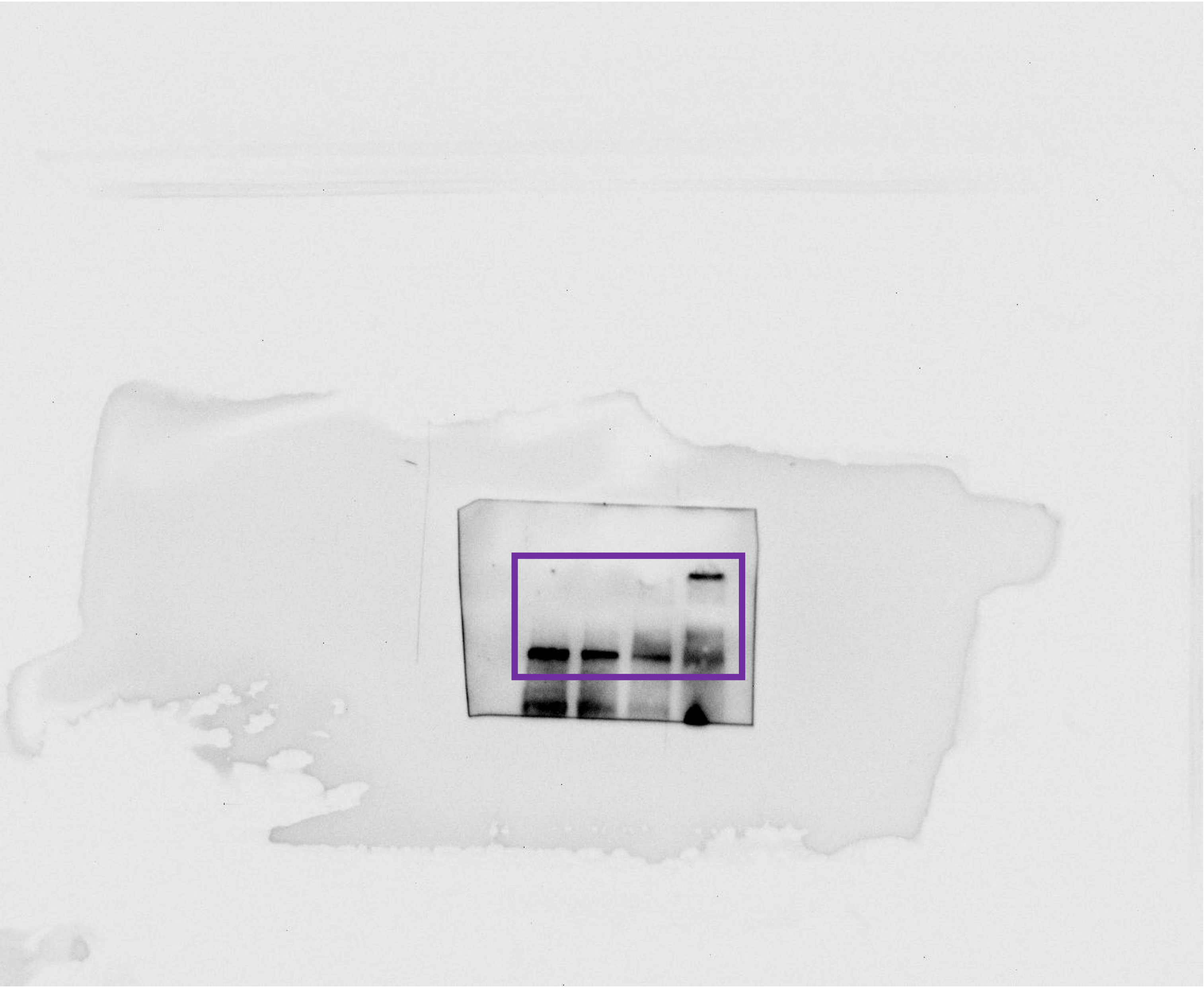

Figure 6C
MHCC97-H CCN FLNA

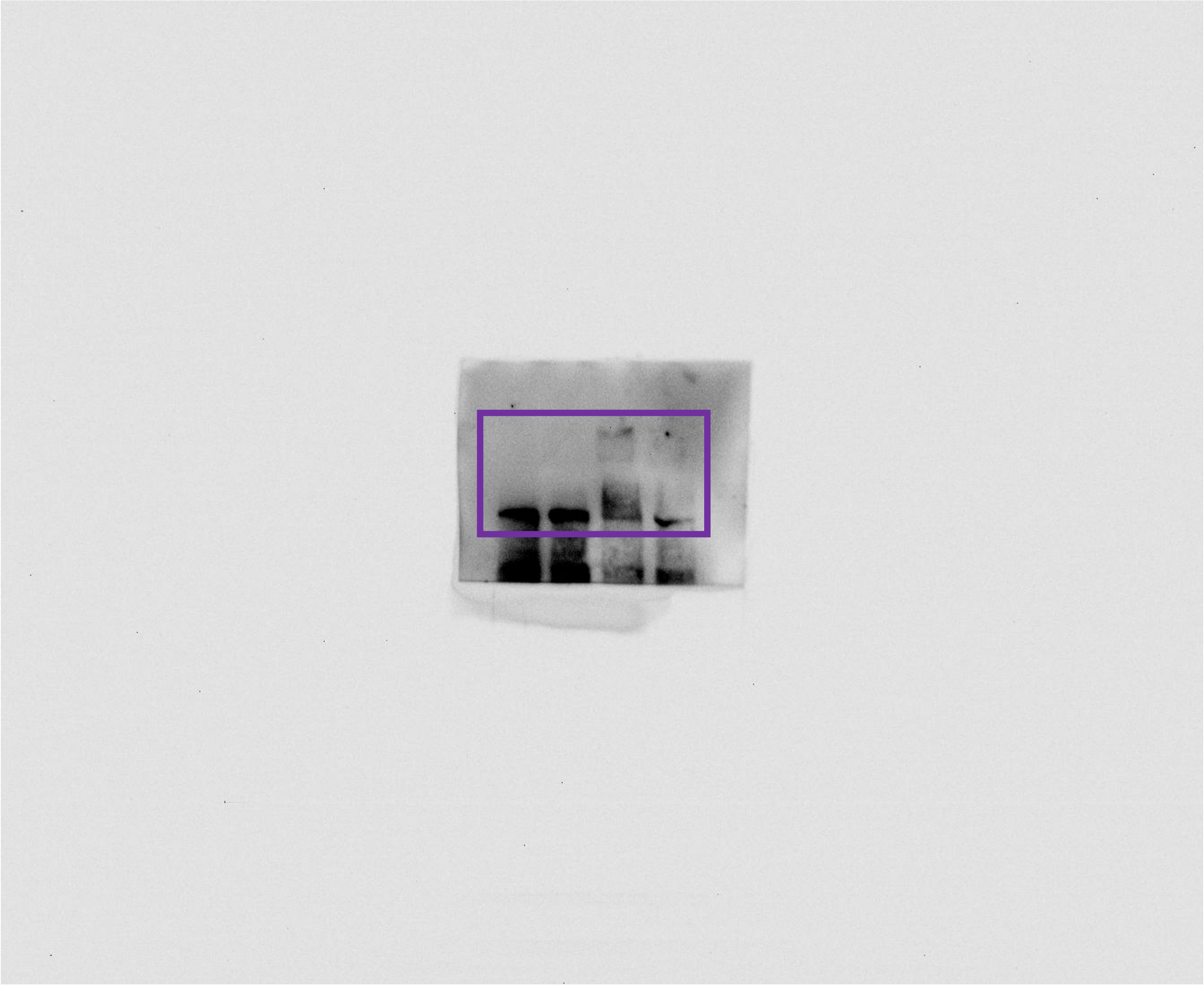

Figure 6C
MHCC97-H 4PA MLT CCN
DREBRIN

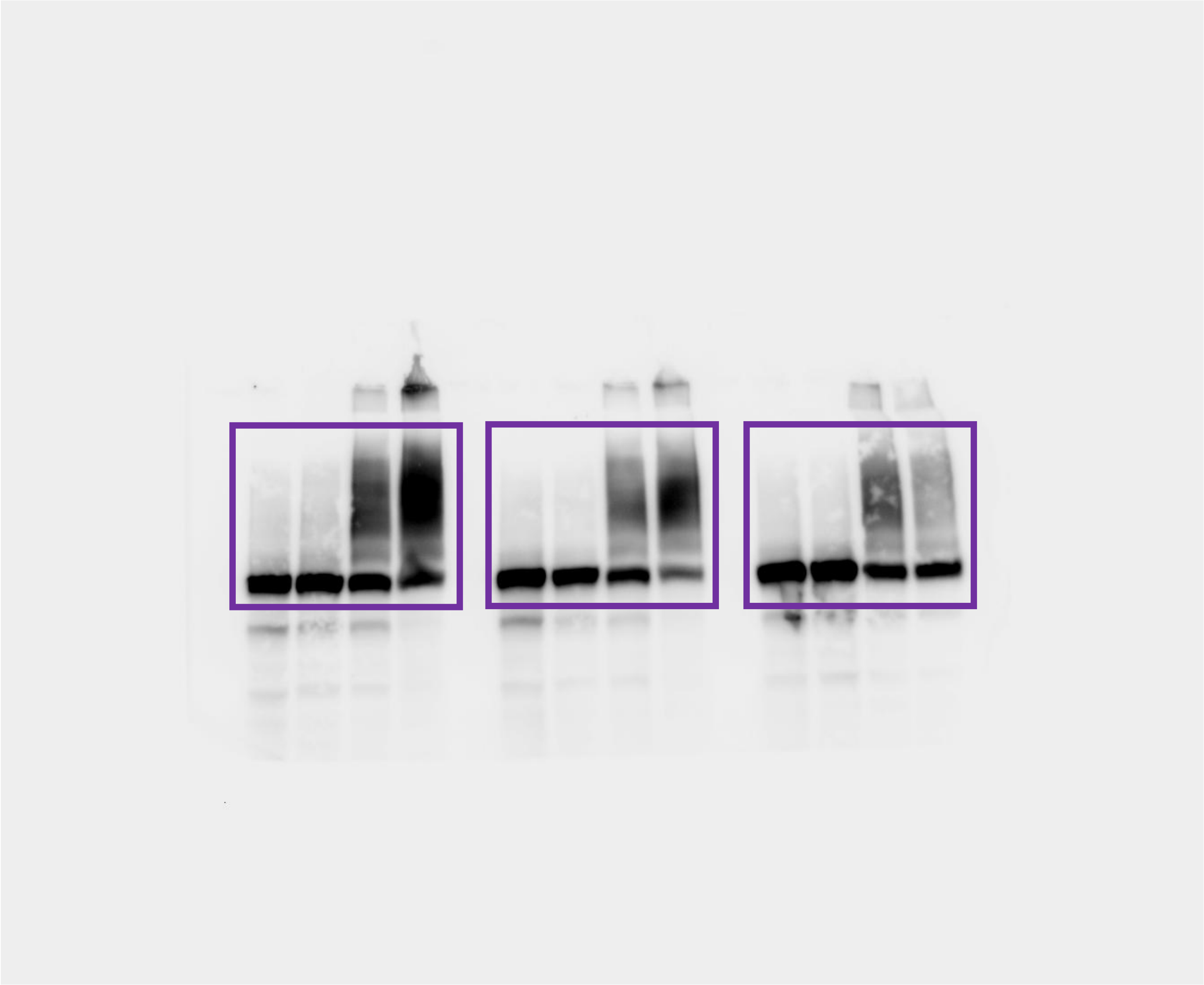

Figure 6C
MHCC97-H 4PA MLT CCN
GAPDH

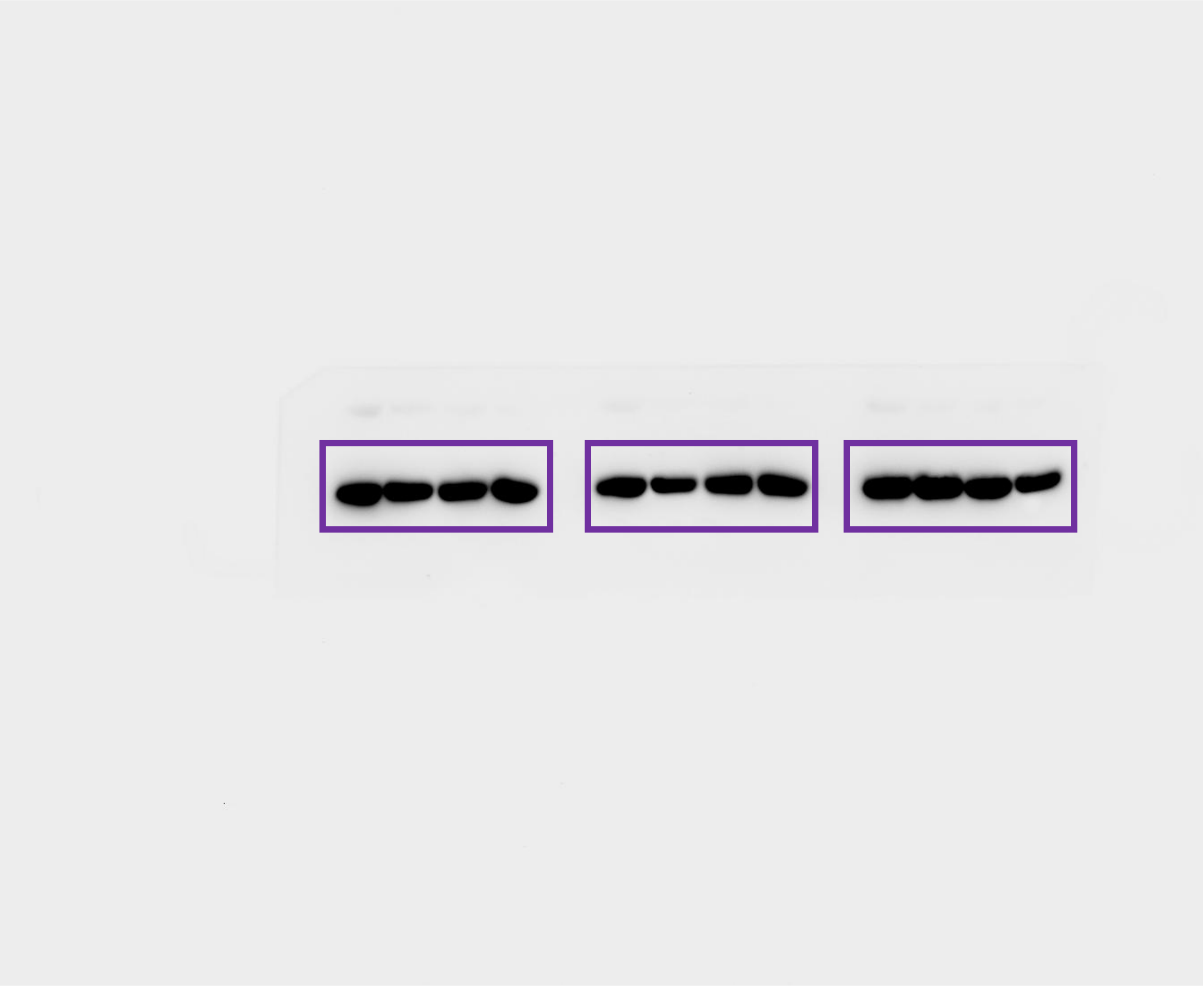

Figure 6C
Hep 3B 4PA MLT
FLNA

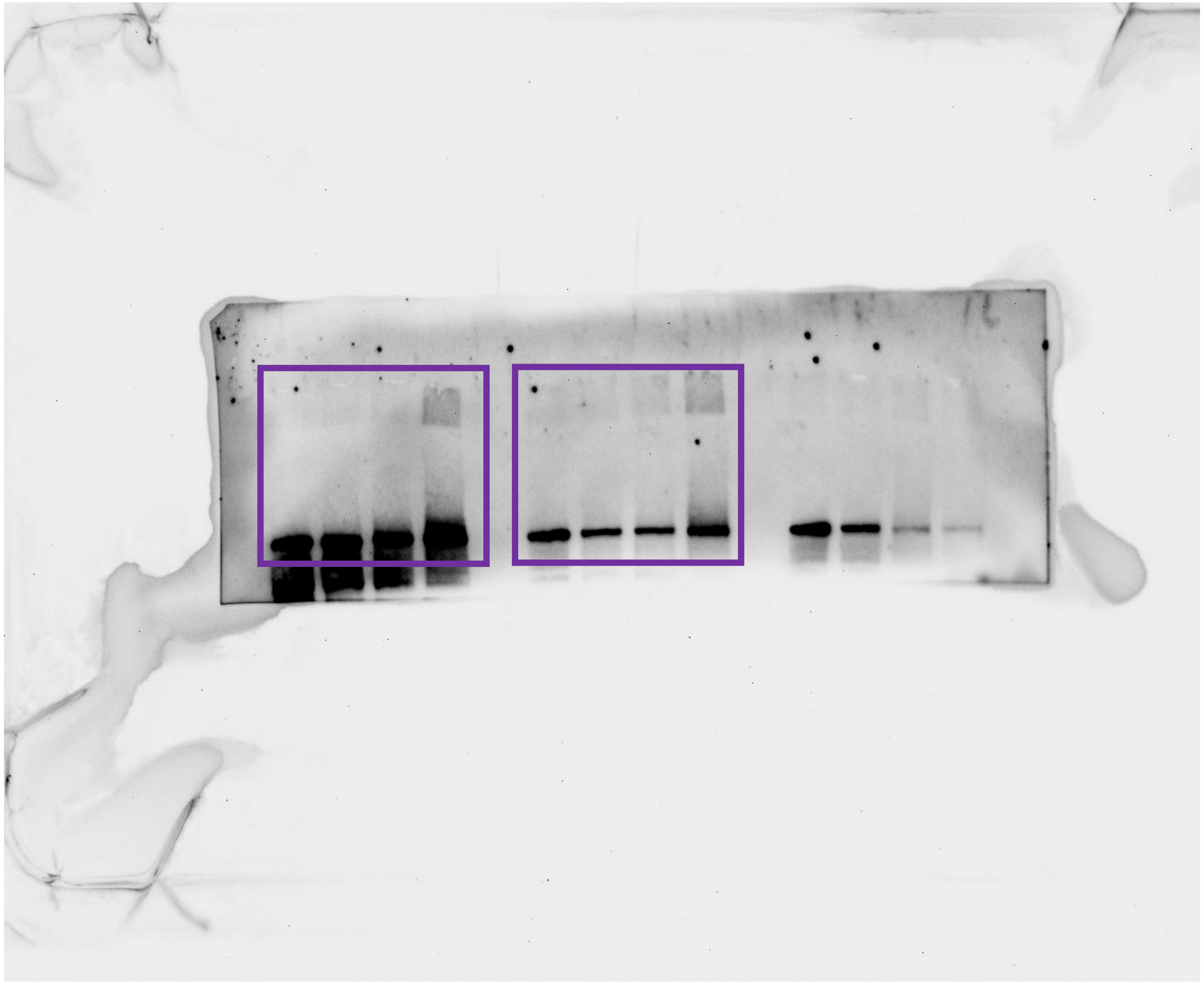

Figure 6C
Hep 3B CCN
FLNA

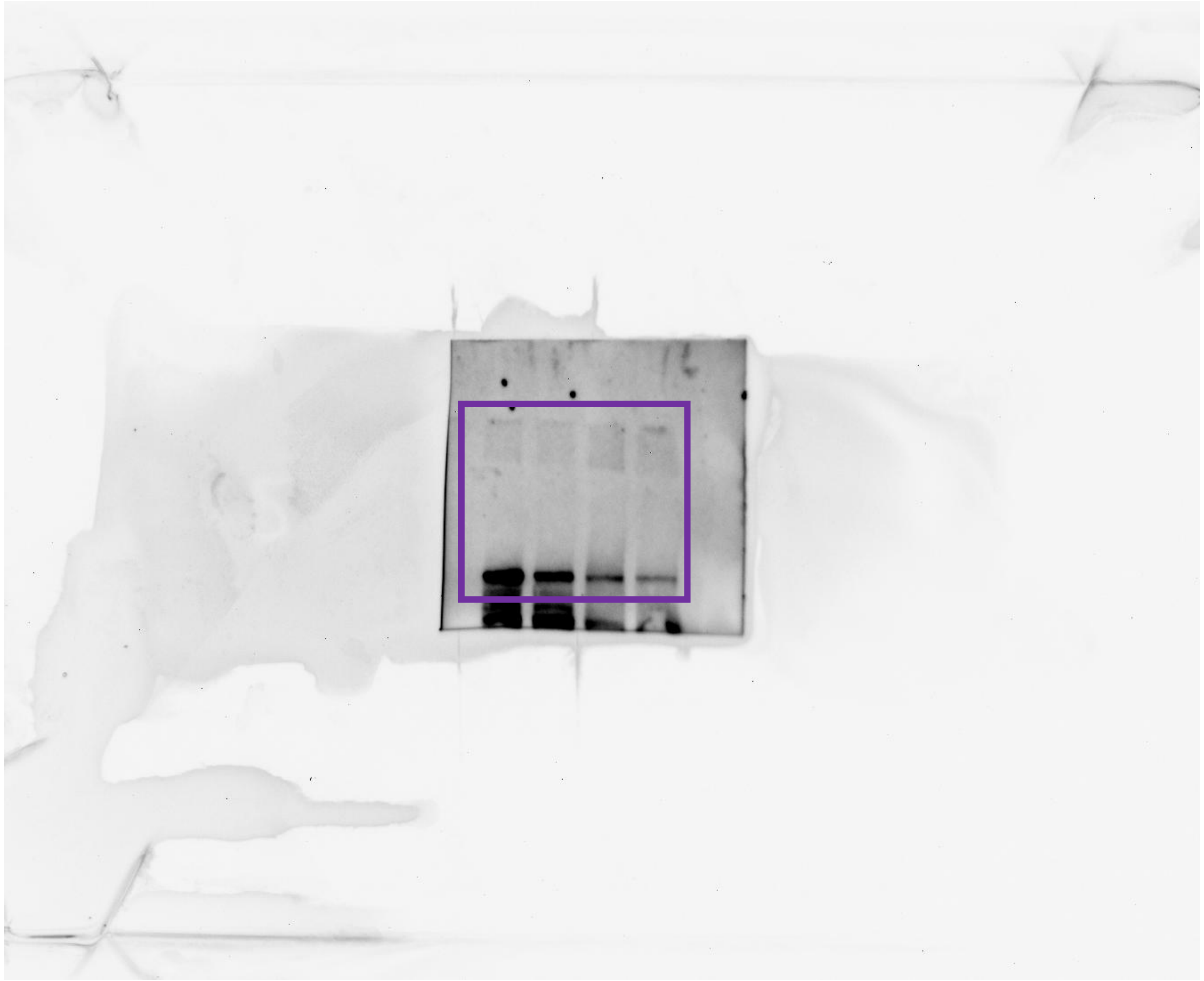

Figure 6C
Hep 3B 4PA MLT
DREBRIN

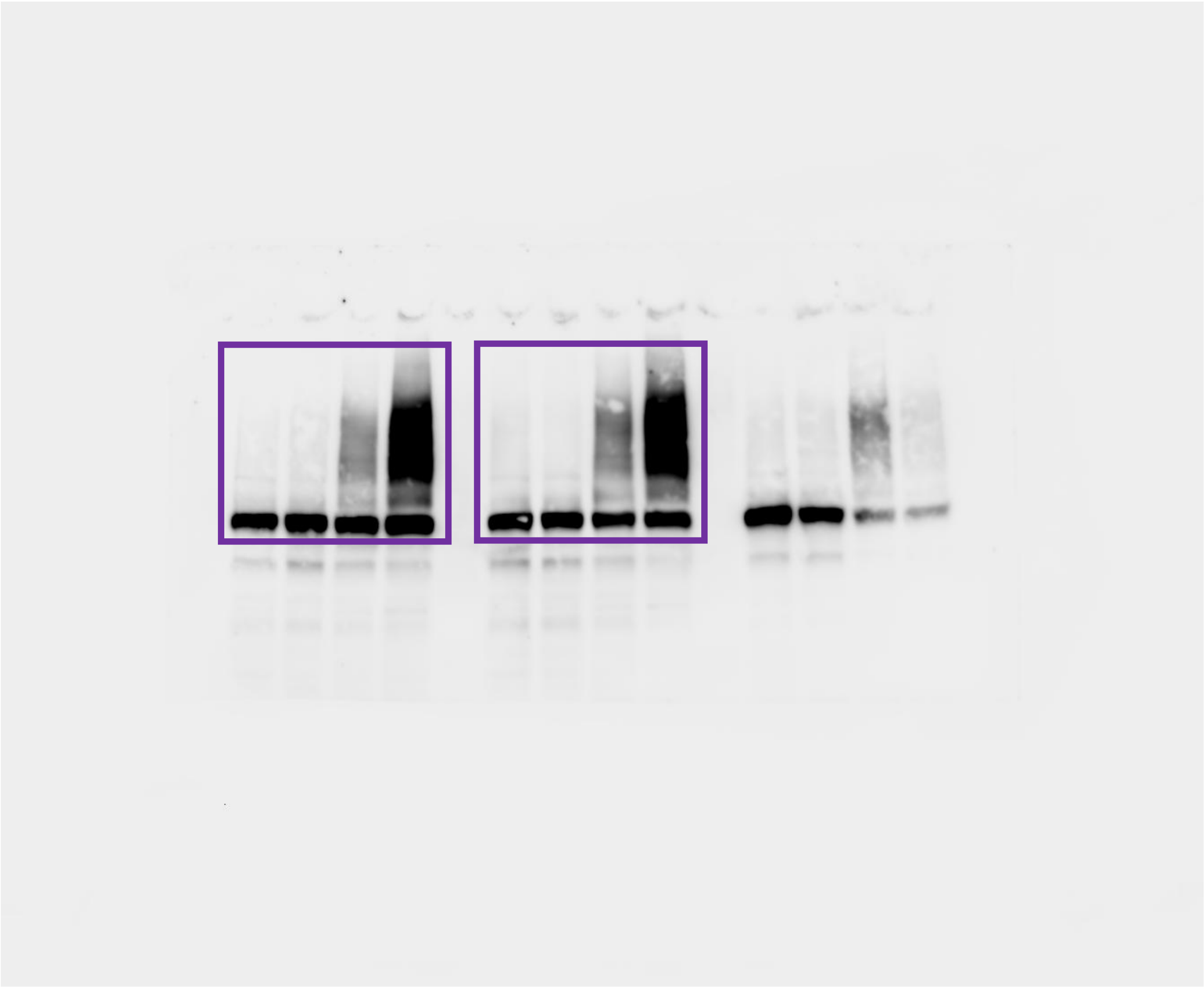

Figure 6C
Hep 3B CCN
DREBRIN

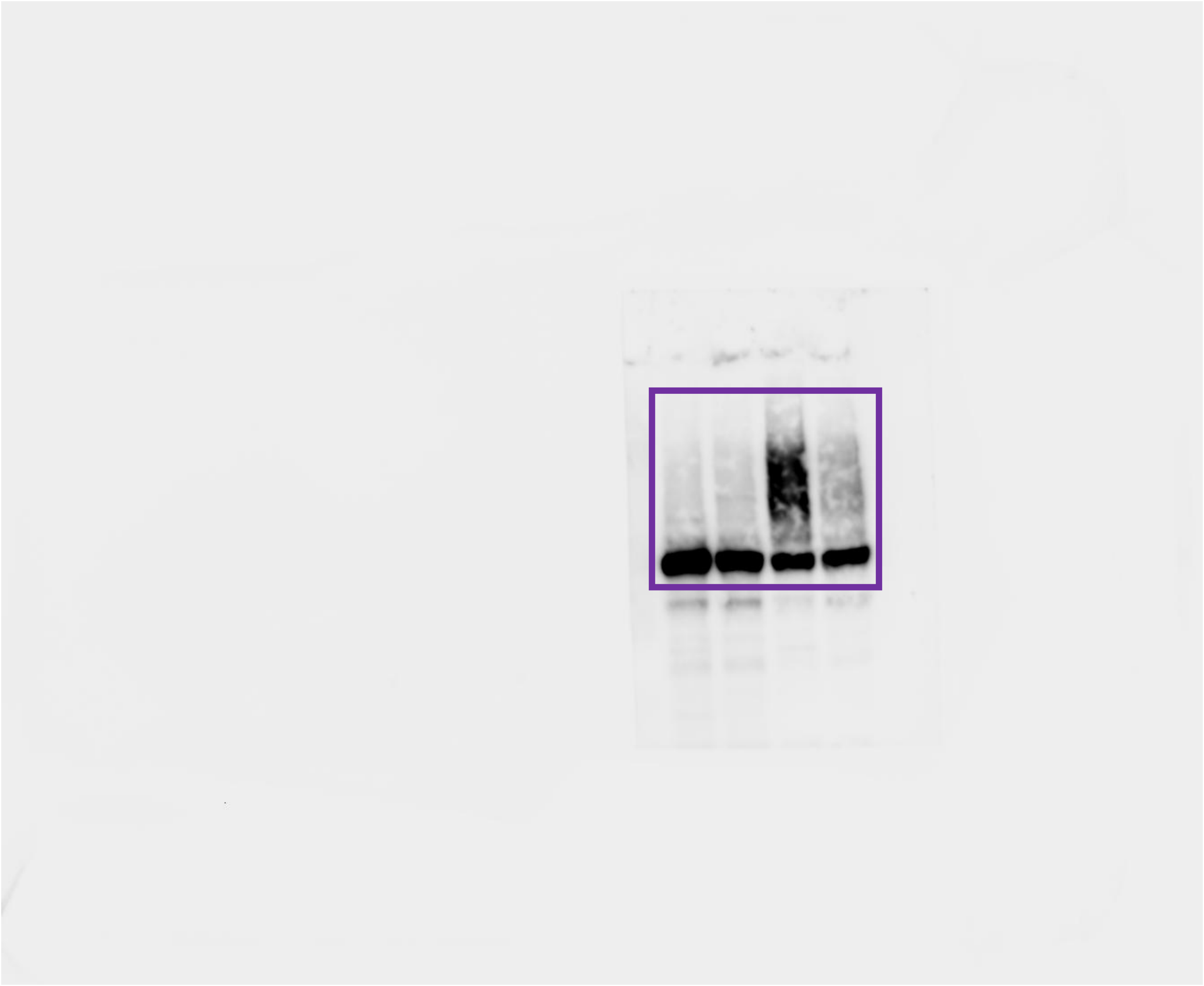

Figure 6C
Hep 3B 4PA MLT CCN
GAPDH

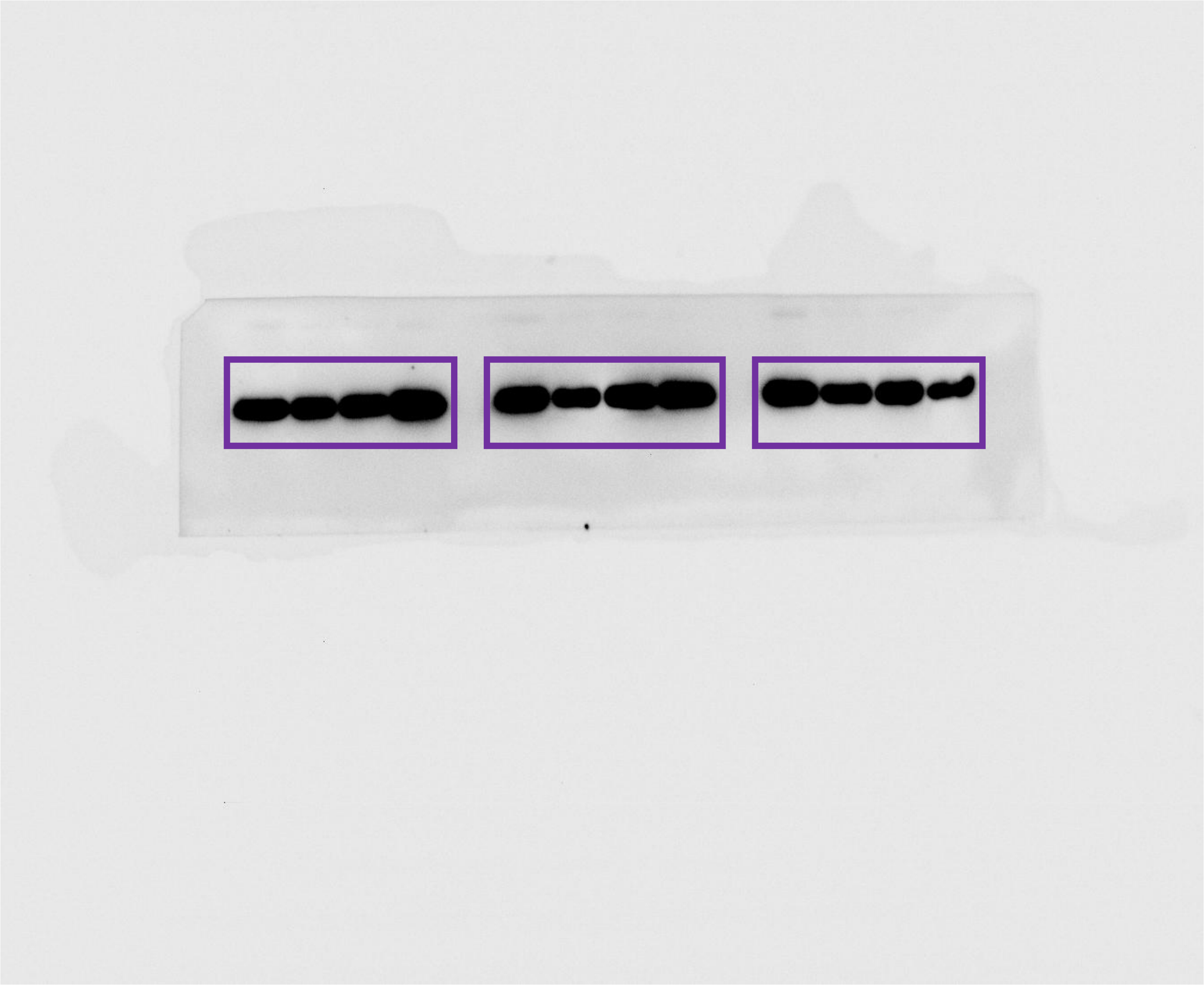

Figure 6D
MHCC97-H ATG7 KO
4PA   FLNA

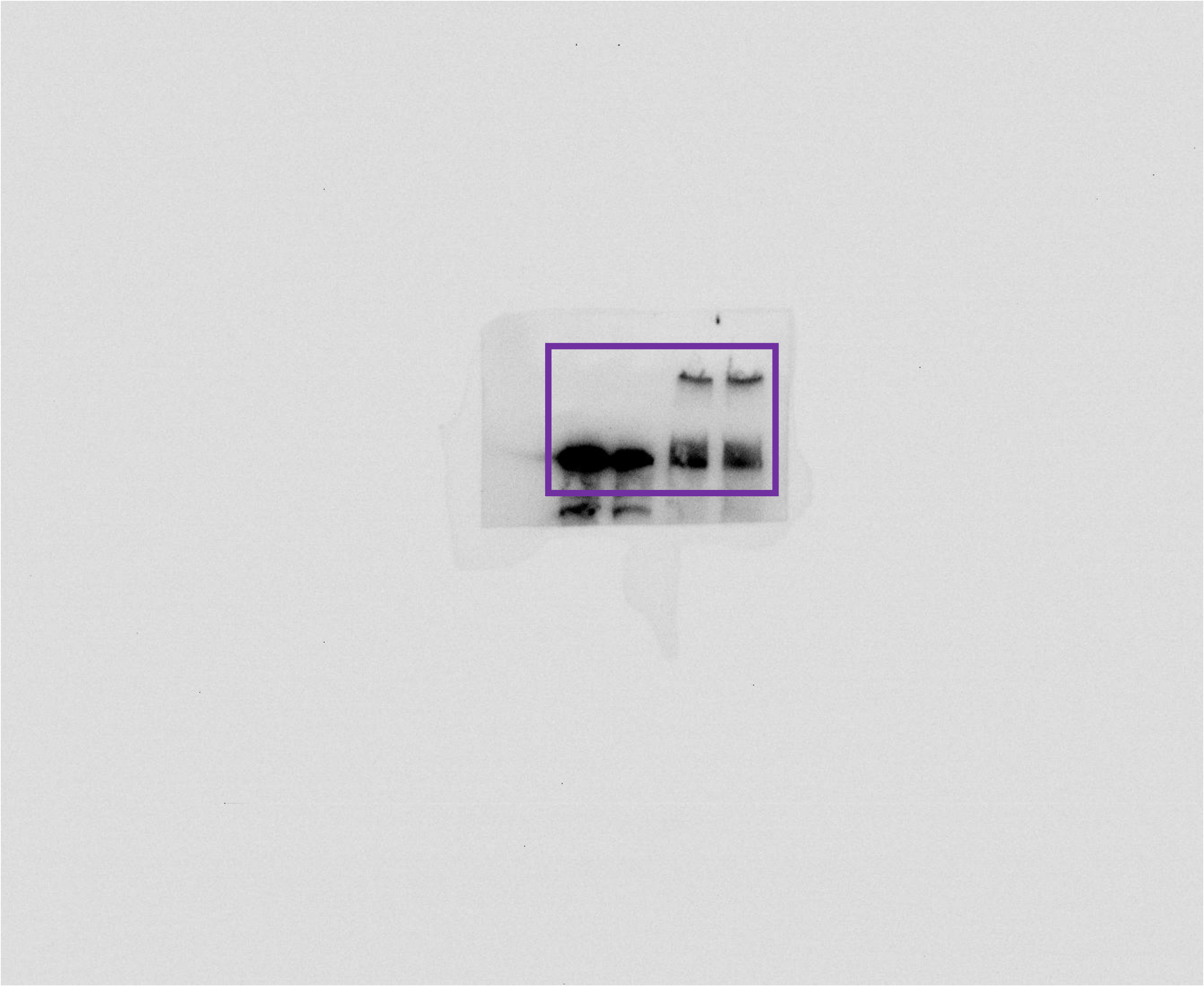

Figure 6D
MHCC97-H ATG7 KO
MLT FLNA

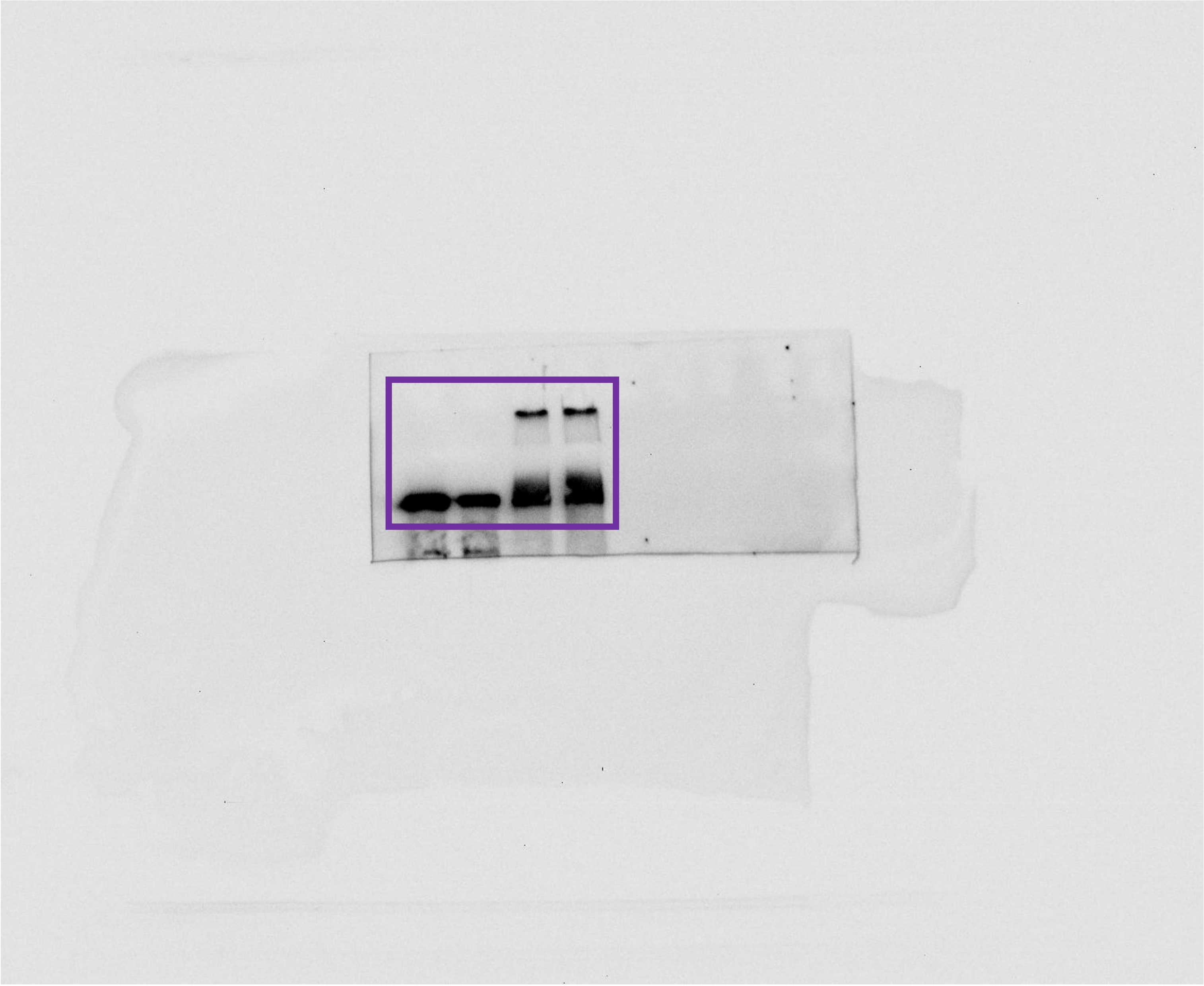

Figure 6D
MHCC97-H ATG7 KO
4PA MLT FLNA

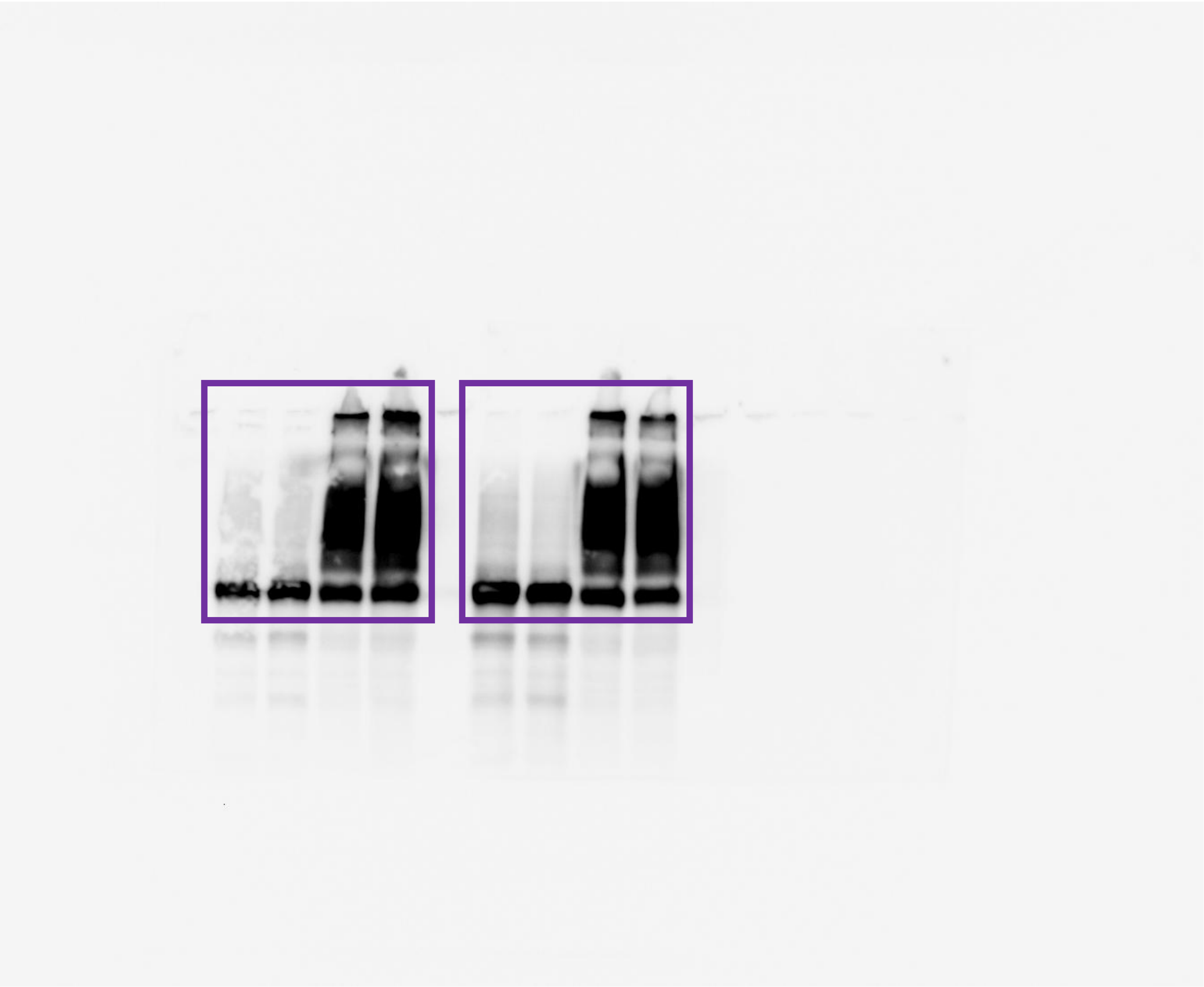

Figure 6D
MHCC97-H ATG7 KO
4PA MLT GAPDH

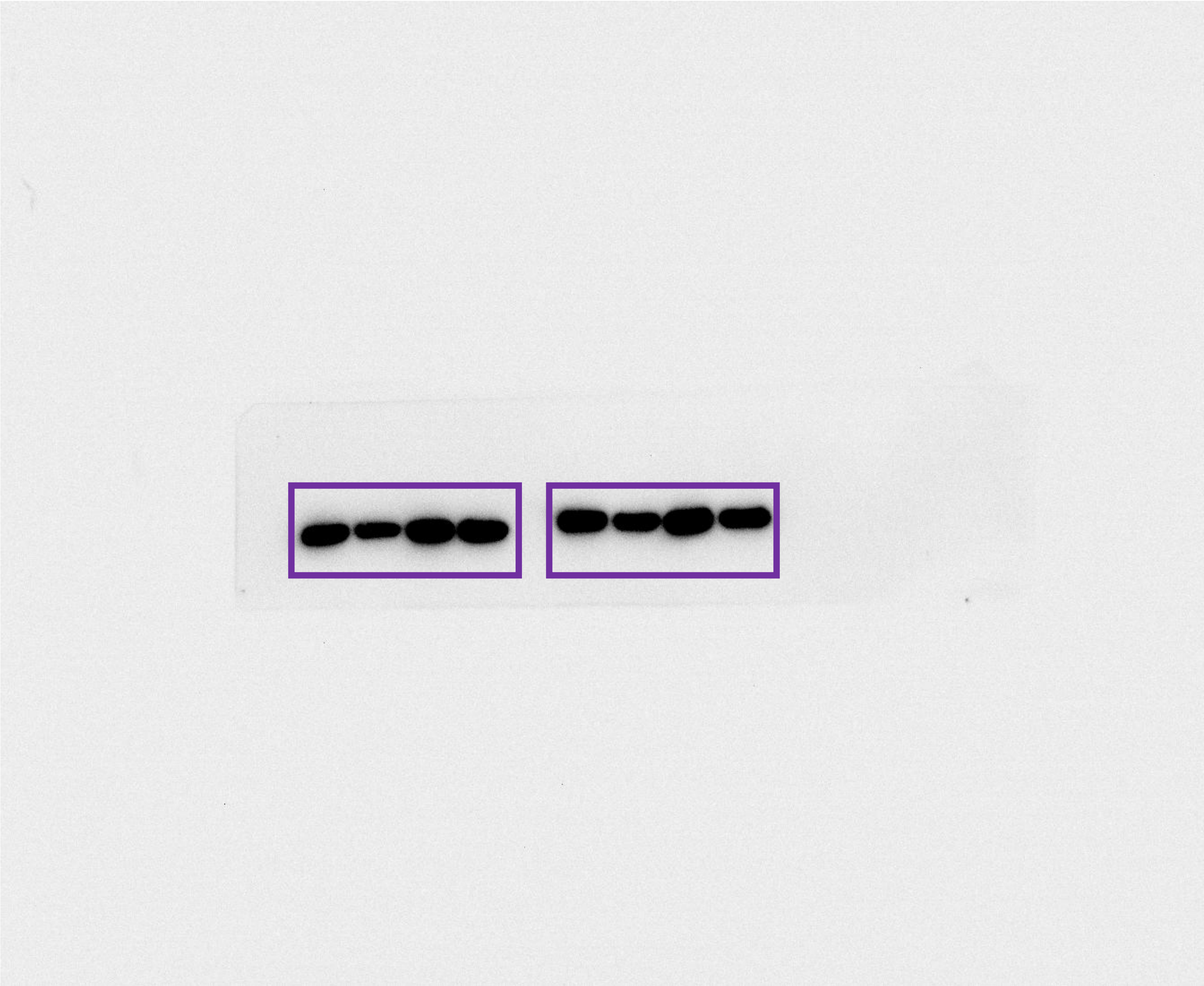

Figure 6D
Hep 3B ATG7 KO
MLT   FLNA

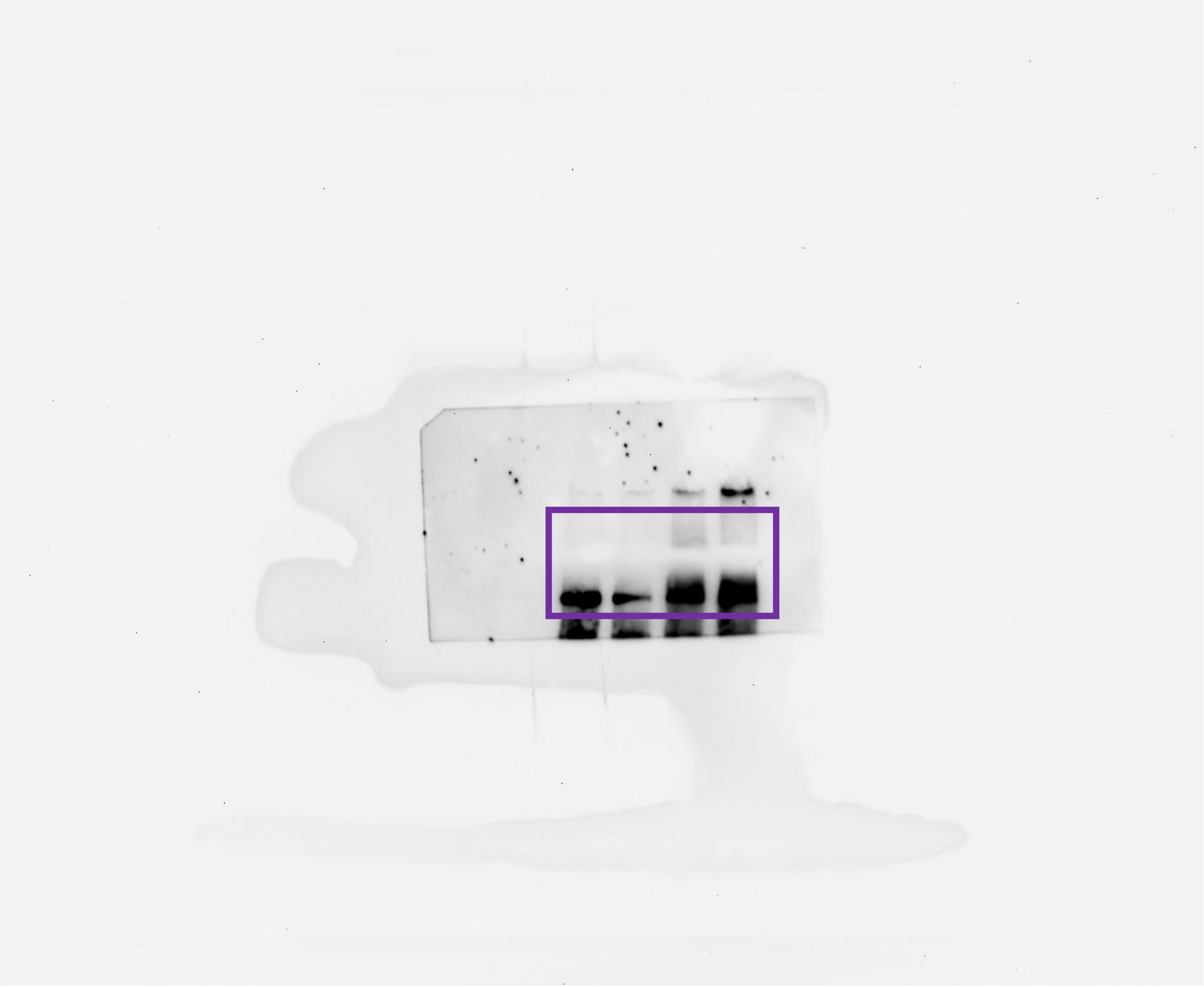

Figure 6D
Hep 3B ATG7 KO
4PA   FLNA

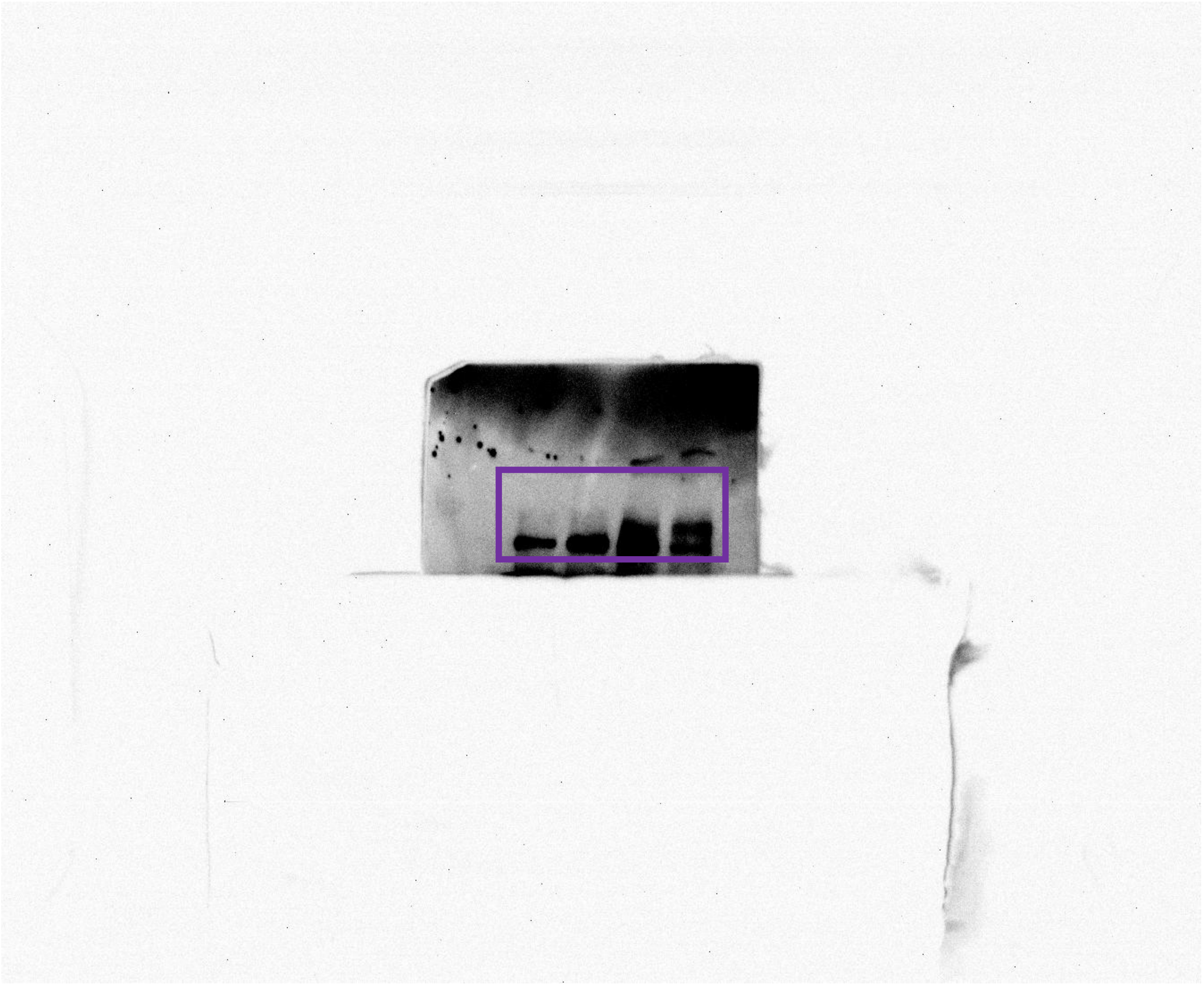

Figure 6D
Hep 3B ATG7 KO
4PA MLT   DREBRIN

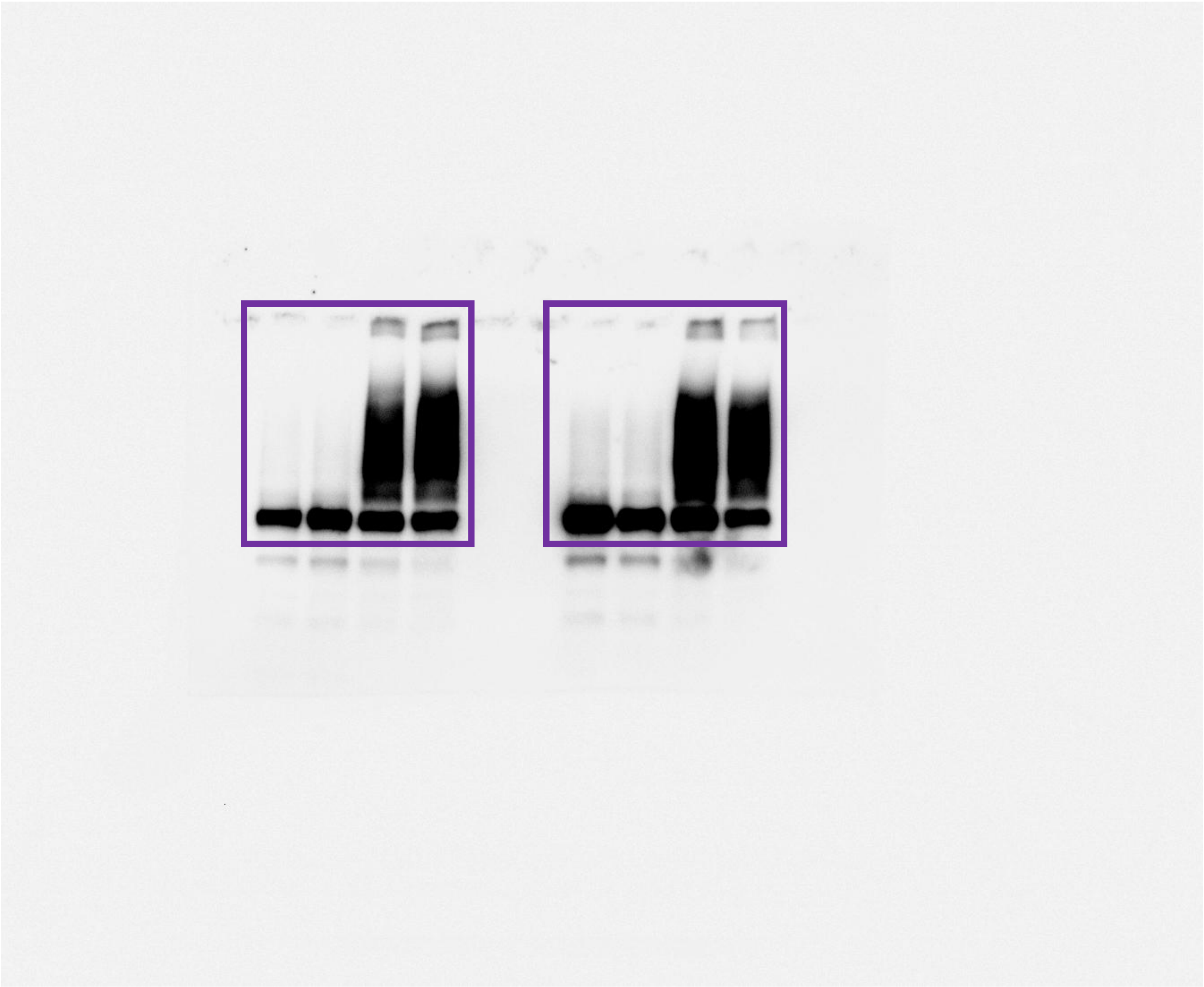

Figure 6D
Hep 3B ATG7 KO
4PA MLT   GAPDH

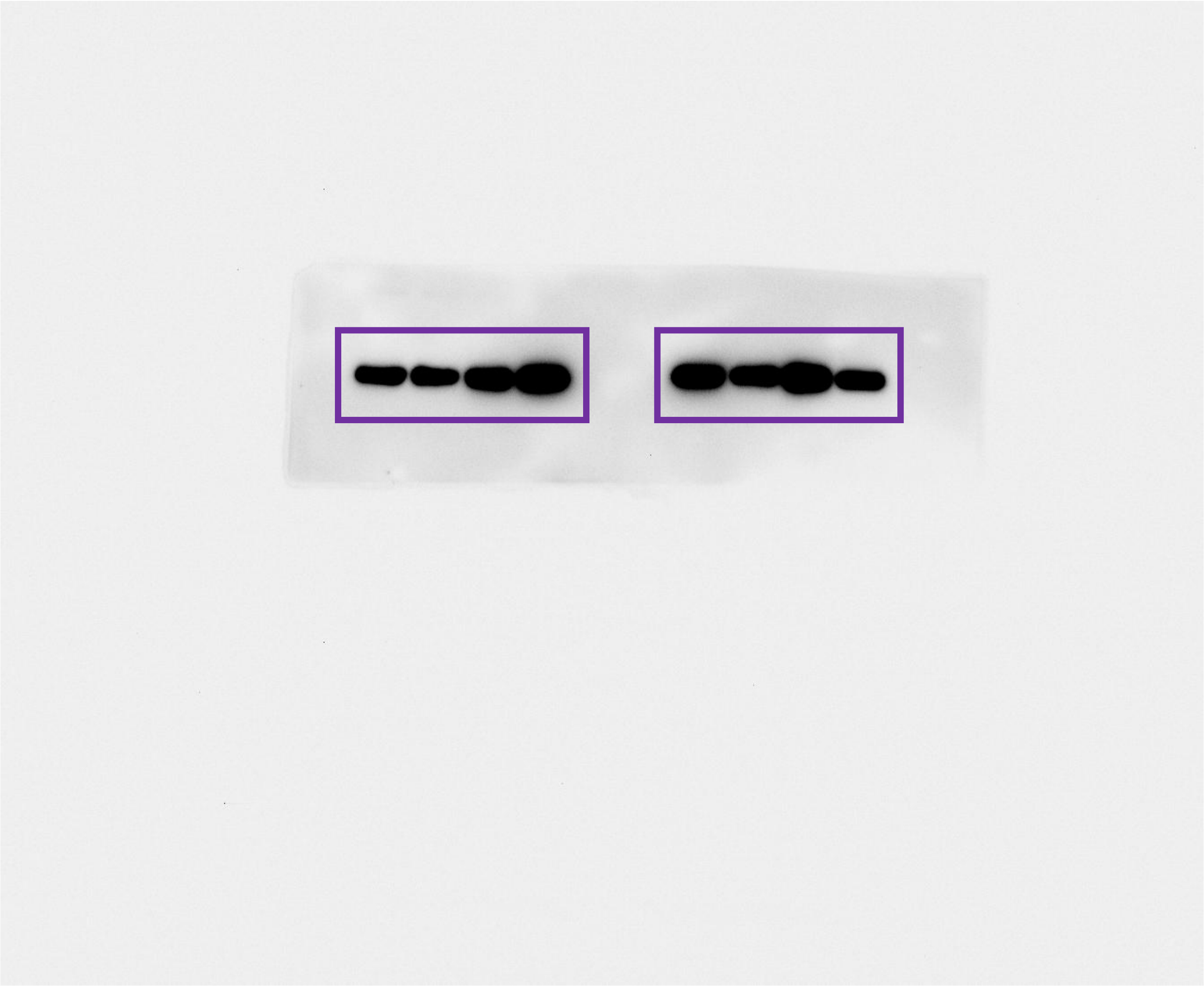

Figure 7B
MHCC97-H   ATF4

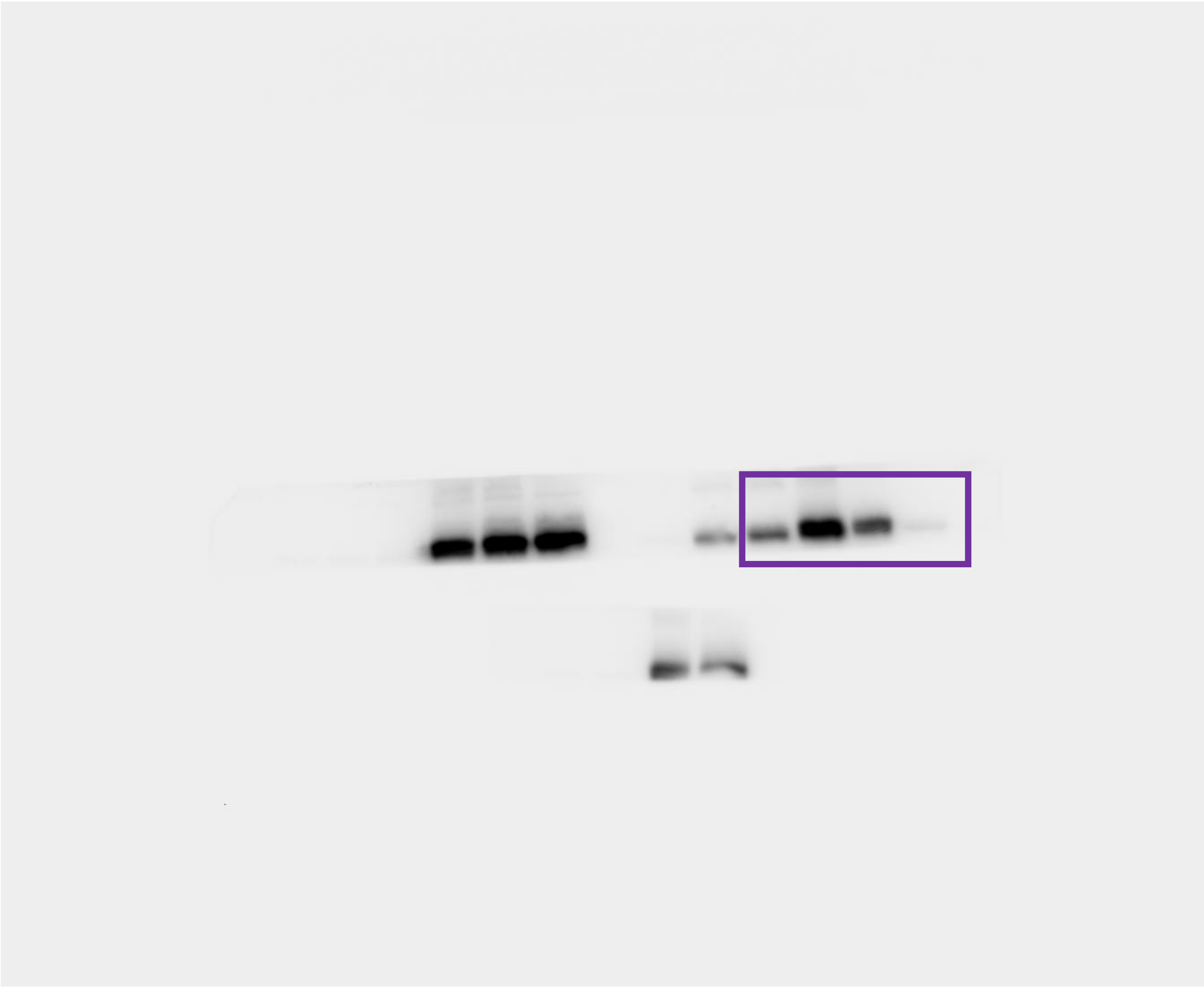

Figure 7B
MHCC97-H p-eIF2α

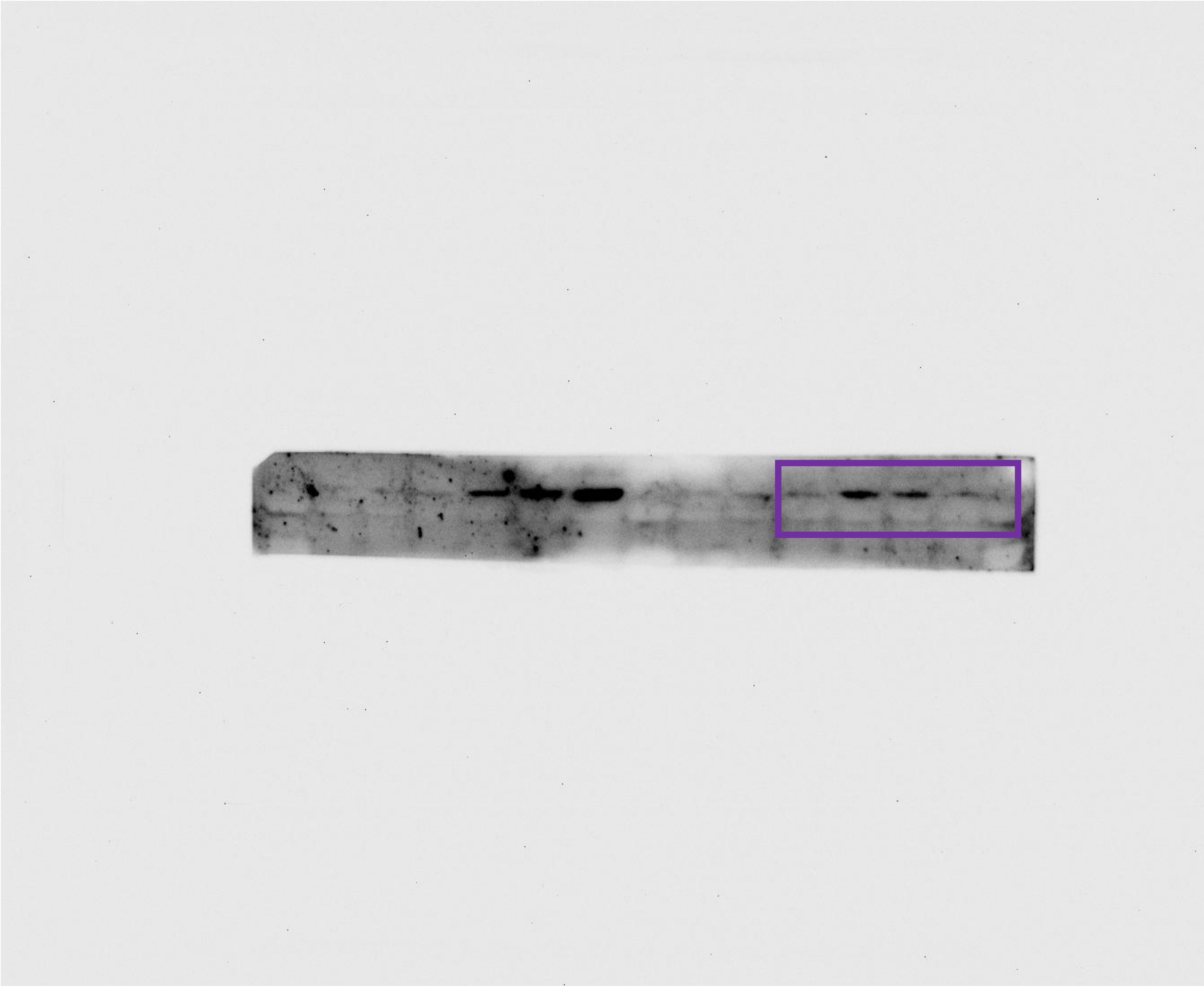

Figure 7B
MHCC97-H eIF2α

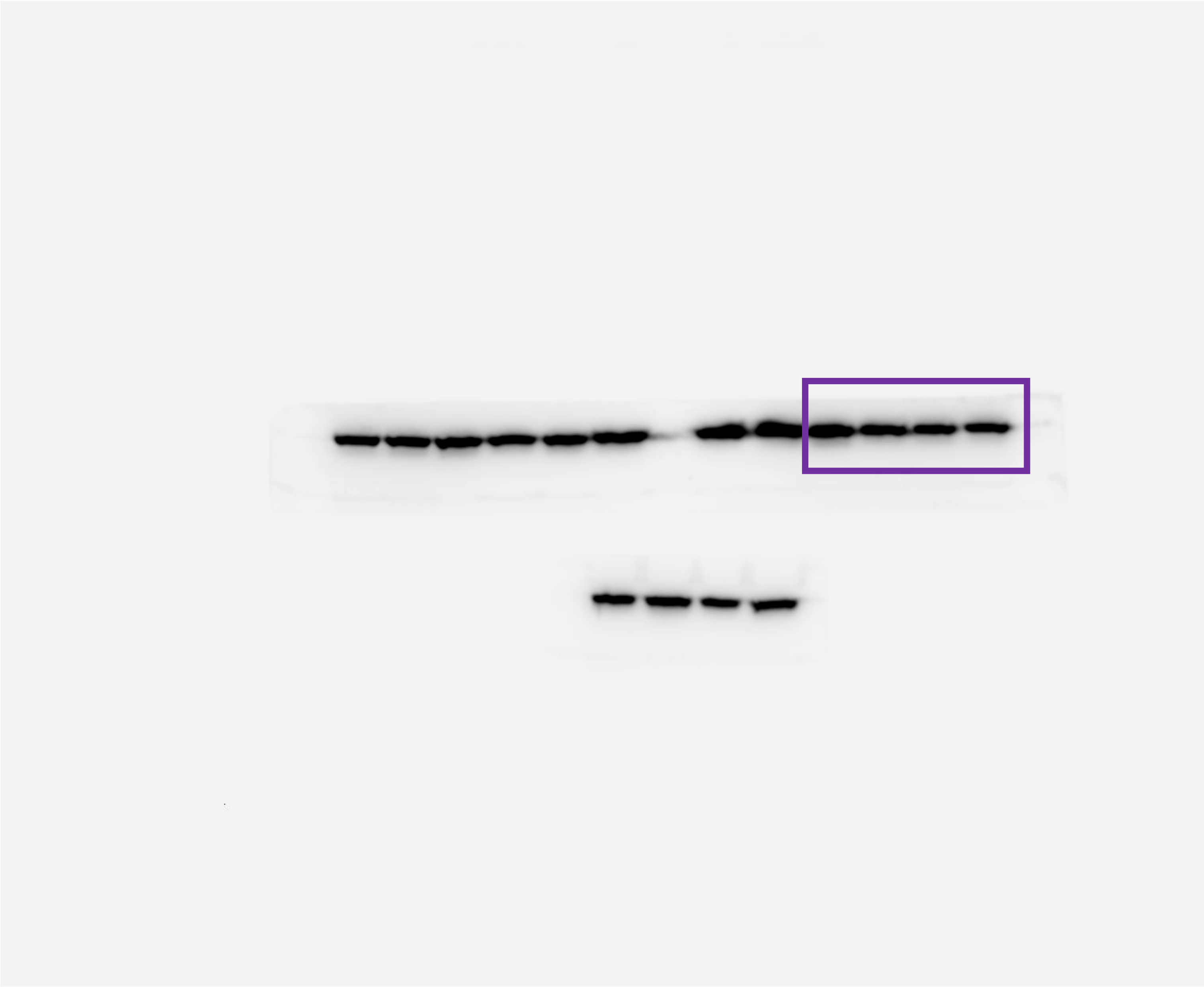

Figure 7B
MHCC97-H GAPDH

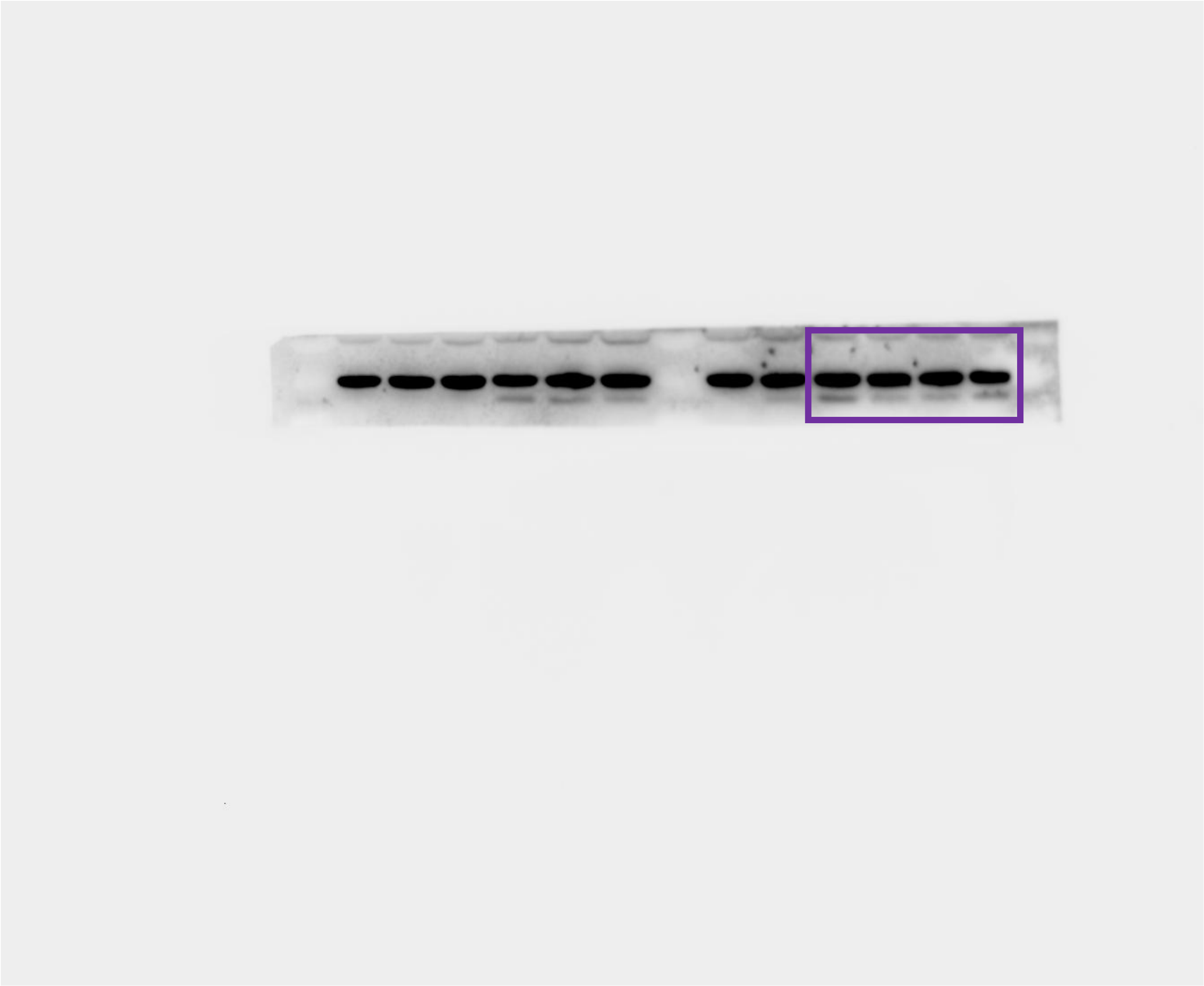

Figure 7B
Hep 3B   ATF4

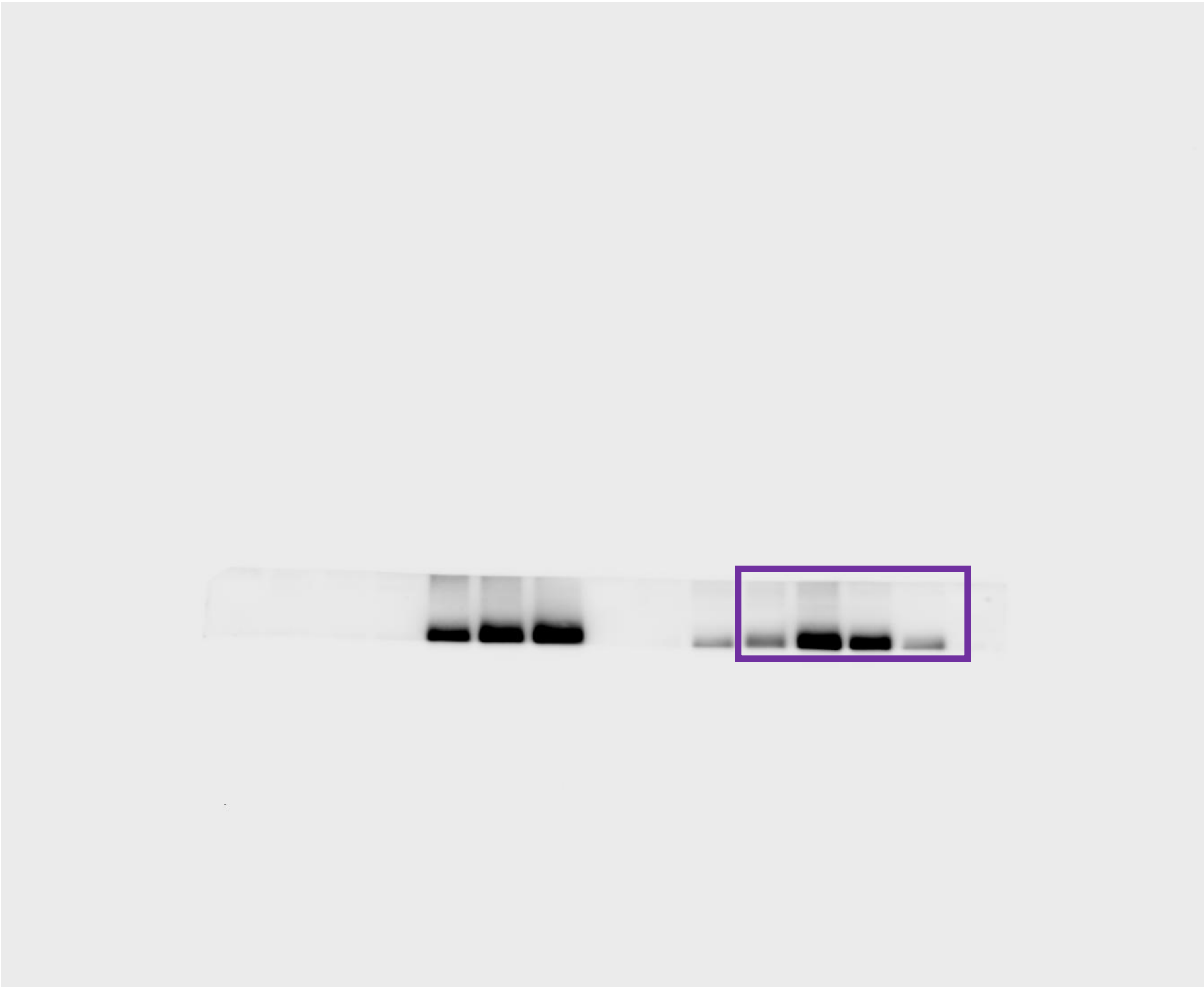

Figure 7B

Hep 3B   p-eIF2α

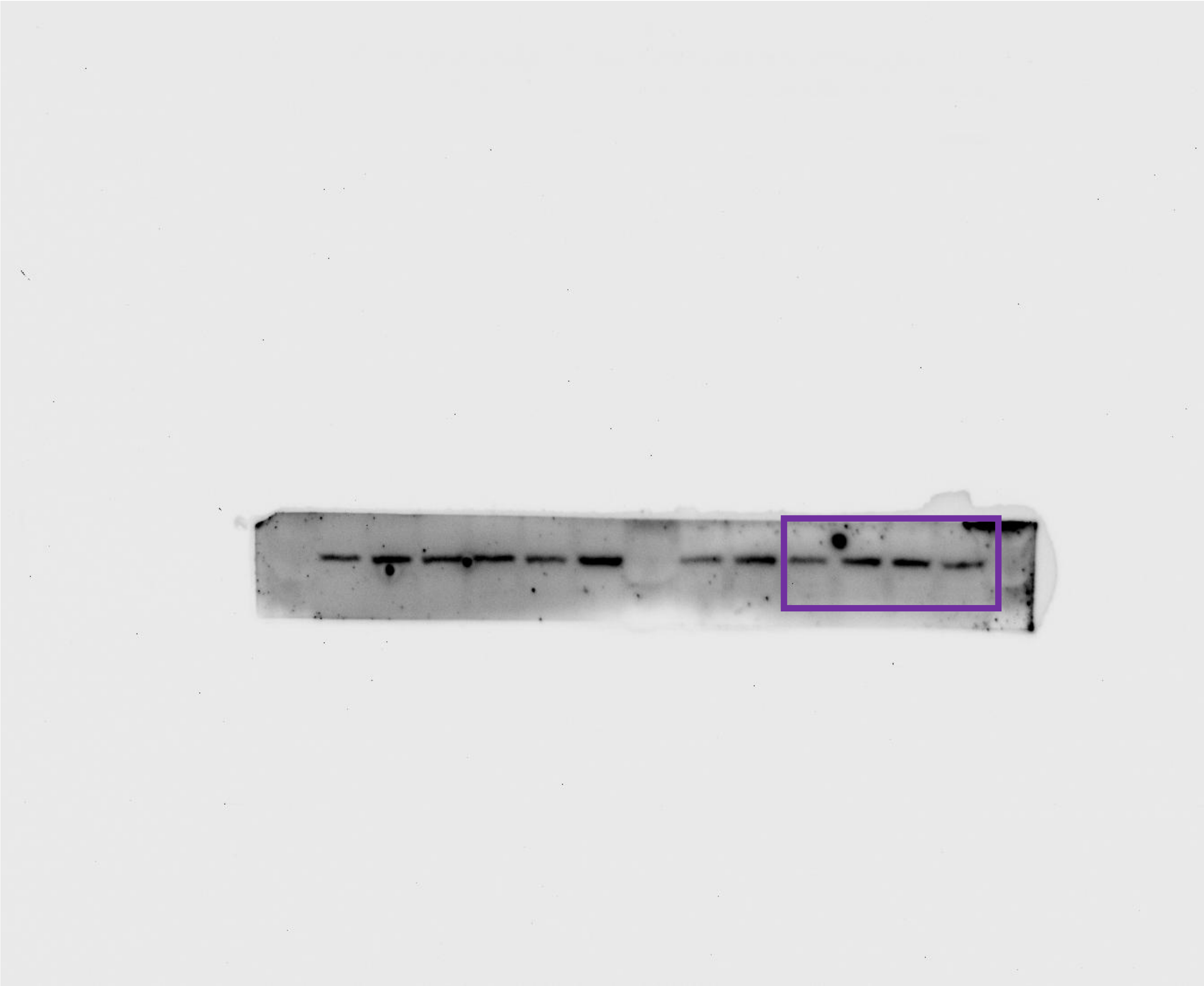

Figure 7B
Hep 3B eIF2α

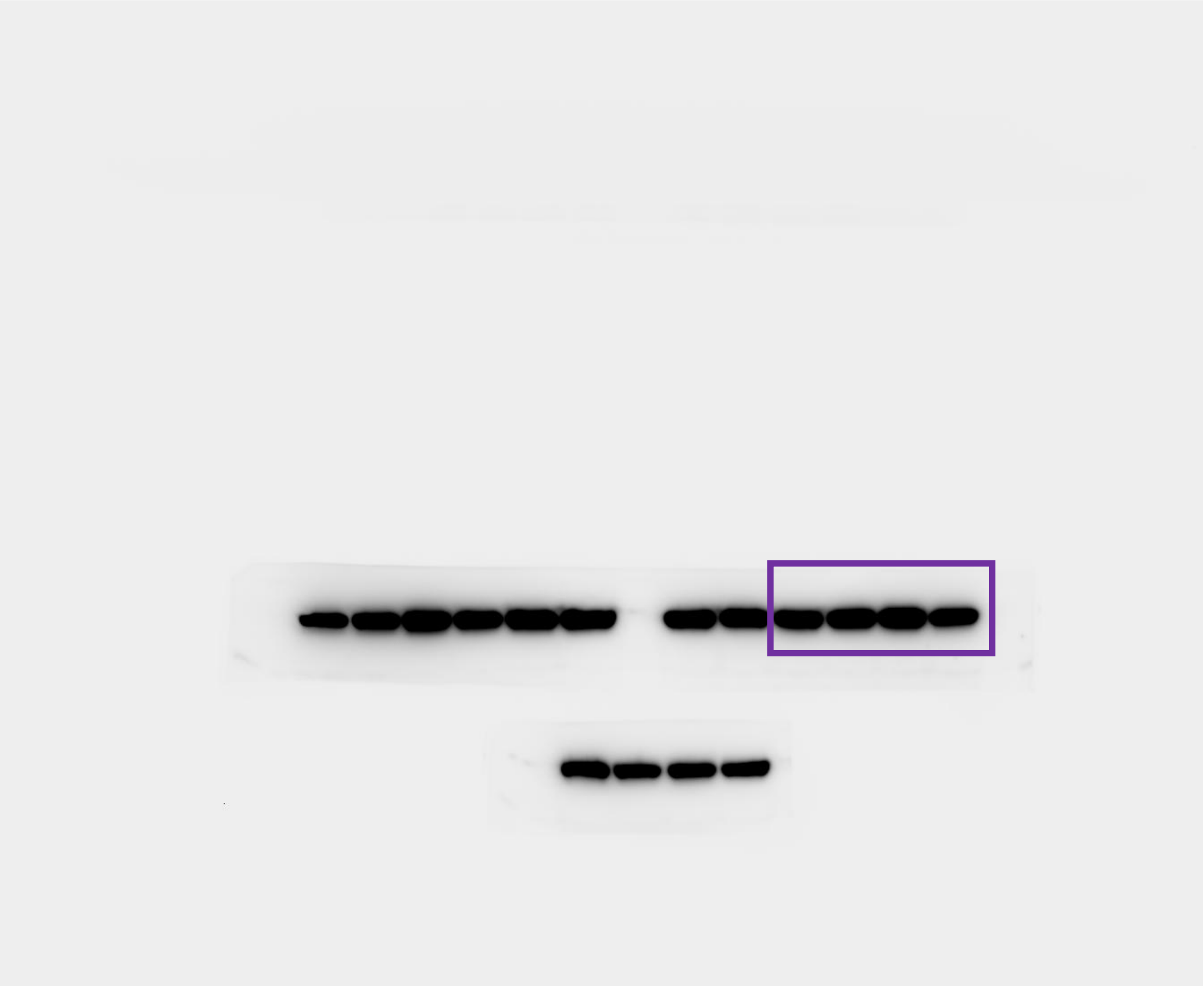

Figure 7B
Hep 3B GAPDH

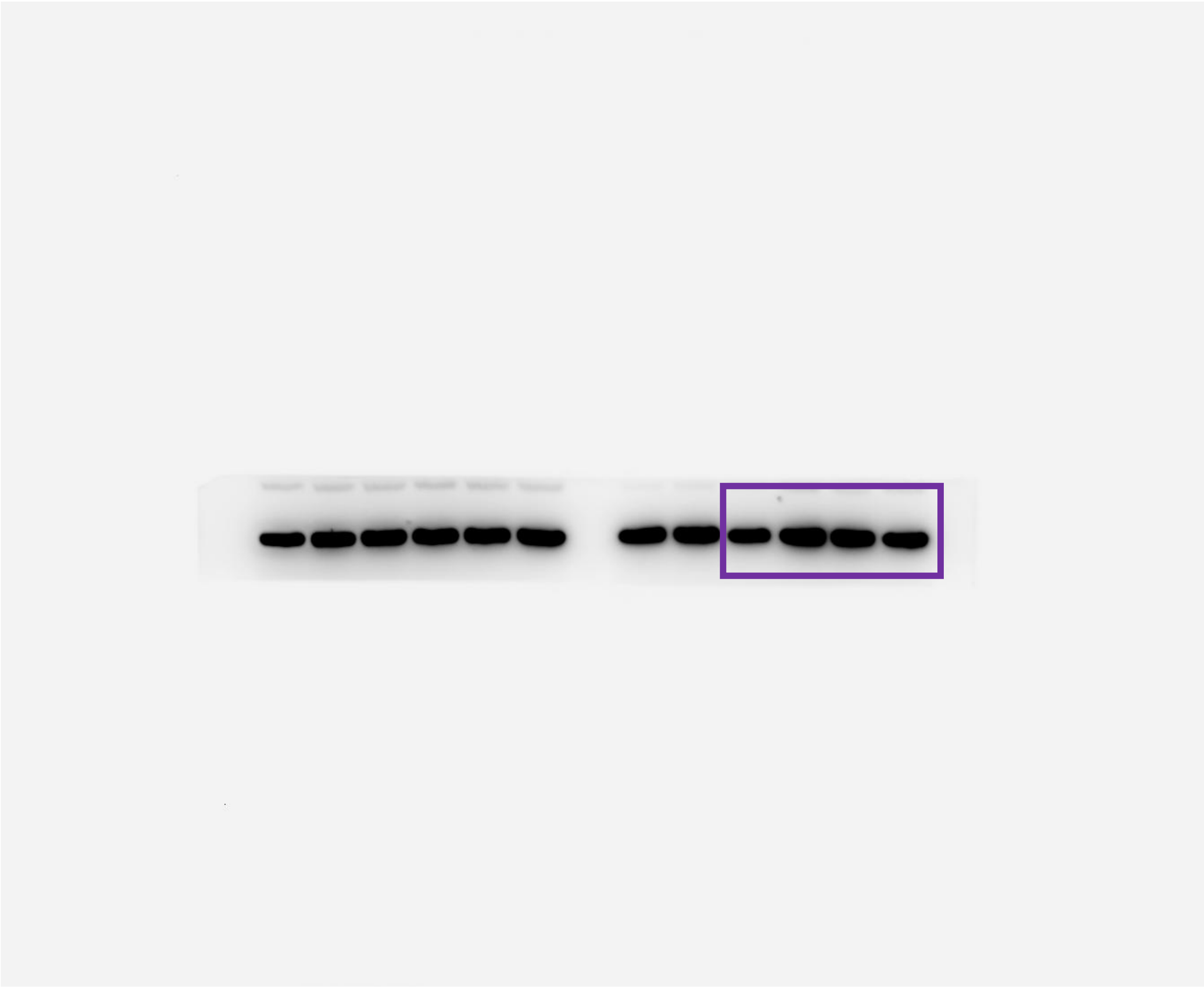

Figure 7C
p62

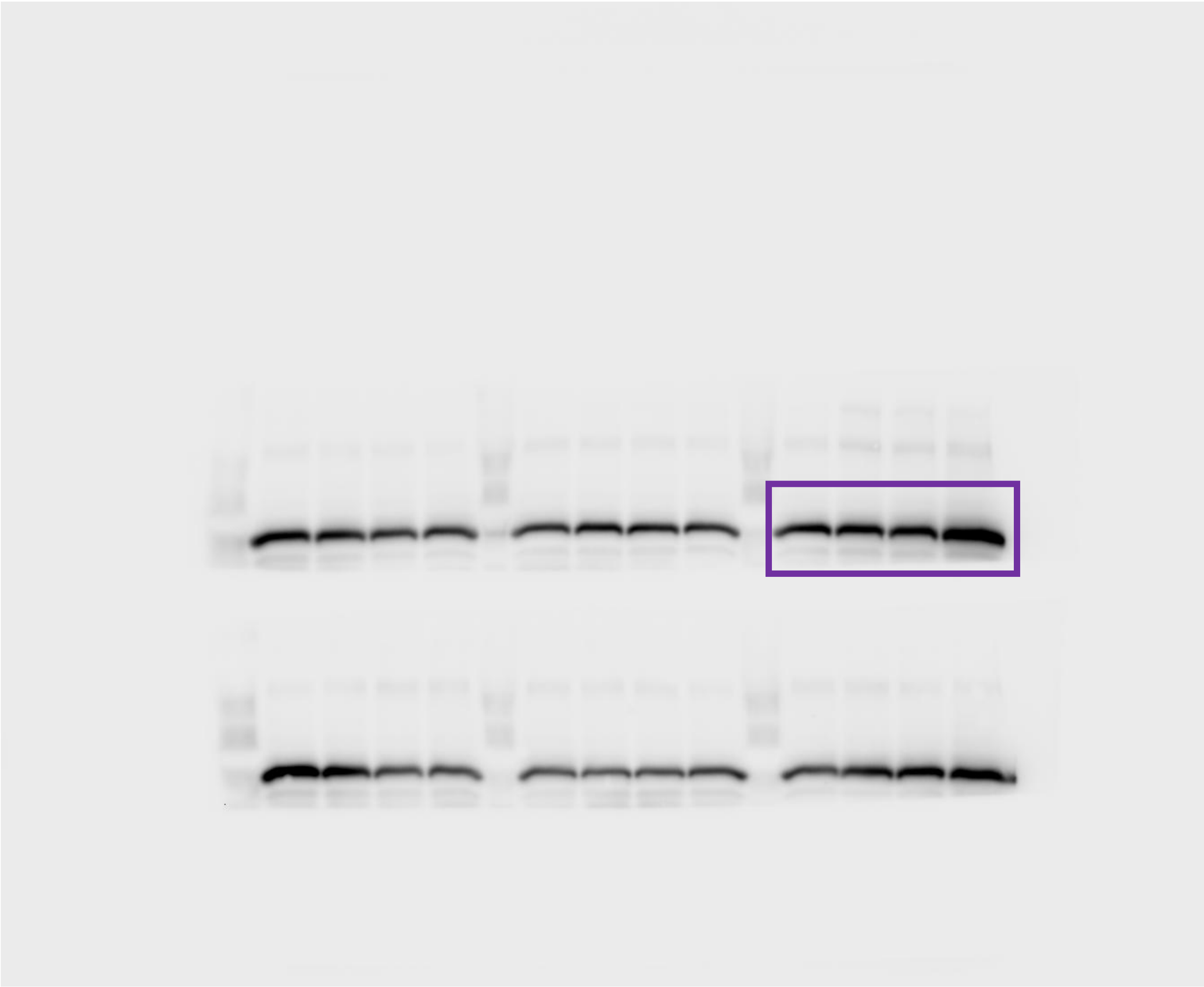

Figure 7C
LC3

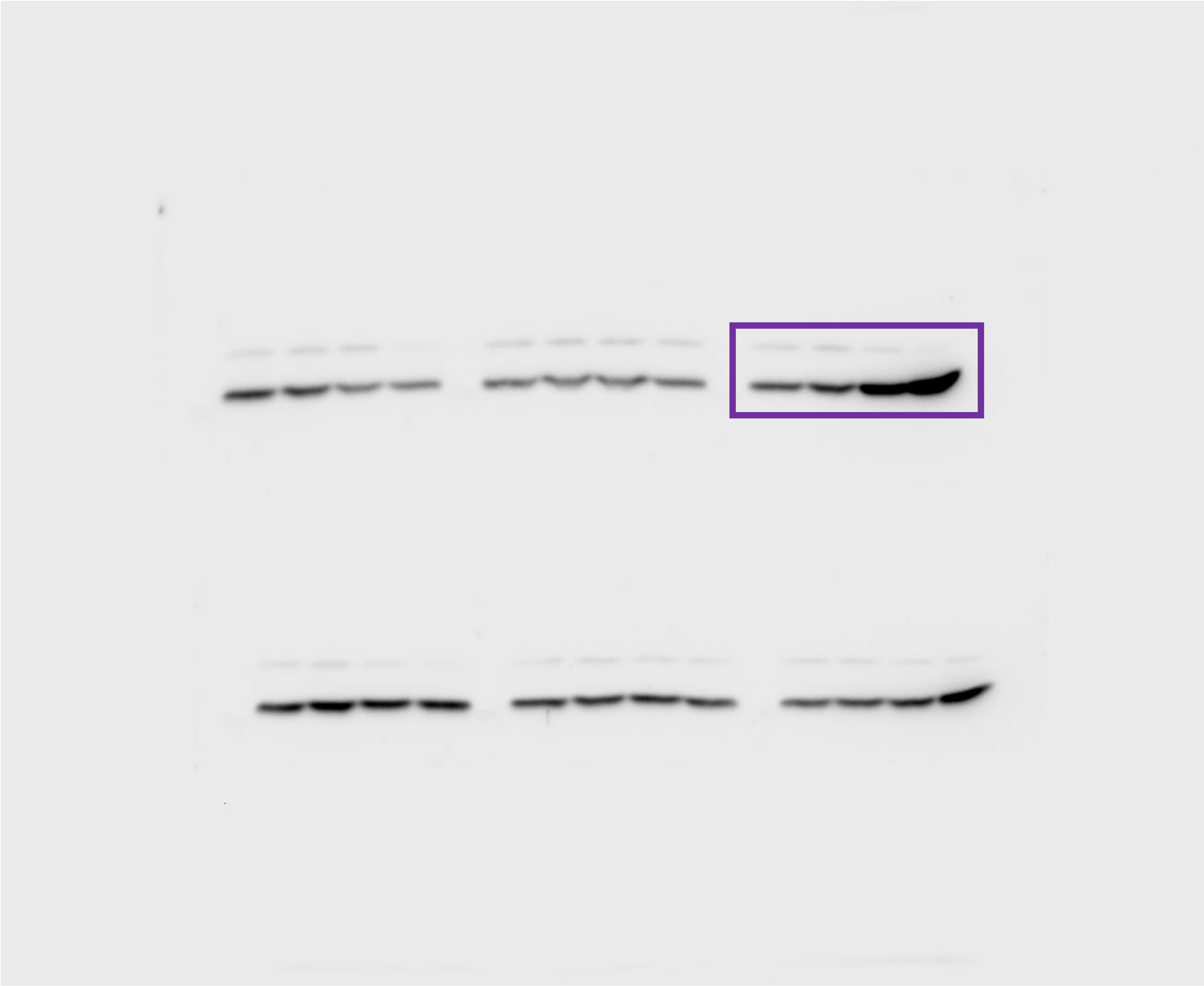

Figure 7C
GAPDH

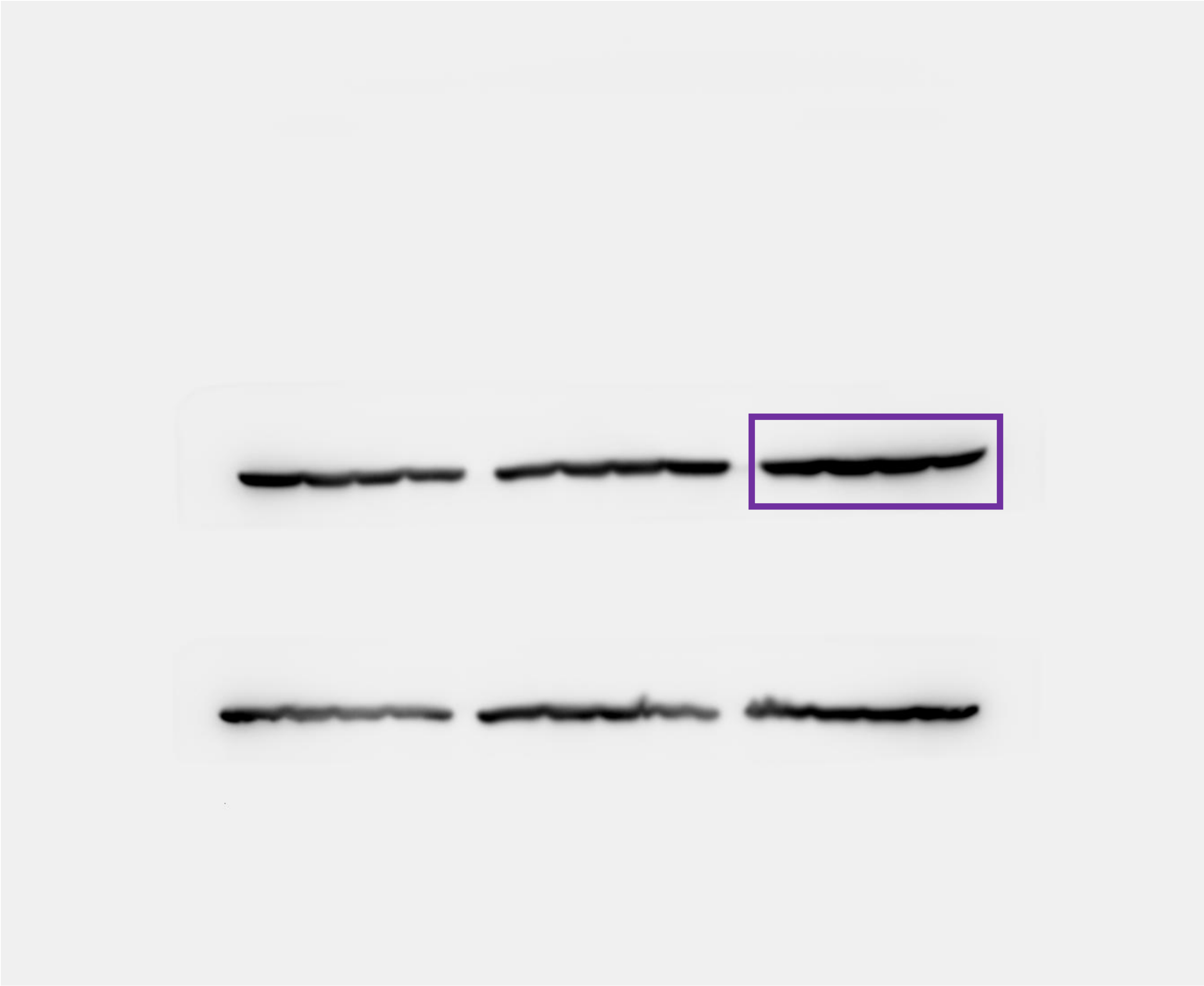

Figure 7C
ATG7

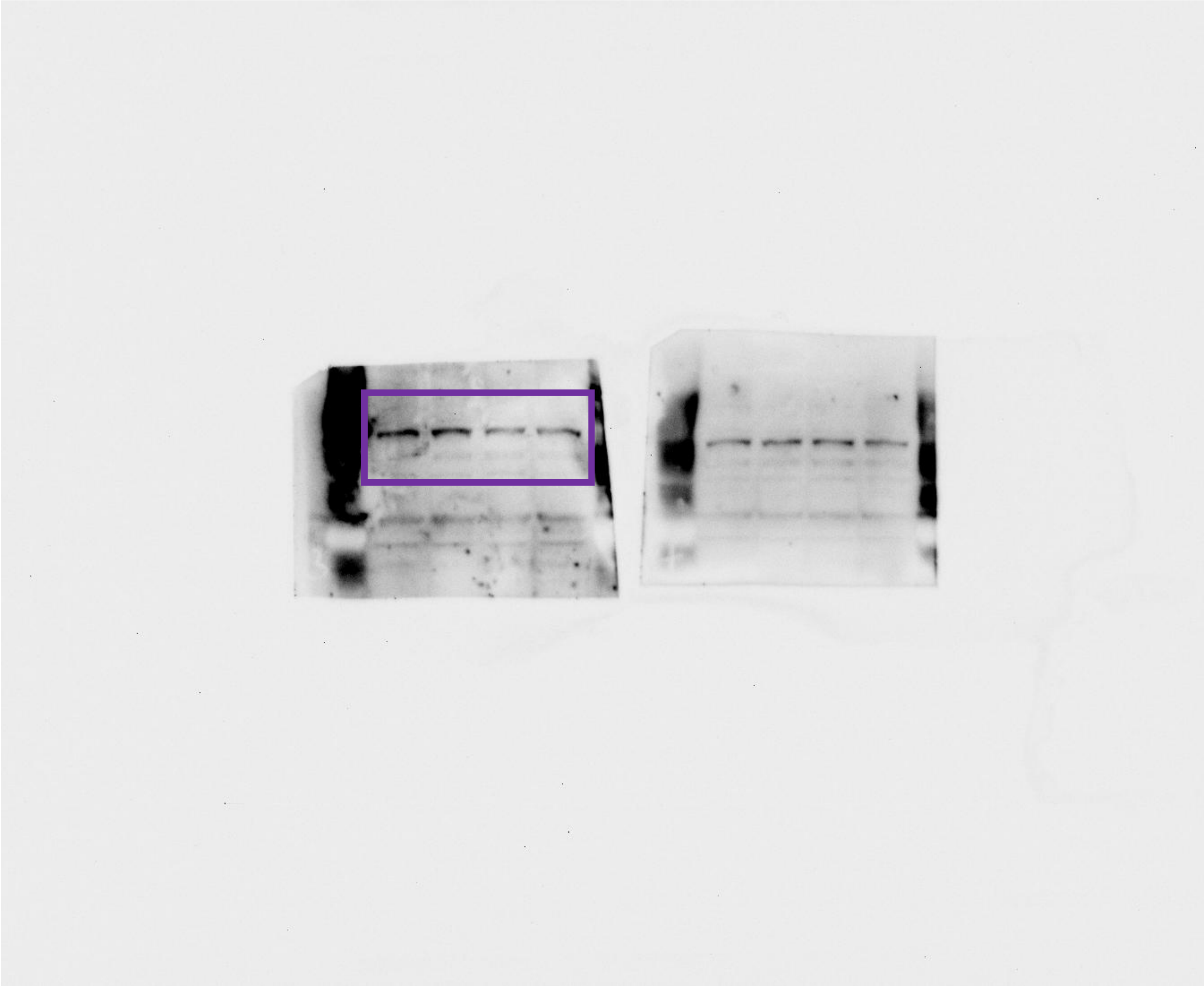

Figure 7C
BECLIN1

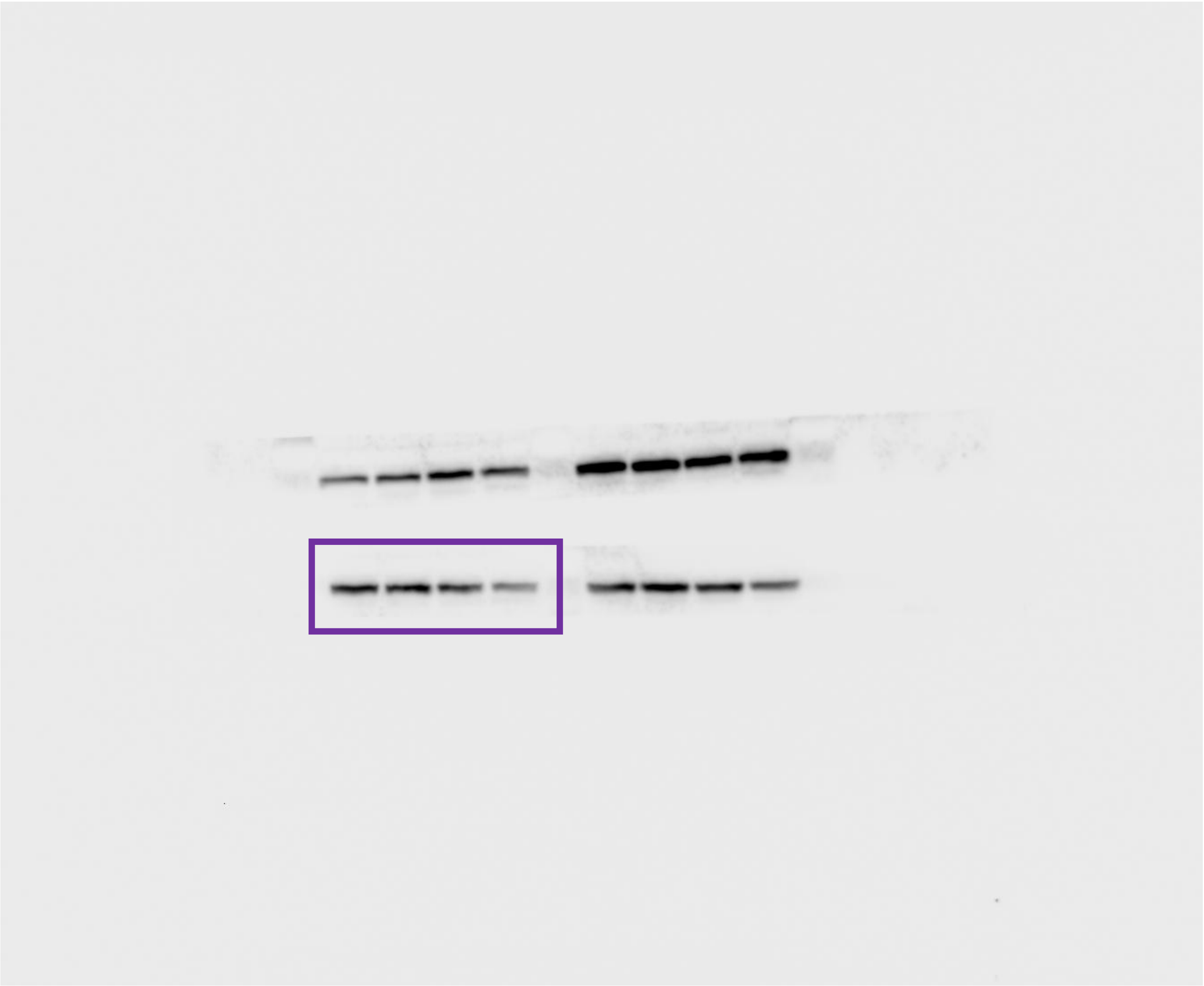

Figure 7C
GAPDH

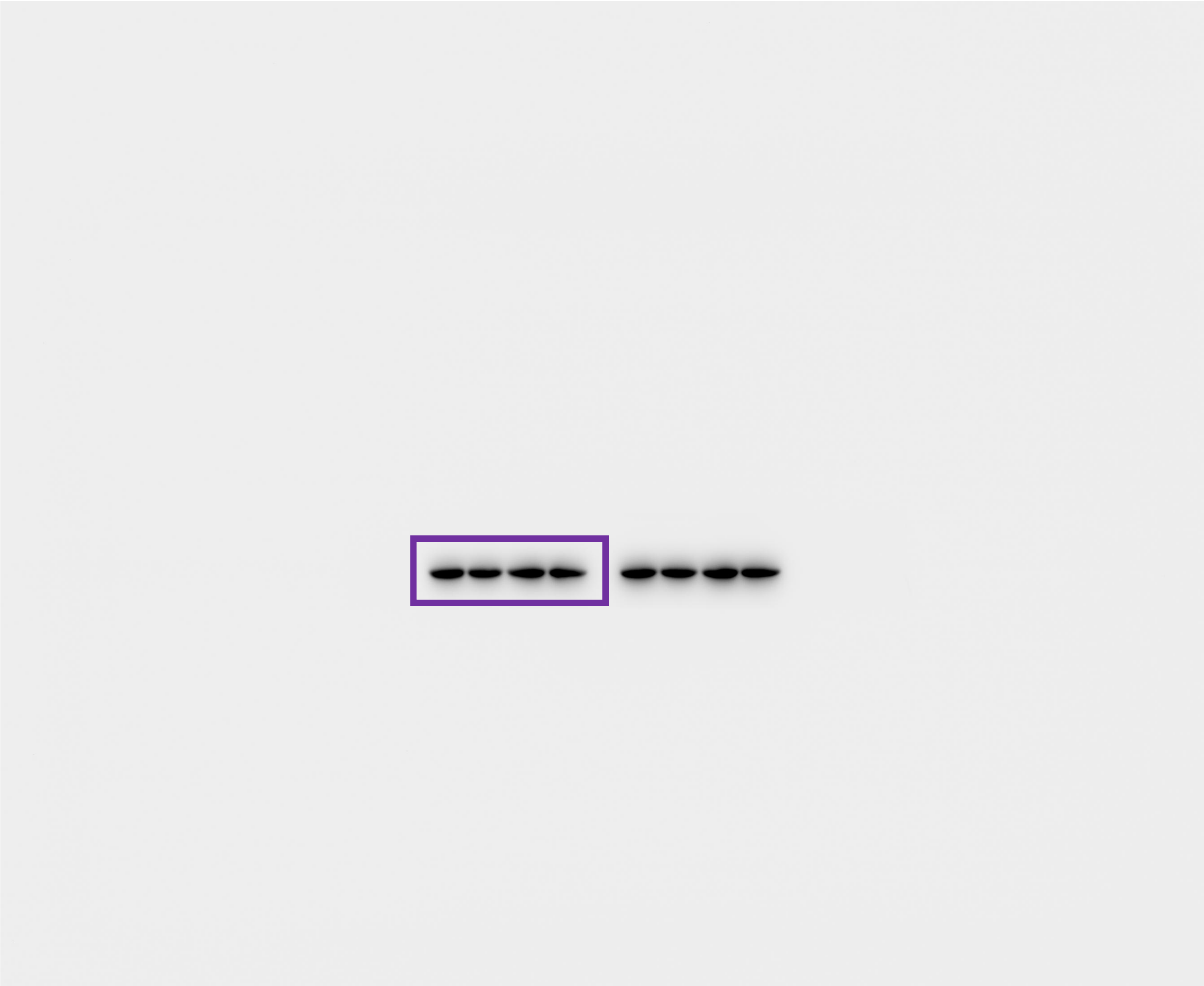

Figure 8C
MHCC97-H FLNA

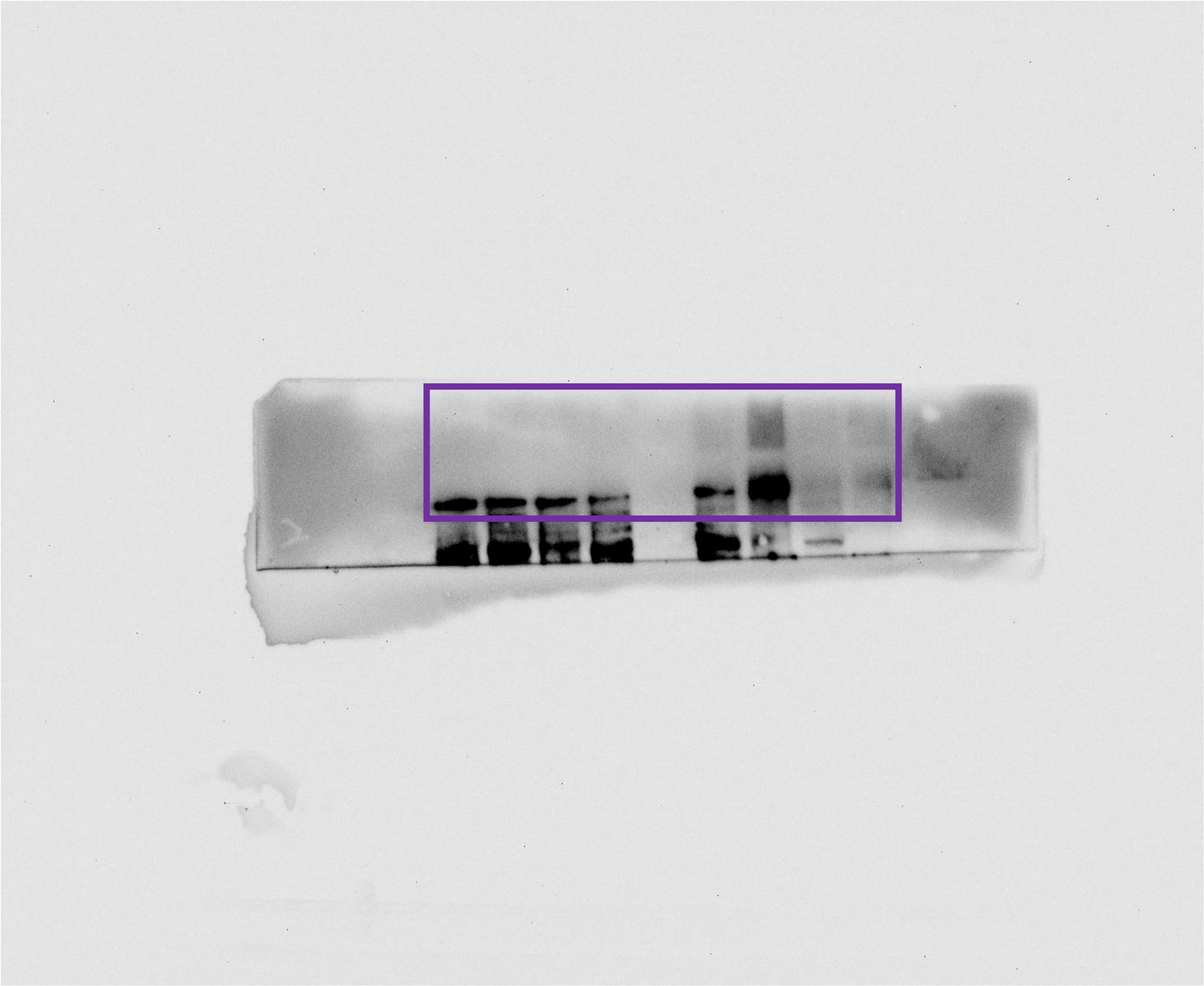

Figure 8C
MHCC97-H DREBRIN

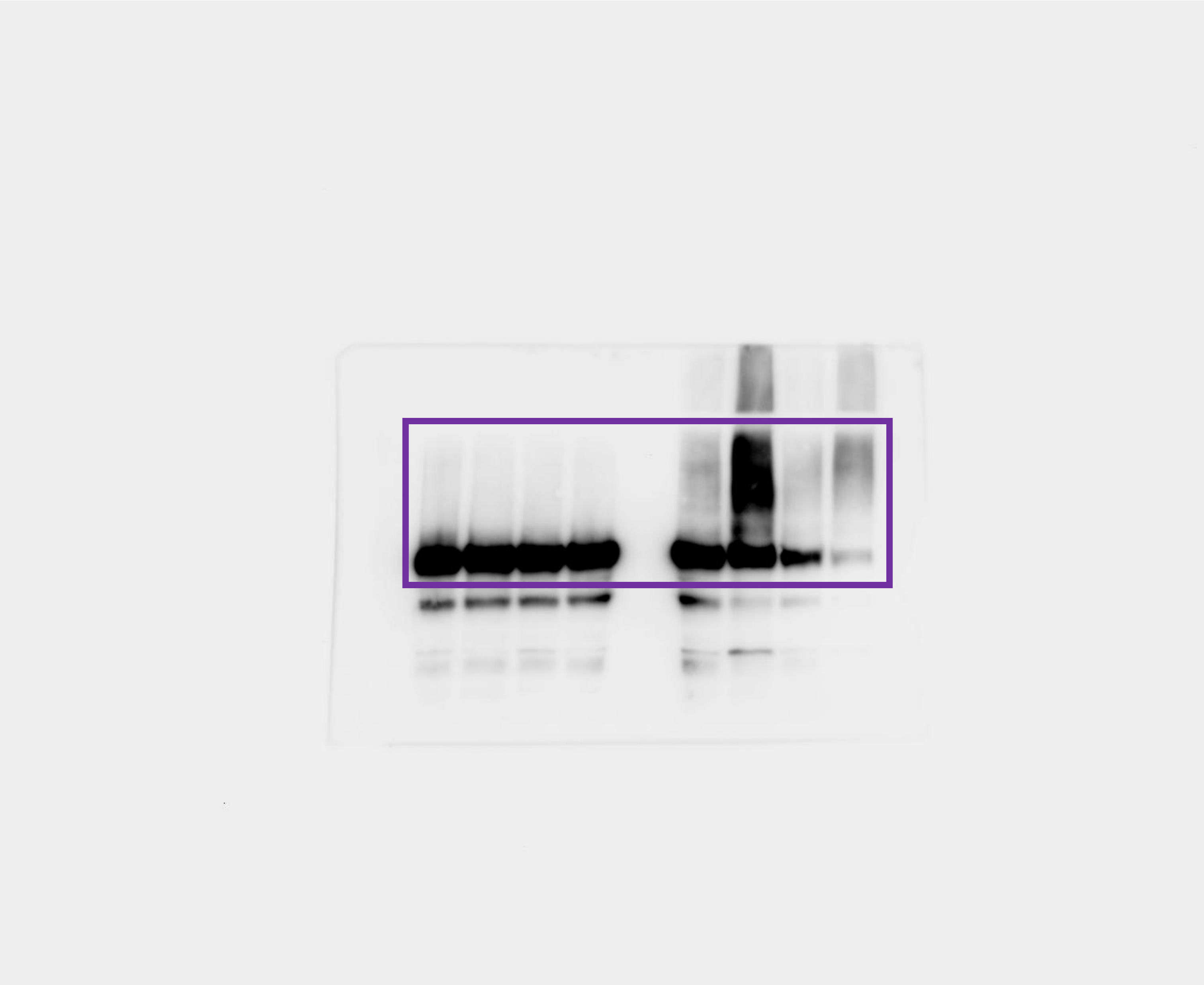

Figure 8C
MHCC97-H GAPDH

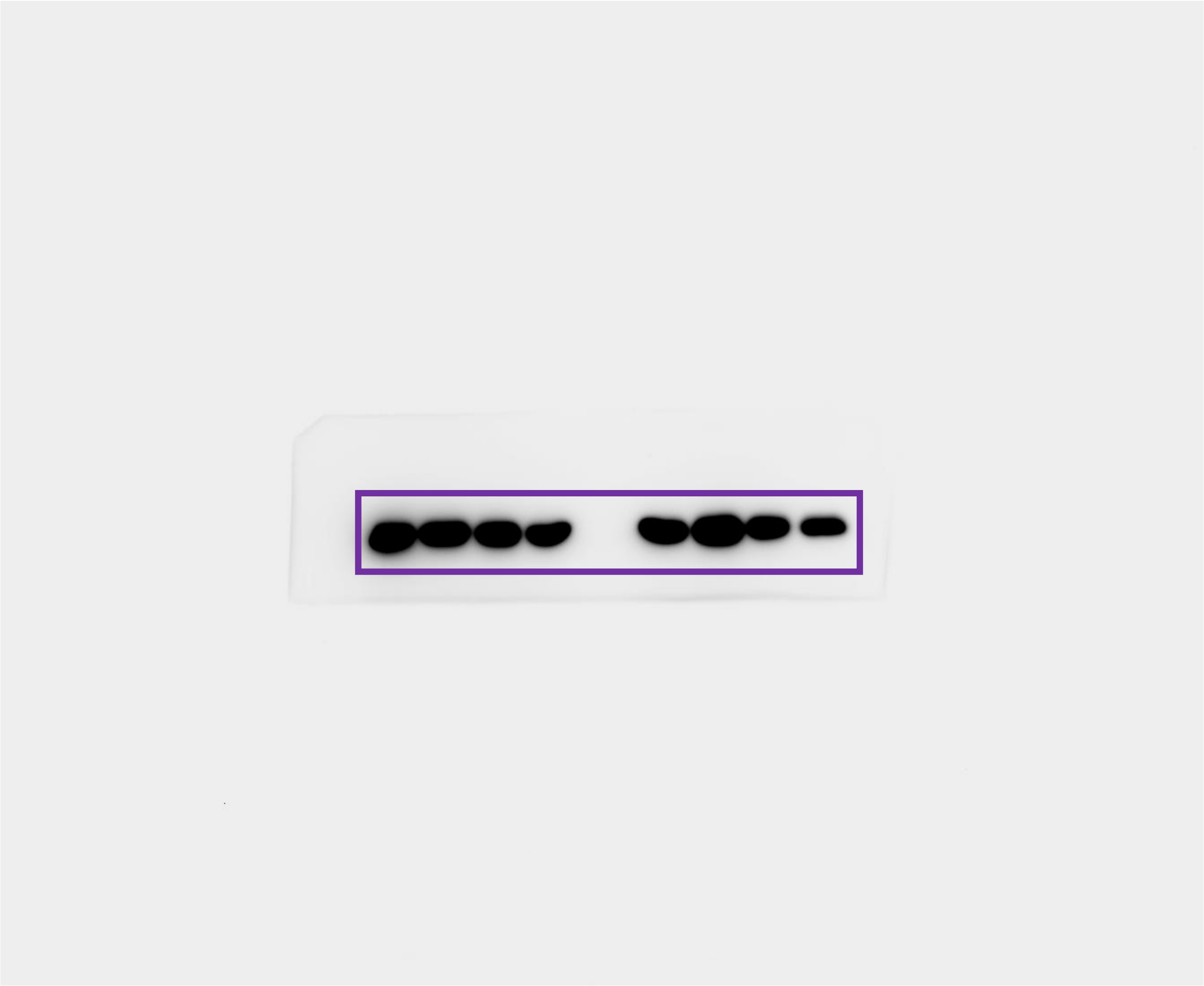

Figure 8D
Hep 3B FLNA

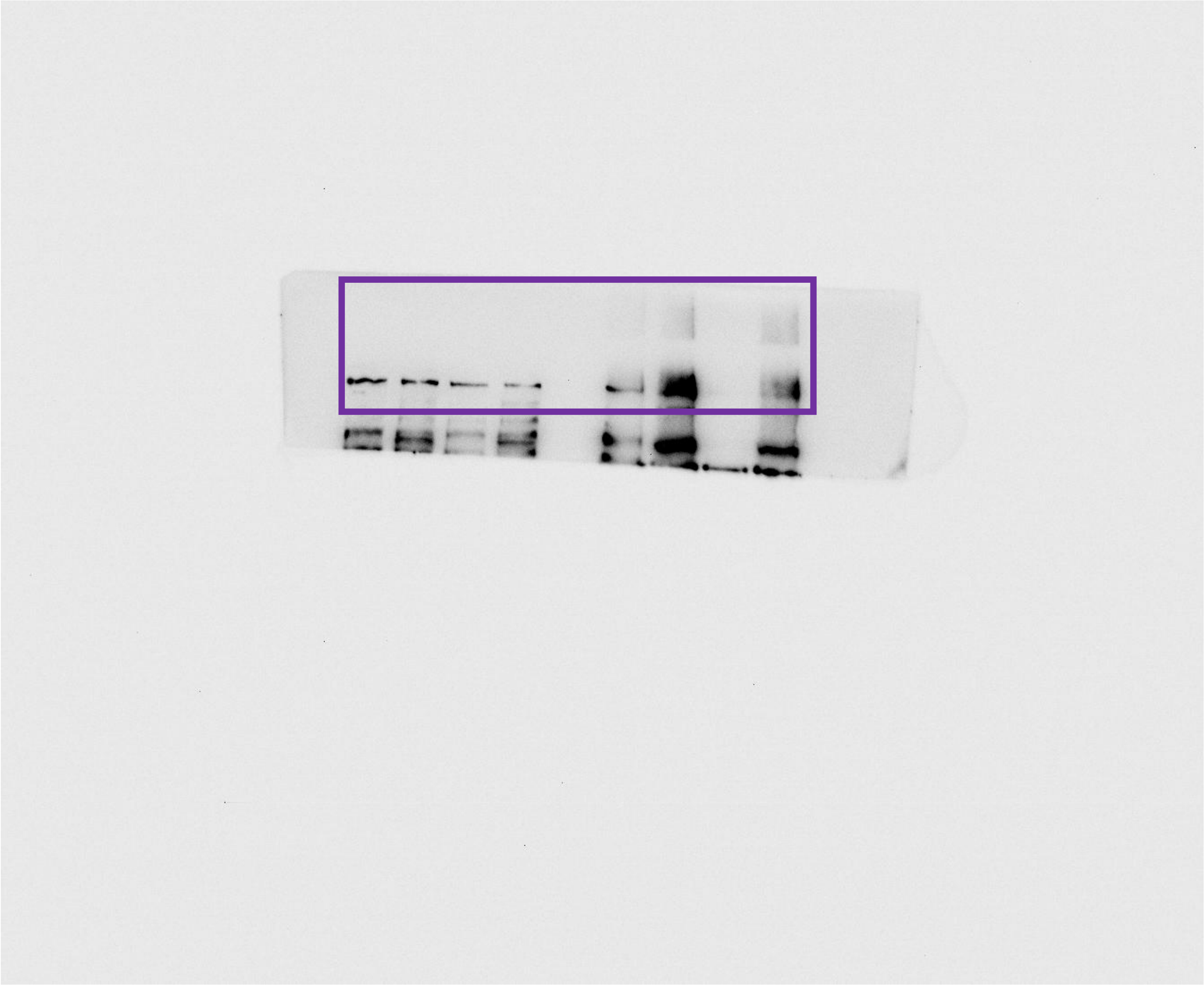

Figure 8D
Hep 3B DREBRIN

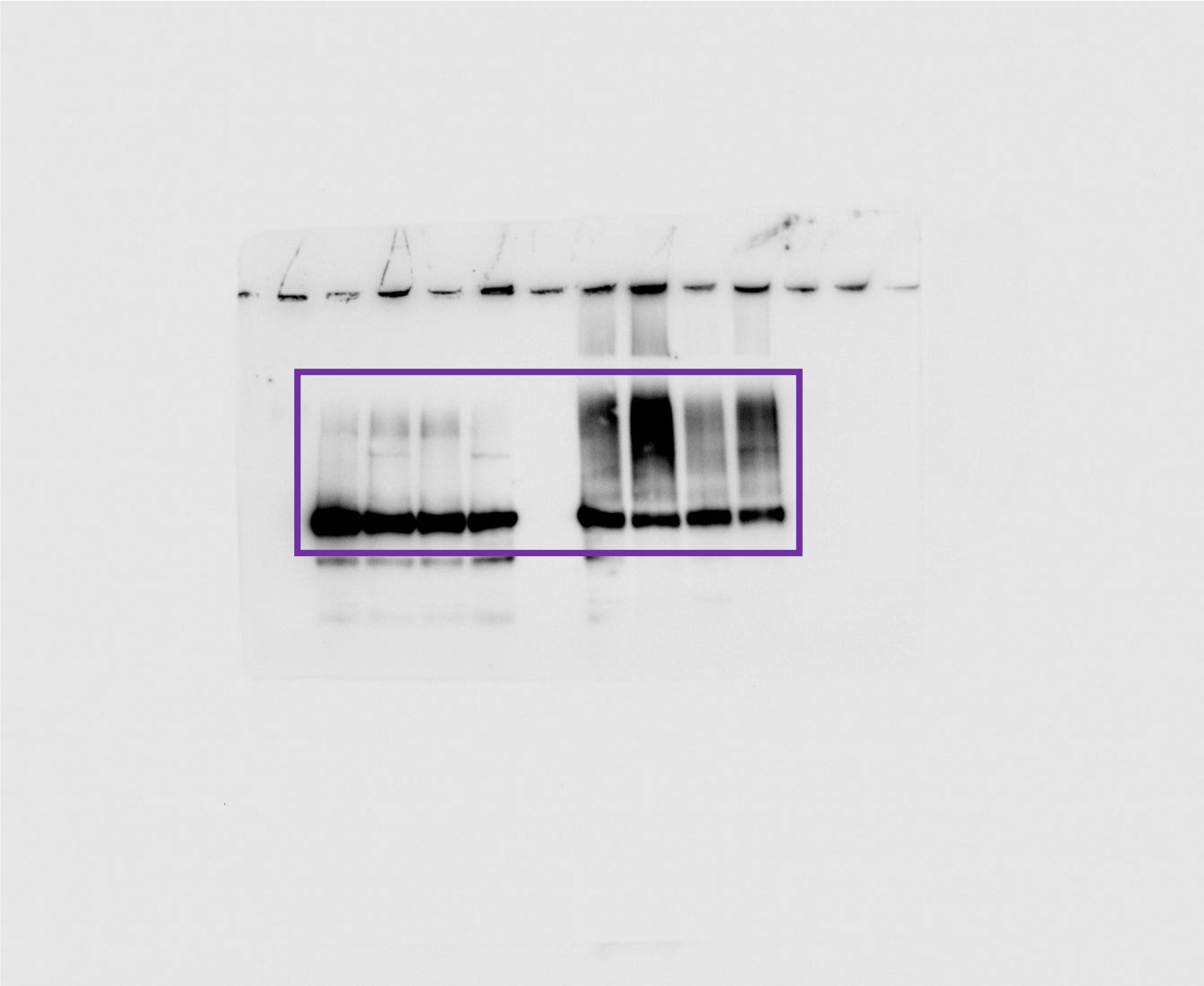

Figure 8D
Hep 3B GAPDH

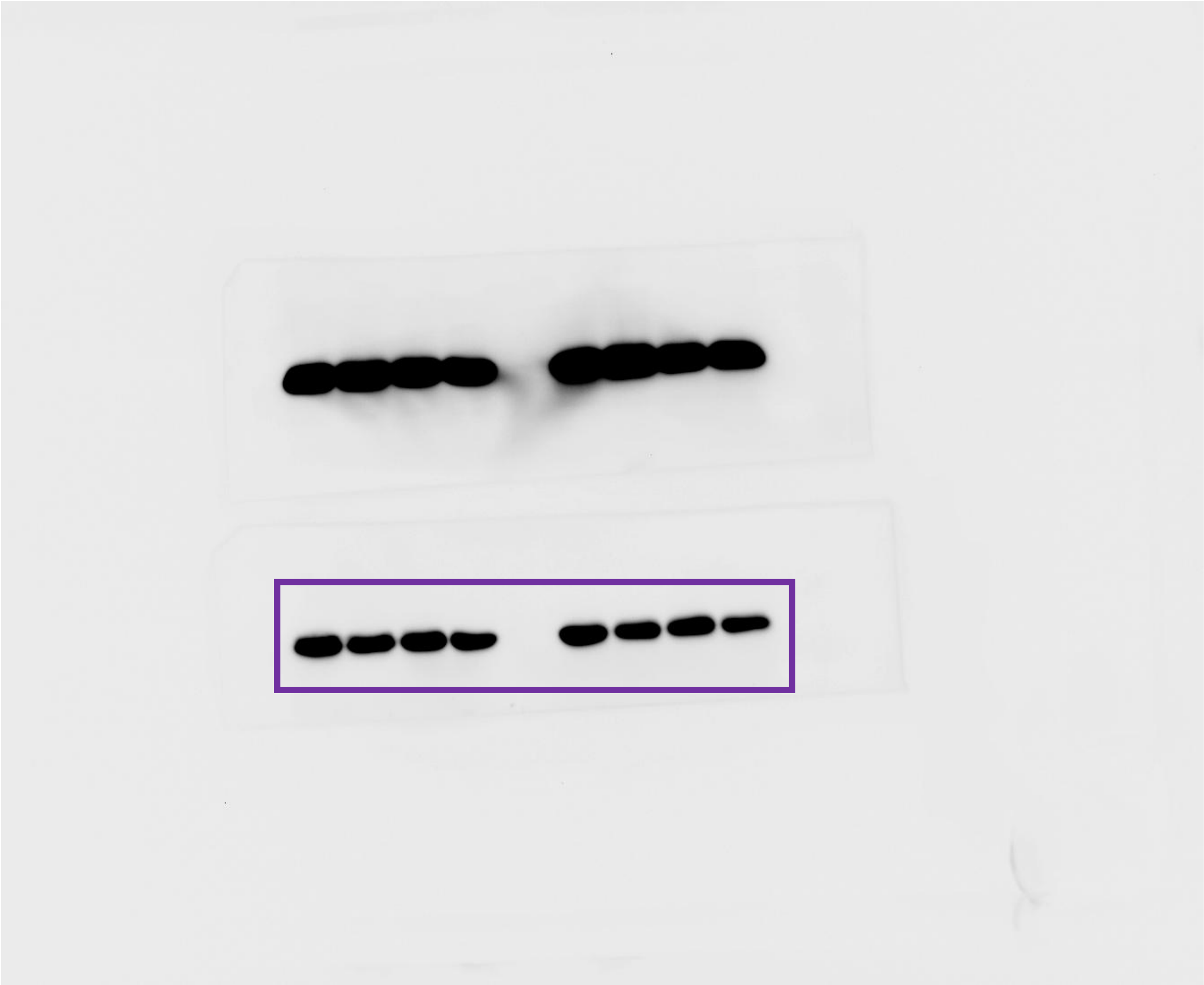

Figure S1A
ATG7

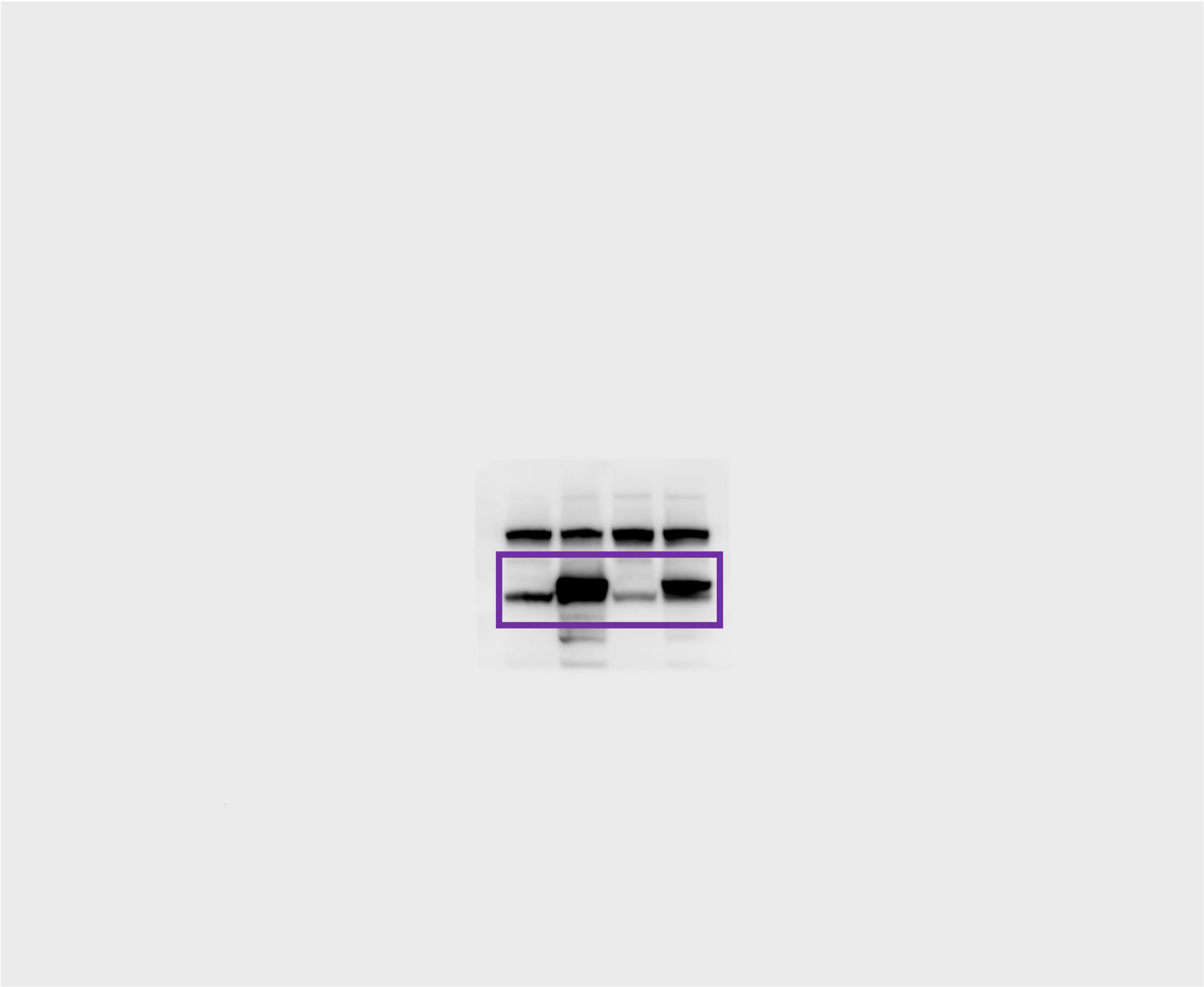

Figure S1A
p62

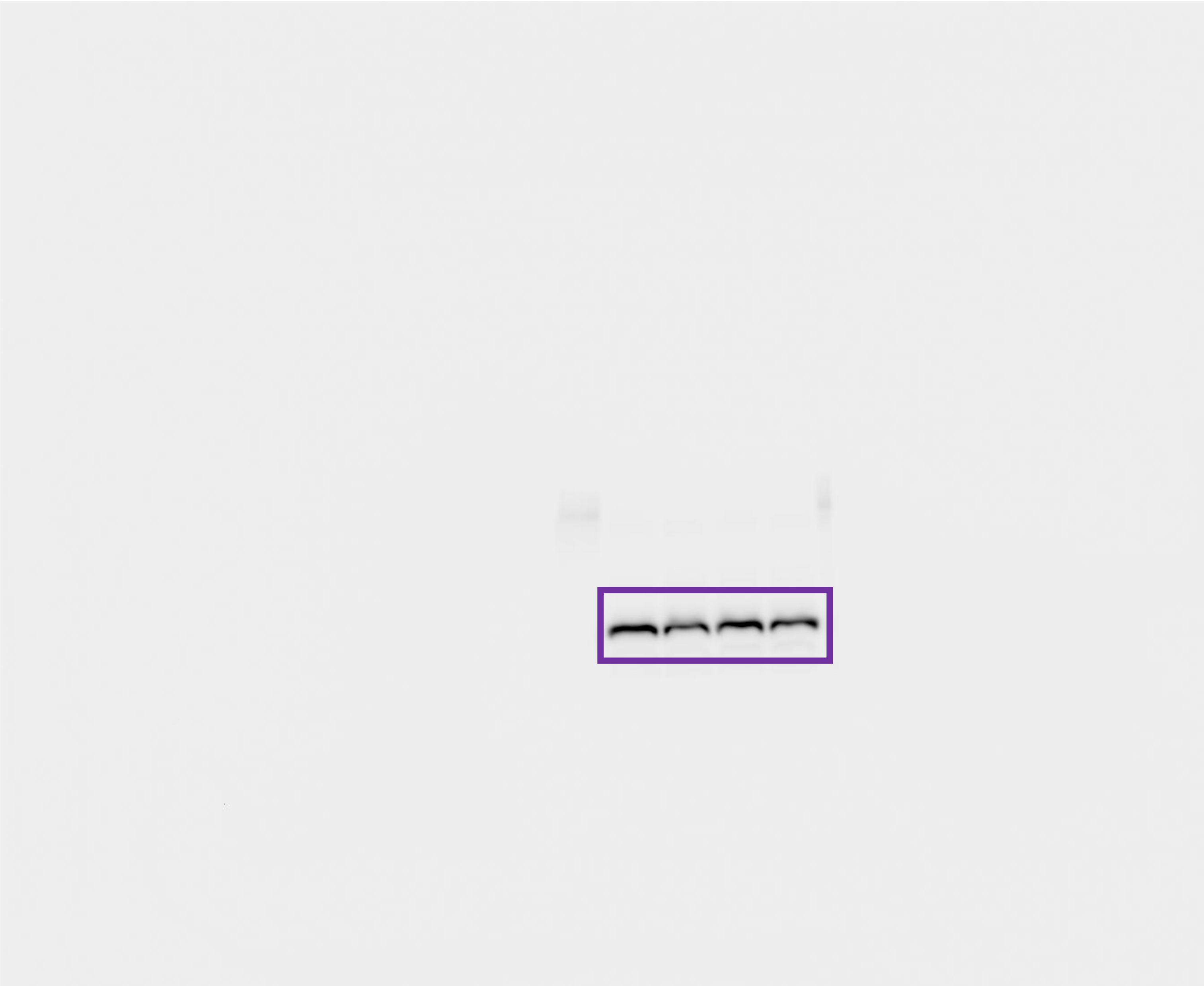

Figure S1A
BECLIN1

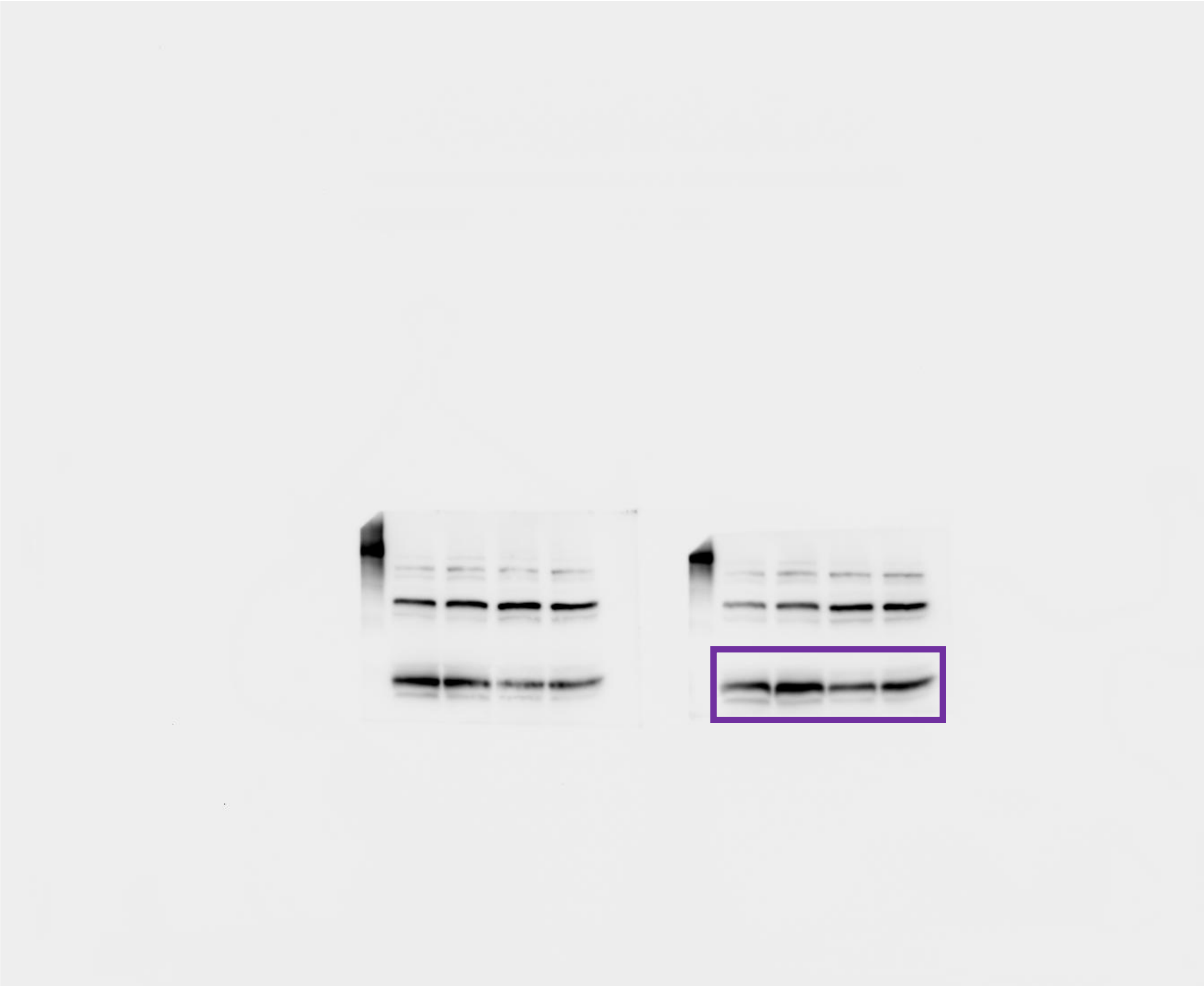

Figure S1A
LC3

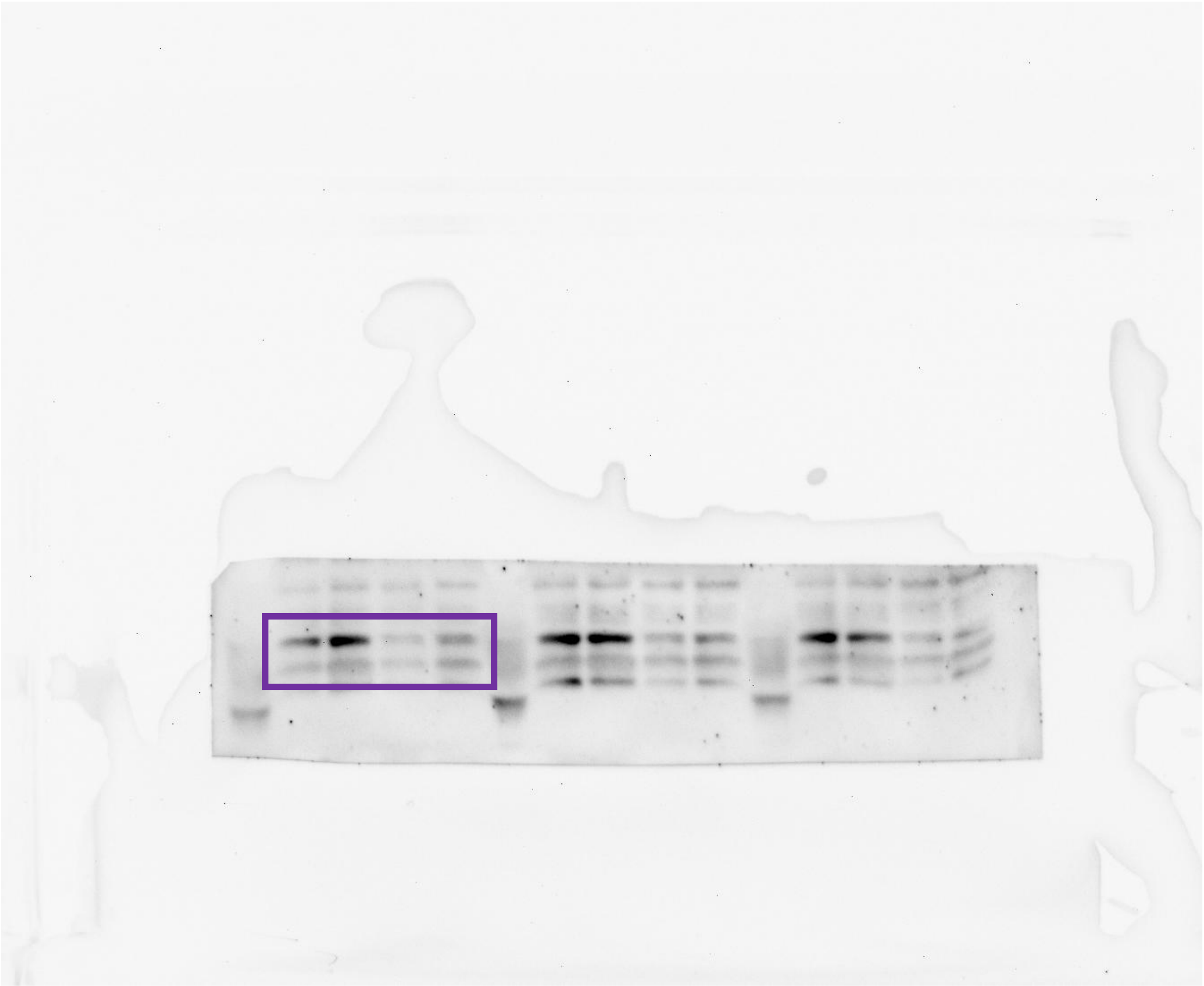

Figure S1A
GAPDH

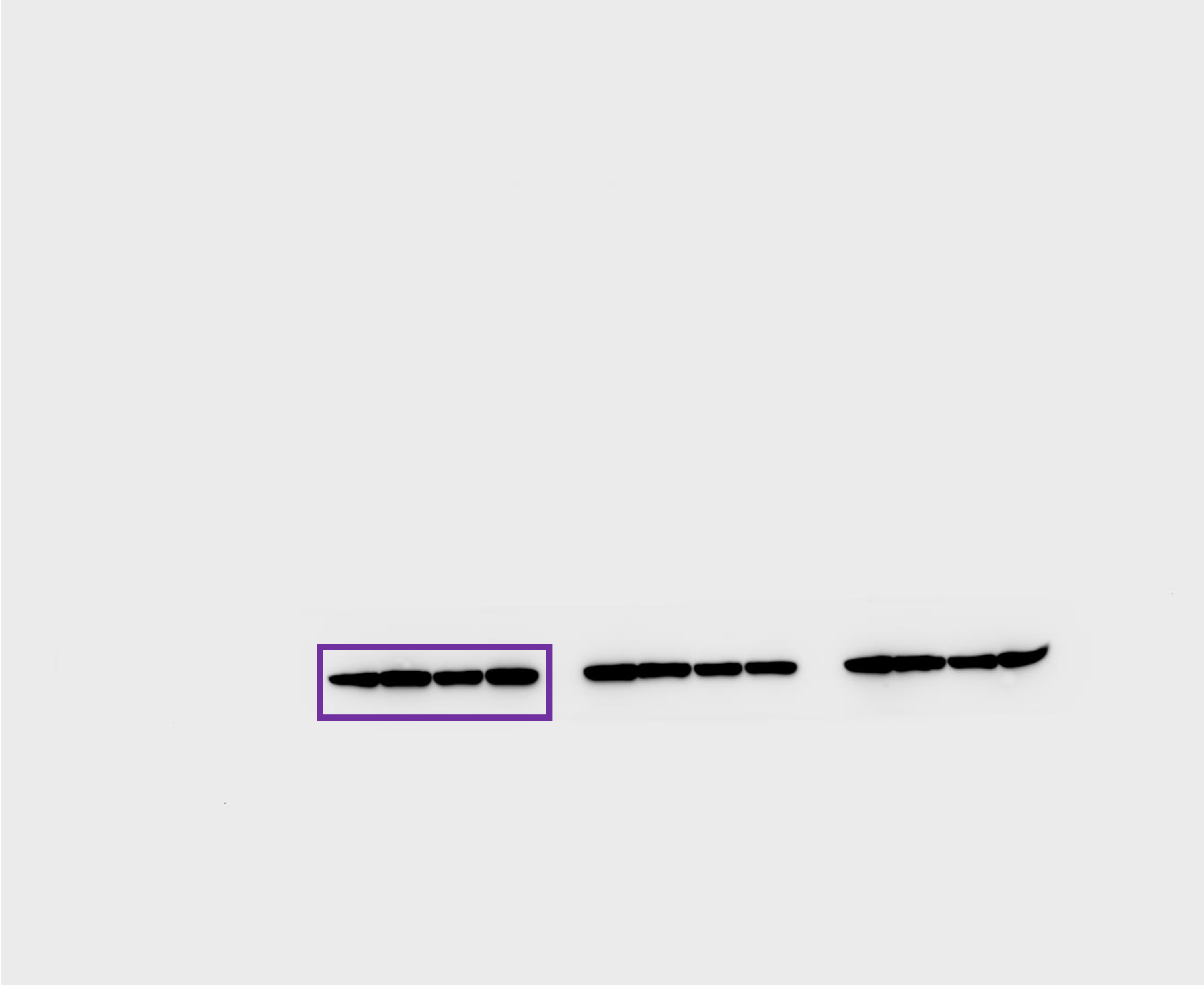

Figure S1C
MHCC97-H GAPDH

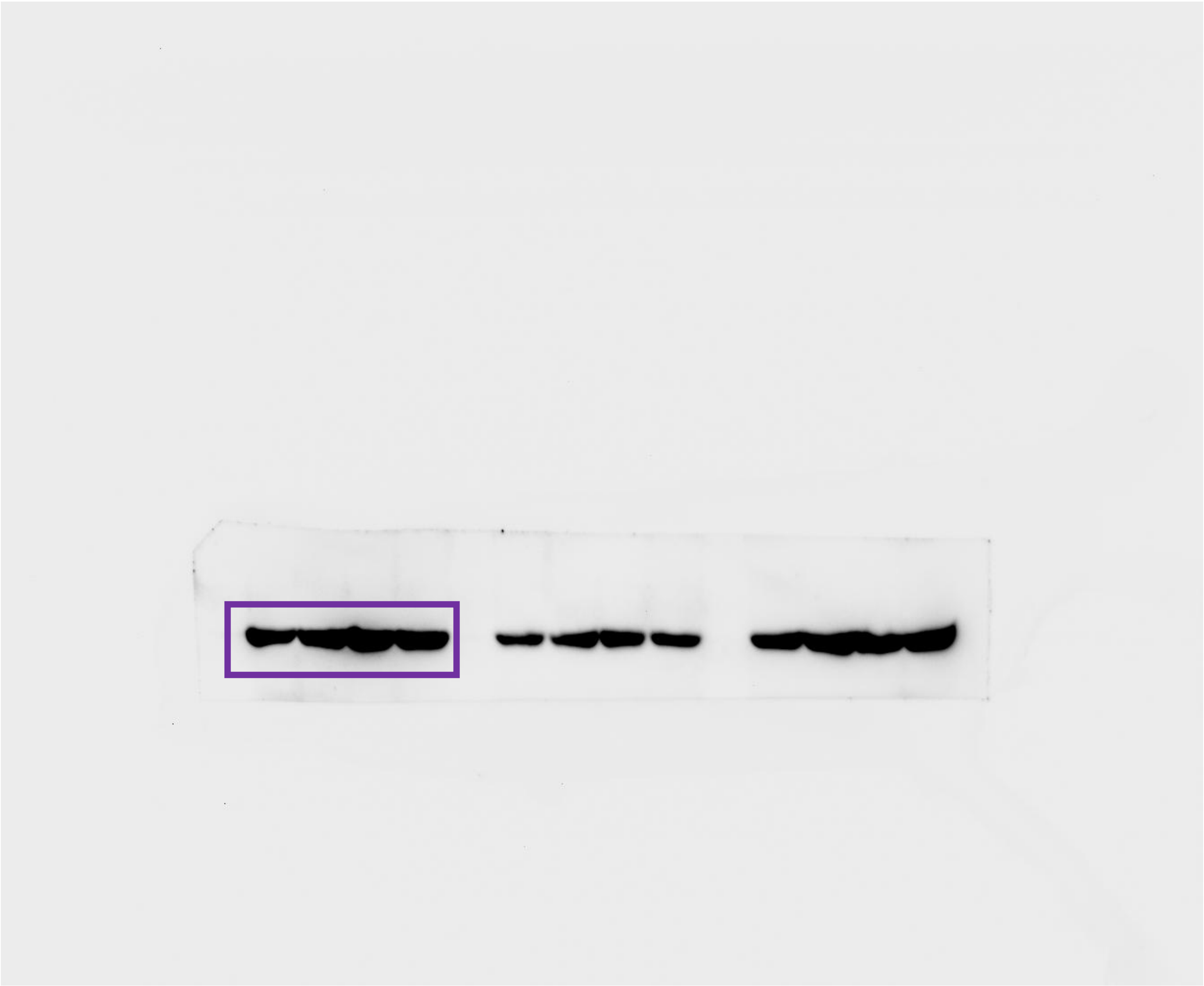

Figure S1C
Hep 3B GAPDH

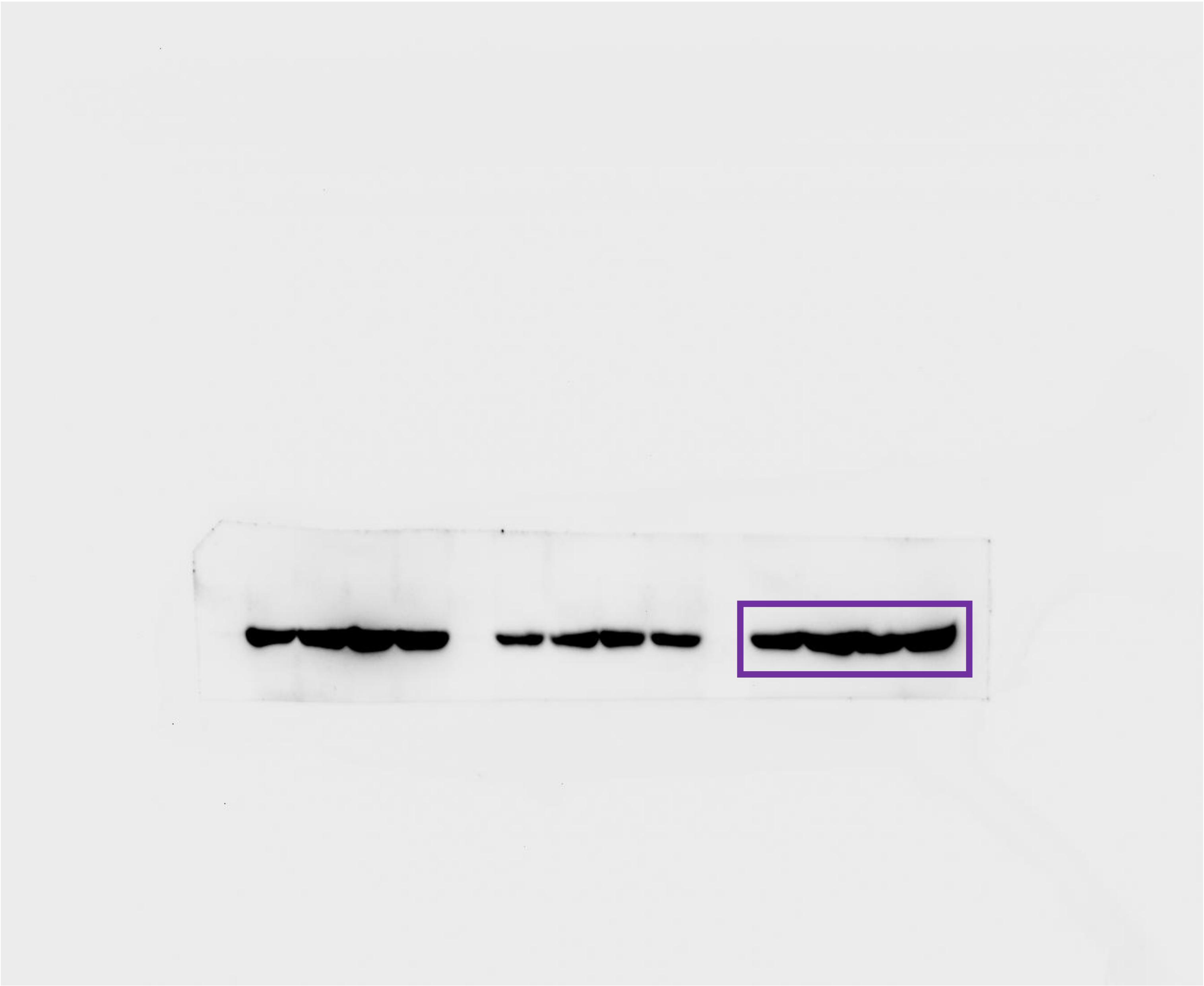

Figure S1C
MHCC97-H DREBRIN

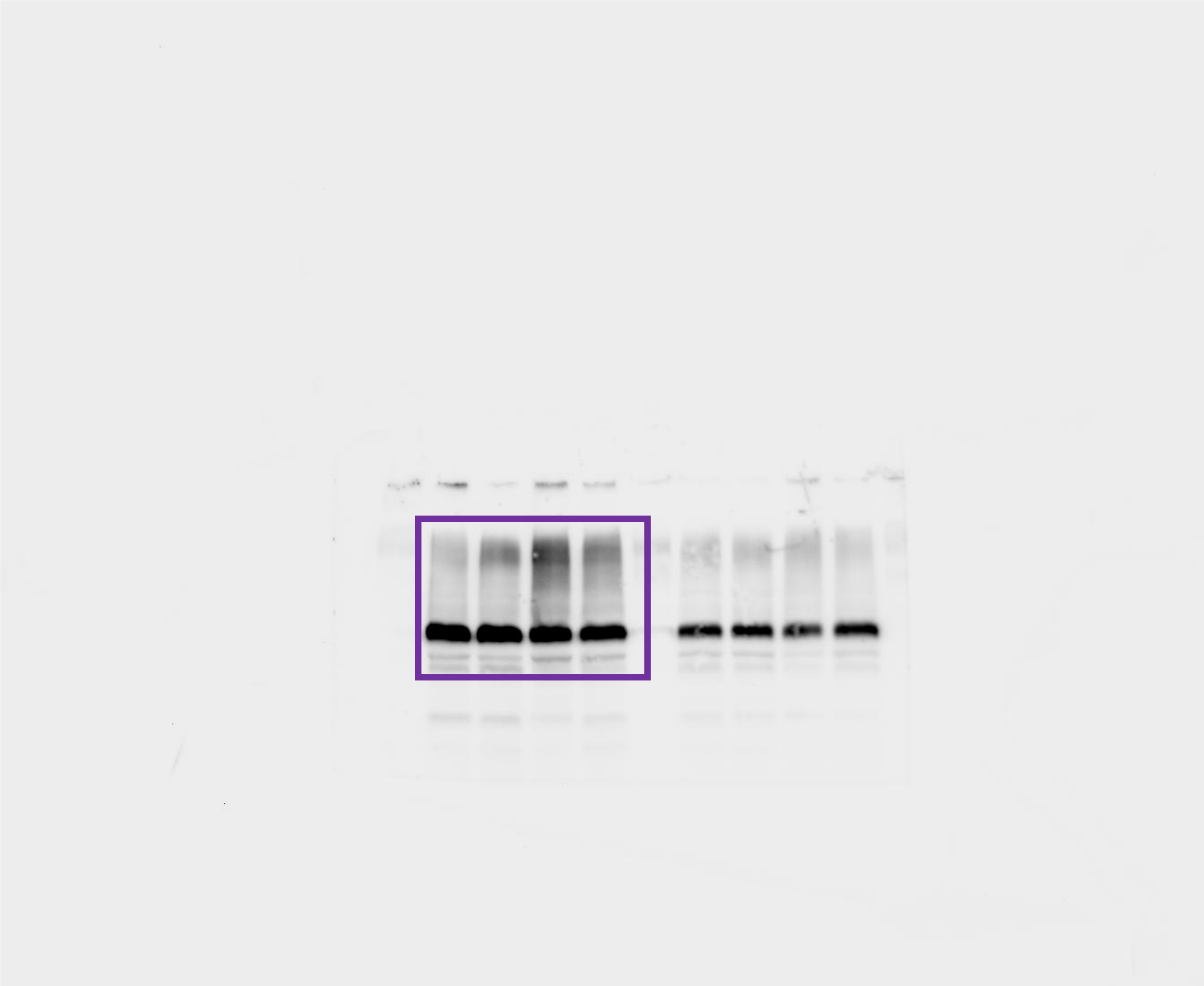

Figure S1C
Hep 3B DREBRIN

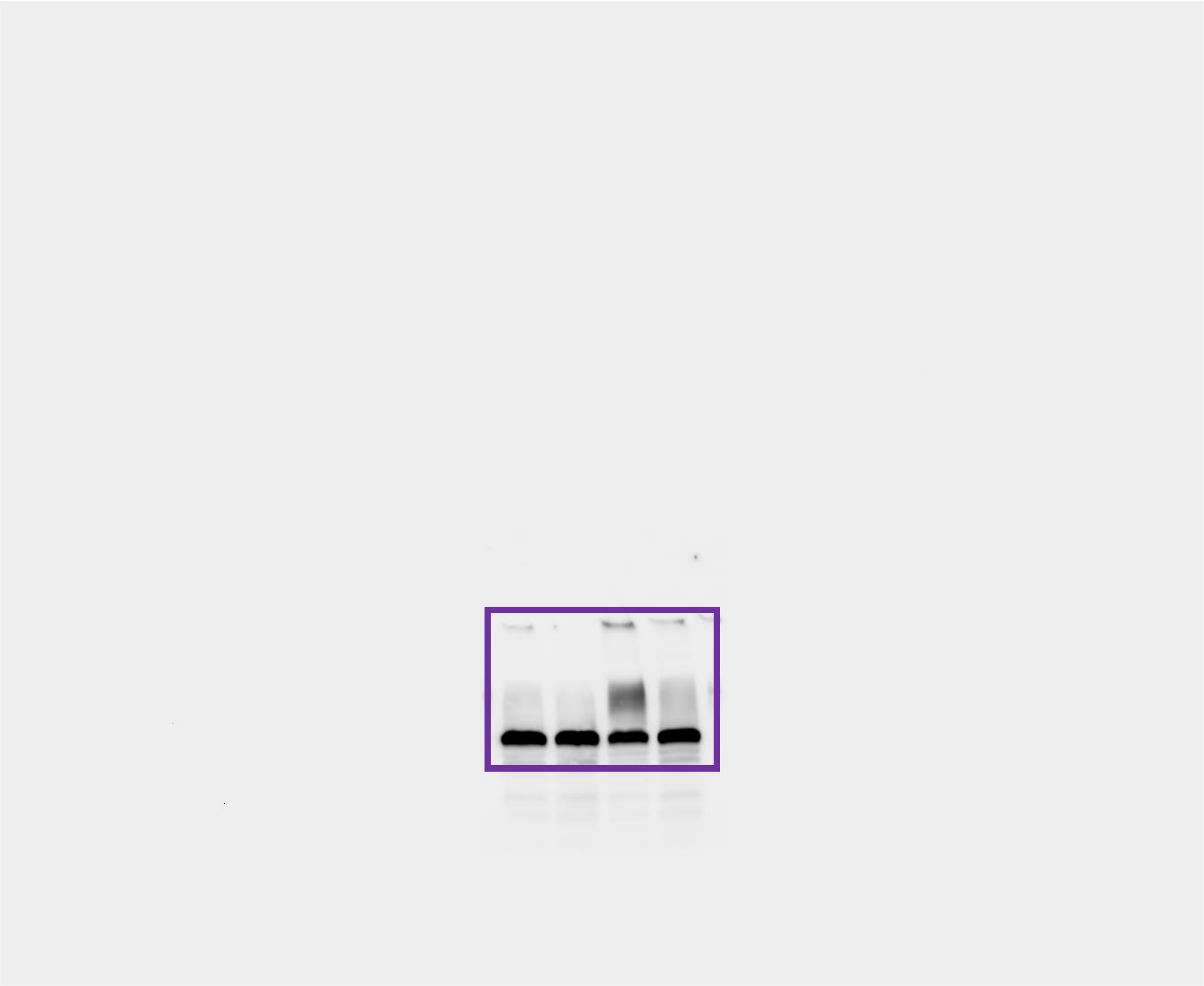

Figure S1C
MHCC97-H FLNA

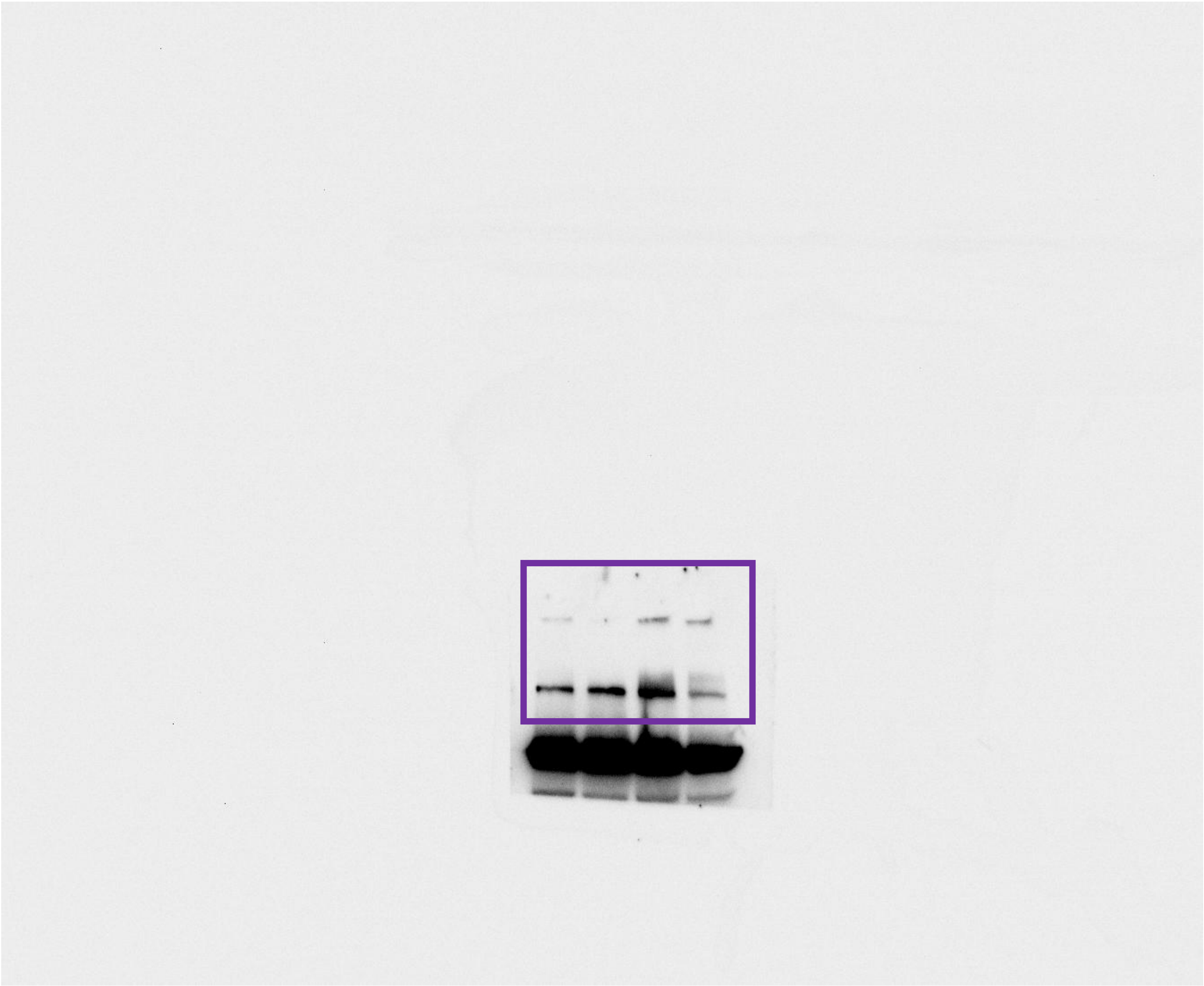

Figure S1C
Hep 3B FLNA

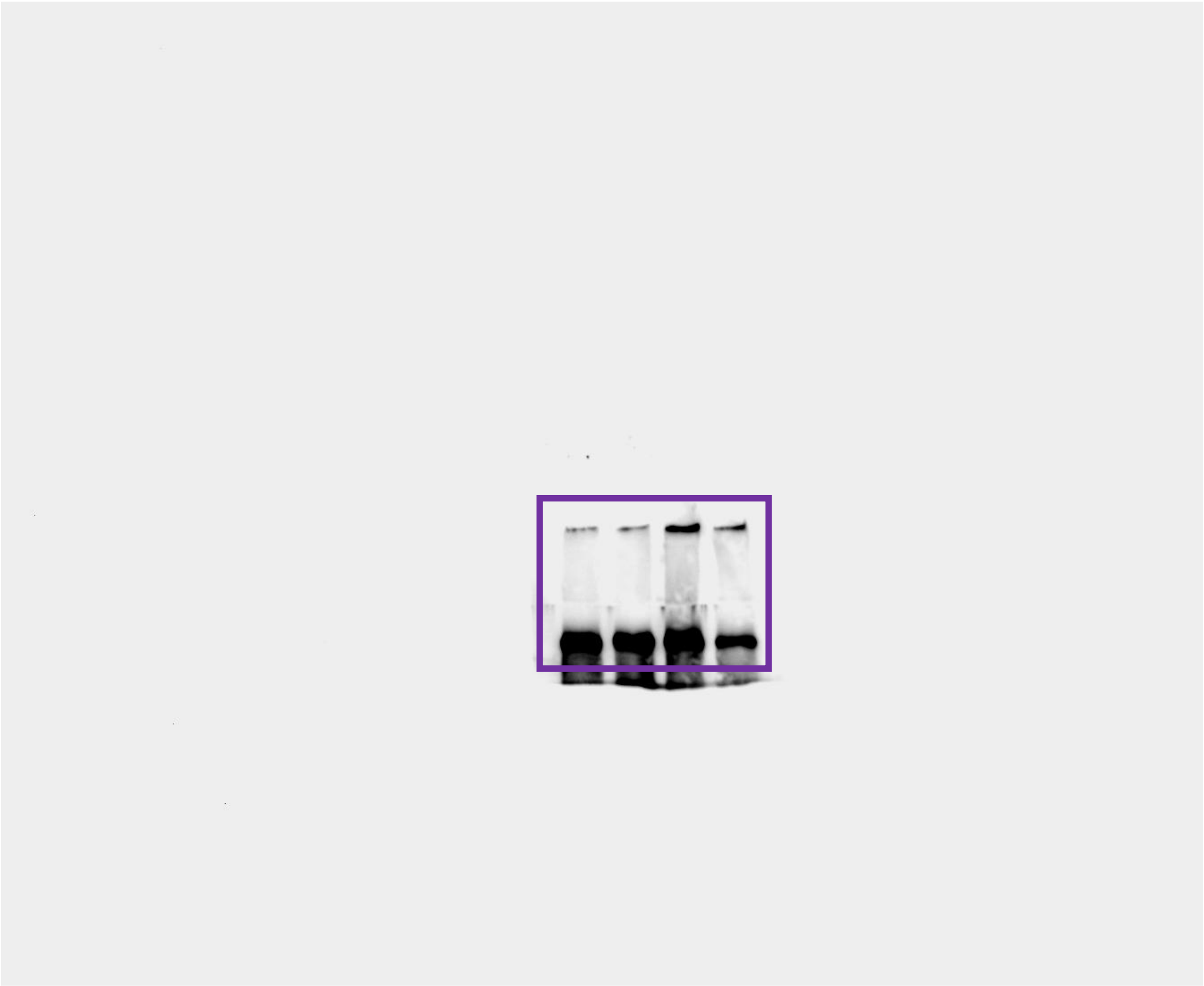

Figure S2A
MHCC97-H ATG7

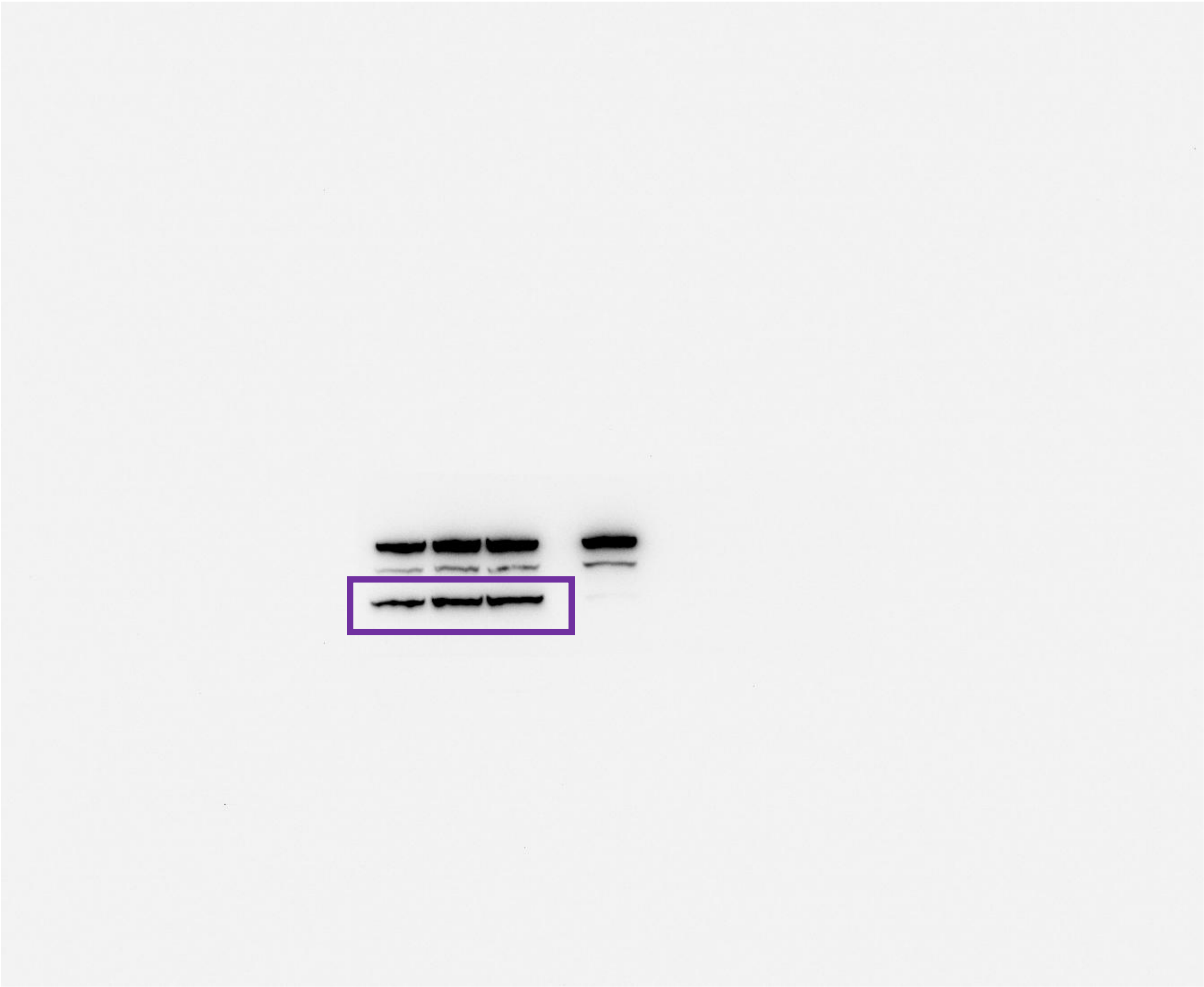

Figure S2A
MHCC97-H p62

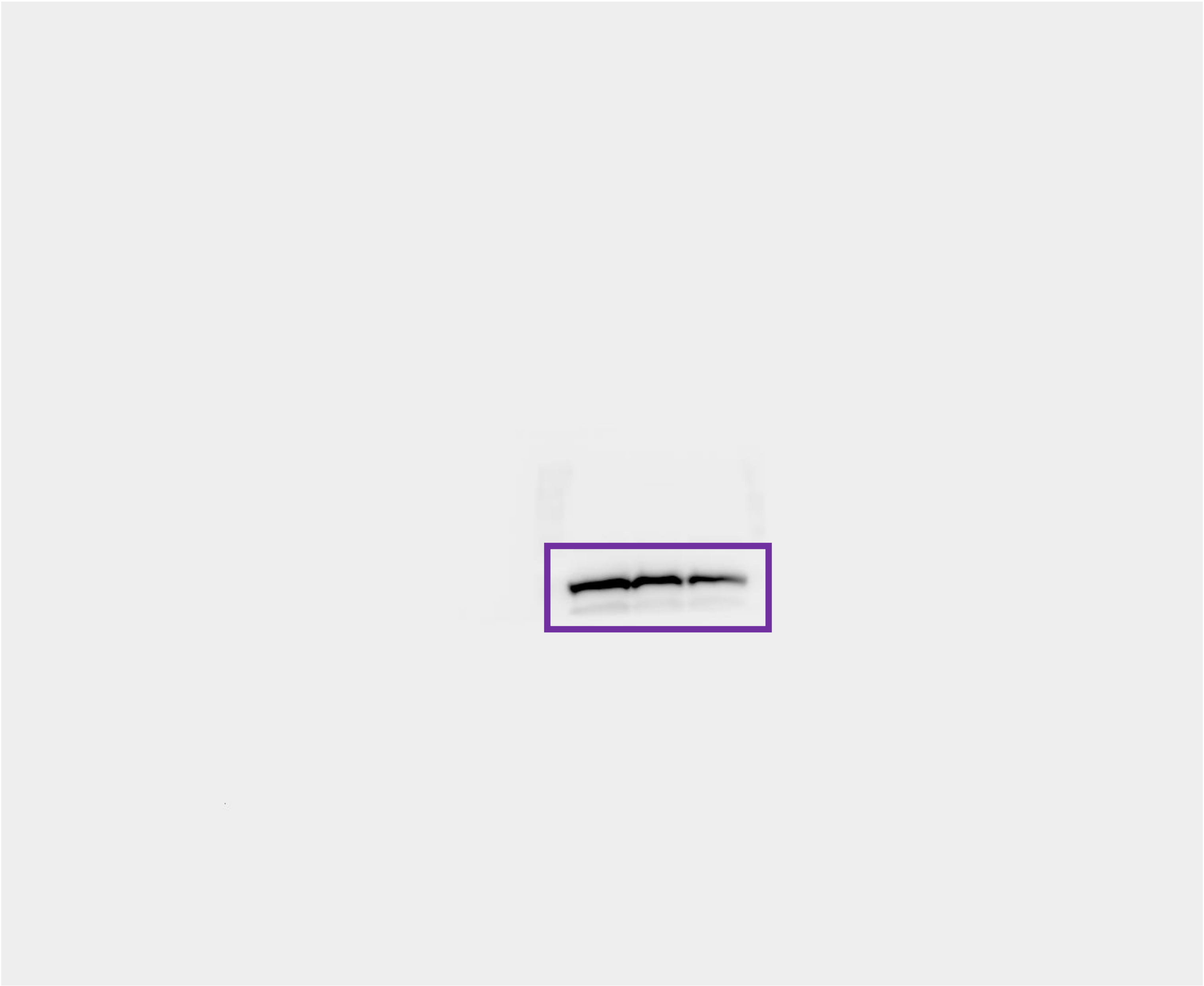

Figure S2A
MHCC97-H BECLIN1

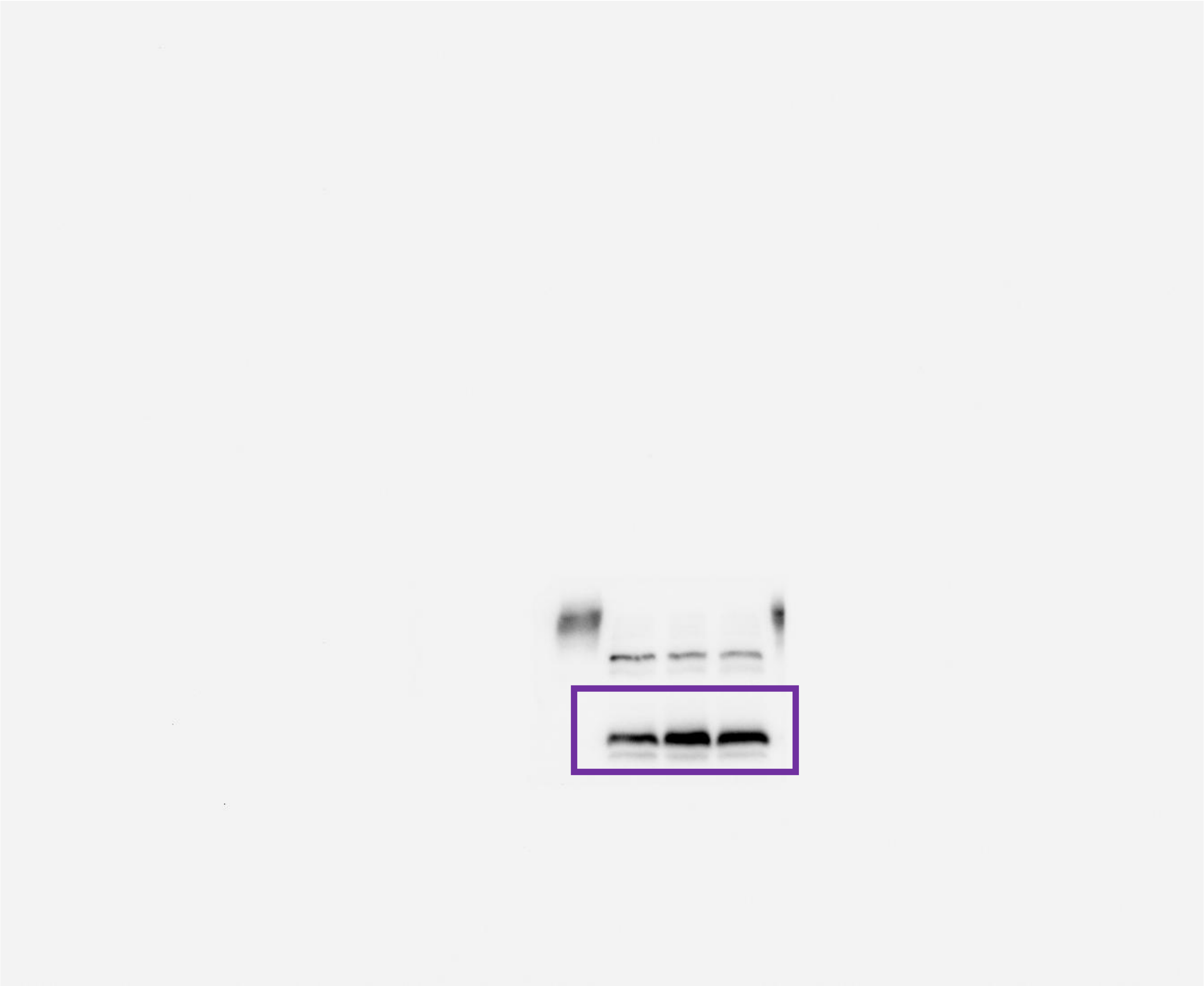

Figure S2A
MHCC97-H LC3

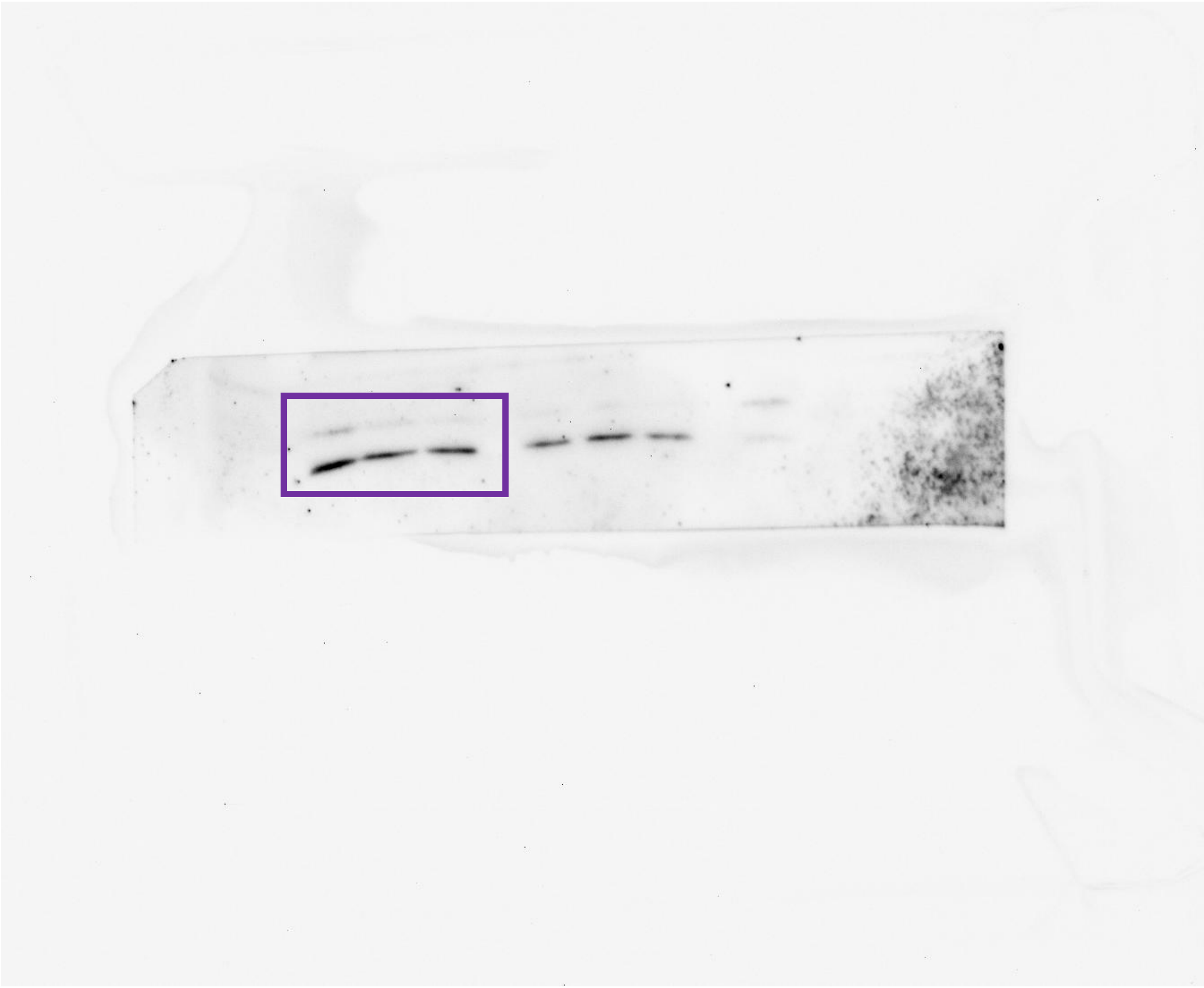

Figure S2A
MHCC97-H GAPDH

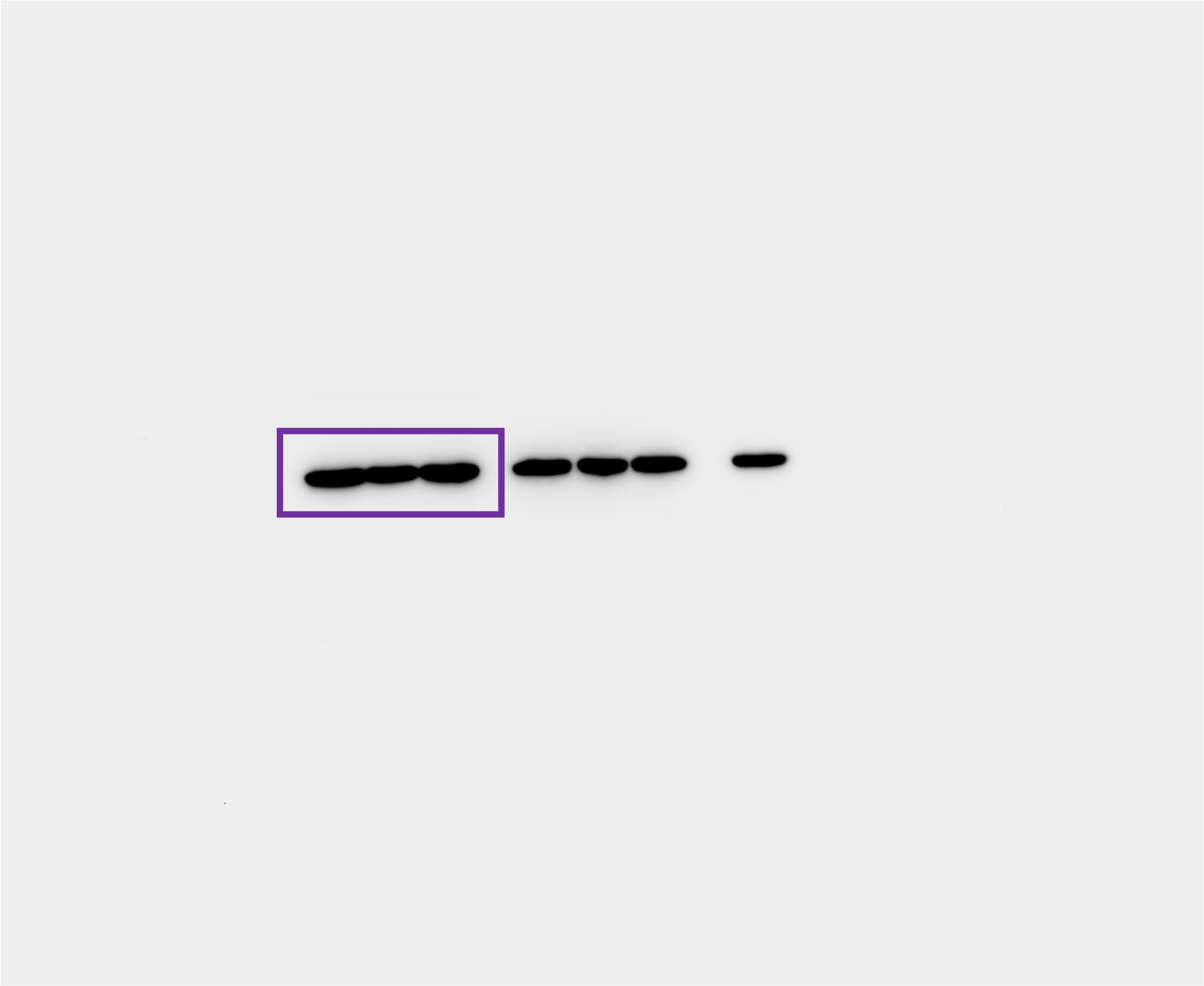

Figure S2A
Hep 3B ATG7

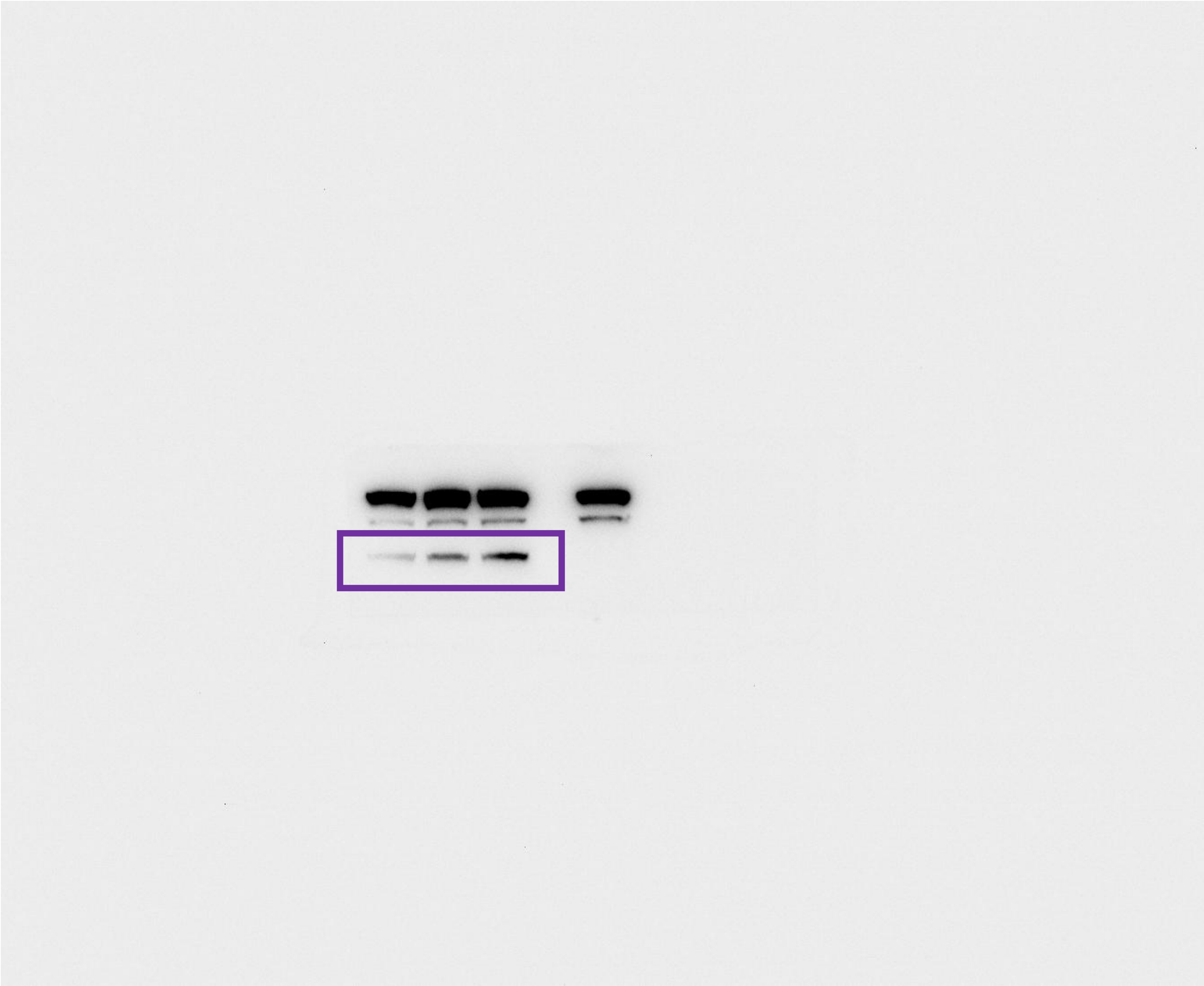

Figure S2A
Hep 3B p62

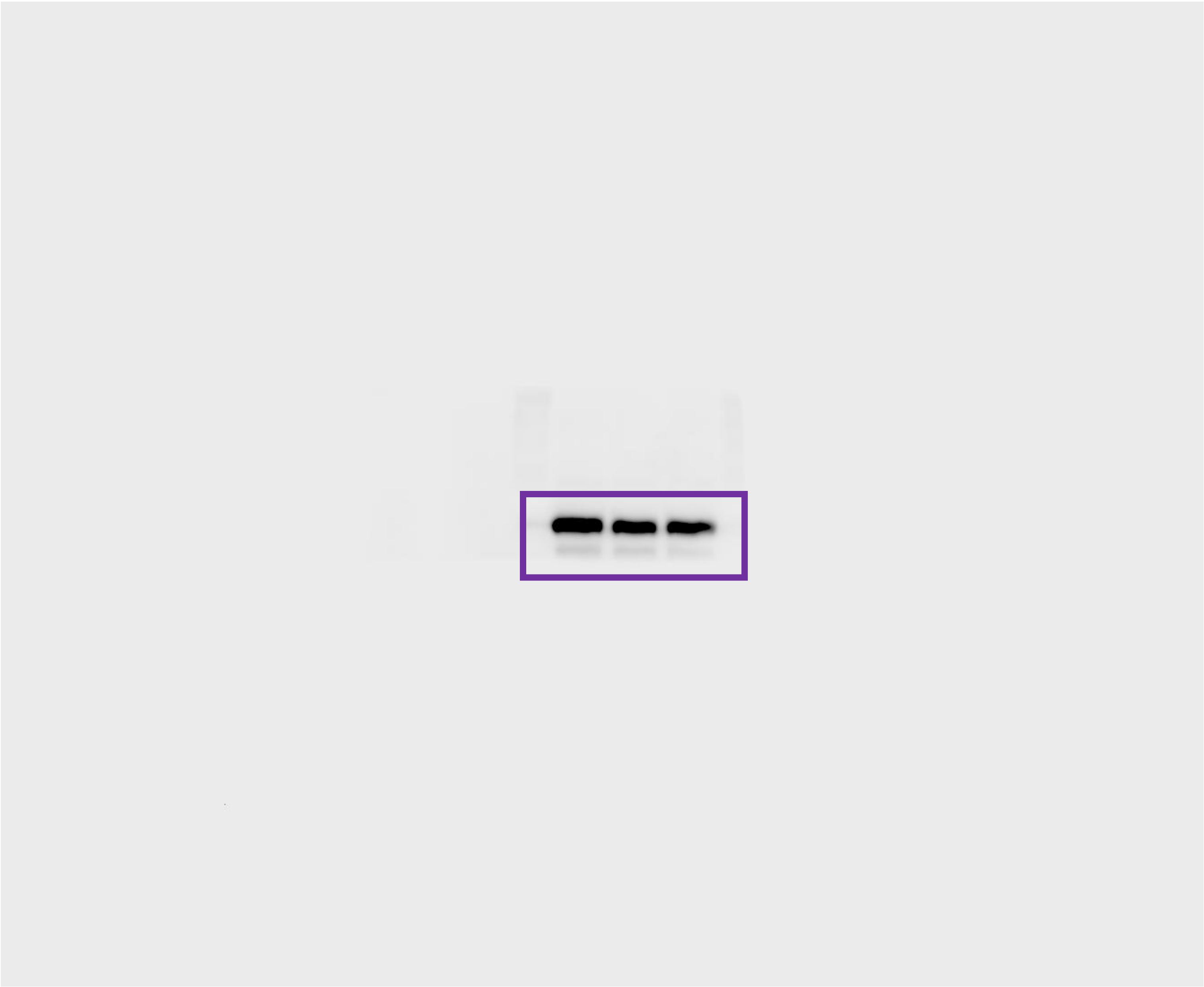

Figure S2A
Hep 3B BECLIN1

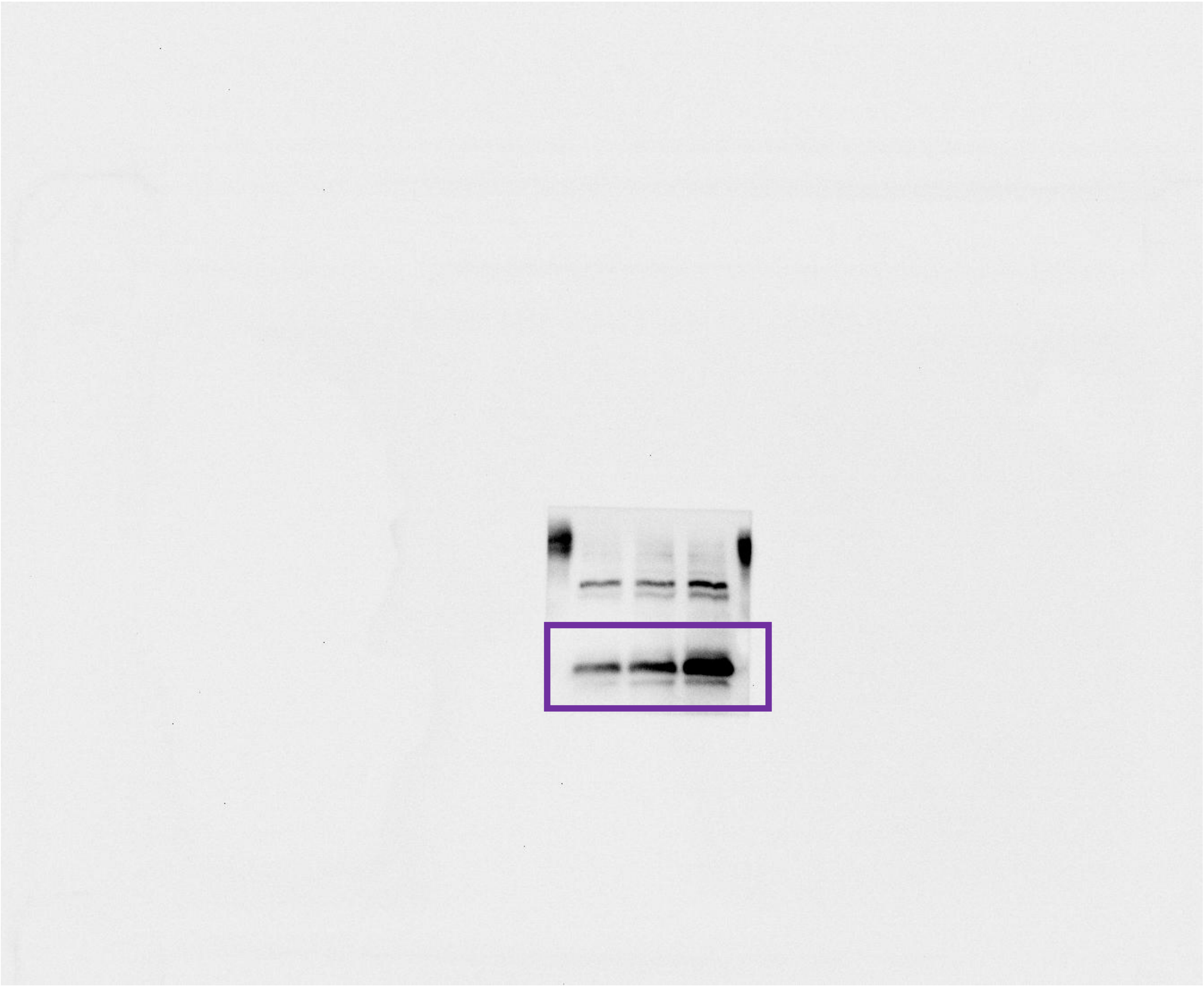

Figure S2A
Hep 3B LC3

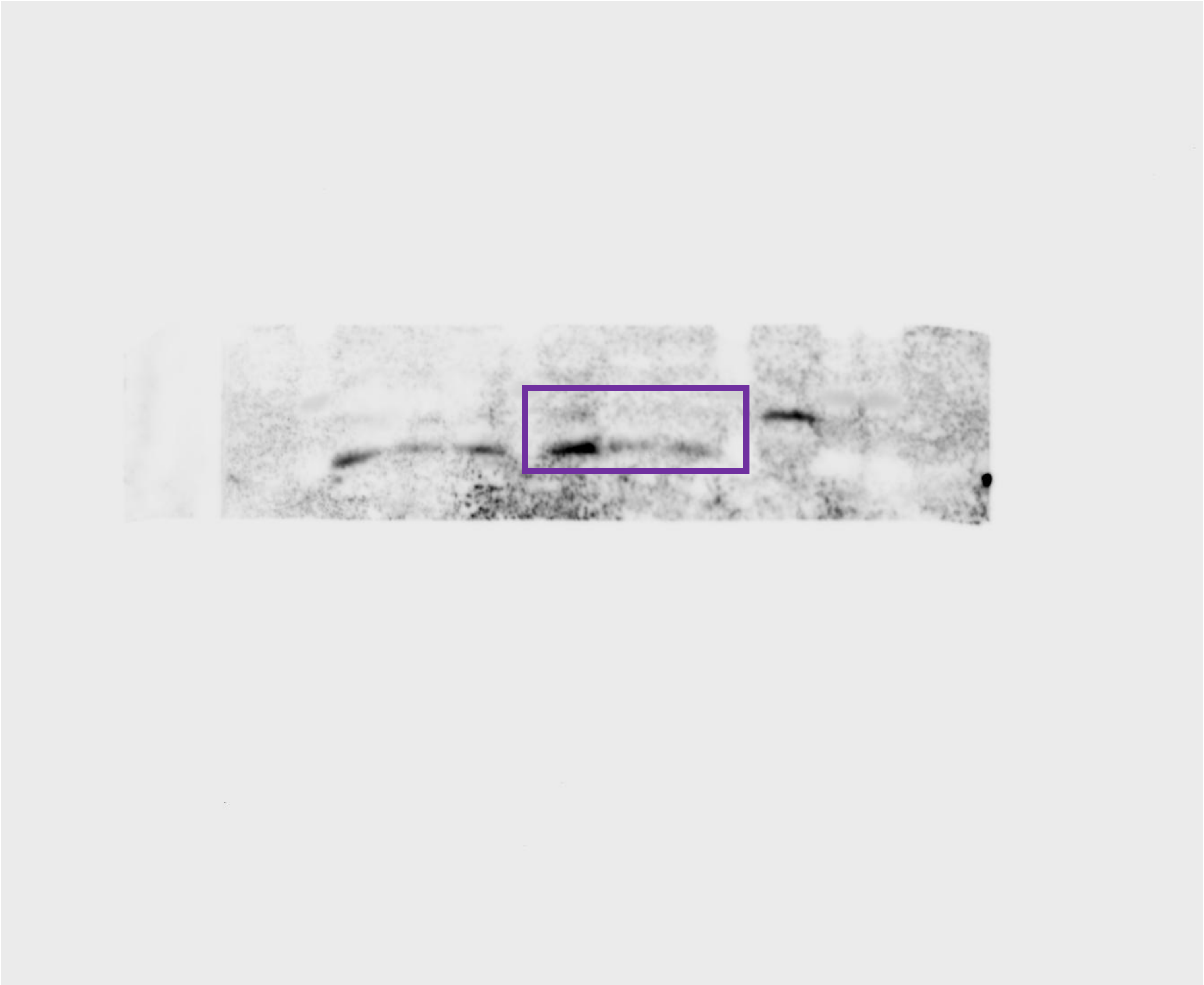

Figure S2A
Hep 3B GAPDH

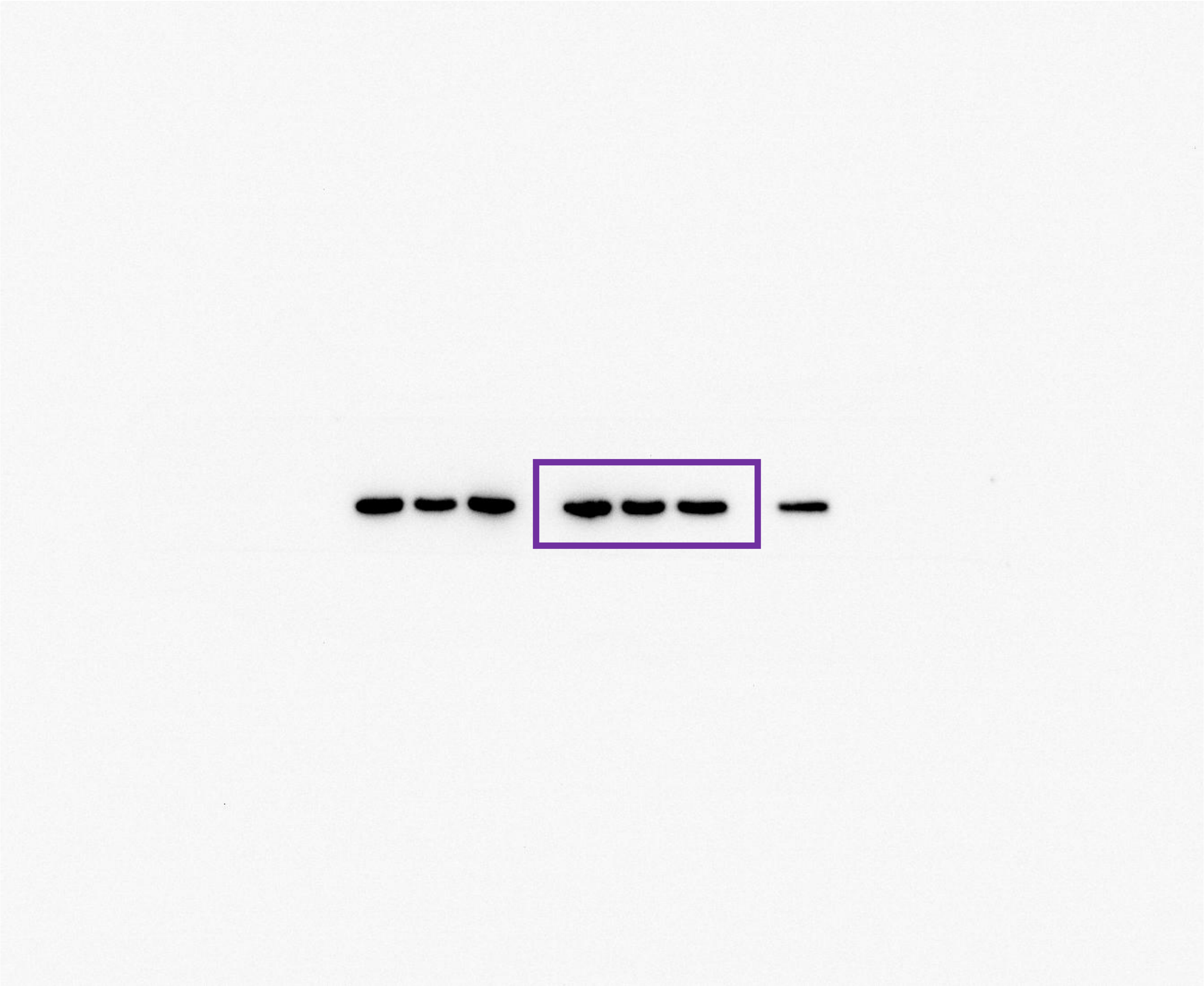

Figure S8C
FLNA

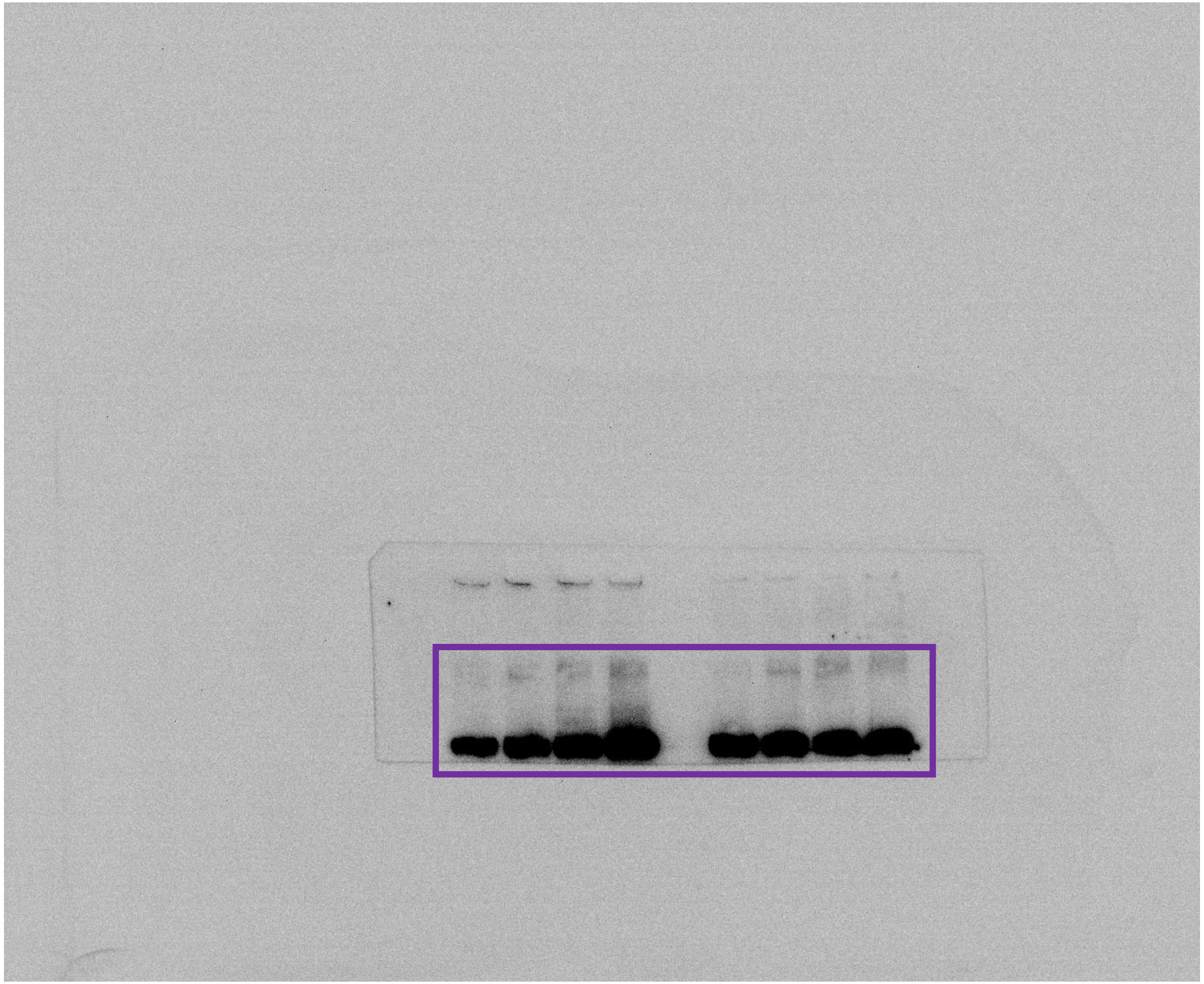

Figure S8C
DREBRIN

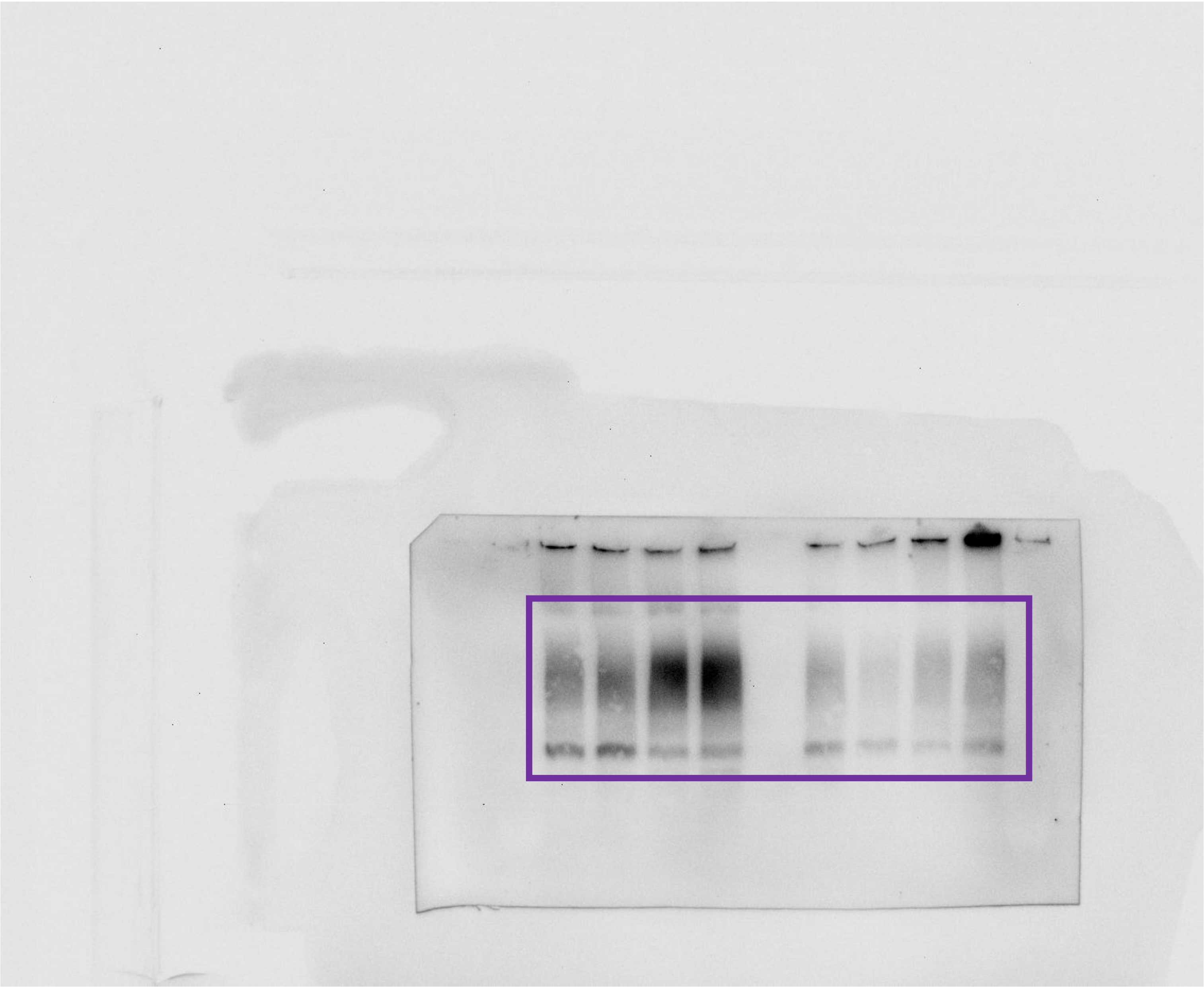

Figure S8C
G6PD

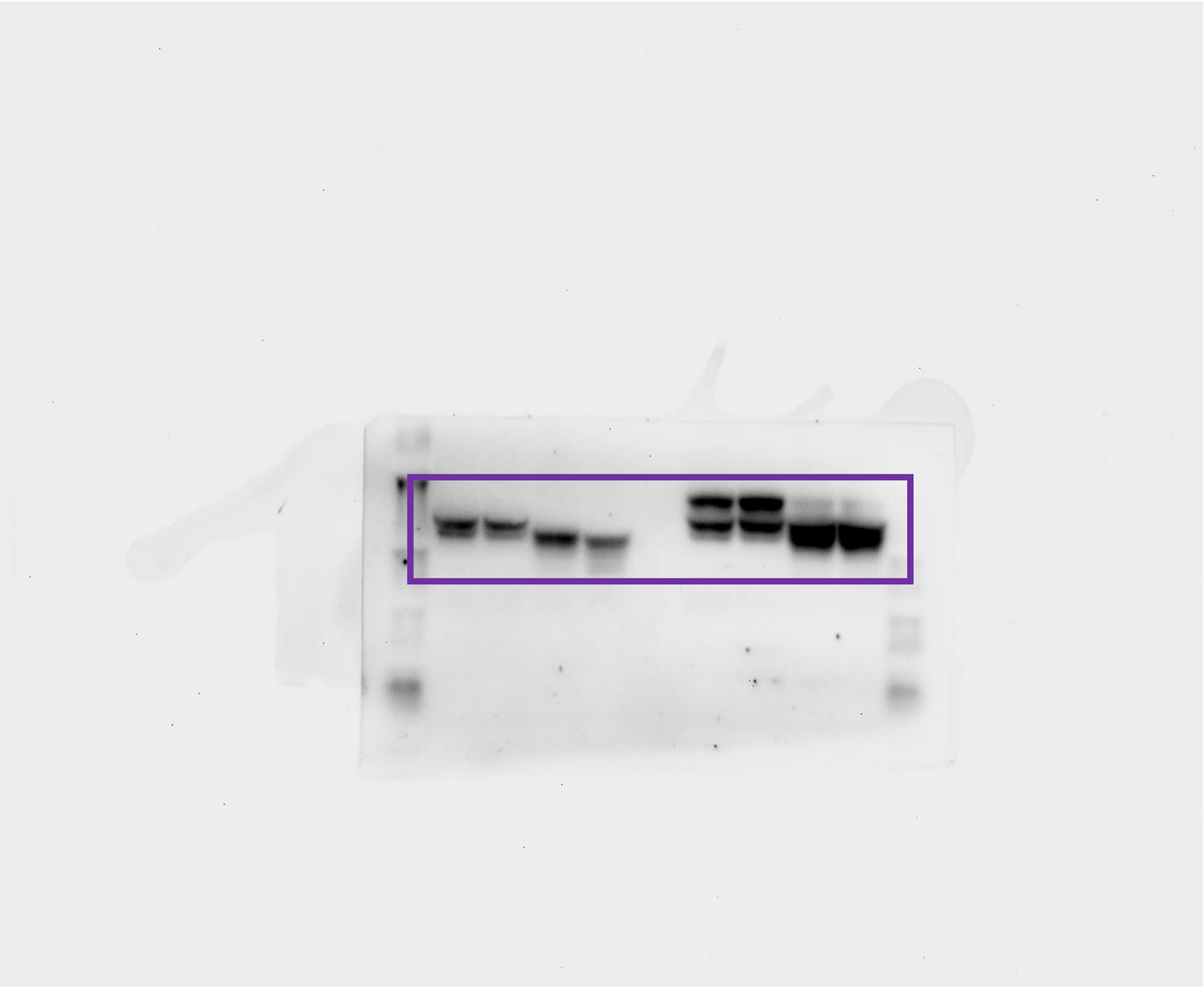

Figure S8C
GAPDH

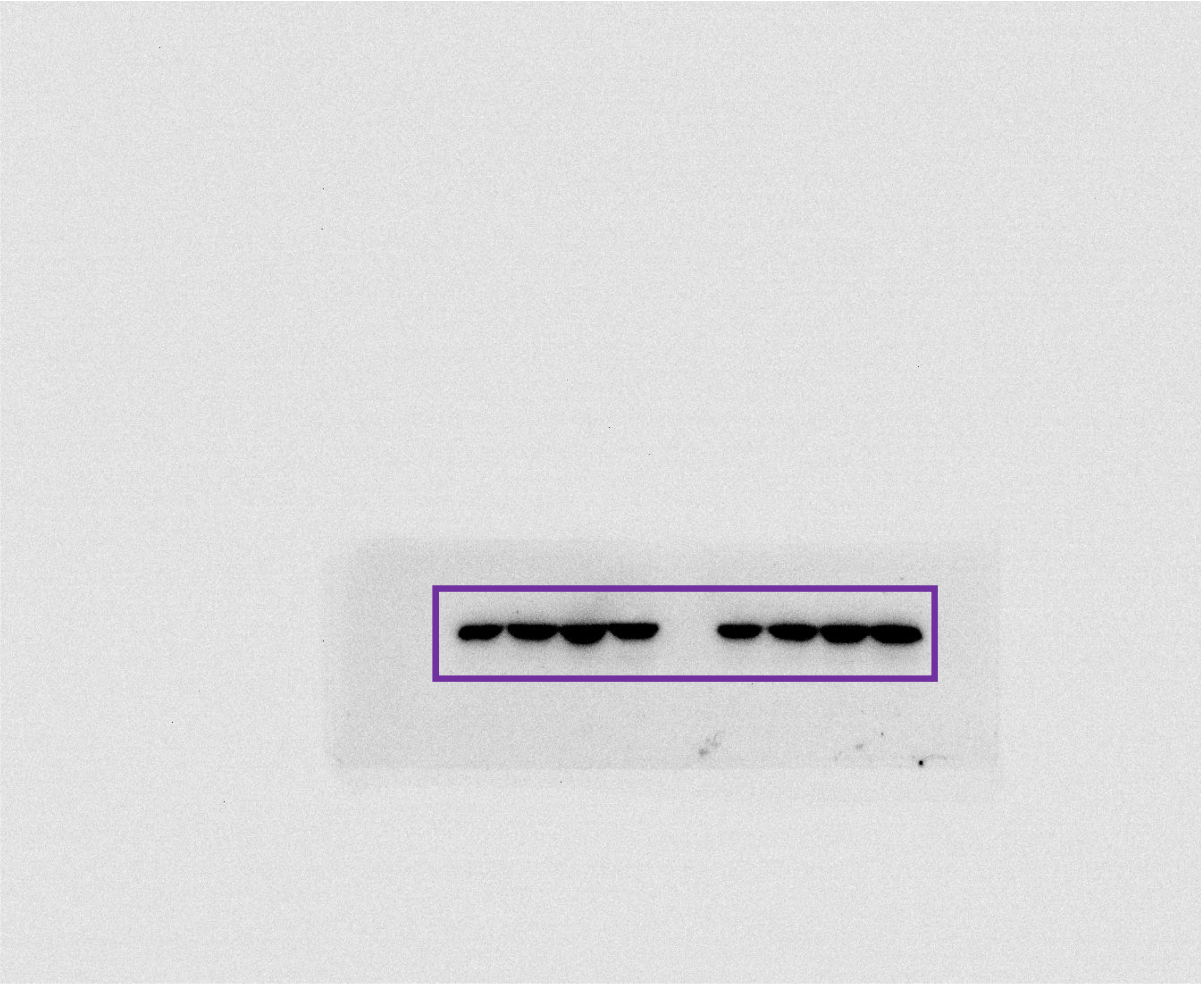

Figure S8D
FLNA

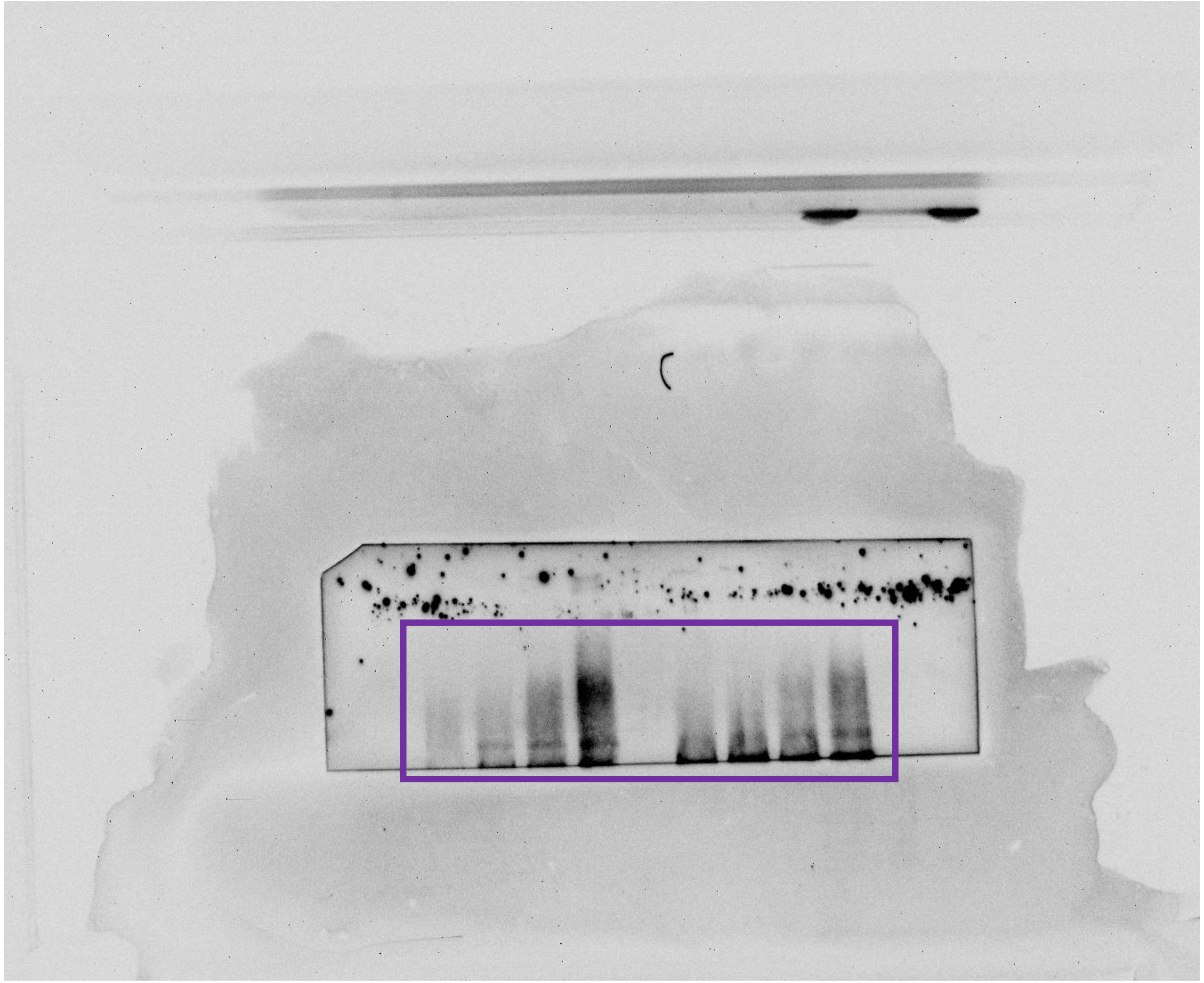

Figure S8D
DREBRIN

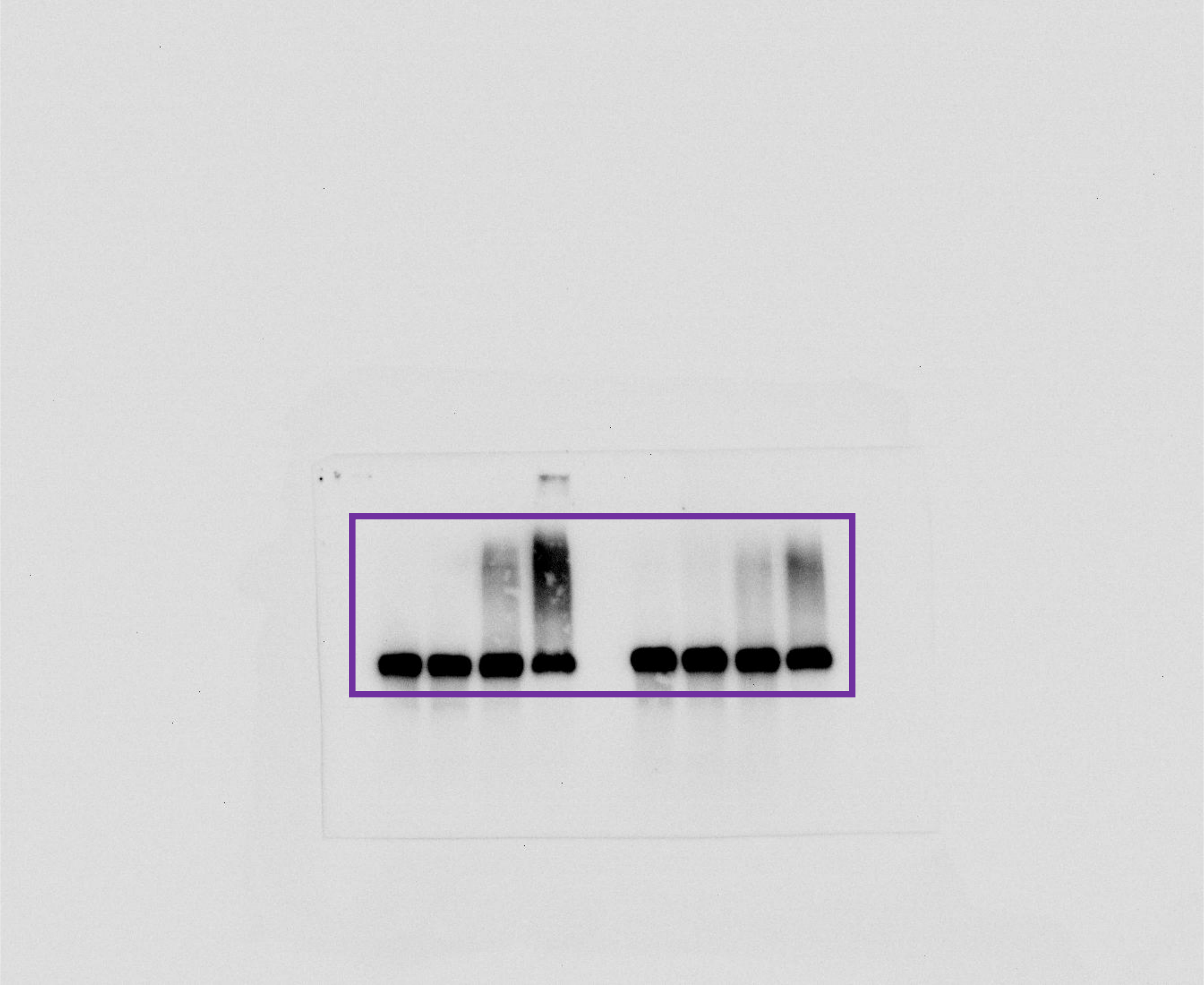

Figure S8D
GAPDH

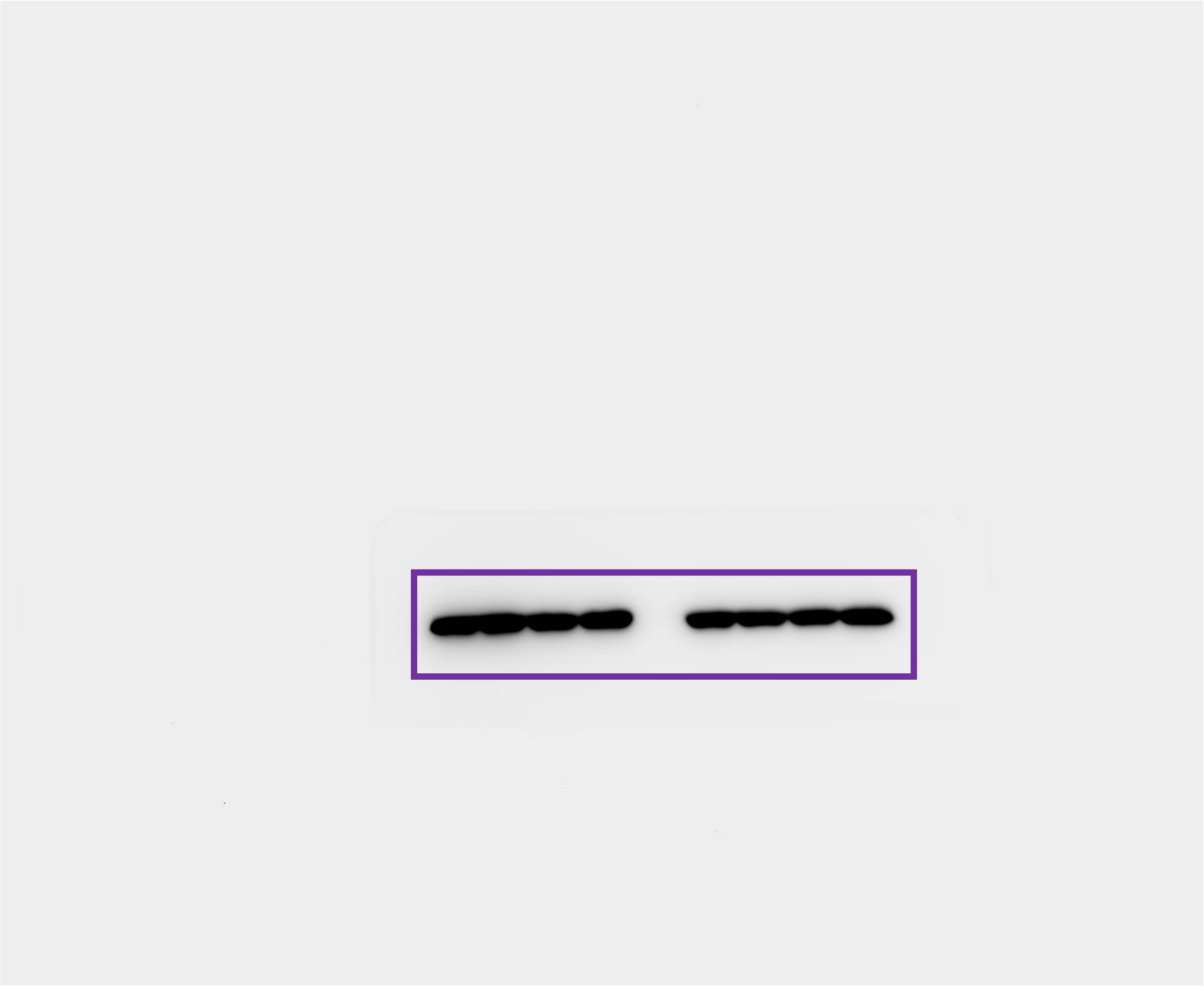

Figure S9B

Ctrl FLNA

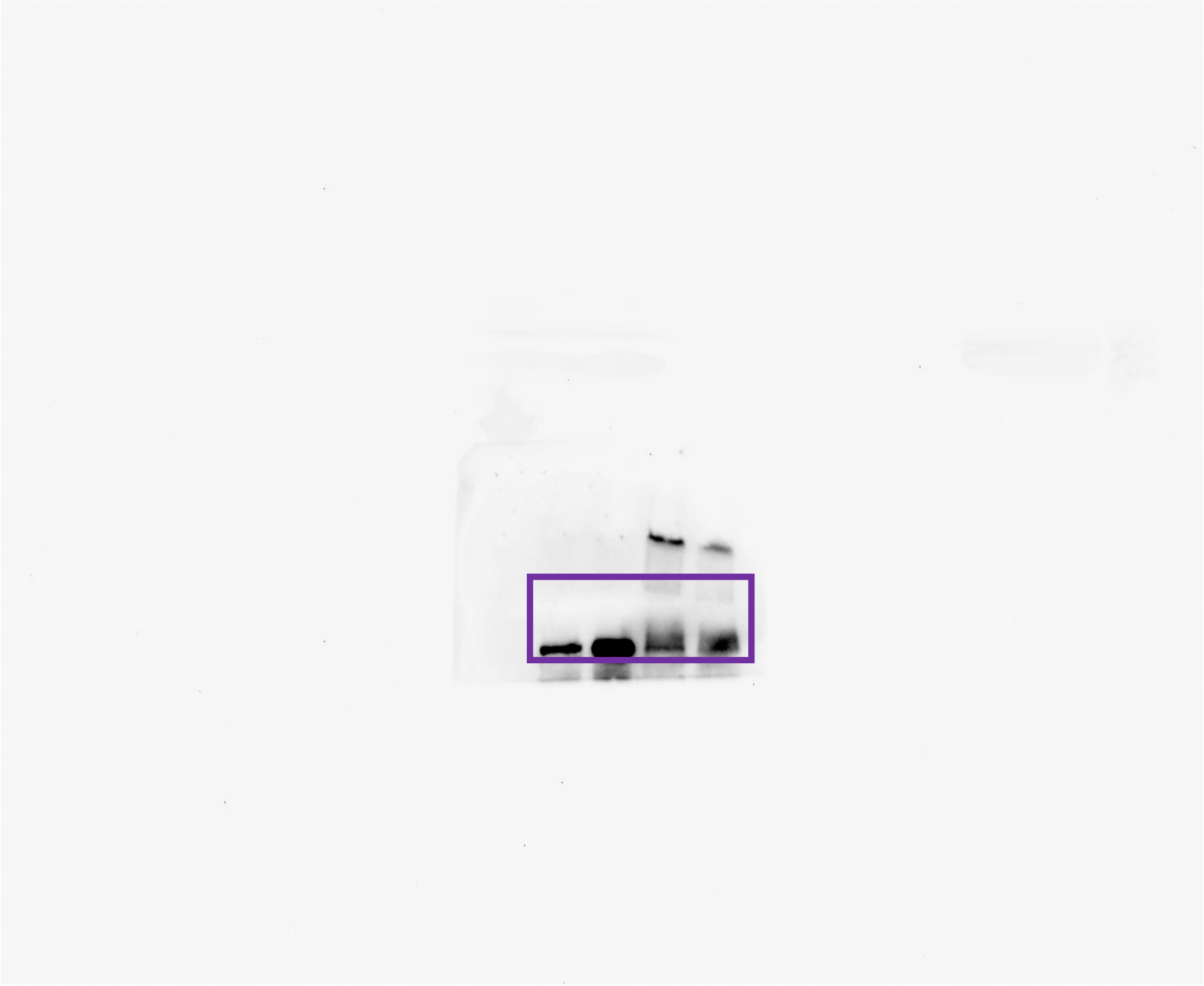

Figure S9B
GSH FLNA

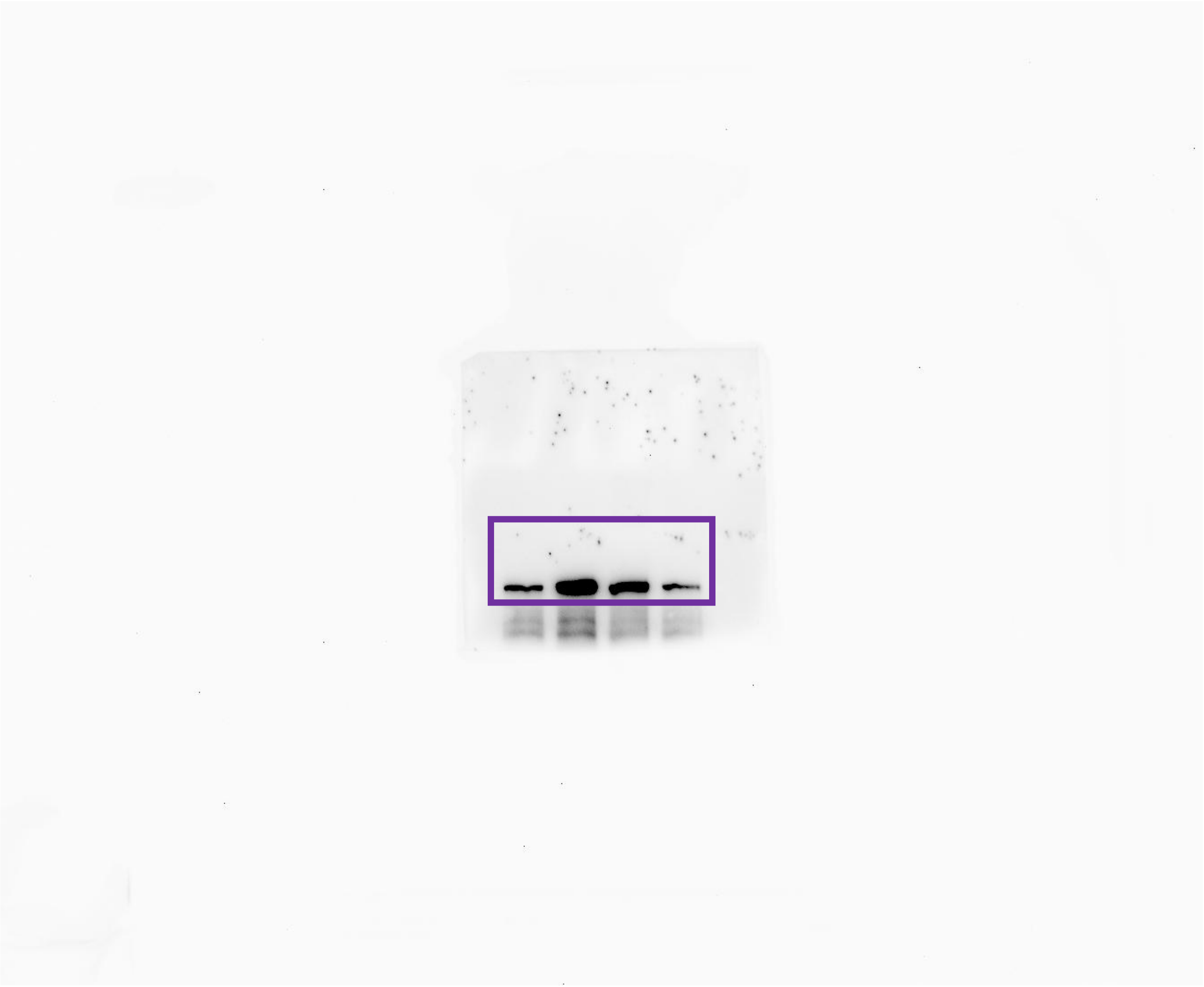

Figure S9B
Ctrl DREBRIN

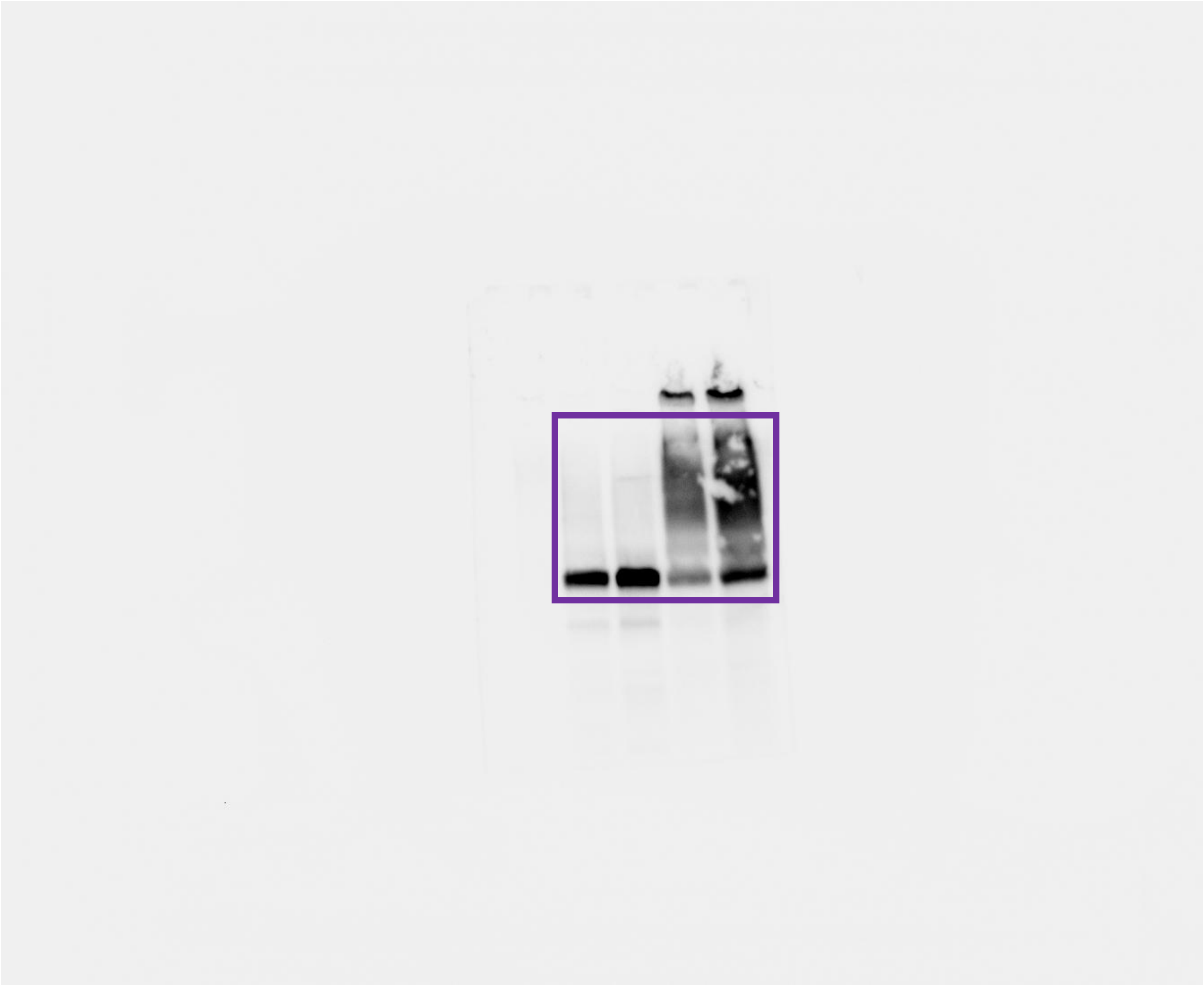

Figure S9B
GSH DREBRIN

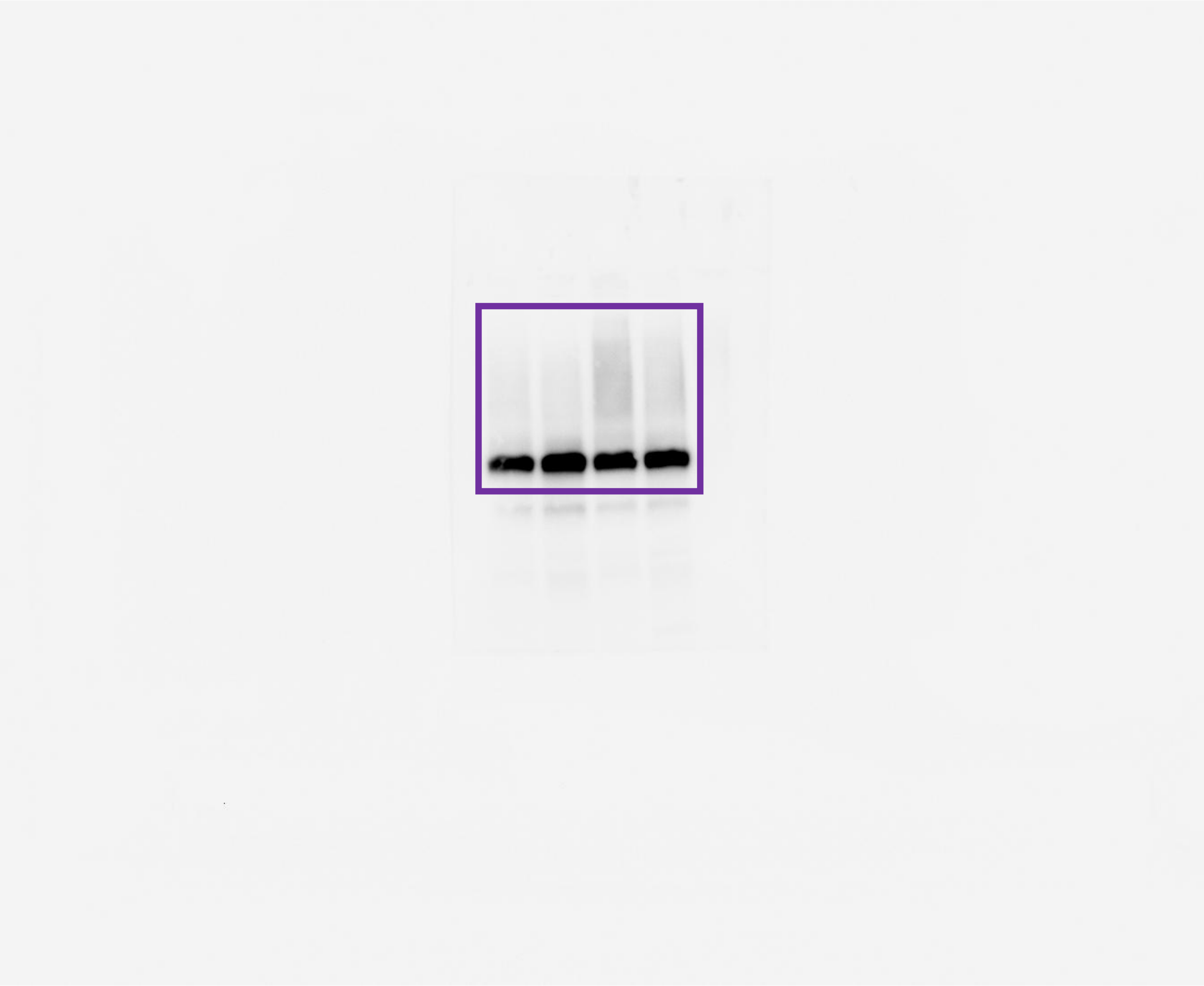

Figure S9B
Ctrl GSH GAPDH

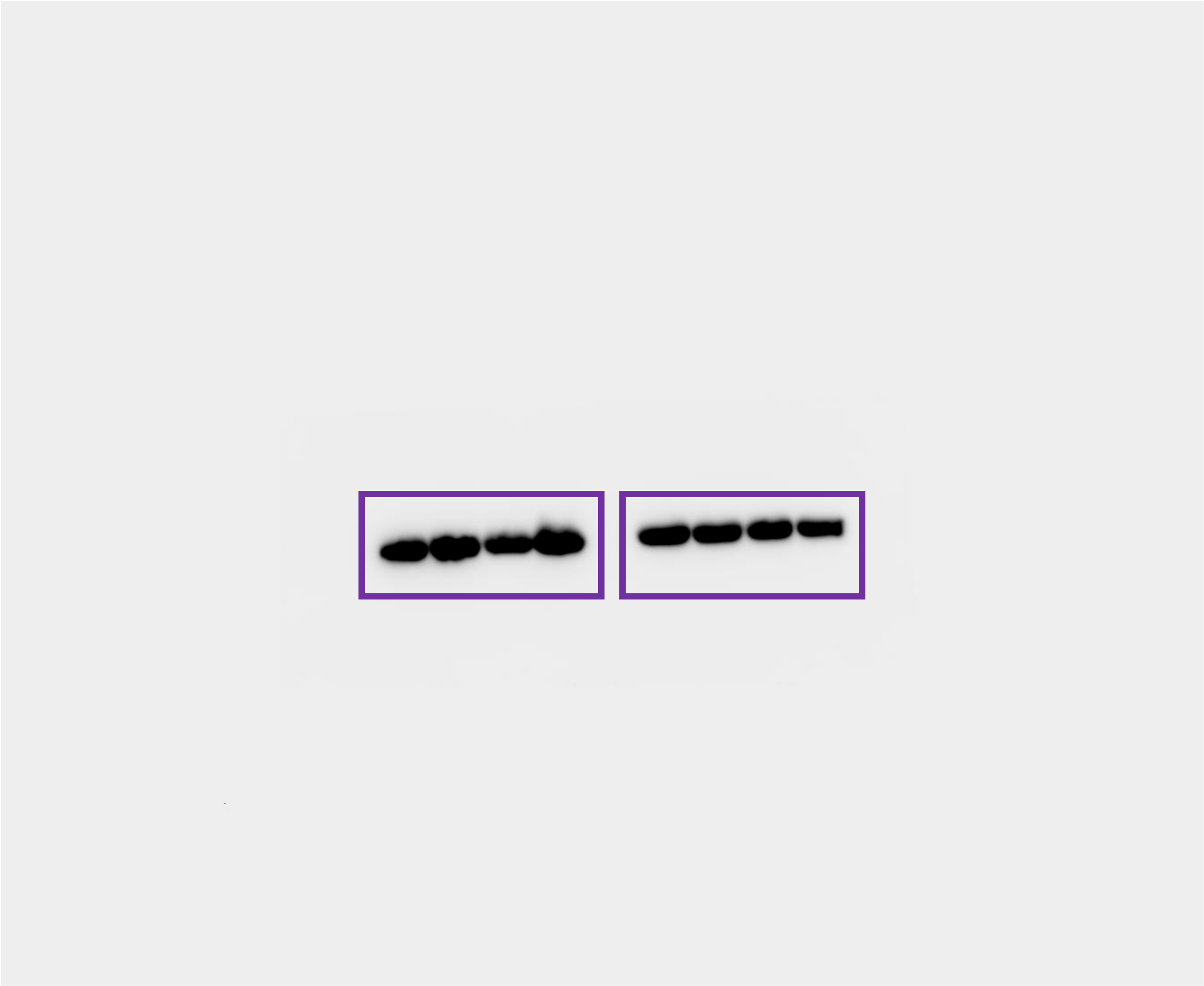

Figure S10A
MTL ATF4

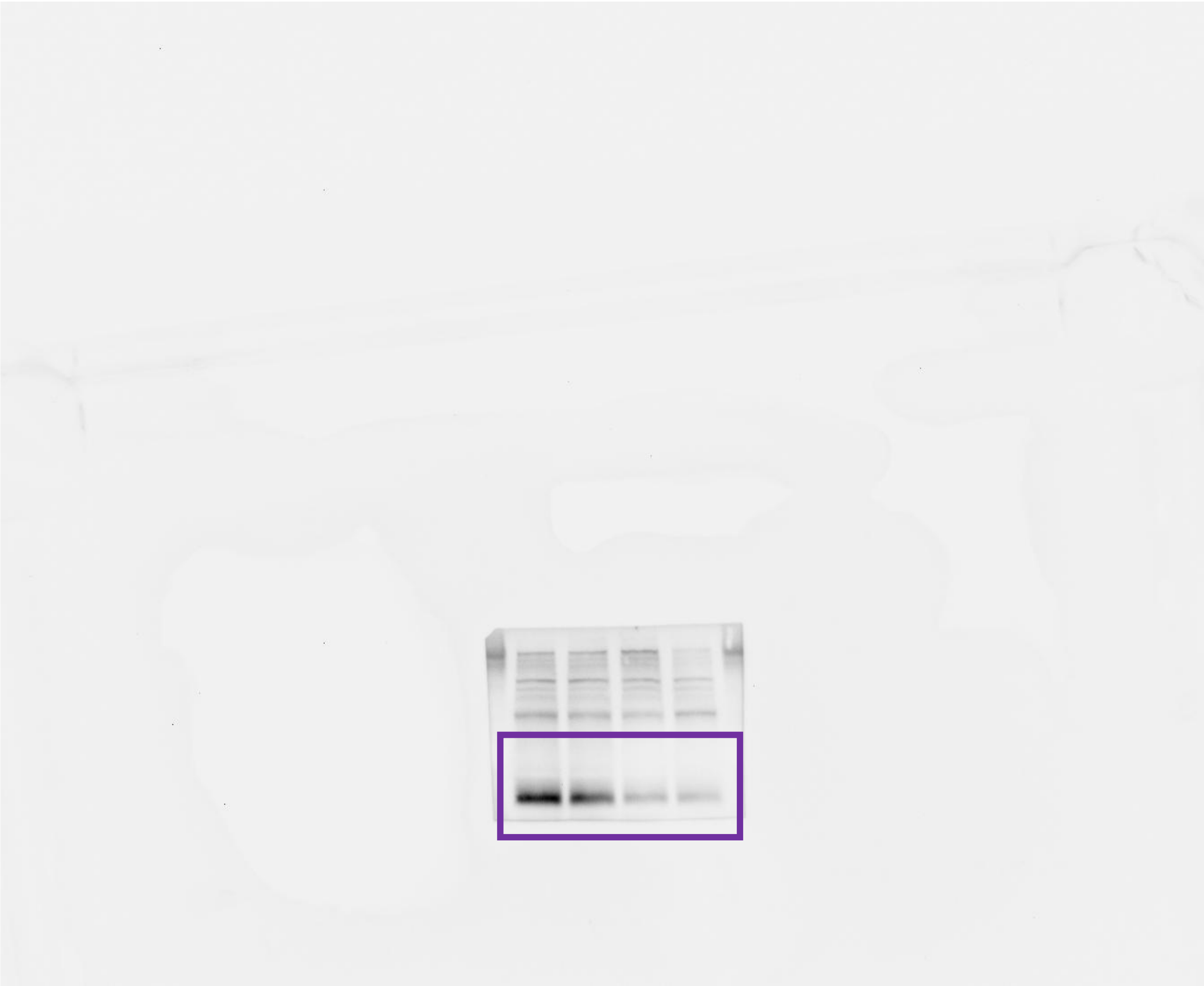

Figure S10A
MLT p-eIF2α

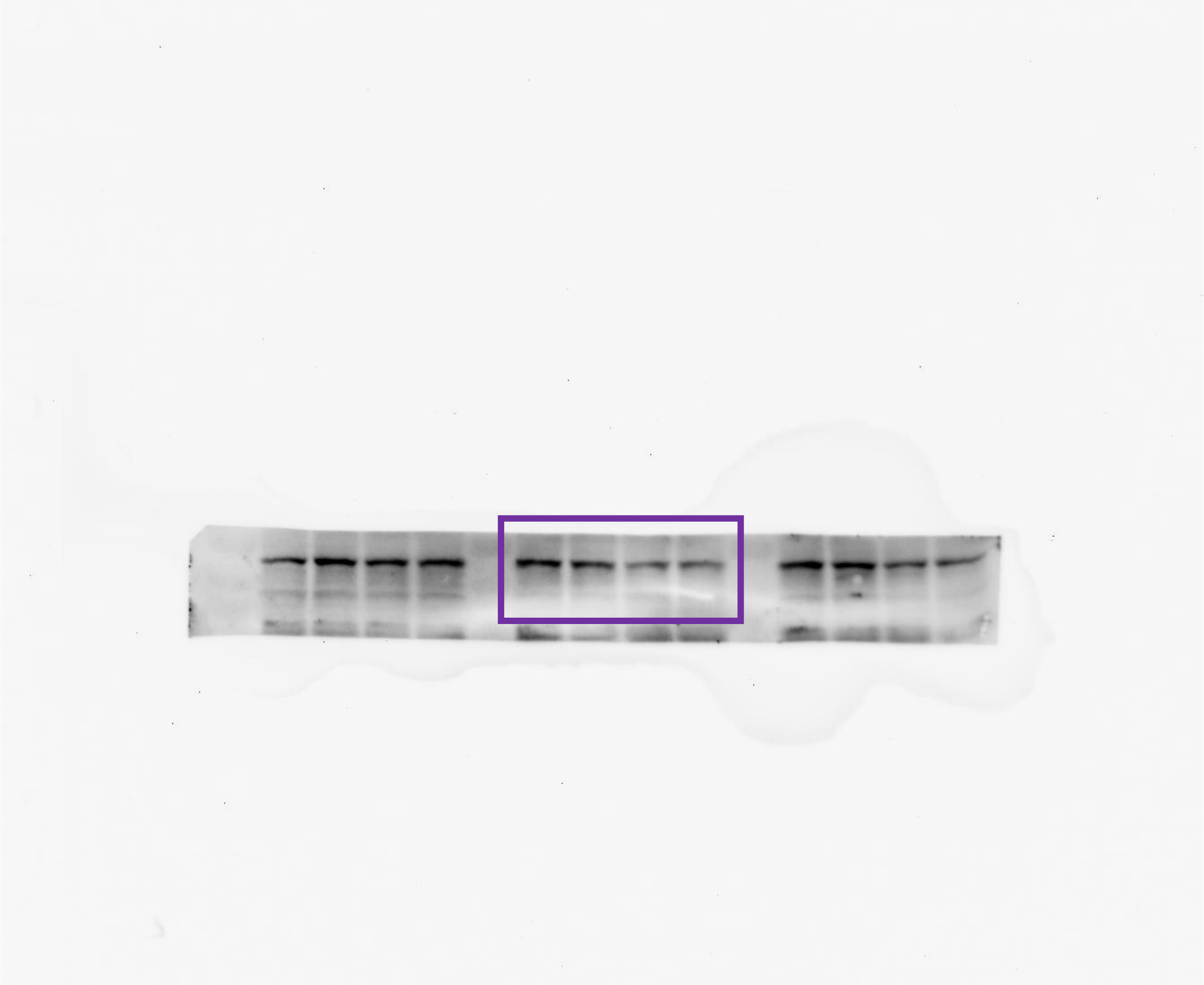

Figure S10A
MLT eIF2α

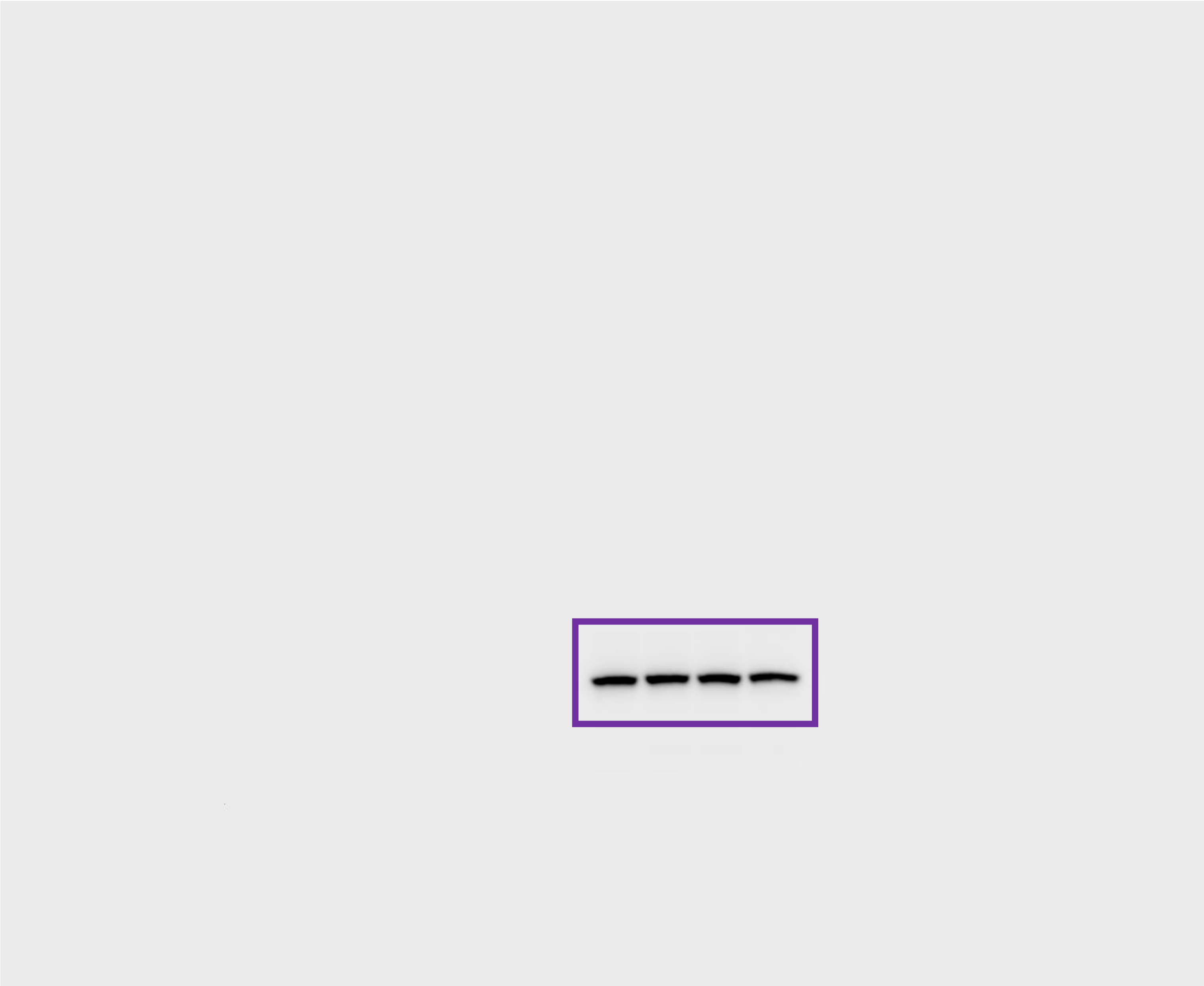

Figure S10A
MLT GAPDH

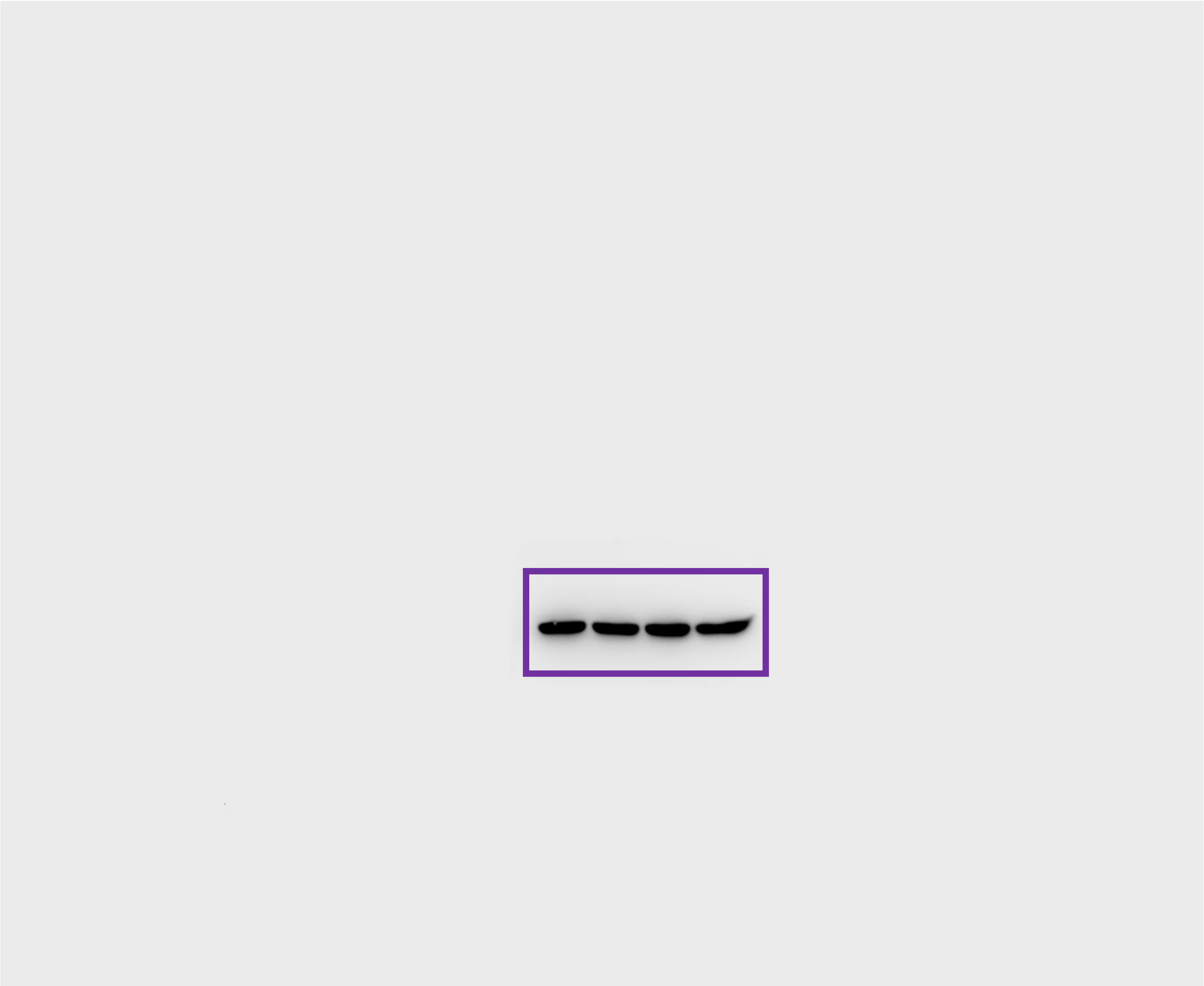

Figure S10B
4PA ATF4

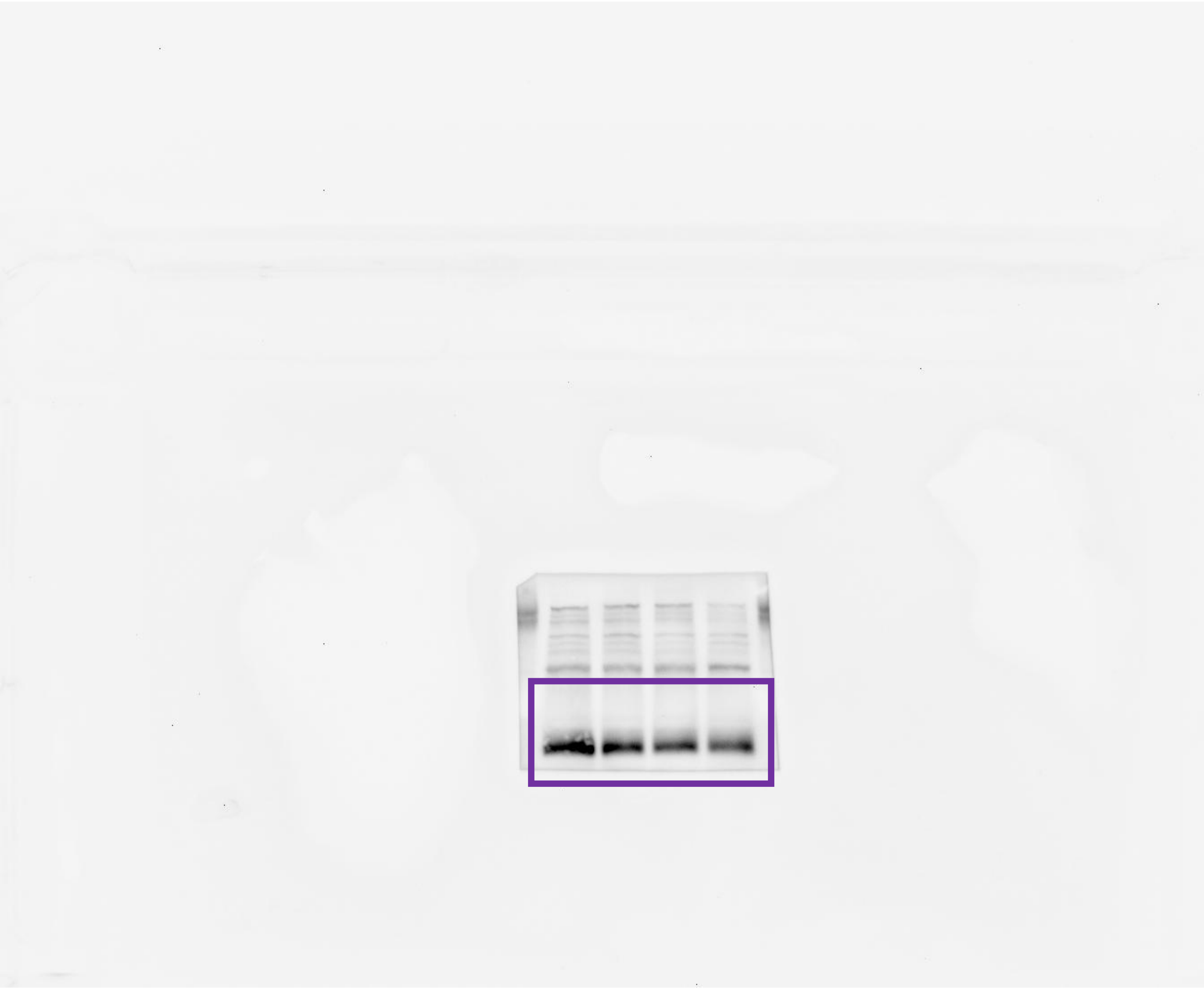

Figure S10B
4PA p-eIF2α

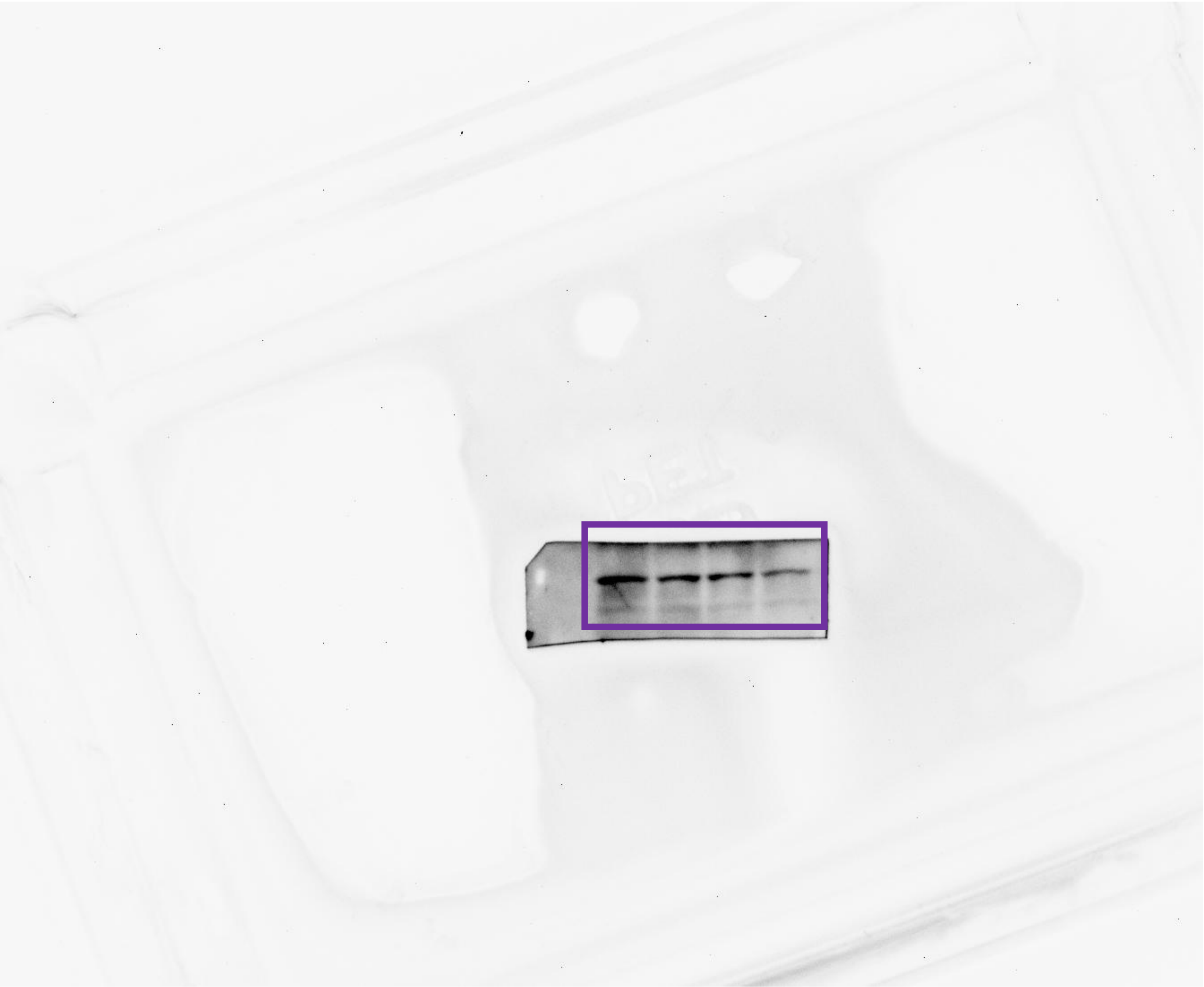

Figure S10B
4PA eIF2α

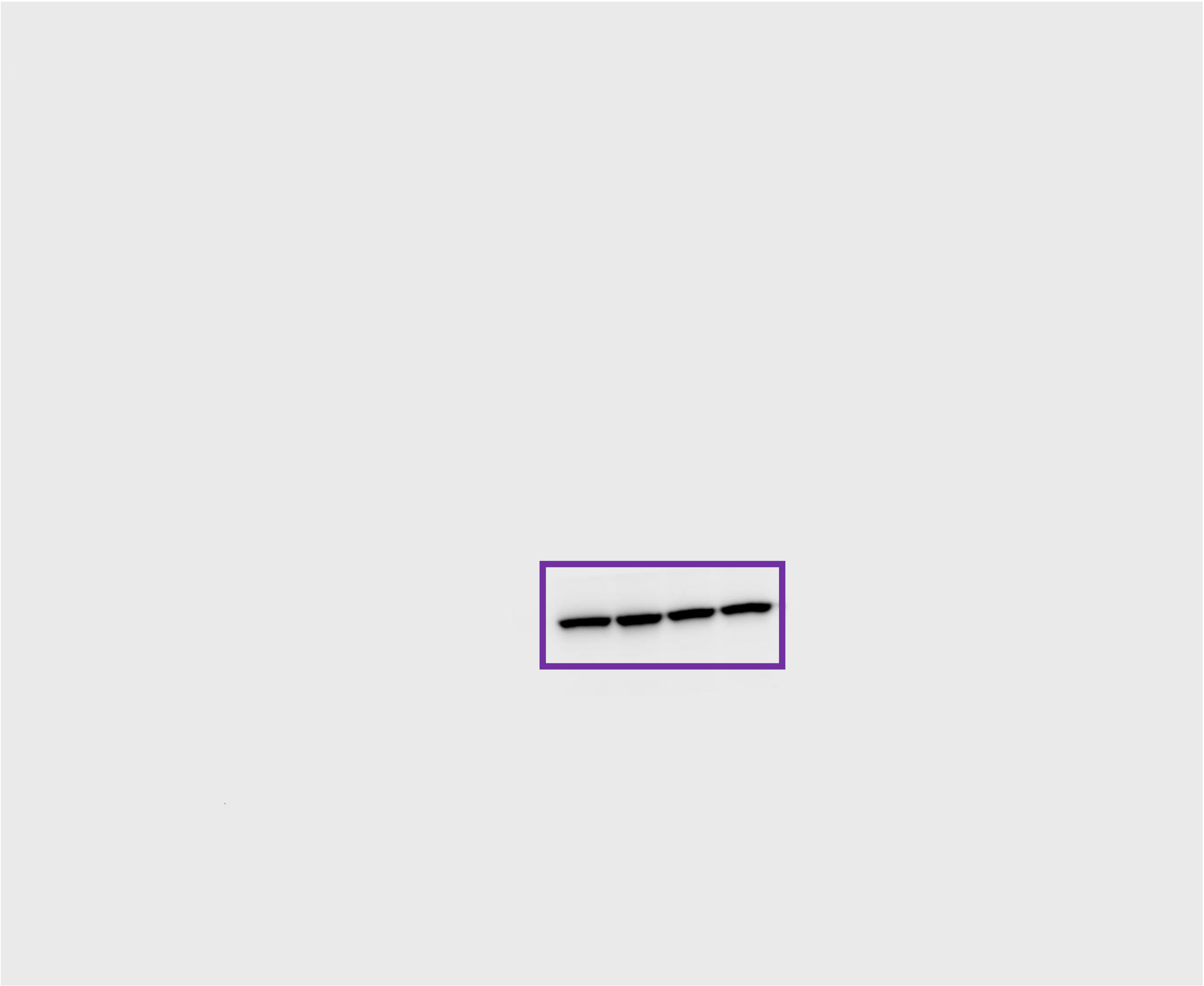

Figure S10B
4PA GAPDH

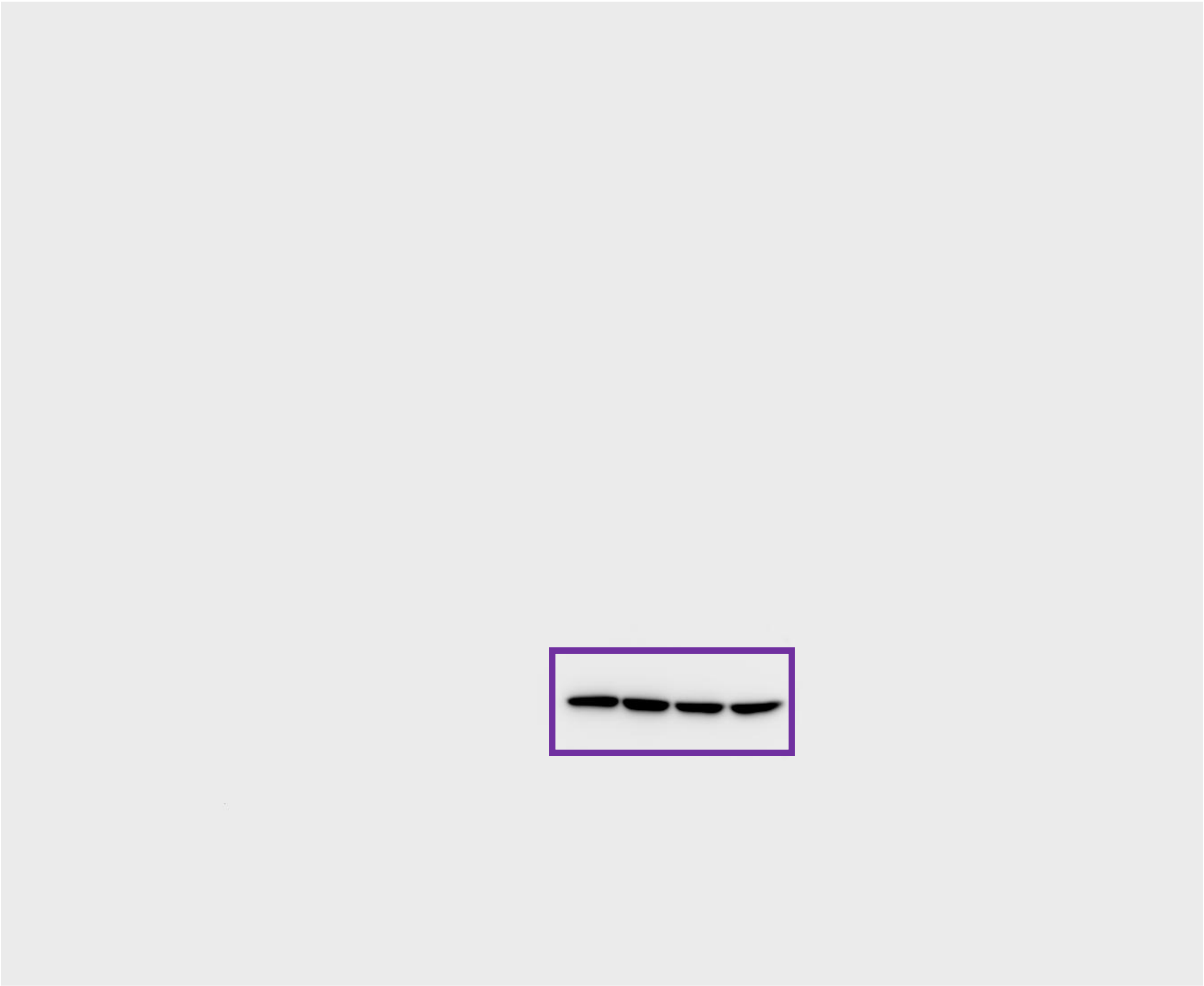

Figure S10C
MLT p62

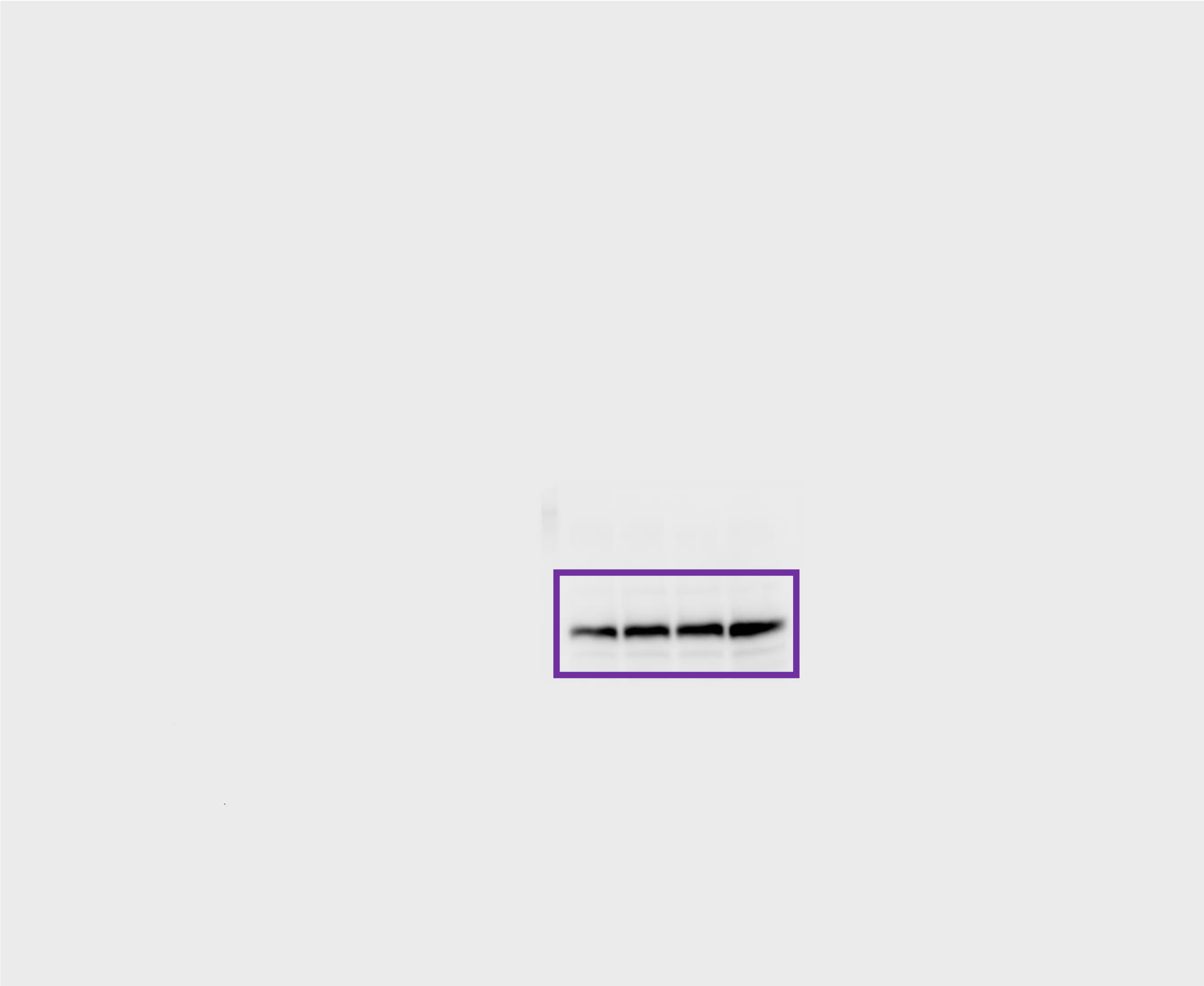

Figure S10C
MLT BECLIN1

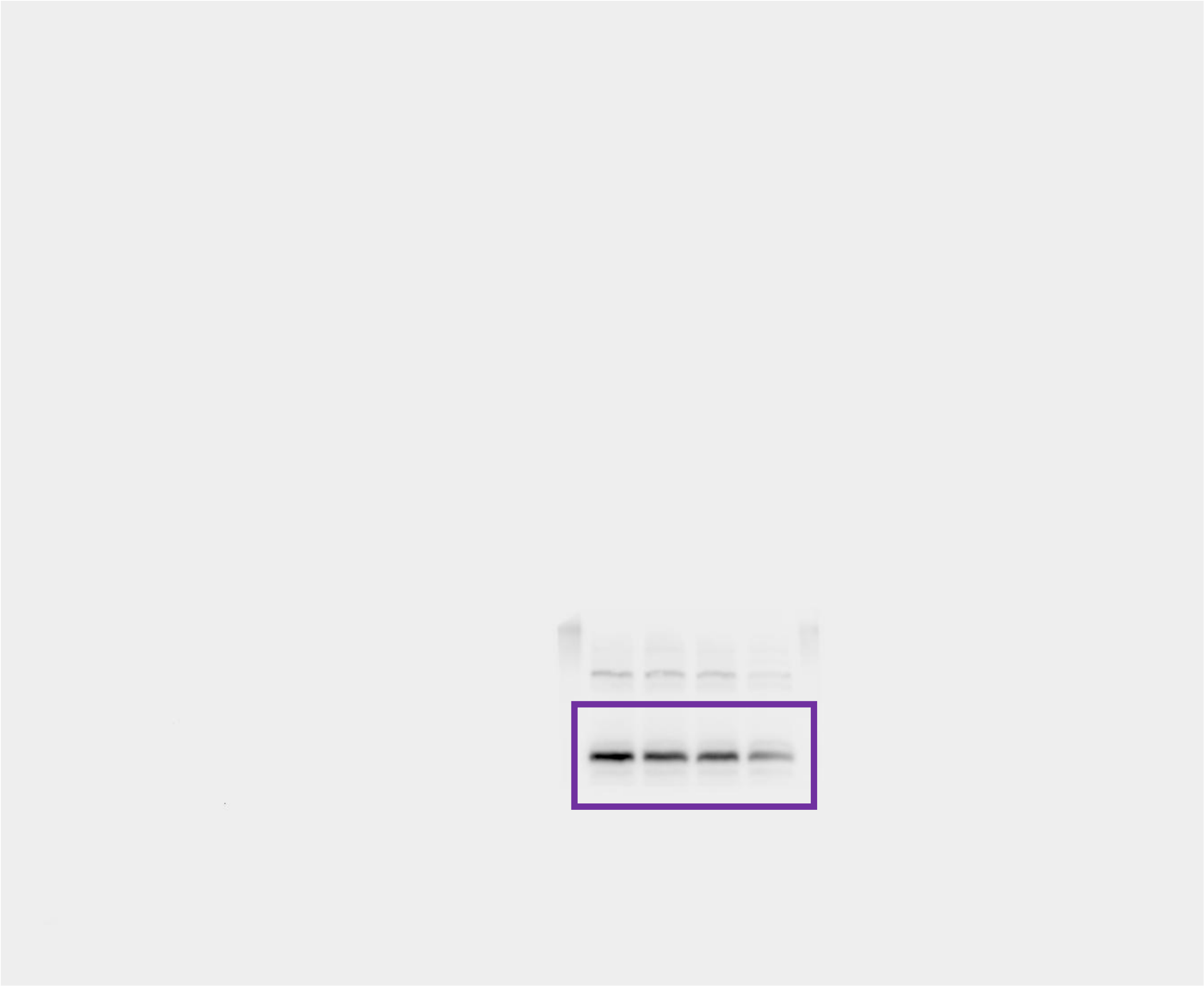

Figure S10C
MLT LC3

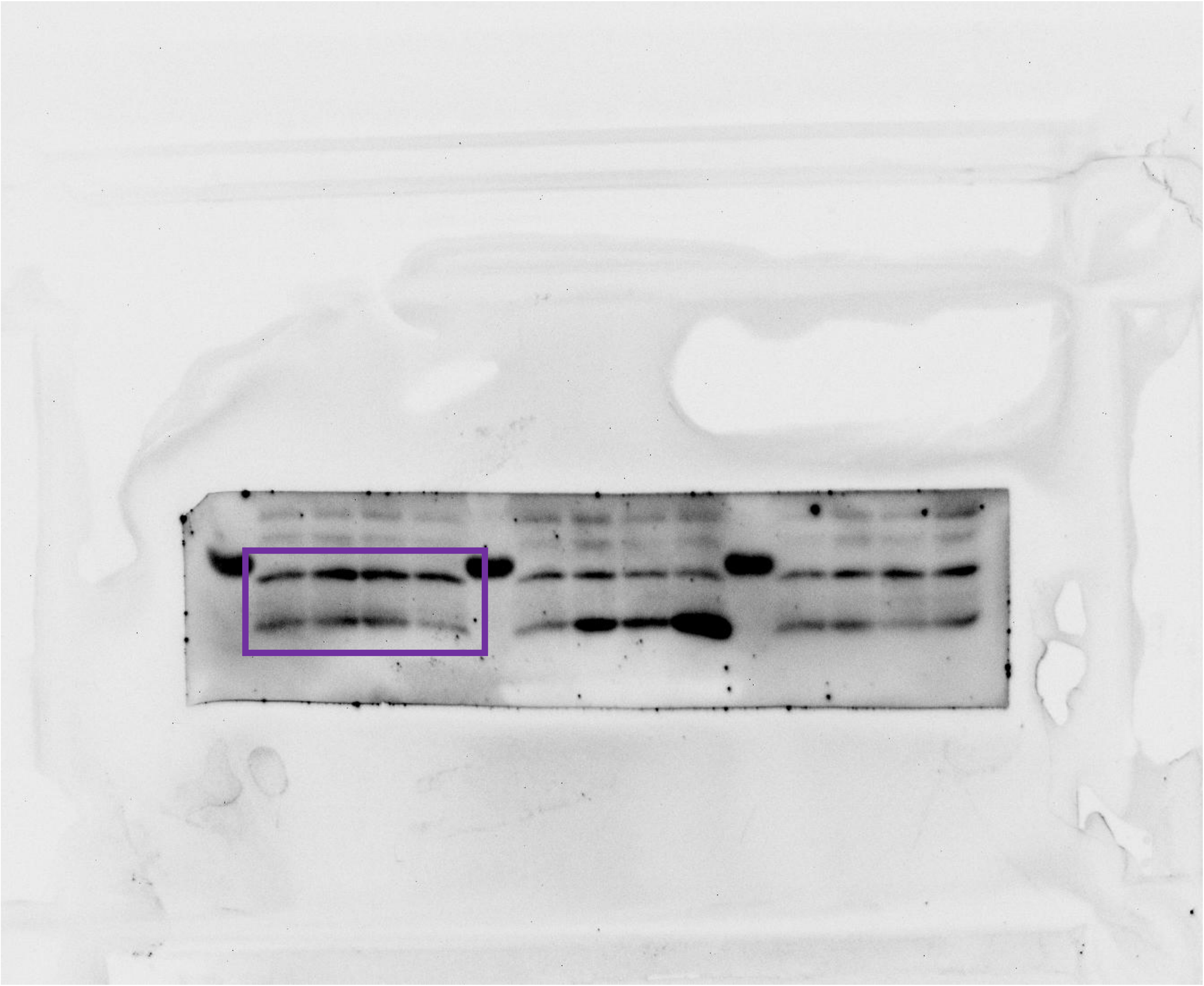

Figure S10C
MLT GAPDH

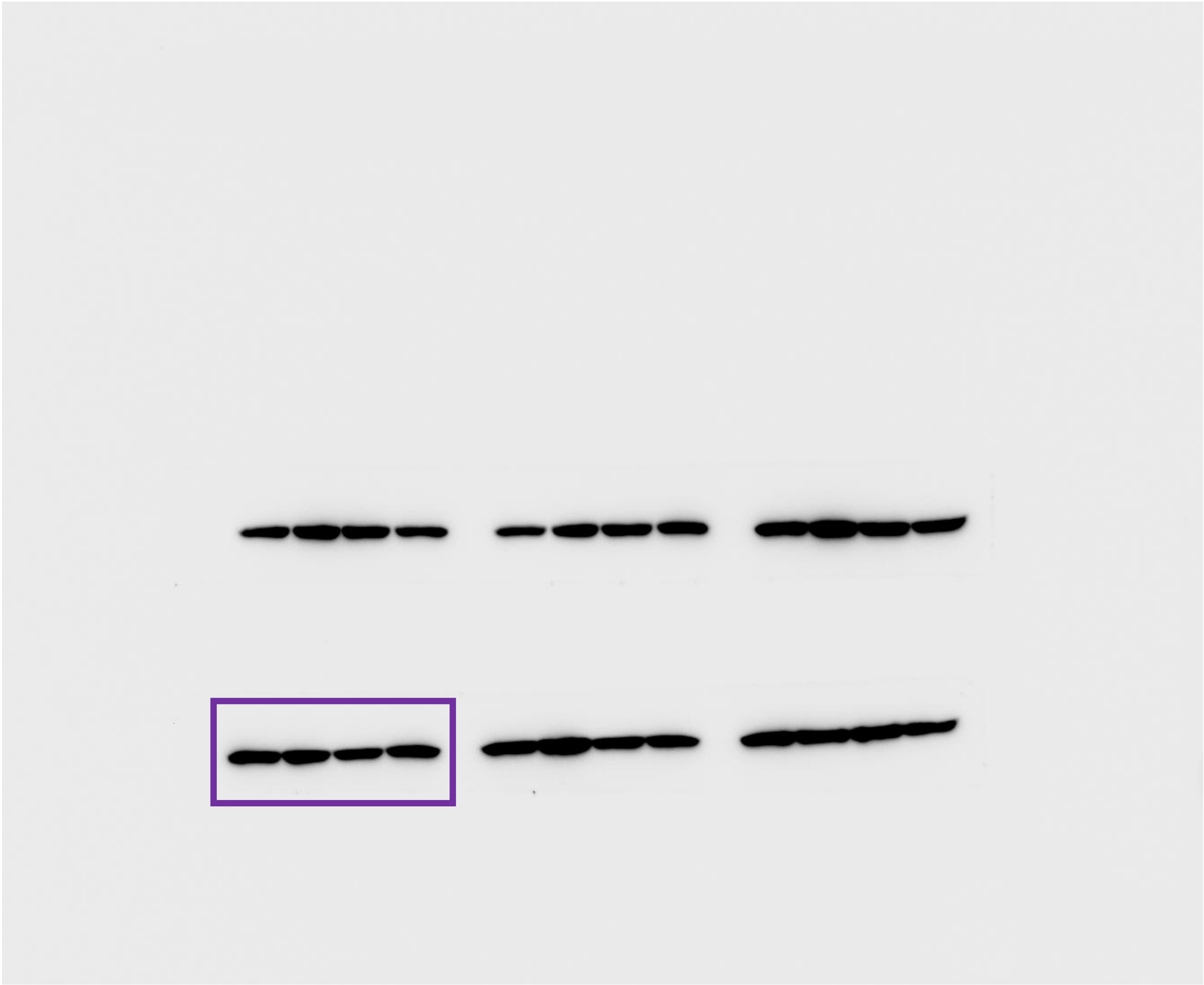

Figure S10D
4PA p62

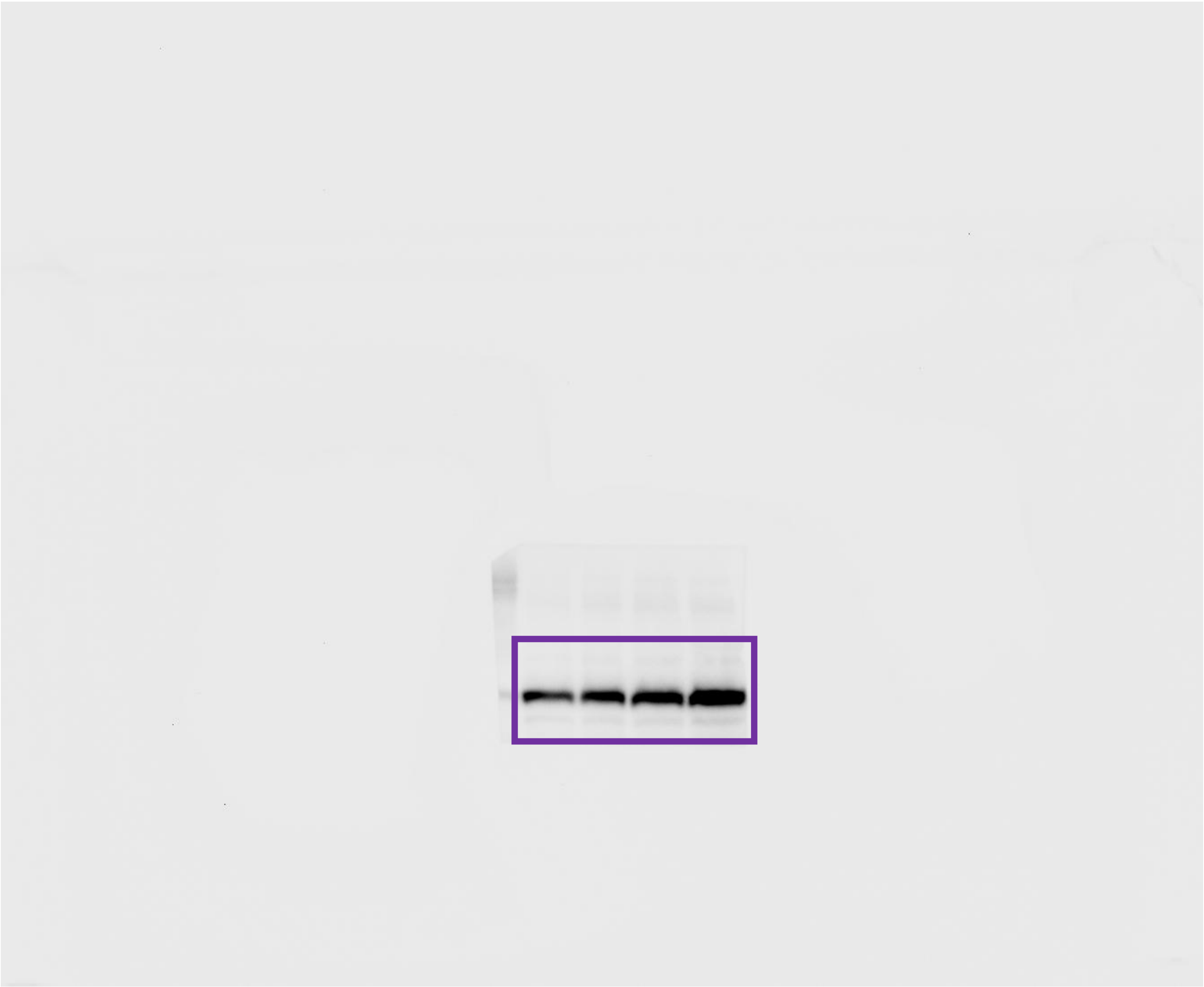

Figure S10D
4PA BECLIN1

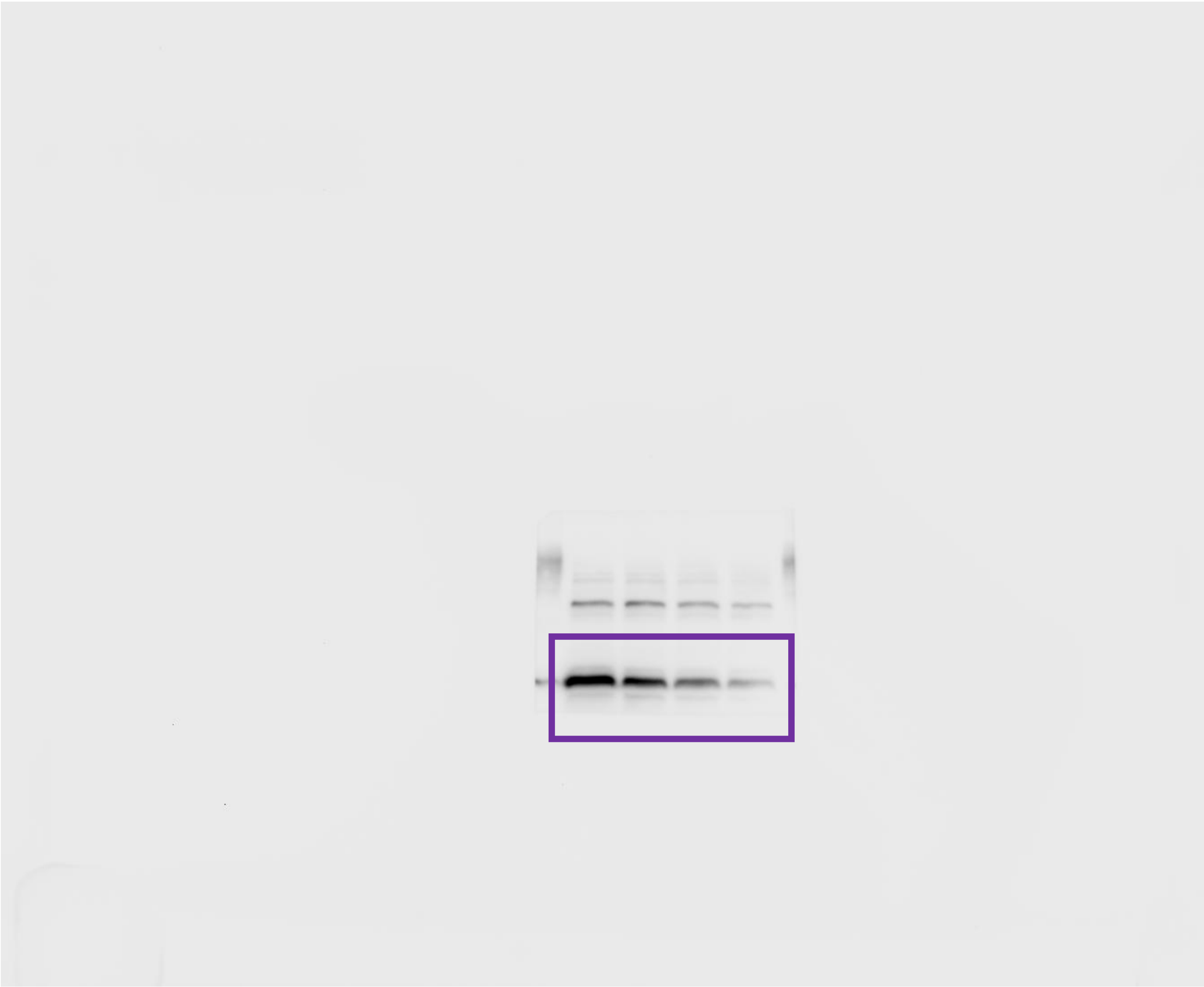

Figure S10D
4PA LC3

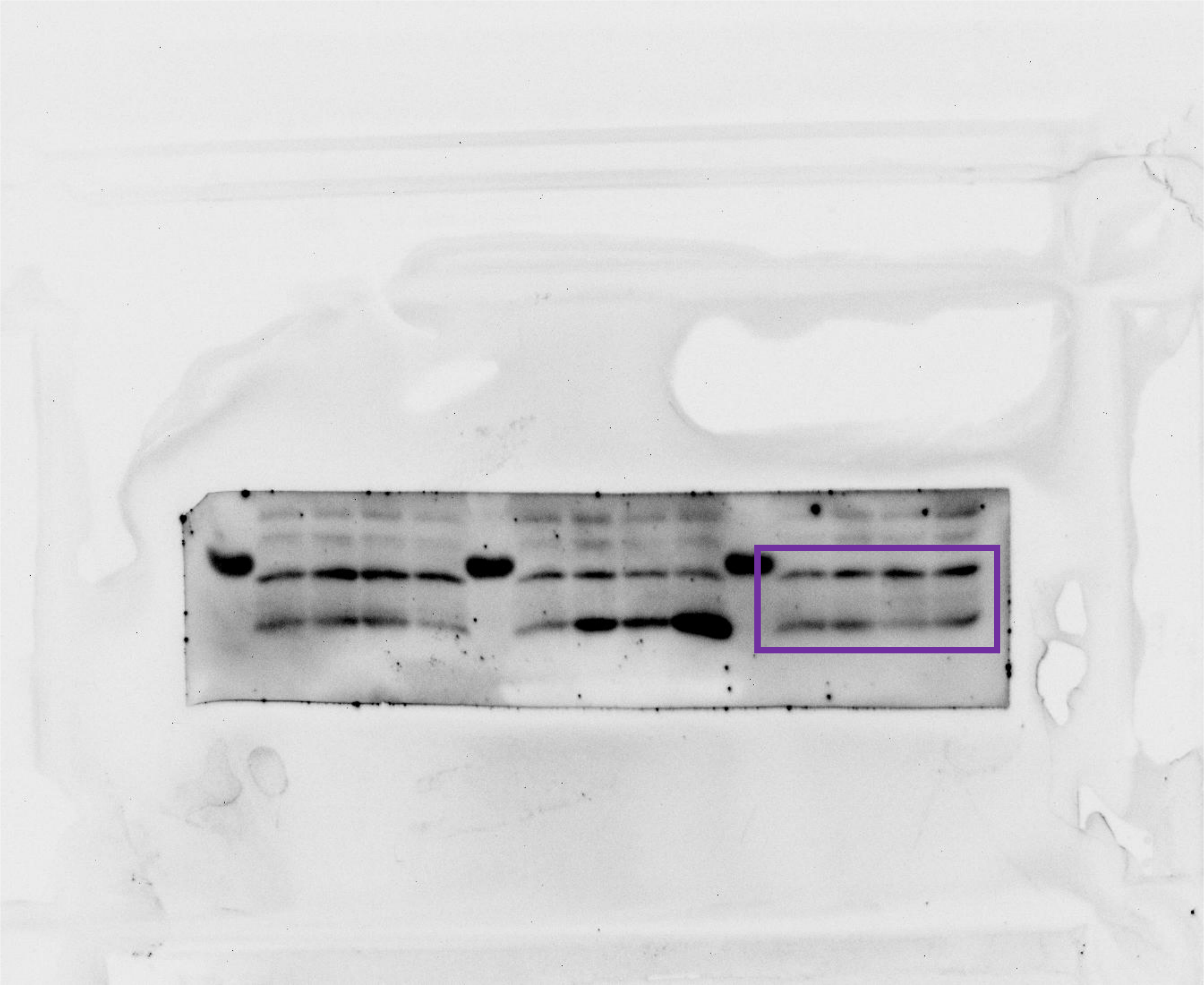

Figure S10D
4PA GAPDH

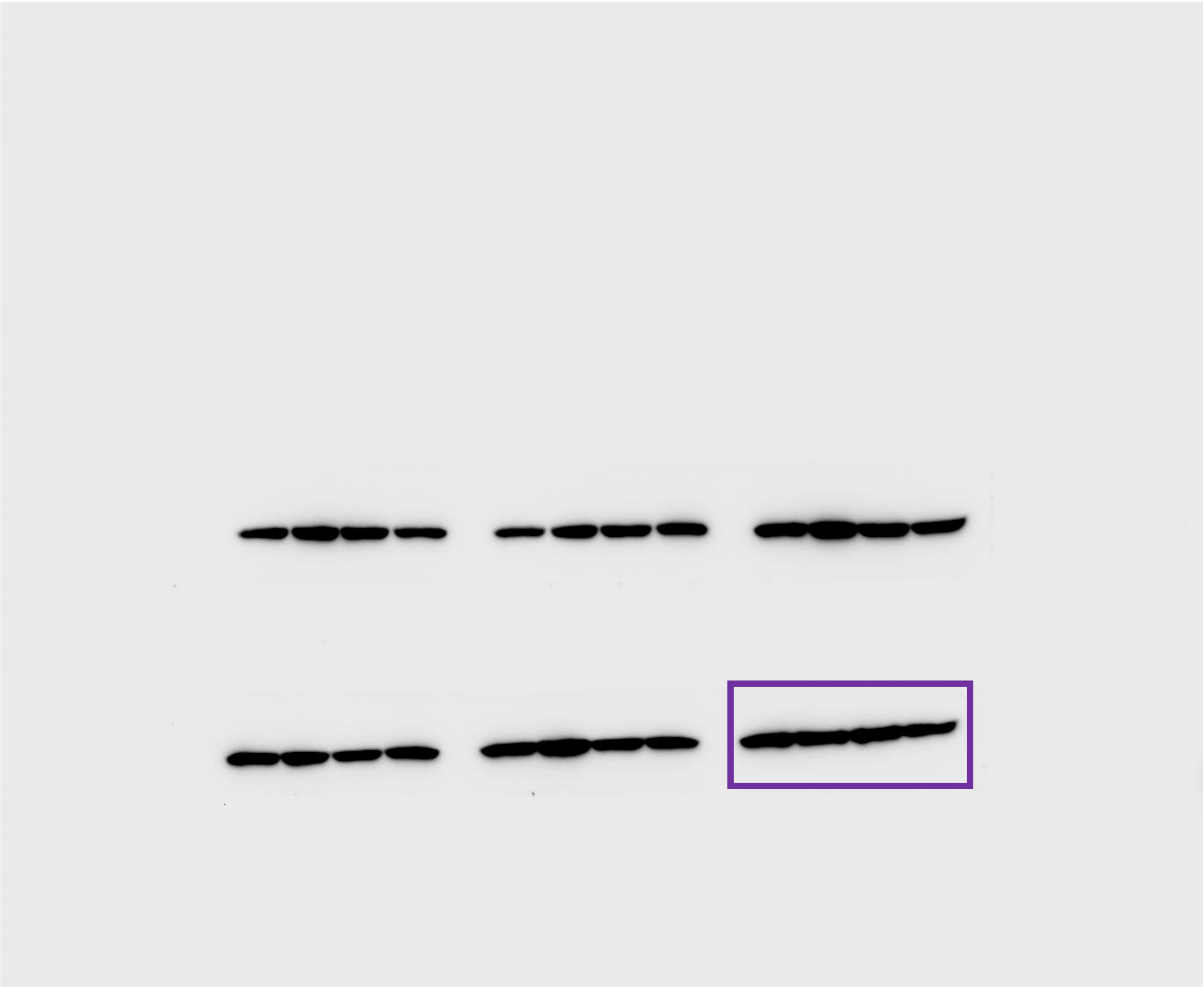

Figure S10E
MLT PINK

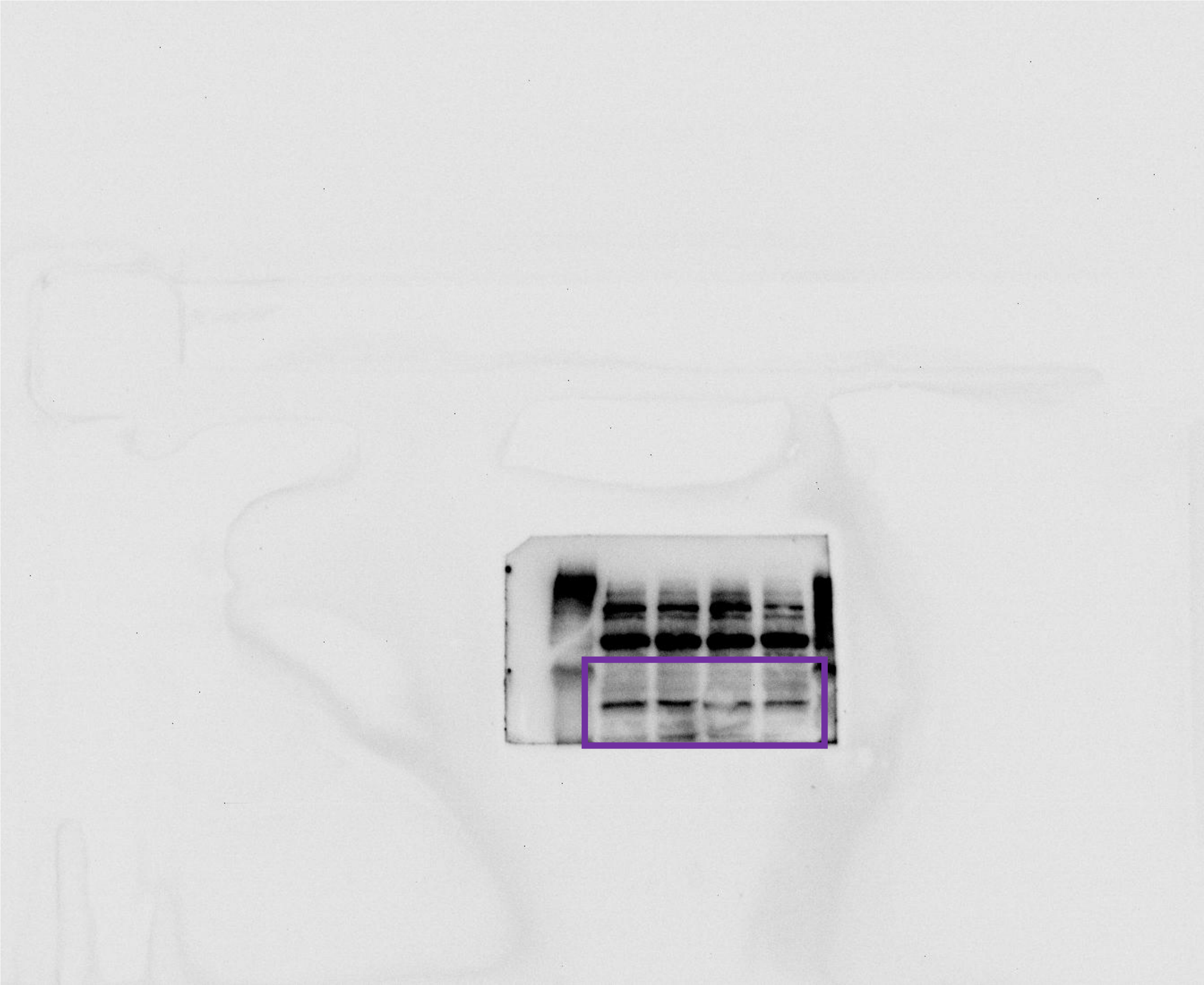

Figure S10E
MLT PARK2

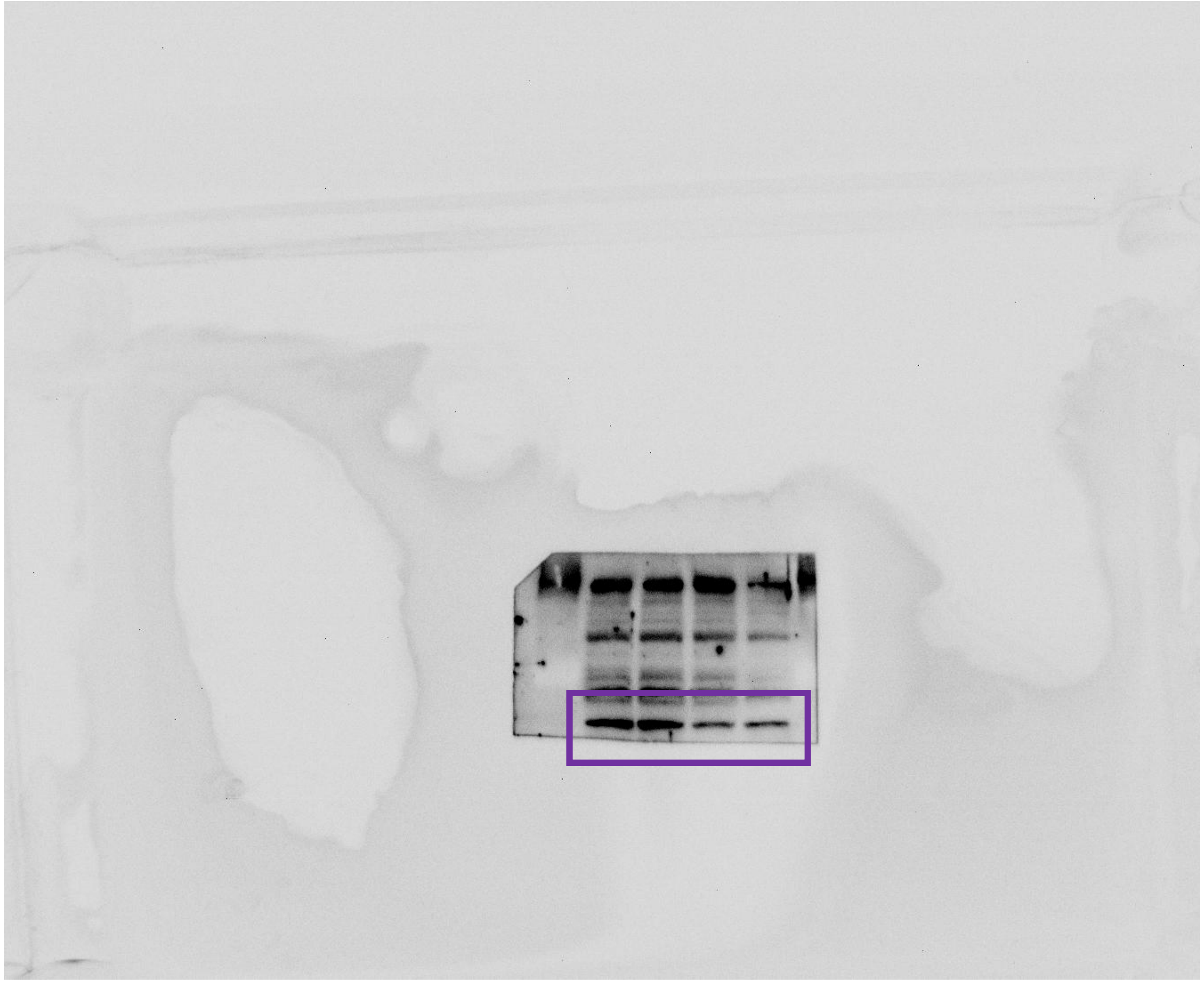

Figure S10E
MLT GAPDH

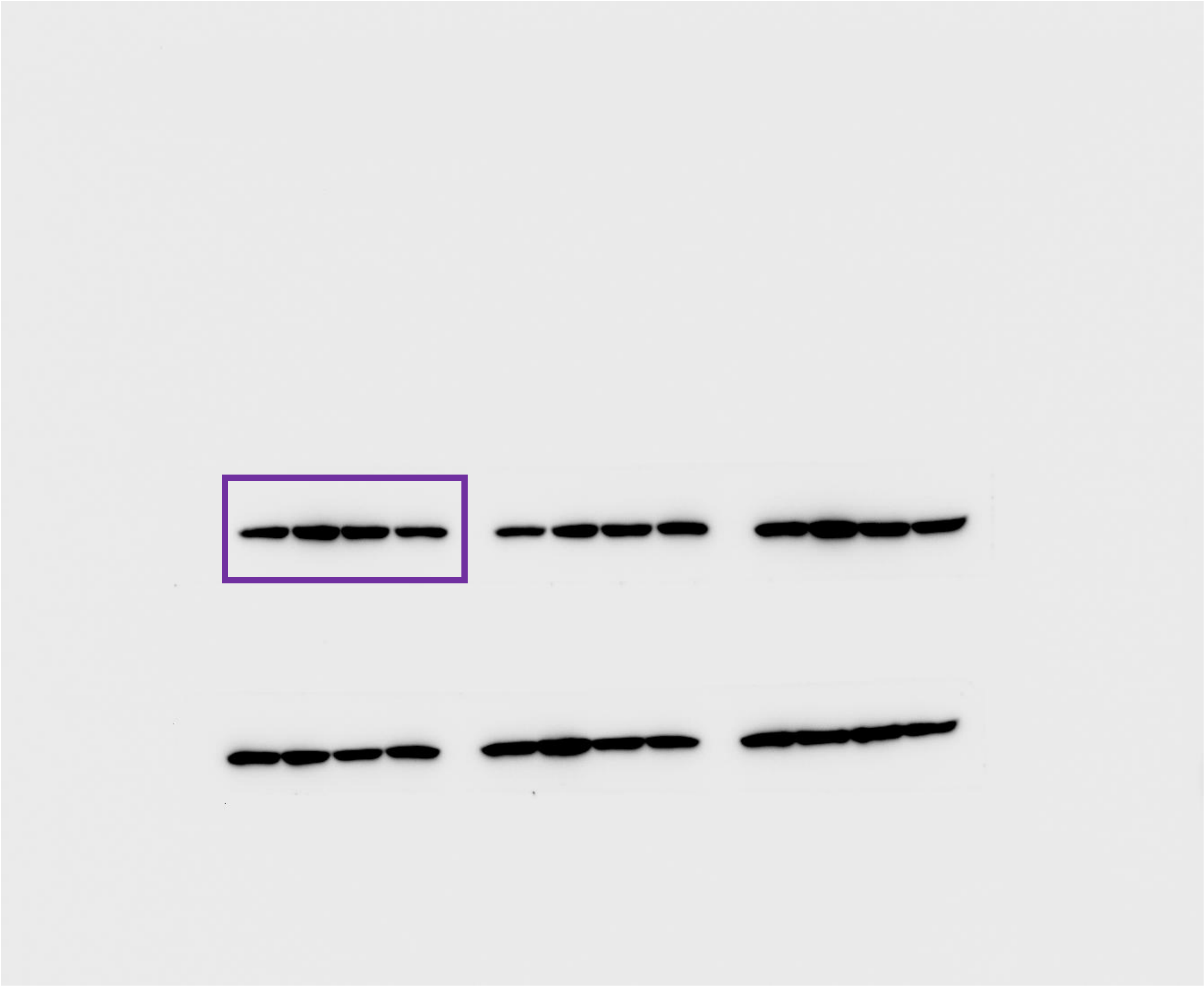

Figure S10F
4PA PINK

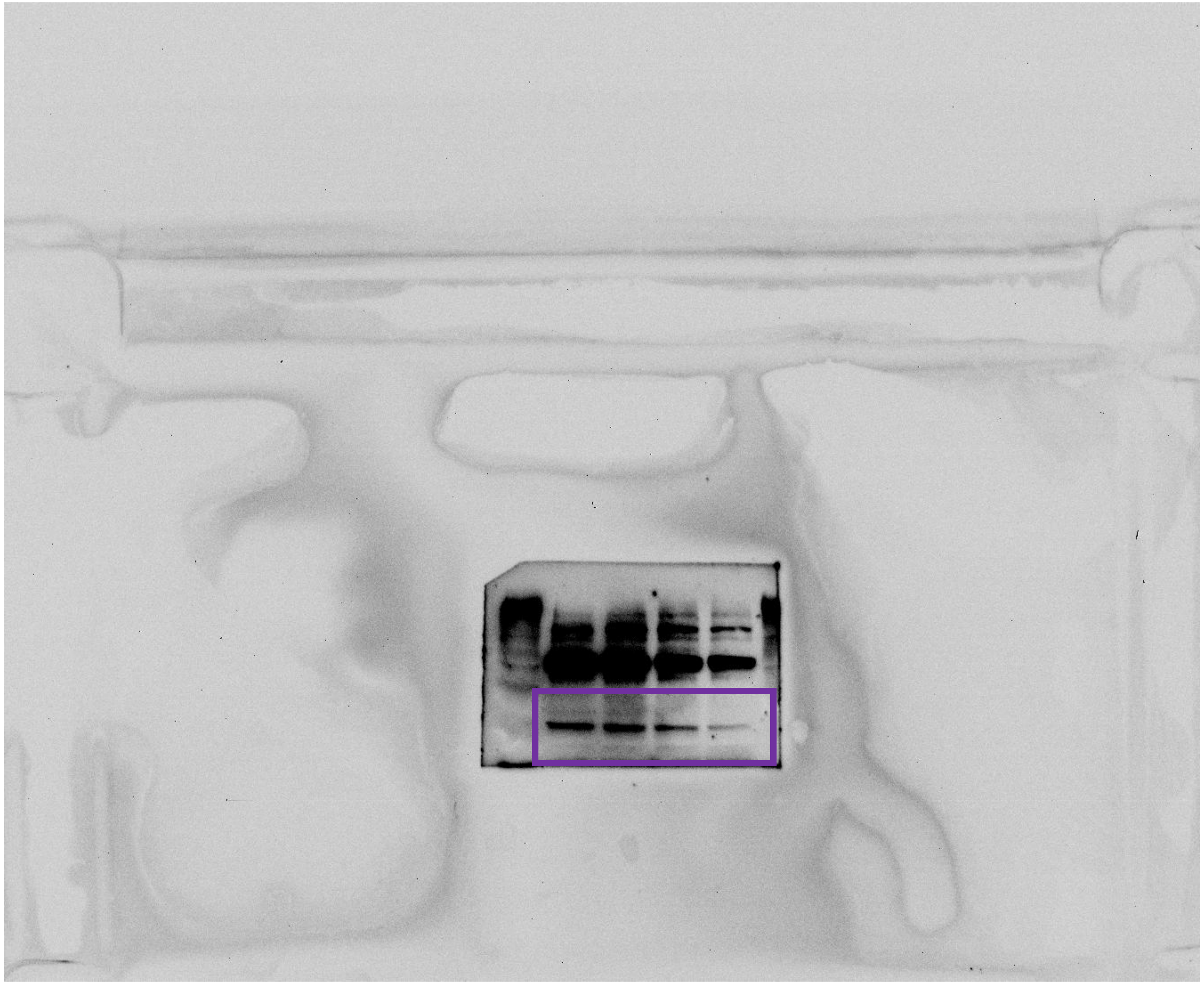

Figure S10F
4PA PARK2

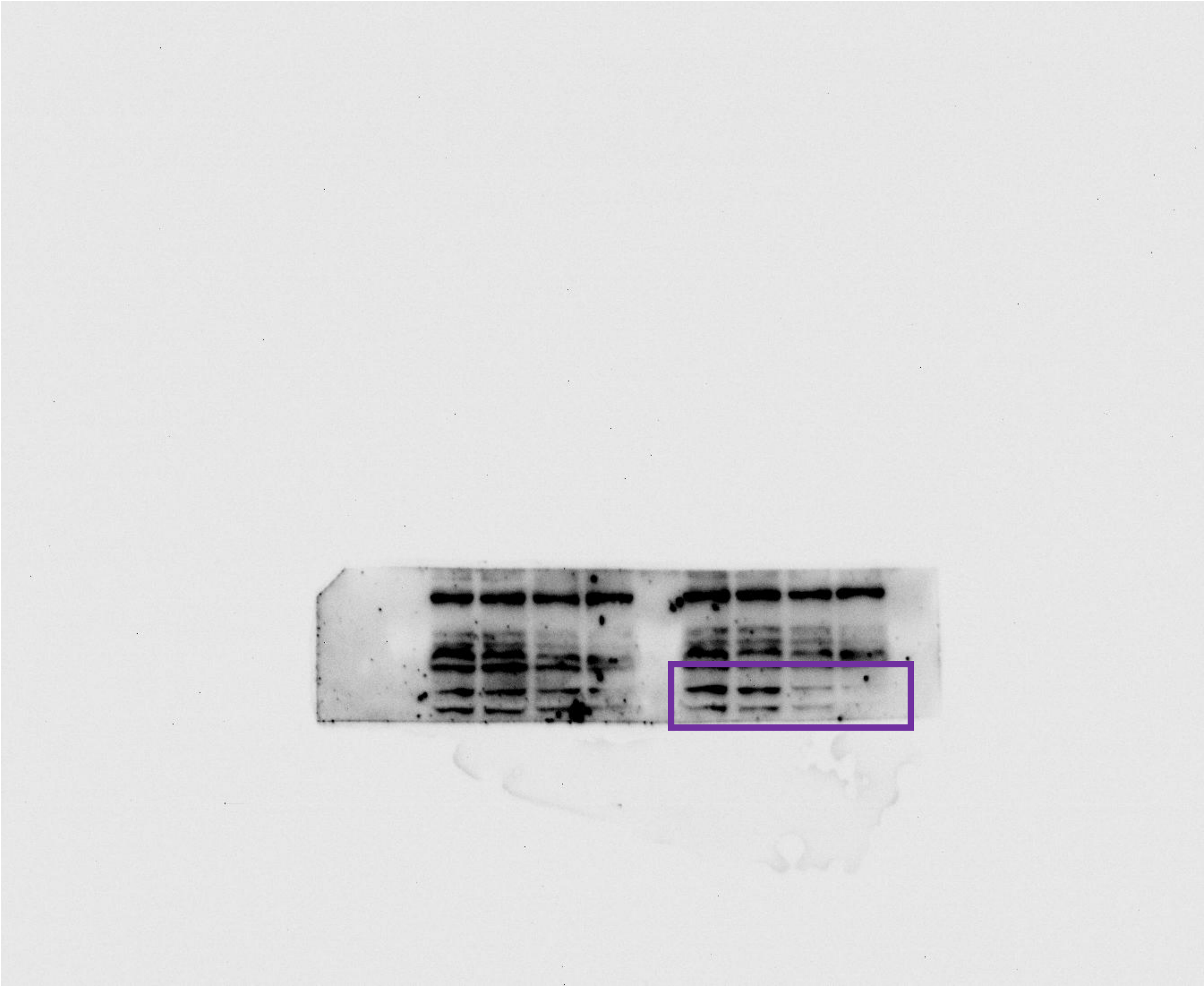

Figure S10F
4PA GAPDH

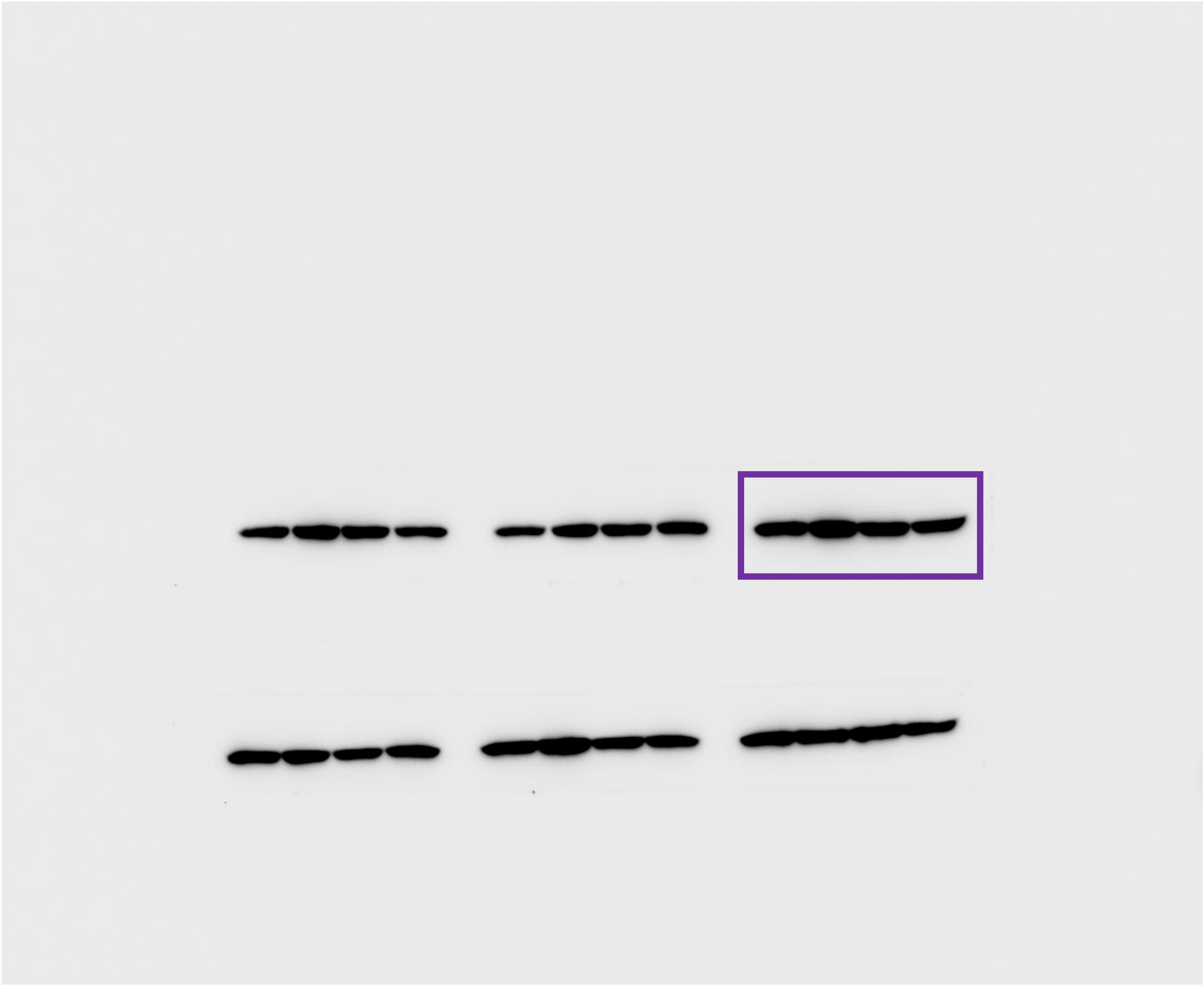

Figure S14B
MHCC97-H FLNA

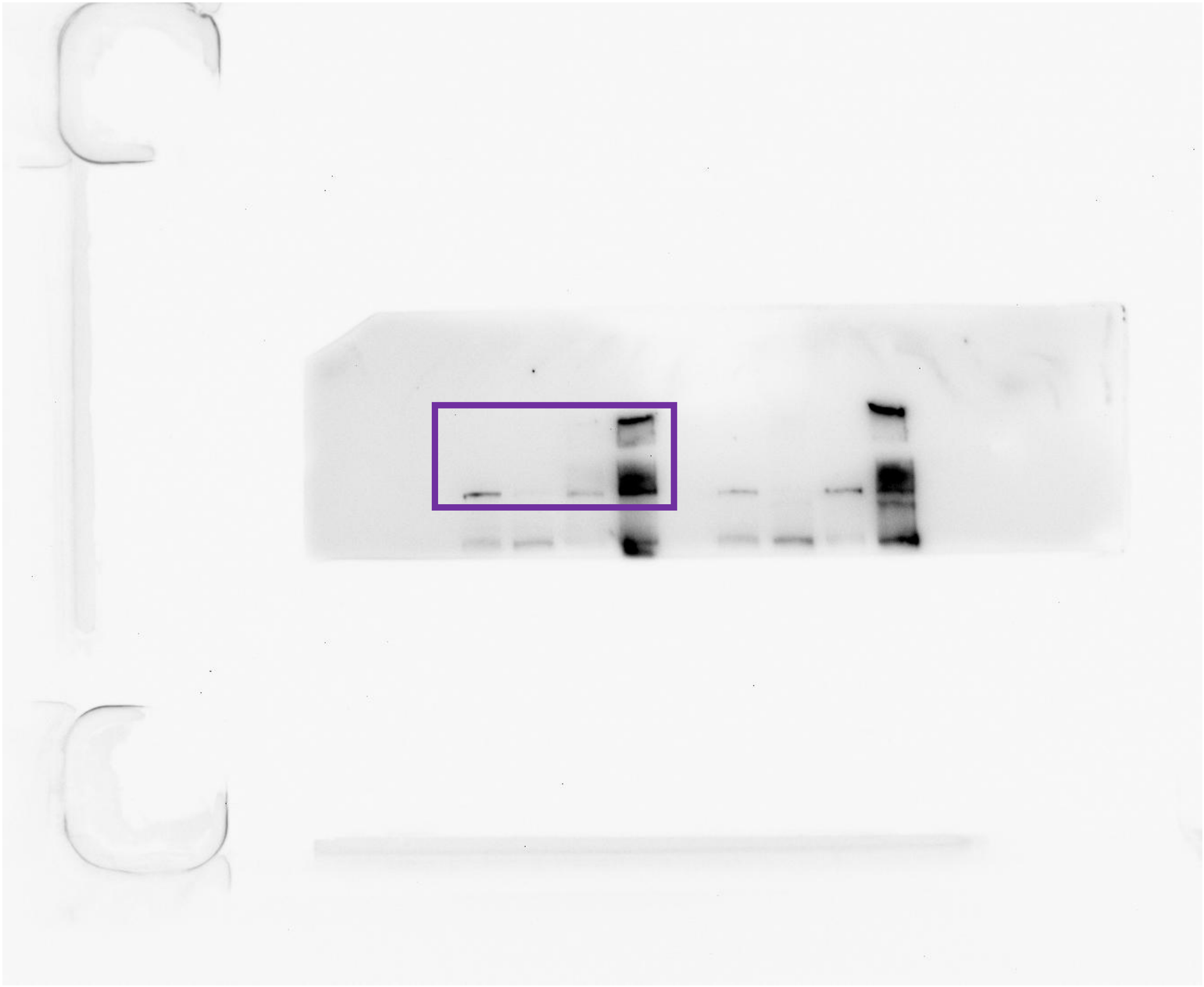

Figure S14B
MHCC97-H DREBRIN

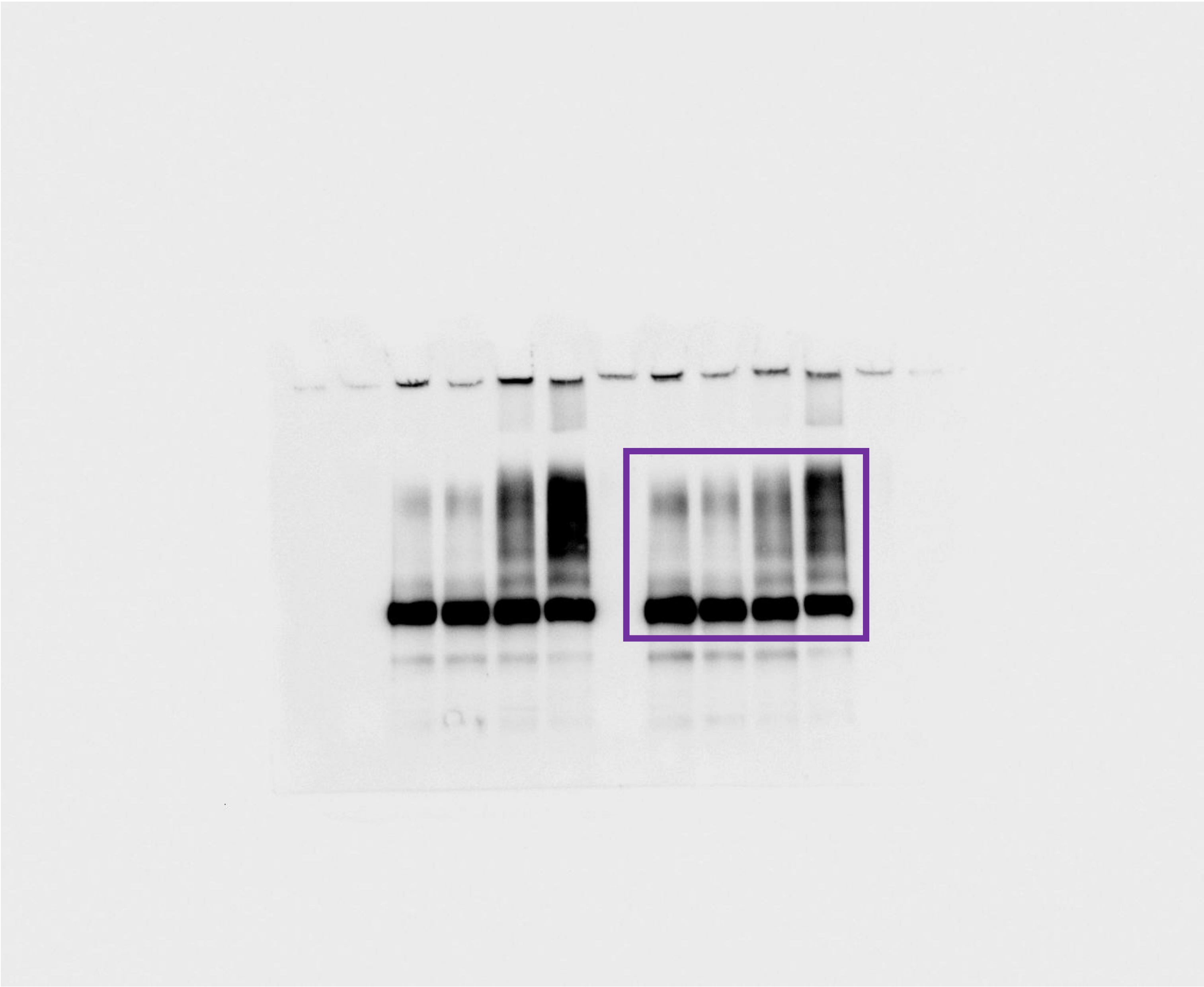

Figure S14B
MHCC97-H GAPDH

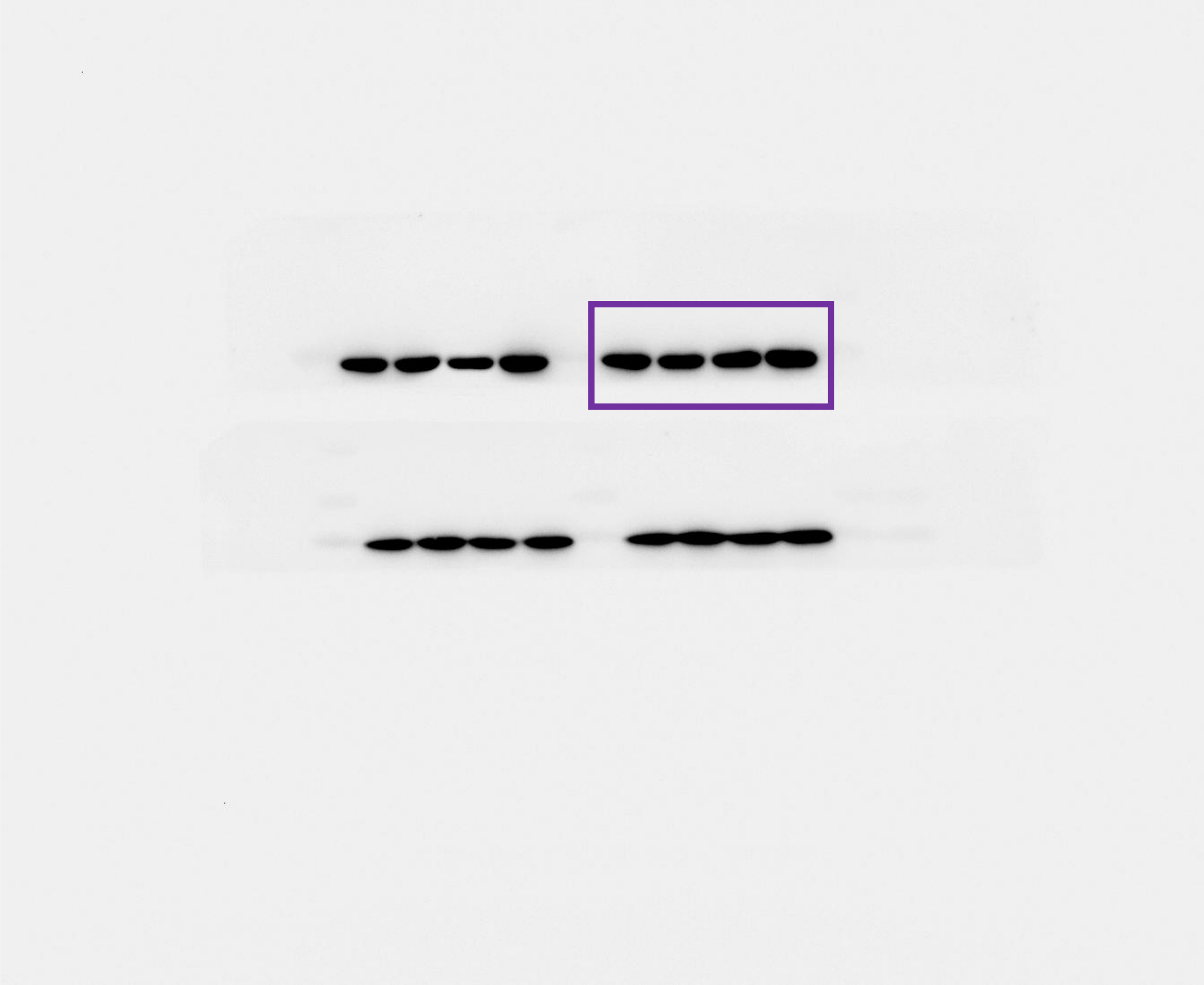

Figure S14B
Hep 3B FLNA

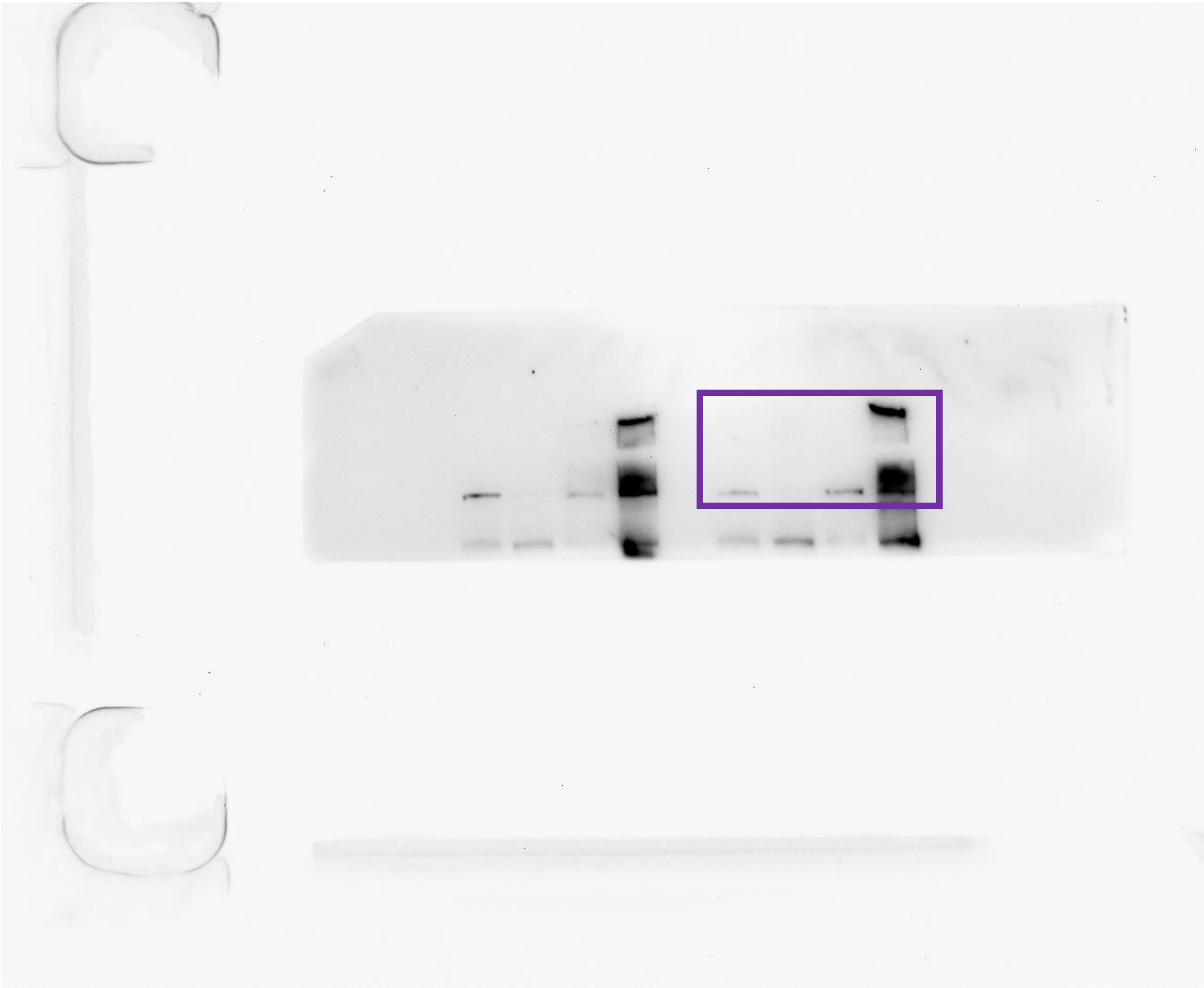

Figure S14B
Hep 3B   DREBRIN

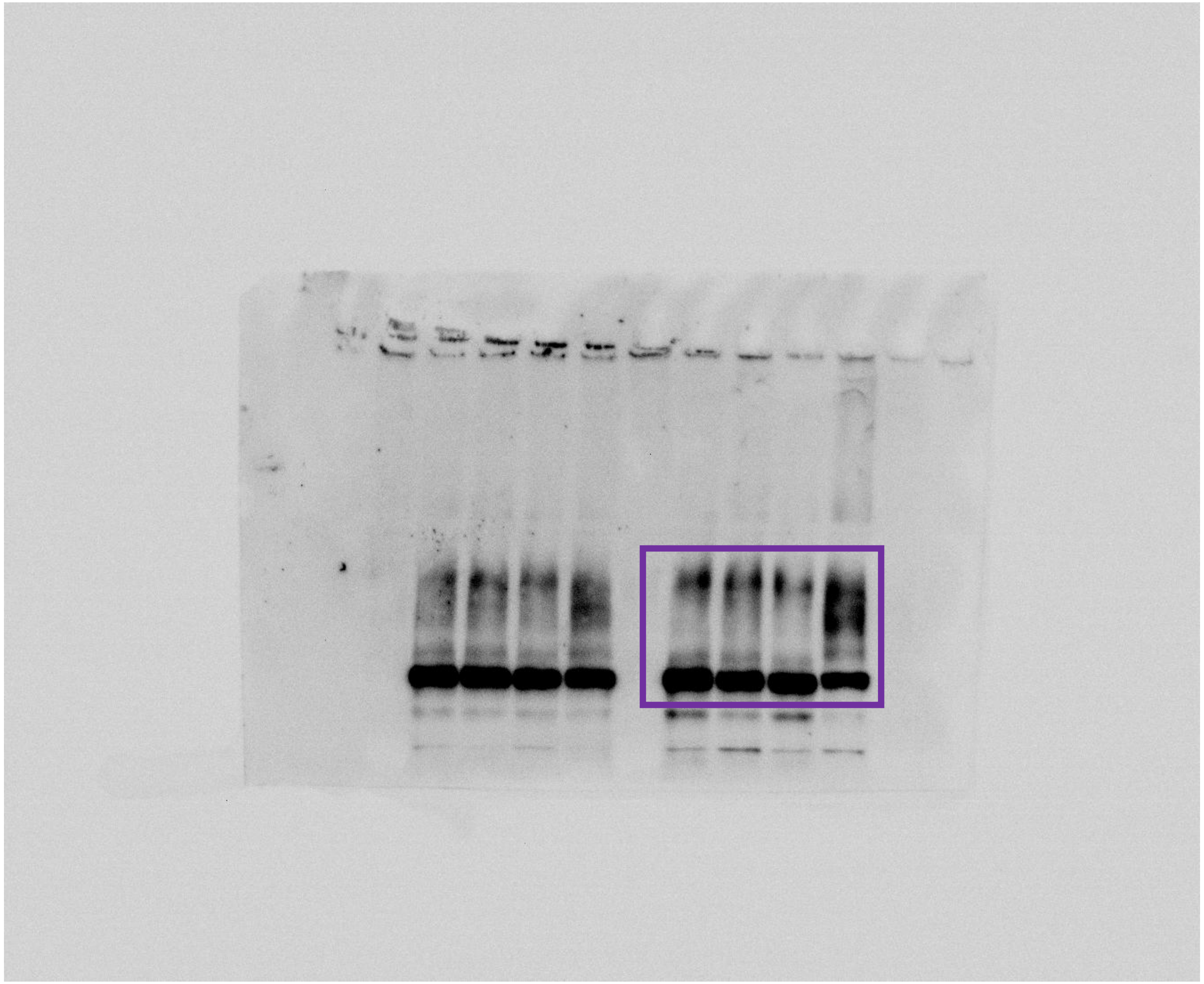

Figure S14B
Hep 3B GAPDH

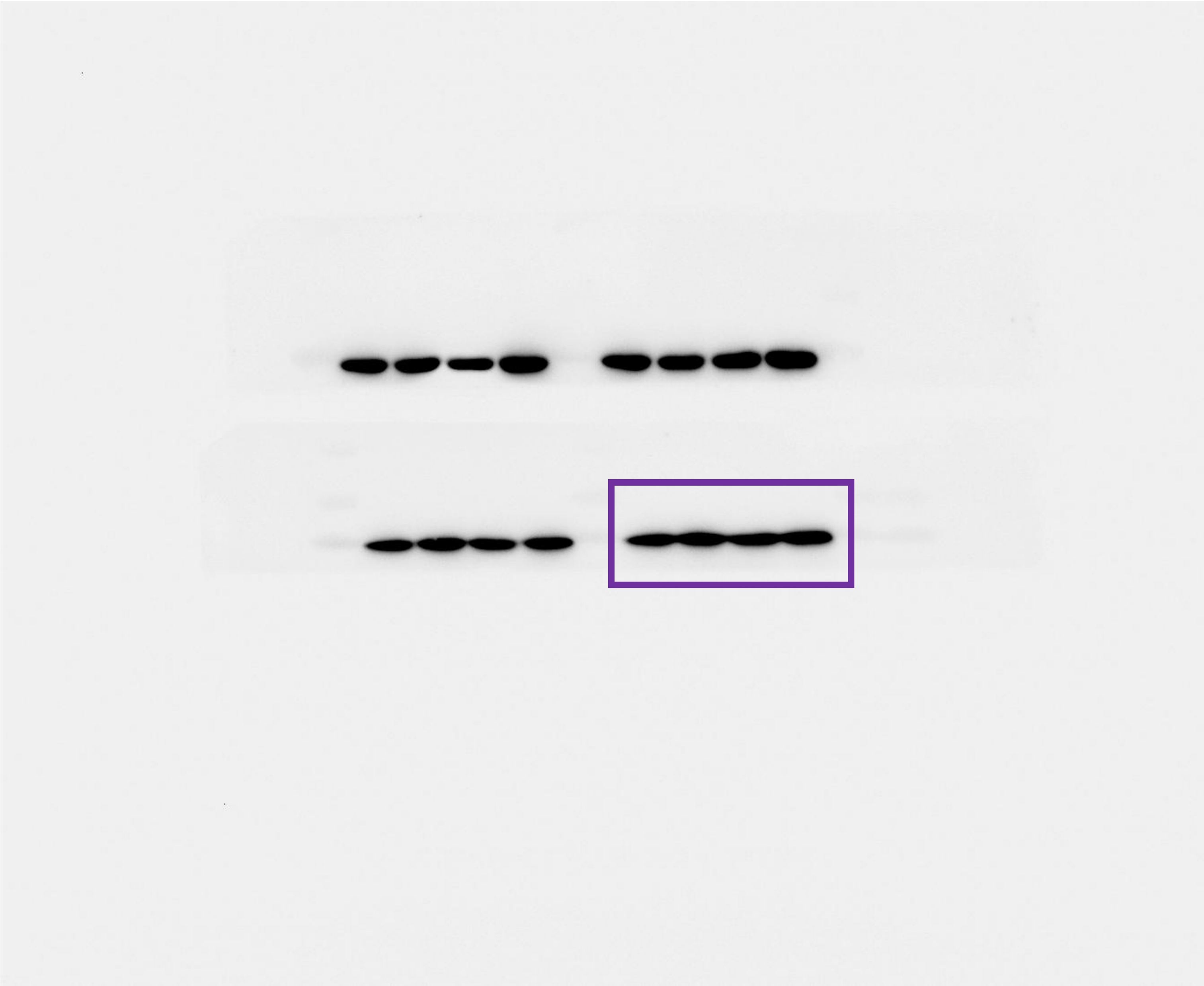

Figure S15D
PINK

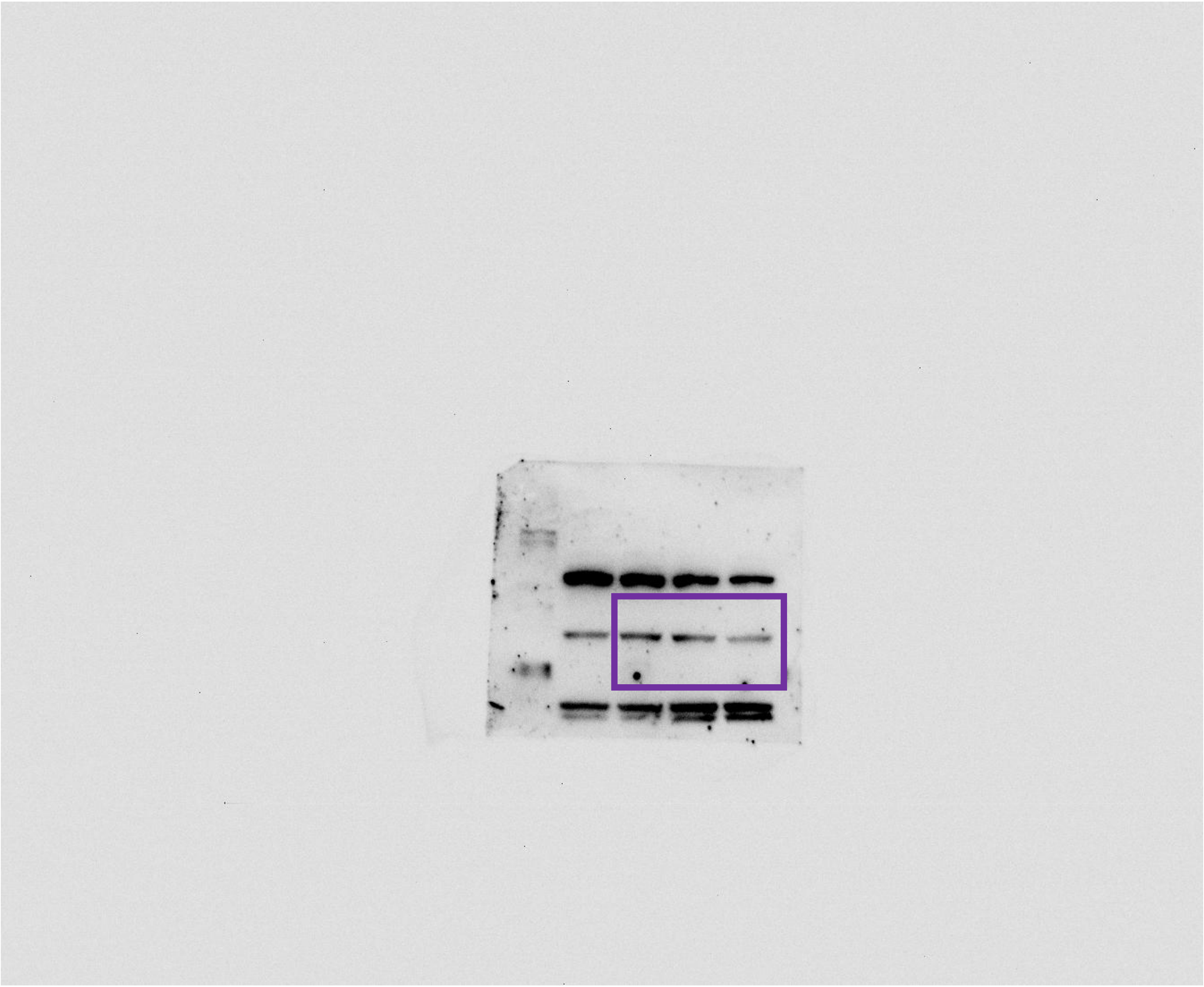

Figure S15D
PARK2

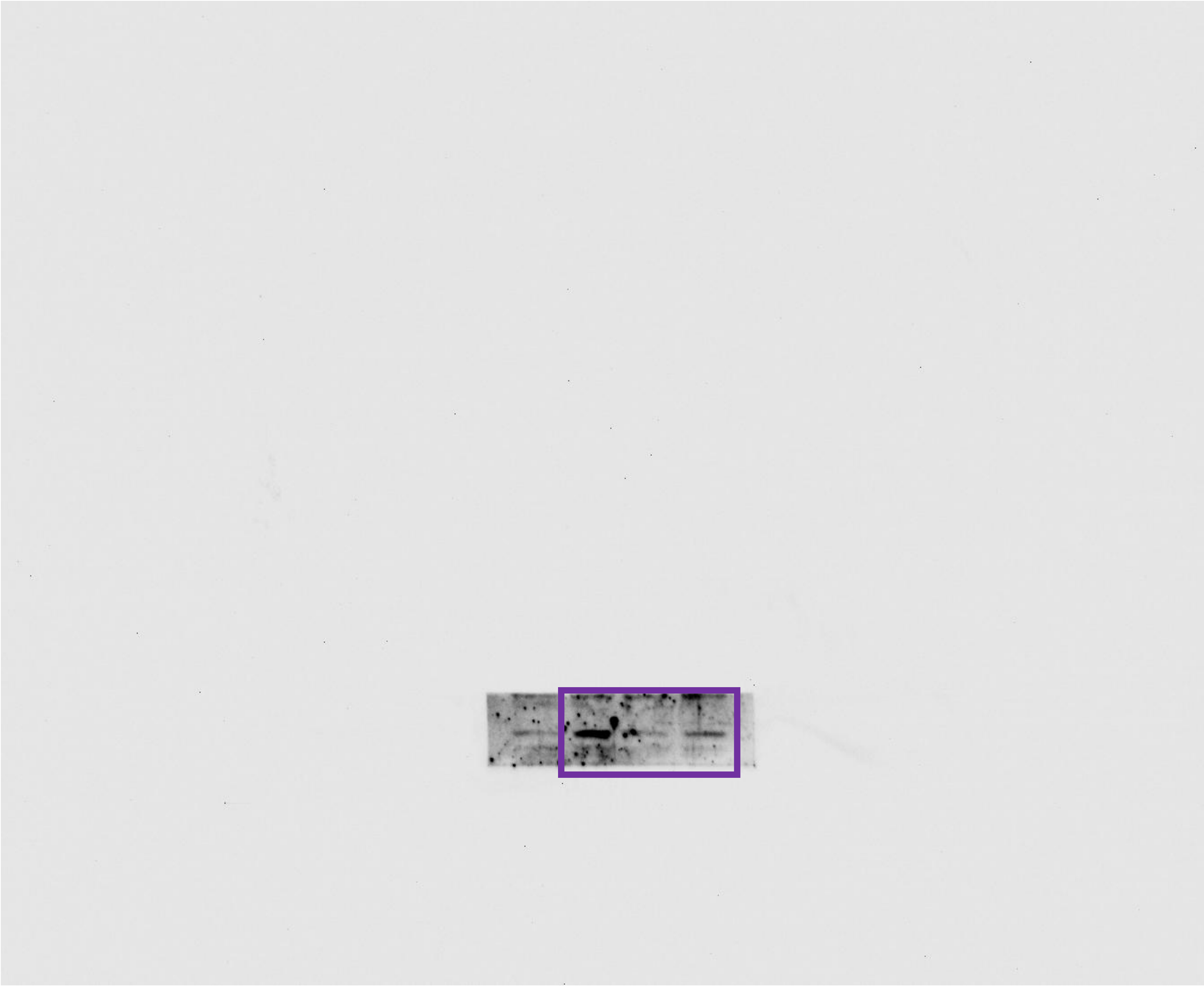

Figure S15D
GAPDH

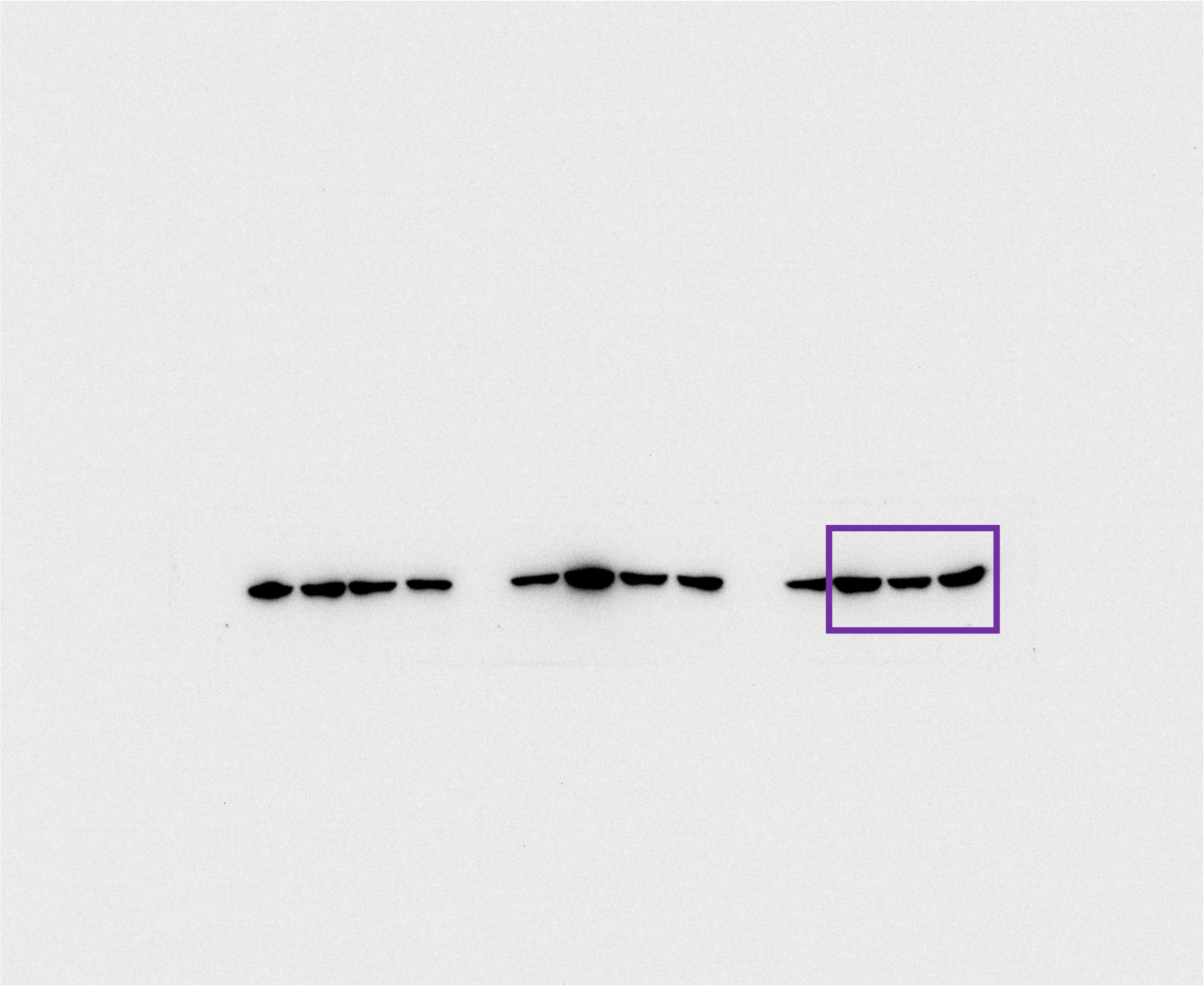

Figure S15D
G6PD

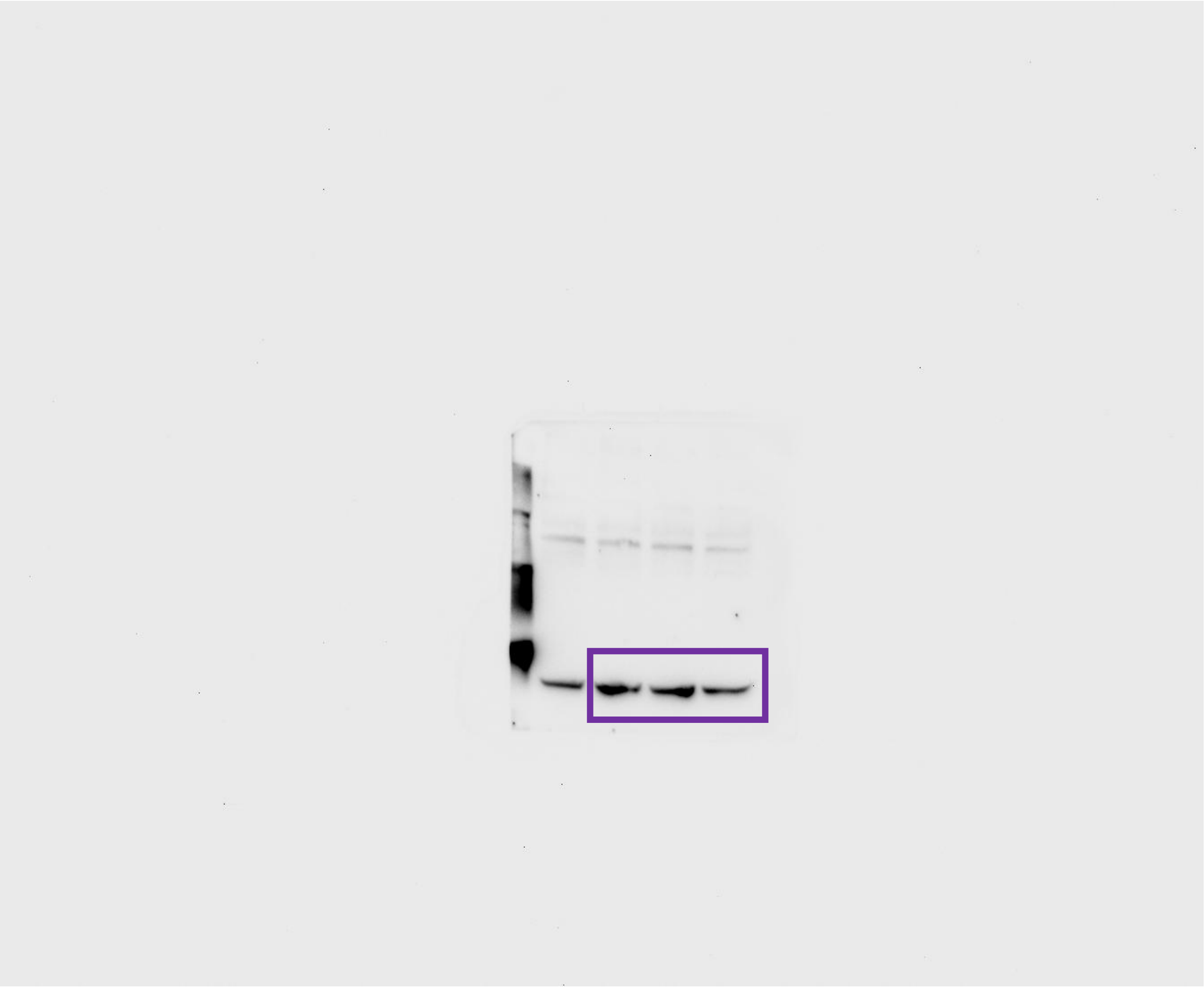

Figure S15D
GAPDH

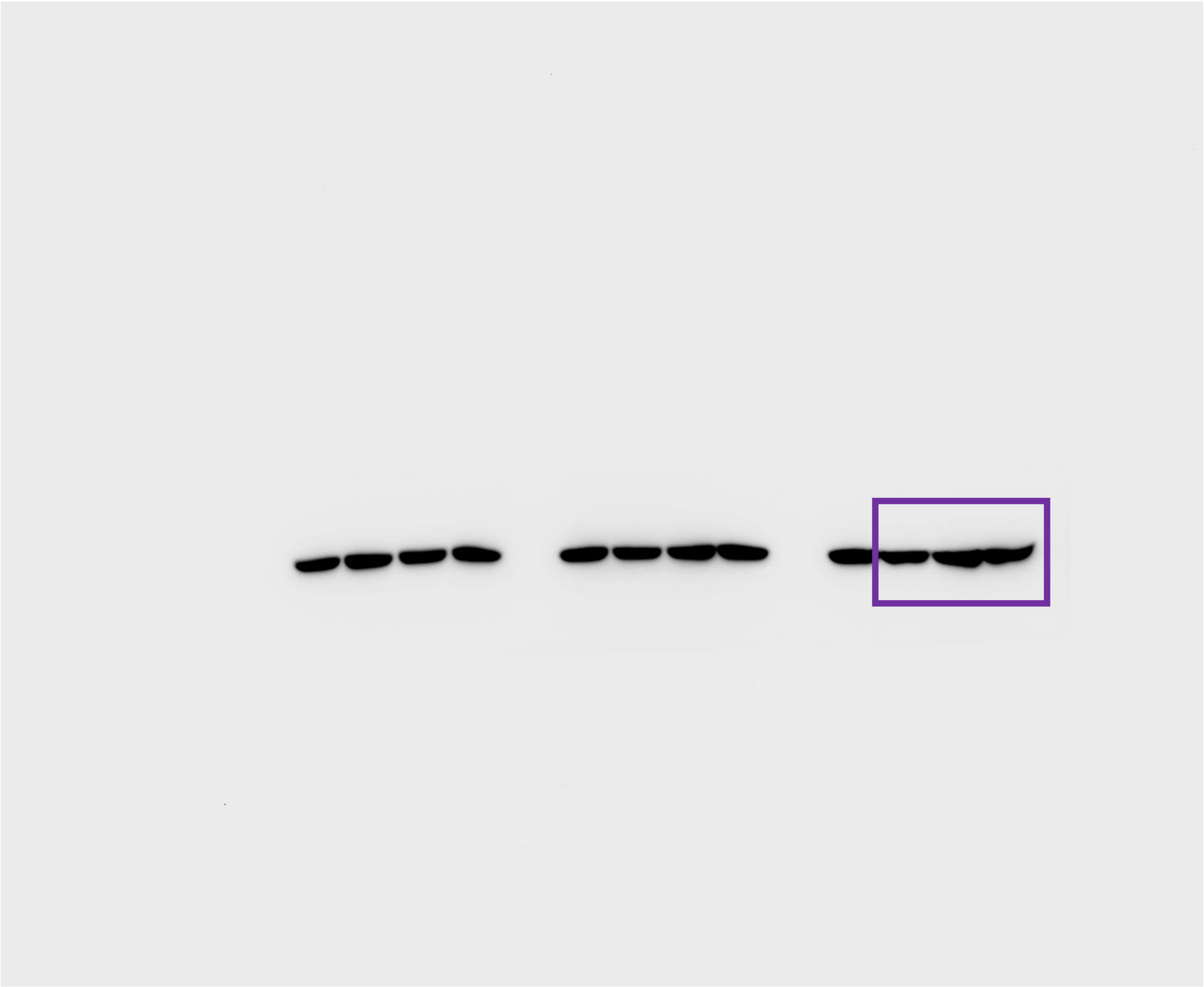

Supplement: Supplementary file 2 [file 41419_2026_9135_MOESM2_ESM.pdf]
